# Redox status, DNA and HSA binding study of naturally occurring naphthoquinone derivatives

**DOI:** 10.17179/excli2019-1859

**Published:** 2020-01-03

**Authors:** Milena D. Vukic, Nenad L. Vukovic, Ana Obradovic, Milos Matic, Maja Djukic, Edina Avdovic

**Affiliations:** 1 Department of Chemistry, Faculty of Science, University of Kragujevac, Radoja Domanovica 12, 34000 Kragujevac, Serbia; 2 Department of Biology and Ecology, Faculty of Science, University of Kragujevac, Radoja Domanovica 12, 34000 Kragujevac, Serbia; 3 Department of Sciences, Institute for Information Technologies Kragujevac, University of Kragujevac, Jovana Cvijića bb, 34000 Kragujevac, Serbia

**Keywords:** naphthoquinone derivatives, redox status, DNA interactions, HSA interactions, colon cancer, breast cancer

## Abstract

In the present work we modified the procedure for isolation of naphthoquinones α-methylbutyrylshikon (***1***), acetylshikonin (***2***) and *β*-hydroxyisovalerylshikonin (***3***) from *Onosma visianii* Clem. We also investigated possible mechanisms of ***1***, ***2*** and ***3*** as antitumor agents. Accordingly, we estimated concentrations of superoxide anion radical (O_2_^.-^), nitrite (NO_2_^ -^) and glutathione in HCT-116 and MDA-MB-231 cell lines. Compounds ***1*** and ***3*** expressed significant prooxidative activity, while all tested compounds exhibited significant increase in nitrite levels. Also, all examined compounds significantly increased the concentration of oxidized glutathione (GSSG), suggesting significant prooxidative disbalance. The levels of reduced glutathione (GSH) were also elevated as a part of antioxidative cell response. The data indicate that induced oxidative imbalance could be one of the triggers for previously recorded decreased viability of HCT-116 and MDA-MB-231 cells exposed to tested naphthoquinone derivatives. Moreover, we examined interactions mode of compounds ***1***, ***2*** and ***3*** with CT-DNA as one of the crucial targets of many molecules that express cytotoxic activity. The results obtained by UV-visible, fluorescence and molecular docking study revealed that ***1***, ***2*** and ***3*** bound to CT-DNA through minor groove binding. Furthermore, the interactions between HSA and ***1***, ***2*** and ***3*** were examined employing the same methods as for the CT-DNA interaction study. Based on the obtained results, it can be concluded that naphthoquinones ***1***, ***2*** and ***3*** could be effectively transported by human serum albumin. As a conclusion, this study provides further insight of antitumor activity of selected naphthoquinones.

## Introduction

According to the World Health Organization, cancer is one of the major causes of mortality. Radical approaches as surgery, radiotherapy or chemotherapy are not efficient in therapy of most cancers. Therefore, discovering novel, effective anticancer drugs is currently in focus of many investigations (Wellington, 2015[50]). The colorectal and breast cancers are among the leading causes of cancer death (Stewart and Wild, 2014[44]). Breast cancer is one of the most common cancer types and represents the leading cause of mortality among women worldwide (Murad et al., 2016[30]). Colorectal cancer, the main cause of death in gastrointestinal cancers, is the third most common cancer worldwide in men and women and the second largest cause of death related to cancer. Risk factors implicated in the etiology of this type of cancer are related to bad alimentary habits, cigarette smoking, low physical activity, inflammatory bowel disease, polyps, genetic factors, and aging (Granados-Romero et al., 2017[16]).

Cell metabolism and survival are strongly dependent on the amount of different oxidants present in cytoplasm. Reactive oxygen species (ROS), produced during mitochondrial respiratory chain activity, are implicated in several physiological process in all aerobic organisms (Cano et al., 2014[6]). Oxidative stress represents an imbalance between the production of these aggressive radicals and their neutralization through compensating antioxidative mechanisms. This imbalance can lead to multiple cellular damages and development of many pathophysiological conditions, including cancer (Jie et al., 2013[20]). ROS induce carcinogenesis through genetic and epigenetic mechanisms. Elevated levels of ROS detected in numerous tumors strongly implicate the role of DNA damage caused by different oxidizing molecules in promoting tumor development and progression (Liou and Storz, 2010[25]). 

Deoxyribonucleic acid (DNA) and human serum albumin (HAS) are important biomacromolecules. DNA is the major cellular target for many therapeutic agents, including plant secondary metabolites. Understanding DNA-drug interactions has become an active research area as binding compounds have potential applications as anti-cancer agents. Small molecules can bind to DNA double helix through three binding modes: electrostatic, intercalation and groove binding (Sirajuddin et al., 2013[43]). Essential to understanding of these interactions, besides the characterization of the binding modes, is determination of strength of binding. Apprehension of aforementioned parameter can lead to design of new and more effective drugs that can target specific site or conformation of DNA. Human serum albumin (HSA) is the key protein in the blood plasma, the crucial soluble protein in the circulatory system. Likewise, HSA plays the main role in bioavailability, distribution and elimination of several biologically active moieties (drugs, toxins, natural products, etc.) from the body. Considering the importance of human serum albumin and deoxyribonucleic acid, studying their interaction with bioactive compounds may provide valuable information on their therapeutic abilities. 

Naphthoquinones, naturally occurring pigments, are secondary metabolites of many species belonging to *Boraginaceae* family, including *Onosma* genus. Previous chemical investigations showed that naphthoquinones belonging to class of shikonin or alkannin derivatives are concentrated in the roots of genus* Onosma *(Cadirci et al., 2007[5]; Özgen et al., 2004[32]; Sagratini et al., 2008[36]; Vukic et al., 2018[48]). These naphthoquinones are responsible for valuable ethnopharmacological usage of these plants in treatments of various ailments (Sezik et al., 1997[38]; Davis, 1988[11]). They exhibit a wide spectrum of biological properties, including antitumor activity (Papageorgiou et al., 1999[34]; Wang et al., 2015[49]; Vukic et al., 2017[47]; Kretschmer et al., 2012[23]). Currently, cytotoxic properties of the naphthoquinones are in the focus of interest of many scientists, and it is already suggested that they are mainly based on their ability to generate reactive oxygen species (ROS) (Papageorgiou et al., 1999[34]; Duan et al., 2014[13]; Gong and Li, 2011[15]; Shahsavari et al., 2015[39]). 

Our previous studies showed that roots of *O. visianii* are rich sources of naphthoquinones and also provided a strong indication of the potential use of α-methylbutyrylshikon (***1***), acetylshikonin (***2***) and *β*-hydroxyisovalerylshikonin (***3***) as cytotoxic agents on human colon cancer HCT-116 and human breast cancer MDA-MB-231 cell lines (Vukic et al., 2017[47]). Considering that, in this study we determined effects on redox status of α-methylbutyrylshikonin (***1***), acetylshikonin (***2***) and *β*-hydroxyisovalerylshikon (***3***) on colon cancer cell line HCT-116 and human breast cancer cell line MDA-MB-231 as a potential mechanism of proapoptotic and antiproliferative properties of these compounds. Additionally, in order to acquire more information on their potential beneficial abilities, we examined interactions of compounds ***1***, ***2*** and ***3*** with calf thymus deoxyribonucleic acid (CT-DNA) and human serum albumin (HSA) by molecular docking and spectroscopic methods - including fluorescence and UV-visible absorption. Also, we modified previously published procedure for isolation of α-methylbutyrylshikon (***1***), acetylshikonin (***2***) and *β*-hydroxyisovalerylshikonin (***3***) from the roots of *O. visianii *(Vukic et al., 2017[47]).

## Materials and Methods

### Instrumentation

Semi-preparative high performance liquid chromatography was performed on Agilent 1100 Series liquid chromatograph (Agilent Technologies, Santa Clara, CA, USA) equipped with diode array detector (DAD; λ=520 nm, λ=450 nm), autosampler, and fraction collector; conditions: injection volume, 600 μL (2 mg/mL, methanol); column Zorbax Eclipse XDB C18 (250 mm x 9.4 mm; 5 μm); mobile phase (6 mL/min), water (40 %) and methanol (60 %). Sephadex-LH-20 purchased from GE Helthcare (Uppsala, Sweden) was used for column chromatography. Preparative TLC was performed by using silica gel P/UV254 with CaSO_4_ (Machery-Nagel, Germany, 2 mm layer of adsorbent). Analytical TLC was performed on silica gel (Silica gel 60, layer 0.20 mm, Alugram Sil G, Mashery-Nagel, Germany). All spectroscopic measurements were performed with a double beam UV-Vis spectrophotometer model Cary 300 (Agilent Technologies, Santa Clara, USA) with 1.0 cm quartz cells. Fluorescence measurements were carried out using a RF-1501 PC spectrofluorometer (Shimadzu, Japan) equipped with a 150 W Xenon lamp source with a 1.0 cm path length quartz cell. Color reaction was measured spectrophotometrically on ELISA (2100C) 96-well microplate reader (Rayto, China).

### Chemicals

Dulbecco's Modified Eagle Medium (DMEM), Fetal bovine serum (FBS), trypsin-EDTA and PBS were obtained from GIBCO, Invitrogen, USA. Nitro blue tetrazolium (NBT) and Nicotinamide Adenine Dinucleotide Phosphate (NADPH) were obtained from SERVA, Germany. Sodium nitrite, phosphoric acid, sulfanilamide, sulfanilic acid, sulfosalicylic acid, and 5,5′-Dithiobis(2-nitrobenzoic acid) (DTNB) were obtained from Sigma-Aldrich, USA. Petroleum ether (boiling point ranges: 40 °C-60 °C), methylene chloride, ethyl acetate, chloroform, acetic acid, dimethyl sulfoxide (DMSO), HPLC grade methanol, highly polymerized calf thymus DNA (CT-DNA), human serum albumin (HSA, lyophilized powder, free fatty acid r0.007 %, purity r96 %, Catalogue No. A1887), phosphate buffered saline (PBS) tablets, ethidium bromide, fetal bovine serum (FBS) and 7-Aminoactinomycin D (7-AAD) were purchased from Sigma-Aldrich, (Steinheim, Germany). Water was treated in a Milli-Q water purification system (TGI Pure Water Systems, Brea, CA, USA). 

### Plant material

Roots of *O. visianii* Clem were collected (Jun 2015) in the region of mountain Rumija (southern Montenegro, altitude 650 m, 42º 06' 10'' N, 19º 11' 37'' E). A voucher herbarium specimen was deposited at the Department of Botany, Faculty of Biology, University of Belgrade, Serbia (17130, BEOU).

### Modified procedure for isolation of selected naphthoquinones

Petroleum ether-methylene chloride extract of the *O. visianii* roots was prepared as previously described in our paper (Vukic et al., 2017[47]). Obtained extract was fractionated using preparative TLC eluted by mobile phases consisting of petroleum ether:chloroform:ethyl acetate:acetic acid (5:2:2.5:0.5) to give eight fractions (*F1-F8*). Preliminary TLC analysis was conducted, and retention times were compared to those of previously isolated compounds. Out of eight obtained fractions, presence of α-methylbutyrylshikon (***1***), acetylshikonin (***2***) and *β*-hydroxyisovalerylshikonin (***3***) was confirmed in three fractions (*F4*, *F6* and *F7*) which were further subjected to Sephadex LH20 column chromatography with methanol as eluent. After column chromatography, another TLC examination was performed using petroleum ether:ethyl acetate (90:10) to confirm isolated compounds. With the aim of obtaining high purity, isolated α-methylbutyrylshikon (***1***), acetylshikonin (***2***) and *β*-hydroxyisovalerylshikonin (***3***) were subjected to semi preparative HPLC on Zorbax Eclipse XDB C18 reversed phase column with isocratic elution of mixture water and methanol (40:60). Obtained spectra (UV, IR, ^1^H NMR and ^13^C NMR) were in agreement with previously published data (Vukic et al., 2017[47]).

### Estimation of redox status 

#### Stock solutions preparation

Tested naphthoquinones α-methylbutyrylshikon (1), acetylshikonin (2) and *β*-hydroxyisovalerylshikonin (3) were diluted in DMSO at the concentration of 1 mg/mL, filtered through a 0.22 µm Millipore filter before use. All treatment concentrations were obtained by serial dilutions of stock solution. Therefore, DMSO concentrations decreased continuously so that the final concentration of DMSO in cell culture medium never exceeded 0.5 % (v/v).

#### Cell preparation and culturing

The human colon cancer adenocarcinoma HCT-116 and human breast cancer MDA-MB-231 cell lines were obtained from the American Tissue Culture Collection. These cells were propagated and maintained in DMEM and supplemented with 10 % FBS and antibiotics (100 IU/mL of penicillin and 100 μg/mL of streptomycin). The cells were grown in 75 cm^2^ culture bottles and supplied with 15 mL of DMEM at a confluence of 70 % - 80 %. After a few passages, the cells were seeded in a 96-well plate and cultured in a humidified atmosphere with 5 % CO_2_ at 37 °C*. *Twenty-four hours later, the cells were treated with 100 μL of medium containing various doses of α-methylbutyrylshikon, acetylshikonin and *β*-hydroxyisovalerylshikonin with increasing concentrations (0.1 μg/mL to 100 μg/mL) during 24 h, 48 h and 72 h, after which the evaluation of levels of superoxide anion radical, nitrites and glutathione was performed. Non-treated cells were used as control. 

#### Determination of superoxide anion radical (NBT assay) 

Concentrations of superoxide anion radical (O_2_^.-^) were determined spectrophotometrically, using NBT assay based on the reduction of nitroblue tetrazolium (NBT) to nitroblue-formazan in the presence of O_2_^.- ^(Auclair and Voisin, 1985[1]). The assay was performed by adding 20 μL of 5 mg/mL NBT to each well and then the cells were incubated for 45 min at 37 °C in 5 % CO_2_. To quantify the formazan product, formazan was solubilized in 10 μL of 2M KOH, and the resulting color reaction was measured spectrophotometrically on microplate reader at 570 nm. The amount of reduced NBT was determined by the change in absorbance at 570 nm (based on molar extinction coefficient for monoformazan 15000 M^-1^ cm^-1^) and the results were expressed as nmoL/mL.

#### Determination of nitrites (Griess assay) 

Spectrophotometric determination of nitrites was performed using Griess method (Griess, 1879[18]). Nitrite standard solution (100 mM) was diluted from 100 μM to 1.6 μM (in triplicate) in a 96-well plate. Equal volumes of N-(1-naphthyl)ethylenediamine (1 mg/mL) and 5 % phosphoric acid solution of sulfanilamide (10 mg/mL) were mixed to form the Griess reagent. Prepared cells were incubated for 5 - 10 min, and after that the absorbance was measured at 550 nm by using microplate reader. The concentrations of nitrite were calculated from the standard curve for nitrite and expressed in nmol/mL.

#### Glutathione determination 

Samples for measurement of concentrations of reduced glutathione (GSH) and oxidized glutathione (GSSG) were obtained by the following procedure: the cell suspension was centrifuged for 10 min at 1000 × g and 4 °C and after the removal of supernatant, the pellet was resuspended in 2.25 % sulfosalicylic acid. Cell membranes were lysed by alternate freezing (- 80 °C) and thawing (37 °C) in three cycles for 15 min followed by 30 min of centrifugation at 1000 × g. The supernatant was used for further analysis. The assay is based on oxidation of a GSH by a DTNB reagent, with an active thiol group, which forms a yellow product of 5′-thio-2-nitrobenzoic acid (Baker et al., 1990[2]). Determination of concentration of GSSG was based on GSH determination assay using glutathione reductase after inhibition of spontaneous GSH oxidation by 4-vinylpyridine (Baker et al., 1990[2]). Glutathione concentration was expressed as μmol/mL.

Color reaction was measured spectrophotometrically on a microplate reader at 405 nm following 10 min incubation. Concentrations of reduced and oxidized glutathione were calculated from a standard curve constructed with determined concentrations. 

#### DNA binding study

Characterization of the binding modes and determination of strength of binding for selected naphthoquinone to CT-DNA were estimated by fluorescence and UV-Vis absorption study. The 2.0 x 10^-3^ M stock solutions of α-methylbutyrylshikon (***1***), acetylshikonin (***2***) and *β*-hydroxyisovalerylshikonin (***3***) were prepared by dissolving each naphthoquinone in small amounts of DMSO and diluting with phosphate buffer (pH 7.4) so the final DMSO concentration did not exceed 0.5 % v/v. The stock solutions of CT-DNA (2.0 x 10^-3^ M), EB (1.0 x 10^-3^ M) and Hoechst 33342 (1.0 x 10^-3^ M) were prepared by dissolving with phosphate buffer solution. Concentration of CT-DNA was determined spectrophotometrically at 260 nm using the molar absorptivity (ε_260 _= 6600 L/mol cm). DNA purity was checked by monitoring the ratio of the absorbance at 260 nm to that at 280 nm. The solution gave a ratio of 1.86 at A_260_/A_280_, which indicates that DNA was free from protein (Sirajuddin et al., 2013[43]; Djukić et al., 2018[12]). Prepared stock solutions were stored at 4 °C and used over no more than four days. All measurements were performed at room temperature. Appropriate blanks were used to correct the fluorescence or UV-Vis background.

The absorbance titration experiments were recorded at 235 - 800 nm using two similar methods. The first consisted of maintaining the constant concentrations of the *α*-methylbutyrylshikon (***1***), acetylshikonin (***2***) and *β*-hydroxyisovalerylshikonin (***3***) (8.0 x 10^-5 ^M), with the increasing concentration of CT-DNA (from 0 to 1.73 x 10^-4^ M), and the second was based on keeping the constant concentration of CT-DNA (1.66 x 10^-5 ^M) and varying the concentrations of the *α*-methylbutyrylshikon (***1***), acetylshikonin (***2***) and *β*-hydroxyisovalerylshikonin (***3***) (from 0 to 2.4 x 10^-5^ M). In addition, spectra containing only CT-DNA, *α*-methylbutyrylshikon (***1***), acetylshikonin (***2***) and *β*-hydroxyisovalerylshikonin (***3***) at the same concentration ratio used to evaluate interaction mode were recorded. 

The fluorescence quenching spectra of titration EB-DNA with selected naphthoquinones solutions were recorded in the range of 550 - 700 nm with an excitation wavelength at λ_ex _= 520 nm. Fluorescence spectra were recorded by keeping the constant concentrations of DNA (1.72 x 10^-5 ^M) and EB (1.2 x 10^-5 ^M), with the increasing concentration of α-methylbutyrylshikon (***1***), acetylshikonin (***2***) and *β*-hydroxyisovalerylshikonin (***3***) (from 0 to 2.4 x 10^-5 ^M). Also, the fluorescence quenching spectra of titration Heochst 33342-DNA with naphthoquinone solutions were recorded in the range of 380 - 600 nm with an excitation wavelength at λ_ex _= 350 nm. The fluorescence spectra of naphthoquinones were recorded under the same experimental conditions and no fluorescence emission was verified.

#### Protein binding study

Binding modes and determination of strength of binding for selected naphthoquinones to HSA were estimated by fluorescence, UV-Vis absorption and molecular docking study. The stock solutions of α-methylbutyrylshikon (***1***), acetylshikonin (***2***) and *β*-hydroxyisovalerylshikonin (***3***) were prepared in the same way as for the DNA binding study. The stock solution of HSA (2.0 x 10^-5^ M) was prepared by dissolving in phosphate buffer solution (pH 7.4). Prepared stock solutions were stored at 4 °C and used over no more than four days. All measurements were performed at room temperature. Proper blanks were used to correct the fluorescence or UV-Vis background. Absorbance titration experiments were recorded at 235-800 nm with the constant concentration of the HAS (2.0 x 10^-6 ^M), while the concentrations of α-methylbutyrylshikon (***1***), acetylshikonin (***2***) and *β*-hydroxyisovalerylshikonin (***3***) varied from 0 to 1.6 x 10^-5^ M. Additionally, the absorbances at 235 - 800 nm for solutions containing only selected naphthoquinones in the concentration ratio used for titration experiments were recorded. 

The emission spectra were recorded between 300 and 460 nm upon excitation at λ_ex _= 295 nm. The naphthoquinone-HSA complexes were prepared by incubating constant amount of HAS (2.0 x 10^-6 ^M) with increasing amounts of the α-methylbutyrylshikon (***1***), acetylshikonin (***2***) and *β*-hydroxyisovalerylshikonin (***3***) (from 0 to 1.6 x 10^-5 ^M). The fluorescence spectra of naphthoquinones were recorded under the same experimental conditions and no fluorescence emission was detected. 

#### Docking analysis

Inhibitory activity of investigated naphthoquinones against human serum albumin (HSA) and deoxyribonucleic acid (DNA) was tested using the AutoDock 4.2 software package (Morris et al., 2009[29]). Three-dimensional crystal structure of HSA (6EZQ) and DNA (dodecamer d(CGCAAATTTGCG)_2_ [PDB: 102D]) was obtained from the RCSB Protein Data Bank (Wenskowsky et al., 2018[51]; Vandevenne et al., 2013[46]). Preparation of HSA and DNA for docking simulations was done by removing the crystallized ligand, water molecules, and cofactors in the Discovery Studio 4.0 (BIOVIA Discovery Studio 2016) (BIOVIA, Dassault Systèmes, 2017[4]). The calculations of Kollman charges and adding of the polar hydrogen were performed using graphical user interface AutoDockTools (ADT). The optimization of the investigated molecules was performed by B3LYP-D3BJ/6-311+G(d,p) level of theory using the Gaussian09 software package (Frisch et al., 2013[14]). The HSA and DNA retained the rigid structure during molecular docking simulations in the ADT, while the investigated molecules were flexible. For calculations of partial charges, the Geistenger method was selected. Lamarckian Genetic Algorithm (LGA) was used in all calculations. A docking box with a grid consisting of 60 x 60 x 60 points with 0.375 Å spacing was placed into the active side of the receptor. All binding sites of the HSA and DNA were thus covered, which enabled free movement of examined compounds.

### Statistical analysis

The data are expressed as mean values ± standard errors (SE). All experiments were performed in triplicate for each dose. One-way analysis of variance (ANOVA) was performed to determine significant differences between means using SPSS for Windows, Version 17 (SPSS Inc., Chicago, IL, USA). 

## Results and Discussion

### The effects of selected naphthoquinones on redox status in HCT-116 and MDA-MB-231 cells

**Figure 1:**
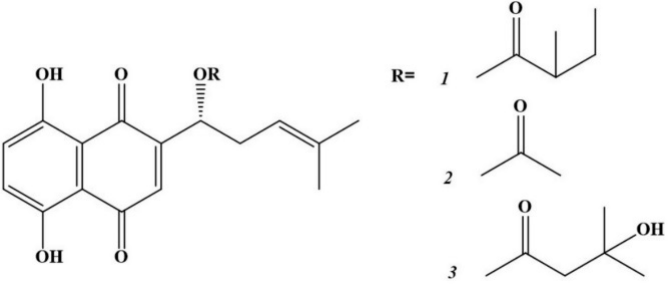
Chemical structures of α-methylbutyrylshikonin (*1*), acetylshikonin (*2*) and *β*-hydroxy-isovalerylshikon (*3*)

In our previous work we showed that α-methylbutyrylshikonin (***1***), acetylshikonin (***2***) and *β*-hydroxyisovalerylshikonin (***3***) induce apoptosis in HCT-116 and MDA-MB-231 cells. Hence, in this paper we investigated the effects of naphthoquinones ***1***, ***2*** and ***3*** (Figure 1(Fig. 1)) on oxidative stress markers as a potential mechanism of proapoptotic and antiproliferative properties (Vukic et al., 2017[47]).

**Figure 2:**
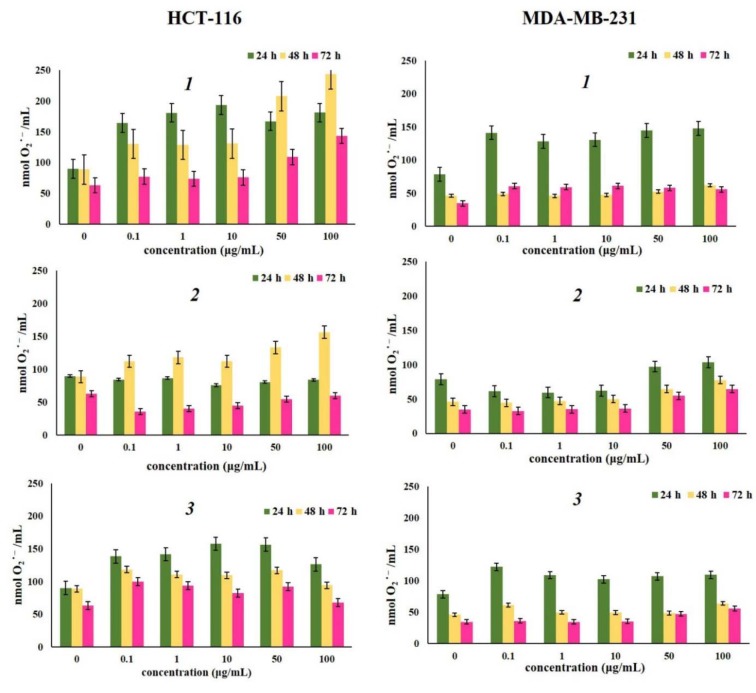
Effects of investigated naphthoquinones on HCT-116 and MDA-MB-231 cell lines, expressed as the nmol O_2_ ˙ ^-^/mL after 24 h, 48 h and 72 h of treatment. The cells were treated with α-methylbutyrylshikonin (*1*), acetylshikonin (*2*) and *β*-hydroxy-isovalerylshikonin (*3*) in concentration range from 0.1 to 100 μg/mL. Results were expressed as the means ± SE from three independent determinations.

Reactive oxygen species (ROS) play key roles in many cell processes essential for maintaining cell homeostasis, and superoxide anion radical is one of their key elements (Liu et al., 2017[26]). Accordingly, in this study we have evaluated effects of various concentrations of naphthoquinones ***1***, ***2*** and ***3 ***on superoxide anion radical production by HCT-116 and MDA-MB-231 cells after 24 h, 48 h, and 72 h treatment. The results presented in Figure 2(Fig. 2) showed that treatment with all compounds at different concentrations increased O_2_^·-^ level in HCT-116 and MDA-MB-231 cells compared to its production in non-treated cells. All investigated compounds showed prooxidative properties during all time treatments, and the strongest effects were detected after 24 h treatment. Overall, compound ***1 ***showed the strongest effect on superoxide anion radical production in HCT-116 and MDA-MB-231 cells compared to control after 24 h, 48 h and 72 h of treatment. Also, it can be observed that HCT-116 cells were more sensitive to treatment with the tested naphthoquinones compared to MDA-MB-231 cells. Acetylshikonin (***2***) increased production of O_2_^·- ^in HCT-116 cells only after 48 h of treatment, while after 24 h and 72 h of treatment the inhibition in production of superoxide anion radical was observed, possibly due to excessive production of nitric oxide which reacted with O_2_^·- ^, leading to formation of highly reactive peroxynitrite (ONOO^-^). However, in MDA-MB-231 cell line naphthoquinone ***2*** increased the production of superoxide anion after all three-time treatments. In general, it can be concluded that the tested naphthoquinones ***1*** and ***2*** show prooxidant activity in HCT-116 and MDA-MB-231 cell lines. Observed increased production of superoxide anion radical after the treatment may also be involved in expression of anti-proliferative effects of these compounds and their ability to induce apoptosis. From the structure point of view, quinone mechanisms of cytotoxicity are closely related to their ability to undergo one-electron reduction reaction catalyzed by enzymes such as NADPH-cytochrome P-450 reductase or mitochondrial NADH-ubiquinone oxidoreductase. This reaction forms semiquinone radical which can autoxidize in the presence of O_2_, generating quinone and superoxide anion radical (Papageorgiou et al., 1999[34]; Yang et al., 2014[54]; Murakami et al., 2010[31]). 

**Figure 3:**
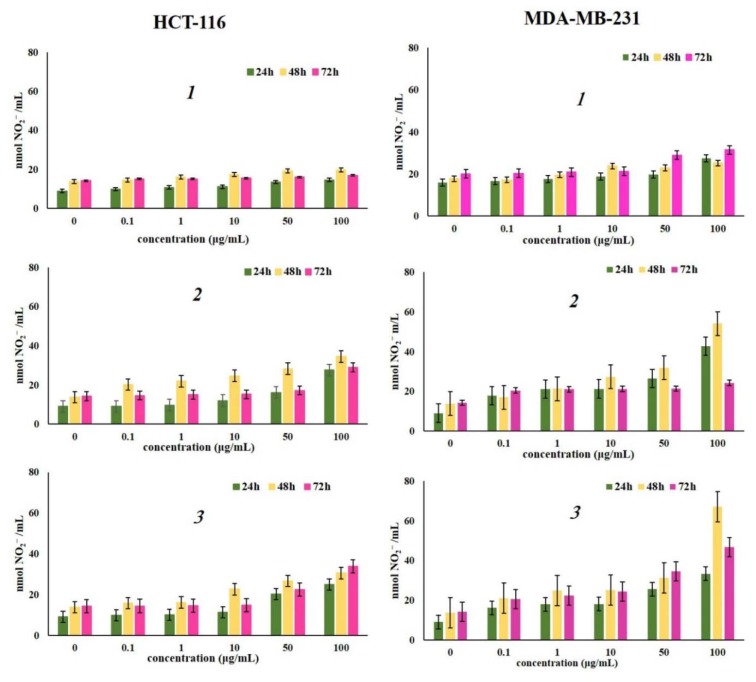
Effects of investigated naphthoquinones on HCT-116 and MDA-MB-231 cell lines, expressed as the nmol NO_2_^-^ /mL after 24 h, 48 h and 72 h of treatment. The cells were treated with α-methylbutyrylshikonin (*1*), acetylshikonin (*2*) and *β*-hydroxy-isovalerylshikonin (*3*) in concentration range from 0.1 to 100 μg/mL. Results were expressed as the means ± SE from three independent determinations.

Nitric oxide (NO) is an important signaling molecule in numerous physiological and pathological processes, with a controversial role in genesis and progression of various cancer types (Liu et al., 2003[27]). The data in Figure 3(Fig. 3) present nitrite concentrations in HCT-116 and MDA-MB-231 cells incubated with various concentrations of tested naphthoquinones after 24 h, 48 h and 72 h. Treatment with all compounds showed a significant increase in production of NO by HCT-116 and MDA-MB-231 cells measured indirectly through nitrite level compared to the level in non-treated cells. The strongest activity was shown by *β*-hydroxy-isovalerylshikonin (*3*) after all treatments, and MDA-MB-231 cells were more sensitive to treatment with the tested naphthoquinone derivatives. In general, for all investigated naphthoquinones, the highest production of NO for both cell lines was observed after 48 h of treatment. The lowest level in production of NO was observed for α-methylbutyrylshikonin (*1*) in both cell lines, which can be explained by generation of peroxynitrite due to elevated superoxide anion production. Jaw-Jou Kang and associates point out that superoxide anion reduces bioavailability of NO, which results in decreased activity of the NO-production pathway or increased oxidative inactivation of NO (Kang et al., 2006[22]).

**Figure 4:**
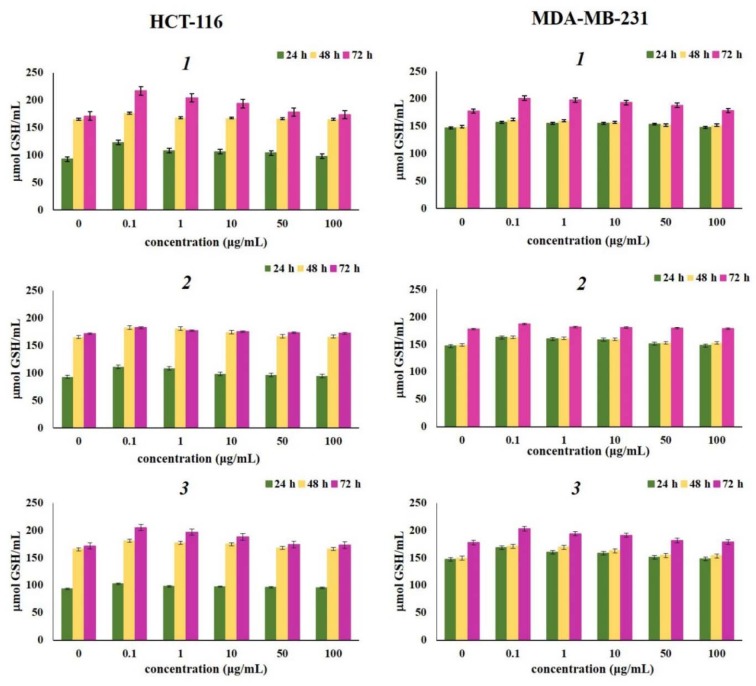
Effects of investigated naphthoquinones on the concentration of reduced glutathione (GSH) after 24 h, 48 h and 72 h of treatment. The HCT-116 and MDA-MB-231 cells were treated with α-methylbutyrylshikonin (*1*), acetylshikonin (*2*) and *β*-hydroxyisovalerylshikonin (*3*) in concentration range from 0.1 to 100 μg/mL. Results were expressed as the means ± SE from three independent determinations.

**Figure 5:**
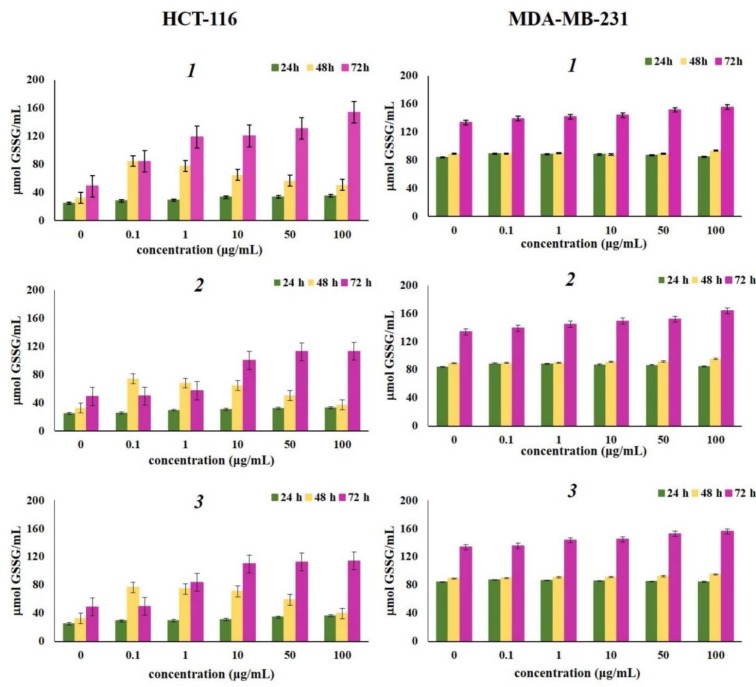
Effects of investigated naphthoquinones on the concentration of oxidized glutathione form (GSSG) after 24 h, 48 h and 72 h of treatment. The HCT-116 and MDA-MB-231 cells were treated with α-methylbutyrylshikonin (*1*), acetylshikonin (*2*) and *β*-hydroxyisovalerylshikonin (*3*) in concentration range from 0.1 to 100 μg/mL. Results were expressed as the means ± SE from three independent determinations.

Concentration of reduced and oxidized glutathione after 24 h, 48 h, and 72 h of incubation with various concentrations of naphthoquinones *1*, *2* and *3*, presented in Figure 4(Fig. 4) and Figure 5(Fig. 5) showed increase in the levels of both reduced and oxidized glutathione compared to control. Glutathione -GSH and its oxidized form -GSSG, represent important markers of the cell redox status (Traverso et al., 2013[45]). As one of the strongest antioxidative components present in cells, reduced glutathione (GSH) exerts various roles (e.g., maintains intracellular thiol status, involved in detoxication of different metabolites, regulates activity of some enzymes and cellular macromolecules) (Chung et al., 2016[8]). Also, being a potent electron donor, GSH is one of the major nonenzymatic antioxidant components (Jones, 2002[21]). Increased levels of oxidized glutathione were noted in cells treated with all tested naphthoquinones, after three experimental times (24 h, 48 h, and 72 h), suggesting a strong antioxidative activity provoked by considerable oxidative burst by the tested compounds. The strongest elevation of both glutathione forms correlates to the high concentration of superoxide anion radical. Elevated levels of reduced glutathione detected in this study may indicate that *1*, *2* and *3 *induced *de novo* synthesis of GSH (Shizhong et al., 2007[42]). GSH is synthesized in cells *de novo* and rapid induction of intracellular GSH synthesis occurs in response to various stressors (Shi et al., 1994[41]).

### DNA binding study

It is well known that ROS overproduction causes DNA damage through oxidation and DNA strand breaks. In addition, production of nitric oxide induces modifications of DNA by directly altering DNA through creating reactive nitrogen oxide species (RNOS) or indirectly by inhibiting various repair processes (Colin et al., 2014[9]; Graziewicz et al., 1996[17]). We have already pointed out that naphthoquinones α-methylbutyrylshikon (***1***), acetylshikonin (***2***) and *β*-hydroxyisovalerylshikonin (***3***) have prooxidant effect in cancer cells and induce production of NO_2_^-^. Hence, our next step was to investigate the possibility that observed result could be the consequence of interaction of ***1***, ***2*** and ***3 ***with calf thymus DNA (CT-DNA).

### UV-Vis measurements

Observing the spectral changes of DNA in the titration reaction with small molecules can provide evidence of binding mode of small molecule to CT-DNA. Effects of increasing amounts of CT-DNA on absorption spectra of ***1***, ***2*** and ***3*** are given in Supplementary Figure 1. It is known that CT-DNA shows absorption maximum at wavelength of 260 nm due to purine and pyrimidine bases (Sirajuddin et al., 2013[43]). Since there was no DNA absorption above mentioned maximum, absorption maximum of ***1***, ***2*** and ***3 ***at wavelength range of 460 - 600 nm followed. With increasing concentration of CT-DNA, minor hyperchromism with no obvious red or blue shift was observed. Presented results revealed that interaction between naphthoquinones and CT-DNA occurred. Intercalation binding mode of small molecules into the DNA helix results in hypochromism and red shift. Hence, these results indicate that naphthoquinones ***1***, ***2*** and ***3*** do not bind to DNA through intercalation binding mode (Qiao et al., 2008[35]). However, spectral changes observed in Supplementary Figure 1 for tested naphthoquinones are a feature that represents non-covalent interactions, particularly electrostatic and groove binding between small molecules and CT-DNA (Sarwar et al., 2015[37]).

**Figure 6:**
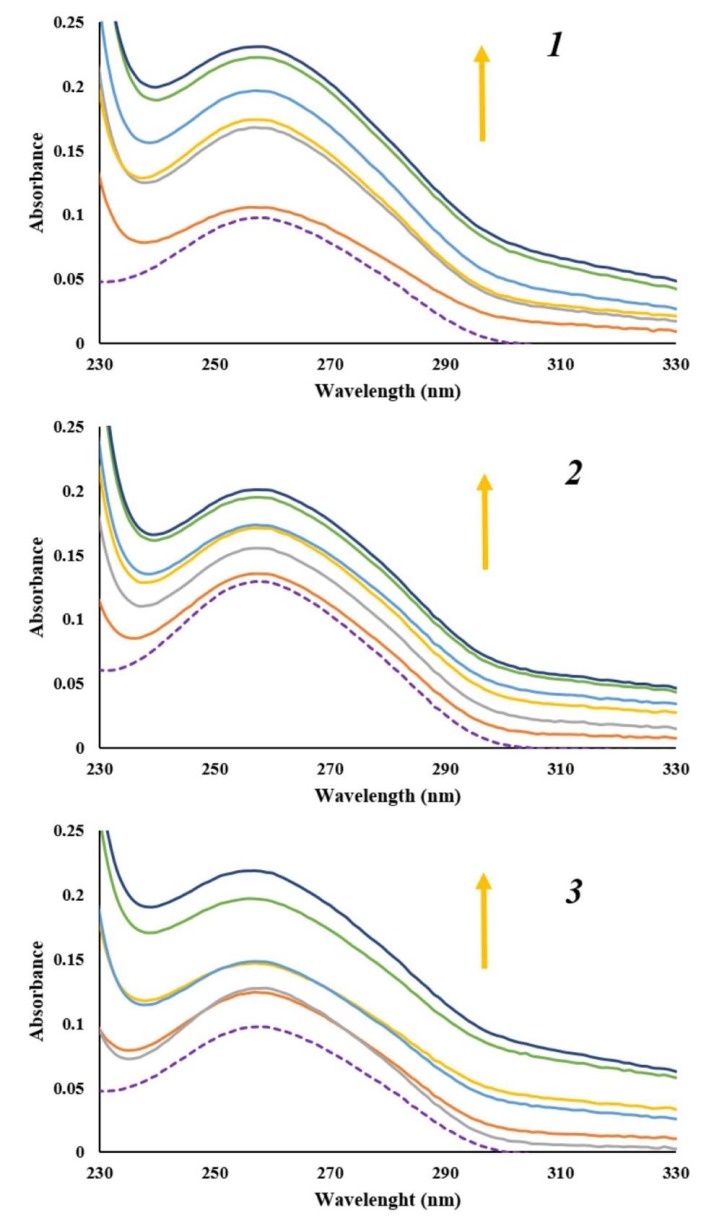
Absorption spectra of CT-DNA (1.77 x 10^-5 ^M) before (purple dashed line) and after addition of α-methylbutyrylshikon (*1*), acetylshikonin (*2*) and *β*-hydroxyisovalerylshikonin (*3*) (0 - 1.80 x 10^-5 ^M). Arrow shows the absorbance changes upon increasing concentration of α-methylbutyrylshikon (*1*), acetylshikonin (*2*) and *β*-hydroxyisovalerylshikonin (*3*).

In order to confirm interaction mode of naphthoquinones ***1***, ***2*** and ***3 ***to CT-DNA, absorption spectra of constant concentration of CT-DNA were recorded, in the absence and presence of increasing amounts of naphthoquinones. As shown in Figure 6(Fig. 6) with increasing concentration of ***1***, ***2*** and ***3*** concomitant hyperchromicity at the CT-DNA absorption maximum of 260 nm was observed. Observed hyperchromic effect is a result of groove binding interaction between tested naphthoquinones and CT-DNA. Also, absence of red or blue shift indicates that the structure of CT-DNA did not change much upon binding of naphthoquinones.

### Fluorescence measurements

In order to further investigate binding mode of naphthoquinones ***1***, ***2*** and ***3***, we employed fluorescence quenching study involving dye displacement. 

### Ethidium bromide displacement assay

**Figure 7:**
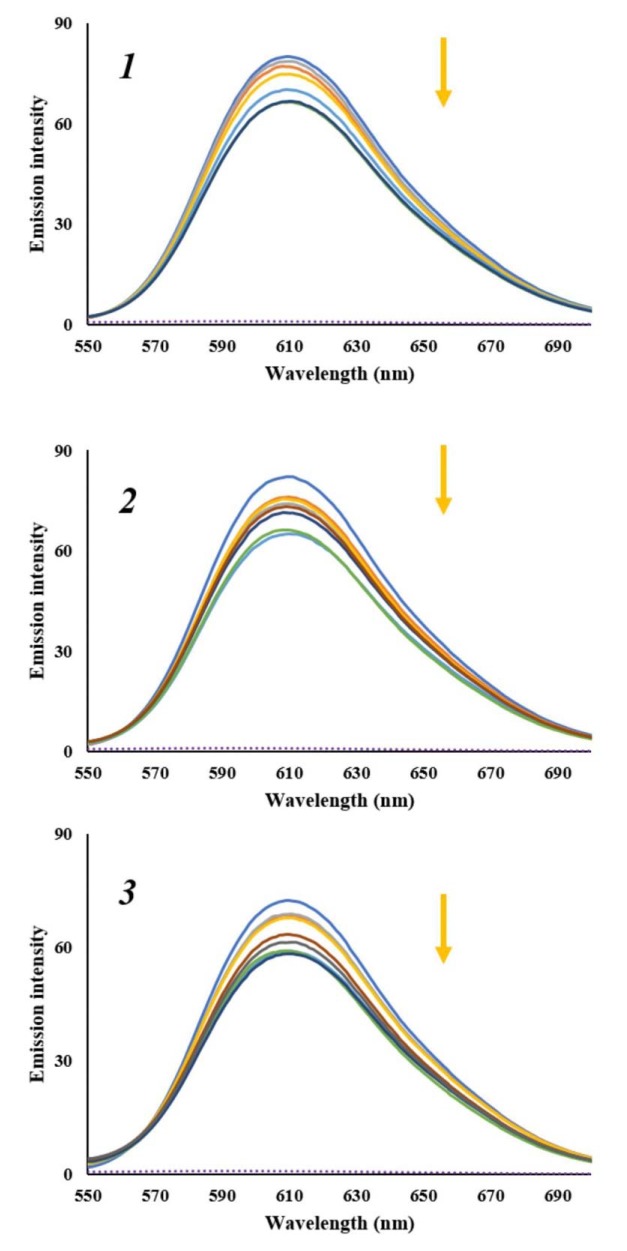
The fluorescence emission spectra of DNA-EB fixed concentration (DNA (1.72 x 10^-5 ^M) and EB (1.2 x 10^-5^ M)), in the absence and presence of increasing concentration of α-methylbutyrylshikon (*1*), acetylshikonin (*2*) and *β*-hydroxyisovalerylshikonin (*3*) (from 0 to 2.4 x 10^-5^ M). Arrow shows the intensity change upon the increase of the naphthoquinone concentration. Purple dashed line represents the emission spectra of *1*, *2* and *3* in the absence of DNA-EB.

Ethidium bromide (EB) shows weak fluorescence in the solvent, but when bonded to DNA, it gives a significant increase in fluorescence emission due to its strong intercalation between the adjacent DNA base pairs. Observed fluorescence emission can be quenched by the addition of a second molecule capable to replace EB or by electron transfer (Sirajuddin et al., 2013[43]; Cox et al., 2009[10]). The emission spectra of DNA-EB complex obtained in the absence and presence of increasing concentration of α-methylbutyrylshikon (***1***), acetylshikonin (***2***) and *β*-hydroxyisovalerylshikonin (***3***) were shown in Figure 7(Fig. 7). Presented results show no significant decrease in the maximum of emission intensity of EB-DNA complex at excitation wavelength of 520 nm with the increasing concentration of the naphthoquinones, which confirms that tested naphthoquinones do not intercalate into the DNA double helix. A slight decrease can be result of forming new non-fluorescence EB-DNA-naphthoquinone complex (Qiao et al., 2008[35]; Bi et al., 2006[3]).

### Hoechst 33342 displacement assay

**Figure 8:**
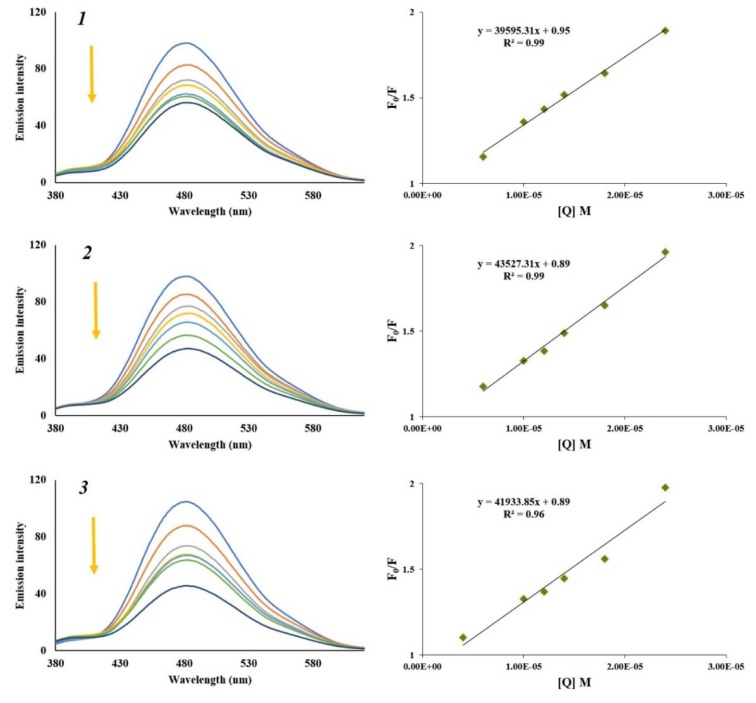
The fluorescence emission spectra of Hoechst-DNA fixed concentration (DNA (1.66 x 10^-5 ^M) and Hoechst (1.2 x 10^-5^ M)), in the absence and presence of increasing concentration of α-methylbutyrylshikon (*1*), acetylshikonin (*2*) and *β*-hydroxyisovalerylshikonin (*3*) (from 0 to 2.4 x 10^-5^ M). Arrow shows the intensity change upon the increase of the naphthoquinone concentration. Purple dashed line represents the emission spectra of *1*, *2* and *3* in the absence of DNA-Hoechst. Right: corresponding plots of F_0_/F versus [Q].

Unlike EB, Hoechst 33342 dye is a well-known minor groove DNA binder, and, like other minor groove binders, it recognizes A-T rich sequences in DNA double helix (Wu et al., 2007[52]; Shi et al., 2015[40]). Fluorescence emission of Hoechst 33342 bound to DNA is enhanced compared to free solution of Hoechst 33342. As well as for EB, addition of a small molecule can result in fluorescence quenching of Hoechst-DNA complex. In Figure 8(Fig. 8), emission spectra of Hoechst-DNA complex obtained in the absence and presence of increasing concentration of α-methylbutyrylshikon (***1***), acetylshikonin (***2***) and *β*-hydroxyisovalerylshikonin (***3***) are presented. Intensity of the emission band of Hoechst-DNA at 480 nm at excitation wavelength of 350 nm decreased with increasing concentration of the tested naphthoquinones. This dye displacement experiment, alongside with results obtained by UV-Vis absorption spectroscopy, suggests that naphthoquinones ***1***, ***2*** and ***3 ***are minor groove binders of DNA. 

The fluorescence quenching data of DNA-Hoechst were analyzed by the Stern-Volmer constant obtained by Eq (1):


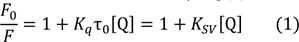


where F_0_ and F are the fluorescence intensities before and after addition of quencher, respectively. *K_q_* is the biomolecular quenching constant, τ_0_ is the life time of the fluorophore in the absence of quencher and and its value is considered to be 10^-8^ s, *K_SV_* is the Stern-Volmer quenching constant, and [Q] is the concentration of free quencher (Lakowicz, 2006[24]). The quenching constants (*K_SV_*) were obtained from slope and their values were 3.96 x 10^4^, 4.35 x 10^4^ and 4.19 x 10^4^ dm^3^ mol^-1^ for naphthoquinones ***1***, ***2*** and ***3***, respectively. Out of the calculated constants we can conclude that all tested naphthoquinones have similar ability to insert between DNA base pairs. 

### Protein binding study

Exploring protein - drug interactions is closely related to drug characteristics such as distribution, transportation and metabolism in the treatment of many diseases. Therefore, absorption and fluorescence quenching experiments were employed to explore the effect of binding of the naphthoquinones ***1***, ***2*** and ***3*** with the carrier protein HSA.

### UV-Vis measurements

**Figure 9:**
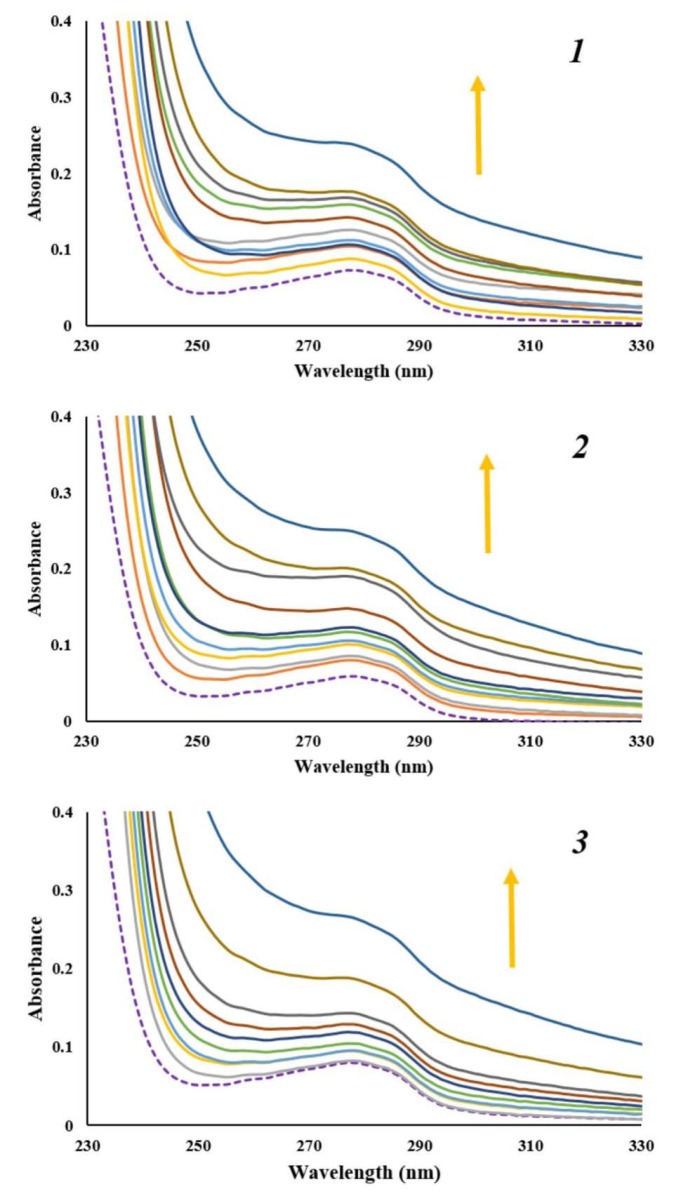
Absorption spectra of HSA (2.00 x 10^-6 ^M) before (purple dashed line) and after addition of α-methylbutyrylshikon (*1*), acetylshikonin (*2*) and *β*-hydroxyisovalerylshikonin (*3*) (0 - 1.60 x 10^-5 ^M). Arrow shows the absorbance changes upon increasing concentration of α-methylbutyrylshikon (*1*), acetylshikonin (*2*) and *β*-hydroxyisovalerylshikonin (*3*).

Titration experiment of HAS with increasing concentration of naphthoquinones ***1***, ***2*** and ***3*** (from 0 to 1.6 x 10^-5 ^M) monitored by UV-Vis spectroscopy and corresponding absorption spectra are reported in Figure 9(Fig. 9). Absorbance spectra of HAS resulted in a weak peak around 280 nm as a result of the cumulative π‐π* transition of Trp, Tyr, and Phe residues (Cao et al., 2019[7]; Yan et al., 2018[53]). Binding of naphthoquinones to HSA resulted in a continuous increase in absorbance maximum at 280 nm, indicating the changes in the local environment in HSA.

### Fluorescence measurements

**Figure 10:**
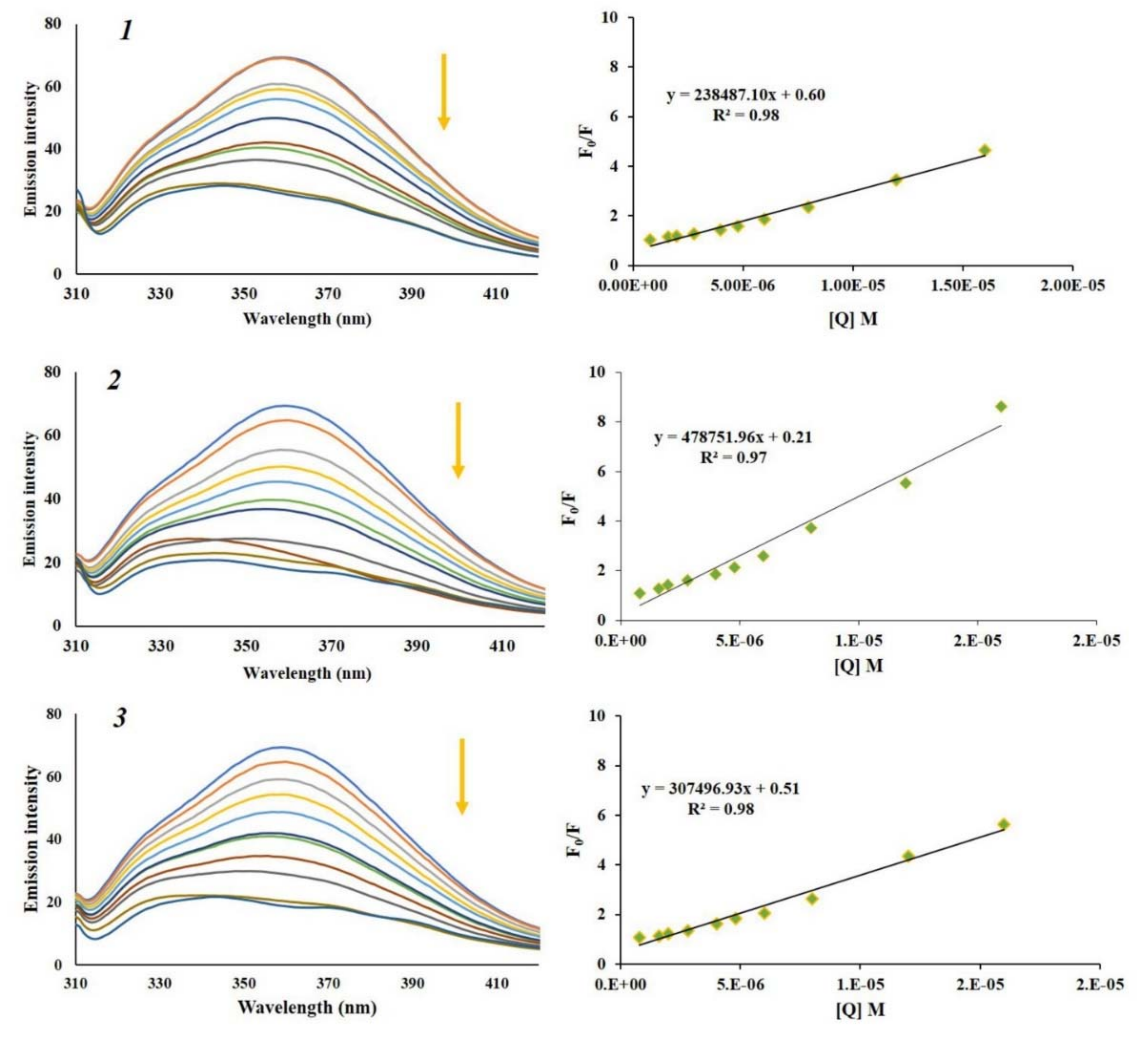
The fluorescence emission spectra of HSA fixed concentration (2.00 x 10^-6^ M), in the absence (blue line) and presence of increasing concentration of α-methylbutyrylshikon (*1*), acetylshikonin (*2*) and *β*-hydroxyisovalerylshikonin (*3*) (from 0 to 1.6 x 10^-5^ M) Insets: plots of F_0_/F versus [naphthoquinone]

In order to confirm results obtained by UV-Vis absorption spectroscopy, we monitored interactions of HSA with selected naphthoquinones by employing fluorescence spectroscopy. Decrease or shift in emission maximum of protein in the presence of increasing concentration of a small molecule could indicate new complex formation, random collisions, energy transfer and excited state reactions between them. Due to the Trp-214 residue, HSA fluorescence emission spectra at excitation wavelength of 295 nm showed a peak at around 360 nm. Hence, in the wavelength range 300 - 450 nm, fluorescence emission spectra of HSA with increasing concentration of naphthoquinones ***1***, ***2*** and ***3*** (from 0 to 1.6 x 10^-5 ^M) were recorded. From obtained spectra (Figure 10(Fig. 10)), decrease in the fluorescence intensity of the HAS was observed, followed by a slight shift to the shorter wavelength, with increasing concentration of naphthoquinones, indicating the changes in the local microenvironment around the Trp-214 residue in HSA by formation of non-fluorescent HSA-naphthoquinone complexes. Additionally, obtained results were in agreement with previously published data for naphthoquinone shikonin (He et al., 2005[19]). 

The fluorescence quenching data were analyzed by the Eq (1), similarly as described above for CT-DNA binding studies and obtained Stern-Volmer quenching constants *K_SV_* and the quenching rate constants *K_q_*, are given in Table 1(Tab. 1). From the slope of the regression line in the derived plot of F_0_/F versus [Q] (insets of Figure 10(Fig. 10)), the *Ksv* values for the complexes were determined to be in order 10^5^ dm^3^mol^-1^, indicating a strong affinity of tested naphthoquinones to HSA. As shown in Table 1(Tab. 1) bimolecular quenching constants were in the order of 10^13^ dm^3^moL^-1^s^-1^, indicating that formation of HSA-naphthoquinone complex is a static process (Makarska-Bialokoz and Lipke, 2019[28]; Panigrahi et al., 2015[33]).

### Binding constant and number of binding sites

The number of binding sites (*n*) and the binding constant (*K_b_*) presented in Table 1(Tab. 1) were obtained from the intercept and slope of the plots of log(F_0_ - F)/F versus log[Q] derived from the Eq (2):


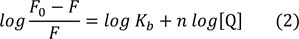


where F_0_ and F are the fluorescence intensities in the absence and presence of the quencher, respectively, *Kb *is binding constant or the apparent association constant for small molecule-protein interaction, *n* is the number of binding sites per protein and [Q] is the concentration of quencher (Lakowicz, 2006[24]). From the number of binding sites calculated to be approximately 1, formation of a ground state complex with a single binding site in HSA for all tested naphthoquinones was confirmed. The values of the binding constants (*K_b_*) showed that acetylshikonin bound more strongly than other investigated naphthoquinones. 

### Molecular docking analysis of naphthoquinones with DNA and HSA

**Figure 11:**
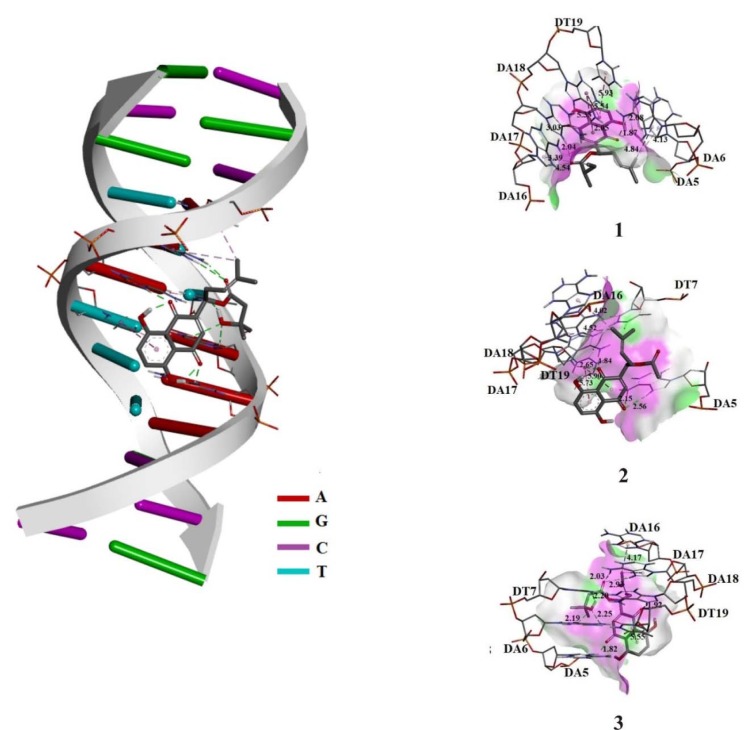
Interactions between α-methylbutyrylshikon (*1*), acetylshikonin (*2*) and *β*-hydroxyisovalerylshikonin (*3*) and DNA. The conventional hydrogen bonds are labeled using green dashed lines and hydrophobic interactions are labeled with purple dashed lines.

**Figure 12:**
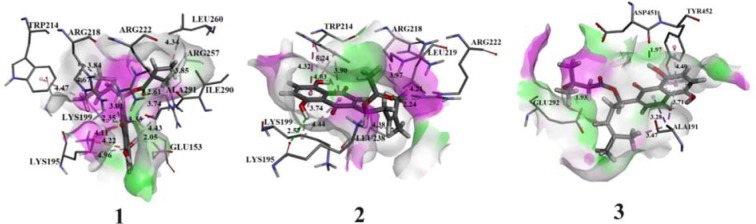
Interactions between α-methylbutyrylshikon (*1*), acetylshikonin (*2*) and *β*-hydroxyisovalerylshikonin (*3*) and amino acids of HSA. The conventional hydrogen bonds are labeled using green dashed lines.

Molecular docking studies were performed for the evaluation of the inhibitory nature of investigated naphthoquinones ***1***, ***2***, and ***3*** against DNA and HSA. In this study, the binding energy of complexes of the DNA-naphthoquinones and of the HSA-naphthoquinones was determined, as well as the prediction of potential investigated compounds-binding sites. Based on the docking results, the most stable conformers with the lowest free energy of binding were obtained. Obtained values of the pairwise interaction energies (E_i_), constant of inhibition (K_i_), free energy of binding (ΔG_bind_) as well as the non-covalent interactions for the investigated models are presented in Supplementary Tables 1 and 2. The most stable conformation complexes of naphthoquinones (***1***, ***2***, and ***3***) with DNA and HSA were presented in Figures 11(Fig. 11) and 12(Fig. 12) respectively.

The obtained results presented in Figure 11(Fig. 11) indicate that naphthoquinones bind in AT-rich region of DNA via conventional hydrogen-bonding and π-alkyl as significant non-covalent interactions (Supplementary Table 1). Obtained values of the pairwise interaction energies (E_i_), constant of inhibition (K_i_), free energy of binding (ΔG_bind_) as well as the non-covalent interactions for the investigated models are presented in Supplementary Table 1. The resulting relative binding energies of ***1***, ***2***, and ***3*** with DNA were -22.99, -20.98, and -20.31 kJ mol^-1^, respectively, which is in accordance with results obtained by employing UV-Vis absorption and fluorescence spectroscopy study and indicates similar DNA-binding affinity of tested naphthoquinones. 

The investigated naphthoquinone derivatives contain several polar groups: one ester, two carbonyl and two or three hydroxyl groups. A large number of polar groups in the investigated compounds increases the number of possible interactions with the amino acids of the HSA protein. The obtained results are shown in Supplementary Table 2 and indicate that the conventional hydrogen bond is the most important type of interaction. In addition, significant non-covalent interactions in naphthoquinones-HSA complexes are alkyl-π, σ-π, π-π, π-cation as well as π-π stacked (in complexes ***2*** and ***3***). All atomic distances of the conventional hydrogen bonds were in the range of 1.5 - 3.0 Å, while other interactions showed longer distances (Supplementary Table 2). These interactions were defined with high pairwise interaction energy and large atomic distances (≥3 Å). The relative free energy of binding for compounds ***1***, ***2***, and ***3*** with HSA were -13.17, -29.08, and -7.74 kJ mol^-1^, respectively. These results confirm the experimental ones, that naphthoquinone ***2*** forms the most stable complex with corresponding protein (Supplementary Table 2).

For more results see the Supplementary data.

## Conclusion

In this study we reported that naturally occurring naphthoquinones α-methylbutyrylshikon (***1***), acetylshikonin (***2***) and *β*-hydroxyisovalerylshikonin (***3***) isolated from *O. visianii* induced disturbance in oxidative homeostasis of colon and breast cancer cells and increased the levels of superoxide anion radical and nitrites. All examined compounds significantly increased the concentration of oxidized glutathione (GSSG), suggesting an activation of cell antioxidant mechanisms. Also, all examined compounds significantly increased the concentration of reduced glutathione (GSH), indicating *de novo* synthesis and further confirming excessive prooxidative conditions. Naphthoquinone ***1*** exerted the strongest prooxidative effects in both cell lines, while compound ***2*** showed the highest increase in nitrite production compared to non-tretaed cells. The levels of oxidized glutathione were highest in the treatment with compound ***1***, which corresponded to the highest concentration of superoxide anion radical among these three derivatives. The results obtained in this experiment indicate a significant prooxidative role of the examined compounds in HCT-116 and MDA-MB-231 cells, which could be one of the potential mechanisms of growth inhibition and apoptosis induction in these cell types. Likewise, as CT-DNA is a possible biomolecule target for antitumor activity, naphthoquinones-CT-DNA interactions study revealed favored binding of the all three compounds at the A-T region in minor groove. As minor groove binders are known to be an important class of derivatives in anticancer therapy, naturally occurring naphthoquinones ***1***, ***2*** and ***3 ***may be further exploited to understand their potential as antiproliferative drugs. In addition, the intersection study with HSA showed that the tested compounds could be transported and distributed through the cells. According to the data presented in this paper, tested naphthoquinones could have a significant beneficial role in developing new strategies against colon and breast cancer. 

## Conflict of interest

The authors confirm that this article content has no conflict of interest.

## Acknowledgements

This work was financially supported by the Ministry of Education, Science and Technological Development of the Republic of Serbia (Grant OI172016 and Grant III41010).

## Supplementary Material

Supplementary material

Supplementary data

## Figures and Tables

**Table 1:**
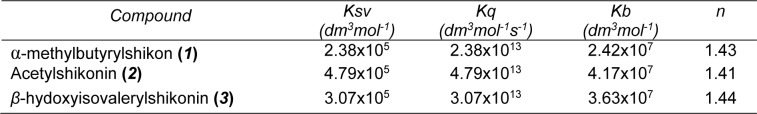
Quenching constant (*K_SV_*), the quenching rate constants (*K_q_*), binding constant (*K_b_*), and number of binding sites (n) for naphthoquinone-HAS interactions
