# Supplementary material for: Redox status, DNA and HSA binding study of naturally occurring naphthoquinone derivatives
Source: EXCLI J. 2020 Jan 3;19:48–70. doi: 10.17179/excli2019-1859 (PMC7003638; doi:10.17179/excli2019-1859)
Supplement: Supplementary material [file EXCLI-19-48-s-001.pdf]

EXCLI Journal 2020;19:48-70 – ISSN 1611-2156
Received: October 03, 2019, accepted: December 19, 2019, published: January 03, 2020

Supplementary material to:

REDOX STATUS, DNA AND HSA BINDING STUDY OF NATURALLY OCCURRING NAPHTHOQUINONE DERIVATIVES

Milena D. Vukic[1], Nenad L. Vukovic[1,*], Ana Obradovic[2], Milos Matic[2], Maja Djukic[1], Edina Avdovic[1,3]

[1] Department of Chemistry, Faculty of Science, University of Kragujevac, Radoja Domanovića 12, 34000 Kragujevac, Serbia
[2] Department of Biology and Ecology, Faculty of Science, University of Kragujevac, Radoja Domanovića 12, 34000 Kragujevac, Serbia
[3] Department of Sciences, Institute for Information Technologies Kragujevac, University of Kragujevac, Jovana Cvijića bb, 34000 Kragujevac, Serbia

[*] **Corresponding author:** Nenad L. Vukovic, Department of Chemistry, Faculty of Science, University of Kragujevac, P.O. Box 60, 34000 Kragujevac, Serbia. Tel: +38134336223; Fax: +38134335040; E-mail: nvukovic@kg.ac.rs

http://dx.doi.org/10.17179/excli2019-1859

This is an Open Access article distributed under the terms of the Creative Commons Attribution License (http://creativecommons.org/licenses/by/4.0/).

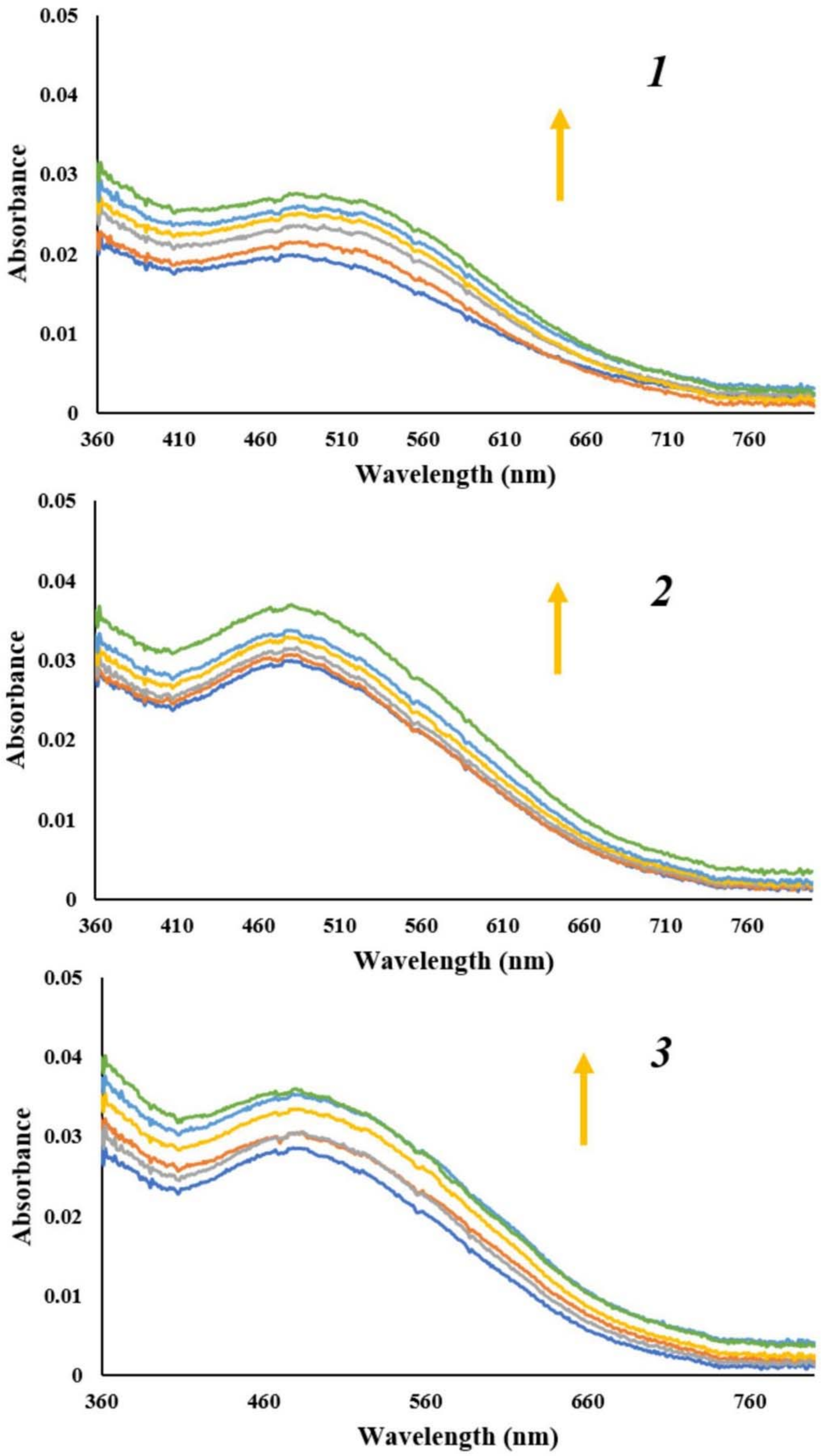

Supplementary Figure 1: Absorption spectra of α-methylbutyrylshikon (***1***), acetylshikonin (***2***) and *β*-hydroxyisovalerylshikonin (***3***) (concentration of each was 8.00 x 10^{-5} M) before (blue line) and after addition of CT-DNA (0.00-1.73 x 10^{-4} M). Arrow shows the absorbance changes upon increasing concentration of CT-DNA.

EXCLI Journal 2020;19:48-70 – ISSN 1611-2156
Received: October 03, 2019, accepted: December 19, 2019, published: January 03, 2020

Supplementary Table 1: Results of docking analysis of the α-methylbutyrylshikonin (***1***), acetylshikonin (***2***) and *β*-hydroxyisovalerylshikonin (***3***) with DNA: corresponding purine bases with its position in the structure of DNA, type of interaction during bond formation, distance in Å between respective active cites of naphthoquinones and purine bases, pairwise interaction energy (Ei) in kJ mol^{-1}, (**ΔG_{bind}**) in kJ mol^{-1}**,** binding energy, estimated inhibition constant (Ki) in µM.

Complexes	Interaction	Type of interaction	Distance (Å)	**E_i** (kJ mol^{-1})	**ΔG_{bind}** (kJ mol^{-1})	**K_i** (µM)
**1-DNA**	DA5:H-LIG:O	Conventional Hydrogen Bond	2.08	-0.60	**-22.99**	**93.14**
	DA6:H-LIG:O	Conventional Hydrogen Bond	1.87	-0.60		
	DA17:H-LIG:O	Conventional Hydrogen Bond	3.03	-0.05		
	DA16:C -LIG:O	Carbon Hydrogen Bond	3.39	-0.03		
	DA17:C-LIG:O	Carbon Hydrogen Bond	2.04	-0.03		
	DA18:H-LIG	Pi-Donor Hydrogen Bond	2.05	-0.09		
	DA18 -LIG	Pi-Pi T-shaped	5.93	-0.01		
	DA18 -LIG	Pi-Pi T-shaped	5.35	-0.01		
	DT19 -LIG	Pi-Pi T-shaped	5.54	-0.01		
	DA5-LIG	Pi-Alkyl	4.13	-0.02		
	DA6-LIG	Pi-Alkyl	4.84	-0.02		
	DA16-LIG	Pi-Alkyl	4.54	-0.02		
**2-DNA**	DA5:H-LIG:O	Conventional Hydrogen Bond	2.56	-0.20	**-20.98**	**209.04**
	DA5:H-LIG:O	Conventional Hydrogen Bond	2.15	-0.60		
	DA18:H-LIG:O	Conventional Hydrogen Bond	2.65	-0.20		
	DA18:H-LIG:O	Conventional Hydrogen Bond	1.84	-0.60		
	DT19 - LIG	Pi-Pi T-shaped	5.73	-0.01		
	DT19 - LIG	Pi-Pi T-shaped	5.90	-0.01		
	DA16 - LIG	Pi-Alkyl	4.02	-0.02		
	DA17 - LIG	Pi-Alkyl	4.52	-0.02		
**3-DNA**	DA5:H-LIG:O	Conventional Hydrogen Bond	1.82	-0.60	**-20.31**	**274.69**
	DA6:H-LIG:O	Conventional Hydrogen Bond	2.19	-0.60		
	DA6:H-LIG:O	Conventional Hydrogen Bond	2.25	-0.60		
	DA17:H-LIG:O	Conventional Hydrogen Bond	2.93	-0.07		
	DA17:H-LIG:O	Conventional Hydrogen Bond	2.03	-0.60		
	DA18:H- LIG:O	Conventional Hydrogen Bond	1.92	-0.60		
	LIG:H - DT7:O4	Conventional Hydrogen Bond	2.20	-0.60		
	DT19 - LIG	Pi-Pi T-shaped	5.55	-0.01		
	DA16 - LIG	Pi-Alkyl	4.17	-0.02		

EXCLI Journal 2020;19:48-70 – ISSN 1611-2156
Received: October 03, 2019, accepted: December 19, 2019, published: January 03, 2020

Supplementary Table 2: Results of docking analysis of the α-methylbutyrylshikonin (***1***), acetylshikonin (***2***) and *β*-hydroxyisovalerylshikonin (***3***) with HSA: corresponding amino acid with its position in the structure of protein, type of interaction during bond formation, distance in Å between respective active cites of naphthoquinones and amino acids, pairwise interaction energy (Ei) in kJ mol^{-1}, (ΔG_{bind}) in kJ mol^{-1} binding energy, estimated inhibition constant (Ki) in µM.

Complexes	Interaction	Type of interaction	Distance (Å)	**E$_i$** (kJ mol^{-1})	Δ**G**$_{bind}$ (kJ mol^{-1})	**K$_i$** (µM)
**1-HSA**	LYS199:H- LIG:O	Conventional Hydrogen Bond	2.35	-2.00	**-13.17**	**4.91**
	ARG222:H - LIG:O	Conventional Hydrogen Bond	3.01	-0.21		
	ARG257:H - LIG:O	Conventional Hydrogen Bond	2.61	-0.08		
	LIG:H - GLU153:O	Conventional Hydrogen Bond	2.05	-2.51		
	LYS195:N - LIG	Pi-Cation	4.22	-0.08		
	LYS195:N- LIG	Pi-Cation	4.11	-0.08		
	ALA291:C - LIG	Pi-Sigma	3.33	-0.17		
	ALA291 - LIG:C	Alkyl	3.74	-0.08		
	LIG:C - ARG257	Alkyl	3.85	-0.08		
	LIG:C - LEU260	Alkyl	4.34	-0.08		
	LIG:C - ARG218	Alkyl	3.84	-0.08		
	LIG:C - ARG218	Alkyl	4.67	-0.08		
	TRP214 - LIG1:C	Pi-Alkyl	4.47	-0.08		
	LIG - LYS195	Pi-Alkyl	4.96	-0.08		
	LIG - ALA291	Pi-Alkyl	4.43	-0.08		
**2-HSA**	ARG222:H - LIG:O	Conventional Hydrogen Bond	2.24	-2.14	**-29.08**	**8.08**
	LIG:H - LYS195:O	Conventional Hydrogen Bond	2.57	-1.13		
	TRP214 - LIG	Pi-Pi Stacked	4.32	-0.08		
	TRP214 - LIG	Pi-Pi Stacked	5.24	-0.04		
	TRP214 - LIG	Pi-Pi Stacked	4.03	-0.08		
	TRP214 - LIG	Pi-Pi Stacked	3.90	-0.08		
	LIG:C - ARG218	Alkyl	3.97	-0.08		
	LIG:C - LEU238	Alkyl	4.38	-0.08		
	LIG:C - LEU219	Alkyl	4.21	-0.08		
	LIG - LYS199	Pi-Alkyl	3.74	-0.08		
	LIG - LYS199	Pi-Alkyl	4.44	-0.08		
**3-HSA**	LIG:H - GLU292:O	Conventional Hydrogen Bond	1.93	-2.51	**-7.74**	**44.21**
	LIG:H - ASP451:O	Conventional Hydrogen Bond	1.97	-2.51		
	ALA191:C - LIG	Pi-Sigma	3.71	-0.08		
	ALA191:C - LIG	Pi-Sigma	3.28	-0.08		
	TYR452 - LIG	Pi-Pi T-shaped	4.40	-0.08		
	ALA191 - LIG:C	Alkyl	3.47	-0.08		
