# Supplementary material for: Redox status, DNA and HSA binding study of naturally occurring naphthoquinone derivatives
Source: EXCLI J. 2020 Jan 3;19:48–70. doi: 10.17179/excli2019-1859 (PMC7003638; doi:10.17179/excli2019-1859)
Supplement: Supplementary data [file EXCLI-19-48-s-002.pdf]

EXCLI Journal 2020;19:48-70 – ISSN 1611-2156
Received: October 03, 2019, accepted: December 19, 2019, published: January 03, 2020

Supplementary data to:

REDOX STATUS, DNA AND HSA BINDING STUDY OF NATURALLY OCCURRING NAPHTHOQUINONE DERIVATIVES

Milena D. Vukic[1], Nenad L. Vukovic[1,*], Ana Obradovic[2], Milos Matic[2], Maja Djukic[1], Edina Avdovic[1,3]

[1] Department of Chemistry, Faculty of Science, University of Kragujevac, Radoja Domanovića 12, 34000 Kragujevac, Serbia
[2] Department of Biology and Ecology, Faculty of Science, University of Kragujevac, Radoja Domanovića 12, 34000 Kragujevac, Serbia
[3] Department of Sciences, Institute for Information Technologies Kragujevac, University of Kragujevac, Jovana Cvijića bb, 34000 Kragujevac, Serbia

[*] **Corresponding author:** Nenad L. Vukovic, Department of Chemistry, Faculty of Science, University of Kragujevac, P.O. Box 60, 34000 Kragujevac, Serbia. Tel: +38134336223; Fax: +38134335040; E-mail: nvukovic@kg.ac.rs

http://dx.doi.org/10.17179/excli2019-1859

This is an Open Access article distributed under the terms of the Creative Commons Attribution License (http://creativecommons.org/licenses/by/4.0/).

EXCLI Journal 2020;19:48-70 – ISSN 1611-2156
Received: October 03, 2019, accepted: December 19, 2019, published: January 03, 2020

Raw tables concerning **Figure 2.** Effects of investigated naphthoquinones on HCT-116 and MDA-MB-231 cell lines, expressed as the nmol $O_2^{\cdot -}$/mL after 24 h, 48 h and 72 h of treatment. The cells were treated with α-methylbutyrylshikonin (***1***), acetylshikonin (***2***) and *β*-hydroxyisovalerylshikonin (***3***) in concentration range from 0.1 to 100 μg/mL. Results were expressed as the means ± SE from three independent determinations.

Table 1: Observed absorbances of effects of α-methylbutyrylshikonin (***1***), acetylshikonin (***2***) and β-hydroxy-isovalerylshikonin (***3***) (in concentration range from 0.1 to 100 μg/mL) on superoxide anion radical production by HCT-116 cell line after 24 h, 48 h and 72 h of treatment.

Time of treatment	Concentration (μg/mL)															
	control	α-methylbutyrylshikonin					acetylshikonin					β-hydroxy-isovalerylshikonin				
	0	*0.1*	*1*	*10*	*50*	*100*	*0.1*	*1*	*10*	*50*	*100*	*0.1*	*1*	*10*	*50*	*100*
**24h**																
	1.136	1.669	1.498	1.598	1.164	1.102	1.022	0.985	0.762	0.426	0.701	1.120	1.102	1.011	1.012	0.817
	1.130	1.651	1.490	1.602	1.140	1.093	1.023	0.981	0.765	0.435	0.698	1.127	1.105	1.005	1.010	0.822
	1.142	1.678	1.495	1.595	1.165	1.089	1.031	0.990	0.756	0.439	0.695	1.113	1.093	1.017	1.015	0.812
	1.117	1.481	1.811	1.589	1.227	1.172	0.969	0.932	0.574	0.682	0.589	1.117	1.135	1.095	1.067	0.870
	1.135	1.500	1.820	1.602	1.219	1.195	0.985	0.939	0.590	0.693	0.598	1.130	1.165	1.092	1.058	0.863
	1.083	1.471	1.813	1.569	1.235	1.149	0.960	0.920	0.559	0.669	0.587	1.103	1.148	1.089	1.065	0.864
	1.121	1.574	1.175	1.569	1.126	1.121	0.909	0.878	0.950	0.932	0.485	1.115	1.045	1.182	1.123	0.771
	1.130	1.580	1.169	1.578	1.132	1.148	0.920	0.869	0.957	0.925	0.479	1.123	1.056	1.169	1.112	0.775
	1.120	1.571	1.172	1.579	1.129	1.134	0.923	0.875	0.935	0.929	0.482	1.119	1.050	1.165	1.105	0.762
**48h**																
	1.022	0.767	0.639	0.994	1.435	1.192	1.059	1.107	1.091	0.983	0.941	1.405	0.724	0.479	1.103	0.614
	1.060	0.776	0.631	0.981	1.486	1.178	1.042	1.099	1.096	0.981	0.943	1.403	0.713	0.498	1.203	0.624
	1.073	0.758	0.677	0.965	1.509	1.189	1.039	1.118	1.079	0.970	0.937	1.431	0.755	0.486	1.091	0.641
	1.082	1.103	1.268	0.789	1.422	1.125	1.112	1.078	0.897	0.891	0.982	0.725	0.829	0.987	0.697	0.813
	1.087	1.105	1.256	0.808	1.619	1.182	1.066	1.118	0.925	0.921	0.997	0.809	0.776	0.889	0.685	0.793
	1.079	1.115	1.262	0.799	1.487	1.122	1.098	1.059	0.868	0.903	0.968	0.769	0.806	0.778	0.699	0.822
	1.192	1.125	0.949	0.995	1.448	1.229	1.131	1.062	0.993	1.068	0.890	0.744	1.188	1.274	0.739	0.567
	1.199	1.118	0.961	1.047	1.459	1.219	1.140	1.055	1.089	1.089	0.907	0.750	1.192	1.282	0.742	0.590
	1.185	1.148	0.958	1.039	1.429	1.239	1.137	1.068	0.897	0.992	0.897	0.738	1.200	1.289	0.736	0.552
**72h**																
	0.690	0.770	0.705	0.700	0.722	0.706	0.359	0.298	0.256	0.282	0.307	0.825	0.636	0.814	0.652	0.201
	0.723	0.790	0.709	0.742	0.785	0.699	0.412	0.265	0.245	0.301	0.300	0.818	0.697	0.717	0.621	0.204
	0.716	0.788	0.712	0.734	0.703	0.705	0.379	0.312	0.311	0.263	0.293	0.839	0.671	0.682	0.633	0.208

EXCLI Journal 2020;19:48-70 – ISSN 1611-2156
Received: October 03, 2019, accepted: December 19, 2019, published: January 03, 2020

Time of treatment	Concentration (μg/mL)															
	control	α-methylbutyrylshikonin					acetylshikonin					β-hydroxy-isovalerylshikonin				
	0	*0.1*	*1*	*10*	*50*	*100*	*0.1*	*1*	*10*	*50*	*100*	*0.1*	*1*	*10*	*50*	*100*
	0.890	0.787	0.703	0.786	0.722	0.651	0.436	0.410	0.286	0.302	0.285	0.772	0.865	0.712	0.625	0.329
	0.894	0.783	0.754	0.762	0.725	0.676	0.413	0.406	0.299	0.305	0.275	0.761	0.877	0.710	0.621	0.358
	0.898	0.780	0.722	0.738	0.713	0.687	0.438	0.403	0.311	0.297	0.283	0.780	0.888	0.708	0.618	0.388
	0.760	0.783	0.733	0.691	0.703	0.642	0.475	0.519	0.281	0.268	0.253	0.708	0.614	0.468	0.338	0.281
	0.753	0.785	0.751	0.697	0.705	0.658	0.481	0.523	0.289	0.258	0.263	0.720	0.613	0.470	0.320	0.274
	0.771	0.780	0.742	0.685	0.701	0.649	0.468	0.515	0.286	0.277	0.268	0.718	0.615	0.465	0.342	0.289

Table 2: Observed absorbances of effects of α-methylbutyrylshikonin (***1***), acetylshikonin (***2***) and β-hydroxy-isovalerylshikonin (***3***) (in concentration range from 0.1 to 100 μg/mL) on superoxide anion radical production by MDA-MB-231 cell line after 24 h, 48 h and 72 h of treatment.

Time of treatment	Concentration (μg/mL)															
	control	α-methylbutyrylshikonin					acetylshikonin					β-hydroxy-isovalerylshikonin				
	0	*0.1*	*1*	*10*	*50*	*100*	*0.1*	*1*	*10*	*50*	*100*	*0.1*	*1*	*10*	*50*	*100*
**24h**																
	0.974	1.403	1.015	1.020	1.204	0.951	0.675	0.565	0.592	0.620	0.510	0.895	0.771	0.729	0.746	0.747
	0.986	1.378	1.001	1.006	1.189	1.005	0.690	0.693	0.640	0.625	0.516	0.899	0.783	0.740	0.764	0.754
	0.978	1.395	1.010	1.012	1.180	0.957	0.680	0.630	0.630	0.599	0.549	0.905	0.765	0.745	0.759	0.742
	0.975	1.028	1.090	1.010	0.998	1.014	0.645	0.556	0.581	0.613	0.499	0.898	0.749	0.778	0.763	0.820
	0.972	1.039	1.079	1.029	1.007	1.018	0.641	0.537	0.561	0.620	0.512	0.895	0.756	0.764	0.751	0.666
	0.970	1.024	1.068	1.021	1.008	1.005	0.654	0.603	0.610	0.626	0.518	0.892	0.764	0.751	0.740	0.756
	1.001	1.179	1.150	1.006	0.825	1.060	0.651	0.695	0.655	0.630	0.508	0.882	0.797	0.741	0.699	0.743
	0.995	1.188	1.156	1.012	0.808	1.047	0.679	0.690	0.658	0.620	0.521	0.895	0.786	0.760	0.763	0.749
	0.998	1.196	1.139	1.002	0.816	1.053	0.664	0.693	0.657	0.625	0.524	0.889	0.789	0.751	0.769	0.750
**48h**																
	0.600	0.530	0.500	0.450	0.458	0.393	0.553	0.539	0.517	0.516	0.506	0.581	0.476	0.412	0.494	0.410
	0.596	0.494	0.492	0.460	0.449	0.406	0.541	0.548	0.505	0.500	0.495	0.556	0.470	0.420	0.487	0.418
	0.575	0.556	0.496	0.420	0.465	0.401	0.534	0.540	0.528	0.492	0.492	0.553	0.481	0.425	0.478	0.417
	0.560	0.486	0.496	0.560	0.442	0.453	0.539	0.593	0.517	0.490	0.508	0.563	0.408	0.430	0.419	0.420
	0.551	0.490	0.492	0.554	0.449	0.458	0.549	0.572	0.533	0.502	0.495	0.558	0.417	0.435	0.422	0.418

EXCLI Journal 2020;19:48-70 – ISSN 1611-2156
Received: October 03, 2019, accepted: December 19, 2019, published: January 03, 2020

	0.562	0.506	0.488	0.549	0.457	0.463	0.550	0.586	0.548	0.499	0.483	0.553	0.425	0.442	0.410	0.417
	0.580	0.565	0.483	0.405	0.460	0.426	0.540	0.493	0.563	0.483	0.502	0.564	0.431	0.459	0.344	0.407
	0.569	0.555	0.492	0.428	0.471	0.443	0.546	0.509	0.533	0.497	0.485	0.543	0.447	0.439	0.356	0.428
	0.575	0.560	0.488	0.416	0.465	0.419	0.550	0.501	0.548	0.490	0.490	0.553	0.462	0.452	0.341	0.417
**72h**																
	0.440	0.448	0.431	0.430	0.428	0.379	0.336	0.376	0.379	0.359	0.274	0.319	0.301	0.290	0.289	0.284
	0.429	0.438	0.425	0.411	0.432	0.382	0.329	0.368	0.372	0.345	0.294	0.324	0.298	0.272	0.291	0.286
	0.428	0.430	0.488	0.420	0.422	0.375	0.345	0.379	0.356	0.360	0.290	0.331	0.310	0.281	0.286	0.275
	0.435	0.404	0.409	0.452	0.369	0.357	0.336	0.402	0.379	0.394	0.360	0.323	0.303	0.330	0.299	0.284
	0.424	0.397	0.402	0.441	0.373	0.366	0.340	0.407	0.370	0.397	0.349	0.328	0.300	0.325	0.309	0.289
	0.446	0.393	0.416	0.464	0.365	0.349	0.333	0.398	0.390	0.390	0.360	0.319	0.315	0.340	0.305	0.279
	0.433	0.482	0.421	0.391	0.398	0.350	0.501	0.324	0.369	0.381	0.359	0.318	0.306	0.303	0.304	0.281
	0.429	0.477	0.429	0.382	0.408	0.355	0.483	0.312	0.359	0.375	0.425	0.324	0.300	0.299	0.318	0.277
	0.426	0.472	0.439	0.390	0.390	0.362	0.495	0.301	0.348	0.368	0.492	0.330	0.295	0.295	0.315	0.300

EXCLI Journal 2020;19:48-70 – ISSN 1611-2156
Received: October 03, 2019, accepted: December 19, 2019, published: January 03, 2020

Table 3: Effect of investigated naphthoquinones α-methylbutyrylshikonin (***1***), acetylshikonin (***2***) and β-hydroxy-isovalerylshikonin (***3***) on superoxide anion radical ($O_2^{\cdot-}$) production in the HCT-116 and MDA-MB-231 cell lines during 24 h, 48 h and 72 h of exposure, compared to non-treated control cells. Concentrations are expressed as nmol/mL.

Compound concentration (µM)	concentration (nmol/mL)					
	HCT-116			MDA-MB-231		
	24 h	48 h	72 h	24 h	48 h	72 h
1						
0	89.91±0.46	88.70±1.74	63.07±2.21	78.66±0.33	45.93±0.43	34.56±0.19
0.1	126.00±2.12*	80.13±4.71	62.63±0.16	96.27±4.20*	42.15±0.88*	35.03±0.92
1	119.50±7.42*	76.46±7.09	58.05±0.53*	86.29±1.62*	39.35±0.13*	34.30±0.66
10	126.94±0.36*	74.82±2.82*	58.09±0.93*	81.04±0.23*	37.70±1.72*	33.60±0.77
50	93.66±1.19*	118.17±1.62*	57.59±0.70	80.30±4.33	36.59±0.25*	31.86±0.69*
100	90.69±0.97	94.89±1.10*	53.97±0.68*	80.98±1.04*	34.33±0.70*	29.09±0.33*
2						
0	89.91±0.46	88.70±1.74	63.07±2.21	78.66±0.33	45.93±0.43	34.56±0.19
0.1	77.71±1.26*	87.32±1.09	34.32±1.13*	53.12±0.47*	43.55±0.16*	31.09±2.09
1	74.39±1.30*	86.79±0.69	32.45±2.65*	50.32±1.76*	43.40±0.97	29.03±1.08*
10	60.87±4.31*	79.42±2.59*	22.79±0.60*	49.65±0.96*	42.58±0.48*	29.52±0.34*
50	54.49±5.72*	78.20±1.82*	22.69±0.49*	49.59±0.25*	39.71±0.26*	29.96±0.48*
100	47.24±2.50*	75.22±1.00*	22.46±0.47*	41.39±0.37*	39.59±0.23*	28.46±1.84*
3						
0	89.91±0.46	88.70±1.74	63.07±2.21	78.66±0.33	45.93±0.43	34.56±0.19*
0.1	89.48±0.21	77.99±8.79	61.70±1.31	71.54±0.17*	44.65±0.28*	25.94±0.13*
1	87.98±1.17	72.74±5.76*	57.57±3.23*	61.85±0.43*	35.70±0.72*	24.24±0.17*
10	87.34±1.19	70.77±9.27*	51.08±3.55*	60.07±0.39*	34.78±0.40*	24.32±0.62*
50	85.04±1.17*	68.41±5.63*	42.40±3.95*	60.04±0.57*	33.33±1.62*	24.14±0.30*
100	65.37±1.12*	53.47±2.91*	22.50±1.83*	59.78±1.04*	33.34±0.16*	22.70±0.20*

Results are mean values ± SE from three experiments. *$p<0.05$

EXCLI Journal 2020;19:48-70 – ISSN 1611-2156
Received: October 03, 2019, accepted: December 19, 2019, published: January 03, 2020

Raw tables concerning **Figure 3**. Effects of investigated naphthoquinones on HCT-116 and MDA-MB-231 cell lines, expressed as the nmol NO_2^- /mL after 24 h, 48 h and 72 h of treatment. The cells were treated with α-methylbutyrylshikonin (*1*), acetylshikonin (*2*) and β-hydroxy-isovalerylshikonin (*3*) in concentration range from 0.1 to 100 μg/mL. Results were expressed as the means ± SE from three independent determinations.

Table 1: Observed absorbances of effects of α-methylbutyrylshikonin (***1***), acetylshikonin (***2***) and β-hydroxy-isovalerylshikonin (***3***) (in concentration range from 0.1 to 100 μg/mL) on nitric oxide production by HCT-116 cell line after 24 h, 48 h and 72 h of treatment.

Time of treatment	Concentration (μg/mL)															
	control	α-methylbutyrylshikonin					acetylshikonin					β-hydroxy-isovalerylshikonin				
	0	*0.1*	*1*	*10*	*50*	*100*	*0.1*	*1*	*10*	*50*	*100*	*0.1*	*1*	*10*	*50*	*100*
**24h**																
	0.031	0.037	0.033	0.044	0.053	0.052	0.028	0.036	0.044	0.055	0.098	0.036	0.033	0.046	0.080	0.086
	0.031	0.030	0.038	0.045	0.048	0.051	0.027	0.038	0.042	0.061	0.090	0.034	0.033	0.039	0.073	0.099
	0.031	0.036	0.043	0.039	0.055	0.054	0.031	0.033	0.041	0.050	0.093	0.030	0.032	0.044	0.086	0.090
	0.031	0.037	0.039	0.045	0.051	0.050	0.028	0.036	0.044	0.064	0.098	0.036	0.032	0.036	0.058	0.086
	0.030	0.033	0.040	0.037	0.043	0.058	0.035	0.030	0.042	0.060	0.101	0.039	0.033	0.039	0.061	0.088
	0.031	0.039	0.048	0.034	0.047	0.054	0.031	0.033	0.049	0.061	0.094	0.043	0.033	0.038	0.060	0.090
	0.031	0.039	0.033	0.040	0.050	0.050	0.040	0.036	0.044	0.051	0.098	0.032	0.039	0.046	0.079	0.086
	0.035	0.037	0.034	0.035	0.041	0.047	0.031	0.033	0.042	0.061	0.107	0.030	0.043	0.039	0.076	0.082
	0.031	0.033	0.030	0.031	0.047	0.054	0.037	0.031	0.045	0.056	0.100	0.040	0.049	0.036	0.070	0.088
**48h**																
	0.050	0.058	0.062	0.062	0.070	0.067	0.072	0.073	0.091	0.100	0.113	0.059	0.061	0.092	0.105	0.116
	0.049	0.055	0.065	0.058	0.066	0.075	0.069	0.071	0.096	0.103	0.122	0.056	0.064	0.090	0.099	0.121
	0.053	0.059	0.060	0.066	0.076	0.059	0.075	0.075	0.085	0.096	0.119	0.061	0.060	0.096	0.103	0.110
	0.049	0.057	0.047	0.062	0.070	0.067	0.072	0.068	0.091	0.100	0.118	0.059	0.057	0.077	0.091	0.106
	0.040	0.056	0.046	0.058	0.066	0.072	0.067	0.070	0.094	0.109	0.121	0.058	0.055	0.071	0.085	0.107
	0.044	0.055	0.045	0.054	0.063	0.076	0.064	0.074	0.098	0.118	0.125	0.049	0.051	0.065	0.085	0.108
	0.051	0.047	0.067	0.069	0.070	0.067	0.072	0.090	0.091	0.090	0.132	0.054	0.061	0.077	0.099	0.102
	0.050	0.035	0.065	0.059	0.067	0.077	0.087	0.091	0.094	0.093	0.127	0.055	0.054	0.071	0.094	0.103
	0.056	0.046	0.060	0.066	0.076	0.069	0.075	0.085	0.055	0.093	0.134	0.049	0.053	0.085	0.098	0.099
**72h**																
	0.050	0.051	0.054	0.058	0.056	0.059	0.050	0.053	0.045	0.063	0.104	0.049	0.049	0.051	0.084	0.117
	0.049	0.054	0.054	0.054	0.062	0.061	0.046	0.050	0.051	0.054	0.100	0.053	0.050	0.040	0.068	0.129

EXCLI Journal 2020;19:48-70 – ISSN 1611-2156
Received: October 03, 2019, accepted: December 19, 2019, published: January 03, 2020

Time of treatment	Concentration (μg/mL)															
	control	α-methylbutyrylshikonin					acetylshikonin					β-hydroxy-isovalerylshikonin				
	0	*0.1*	*1*	*10*	*50*	*100*	*0.1*	*1*	*10*	*50*	*100*	*0.1*	*1*	*10*	*50*	*100*
	0.045	0.058	0.056	0.045	0.069	0.064	0.042	0.060	0.058	0.067	0.103	0.053	0.044	0.045	0.080	0.125
	0.056	0.058	0.054	0.058	0.056	0.059	0.054	0.054	0.055	0.063	0.104	0.049	0.056	0.055	0.084	0.117
	0.059	0.054	0.052	0.054	0.055	0.061	0.055	0.050	0.052	0.059	0.100	0.053	0.053	0.055	0.081	0.119
	0.052	0.054	0.056	0.062	0.057	0.057	0.052	0.059	0.058	0.068	0.108	0.045	0.059	0.056	0.089	0.114
	0.052	0.058	0.054	0.052	0.054	0.059	0.054	0.054	0.055	0.063	0.104	0.049	0.050	0.053	0.084	0.117
	0.049	0.054	0.051	0.054	0.055	0.061	0.055	0.050	0.053	0.054	0.096	0.057	0.045	0.055	0.081	0.119
	0.045	0.051	0.056	0.057	0.057	0.064	0.057	0.059	0.058	0.062	0.100	0.053	0.056	0.058	0.078	0.120

Table 2: Observed absorbances of effects of α-methylbutyrylshikonin (***1***), acetylshikonin (***2***) and β-hydroxy-isovalerylshikonin (***3***) (in concentration range from 0.1 to 100 μg/mL) on nitric oxide production by MDA-MB-231 cell line after 24 h, 48 h and 72 h of treatment.

Time of treatment	Concentration (μg/mL)															
	control	α-methylbutyrylshikonin					acetylshikonin					β-hydroxy-isovalerylshikonin				
	0	*0.1*	*1*	*10*	*50*	*100*	*0.1*	*1*	*10*	*50*	*100*	*0.1*	*1*	*10*	*50*	*100*
**24h**																
	0.063	0.060	0.058	0.070	0.055	0.087	0.066	0.069	0.074	0.095	0.154	0.057	0.073	0.075	0.099	0.121
	0.060	0.054	0.049	0.067	0.070	0.101	0.080	0.080	0.066	0.089	0.151	0.075	0.061	0.064	0.098	0.111
	0.066	0.053	0.051	0.057	0.067	0.086	0.071	0.079	0.089	0.080	0.140	0.062	0.076	0.078	0.108	0.101
	0.055	0.062	0.052	0.060	0.075	0.099	0.059	0.069	0.080	0.083	0.154	0.057	0.058	0.053	0.089	0.124
	0.050	0.060	0.046	0.067	0.072	0.108	0.066	0.073	0.070	0.099	0.159	0.055	0.064	0.067	0.093	0.116
	0.059	0.063	0.058	0.061	0.074	0.090	0.052	0.072	0.092	0.103	0.149	0.060	0.051	0.040	0.084	0.132
	0.058	0.060	0.080	0.068	0.075	0.099	0.062	0.069	0.071	0.095	0.151	0.057	0.058	0.050	0.083	0.128
	0.053	0.058	0.078	0.077	0.072	0.106	0.058	0.080	0.064	0.099	0.155	0.049	0.064	0.067	0.078	0.118
	0.049	0.056	0.090	0.079	0.069	0.101	0.055	0.090	0.075	0.102	0.159	0.040	0.070	0.084	0.085	0.109
**48h**																
	0.060	0.065	0.055	0.074	0.088	0.106	0.066	0.064	0.102	0.102	0.178	0.089	0.090	0.098	0.108	0.237
	0.062	0.071	0.060	0.071	0.083	0.085	0.059	0.056	0.098	0.106	0.176	0.087	0.086	0.101	0.115	0.248
	0.071	0.060	0.051	0.080	0.093	0.126	0.073	0.074	0.105	0.099	0.179	0.092	0.101	0.096	0.101	0.225
	0.062	0.050	0.106	0.075	0.076	0.079	0.066	0.069	0.103	0.113	0.178	0.079	0.079	0.090	0.126	0.237
	0.066	0.055	0.093	0.078	0.083	0.085	0.059	0.082	0.100	0.117	0.184	0.066	0.090	0.076	0.122	0.248

EXCLI Journal 2020;19:48-70 – ISSN 1611-2156
Received: October 03, 2019, accepted: December 19, 2019, published: January 03, 2020

Time of treatment	Concentration (µg/mL)															
	control	α-methylbutyrylshikonin					acetylshikonin					β-hydroxy-isovalerylshikonin				
	0	*0.1*	*1*	*10*	*50*	*100*	*0.1*	*1*	*10*	*50*	*100*	*0.1*	*1*	*10*	*50*	*100*
	0.070	0.056	0.097	0.081	0.091	0.091	0.053	0.075	0.090	0.121	0.179	0.058	0.087	0.092	0.119	0.260
	0.050	0.068	0.055	0.095	0.080	0.069	0.060	0.096	0.102	0.115	0.229	0.059	0.079	0.076	0.104	0.240
	0.058	0.059	0.057	0.098	0.061	0.090	0.050	0.096	0.098	0.119	0.218	0.067	0.085	0.082	0.102	0.228
	0.070	0.061	0.061	0.098	0.071	0.073	0.055	0.078	0.076	0.122	0.211	0.080	0.070	0.093	0.107	0.220
**72h**																
	0.075	0.060	0.070	0.089	0.085	0.103	0.075	0.070	0.080	0.068	0.073	0.080	0.100	0.110	0.115	0.170
	0.070	0.063	0.067	0.085	0.100	0.110	0.073	0.074	0.076	0.072	0.082	0.076	0.110	0.096	0.110	0.177
	0.082	0.070	0.073	0.075	0.101	0.117	0.072	0.079	0.074	0.075	0.082	0.071	0.100	0.088	0.103	0.152
	0.075	0.064	0.074	0.083	0.098	0.110	0.073	0.074	0.076	0.078	0.091	0.080	0.066	0.090	0.124	0.157
	0.066	0.075	0.082	0.072	0.105	0.119	0.072	0.074	0.071	0.080	0.086	0.075	0.063	0.076	0.138	0.152
	0.058	0.086	0.079	0.080	0.100	0.123	0.073	0.074	0.076	0.086	0.088	0.072	0.059	0.070	0.141	0.146
	0.075	0.069	0.070	0.068	0.095	0.110	0.073	0.074	0.075	0.072	0.096	0.076	0.077	0.096	0.110	0.172
	0.066	0.078	0.078	0.078	0.115	0.112	0.072	0.074	0.079	0.078	0.088	0.065	0.065	0.078	0.134	0.182
	0.075	0.086	0.070	0.058	0.125	0.103	0.075	0.079	0.072	0.076	0.090	0.060	0.070	0.082	0.128	0.191

EXCLI Journal 2020;19:48-70 – ISSN 1611-2156
Received: October 03, 2019, accepted: December 19, 2019, published: January 03, 2020

Table 3: Effect of investigated naphthoquinones α-methylbutyrylshikonin (***1***), acetylshikonin (***2***) and β-hydroxy-isovalerylshikonin (***3***) on the nitrite (NO_2^-) production in the HCT-116 and MDA-MB-231 cell lines during 24 h, 48 h and 72 h of exposure, compared to non-treated control cells. Concentrations are expressed as nmol/mL.

Compound concentration (µM)	concentration (nmol/mL)					
	HCT-116			MDA-MB-231		
	24 h	48 h	72 h	24 h	48 h	72 h
1						
0	8.87±0.15	13.81±0.44	14.31±0.43	16.00±0.54	17.79±0.63	20.07±0.67
0.1	10.05±0.29*	14.63±0.75	15.42±0.25*	16.50±0.33	17.06±0.61	20.38±0.91
1	10.65±0.53*	16.16±0.82*	15.28±0.17	17.61±1.50	19.87±2.01	20.75±0.47
10	10.99±0.48*	17.38±0.44*	15.44±0.46	19.02±0.69*	23.56±1.00*	21.62±0.89
50	13.68±0.41*	19.50±0.41*	16.30±0.45*	19.71±0.58*	22.72±0.94*	28.91±1.09*
100	14.75±0.30*	19.72±0.56*	17.06±0.22*	27.45±0.76*	25.18±1.64*	31.55±0.64*
2						
0	8.87±0.15	13.81±0.44	14.31±0.43	16.00±0.54	17.79±0.63	20.07±0.67
0.1	9.04±0.42	20.42±0.61*	14.56±0.46	17.80±0.81*	16.91±0.66	20.60±0.11
1	9.60±0.24*	21.86±0.83*	15.32±0.37	21.34±0.68*	21.61±1.27*	21.05±0.27
10	12.21±0.24*	24.85±1.23*	15.13±0.40	21.30±0.92*	27.38±0.84*	21.27±0.29
50	16.27±0.45*	28.25±0.84*	17.35±0.49*	26.49±0.76*	31.78±0.80*	21.45±0.50
100	27.57±0.49*	34.79±0.63*	28.82±0.34*	42.99±0.54*	54.24±1.95*	24.28±0.63*
3						
0	8.87±0.15	13.81±0.44*	14.31±0.43	16.00±0.54	17.79±0.63	20.07±0.67
0.1	10.04±0.43	15.66±0.41*	14.45±0.33	16.03±0.90	21.16±1.23*	20.51±0.61
1	10.24±0.58*	16.12±0.40*	14.44±0.49	18.01±0.74*	24.04±0.82*	22.23±1.80
10	11.33±0.37*	22.68±1.00*	14.64±0.54	18.12±1.34	25.15±0.87*	24.57±1.15*
50	20.13±0.93*	26.90±0.68*	22.77±0.54*	25.64±0.90*	31.47±0.86*	34.56±1.28*
100	24.93±0.43*	30.44±0.65*	33.77±0.43*	33.21±0.92*	67.16±1.89*	46.91±1.45*

Results are mean values ± SE from three experiments. *p<0.05

EXCLI Journal 2020;19:48-70 – ISSN 1611-2156
Received: October 03, 2019, accepted: December 19, 2019, published: January 03, 2020

Raw tables concerning **Figure 4.** Effects of investigated naphthoquinones on the concentration of reduced glutathione (GSH) after 24 h, 48 h and 72 h of treatment. The HCT-116 and MDA-MB-231 cells were treated with α-methylbutyrylshikonin (***1***), acetylshikonin (***2***) and β-hydroxy-isovalerylshikonin (***3***) in concentration range from 0.1 to 100 μg/mL. Results were expressed as the means ± SE from three independent determinations.

Table 1: Observed absorbances in HCT-116 cell line of effects of α-methylbutyrylshikonin (***1***), acetylshikonin (***2***) and β-hydroxy-isovalerylshikonin (***3***) (in concentration range from 0.1 to 100 μg/mL) on the concentration of reduced glutathione (GSH) after 24 h, 48 h and 72 h of treatment.

Time of treatment	Concentration (μg/mL)															
	control	α-methylbutyrylshikonin					acetylshikonin					β-hydroxy-isovalerylshikonin				
	0	*0.1*	*1*	*10*	*50*	*100*	*0.1*	*1*	*10*	*50*	*100*	*0.1*	*1*	*10*	*50*	*100*
**24h**																
	0.167	0.218	0.183	0.194	0.170	0.169	0.162	0.186	0.162	0.177	0.160	0.174	0.170	0.174	0.170	0.165
	0.168	0.208	0.179	0.181	0.191	0.183	0.184	0.190	0.182	0.168	0.161	0.176	0.177	0.165	0.173	0.163
	0.165	0.212	0.185	0.192	0.183	0.179	0.168	0.174	0.171	0.187	0.165	0.180	0.170	0.175	0.168	0.170
	0.170	0.218	0.181	0.190	0.172	0.165	0.186	0.198	0.170	0.177	0.176	0.177	0.170	0.174	0.167	0.165
	0.160	0.222	0.179	0.194	0.193	0.176	0.199	0.179	0.158	0.161	0.169	0.171	0.166	0.160	0.165	0.160
	0.151	0.214	0.176	0.197	0.187	0.166	0.198	0.190	0.167	0.152	0.163	0.172	0.168	0.165	0.162	0.164
	0.147	0.206	0.198	0.164	0.173	0.160	0.201	0.193	0.174	0.161	0.150	0.177	0.170	0.171	0.166	0.165
	0.147	0.210	0.198	0.171	0.165	0.171	0.221	0.183	0.182	0.156	0.169	0.176	0.176	0.163	0.162	0.161
	0.163	0.196	0.201	0.160	0.179	0.153	0.215	0.181	0.165	0.158	0.156	0.183	0.154	0.170	0.154	0.166
**48h**																
	0.288	0.314	0.298	0.301	0.278	0.285	0.295	0.299	0.319	0.286	0.289	0.290	0.291	0.301	0.300	0.271
	0.285	0.304	0.280	0.294	0.295	0.284	0.298	0.296	0.298	0.290	0.269	0.295	0.304	0.300	0.298	0.286
	0.293	0.316	0.294	0.299	0.294	0.302	0.303	0.300	0.309	0.282	0.282	0.305	0.299	0.295	0.295	0.280
	0.286	0.305	0.295	0.298	0.282	0.273	0.332	0.352	0.286	0.296	0.280	0.309	0.296	0.299	0.298	0.279
	0.282	0.300	0.298	0.294	0.278	0.286	0.328	0.339	0.292	0.288	0.292	0.320	0.318	0.301	0.282	0.288
	0.276	0.296	0.300	0.291	0.276	0.282	0.315	0.330	0.299	0.279	0.303	0.319	0.325	0.302	0.287	0.296
	0.281	0.303	0.280	0.268	0.319	0.291	0.315	0.300	0.309	0.299	0.278	0.329	0.303	0.304	0.273	0.299
	0.289	0.309	0.276	0.278	0.289	0.283	0.320	0.298	0.289	0.286	0.298	0.314	0.296	0.299	0.280	0.283
	0.282	0.300	0.291	0.276	0.278	0.278	0.329	0.290	0.295	0.288	0.292	0.326	0.313	0.305	0.285	0.288

EXCLI Journal 2020;19:48-70 – ISSN 1611-2156
Received: October 03, 2019, accepted: December 19, 2019, published: January 03, 2020

Time of treatment	Concentration (μg/mL)															
	control	α-methylbutyrylshikonin					acetylshikonin					β-hydroxy-isovalerylshikonin				
	0	*0.1*	*1*	*10*	*50*	*100*	*0.1*	*1*	*10*	*50*	*100*	*0.1*	*1*	*10*	*50*	*100*
**72h**																
	0.285	0.350	0.348	0.339	0.325	0.294	0.300	0.296	0.300	0.298	0.310	0.396	0.304	0.326	0.298	0.286
	0.296	0.368	0.362	0.327	0.320	0.301	0.320	0.300	0.308	0.303	0.305	0.386	0.336	0.336	0.294	0.298
	0.299	0.371	0.341	0.339	0.312	0.310	0.314	0.304	0.319	0.295	0.294	0.386	0.350	0.340	0.286	0.304
	0.300	0.380	0.368	0.329	0.295	0.294	0.327	0.305	0.298	0.297	0.299	0.323	0.334	0.300	0.298	0.289
	0.291	0.375	0.362	0.323	0.292	0.301	0.320	0.299	0.304	0.303	0.292	0.316	0.330	0.298	0.294	0.299
	0.298	0.381	0.356	0.330	0.289	0.309	0.312	0.289	0.310	0.310	0.286	0.309	0.345	0.295	0.290	0.305
	0.300	0.388	0.345	0.328	0.320	0.284	0.298	0.320	0.298	0.289	0.289	0.365	0.356	0.350	0.300	0.297
	0.298	0.379	0.340	0.331	0.305	0.301	0.320	0.315	0.290	0.301	0.299	0.346	0.345	0.329	0.323	0.311
	0.299	0.376	0.349	0.359	0.312	0.293	0.318	0.318	0.294	0.295	0.294	0.355	0.350	0.340	0.312	0.304

Table 2: Observed absorbances in MDA-MB-231 cell line of effects of α-methylbutyrylshikonin (***1***), acetylshikonin (***2***) and β-hydroxy-isovalerylshikonin (***3***) (in concentration range from 0.1 to 100 μg/mL) on the concentration of reduced glutathione (GSH) after 24 h, 48 h and 72 h of treatment.

Time of treatment	Concentration (μg/mL)															
	control	α-methylbutyrylshikonin					acetylshikonin					β-hydroxy-isovalerylshikonin				
	0	*0.1*	*1*	*10*	*50*	*100*	*0.1*	*1*	*10*	*50*	*100*	*0.1*	*1*	*10*	*50*	*100*
**24h**																
	0.240	0.272	0.276	0.277	0.261	0.281	0.289	0.281	0.272	0.272	0.246	0.288	0.266	0.276	0.268	0.262
	0.248	0.289	0.269	0.290	0.260	0.267	0.281	0.273	0.276	0.256	0.242	0.292	0.272	0.272	0.256	0.250
	0.256	0.269	0.264	0.264	0.259	0.275	0.274	0.280	0.280	0.261	0.238	0.298	0.278	0.268	0.245	0.245
	0.266	0.260	0.270	0.258	0.268	0.249	0.276	0.281	0.268	0.275	0.265	0.289	0.282	0.288	0.266	0.250
	0.248	0.262	0.269	0.273	0.270	0.260	0.271	0.278	0.273	0.256	0.257	0.293	0.287	0.279	0.259	0.245
	0.255	0.270	0.252	0.269	0.269	0.243	0.264	0.280	0.270	0.259	0.268	0.299	0.278	0.284	0.270	0.263
	0.262	0.272	0.280	0.250	0.272	0.252	0.290	0.281	0.280	0.248	0.263	0.284	0.277	0.253	0.268	0.272
	0.246	0.277	0.269	0.260	0.260	0.244	0.285	0.258	0.276	0.256	0.242	0.273	0.270	0.272	0.256	0.265
	0.256	0.270	0.264	0.269	0.269	0.230	0.297	0.268	0.264	0.263	0.269	0.289	0.278	0.266	0.260	0.245
**48h**																
	0.254	0.283	0.271	0.270	0.279	0.272	0.271	0.269	0.275	0.256	0.257	0.281	0.292	0.265	0.260	0.261
	0.250	0.275	0.293	0.262	0.276	0.265	0.278	0.277	0.271	0.256	0.270	0.290	0.303	0.290	0.256	0.250

EXCLI Journal 2020;19:48-70 – ISSN 1611-2156
Received: October 03, 2019, accepted: December 19, 2019, published: January 03, 2020

Time of treatment	Concentration (µg/mL)															
	control	α-methylbutyrylshikonin					acetylshikonin					β-hydroxy-isovalerylshikonin				
	0	*0.1*	*1*	*10*	*50*	*100*	*0.1*	*1*	*10*	*50*	*100*	*0.1*	*1*	*10*	*50*	*100*
	0.266	0.270	0.276	0.282	0.266	0.250	0.289	0.281	0.269	0.242	0.275	0.288	0.278	0.279	0.266	0.257
	0.255	0.294	0.271	0.260	0.260	0.259	0.276	0.269	0.265	0.256	0.258	0.310	0.295	0.293	0.276	0.259
	0.248	0.289	0.268	0.266	0.256	0.267	0.271	0.280	0.275	0.251	0.260	0.315	0.303	0.286	0.270	0.262
	0.240	0.297	0.266	0.273	0.251	0.269	0.281	0.283	0.272	0.261	0.263	0.320	0.313	0.279	0.264	0.267
	0.258	0.275	0.264	0.280	0.267	0.242	0.283	0.286	0.282	0.273	0.254	0.282	0.273	0.276	0.265	0.270
	0.265	0.281	0.297	0.266	0.256	0.265	0.296	0.277	0.279	0.292	0.270	0.280	0.291	0.282	0.270	0.266
	0.276	0.261	0.280	0.287	0.261	0.274	0.289	0.281	0.272	0.280	0.265	0.289	0.282	0.279	0.268	0.279
**72h**																
	0.296	0.342	0.359	0.330	0.326	0.329	0.330	0.298	0.299	0.291	0.318	0.361	0.325	0.328	0.297	0.297
	0.308	0.361	0.345	0.345	0.330	0.319	0.323	0.317	0.324	0.314	0.301	0.340	0.331	0.350	0.310	0.2893
	0.301	0.349	0.337	0.350	0.333	0.321	0.315	0.319	0.318	0.304	0.303	0.356	0.339	0.340	0.319	0.3012
	0.296	0.327	0.353	0.328	0.309	0.318	0.321	0.324	0.301	0.322	0.296	0.328	0.328	0.363	0.297	0.307
	0.305	0.335	0.343	0.329	0.318	0.309	0.329	0.317	0.307	0.317	0.311	0.340	0.331	0.350	0.313	0.3252
	0.315	0.343	0.332	0.330	0.328	0.299	0.341	0.311	0.313	0.312	0.325	0.352	0.335	0.336	0.319	0.312
	0.296	0.363	0.320	0.328	0.326	0.289	0.296	0.297	0.298	0.298	0.293	0.368	0.328	0.291	0.325	0.323
	0.320	0.350	0.343	0.329	0.318	0.309	0.330	0.320	0.317	0.317	0.311	0.351	0.348	0.310	0.310	0.3092
	0.319	0.356	0.332	0.334	0.330	0.281	0.313	0.315	0.325	0.307	0.303	0.359	0.339	0.301	0.321	0.3052

EXCLI Journal 2020;19:48-70 – ISSN 1611-2156
Received: October 03, 2019, accepted: December 19, 2019, published: January 03, 2020

Table 3: Effect of investigated naphthoquinones α-methylbutyrylshikonin (***1***), acetylshikonin (***2***) and β-hydroxy-isovalerylshikonin (***3***) on the concentration of reduced glutathione (GSH) in the HCT-116 and MDA-MB-231 cell lines during 24 h, 48 h and 72 h of exposure, compared to non-treated control cells. Concentrations are expressed as μmol/mL.

Compound concentration (μM)	concentration (μmol/mL) HCT-116			MDA-MB-231		
	24 h	48 h	72 h	24 h	48 h	72 h
1						
0	92.63±1.77	165.18±0.95	171.83±0.94	146.65±1.59	148.99±2.11	177.61±1.91
0.1	122.66±1.52*	176.99±1.32*	217.19±2.09*	157.20±1.65*	162.78±2.23*	201.33±2.26*
1	108.29±1.85*	168.24±1.73	204.30±1.94*	155.46±1.55*	160.24±2.24*	197.48±2.26*
10	105.85±2.72*	167.52±2.27	193.66±2.09*	155.26±2.27*	157.49±1.83*	193.42±1.62*
50	103.99±1.91*	166.81±2.68	178.51±2.56	153.88±1.00*	152.88±1.79	187.95±1.44*
100	98.07±1.81*	165.20±1.57	173.14±1.60	148.25±3.20	152.29±2.02	178.87±3.06
2						
0	92.63±1.77	165.18±0.95	171.83±0.94	146.65±1.59	148.99±2.11	177.61±1.91
0.1	111.78±3.81*	182.73±2.66*	182.19±1.86*	162.83±2.05*	163.24±1.67*	186.81±2.54*
1	107.77±1.43*	180.66±4.36*	176.95±2.04*	159.75±1.53*	161.25±1.12*	181.51±1.82
10	98.62±1.60	173.69±2.07*	175.35±1.72	158.37±1.05*	158.58±0.98*	180.55±2.01
50	96.46±2.27*	167.10±1.22	173.45±1.17	151.24±1.63	152.43±3.02	179.27±1.97
100	94.66±1.48	166.52±2.05	171.89±1.59	147.62±2.40	152.79±1.35	178.01±2.00
3						
0	92.63±1.77	165.18±0.95	171.83±0.94	146.65±1.59	148.99±2.11	177.61±1.91
0.1	102.18±0.75*	180.90±2.59*	205.03±6.26*	167.99±1.50*	171.12±3.00*	203.32±2.40*
1	97.96±1.30*	176.90±2.20*	196.55±3.00*	160.39±1.23*	169.48±2.48*	193.66±1.40*
10	97.83±1.04*	174.40±0.58*	187.82±4.02*	158.35±2.03*	162.91±1.56*	191.39±4.74*
50	95.71±1.08	167.46±1.86	173.65±2.21	151.27±1.56	154.23±1.16*	181.10±1.96
100	95.30±0.53	165.64±1.65	173.48±1.52	148.14±1.98	152.85±1.63	178.46±2.21

Results are mean values ± SE from three experiments. *$p<0.05$

EXCLI Journal 2020;19:48-70 – ISSN 1611-2156
Received: October 03, 2019, accepted: December 19, 2019, published: January 03, 2020

Raw tables concerning **Figure 5.** Effects of investigated naphthoquinones on the concentration of oxidized glutauione form (GSSG) after 24 h, 48 h and 72 h of treatment. The HCT-116 and MDA-MB-231 cells were treated with α-methylbutyrylshikonin (***1***), acetylshikonin (***2***) and β-hydroxy-isovalerylshikonin (***3***) in concentration range from 0.1 to 100 μg/mL. Results were expressed as the means ± SE from three independent determinations.

Table 1: Observed absorbances in HCT-116 cell line of effects of α-methylbutyrylshikonin (***1***), acetylshikonin (***2***) and β-hydroxy-isovalerylshikonin (***3***) (in concentration range from 0.1 to 100 μg/mL) on the concentration of oxidized glutauione form (GSSG) after 24 h, 48 h and 72 h of treatment.

Time of treatment	Concentration (μg/mL)															
	control	α-methylbutyrylshikonin					acetylshikonin					β-hydroxy-isovalerylshikonin				
	0	*0.1*	*1*	*10*	*50*	*100*	*0.1*	*1*	*10*	*50*	*100*	*0.1*	*1*	*10*	*50*	*100*
**24h**																
	0.139	0.177	0.160	0.215	0.189	0.230	0.150	0.199	0.215	0.190	0.173	0.175	0.159	0.180	0.201	0.222
	0.130	0.161	0.173	0.192	0.183	0.191	0.149	0.168	0.228	0.189	0.163	0.171	0.157	0.165	0.195	0.209
	0.140	0.157	0.161	0.199	0.202	0.238	0.164	0.187	0.197	0.179	0.178	0.180	0.164	0.191	0.220	0.215
	0.156	0.190	0.178	0.180	0.189	0.189	0.140	0.149	0.149	0.199	0.213	0.160	0.174	0.158	0.205	0.200
	0.146	0.185	0.173	0.192	0.183	0.191	0.149	0.168	0.152	0.205	0.215	0.155	0.168	0.165	0.199	0.196
	0.136	0.182	0.169	0.181	0.178	0.194	0.159	0.167	0.166	0.190	0.199	0.164	0.194	0.173	0.189	0.203
	0.139	0.130	0.156	0.180	0.215	0.202	0.140	0.162	0.160	0.175	0.188	0.159	0.158	0.190	0.190	0.222
	0.148	0.139	0.167	0.190	0.198	0.195	0.139	0.168	0.165	0.170	0.202	0.173	0.168	0.174	0.192	0.202
	0.145	0.125	0.161	0.189	0.216	0.199	0.140	0.167	0.174	0.177	0.181	0.166	0.184	0.195	0.186	0.212
**48h**																
	0.202	0.520	0.415	0.286	0.361	0.241	0.433	0.380	0.310	0.267	0.200	0.529	0.469	0.445	0.232	0.217
	0.193	0.524	0.432	0.299	0.362	0.277	0.419	0.355	0.305	0.279	0.199	0.522	0.473	0.431	0.220	0.231
	0.212	0.517	0.421	0.295	0.375	0.204	0.451	0.406	0.322	0.255	0.202	0.530	0.465	0.459	0.245	0.202
	0.178	0.526	0.480	0.466	0.270	0.365	0.429	0.338	0.353	0.257	0.219	0.430	0.378	0.405	0.351	0.227
	0.173	0.521	0.473	0.453	0.280	0.345	0.412	0.349	0.342	0.241	0.229	0.425	0.381	0.414	0.341	0.241
	0.159	0.533	0.465	0.456	0.289	0.366	0.421	0.360	0.352	0.246	0.238	0.440	0.385	0.423	0.331	0.255
	0.199	0.400	0.460	0.382	0.338	0.289	0.431	0.436	0.689	0.354	0.202	0.350	0.405	0.369	0.429	0.220
	0.202	0.415	0.445	0.363	0.321	0.273	0.434	0.439	0.312	0.341	0.211	0.356	0.452	0.359	0.445	0.215
	0.170	0.428	0.439	0.365	0.331	0.270	0.420	0.445	0.349	0.357	0.226	0.374	0.433	0.350	0.450	0.230
**72h**																
	0.317	0.484	0.661	0.664	0.844	0.872	0.305	0.330	0.527	0.651	0.641	0.244	0.529	0.620	0.673	0.518
	0.323	0.478	0.677	0.670	0.855	0.879	0.290	0.334	0.519	0.644	0.656	0.251	0.520	0.615	0.665	0.525
	0.311	0.491	0.670	0.657	0.833	0.890	0.295	0.354	0.535	0.658	0.651	0.238	0.538	0.626	0.680	0.510
	0.266	0.449	0.699	0.730	0.695	0.880	0.265	0.230	0.515	0.638	0.660	0.269	0.510	0.790	0.650	0.720

EXCLI Journal 2020;19:48-70 – ISSN 1611-2156
Received: October 03, 2019, accepted: December 19, 2019, published: January 03, 2020

Time of treatment	Concentration (µg/mL)															
	control	α-methylbutyrylshikonin					acetylshikonin					β-hydroxy-isovalerylshikonin				
	0	*0.1*	*1*	*10*	*50*	*100*	*0.1*	*1*	*10*	*50*	*100*	*0.1*	*1*	*10*	*50*	*100*
	0.261	0.468	0.682	0.719	0.699	0.898	0.255	0.239	0.507	0.651	0.655	0.249	0.505	0.785	0.658	0.709
	0.270	0.458	0.690	0.730	0.697	0.889	0.260	0.234	0.509	0.644	0.657	0.259	0.508	0.772	0.645	0.715
	0.266	0.503	0.690	0.689	0.700	0.876	0.290	0.400	0.679	0.635	0.633	0.355	0.402	0.498	0.622	0.729
	0.251	0.507	0.682	0.684	0.720	0.885	0.292	0.404	0.689	0.639	0.642	0.350	0.405	0.493	0.616	0.731
	0.262	0.510	0.698	0.681	0.725	0.895	0.295	0.409	0.700	0.650	0.651	0.346	0.408	0.489	0.610	0.732

Table 2: Observed absorbances in MDA-MB-231 cell line of effects of α-methylbutyrylshikonin (***1***), acetylshikonin (***2***) and β-hydroxy-isovalerylshikonin (***3***) (in concentration range from 0.1 to 100 µg/mL) on the concentration of oxidized glutauione form (GSSG) after 24 h, 48 h and 72 h of treatment.

Time of treatment	Concentration (µg/mL)															
	control	α-methylbutyrylshikonin					acetylshikonin					β-hydroxy-isovalerylshikonin				
	0	*0.1*	*1*	*10*	*50*	*100*	*0.1*	*1*	*10*	*50*	*100*	*0.1*	*1*	*10*	*50*	*100*
**24h**																
	0.487	0.511	0.493	0.501	0.495	0.490	0.505	0.530	0.486	0.514	0.471	0.490	0.493	0.480	0.493	0.476
	0.482	0.514	0.490	0.496	0.519	0.481	0.518	0.495	0.496	0.498	0.469	0.503	0.482	0.498	0.500	0.470
	0.479	0.508	0.476	0.508	0.503	0.467	0.516	0.563	0.509	0.510	0.470	0.479	0.501	0.489	0.498	0.450
	0.481	0.509	0.549	0.516	0.494	0.510	0.499	0.499	0.479	0.489	0.478	0.498	0.508	0.495	0.489	0.492
	0.478	0.516	0.550	0.510	0.497	0.498	0.502	0.495	0.489	0.482	0.502	0.502	0.497	0.498	0.495	0.489
	0.503	0.509	0.548	0.532	0.490	0.495	0.495	0.503	0.501	0.509	0.491	0.490	0.520	0.486	0.483	0.505
	0.482	0.521	0.499	0.502	0.499	0.487	0.526	0.490	0.490	0.495	0.499	0.526	0.508	0.495	0.489	0.495
	0.472	0.525	0.490	0.499	0.505	0.481	0.509	0.493	0.520	0.501	0.508	0.519	0.487	0.508	0.469	0.492
	0.492	0.515	0.498	0.495	0.486	0.475	0.526	0.501	0.549	0.499	0.517	0.512	0.466	0.490	0.479	0.505
**48h**																
	0.512	0.520	0.580	0.507	0.509	0.548	0.487	0.474	0.504	0.560	0.475	0.549	0.471	0.502	0.530	0.546
	0.513	0.529	0.578	0.495	0.522	0.556	0.479	0.468	0.509	0.556	0.469	0.519	0.477	0.490	0.519	0.552
	0.510	0.511	0.585	0.519	0.499	0.541	0.496	0.480	0.499	0.566	0.490	0.578	0.489	0.493	0.537	0.539
	0.512	0.515	0.471	0.490	0.451	0.478	0.552	0.520	0.542	0.565	0.576	0.519	0.536	0.492	0.529	0.565
	0.509	0.509	0.478	0.495	0.462	0.499	0.542	0.519	0.555	0.558	0.571	0.515	0.547	0.481	0.520	0.541
	0.519	0.502	0.493	0.499	0.473	0.508	0.545	0.506	0.549	0.555	0.560	0.503	0.558	0.485	0.505	0.546
	0.515	0.509	0.496	0.527	0.550	0.571	0.505	0.574	0.519	0.460	0.598	0.495	0.548	0.595	0.554	0.556

EXCLI Journal 2020;19:48-70 – ISSN 1611-2156
Received: October 03, 2019, accepted: December 19, 2019, published: January 03, 2020

Time of treatment	Concentration (µg/mL)															
	control	α-methylbutyrylshikonin					acetylshikonin					β-hydroxy-isovalerylshikonin				
	0	*0.1*	*1*	*10*	*50*	*100*	*0.1*	*1*	*10*	*50*	*100*	*0.1*	*1*	*10*	*50*	*100*
	0.518	0.515	0.481	0.509	0.562	0.585	0.516	0.546	0.529	0.456	0.590	0.485	0.547	0.582	0.538	0.546
	0.502	0.511	0.493	0.519	0.578	0.577	0.525	0.570	0.508	0.469	0.599	0.489	0.535	0.593	0.549	0.531
**72h**																
	0.795	0.772	0.708	0.880	0.882	0.874	0.751	0.863	0.920	0.856	0.960	0.722	0.753	0.959	0.961	0.844
	0.786	0.765	0.760	0.879	0.872	0.895	0.779	0.865	0.910	0.860	0.973	0.730	0.762	0.952	0.966	0.855
	0.776	0.767	0.802	0.877	0.861	0.901	0.764	0.866	0.901	0.903	0.987	0.737	0.772	0.945	0.972	0.820
	0.759	0.759	0.929	0.871	0.869	0.916	0.760	0.777	0.770	0.936	0.866	0.848	0.795	0.716	0.700	0.958
	0.749	0.775	0.856	0.885	0.879	0.905	0.768	0.759	0.791	0.893	0.887	0.855	0.762	0.720	0.697	0.984
	0.769	0.767	0.834	0.877	0.860	0.909	0.764	0.763	0.770	0.915	0.870	0.851	0.789	0.708	0.703	0.967
	0.751	0.865	0.819	0.717	0.882	0.877	0.851	0.856	0.892	0.826	0.960	0.759	0.954	0.842	0.957	0.872
	0.768	0.873	0.799	0.735	0.871	0.868	0.877	0.865	0.908	0.837	0.973	0.749	0.925	0.829	0.966	0.878
	0.759	0.864	0.817	0.722	0.864	0.892	0.864	0.871	0.870	0.819	0.989	0.771	0.912	0.821	0.975	0.892

EXCLI Journal 2020;19:48-70 – ISSN 1611-2156
Received: October 03, 2019, accepted: December 19, 2019, published: January 03, 2020

Table 3: Effect of investigated naphthoquinones α-methylbutyrylshikonin (***1***), acetylshikonin (***2***) and β-hydroxy-isovalerylshikonin (***3***) on the concentration of oxidized glutathione (GSSG) in the HCT-116 and MDA-MB-231 cell lines during 24 h, 48 h and 72 h of exposure, compared to non-treated control cells. Concentrations are expressed as μmol/mL.

Compound concentration (μM)	concentration (μmol/mL) HCT-116			MDA-MB-231		
	24 h	48 h	72 h	24 h	48 h	72 h
1						
0	24.72±0.45	32.62±1.04	48.84±1.62	84.20±0.51	89.14±0.29	133.63±0.90
0.1	27.97±1.43	84.72±3.20*	84.08±1.26*	89.47±0.34*	89.34±0.45	139.32±2.91
1	28.96±0.42*	77.91±1.34*	118.88±0.73*	88.79±1.73*	90.00±2.82	141.59±3.55
10	33.23±0.64*	65.04±4.16*	120.31±1.63*	88.14±0.68*	88.16±0.75	143.88±4.47*
50	33.88±0.80*	56.57±2.22*	130.84±4.06*	86.77±0.56*	89.06±2.62	151.57±0.50*
100	35.35±1.05*	50.86±3.25*	153.95±0.51*	84.77±0.74	93.98±2.16	155.38±0.97*
2						
0	24.72±0.45	32.62±1.04	48.84±1.62	84.20±0.51	89.14±0.29	133.63±0.90
0.1	25.70±0.53	74.41±0.67*	49.24±1.04	88.88±0.66*	89.82±1.53	138.80±2.94
1	29.66±0.84*	67.81±2.46*	56.75±4.33	88.36±1.38*	90.02±2.30	144.68±2.87*
10	31.04±1.64*	64.46±7.02*	100.14±4.98*	87.37±1.25*	91.14±1.21	149.48±3.68*
50	32.37±0.67*	50.17±2.78*	112.33±0.43*	86.95±0.60*	91.72±2.86	151.70±2.41*
100	33.08±1.05*	37.21±0.84*	113.02±0.51*	85.13±1.06	95.27±3.13	163.65±2.95*
3						
0	24.72±0.45	32.62±1.04	48.84±1.62	84.20±0.51	89.14±0.29	133.63±0.90
0.1	29.05±0.49*	76.49±4.23*	49.52±2.90	87.35±0.88*	89.93±1.75	135.77±3.20
1	29.47±0.73*	74.26±2.32*	83.63±3.34*	86.25±0.94	90.99±1.98	143.55±4.69
10	30.76±0.76*	70.65±2.26*	109.96±7.28*	85.83±0.48	91.10±2.90	144.83±5.98
50	34.37±0.61*	58.86±5.28*	112.51±1.48*	84.93±0.57	92.40±0.88*	152.64±7.72*
100	36.36±0.55*	39.41±0.90*	113.85±5.97*	84.56±1.02	95.15±0.58*	156.01±3.41*

Results are mean values ± SE from three experiments. *p<0.05

Received: October 03, 2019, accepted: December 19, 2019, published: January 03, 2020

Raw tables concerning **Figure 6.** Absorption spectra of CT-DNA (1.77 x 10^{-5} M) before (purple dashed line) and after addition of α-methylbutyrylshikon (***1***), acetylshikonin (***2***) and *β*-hydroxyisovalerylshikonin (***3***) (0 - 1.80 x 10^{-5} M). Arrow shows the absorbance changes upon increasing concentration of *α*-methylbutyrylshikon (***1***), acetylshikonin (***2***) and *β*-hydroxyisovalerylshikonin (***3***).

Table 1: Absorption intensity in the wavelength range of 200-800 nm for absorption spectra of CT-DNA fixed concentration (1.77 x 10^{-5} M), in the absence (*A*) and presence of increasing concentration of α-methylbutyrylshikon (*B*-*G*)

Absorption intensity							
**Wavelength (nm)**	**concentration of α-methylbutyrylshikon (M)**						
	A	*B*	*C*	*D*	*E*	*F*	*G*
	0.00	4.0x10^{-6}	8.0x10^{-6}	1.0x10^{-5}	1.4x10^{-5}	1.6x10^{-5}	1.8x10^{-5}
800	-0.00205	-0.00103	-0.00102	0.001437	-0.00039	0.003144	0.004295
799	-0.00225	-0.00105	-0.00078	0.001397	-0.00014	0.003181	0.004353
798	-0.00198	-0.00079	-0.00082	0.001699	-7.1E-06	0.003388	0.004727
797	-0.00156	-0.00074	-0.0004	0.001736	0.000297	0.003587	0.004648
796	-0.00208	-0.00106	-0.00105	0.001593	-9.9E-05	0.003195	0.004267
795	-0.00186	-0.00077	-0.00084	0.001669	-0.00019	0.003371	0.004646
794	-0.00203	-0.00099	-0.00077	0.001581	-0.00017	0.00337	0.004412
793	-0.00194	-0.00113	-0.00132	0.001277	-0.00021	0.003166	0.004275
792	-0.00197	-0.00115	-0.00078	0.001644	-0.00011	0.003374	0.004433
791	-0.00231	-0.00101	-0.00099	0.001485	-7.5E-05	0.003258	0.004333
790	-0.00194	-0.00084	-0.00082	0.001672	-6.6E-05	0.003469	0.004659
789	-0.0021	-0.00098	-0.00093	0.001284	7.61E-06	0.003316	0.004602
788	-0.00226	-0.00116	-0.00077	0.001529	-2.8E-05	0.00346	0.004464
787	-0.00198	-0.00107	-0.00077	0.001758	-0.00024	0.003338	0.004503
786	-0.00228	-0.00076	-0.0009	0.001751	0.000123	0.003534	0.004635
785	-0.00221	-0.00067	-0.00078	0.001622	7E-05	0.003744	0.004564
784	-0.00202	-0.00086	-0.0007	0.001704	-0.00015	0.003792	0.00477
783	-0.00225	-0.00071	-0.00068	0.00161	4.28E-05	0.00343	0.00495
782	-0.00227	-0.0011	-0.00087	0.001596	-0.00011	0.003627	0.004478
781	-0.00239	-0.00094	-0.00076	0.00158	-0.00012	0.00343	0.004532
780	-0.00211	-0.00088	-0.00072	0.00163	0.000171	0.003623	0.004776
779	-0.002	-0.00104	-0.00074	0.001541	0.000103	0.003486	0.004634
778	-0.00212	-0.00092	-0.00046	0.001626	7.71E-05	0.003745	0.004798
777	-0.00207	-0.00072	-0.00085	0.001716	-0.00012	0.003591	0.004846
776	-0.00212	-0.00094	-0.00066	0.001545	9.64E-05	0.003819	0.004844
775	-0.00211	-0.0008	-0.00057	0.001719	0.000189	0.003843	0.004967
774	-0.00209	-0.00108	-0.00089	0.001654	5.95E-06	0.003677	0.004916
773	-0.00232	-0.00085	-0.0006	0.001598	0.000322	0.003849	0.004977
772	-0.00195	-0.00089	-0.00099	0.00171	-0.00015	0.0039	0.004822
771	-0.002	-0.00071	-0.00066	0.001722	6.63E-05	0.003894	0.005034
770	-0.00218	-0.00058	-0.00045	0.001991	0.000204	0.003838	0.005103
769	-0.00231	-0.00097	-0.00065	0.001677	-1.9E-05	0.003774	0.00509
768	-0.00188	-0.00071	-0.00056	0.001844	0.000312	0.00393	0.005091
767	-0.00218	-0.00056	-0.00042	0.001778	0.00018	0.003955	0.005131
766	-0.0019	-0.00063	-0.00044	0.001934	0.000346	0.004152	0.005333
765	-0.00208	-0.00092	-0.00069	0.001854	0.000268	0.003882	0.004883
764	-0.0022	-0.00078	-0.0006	0.001735	0.000246	0.004118	0.005106
763	-0.00212	-0.00085	-0.00061	0.001851	0.000127	0.003892	0.005117

Received: October 03, 2019, accepted: December 19, 2019, published: January 03, 2020

Wavelength (nm)	*Absorption intensity*						
	**concentration of α-methylbutyrylshikon (M)**						
	A	*B*	*C*	*D*	*E*	*F*	*G*
	0.00	$4.0x10^{-6}$	$8.0x10^{-6}$	$1.0x10^{-5}$	$1.4x10^{-5}$	$1.6x10^{-5}$	$1.8x10^{-5}$
762	-0.00215	-0.00059	-0.00023	0.00209	0.000427	0.004204	0.005379
761	-0.00216	-0.00075	-0.00058	0.002067	0.000221	0.004048	0.005182
760	-0.00196	-0.00052	-0.00034	0.001989	0.000549	0.004312	0.005353
759	-0.00209	-0.00085	-0.00055	0.00175	0.000171	0.004148	0.005473
758	-0.00231	-0.00094	-0.00064	0.001632	5.02E-05	0.003849	0.005017
757	-0.00209	-0.0008	-0.00035	0.001832	0.000484	0.003992	0.005304
756	-0.0022	-0.00075	-0.00042	0.001798	0.000561	0.004008	0.005363
755	-0.00226	-0.00076	-0.00049	0.001715	0.000311	0.004164	0.005381
754	-0.00204	-0.00048	-0.00049	0.001995	0.000425	0.004262	0.005615
753	-0.00211	-0.00071	-0.00052	0.002044	0.000666	0.004387	0.005515
752	-0.00206	-0.00078	-0.00021	0.002161	0.000436	0.004273	0.005486
751	-0.002	-0.00045	-0.00019	0.002005	0.000677	0.00436	0.005614
750	-0.00238	-0.00057	-0.00056	0.00211	0.000416	0.004273	0.005331
749	-0.0023	-0.00064	-0.00038	0.002085	0.000425	0.004466	0.005559
748	-0.00236	-0.00083	-0.00046	0.001996	0.000365	0.004275	0.005556
747	-0.0021	-0.00074	-0.00037	0.002079	0.000509	0.004358	0.005397
746	-0.00236	-0.001	-0.00058	0.001903	0.000318	0.004276	0.005429
745	-0.00214	-0.0005	-0.0002	0.002161	0.000688	0.004538	0.005855
744	-0.00215	-0.00062	-0.00036	0.002073	0.000561	0.00447	0.005643
743	-0.00222	-0.00066	-0.00036	0.002037	0.000537	0.004586	0.005714
742	-0.00218	-0.00045	-0.00036	0.002083	0.000581	0.004406	0.005748
741	-0.00224	-0.00065	-0.00022	0.002021	0.00059	0.00453	0.005781
740	-0.00208	-0.00054	-0.00019	0.002161	0.000776	0.004693	0.005942
739	-0.00208	-0.00048	-6.7E-05	0.002202	0.000807	0.004793	0.005962
738	-0.00222	-0.00047	-0.0002	0.002198	0.000817	0.004646	0.006003
737	-0.0022	-0.00053	-0.00027	0.002162	0.000752	0.004948	0.006132
736	-0.00229	-0.00041	4.23E-05	0.002264	0.000919	0.004785	0.00623
735	-0.00204	-0.00056	-0.00013	0.00217	0.000895	0.004868	0.006168
734	-0.00221	-0.0005	-0.00043	0.002006	0.000724	0.004602	0.005947
733	-0.00213	-0.00033	-4.5E-05	0.002276	0.001073	0.005009	0.006345
732	-0.00233	-0.00049	-0.00016	0.002166	0.000962	0.00496	0.006279
731	-0.00228	-0.00045	-0.00019	0.002146	0.000854	0.004849	0.00629
730	-0.00218	-0.00026	-2.8E-05	0.002389	0.000887	0.004944	0.006328
729	-0.0023	-0.00049	-0.00021	0.002204	0.000994	0.004937	0.006274
728	-0.00204	-0.00017	0.000255	0.00249	0.001123	0.005156	0.006435
727	-0.00224	-0.00025	-0.00016	0.002257	0.001103	0.005059	0.006313
726	-0.00205	-0.0002	8.24E-05	0.002409	0.001101	0.005128	0.006339
725	-0.00232	-0.00027	-1.3E-05	0.002345	0.001042	0.005167	0.006593
724	-0.00208	-0.00034	7.7E-05	0.002447	0.001191	0.005223	0.006679
723	-0.00213	-0.00051	0.000204	0.002461	0.001101	0.005238	0.006516
722	-0.00234	-0.00038	-2.5E-05	0.002444	0.001125	0.005165	0.006593
721	-0.00218	-0.00015	0.000275	0.002637	0.001281	0.00535	0.006708
720	-0.00226	-0.00014	0.000145	0.002647	0.001294	0.005484	0.006764
719	-0.00234	-0.00032	8.45E-05	0.002424	0.001293	0.005492	0.006806
718	-0.00225	-0.00014	0.000403	0.002597	0.001363	0.005588	0.006903
717	-0.00224	-4E-05	0.000361	0.002479	0.001419	0.005694	0.007079
716	-0.00214	-3.7E-05	0.000448	0.002678	0.001517	0.005751	0.007181
715	-0.00211	2.09E-05	0.000488	0.002763	0.001726	0.005823	0.007224

EXCLI Journal 2020;19:48-70 – ISSN 1611-2156
Received: October 03, 2019, accepted: December 19, 2019, published: January 03, 2020

Wavelength (nm)	***Absorption intensity***						
	**concentration of α-methylbutyrylshikon (M)**						
	A	*B*	*C*	*D*	*E*	*F*	*G*
	0.00	$4.0x10^{-6}$	$8.0x10^{-6}$	$1.0x10^{-5}$	$1.4x10^{-5}$	$1.6x10^{-5}$	$1.8x10^{-5}$
714	-0.00217	2.57E-05	0.000477	0.002718	0.001571	0.005792	0.007194
713	-0.00209	1.52E-05	0.000601	0.002943	0.001767	0.005937	0.007348
712	-0.00202	8.14E-05	0.000552	0.002884	0.001771	0.00617	0.007433
711	-0.00207	0.000124	0.000505	0.002819	0.00179	0.006064	0.00746
710	-0.00193	0.000235	0.000708	0.003044	0.001882	0.006378	0.007451
709	-0.00234	-8.8E-05	0.000439	0.002822	0.001713	0.006119	0.007456
708	-0.00178	0.000101	0.00065	0.003111	0.001933	0.006375	0.007692
707	-0.00185	0.000231	0.000703	0.002918	0.001989	0.006329	0.007727
706	-0.00212	0.000357	0.000768	0.002919	0.001923	0.006546	0.007957
705	-0.00213	0.000263	0.000841	0.003102	0.002077	0.006461	0.007699
704	-0.002	0.000313	0.000866	0.003137	0.002042	0.006594	0.007967
703	-0.00219	0.000346	0.000719	0.00305	0.002011	0.006623	0.007811
702	-0.00211	0.000339	0.000833	0.003148	0.002259	0.006662	0.007931
701	-0.00201	0.000387	0.000904	0.003272	0.002192	0.006831	0.008145
700	-0.00202	0.000468	0.001036	0.003181	0.00234	0.006868	0.008179
699	-0.00219	0.000407	0.001119	0.003277	0.00241	0.007023	0.008284
698	-0.00203	0.000484	0.00111	0.003287	0.002422	0.007073	0.00842
697	-0.00204	0.000432	0.001108	0.003272	0.00241	0.007028	0.008364
696	-0.00199	0.000635	0.001232	0.003516	0.002632	0.007485	0.008555
695	-0.00214	0.000364	0.001193	0.00341	0.002485	0.007176	0.008533
694	-0.00231	0.000357	0.001112	0.00331	0.002525	0.007283	0.008508
693	-0.00211	0.000602	0.001337	0.003608	0.002619	0.007448	0.008834
692	-0.00235	0.000526	0.001222	0.003494	0.002788	0.007389	0.00887
691	-0.00235	0.000677	0.001353	0.003577	0.002763	0.007715	0.008963
690	-0.00219	0.000616	0.001288	0.003471	0.002938	0.007686	0.009083
689	-0.00231	0.000828	0.001449	0.003679	0.002849	0.007781	0.009306
688	-0.00232	0.000757	0.001614	0.003788	0.003101	0.00807	0.009318
687	-0.00222	0.000732	0.001696	0.003795	0.00308	0.007956	0.009163
686	-0.00228	0.000705	0.001668	0.003652	0.003149	0.007995	0.00941
685	-0.00236	0.000701	0.001626	0.003971	0.003194	0.008175	0.009558
684	-0.00201	0.001131	0.002051	0.004122	0.003434	0.00851	0.009796
683	-0.00229	0.000865	0.001838	0.003948	0.003329	0.008524	0.009793
682	-0.00228	0.000846	0.001945	0.004006	0.003313	0.00842	0.009819
681	-0.00212	0.000955	0.002112	0.00414	0.003623	0.00884	0.010127
680	-0.00219	0.001084	0.002036	0.004095	0.003627	0.008941	0.010095
679	-0.00185	0.001265	0.00221	0.004432	0.003908	0.009009	0.010431
678	-0.00221	0.001168	0.002179	0.004469	0.003948	0.009076	0.010434
677	-0.00238	0.001092	0.002376	0.004112	0.003983	0.009208	0.010527
676	-0.00232	0.001175	0.002346	0.004289	0.003967	0.009344	0.010584
675	-0.00231	0.001356	0.00243	0.004351	0.004086	0.009439	0.010705
674	-0.00228	0.001336	0.002453	0.004538	0.0043	0.009578	0.010898
673	-0.00231	0.001286	0.002543	0.004644	0.004262	0.009664	0.010933
672	-0.00239	0.001479	0.002765	0.004642	0.0044	0.009931	0.01101
671	-0.00254	0.001107	0.002548	0.00458	0.00431	0.009784	0.011163
670	-0.00237	0.001635	0.002939	0.00492	0.004665	0.010193	0.011351
669	-0.00225	0.001494	0.002999	0.00493	0.004721	0.010382	0.011499
668	-0.00239	0.001631	0.003174	0.005128	0.004935	0.010686	0.011895
667	-0.00237	0.001694	0.003285	0.005072	0.004926	0.010703	0.011854

Wavelength (nm)	***Absorption intensity***						
	**concentration of α-methylbutyrylshikon (M)**						
	A	*B*	*C*	*D*	*E*	*F*	*G*
	0.00	$4.0x10^{-6}$	$8.0x10^{-6}$	$1.0x10^{-5}$	$1.4x10^{-5}$	$1.6x10^{-5}$	$1.8x10^{-5}$
666	-0.00238	0.001742	0.003324	0.005224	0.005115	0.010803	0.012083
665	-0.00225	0.001951	0.003584	0.005427	0.005436	0.011246	0.012469
664	-0.0022	0.002008	0.003455	0.005625	0.00549	0.011241	0.012565
663	-0.00218	0.002034	0.003684	0.005453	0.005597	0.011427	0.012648
662	-0.00231	0.001997	0.00371	0.005607	0.005683	0.011648	0.012745
661	-0.0022	0.002148	0.003854	0.005698	0.005938	0.011781	0.01315
660	-0.00218	0.002109	0.004056	0.005906	0.005943	0.011913	0.013226
659	-0.00234	0.002307	0.004014	0.005883	0.006144	0.012172	0.01329
658	-0.00221	0.002323	0.00417	0.00597	0.006242	0.012384	0.013574
657	-0.0026	0.002114	0.004152	0.005719	0.006088	0.012252	0.013439
656	-0.00233	0.002484	0.004479	0.006209	0.006457	0.012594	0.013739
655	-0.00222	0.00255	0.004661	0.006291	0.006676	0.012875	0.01422
654	-0.00248	0.002517	0.004631	0.006198	0.006838	0.012917	0.01419
653	-0.00222	0.002662	0.004809	0.006469	0.007032	0.013423	0.014522
652	-0.00234	0.002624	0.004883	0.006529	0.007026	0.013444	0.014771
651	-0.00244	0.002546	0.004997	0.006578	0.007115	0.013479	0.01474
650	-0.00231	0.002947	0.005036	0.006692	0.007425	0.013915	0.015036
649	-0.0023	0.002915	0.005223	0.006792	0.007494	0.014133	0.015286
648	-0.00216	0.003084	0.005389	0.006951	0.007857	0.01436	0.01554
647	-0.00221	0.003049	0.005387	0.006951	0.007913	0.014442	0.01568
646	-0.00234	0.003078	0.005552	0.007042	0.007914	0.014728	0.015883
645	-0.00234	0.003108	0.005604	0.007169	0.0081	0.014716	0.016064
644	-0.00225	0.003376	0.005902	0.007429	0.008335	0.01519	0.016263
643	-0.00225	0.003289	0.00611	0.007561	0.008483	0.015361	0.01648
642	-0.00244	0.0033	0.006106	0.007505	0.008409	0.015475	0.016493
641	-0.0023	0.003429	0.006204	0.007537	0.008622	0.01575	0.016791
640	-0.00228	0.003644	0.006363	0.007758	0.008869	0.015942	0.01709
639	-0.00237	0.003617	0.006401	0.007671	0.008914	0.016036	0.017252
638	-0.00214	0.003801	0.006632	0.008024	0.009243	0.016485	0.017658
637	-0.00243	0.003752	0.006635	0.007962	0.009108	0.016456	0.017659
636	-0.00236	0.003947	0.006752	0.007996	0.009438	0.016815	0.017925
635	-0.00237	0.003902	0.006827	0.00811	0.009419	0.01695	0.017792
634	-0.00231	0.003925	0.007012	0.00829	0.009715	0.01722	0.018207
633	-0.00243	0.003854	0.006883	0.00826	0.009708	0.017315	0.018357
632	-0.00248	0.003934	0.007143	0.008254	0.009906	0.017526	0.018571
631	-0.00243	0.003961	0.007247	0.008518	0.010083	0.017722	0.01881
630	-0.00237	0.004187	0.007505	0.008533	0.010224	0.017919	0.019112
629	-0.00255	0.004047	0.007447	0.008478	0.010328	0.018189	0.019047
628	-0.00257	0.004104	0.007445	0.008582	0.010362	0.018201	0.019281
627	-0.00242	0.004283	0.007607	0.008771	0.010681	0.018637	0.01966
626	-0.00246	0.004283	0.007701	0.008801	0.010719	0.018899	0.019777
625	-0.00235	0.004276	0.00797	0.008849	0.010834	0.018968	0.019936
624	-0.00226	0.004504	0.008057	0.009074	0.011045	0.019265	0.020197
623	-0.00247	0.004459	0.007926	0.009033	0.011019	0.019254	0.020273
622	-0.00229	0.00481	0.008418	0.009322	0.011612	0.019744	0.020809
621	-0.00238	0.004739	0.008347	0.009323	0.011525	0.019775	0.020852
620	-0.0025	0.00478	0.008477	0.0095	0.011716	0.020012	0.021006
619	-0.00242	0.005087	0.008835	0.009572	0.012005	0.020354	0.021396

EXCLI Journal 2020;19:48-70 – ISSN 1611-2156
Received: October 03, 2019, accepted: December 19, 2019, published: January 03, 2020

Wavelength (nm)	***Absorption intensity***						
	**concentration of α-methylbutyrylshikon (M)**						
	A	*B*	*C*	*D*	*E*	*F*	*G*
	0.00	$4.0x10^{-6}$	$8.0x10^{-6}$	$1.0x10^{-5}$	$1.4x10^{-5}$	$1.6x10^{-5}$	$1.8x10^{-5}$
618	-0.00247	0.004894	0.008807	0.009579	0.011931	0.020419	0.021551
617	-0.00234	0.004895	0.009065	0.009808	0.012234	0.020875	0.021752
616	-0.00235	0.005076	0.009094	0.009869	0.012321	0.021064	0.021998
615	-0.00242	0.005065	0.009292	0.009961	0.012468	0.021251	0.02228
614	-0.00249	0.005215	0.009243	0.010082	0.012651	0.021502	0.02245
613	-0.00237	0.005317	0.009607	0.010284	0.012893	0.021828	0.022739
612	-0.00231	0.005451	0.009612	0.010336	0.013084	0.02211	0.022957
611	-0.00243	0.005427	0.009736	0.010356	0.01317	0.022375	0.023297
610	-0.0023	0.005649	0.009863	0.010431	0.013325	0.022508	0.023516
609	-0.00241	0.005648	0.010022	0.010661	0.013647	0.022788	0.023661
608	-0.00241	0.005801	0.010236	0.010709	0.013911	0.023069	0.024175
607	-0.00227	0.005919	0.010453	0.01091	0.014176	0.023474	0.024338
606	-0.00234	0.006023	0.010444	0.011066	0.01433	0.023822	0.024719
605	-0.00234	0.006158	0.010769	0.011159	0.014439	0.024039	0.024824
604	-0.00228	0.006126	0.010797	0.011308	0.014582	0.024183	0.025076
603	-0.00236	0.006337	0.011041	0.011506	0.014869	0.024686	0.025498
602	-0.00231	0.006466	0.011109	0.011699	0.015062	0.02492	0.025536
601	-0.00233	0.006473	0.011354	0.011644	0.015339	0.025377	0.026015
600	-0.00241	0.006571	0.011527	0.011877	0.015454	0.025536	0.026138
599	-0.00237	0.00662	0.011544	0.011949	0.015592	0.025771	0.026474
598	-0.00232	0.006732	0.011812	0.012137	0.015904	0.026144	0.026875
597	-0.00234	0.006959	0.011921	0.012352	0.016147	0.026541	0.027146
596	-0.0023	0.006955	0.012163	0.012326	0.016359	0.026903	0.027576
595	-0.0024	0.007064	0.012258	0.012486	0.016545	0.027218	0.027838
594	-0.0023	0.007201	0.012433	0.012511	0.016897	0.027478	0.028215
593	-0.0024	0.007178	0.012433	0.012645	0.016924	0.027752	0.028391
592	-0.00236	0.007373	0.012582	0.012797	0.017261	0.028291	0.028798
591	-0.00222	0.007376	0.012979	0.013041	0.017586	0.028688	0.029343
590	-0.00229	0.007504	0.013215	0.013025	0.017835	0.02905	0.029665
589	-0.0025	0.007567	0.0134	0.013335	0.018041	0.029385	0.029964
588	-0.00237	0.007562	0.013474	0.013378	0.018266	0.029972	0.030402
587	-0.0024	0.007739	0.013829	0.013587	0.018624	0.030493	0.030861
586	-0.00252	0.007675	0.013834	0.013763	0.018889	0.030878	0.031301
585	-0.00234	0.008117	0.014226	0.014084	0.019379	0.031447	0.03177
584	-0.00246	0.00815	0.014292	0.014144	0.019528	0.031901	0.032236
583	-0.00244	0.008234	0.014606	0.014252	0.01982	0.032254	0.032492
582	-0.00256	0.008312	0.014743	0.0144	0.020313	0.032786	0.033167
581	-0.00252	0.008498	0.014942	0.014636	0.020467	0.033436	0.033472
580	-0.00262	0.008537	0.015169	0.014785	0.020755	0.033925	0.034091
579	-0.00243	0.008774	0.015454	0.015184	0.021402	0.034507	0.034609
578	-0.00245	0.00882	0.015786	0.015203	0.02158	0.03478	0.035155
577	-0.00237	0.009008	0.015933	0.015341	0.022009	0.035142	0.03527
576	-0.00246	0.009153	0.01609	0.015495	0.022249	0.035426	0.035606
575	-0.00235	0.009313	0.016564	0.015754	0.022613	0.036091	0.036078
574	-0.00258	0.009305	0.01637	0.015812	0.022825	0.03643	0.036305
573	-0.00225	0.00953	0.016884	0.016229	0.023079	0.036773	0.036786
572	-0.00243	0.009577	0.016898	0.016105	0.023186	0.03718	0.037217
571	-0.0025	0.009525	0.016871	0.016127	0.023458	0.037457	0.037203

Absorption intensity							
**Wavelength (nm)**	**concentration of α-methylbutyrylshikon (M)**						
	A	*B*	*C*	*D*	*E*	*F*	*G*
	0.00	$4.0x10^{-6}$	$8.0x10^{-6}$	$1.0x10^{-5}$	$1.4x10^{-5}$	$1.6x10^{-5}$	$1.8x10^{-5}$
570	-0.00236	0.009652	0.01725	0.016413	0.02358	0.037946	0.037824
569	-0.00251	0.009618	0.017243	0.01645	0.02346	0.038011	0.037962
568	-0.00248	0.009902	0.017552	0.016715	0.024289	0.038794	0.038644
567	-0.00236	0.009835	0.017528	0.01658	0.024221	0.038916	0.038722
566	-0.00226	0.01007	0.017664	0.016654	0.024351	0.0389	0.038912
565	-0.00243	0.009817	0.017632	0.016845	0.024434	0.039039	0.0388
564	-0.00253	0.009942	0.017577	0.016572	0.024476	0.039046	0.038929
563	-0.00249	0.009972	0.017796	0.01692	0.024529	0.039259	0.039175
562	-0.00231	0.010103	0.017899	0.016999	0.024671	0.039517	0.039312
561	-0.00245	0.010056	0.017961	0.017133	0.024716	0.039523	0.039436
560	-0.00242	0.010182	0.018073	0.017045	0.024742	0.039595	0.039669
559	-0.00282	0.009998	0.017706	0.016906	0.024635	0.039357	0.039329
558	-0.00251	0.010136	0.01805	0.017144	0.024818	0.039851	0.039727
557	-0.0025	0.010413	0.01825	0.017375	0.025178	0.040124	0.040003
556	-0.00213	0.010234	0.018252	0.017223	0.025291	0.040222	0.040038
555	-0.00209	0.0104	0.018396	0.017469	0.025487	0.040613	0.040415
554	-0.00245	0.010494	0.018445	0.017487	0.025453	0.040704	0.040483
553	-0.00236	0.010655	0.018634	0.017792	0.025741	0.041115	0.040744
552	-0.0024	0.01062	0.018647	0.017763	0.025821	0.041061	0.040952
551	-0.00246	0.010711	0.018868	0.017861	0.025937	0.041356	0.041282
550	-0.00247	0.01078	0.0191	0.017997	0.026288	0.041759	0.041336
549	-0.00242	0.010783	0.019147	0.018107	0.026413	0.041931	0.041596
548	-0.00251	0.011073	0.01927	0.018209	0.026534	0.04224	0.041875
547	-0.00243	0.010992	0.019367	0.018356	0.026732	0.042597	0.042205
546	-0.00239	0.011155	0.019532	0.018478	0.026915	0.042861	0.042524
545	-0.00225	0.011301	0.019627	0.018625	0.02714	0.043189	0.04279
544	-0.00234	0.01141	0.019728	0.018593	0.027357	0.043484	0.042984
543	-0.00228	0.011622	0.019853	0.018745	0.027648	0.043897	0.043514
542	-0.00237	0.011541	0.020058	0.018971	0.027698	0.044214	0.04371
541	-0.00242	0.011619	0.020294	0.019152	0.028	0.044614	0.044031
540	-0.00243	0.011601	0.020443	0.019185	0.02816	0.044926	0.044409
539	-0.00223	0.011949	0.020786	0.019497	0.028657	0.045389	0.044869
538	-0.00253	0.01178	0.020813	0.019401	0.028802	0.045684	0.045031
537	-0.00232	0.012017	0.021154	0.019921	0.029134	0.046075	0.045534
536	-0.00238	0.012169	0.021243	0.020008	0.029378	0.046652	0.045853
535	-0.00235	0.012128	0.02133	0.020092	0.029581	0.047014	0.046254
534	-0.0023	0.012253	0.021655	0.020173	0.029993	0.047397	0.046697
533	-0.00247	0.012221	0.021554	0.02027	0.030065	0.047595	0.046855
532	-0.00245	0.012318	0.02169	0.020396	0.030363	0.047999	0.047159
531	-0.00235	0.012606	0.021912	0.020567	0.03056	0.048174	0.047603
530	-0.00241	0.012458	0.021965	0.020475	0.030479	0.048514	0.047496
529	-0.00245	0.012637	0.022194	0.020742	0.030855	0.048877	0.047966
528	-0.00236	0.01267	0.02212	0.020683	0.030829	0.048881	0.048061
527	-0.00243	0.012727	0.022128	0.020812	0.031033	0.048985	0.048106
526	-0.00243	0.012843	0.022357	0.020937	0.031179	0.049119	0.048313
525	-0.00236	0.012828	0.022288	0.020899	0.031089	0.049111	0.0482
524	-0.00255	0.012727	0.022199	0.020733	0.031006	0.049027	0.048164
523	-0.00238	0.01288	0.022298	0.020889	0.031252	0.049131	0.048328

EXCLI Journal 2020;19:48-70 – ISSN 1611-2156
Received: October 03, 2019, accepted: December 19, 2019, published: January 03, 2020

Wavelength (nm)	***Absorption intensity***						
	**concentration of α-methylbutyrylshikon (M)**						
	A	*B*	*C*	*D*	*E*	*F*	*G*
	0.00	$4.0 x 10^{-6}$	$8.0 x 10^{-6}$	$1.0 x 10^{-5}$	$1.4 x 10^{-5}$	$1.6 x 10^{-5}$	$1.8 x 10^{-5}$
522	-0.00247	0.012814	0.022394	0.020833	0.030926	0.049014	0.048117
521	-0.00241	0.012763	0.022111	0.020935	0.031033	0.049173	0.048157
520	-0.00242	0.012677	0.022125	0.020915	0.030997	0.048985	0.048169
519	-0.00246	0.012579	0.022094	0.020795	0.030696	0.04885	0.047879
518	-0.00213	0.012766	0.022212	0.020986	0.030932	0.048845	0.047959
517	-0.00226	0.012431	0.021898	0.020703	0.030674	0.048458	0.047832
516	-0.00197	0.012593	0.021948	0.020831	0.030755	0.048498	0.047963
515	-0.00211	0.012674	0.021847	0.020673	0.030764	0.048538	0.04767
514	-0.00234	0.012714	0.021839	0.020775	0.030638	0.04837	0.047585
513	-0.00257	0.012622	0.021689	0.020564	0.030522	0.048108	0.047393
512	-0.00256	0.012525	0.021638	0.020545	0.030375	0.048135	0.047377
511	-0.00241	0.012307	0.021676	0.020676	0.030358	0.04811	0.047243
510	-0.00261	0.012344	0.021555	0.020511	0.030245	0.047905	0.046981
509	-0.00264	0.012426	0.021483	0.020565	0.030062	0.047748	0.047077
508	-0.00269	0.012279	0.021386	0.020305	0.029923	0.04769	0.046829
507	-0.00286	0.012159	0.021136	0.020289	0.029929	0.047476	0.046854
506	-0.00279	0.012024	0.021301	0.020383	0.029839	0.047534	0.046813
505	-0.00285	0.012166	0.02106	0.020108	0.029703	0.047451	0.046557
504	-0.00284	0.012065	0.021191	0.020314	0.029811	0.047319	0.046694
503	-0.00286	0.012147	0.021048	0.020292	0.029847	0.04754	0.046702
502	-0.00274	0.012141	0.021037	0.020252	0.029778	0.047336	0.046678
501	-0.00275	0.012111	0.020992	0.020358	0.029865	0.047291	0.046673
500	-0.00261	0.012281	0.021092	0.020482	0.029884	0.047376	0.046773
499	-0.00282	0.012007	0.021042	0.020275	0.029776	0.047181	0.04671
498	-0.00261	0.012229	0.02116	0.020369	0.029819	0.04736	0.046659
497	-0.0026	0.012197	0.021227	0.020408	0.029794	0.047318	0.046637
496	-0.00245	0.012368	0.021291	0.020474	0.029909	0.047308	0.046699
495	-0.00249	0.012354	0.021094	0.020499	0.029887	0.047322	0.046849
494	-0.00256	0.012198	0.021092	0.020413	0.029669	0.047222	0.046577
493	-0.00216	0.012311	0.021076	0.020661	0.029726	0.047211	0.046604
492	-0.0021	0.012275	0.020947	0.020481	0.029403	0.046903	0.046236
491	-0.00219	0.012003	0.020773	0.020281	0.029423	0.046811	0.046202
490	-0.00219	0.01207	0.020676	0.020337	0.029238	0.046522	0.045966
489	-0.0022	0.012132	0.020557	0.020343	0.029332	0.046468	0.045961
488	-0.00218	0.012037	0.020307	0.020192	0.028862	0.046044	0.045561
487	-0.00226	0.011977	0.020231	0.019946	0.028716	0.045658	0.045386
486	-0.00233	0.012001	0.020338	0.02013	0.028874	0.045673	0.04526
485	-0.00226	0.011909	0.020242	0.019922	0.028609	0.045301	0.045057
484	-0.00242	0.011771	0.019941	0.019791	0.028415	0.044872	0.044656
483	-0.00252	0.011584	0.019681	0.019572	0.028092	0.044721	0.044449
482	-0.00243	0.011776	0.019647	0.019639	0.027999	0.044526	0.044141
481	-0.00242	0.011615	0.01965	0.019523	0.027928	0.0442	0.043925
480	-0.00252	0.011512	0.019251	0.019365	0.027479	0.043643	0.043516
479	-0.00258	0.011414	0.019147	0.019408	0.027384	0.043399	0.043227
478	-0.00242	0.01135	0.018984	0.019041	0.027084	0.04309	0.043115
477	-0.00242	0.011048	0.018625	0.018922	0.026824	0.04276	0.042592
476	-0.00214	0.01119	0.018699	0.018903	0.026555	0.042601	0.04247
475	-0.002	0.011179	0.01858	0.018814	0.026438	0.042323	0.042339

Wavelength (nm)	***Absorption intensity***						
	**concentration of α-methylbutyrylshikon (M)**						
	A	*B*	*C*	*D*	*E*	*F*	*G*
	0.00	$4.0x10^{-6}$	$8.0x10^{-6}$	$1.0x10^{-5}$	$1.4x10^{-5}$	$1.6x10^{-5}$	$1.8x10^{-5}$
474	-0.00212	0.010921	0.018478	0.018752	0.026354	0.041982	0.041921
473	-0.00246	0.011022	0.018245	0.018542	0.026071	0.041531	0.041709
472	-0.00285	0.010727	0.017833	0.018269	0.025659	0.041078	0.041153
471	-0.00285	0.010384	0.017598	0.018107	0.025502	0.040681	0.040919
470	-0.00245	0.010949	0.017695	0.018321	0.025661	0.040863	0.041015
469	-0.00255	0.010723	0.017705	0.018138	0.025359	0.040475	0.040724
468	-0.00255	0.010614	0.017516	0.018129	0.025218	0.040371	0.040664
467	-0.00251	0.010572	0.017417	0.017946	0.025091	0.039951	0.040303
466	-0.00241	0.010599	0.017191	0.018038	0.024791	0.039755	0.040043
465	-0.00245	0.010595	0.017118	0.017971	0.024819	0.039571	0.040023
464	-0.00243	0.010482	0.017128	0.017879	0.02461	0.039208	0.039491
463	-0.00252	0.010213	0.016731	0.01778	0.024457	0.038696	0.039194
462	-0.00261	0.010331	0.016815	0.017688	0.024399	0.038519	0.038843
461	-0.00239	0.010057	0.016534	0.017597	0.023746	0.03813	0.038638
460	-0.00258	0.010261	0.016206	0.017333	0.02357	0.037955	0.038495
459	-0.00253	0.010463	0.016179	0.277549	0.023812	0.037993	0.038533
458	-0.00251	0.010043	0.016101	0.017298	0.023661	0.037724	0.038338
457	-0.00245	0.01005	0.016049	0.017073	0.023373	0.037401	0.038035
456	-0.00256	0.009891	0.01571	0.017148	0.023141	0.037133	0.037836
455	-0.00258	0.00972	0.01581	0.016996	0.023017	0.036848	0.037685
454	-0.00241	0.009809	0.015465	0.016868	0.02279	0.036494	0.037369
453	-0.00249	0.009718	0.015492	0.016723	0.02257	0.03639	0.037133
452	-0.00233	0.009736	0.015404	0.016715	0.02251	0.036051	0.03713
451	-0.00241	0.009468	0.015099	0.01668	0.02229	0.035734	0.03676
450	-0.00244	0.009607	0.015147	0.016541	0.022209	0.035607	0.036621
449	-0.00252	0.009315	0.014824	0.016484	0.021965	0.035339	0.036253
448	-0.00238	0.009521	0.014728	0.016334	0.021709	0.035115	0.036171
447	-0.00239	0.009496	0.014624	0.016257	0.021761	0.034823	0.036051
446	-0.00229	0.009281	0.014716	0.016343	0.021562	0.034831	0.035836
445	-0.00236	0.009074	0.014219	0.016036	0.021289	0.034214	0.03531
444	-0.00242	0.009286	0.014327	0.016197	0.021318	0.034218	0.035422
443	-0.00228	0.009138	0.014177	0.015848	0.021048	0.033808	0.035118
442	-0.00232	0.009337	0.014205	0.01599	0.021118	0.033695	0.035152
441	-0.0024	0.009133	0.013874	0.015737	0.020828	0.033501	0.034785
440	-0.00219	0.008982	0.013792	0.015833	0.020758	0.033355	0.034767
439	-0.00253	0.008902	0.013699	0.015742	0.020577	0.033015	0.034506
438	-0.0024	0.008871	0.013475	0.015742	0.020278	0.032843	0.034246
437	-0.00243	0.008768	0.013483	0.015622	0.020352	0.032716	0.034205
436	-0.00281	0.008577	0.013296	0.015287	0.020072	0.032292	0.033766
435	-0.0028	0.008667	0.013191	0.015277	0.019981	0.032182	0.03362
434	-0.00255	0.008689	0.013274	0.015448	0.019946	0.032017	0.033942
433	-0.00275	0.008511	0.012957	0.015077	0.019647	0.031806	0.033308
432	-0.00269	0.008608	0.012951	0.01523	0.019535	0.031619	0.03354
431	-0.00225	0.008965	0.013322	0.015522	0.019989	0.031718	0.033723
430	-0.00244	0.008642	0.012922	0.015246	0.019677	0.031497	0.033451
429	-0.00246	0.008821	0.012955	0.015242	0.019622	0.031324	0.033347
428	-0.00233	0.008733	0.012838	0.015213	0.019502	0.031336	0.033127
427	-0.00251	0.008476	0.012825	0.01508	0.019028	0.030976	0.03287

EXCLI Journal 2020;19:48-70 – ISSN 1611-2156
Received: October 03, 2019, accepted: December 19, 2019, published: January 03, 2020

Wavelength (nm)	Absorption intensity						
	concentration of α-methylbutyrylshikon (M)						
	A	*B*	*C*	*D*	*E*	*F*	*G*
	0.00	$4.0x10^{-6}$	$8.0x10^{-6}$	$1.0x10^{-5}$	$1.4x10^{-5}$	$1.6x10^{-5}$	$1.8x10^{-5}$
426	-0.00237	0.008659	0.012719	0.015152	0.019308	0.031026	0.033052
425	-0.00234	0.008637	0.0126	0.015365	0.019305	0.031042	0.032955
424	-0.00242	0.00835	0.012246	0.01479	0.019006	0.030512	0.032441
423	-0.00229	0.008515	0.012464	0.014839	0.018774	0.030482	0.032531
422	-0.00235	0.008472	0.012322	0.014985	0.018929	0.030222	0.032344
421	-0.00234	0.008524	0.012227	0.014817	0.018506	0.030015	0.032444
420	-0.00238	0.008371	0.011738	0.014658	0.018387	0.029697	0.031932
419	-0.00235	0.008158	0.011996	0.014574	0.018467	0.029555	0.031845
418	-0.00251	0.008053	0.011604	0.014463	0.018057	0.029315	0.031672
417	-0.00242	0.008083	0.011449	0.014373	0.01822	0.029183	0.03147
416	-0.00205	0.008215	0.011642	0.01446	0.018258	0.029266	0.031816
415	-0.00236	0.007818	0.011271	0.014135	0.017578	0.0288	0.031255
414	-0.00172	0.008059	0.011322	0.014203	0.017683	0.028946	0.031352
413	-0.00228	0.007773	0.011147	0.014059	0.017566	0.028529	0.031171
412	-0.00233	0.007635	0.010826	0.01364	0.017492	0.028254	0.030989
411	-0.00247	0.007634	0.010586	0.01378	0.017115	0.028059	0.030689
410	-0.00249	0.007302	0.010708	0.013646	0.017058	0.027798	0.030449
409	-0.00254	0.007521	0.010797	0.013847	0.017136	0.027908	0.030711
408	-0.0023	0.007621	0.010748	0.013946	0.017118	0.027931	0.03059
407	-0.00241	0.007671	0.010837	0.013881	0.01724	0.027656	0.030695
406	-0.00239	0.007408	0.010524	0.013808	0.0168	0.02768	0.030319
405	-0.00225	0.007449	0.010275	0.01364	0.016912	0.027091	0.029933
404	-0.00242	0.007437	0.010442	0.013725	0.016968	0.027144	0.030079
403	-0.00237	0.007859	0.010699	0.013711	0.016957	0.02747	0.030368
402	-0.00236	0.007669	0.010476	0.013801	0.01715	0.027193	0.030344
401	-0.00237	0.007264	0.010232	0.013588	0.016653	0.026886	0.030153
400	-0.0021	0.007589	0.010385	0.013925	0.016816	0.027306	0.030382
399	-0.00228	0.007245	0.010213	0.013817	0.016682	0.026895	0.029926
398	-0.00225	0.00727	0.010175	0.013754	0.016668	0.026842	0.030096
397	-0.00243	0.00727	0.010182	0.013526	0.016613	0.026595	0.029747
396	-0.00257	0.007288	0.009951	0.013626	0.016572	0.026637	0.030058
395	-0.00258	0.007001	0.009981	0.013217	0.016313	0.026402	0.029765
394	-0.00201	0.007372	0.010038	0.013576	0.01635	0.026548	0.029765
393	-0.00267	0.007218	0.009686	0.013568	0.016453	0.026326	0.029789
392	-0.00267	0.007043	0.00965	0.013212	0.016091	0.026301	0.02981
391	-0.00223	0.007503	0.010067	0.013935	0.016702	0.02656	0.030331
390	-0.00228	0.007599	0.010001	0.013906	0.016568	0.026903	0.030325
389	-0.00236	0.00761	0.010434	0.014056	0.016713	0.026301	0.030079
388	-0.00228	0.007464	0.010054	0.013829	0.016341	0.026538	0.030165
387	-0.00201	0.00723	0.009654	0.013653	0.016543	0.026327	0.029902
386	-0.00234	0.007086	0.009915	0.013727	0.016312	0.026303	0.030237
385	-0.00258	0.007174	0.009711	0.013487	0.016437	0.026436	0.029902
384	-0.00229	0.007197	0.009566	0.013657	0.016271	0.02643	0.030381
383	-0.00219	0.007335	0.009915	0.01368	0.016737	0.026458	0.030055
382	-0.00227	0.007251	0.009898	0.013786	0.016548	0.02658	0.030543
381	-0.002	0.007501	0.009852	0.013802	0.016659	0.026519	0.030952
380	-0.00218	0.007249	0.009825	0.013727	0.016473	0.026699	0.030518
379	-0.00251	0.007333	0.009813	0.013803	0.016693	0.026798	0.03028

EXCLI Journal 2020;19:48-70 – ISSN 1611-2156
Received: October 03, 2019, accepted: December 19, 2019, published: January 03, 2020

Wavelength (nm)	***Absorption intensity***						
	**concentration of α-methylbutyrylshikon (M)**						
	A	*B*	*C*	*D*	*E*	*F*	*G*
	0.00	$4.0x10^{-6}$	$8.0x10^{-6}$	$1.0x10^{-5}$	$1.4x10^{-5}$	$1.6x10^{-5}$	$1.8x10^{-5}$
378	-0.00182	0.007651	0.009893	0.014038	0.017011	0.026862	0.031006
377	-0.00227	0.007427	0.010238	0.013992	0.017237	0.027022	0.03118
376	-0.00333	0.006883	0.009512	0.013532	0.016404	0.026576	0.03085
375	-0.00205	0.008721	0.011205	0.015367	0.01848	0.027128	0.031544
374	-0.0023	0.007701	0.010351	0.014486	0.017322	0.02754	0.031293
373	-0.0025	0.007295	0.0096	0.013897	0.016752	0.026881	0.031214
372	-0.00142	0.008423	0.011042	0.015033	0.018274	0.028077	0.032065
371	-0.00163	0.00824	0.010735	0.014799	0.018002	0.027953	0.032077
370	-0.00198	0.007516	0.010756	0.014622	0.017886	0.027952	0.032158
369	-0.00169	0.007943	0.010689	0.014602	0.018214	0.02803	0.032351
368	-0.00179	0.008004	0.010816	0.014719	0.018093	0.028957	0.033166
367	-0.00208	0.008469	0.010854	0.014835	0.018202	0.028304	0.033305
366	-0.00163	0.008245	0.010901	0.014917	0.019058	0.029098	0.033479
365	-0.0016	0.008868	0.011849	0.01566	0.018693	0.02967	0.034288
364	-0.002	0.008743	0.01128	0.01553	0.019167	0.029133	0.033504
363	-0.00163	0.008436	0.011747	0.015417	0.019068	0.03019	0.034796
362	-0.00014	0.011066	0.012073	0.01598	0.019119	0.029875	0.034672
361	-0.00399	0.006514	0.010526	0.014058	0.017878	0.029174	0.033352
360	-0.00268	0.007823	0.013614	0.017232	0.021466	0.032095	0.036652
359	-0.00146	0.009111	0.012842	0.016595	0.020436	0.031023	0.035758
358	-0.00266	0.009151	0.012586	0.016404	0.02023	0.03095	0.034793
357	-0.00079	0.01003	0.013023	0.015971	0.020329	0.032421	0.035996
356	-0.00425	0.007613	0.010539	0.014795	0.018457	0.029796	0.035229
355	-0.00168	0.009195	0.015939	0.01921	0.023469	0.03497	0.039609
354	-0.00211	0.008748	0.012467	0.01632	0.020431	0.032299	0.037051
353	-0.00289	0.010206	0.011885	0.015749	0.020021	0.032101	0.037266
352	-0.00233	0.009668	0.009868	0.01373	0.019192	0.030787	0.034737
351	0.000134	0.011631	0.016089	0.020373	0.025426	0.036948	0.042327
350	-0.00101	0.011233	0.015195	0.019774	0.022431	0.035057	0.039379
349	-0.00278	0.009211	0.014417	0.016904	0.021089	0.034587	0.039493
348	-0.00519	0.005852	0.010904	0.016021	0.019123	0.031951	0.037703
347	-0.00558	0.006055	0.011429	0.016198	0.019774	0.031582	0.038428
346	-0.00481	0.007031	0.012627	0.017475	0.021069	0.032882	0.039738
345	-0.00511	0.007065	0.012053	0.017014	0.020508	0.033232	0.039205
344	-0.00459	0.007321	0.012194	0.017233	0.021415	0.03403	0.040532
343	-0.00529	0.006918	0.012665	0.016849	0.021207	0.034112	0.040534
342	-0.0052	0.007562	0.012764	0.017521	0.021886	0.035311	0.041572
341	-0.00539	0.006899	0.01307	0.018223	0.022067	0.035279	0.042059
340	-0.00575	0.006615	0.013095	0.017039	0.021763	0.035113	0.041701
339	-0.0051	0.007708	0.0134	0.018744	0.023015	0.036044	0.042905
338	-0.00434	0.00827	0.014587	0.018992	0.023912	0.037712	0.04426
337	-0.00483	0.008035	0.014016	0.01939	0.023744	0.037792	0.044569
336	-0.00473	0.008331	0.014871	0.019354	0.024435	0.038547	0.044841
335	-0.00492	0.008123	0.01481	0.019343	0.025112	0.038816	0.045116
334	-0.00453	0.009123	0.01587	0.020711	0.025408	0.039699	0.046657
333	-0.00419	0.009254	0.016388	0.020731	0.026243	0.040573	0.047325
332	-0.00456	0.008933	0.016975	0.020748	0.026607	0.041065	0.047604
331	-0.00458	0.009831	0.016399	0.020969	0.027002	0.041668	0.04828

EXCLI Journal 2020;19:48-70 – ISSN 1611-2156
Received: October 03, 2019, accepted: December 19, 2019, published: January 03, 2020

Wavelength (nm)	Absorption intensity						
	concentration of α-methylbutyrylshikon (M)						
	A	*B*	*C*	*D*	*E*	*F*	*G*
	0.00	4.0x10^{-6}	8.0x10^{-6}	1.0x10^{-5}	1.4x10^{-5}	1.6x10^{-5}	1.8x10^{-5}
330	-0.00448	0.009103	0.016916	0.021276	0.026716	0.042238	0.048769
329	-0.00428	0.010262	0.017479	0.021859	0.027988	0.043557	0.05003
328	-0.00393	0.010364	0.018472	0.022206	0.028691	0.04455	0.051262
327	-0.00413	0.010563	0.018143	0.022442	0.028853	0.044505	0.051657
326	-0.00444	0.009388	0.018701	0.022199	0.029291	0.045102	0.051956
325	-0.00359	0.011161	0.019049	0.023546	0.030711	0.046446	0.053327
324	-0.00428	0.011152	0.018949	0.023602	0.03069	0.047293	0.053974
323	-0.00387	0.011469	0.019753	0.023858	0.031326	0.048232	0.054549
322	-0.00326	0.011741	0.020765	0.02422	0.031815	0.04929	0.056004
321	-0.00381	0.01111	0.020761	0.024192	0.032404	0.049663	0.056437
320	-0.00311	0.012272	0.021741	0.025243	0.033584	0.050864	0.057388
319	-0.00306	0.012193	0.021754	0.025635	0.033851	0.051818	0.058427
318	-0.00304	0.013039	0.022713	0.026141	0.034548	0.052941	0.059298
317	-0.003	0.012923	0.022699	0.025788	0.034934	0.053041	0.059747
316	-0.00355	0.012875	0.022978	0.026108	0.035325	0.054069	0.060375
315	-0.00263	0.013805	0.023855	0.026837	0.036545	0.054984	0.061875
314	-0.00221	0.014453	0.024887	0.027749	0.03758	0.056312	0.063204
313	-0.00276	0.014265	0.024425	0.028247	0.037968	0.057512	0.063678
312	-0.00237	0.015034	0.025256	0.02845	0.038001	0.058161	0.064777
311	-0.00205	0.014852	0.025651	0.02912	0.038941	0.059337	0.065883
310	-0.00228	0.014753	0.026335	0.029599	0.039761	0.060084	0.066343
309	-0.00206	0.015275	0.02681	0.029592	0.040339	0.061547	0.067807
308	-0.00202	0.015678	0.027445	0.030532	0.040872	0.062635	0.068864
307	-0.00186	0.015972	0.028189	0.030815	0.041958	0.063491	0.069731
306	-0.00211	0.01665	0.028553	0.031349	0.04249	0.06453	0.071146
305	-0.00161	0.016487	0.028672	0.032175	0.04327	0.066161	0.072057
304	-0.00087	0.01732	0.030075	0.033073	0.044935	0.067563	0.073705
303	-0.00059	0.017735	0.030778	0.033973	0.046003	0.069232	0.075555
302	0.000218	0.018751	0.03173	0.034781	0.047797	0.070813	0.077118
301	-3.10E-05	0.019258	0.032877	0.036281	0.048721	0.071592	0.078248
300	0.000913	0.020207	0.034265	0.03713	0.050407	0.074325	0.08029
299	0.001784	0.020444	0.035475	0.038142	0.051702	0.076594	0.082628
298	0.002942	0.021718	0.037363	0.040582	0.054156	0.078623	0.085091
297	0.004113	0.023121	0.039171	0.042478	0.056332	0.08136	0.087642
296	0.00545	0.024457	0.041553	0.044647	0.058591	0.084088	0.090084
295	0.00727	0.026653	0.044098	0.047254	0.061645	0.087214	0.093091
294	0.00925	0.028218	0.046628	0.050042	0.065059	0.090563	0.096945
293	0.01117	0.030363	0.049969	0.053348	0.068248	0.094482	0.100638
292	0.013862	0.032193	0.053597	0.056639	0.072335	0.097979	0.10462
291	0.016338	0.034838	0.05697	0.060481	0.076211	0.102323	0.108649
290	0.018448	0.037149	0.060418	0.06381	0.080011	0.106836	0.112659
289	0.021878	0.039724	0.064847	0.068081	0.084956	0.111189	0.117506
288	0.024238	0.041754	0.068336	0.07179	0.088529	0.114613	0.121148
287	0.027943	0.045002	0.072688	0.076467	0.093296	0.119991	0.126109
286	0.030726	0.047512	0.07724	0.080432	0.098108	0.124313	0.130801
285	0.034348	0.050617	0.081557	0.085443	0.102884	0.129408	0.135988
284	0.036704	0.053178	0.085501	0.089602	0.107472	0.133727	0.14068
283	0.040967	0.056215	0.090612	0.094644	0.112468	0.138929	0.145495

EXCLI Journal 2020;19:48-70 – ISSN 1611-2156
Received: October 03, 2019, accepted: December 19, 2019, published: January 03, 2020

Wavelength (nm)	***Absorption intensity***						
	**concentration of α-methylbutyrylshikon (M)**						
	A	*B*	*C*	*D*	*E*	*F*	*G*
	0.00	$4.0x10^{-6}$	$8.0x10^{-6}$	$1.0x10^{-5}$	$1.4x10^{-5}$	$1.6x10^{-5}$	$1.8x10^{-5}$
282	0.044302	0.058887	0.09519	0.09912	0.117563	0.143528	0.150413
281	0.047006	0.061471	0.099316	0.103524	0.122207	0.147994	0.155059
280	0.049764	0.064297	0.103052	0.107393	0.126496	0.15261	0.159213
279	0.053461	0.066931	0.107304	0.112157	0.131443	0.15704	0.163848
278	0.055833	0.069549	0.111138	0.115544	0.135531	0.161376	0.168329
277	0.058592	0.071978	0.115236	0.119612	0.139295	0.16549	0.172687
276	0.061392	0.074708	0.118891	0.123991	0.143905	0.170445	0.177079
275	0.064487	0.077162	0.123329	0.128236	0.148299	0.174836	0.181919
274	0.067152	0.07969	0.127126	0.13196	0.152633	0.179139	0.186363
273	0.070379	0.082395	0.131007	0.136041	0.156802	0.183431	0.190632
272	0.072816	0.084623	0.134883	0.13975	0.161165	0.187623	0.19472
271	0.075495	0.086737	0.138628	0.143807	0.164684	0.19184	0.199199
270	0.077832	0.089374	0.141798	0.147191	0.168918	0.195535	0.202568
269	0.080519	0.092157	0.145575	0.150812	0.172301	0.199451	0.206402
268	0.082937	0.093546	0.148286	0.153884	0.175843	0.202859	0.209778
267	0.08531	0.095215	0.151323	0.157086	0.179141	0.20574	0.213148
266	0.086812	0.096902	0.154363	0.159846	0.181839	0.208643	0.216087
265	0.089228	0.09925	0.156876	0.162666	0.184817	0.211494	0.219082
264	0.090958	0.100306	0.158997	0.165078	0.187516	0.213925	0.221359
263	0.092668	0.10205	0.161658	0.167816	0.190132	0.216444	0.224026
262	0.094166	0.102886	0.16367	0.169747	0.19202	0.218582	0.226084
261	0.09525	0.104033	0.164816	0.171281	0.193801	0.219785	0.22798
260	0.096505	0.105115	0.166662	0.173232	0.195485	0.221464	0.22959
259	0.097337	0.105599	0.167303	0.173841	0.19618	0.22197	0.230778
258	0.097545	0.105546	0.167318	0.174085	0.196278	0.222443	0.23114
257	0.097359	0.105773	0.167835	0.174189	0.196691	0.222369	0.231038
256	0.097161	0.105871	0.167519	0.174074	0.196125	0.222219	0.23083
255	0.096429	0.105379	0.166313	0.173695	0.195578	0.221185	0.23045
254	0.095511	0.104603	0.165922	0.17237	0.194805	0.220385	0.229544
253	0.093743	0.103453	0.16411	0.170907	0.193218	0.219335	0.228373
252	0.092228	0.102586	0.161757	0.168692	0.191437	0.21755	0.226832
251	0.090784	0.101194	0.159771	0.166861	0.189512	0.215661	0.225238
250	0.088173	0.099114	0.156771	0.163749	0.186436	0.213256	0.222792
249	0.085887	0.097795	0.153881	0.160904	0.183578	0.211009	0.220447
248	0.083333	0.095118	0.150943	0.157475	0.180512	0.20787	0.217626
247	0.080622	0.09325	0.147625	0.154478	0.177077	0.205036	0.215049
246	0.077512	0.091145	0.144166	0.150718	0.173628	0.201779	0.212085
245	0.074406	0.088608	0.140375	0.147067	0.170329	0.198899	0.208932
244	0.071717	0.086703	0.137453	0.143742	0.167381	0.196468	0.206548
243	0.068921	0.084714	0.134132	0.140461	0.164329	0.193854	0.204323
242	0.065782	0.082507	0.131235	0.137098	0.161479	0.191742	0.202192
241	0.063346	0.081271	0.128842	0.134655	0.159144	0.190155	0.200899
240	0.059981	0.079468	0.126302	0.132167	0.156998	0.189077	0.199587
239	0.057943	0.078901	0.125338	0.130001	0.156168	0.189771	0.19983
238	0.055584	0.078345	0.124789	0.128972	0.156324	0.191315	0.201553
237	0.053574	0.078526	0.125245	0.128764	0.157919	0.19458	0.204321
236	0.051933	0.079932	0.127422	0.130379	0.160756	0.20111	0.209953
235	0.05052	0.08178	0.131378	0.133022	0.166593	0.210101	0.217795

EXCLI Journal 2020;19:48-70 – ISSN 1611-2156
Received: October 03, 2019, accepted: December 19, 2019, published: January 03, 2020

Wavelength (nm)	***Absorption intensity***						
	**concentration of α-methylbutyrylshikon (M)**						
	A	*B*	*C*	*D*	*E*	*F*	*G*
	0.00	$4.0x10^{-6}$	$8.0x10^{-6}$	$1.0x10^{-5}$	$1.4x10^{-5}$	$1.6x10^{-5}$	$1.8x10^{-5}$
234	0.049093	0.085831	0.137866	0.138144	0.17494	0.224021	0.230013
233	0.048102	0.091917	0.148346	0.146019	0.18752	0.242767	0.246867
232	0.047873	0.101059	0.162544	0.157106	0.205569	0.269896	0.271315
231	0.047391	0.112374	0.182826	0.173211	0.230005	0.305708	0.303449
230	0.047858	0.129471	0.210299	0.195516	0.263779	0.354383	0.347724
229	0.048451	0.151887	0.247304	0.225164	0.308349	0.418541	0.406213
228	0.049407	0.181384	0.296492	0.264481	0.367653	0.503709	0.48333
227	0.050614	0.220189	0.359711	0.31581	0.444278	0.612356	0.58223
226	0.052243	0.270533	0.441531	0.382622	0.543517	0.752888	0.710497
225	0.054389	0.333824	0.544349	0.465735	0.666874	0.927472	0.86976
224	0.057025	0.414813	0.674583	0.572184	0.823877	1.147618	1.071198
223	0.060047	0.513903	0.834576	0.702241	1.015465	1.414294	1.316271
222	0.062965	0.634947	1.029849	0.861108	1.249403	1.739009	1.615285
221	0.067167	0.780981	1.261601	1.051683	1.526297	2.121956	1.964827
220	0.071357	0.952662	1.535843	1.275128	1.851383	2.559948	2.374148
219	0.076636	1.149022	1.848439	1.5319	2.221077	3.051076	2.833935
218	0.081748	1.37464	2.203461	1.824722	2.639034	3.53274	3.312869
217	0.08672	1.621182	2.592281	2.143907	3.078754	3.979295	3.787259
216	0.092059	1.891909	2.995246	2.493423	3.515566	4.254156	4.14602
215	0.097179	2.175622	3.415645	2.851583	3.931816	4.458649	4.39852
214	0.103344	2.467276	3.767301	3.214597	4.261034	4.744187	4.77533
213	0.109023	2.74922	4.10081	3.55905	4.43396	4.745598	4.703088
212	0.11476	3.025046	4.416883	3.89079	4.710759	4.830727	4.952612
211	0.12031	3.261539	4.529682	4.21757	4.963376	5.324794	5.743496
210	0.125045	3.481599	4.621264	4.359833	4.866578	5.045238	5.032066
209	0.131481	3.637647	4.635894	4.450986	4.772308	4.876023	4.875486
208	0.137277	3.714113	4.607881	4.496354	4.815233	5.033036	4.888535
207	0.142533	3.75905	4.706416	4.426709	4.729309	4.894142	5.133417
206	0.148924	3.785439	4.684474	4.605399	4.670239	4.897457	4.865519
205	0.157314	3.758472	4.730134	4.78703	5.188178	5.07448	5.366031
204	0.160151	3.695644	4.312085	4.344639	4.516486	4.537709	4.530661
203	0.167487	3.544953	4.235178	4.15501	4.272343	4.320246	4.460322
202	0.175696	3.402086	3.902531	3.957329	4.046256	4.112654	4.062261
201	0.172119	3.169246	3.471914	3.417238	3.452718	3.558007	3.561023
200	0.169385	2.682266	2.790239	3.000286	2.905946	2.933525	3.001377

EXCLI Journal 2020;19:48-70 – ISSN 1611-2156
Received: October 03, 2019, accepted: December 19, 2019, published: January 03, 2020

Table 2: Absorption intensity in the wavelength range of 200-800 nm for absorption spectra of CT-DNA fixed concentration (1.77 x 10^{-5} M), in the absence (*A*) and presence of increasing concentration of acetylshikonin (*B*-*G*)

Wavelength (nm)	***Absorption intensity***						
	**concentration of acetylshikonin (M)**						
	A	*B*	*C*	*D*	*E*	*F*	*G*
	0.00	$4.0x10^{-6}$	$8.0x10^{-6}$	$1.0x10^{-5}$	$1.4x10^{-5}$	$1.6x10^{-5}$	$1.8x10^{-5}$
800	-0.00126	0.000889	0.002986	0.007536	0.010938	0.013996	0.015181
799	-0.00161	0.000695	0.002998	0.007278	0.010552	0.013868	0.015042
798	-0.00137	0.000881	0.003218	0.007982	0.010684	0.014002	0.015552
797	-0.00101	0.000898	0.003209	0.007749	0.010957	0.014304	0.015361
796	-0.0016	0.00083	0.002864	0.00729	0.010682	0.014064	0.014961
795	-0.00128	0.000954	0.00299	0.007783	0.01077	0.014465	0.015412
794	-0.00152	0.000927	0.002941	0.007638	0.010832	0.014191	0.0153
793	-0.00175	0.000847	0.002934	0.007307	0.010651	0.014012	0.01518
792	-0.00123	0.000808	0.003132	0.007553	0.010683	0.014057	0.015278
791	-0.00134	0.000687	0.003005	0.007395	0.010559	0.014158	0.015312
790	-0.00126	0.001014	0.003296	0.007552	0.010781	0.01451	0.015523
789	-0.00108	0.001039	0.003052	0.007754	0.010829	0.014258	0.015547
788	-0.00154	0.000855	0.002907	0.007461	0.010524	0.014244	0.015287
787	-0.00114	0.000691	0.003061	0.007612	0.010507	0.014507	0.015523
786	-0.00131	0.000438	0.003023	0.007658	0.010793	0.014342	0.015734
785	-0.00159	0.000686	0.003349	0.007871	0.01061	0.014361	0.015438
784	-0.00128	0.000916	0.003034	0.008083	0.010836	0.014508	0.015775
783	-0.0013	0.000884	0.003077	0.007847	0.010943	0.014372	0.015605
782	-0.00152	0.000833	0.00302	0.0077	0.01053	0.014465	0.015359
781	-0.00156	0.000883	0.002951	0.007631	0.010821	0.014392	0.015384
780	-0.00155	0.000784	0.003146	0.007825	0.011131	0.014633	0.015666
779	-0.00142	0.00088	0.003106	0.007668	0.011035	0.014798	0.015537
778	-0.00106	0.000869	0.003143	0.007954	0.010964	0.014874	0.015669
777	-0.00144	0.001159	0.002926	0.007978	0.010926	0.01455	0.015594
776	-0.0014	0.000826	0.003177	0.007984	0.010892	0.014825	0.015859
775	-0.00153	0.000941	0.00311	0.007901	0.011025	0.014567	0.015738
774	-0.00151	0.000893	0.003082	0.008007	0.010884	0.014906	0.015658
773	-0.00124	0.000916	0.003266	0.00801	0.011011	0.014753	0.015828
772	-0.00139	0.000911	0.003225	0.008156	0.011113	0.014734	0.015799
771	-0.0013	0.001013	0.003259	0.007913	0.011156	0.015127	0.015796
770	-0.00128	0.000794	0.003131	0.007994	0.011304	0.014832	0.015887
769	-0.00161	0.000853	0.003236	0.008109	0.01103	0.014944	0.015817
768	-0.00131	0.000799	0.003283	0.007985	0.011051	0.015049	0.016147
767	-0.00159	0.00099	0.003104	0.0081	0.011321	0.015125	0.016066
766	-0.00133	0.001032	0.00337	0.008236	0.011428	0.014993	0.015852
765	-0.00156	0.000644	0.00331	0.008023	0.011204	0.014622	0.015911
764	-0.00134	0.001036	0.003324	0.008117	0.011372	0.015224	0.016007
763	-0.00141	0.000747	0.003052	0.008074	0.01111	0.01503	0.015861
762	-0.00111	0.00104	0.003195	0.008209	0.011498	0.015288	0.016271
761	-0.00147	0.000981	0.003047	0.008179	0.011281	0.015176	0.016059
760	-0.00121	0.001007	0.003291	0.008312	0.011464	0.015303	0.016374
759	-0.00132	0.001078	0.00312	0.008274	0.011278	0.014934	0.015993
758	-0.00143	0.000765	0.002934	0.008147	0.011005	0.014993	0.015896
757	-0.00146	0.000987	0.003248	0.008336	0.01133	0.015177	0.016092

Wavelength (nm)	Absorption intensity						
	concentration of acetylshikonin (M)						
	A	*B*	*C*	*D*	*E*	*F*	*G*
	0.00	$4.0x10^{-6}$	$8.0x10^{-6}$	$1.0x10^{-5}$	$1.4x10^{-5}$	$1.6x10^{-5}$	$1.8x10^{-5}$
756	-0.00147	0.000938	0.003196	0.008225	0.011284	0.01519	0.016432
755	-0.00153	0.001003	0.003284	0.008163	0.011392	0.015319	0.016117
754	-0.00159	0.000884	0.00342	0.008507	0.011305	0.015349	0.016442
753	-0.00156	0.00101	0.003379	0.008373	0.011393	0.015369	0.016188
752	-0.0015	0.00104	0.003368	0.008411	0.011486	0.015514	0.016488
751	-0.00124	0.000957	0.00357	0.008787	0.011698	0.015774	0.016505
750	-0.00148	0.000893	0.003056	0.008488	0.011456	0.015447	0.016294
749	-0.00141	0.000971	0.003317	0.008321	0.011515	0.015408	0.016398
748	-0.00142	0.000789	0.003394	0.008563	0.011525	0.015457	0.016436
747	-0.00147	0.00114	0.003183	0.008426	0.011604	0.015426	0.01642
746	-0.00148	0.000783	0.00326	0.008397	0.011284	0.015477	0.01634
745	-0.0014	0.001136	0.003577	0.008638	0.011815	0.015936	0.016613
744	-0.00138	0.000896	0.003357	0.008487	0.011585	0.015746	0.016598
743	-0.0014	0.001009	0.003288	0.008719	0.011652	0.015733	0.01648
742	-0.00126	0.000923	0.003543	0.008619	0.011474	0.015776	0.016655
741	-0.00152	0.000835	0.003291	0.008456	0.01159	0.015643	0.016551
740	-0.00154	0.00103	0.003453	0.00867	0.011639	0.015984	0.016647
739	-0.0011	0.000954	0.003647	0.008679	0.011868	0.015985	0.016789
738	-0.0015	0.001044	0.003471	0.008751	0.011681	0.015904	0.016597
737	-0.00147	0.000907	0.003484	0.00861	0.011744	0.015832	0.016677
736	-0.00151	0.001049	0.00374	0.008784	0.011918	0.016059	0.016803
735	-0.00149	0.000951	0.003615	0.008942	0.011996	0.016081	0.01682
734	-0.00166	0.000955	0.003509	0.008616	0.011696	0.015904	0.016709
733	-0.00129	0.001167	0.003847	0.008939	0.011867	0.016288	0.017002
732	-0.00153	0.001007	0.003662	0.008955	0.011923	0.016209	0.016872
731	-0.00147	0.000999	0.003598	0.008794	0.011837	0.016186	0.01684
730	-0.00132	0.000904	0.003716	0.008982	0.011891	0.016313	0.017117
729	-0.00135	0.000929	0.003712	0.00905	0.011887	0.01611	0.017198
728	-0.00131	0.001202	0.00379	0.009203	0.01222	0.016485	0.017309
727	-0.0014	0.000978	0.003814	0.009037	0.011945	0.016373	0.017127
726	-0.00147	0.000947	0.003861	0.009102	0.012051	0.016493	0.017089
725	-0.0014	0.000994	0.003902	0.009059	0.012124	0.016502	0.017187
724	-0.00122	0.001114	0.004041	0.009168	0.012148	0.016591	0.017444
723	-0.00148	0.000929	0.003942	0.009121	0.011936	0.016511	0.017226
722	-0.00148	0.000947	0.003815	0.008943	0.011988	0.01658	0.017229
721	-0.00137	0.000914	0.004137	0.009305	0.012186	0.016836	0.017499
720	-0.00117	0.001007	0.004136	0.009446	0.01231	0.016737	0.017519
719	-0.00142	0.00099	0.003841	0.009226	0.012234	0.016695	0.0175
718	-0.00127	0.001034	0.004186	0.009455	0.012207	0.016893	0.017611
717	-0.00129	0.001068	0.003943	0.009342	0.01219	0.016983	0.017566
716	-0.00124	0.001103	0.004301	0.009512	0.012474	0.017127	0.017724
715	-0.00129	0.001133	0.004375	0.00956	0.012486	0.017289	0.017691
714	-0.00129	0.001091	0.00438	0.009531	0.012421	0.017222	0.017798
713	-0.00121	0.001151	0.004274	0.0097	0.012586	0.017098	0.017871
712	-0.00124	0.001262	0.004358	0.009695	0.01246	0.017347	0.017827
711	-0.00117	0.001116	0.004421	0.009733	0.012444	0.01749	0.018023
710	-0.00128	0.001137	0.004447	0.009802	0.012521	0.017436	0.018059
709	-0.00133	0.001038	0.004222	0.009674	0.012357	0.017447	0.018001

EXCLI Journal 2020;19:48-70 – ISSN 1611-2156
Received: October 03, 2019, accepted: December 19, 2019, published: January 03, 2020

Wavelength (nm)	***Absorption intensity***						
	**concentration of acetylshikonin (M)**						
	A	*B*	*C*	*D*	*E*	*F*	*G*
	0.00	$4.0x10^{-6}$	$8.0x10^{-6}$	$1.0x10^{-5}$	$1.4x10^{-5}$	$1.6x10^{-5}$	$1.8x10^{-5}$
708	-0.00112	0.001315	0.004367	0.009784	0.01261	0.017527	0.018159
707	-0.00134	0.001229	0.004448	0.00987	0.012718	0.017628	0.018174
706	-0.00116	0.001203	0.004505	0.009969	0.012703	0.017726	0.018218
705	-0.00123	0.00121	0.004571	0.009996	0.012702	0.017667	0.018215
704	-0.00138	0.001168	0.004506	0.009921	0.012748	0.017664	0.018219
703	-0.00126	0.001104	0.004557	0.009964	0.01267	0.017612	0.018354
702	-0.00126	0.001119	0.004413	0.01004	0.012987	0.017739	0.018506
701	-0.00126	0.001382	0.004647	0.010029	0.012956	0.017844	0.018374
700	-0.00113	0.001132	0.004528	0.009889	0.012943	0.018016	0.018527
699	-0.00114	0.001225	0.004603	0.010151	0.012913	0.018089	0.018675
698	-0.00112	0.001356	0.004615	0.01022	0.01307	0.018234	0.018767
697	-0.00122	0.001255	0.004656	0.010179	0.013117	0.018312	0.018672
696	-0.00107	0.001415	0.00486	0.010294	0.013095	0.018361	0.018904
695	-0.00124	0.001218	0.004674	0.010213	0.013094	0.018182	0.018766
694	-0.00159	0.001203	0.004527	0.010171	0.013022	0.018158	0.018746
693	-0.00122	0.001457	0.0049	0.010441	0.013359	0.018521	0.019002
692	-0.00123	0.001165	0.004699	0.010408	0.013178	0.018423	0.018941
691	-0.00124	0.001193	0.004804	0.010396	0.013124	0.018539	0.019134
690	-0.00118	0.001325	0.004811	0.010423	0.013518	0.018504	0.019089
689	-0.00123	0.001339	0.004891	0.010608	0.013557	0.018623	0.019264
688	-0.00124	0.001569	0.004962	0.010647	0.013543	0.01869	0.019185
687	-0.00125	0.001303	0.004976	0.010479	0.013619	0.018653	0.019313
686	-0.00123	0.00135	0.00505	0.010701	0.013499	0.018738	0.019483
685	-0.00122	0.001365	0.005002	0.010503	0.013843	0.018982	0.019517
684	-0.00108	0.001707	0.00539	0.010879	0.013986	0.019266	0.019654
683	-0.00126	0.001439	0.005019	0.010776	0.013811	0.019166	0.019598
682	-0.00131	0.001453	0.004935	0.010655	0.013818	0.019155	0.019684
681	-0.00104	0.001612	0.00527	0.01098	0.014042	0.019226	0.019865
680	-0.00126	0.00161	0.005206	0.010953	0.014051	0.019297	0.019965
679	-0.00101	0.001664	0.005478	0.011141	0.014136	0.01954	0.020158
678	-0.00116	0.001667	0.005324	0.011263	0.014162	0.019531	0.020128
677	-0.00122	0.001574	0.005443	0.01106	0.01427	0.019499	0.020087
676	-0.00116	0.001642	0.005399	0.011178	0.014355	0.01965	0.020112
675	-0.00125	0.001643	0.005595	0.011231	0.014348	0.019665	0.020194
674	-0.00131	0.001617	0.005541	0.011259	0.01446	0.019759	0.020266
673	-0.00121	0.001498	0.00564	0.011329	0.014529	0.019901	0.020593
672	-0.00113	0.001576	0.005618	0.011336	0.014593	0.020098	0.020627
671	-0.00146	0.001351	0.005597	0.011392	0.014448	0.019969	0.02057
670	-0.00105	0.001697	0.005775	0.011498	0.014827	0.020229	0.020968
669	-0.00127	0.001774	0.00578	0.011605	0.014814	0.020328	0.020964
668	-0.001	0.001801	0.006017	0.011887	0.015048	0.02058	0.021149
667	-0.00091	0.001832	0.005898	0.011988	0.015016	0.020649	0.021387
666	-0.00115	0.001819	0.00605	0.011987	0.015137	0.020789	0.021399
665	-0.00099	0.002042	0.006124	0.012407	0.015303	0.020911	0.021621
664	-0.00096	0.001901	0.006231	0.0122	0.015477	0.021126	0.021757
663	-0.00101	0.001987	0.006346	0.012389	0.015444	0.021102	0.021817
662	-0.00097	0.002089	0.006351	0.012534	0.015642	0.021371	0.021955
661	-0.00118	0.002111	0.006574	0.012579	0.015732	0.021401	0.022042

EXCLI Journal 2020;19:48-70 – ISSN 1611-2156
Received: October 03, 2019, accepted: December 19, 2019, published: January 03, 2020

Wavelength (nm)	***Absorption intensity***						
	**concentration of acetylshikonin (M)**						
	A	*B*	*C*	*D*	*E*	*F*	*G*
	0.00	$4.0x10^{-6}$	$8.0x10^{-6}$	$1.0x10^{-5}$	$1.4x10^{-5}$	$1.6x10^{-5}$	$1.8x10^{-5}$
660	-0.0009	0.002016	0.006518	0.012652	0.015894	0.021561	0.022216
659	-0.00092	0.002132	0.006619	0.012869	0.01594	0.021726	0.022535
658	-0.00098	0.002136	0.006681	0.012913	0.016149	0.021982	0.022712
657	-0.00125	0.001992	0.00642	0.012839	0.015817	0.021905	0.022512
656	-0.00087	0.002156	0.006873	0.012982	0.01613	0.022303	0.022956
655	-0.00094	0.002354	0.007072	0.013309	0.01648	0.022654	0.023257
654	-0.00109	0.002233	0.007015	0.013383	0.016367	0.022595	0.023361
653	-0.00084	0.002443	0.007205	0.013654	0.01662	0.022935	0.023602
652	-0.00096	0.002302	0.007045	0.013636	0.016745	0.023098	0.023703
651	-0.00099	0.002289	0.007169	0.0138	0.01672	0.022921	0.023779
650	-0.00082	0.002442	0.007399	0.01396	0.016945	0.023289	0.024148
649	-0.00089	0.002479	0.007495	0.013976	0.017194	0.023515	0.024252
648	-0.00082	0.002643	0.007805	0.014361	0.017357	0.02363	0.024491
647	-0.00092	0.002481	0.007733	0.014266	0.017484	0.023823	0.024713
646	-0.001	0.002513	0.007864	0.014417	0.017499	0.024003	0.024865
645	-0.00101	0.002519	0.007911	0.014505	0.017597	0.024169	0.024953
644	-0.00097	0.002729	0.008138	0.01477	0.017919	0.024566	0.025417
643	-0.00085	0.002759	0.008116	0.015001	0.018076	0.024665	0.0256
642	-0.00104	0.002781	0.008135	0.015023	0.018091	0.024699	0.025724
641	-0.00088	0.002868	0.008371	0.015223	0.018263	0.025012	0.025807
640	-0.00098	0.002853	0.008458	0.015325	0.018381	0.025351	0.026335
639	-0.00102	0.002808	0.008432	0.015467	0.018445	0.025394	0.026362
638	-0.00086	0.003032	0.00872	0.015845	0.018841	0.025807	0.026737
637	-0.00106	0.002887	0.008641	0.015666	0.018662	0.025685	0.026762
636	-0.00083	0.002891	0.008927	0.015904	0.018953	0.026089	0.0271
635	-0.00098	0.002861	0.008896	0.016083	0.019026	0.026148	0.027096
634	-0.00079	0.00313	0.009027	0.016174	0.019261	0.026389	0.027533
633	-0.00105	0.002998	0.009042	0.016181	0.01934	0.026466	0.027521
632	-0.00105	0.003155	0.009093	0.016216	0.019506	0.026601	0.027935
631	-0.001	0.003179	0.009257	0.016525	0.019646	0.026913	0.028032
630	-0.00102	0.003098	0.009334	0.016582	0.019621	0.027135	0.02819
629	-0.00107	0.003034	0.009401	0.016616	0.019646	0.027074	0.028302
628	-0.00122	0.003001	0.009455	0.016792	0.019925	0.027382	0.028496
627	-0.00095	0.003258	0.009607	0.016957	0.020043	0.027573	0.028835
626	-0.00104	0.003182	0.009483	0.017087	0.020194	0.027654	0.028792
625	-0.00106	0.003263	0.009636	0.017081	0.020228	0.027894	0.029066
624	-0.00094	0.003312	0.009883	0.017298	0.020471	0.028133	0.029268
623	-0.00113	0.003305	0.009799	0.017526	0.020445	0.028057	0.029316
622	-0.00092	0.00337	0.010003	0.017641	0.020771	0.028417	0.029725
621	-0.00096	0.00328	0.010002	0.017636	0.020783	0.028416	0.029691
620	-0.00114	0.003414	0.010047	0.017718	0.020729	0.028577	0.029979
619	-0.0009	0.003645	0.010262	0.017928	0.020957	0.029069	0.030289
618	-0.00106	0.003489	0.010133	0.017828	0.020995	0.029022	0.030315
617	-0.00102	0.00351	0.010289	0.018069	0.021148	0.029205	0.030545
616	-0.00085	0.003633	0.010364	0.018237	0.02137	0.029408	0.030825
615	-0.00086	0.003505	0.010484	0.018159	0.021294	0.029577	0.030933
614	-0.00096	0.003575	0.010422	0.018177	0.021485	0.029608	0.031036
613	-0.00085	0.003733	0.010605	0.018476	0.02157	0.030032	0.031129

EXCLI Journal 2020;19:48-70 – ISSN 1611-2156
Received: October 03, 2019, accepted: December 19, 2019, published: January 03, 2020

Wavelength (nm)	***Absorption intensity***						
	**concentration of acetylshikonin (M)**						
	A	*B*	*C*	*D*	*E*	*F*	*G*
	0.00	$4.0x10^{-6}$	$8.0x10^{-6}$	$1.0x10^{-5}$	$1.4x10^{-5}$	$1.6x10^{-5}$	$1.8x10^{-5}$
612	-0.00092	0.003729	0.010489	0.018544	0.021576	0.02995	0.031385
611	-0.00087	0.003787	0.010805	0.018634	0.021858	0.030186	0.03168
610	-0.00074	0.003804	0.010807	0.018722	0.021799	0.030325	0.03173
609	-0.00083	0.003707	0.010979	0.018963	0.022137	0.030471	0.031851
608	-0.00073	0.003889	0.011083	0.019074	0.02212	0.030824	0.032166
607	-0.00064	0.003981	0.011314	0.019379	0.02246	0.031082	0.032425
606	-0.0006	0.004045	0.011406	0.019486	0.022619	0.031207	0.0326
605	-0.00064	0.004011	0.011383	0.019505	0.022615	0.031212	0.032702
604	-0.00061	0.004051	0.01139	0.019551	0.022936	0.03135	0.032945
603	-0.00052	0.004235	0.011533	0.019843	0.022968	0.031846	0.033225
602	-0.00046	0.004203	0.011739	0.020016	0.023103	0.031857	0.033407
601	-0.00056	0.004341	0.011771	0.020061	0.023284	0.032176	0.033587
600	-0.00056	0.004299	0.011841	0.020206	0.02334	0.032299	0.033858
599	-0.0006	0.004353	0.011902	0.020233	0.023484	0.032247	0.034043
598	-0.00051	0.004394	0.012044	0.020483	0.023692	0.032716	0.034394
597	-0.00052	0.004471	0.012228	0.02063	0.023879	0.032959	0.034516
596	-0.00041	0.004406	0.012318	0.020781	0.024038	0.033022	0.034805
595	-0.00053	0.004493	0.012354	0.020873	0.024164	0.033263	0.035025
594	-0.00054	0.004429	0.012408	0.020993	0.024369	0.033359	0.035157
593	-0.00053	0.004286	0.012484	0.021132	0.024421	0.033651	0.035464
592	-0.00055	0.004547	0.012672	0.021175	0.024723	0.033828	0.035746
591	-0.00055	0.004561	0.012749	0.021446	0.024754	0.034098	0.035984
590	-0.00058	0.004546	0.013016	0.021496	0.025057	0.034358	0.036183
589	-0.00059	0.004569	0.012937	0.021684	0.025132	0.034564	0.036548
588	-0.00065	0.004439	0.013105	0.021688	0.025391	0.034846	0.036695
587	-0.00073	0.004597	0.013309	0.022124	0.025568	0.03514	0.037113
586	-0.00066	0.004685	0.01318	0.022175	0.02562	0.035342	0.037241
585	-0.0006	0.004791	0.013662	0.022622	0.025964	0.035778	0.037908
584	-0.0007	0.004834	0.013744	0.022762	0.026214	0.035883	0.038107
583	-0.00074	0.004721	0.013684	0.023013	0.026352	0.036169	0.038146
582	-0.00066	0.004703	0.013993	0.022987	0.026589	0.03628	0.038659
581	-0.00076	0.004914	0.014163	0.023341	0.026883	0.03662	0.038974
580	-0.00072	0.004954	0.014264	0.023503	0.027058	0.036908	0.039336
579	-0.00058	0.005009	0.01463	0.023783	0.027353	0.037312	0.039446
578	-0.00076	0.005072	0.01453	0.023891	0.027434	0.037368	0.039675
577	-0.00067	0.005251	0.014888	0.02418	0.027689	0.037356	0.039975
576	-0.00066	0.005182	0.014968	0.024416	0.027892	0.037743	0.04029
575	-0.00064	0.005435	0.015193	0.024547	0.028169	0.038048	0.040565
574	-0.0008	0.005196	0.015142	0.024815	0.028006	0.038123	0.040659
573	-0.00057	0.005463	0.015527	0.024845	0.028156	0.03841	0.041175
572	-0.00062	0.005478	0.015429	0.024836	0.028349	0.038503	0.041249
571	-0.0006	0.005462	0.015577	0.024998	0.028424	0.038873	0.041709
570	-0.0006	0.005617	0.015751	0.025271	0.028892	0.039078	0.041924
569	-0.00069	0.005671	0.015734	0.025403	0.028915	0.039275	0.041984
568	-0.00058	0.005904	0.016162	0.026069	0.029514	0.039928	0.042935
567	-0.00057	0.005739	0.016188	0.025877	0.029551	0.039853	0.042968
566	-0.00054	0.005739	0.016086	0.026053	0.029797	0.040118	0.043208
565	-0.00052	0.005783	0.016265	0.026053	0.029741	0.040174	0.043265

EXCLI Journal 2020;19:48-70 – ISSN 1611-2156
Received: October 03, 2019, accepted: December 19, 2019, published: January 03, 2020

**Wavelength (nm)**	***Absorption intensity***						
	**concentration of acetylshikonin (M)**						
	A	*B*	*C*	*D*	*E*	*F*	*G*
	0.00	$4.0x10^{-6}$	$8.0x10^{-6}$	$1.0x10^{-5}$	$1.4x10^{-5}$	$1.6x10^{-5}$	$1.8x10^{-5}$
564	-0.00072	0.005783	0.016122	0.026002	0.029775	0.040234	0.0433
563	-0.00063	0.005893	0.016274	0.026299	0.030006	0.040248	0.043531
562	-0.00046	0.005948	0.016442	0.026523	0.030053	0.040712	0.043671
561	-0.00057	0.006052	0.016514	0.026525	0.03016	0.040603	0.043631
560	-0.00057	0.005969	0.016445	0.026459	0.030027	0.040577	0.043718
559	-0.00083	0.005598	0.016173	0.026181	0.029916	0.040295	0.043473
558	-0.00069	0.005939	0.0163	0.026438	0.030132	0.040665	0.043817
557	-0.00048	0.006059	0.016516	0.026641	0.030289	0.040809	0.044075
556	-0.00053	0.005888	0.016629	0.026497	0.030339	0.040729	0.043951
555	-0.00054	0.006048	0.016556	0.026789	0.030485	0.040881	0.044272
554	-0.00061	0.005907	0.016475	0.026682	0.030451	0.040931	0.044231
553	-0.00042	0.006062	0.016685	0.026955	0.030577	0.041096	0.04439
552	-0.00048	0.006077	0.016529	0.026814	0.03054	0.041143	0.044533
551	-0.00057	0.006161	0.016598	0.026826	0.030517	0.041259	0.044656
550	-0.00051	0.006215	0.016765	0.026967	0.030552	0.041356	0.044805
549	-0.00041	0.006177	0.01671	0.027071	0.030726	0.041452	0.0449
548	-0.00038	0.006229	0.016887	0.027184	0.03073	0.041575	0.044989
547	-0.00034	0.006068	0.016915	0.027264	0.030962	0.041673	0.045229
546	-0.00037	0.006202	0.017062	0.027458	0.031084	0.041886	0.045315
545	-0.00034	0.00629	0.017121	0.027587	0.031281	0.042033	0.045506
544	-0.0003	0.006291	0.017048	0.027688	0.031368	0.042149	0.045656
543	-0.00036	0.006468	0.017327	0.027873	0.031522	0.042421	0.045951
542	-0.00032	0.006366	0.017316	0.027783	0.031728	0.042511	0.046022
541	-0.00018	0.006459	0.017488	0.028084	0.031894	0.042583	0.046448
540	-0.00035	0.006483	0.017578	0.028171	0.032025	0.042884	0.046596
539	-0.00023	0.00665	0.017893	0.02842	0.032399	0.043176	0.047085
538	-0.00035	0.006488	0.017787	0.02854	0.032262	0.04335	0.047083
537	-0.00023	0.006662	0.01796	0.028828	0.032612	0.043837	0.047476
536	-0.00018	0.006683	0.018148	0.028968	0.032808	0.043979	0.047851
535	-0.00022	0.006795	0.018259	0.029258	0.033046	0.044146	0.048133
534	-0.0001	0.006934	0.018488	0.029429	0.033355	0.044451	0.048416
533	-0.00023	0.006844	0.01846	0.029555	0.033305	0.044462	0.048363
532	-0.00024	0.006937	0.01855	0.029632	0.033612	0.044695	0.048854
531	-0.00019	0.00708	0.018866	0.030035	0.033892	0.045057	0.049056
530	-0.00034	0.007021	0.018822	0.029968	0.033938	0.04519	0.049257
529	-0.00023	0.007199	0.019006	0.030251	0.034093	0.045445	0.049569
528	-0.00016	0.007173	0.01913	0.030282	0.034317	0.045532	0.049693
527	-0.00015	0.007143	0.019206	0.030305	0.034493	0.045885	0.049987
526	-0.00014	0.007315	0.019479	0.030672	0.03462	0.045971	0.050239
525	-0.00033	0.007362	0.019403	0.030649	0.03469	0.046066	0.050215
524	-0.00032	0.007107	0.019335	0.030692	0.034685	0.045757	0.050215
523	-7.64E-05	0.007401	0.019531	0.030865	0.034863	0.046122	0.050618
522	-0.00011	0.007314	0.019427	0.030812	0.034918	0.046075	0.050281
521	-0.00011	0.007425	0.019678	0.030909	0.034892	0.046272	0.050409
520	-0.00012	0.007358	0.019431	0.030739	0.035017	0.046324	0.050308
519	-0.00023	0.007216	0.019307	0.03084	0.034823	0.045933	0.050299
518	9.73E-06	0.007484	0.01949	0.030902	0.034923	0.046127	0.050335
517	-0.00012	0.007182	0.019257	0.030578	0.034667	0.045945	0.050231

EXCLI Journal 2020;19:48-70 – ISSN 1611-2156
Received: October 03, 2019, accepted: December 19, 2019, published: January 03, 2020

Wavelength (nm)	***Absorption intensity***						
	**concentration of acetylshikonin (M)**						
	A	*B*	*C*	*D*	*E*	*F*	*G*
	0.00	$4.0x10^{-6}$	$8.0x10^{-6}$	$1.0x10^{-5}$	$1.4x10^{-5}$	$1.6x10^{-5}$	$1.8x10^{-5}$
516	1.18E-05	0.007407	0.01949	0.030805	0.034911	0.045952	0.05031
515	-0.00016	0.007137	0.019401	0.030657	0.034766	0.045845	0.050115
514	-0.00016	0.007412	0.019337	0.030675	0.034749	0.045868	0.050047
513	-0.00016	0.007209	0.019194	0.030432	0.034464	0.045569	0.049927
512	-0.0002	0.007039	0.019149	0.030398	0.034561	0.045533	0.049669
511	-0.00022	0.007165	0.018968	0.030265	0.034385	0.04542	0.049702
510	-0.00021	0.006951	0.018846	0.030103	0.034205	0.045319	0.049622
509	-0.00024	0.007083	0.018941	0.030209	0.034272	0.045245	0.049407
508	-0.00041	0.006805	0.018736	0.029848	0.034115	0.044881	0.049185
507	-0.00031	0.007018	0.018559	0.02989	0.03401	0.044692	0.049102
506	-0.00043	0.006802	0.018509	0.029744	0.033839	0.044791	0.049108
505	-0.0006	0.006787	0.018244	0.029743	0.033898	0.044538	0.048797
504	-0.00038	0.00677	0.018531	0.029651	0.033942	0.044651	0.048769
503	-0.00041	0.006785	0.018525	0.029837	0.033825	0.04475	0.048899
502	-0.00037	0.006731	0.018387	0.029675	0.03384	0.044493	0.048724
501	-0.00042	0.006867	0.018449	0.02965	0.033837	0.04443	0.048749
500	-0.00034	0.006845	0.018545	0.029691	0.033928	0.044666	0.048839
499	-0.00037	0.006769	0.018332	0.029703	0.033746	0.044574	0.048718
498	-0.00029	0.007028	0.018433	0.029652	0.033865	0.044526	0.048813
497	-9.37E-05	0.007041	0.018499	0.029744	0.033965	0.044586	0.048858
496	2.65E-05	0.007159	0.018491	0.029804	0.034068	0.044639	0.048973
495	-4.47E-05	0.00718	0.018678	0.029766	0.033953	0.044658	0.048821
494	-9.90E-05	0.007019	0.018376	0.029637	0.033817	0.044572	0.04879
493	0.00012	0.007212	0.018527	0.029836	0.034011	0.044522	0.048887
492	-0.00011	0.007229	0.018537	0.0296	0.03388	0.04424	0.048633
491	2.93E-05	0.007209	0.018334	0.02957	0.03398	0.044224	0.048505
490	-8.18E-05	0.007031	0.01842	0.029434	0.033754	0.044055	0.04843
489	0.000109	0.007072	0.018232	0.029505	0.033702	0.043907	0.048081
488	6.78E-05	0.006925	0.018337	0.029368	0.033606	0.043869	0.048167
487	-0.00013	0.007009	0.018034	0.029246	0.033464	0.043466	0.047999
486	-9.32E-05	0.007267	0.018108	0.029169	0.033635	0.043535	0.048076
485	0.000137	0.007027	0.018118	0.029087	0.033527	0.043372	0.047694
484	6.43E-05	0.006985	0.017941	0.028874	0.033219	0.043198	0.047438
483	0.000144	0.007094	0.017726	0.0286	0.033093	0.042905	0.047309
482	0.0002	0.007073	0.017846	0.028664	0.03312	0.04293	0.047127
481	0.000136	0.007155	0.017676	0.02872	0.032847	0.042774	0.046927
480	5.07E-05	0.006867	0.017383	0.028173	0.032655	0.042296	0.046512
479	0.000221	0.006917	0.017299	0.028222	0.032414	0.042227	0.046336
478	9.06E-05	0.006894	0.017001	0.028022	0.032304	0.041837	0.046151
477	-2.35E-05	0.006618	0.016798	0.027674	0.031894	0.041462	0.04552
476	7.27E-05	0.00672	0.017055	0.027592	0.031887	0.041458	0.045643
475	0.000232	0.006822	0.016762	0.027563	0.031828	0.041223	0.045281
474	0.000172	0.006717	0.016682	0.027212	0.031654	0.040866	0.044995
473	0.000118	0.006695	0.016504	0.026994	0.031463	0.040615	0.04483
472	-0.00022	0.006318	0.016226	0.026744	0.030877	0.040144	0.044284
471	-0.00028	0.006241	0.01594	0.026376	0.030814	0.039953	0.043972
470	0.000309	0.006639	0.016161	0.026636	0.030879	0.040013	0.044191
469	0.000252	0.006589	0.016032	0.026471	0.030647	0.039803	0.043871

EXCLI Journal 2020;19:48-70 – ISSN 1611-2156
Received: October 03, 2019, accepted: December 19, 2019, published: January 03, 2020

Wavelength (nm)	***Absorption intensity***						
	**concentration of acetylshikonin (M)**						
	A	*B*	*C*	*D*	*E*	*F*	*G*
	0.00	$4.0x10^{-6}$	$8.0x10^{-6}$	$1.0x10^{-5}$	$1.4x10^{-5}$	$1.6x10^{-5}$	$1.8x10^{-5}$
468	0.000296	0.006535	0.01569	0.026299	0.030456	0.039339	0.043572
467	0.000221	0.006505	0.015837	0.026126	0.030295	0.039257	0.043404
466	0.000261	0.006318	0.015446	0.025929	0.03017	0.038989	0.043048
465	0.000463	0.006566	0.015546	0.025917	0.030122	0.038887	0.042718
464	0.000301	0.006575	0.015514	0.025655	0.029967	0.03821	0.042444
463	0.000273	0.006487	0.01525	0.02542	0.029655	0.038102	0.041935
462	0.000287	0.006469	0.015124	0.025262	0.029314	0.037949	0.041946
461	0.000341	0.006434	0.015092	0.024861	0.029165	0.037594	0.041603
460	0.000313	0.006424	0.014816	0.024864	0.029034	0.037437	0.041526
459	0.000556	0.006718	0.014989	0.024931	0.029149	0.037537	0.04154
458	0.000661	0.006364	0.01473	0.024912	0.029044	0.037264	0.041349
457	0.000486	0.006217	0.01481	0.024556	0.028833	0.037083	0.041155
456	0.000232	0.006228	0.014276	0.024231	0.028634	0.036688	0.040947
455	0.000371	0.006412	0.014502	0.024189	0.028652	0.036552	0.040576
454	0.000248	0.006243	0.014186	0.023991	0.028407	0.036218	0.040446
453	0.000419	0.006355	0.014026	0.023902	0.028147	0.036008	0.040096
452	0.000367	0.006333	0.0141	0.023953	0.027949	0.035776	0.039852
451	0.000457	0.006219	0.013827	0.023593	0.02773	0.035501	0.039596
450	0.000458	0.006125	0.01366	0.023389	0.027683	0.035351	0.039398
449	0.000373	0.006103	0.013494	0.023167	0.027466	0.03493	0.039205
448	0.000532	0.006067	0.01358	0.023146	0.027402	0.034856	0.038929
447	0.0005	0.006193	0.013419	0.02288	0.027141	0.034664	0.038714
446	0.000609	0.006331	0.013441	0.022924	0.027078	0.034532	0.038486
445	0.000361	0.005821	0.012931	0.022466	0.026824	0.034216	0.03812
444	0.000507	0.006154	0.013014	0.022421	0.026847	0.03412	0.038141
443	0.000542	0.00601	0.012995	0.02238	0.026618	0.033898	0.037835
442	0.000577	0.006238	0.012804	0.022374	0.026506	0.0337	0.037846
441	0.000499	0.005928	0.012779	0.022063	0.02636	0.033574	0.037433
440	0.000569	0.006019	0.012626	0.022013	0.0261	0.033404	0.037403
439	0.000481	0.00604	0.012473	0.021935	0.026154	0.033264	0.037176
438	0.000462	0.005858	0.012381	0.021746	0.025829	0.032776	0.037065
437	0.000414	0.005954	0.012168	0.02169	0.025793	0.032863	0.036894
436	0.000448	0.00591	0.011997	0.021281	0.025693	0.032484	0.036527
435	0.000306	0.005759	0.012083	0.02144	0.025545	0.032565	0.036466
434	0.000597	0.006034	0.012142	0.02139	0.025686	0.032428	0.036403
433	0.000229	0.00575	0.011679	0.021094	0.025469	0.031973	0.036131
432	0.000393	0.005849	0.01192	0.021026	0.025514	0.031991	0.036197
431	0.000951	0.006089	0.012199	0.021215	0.025714	0.032382	0.036403
430	0.000606	0.006094	0.012001	0.021084	0.025475	0.032215	0.036242
429	0.000675	0.00615	0.012036	0.021075	0.025466	0.032138	0.036089
428	0.000746	0.005933	0.011879	0.021019	0.025271	0.032097	0.036075
427	0.000697	0.005996	0.011924	0.020754	0.025225	0.031839	0.035993
426	0.000966	0.006415	0.011843	0.021064	0.025147	0.031931	0.035783
425	0.000975	0.00621	0.011842	0.021014	0.025324	0.031967	0.036004
424	0.000962	0.006016	0.01152	0.02062	0.02491	0.031494	0.035508
423	0.000802	0.006056	0.011621	0.020857	0.025091	0.031574	0.035699
422	0.000875	0.006163	0.011599	0.020862	0.025072	0.031492	0.035874
421	0.000901	0.006222	0.011741	0.020705	0.024876	0.031383	0.03536

Wavelength (nm)	Absorption intensity						
	concentration of acetylshikonin (M)						
	A	*B*	*C*	*D*	*E*	*F*	*G*
	0.00	$4.0x10^{-6}$	$8.0x10^{-6}$	$1.0x10^{-5}$	$1.4x10^{-5}$	$1.6x10^{-5}$	$1.8x10^{-5}$
420	0.000905	0.006088	0.011456	0.020328	0.02483	0.031397	0.035174
419	0.001076	0.005935	0.011337	0.020521	0.024686	0.031292	0.035353
418	0.000541	0.00574	0.011149	0.02009	0.024758	0.031246	0.035109
417	0.000655	0.005759	0.011356	0.020261	0.024589	0.031278	0.035234
416	0.000714	0.006059	0.011391	0.020514	0.024798	0.031262	0.035383
415	0.000504	0.005549	0.010997	0.019935	0.024484	0.030887	0.035153
414	0.000811	0.006067	0.011043	0.020378	0.024776	0.031187	0.035193
413	0.000709	0.005696	0.011074	0.020196	0.024543	0.03111	0.035064
412	0.000439	0.00568	0.010691	0.020142	0.024183	0.030938	0.034718
411	0.000374	0.005512	0.010717	0.019978	0.024356	0.030794	0.034767
410	0.000347	0.005324	0.010825	0.019891	0.024286	0.030622	0.03473
409	0.000543	0.005832	0.01082	0.020254	0.024256	0.030976	0.034926
408	0.000529	0.005703	0.01103	0.020148	0.024558	0.031207	0.035368
407	0.00069	0.005696	0.011168	0.020265	0.024495	0.031257	0.035157
406	0.000499	0.005581	0.010973	0.020084	0.0246	0.031194	0.035002
405	0.000353	0.005579	0.010839	0.020187	0.024467	0.030681	0.034813
404	0.000906	0.005795	0.011161	0.020182	0.024571	0.031414	0.035193
403	0.001012	0.006144	0.011257	0.020474	0.024746	0.031631	0.035477
402	0.000987	0.005961	0.011178	0.020445	0.024898	0.031714	0.035617
401	0.00079	0.005678	0.010972	0.020156	0.0248	0.031396	0.035267
400	0.000874	0.005954	0.011508	0.020686	0.024881	0.031972	0.035958
399	0.000764	0.005913	0.011233	0.020557	0.024919	0.031619	0.035801
398	0.000886	0.005854	0.011146	0.020666	0.024696	0.031707	0.03574
397	0.000785	0.005776	0.011124	0.020618	0.024857	0.031662	0.035769
396	0.000717	0.005905	0.011114	0.020665	0.025065	0.031867	0.035931
395	0.000561	0.0056	0.011201	0.020497	0.024879	0.031774	0.036091
394	0.000758	0.005989	0.011185	0.021042	0.025235	0.031813	0.036096
393	0.000698	0.005795	0.011383	0.020668	0.025345	0.032038	0.03644
392	0.000323	0.005861	0.011235	0.020781	0.02483	0.032029	0.036047
391	0.001099	0.006434	0.011774	0.02103	0.025628	0.032941	0.036689
390	0.000898	0.006332	0.011565	0.021362	0.025928	0.03291	0.036951
389	0.000939	0.006376	0.011853	0.021027	0.025683	0.032594	0.036375
388	0.001548	0.006257	0.011518	0.021304	0.025729	0.032903	0.036953
387	0.000762	0.006042	0.011539	0.021106	0.025631	0.032715	0.036939
386	0.001023	0.006026	0.011528	0.021449	0.025741	0.032794	0.036789
385	0.000613	0.0059	0.011389	0.021347	0.025718	0.032873	0.037007
384	0.000752	0.005944	0.011684	0.021618	0.025974	0.033141	0.037137
383	0.001178	0.258889	0.012038	0.021504	0.025715	0.033094	0.037541
382	0.001465	0.006407	0.012061	0.022011	0.026322	0.033486	0.037674
381	0.001021	0.006398	0.011837	0.021809	0.026379	0.033558	0.037907
380	0.000872	0.00646	0.011963	0.021765	0.026556	0.033957	0.037691
379	0.000992	0.006356	0.011911	0.022125	0.026577	0.033802	0.038006
378	0.000972	0.006416	0.011975	0.022241	0.026752	0.034042	0.03846
377	0.001276	0.006637	0.012297	0.022657	0.026695	0.03417	0.038254
376	0.000403	0.005809	0.011692	0.021682	0.026684	0.034223	0.038331
375	0.001908	0.00778	0.013455	0.02381	0.027006	0.03492	0.03903
374	0.001065	0.006663	0.012781	0.022776	0.027334	0.035114	0.038993
373	0.000622	0.006255	0.012366	0.022684	0.026773	0.034396	0.038574

EXCLI Journal 2020;19:48-70 – ISSN 1611-2156
Received: October 03, 2019, accepted: December 19, 2019, published: January 03, 2020

Wavelength (nm)	***Absorption intensity***						
	**concentration of acetylshikonin (M)**						
	A	*B*	*C*	*D*	*E*	*F*	*G*
	0.00	$4.0x10^{-6}$	$8.0x10^{-6}$	$1.0x10^{-5}$	$1.4x10^{-5}$	$1.6x10^{-5}$	$1.8x10^{-5}$
372	0.001739	0.007211	0.013285	0.023455	0.027841	0.03546	0.039591
371	0.001224	0.007089	0.012778	0.023455	0.027661	0.035596	0.039652
370	0.001115	0.006655	0.012296	0.022883	0.027269	0.035312	0.039462
369	0.001296	0.006951	0.012668	0.023211	0.027584	0.035761	0.039994
368	0.001327	0.007162	0.013159	0.023629	0.028165	0.036229	0.040373
367	0.001203	0.006692	0.012996	0.023865	0.027991	0.036069	0.040242
366	0.00143	0.006827	0.013122	0.023872	0.028168	0.036598	0.040686
365	0.002209	0.007389	0.01426	0.024584	0.028774	0.037547	0.04107
364	0.000927	0.007526	0.013443	0.024296	0.029011	0.03659	0.040658
363	0.00154	0.006996	0.013313	0.024142	0.028363	0.03709	0.041617
362	0.003677	0.007401	0.014335	0.025003	0.029791	0.037678	0.041737
361	0.000324	0.00566	0.011938	0.023186	0.027558	0.035945	0.040162
360	0.001341	0.008375	0.015391	0.026433	0.031106	0.039174	0.04235
359	0.002628	0.008076	0.013875	0.025565	0.030123	0.038286	0.041497
358	0.001751	0.007154	0.014839	0.025382	0.029055	0.038086	0.041873
357	0.002617	0.007005	0.014871	0.025418	0.029549	0.038448	0.042124
356	-0.00097	0.005831	0.012397	0.023993	0.027719	0.036707	0.041047
355	0.001146	0.010654	0.017112	0.028154	0.032441	0.041211	0.045952
354	0.001498	0.007356	0.014301	0.024705	0.028574	0.038344	0.042962
353	0.000498	0.007208	0.014081	0.025966	0.029105	0.039646	0.042963
352	0.001152	0.005609	0.01221	0.023195	0.028234	0.037403	0.041094
351	0.002224	0.012285	0.017692	0.029661	0.034596	0.04346	0.047103
350	0.003625	0.008551	0.015582	0.027688	0.03188	0.043	0.045588
349	0.001199	0.009464	0.014472	0.026279	0.030354	0.0389	0.043612
348	-0.00325	0.006188	0.011594	0.023457	0.029789	0.037181	0.041099
347	-0.0028	0.006447	0.011564	0.022822	0.029777	0.03825	0.041296
346	-0.00216	0.006638	0.012869	0.024445	0.030441	0.03854	0.042463
345	-0.00253	0.006422	0.012434	0.023897	0.029951	0.038682	0.041572
344	-0.00252	0.006661	0.012482	0.024469	0.031063	0.03888	0.042566
343	-0.00204	0.006509	0.012745	0.024236	0.030325	0.039087	0.042317
342	-0.00179	0.007107	0.01283	0.02486	0.031324	0.039747	0.04372
341	-0.00245	0.006935	0.01301	0.0247	0.031454	0.039811	0.043178
340	-0.00293	0.005591	0.012645	0.023921	0.030376	0.039129	0.042787
339	-0.00236	0.007192	0.01395	0.025721	0.031466	0.040768	0.04433
338	-0.00212	0.007839	0.014119	0.026243	0.032396	0.041146	0.044626
337	-0.00219	0.00712	0.013258	0.025589	0.03188	0.040856	0.044945
336	-0.00218	0.007768	0.014409	0.026244	0.032816	0.041948	0.045225
335	-0.00272	0.007323	0.013762	0.026034	0.032499	0.041964	0.045309
334	-0.00221	0.008149	0.014668	0.026791	0.033255	0.042685	0.045811
333	-0.00159	0.007664	0.014866	0.027056	0.03374	0.042727	0.04639
332	-0.0017	0.00795	0.014274	0.027232	0.033357	0.042937	0.046668
331	-0.00195	0.007836	0.014673	0.027311	0.033663	0.043266	0.04681
330	-0.00245	0.00766	0.014436	0.027798	0.034076	0.04287	0.046311
329	-0.00196	0.008011	0.015562	0.028185	0.034727	0.044304	0.047717
328	-0.00149	0.008671	0.016013	0.028955	0.035009	0.045017	0.048134
327	-0.00166	0.008036	0.015538	0.028226	0.034877	0.044821	0.048054
326	-0.00238	0.008043	0.015526	0.027889	0.034879	0.044999	0.048279
325	-0.00157	0.008699	0.016581	0.029011	0.035898	0.046354	0.049508

Wavelength (nm)	***Absorption intensity***						
	**concentration of acetylshikonin (M)**						
	A	*B*	*C*	*D*	*E*	*F*	*G*
	0.00	$4.0x10^{-6}$	$8.0x10^{-6}$	$1.0x10^{-5}$	$1.4x10^{-5}$	$1.6x10^{-5}$	$1.8x10^{-5}$
324	-0.00189	0.008189	0.015949	0.028811	0.035472	0.04655	0.049619
323	-0.00161	0.008317	0.016612	0.029349	0.0366	0.046582	0.049736
322	-0.00124	0.009007	0.016942	0.029966	0.036861	0.047088	0.050897
321	-0.0016	0.008284	0.017049	0.029894	0.036726	0.047171	0.050847
320	-0.00136	0.00972	0.017513	0.030735	0.037781	0.048004	0.05158
319	-0.00163	0.009311	0.017968	0.030739	0.03803	0.048335	0.052223
318	-0.00074	0.00939	0.017961	0.03126	0.038421	0.04859	0.052684
317	-0.00134	0.009517	0.017555	0.03128	0.037828	0.048785	0.052549
316	-0.00128	0.009194	0.018202	0.031106	0.038539	0.049392	0.053301
315	-0.00129	0.009519	0.018498	0.031926	0.039397	0.050394	0.054188
314	-0.00056	0.010545	0.019803	0.033165	0.040368	0.051119	0.055166
313	-0.00087	0.010321	0.019611	0.033003	0.040472	0.05107	0.055434
312	-0.00054	0.010618	0.019632	0.032931	0.040967	0.052116	0.05563
311	-0.00052	0.010543	0.019926	0.033364	0.04103	0.052426	0.05634
310	-0.00043	0.010638	0.020541	0.033755	0.04136	0.052787	0.056439
309	-0.00043	0.010842	0.019882	0.034015	0.041558	0.053149	0.05717
308	-0.00127	0.010574	0.020678	0.034614	0.041923	0.053627	0.057426
307	-0.00058	0.011257	0.020702	0.034665	0.042544	0.054468	0.058306
306	-0.0005	0.010807	0.021367	0.035419	0.042995	0.055148	0.058608
305	-7.06E-05	0.011532	0.021824	0.035687	0.043768	0.055808	0.059491
304	0.000728	0.012437	0.022813	0.036812	0.044749	0.057227	0.061194
303	0.000666	0.012918	0.023349	0.037323	0.045005	0.057687	0.061341
302	0.001533	0.01338	0.024474	0.038214	0.046293	0.058836	0.063039
301	0.00213	0.013553	0.025086	0.039046	0.047572	0.060249	0.064015
300	0.002927	0.015204	0.026695	0.04038	0.048626	0.061646	0.065704
299	0.003875	0.016016	0.027411	0.041684	0.049779	0.062849	0.067078
298	0.005339	0.017586	0.029131	0.043527	0.051763	0.064917	0.069317
297	0.007071	0.018853	0.031163	0.045324	0.053238	0.066775	0.071234
296	0.008874	0.020936	0.033304	0.048056	0.055781	0.068856	0.07364
295	0.011067	0.022738	0.035505	0.0504	0.058154	0.072346	0.076294
294	0.013146	0.025274	0.038151	0.05293	0.06077	0.074707	0.079514
293	0.016226	0.028582	0.041106	0.056215	0.064084	0.078745	0.083362
292	0.018948	0.031131	0.04468	0.059834	0.067083	0.082062	0.086713
291	0.022446	0.034516	0.047996	0.063578	0.071045	0.085612	0.090598
290	0.025916	0.037962	0.051848	0.067104	0.074378	0.08948	0.09465
289	0.029849	0.041781	0.056272	0.071583	0.078659	0.09398	0.099201
288	0.03251	0.044592	0.05931	0.074463	0.081209	0.097128	0.102308
287	0.037729	0.04911	0.064195	0.07925	0.086709	0.102249	0.107812
286	0.041615	0.052952	0.068466	0.083962	0.090416	0.1064	0.111838
285	0.045776	0.057355	0.072685	0.08846	0.095054	0.111468	0.116616
284	0.049748	0.060628	0.076884	0.09243	0.098664	0.115432	0.12092
283	0.054575	0.065358	0.081914	0.097553	0.103549	0.120426	0.125956
282	0.058896	0.069467	0.086179	0.102274	0.1079	0.124732	0.130701
281	0.062411	0.073149	0.090321	0.106091	0.111598	0.129195	0.135006
280	0.066558	0.076959	0.09421	0.110011	0.115423	0.133322	0.13915
279	0.070957	0.080655	0.098224	0.114371	0.119321	0.137661	0.143556
278	0.074677	0.084489	0.101823	0.117926	0.123002	0.141274	0.147405
277	0.078154	0.087529	0.105744	0.121356	0.12627	0.144805	0.151083

EXCLI Journal 2020;19:48-70 – ISSN 1611-2156
Received: October 03, 2019, accepted: December 19, 2019, published: January 03, 2020

**Wavelength (nm)**	***Absorption intensity***						
	**concentration of acetylshikonin (M)**						
	A	*B*	*C*	*D*	*E*	*F*	*G*
	0.00	$4.0x10^{-6}$	$8.0x10^{-6}$	$1.0x10^{-5}$	$1.4x10^{-5}$	$1.6x10^{-5}$	$1.8x10^{-5}$
276	0.082078	0.091252	0.10935	0.125396	0.130013	0.148722	0.155218
275	0.085891	0.094801	0.113209	0.129302	0.13341	0.152603	0.158954
274	0.08933	0.098423	0.116974	0.132935	0.137045	0.156289	0.162619
273	0.093412	0.101698	0.12028	0.136485	0.140366	0.160012	0.166347
272	0.096806	0.105188	0.123806	0.140272	0.143876	0.163539	0.169832
271	0.100143	0.10861	0.127379	0.143561	0.146935	0.166851	0.173314
270	0.103668	0.111433	0.130422	0.146669	0.150016	0.169985	0.176726
269	0.107016	0.11468	0.133544	0.150108	0.153159	0.173273	0.179895
268	0.110256	0.117641	0.13654	0.152746	0.155784	0.175923	0.182685
267	0.113131	0.120138	0.13925	0.155601	0.158316	0.178919	0.185661
266	0.115807	0.123138	0.141599	0.157982	0.160765	0.181107	0.187787
265	0.11853	0.125566	0.144089	0.16096	0.163245	0.183877	0.190556
264	0.120913	0.12766	0.146475	0.162851	0.165247	0.185948	0.192599
263	0.123431	0.129792	0.14904	0.165488	0.167544	0.188456	0.194968
262	0.125141	0.131639	0.150731	0.167002	0.169122	0.190267	0.196913
261	0.127071	0.133211	0.152045	0.168464	0.170651	0.19177	0.19842
260	0.128512	0.134763	0.154121	0.170685	0.172265	0.193697	0.20011
259	0.129339	0.135389	0.154831	0.170836	0.172809	0.19416	0.200659
258	0.129525	0.135515	0.154954	0.171124	0.172927	0.194465	0.200712
257	0.12962	0.135588	0.155058	0.171287	0.173352	0.194533	0.200795
256	0.128887	0.135368	0.154735	0.171042	0.172859	0.194253	0.200427
255	0.127991	0.134455	0.153967	0.169993	0.172177	0.193265	0.200032
254	0.12677	0.133366	0.152354	0.168964	0.170982	0.192783	0.198801
253	0.125009	0.131685	0.150808	0.167007	0.169101	0.191169	0.197064
252	0.122959	0.129773	0.148953	0.164939	0.167635	0.189662	0.195644
251	0.12054	0.127536	0.146614	0.163332	0.165519	0.188007	0.193577
250	0.117081	0.124777	0.143697	0.160236	0.163122	0.184942	0.190837
249	0.11367	0.121653	0.140949	0.157272	0.16053	0.182443	0.188424
248	0.11032	0.118477	0.13758	0.154115	0.157837	0.17969	0.184988
247	0.106267	0.115338	0.134206	0.150916	0.154234	0.176953	0.18213
246	0.102378	0.11109	0.130336	0.147005	0.151245	0.173799	0.179063
245	0.098374	0.108308	0.126908	0.143941	0.148137	0.171057	0.175887
244	0.093914	0.104752	0.123606	0.140949	0.145039	0.168072	0.173572
243	0.090237	0.101125	0.120646	0.137891	0.142677	0.165713	0.170816
242	0.086018	0.097894	0.117782	0.13479	0.139815	0.163541	0.168538
241	0.082428	0.094948	0.115131	0.132294	0.137672	0.162417	0.16681
240	0.078295	0.091623	0.112227	0.130336	0.136202	0.161104	0.165948
239	0.074936	0.089155	0.110754	0.129309	0.13512	0.161118	0.165758
238	0.071586	0.087409	0.110133	0.128974	0.135157	0.162696	0.167218
237	0.068873	0.085707	0.109714	0.128971	0.136517	0.165181	0.170314
236	0.066419	0.085361	0.111235	0.131634	0.139621	0.170596	0.175773
235	0.064184	0.085746	0.114494	0.13609	0.144701	0.178209	0.184599
234	0.062489	0.087335	0.119297	0.142713	0.152534	0.190416	0.197075
233	0.061125	0.090435	0.126648	0.152911	0.163664	0.207086	0.215423
232	0.060538	0.095656	0.137911	0.167888	0.180435	0.231215	0.240944
231	0.060358	0.102924	0.153698	0.18784	0.203494	0.263662	0.275814
230	0.060708	0.113889	0.175786	0.215939	0.235022	0.30774	0.323719
229	0.06145	0.128299	0.20529	0.252874	0.276886	0.366604	0.386093

EXCLI Journal 2020;19:48-70 – ISSN 1611-2156
Received: October 03, 2019, accepted: December 19, 2019, published: January 03, 2020

Wavelength (nm)	Absorption intensity						
	concentration of acetylshikonin (M)						
	A	*B*	*C*	*D*	*E*	*F*	*G*
	0.00	$4.0x10^{-6}$	$8.0x10^{-6}$	$1.0x10^{-5}$	$1.4x10^{-5}$	$1.6x10^{-5}$	$1.8x10^{-5}$
228	0.062246	0.148048	0.244135	0.301989	0.332787	0.444237	0.469529
227	0.064137	0.174127	0.294897	0.36566	0.404672	0.544161	0.576338
226	0.066345	0.207937	0.360795	0.447841	0.497919	0.673816	0.715527
225	0.068957	0.251042	0.442811	0.551177	0.615165	0.834924	0.887151
224	0.072418	0.305778	0.548178	0.682252	0.764099	1.039092	1.105302
223	0.076062	0.372858	0.676914	0.841998	0.945559	1.288349	1.370284
222	0.080627	0.455285	0.834305	1.038151	1.167394	1.589977	1.692098
221	0.086253	0.554417	1.022067	1.270698	1.431342	1.945805	2.071082
220	0.09186	0.671082	1.244175	1.54533	1.741143	2.363351	2.513694
219	0.098305	0.805014	1.497749	1.859762	2.094455	2.821687	2.997033
218	0.10533	0.95746	1.785359	2.213409	2.492331	3.324754	3.493207
217	0.112159	1.124081	2.102714	2.599484	2.919472	3.772183	3.918432
216	0.119712	1.307404	2.445003	3.01464	3.355706	4.15908	4.239654
215	0.126035	1.496349	2.798226	3.418548	3.758468	4.414317	4.470268
214	0.133953	1.692627	3.155924	3.817026	4.148371	4.652831	4.616613
213	0.141029	1.884617	3.493146	4.112285	4.423019	4.881563	4.845035
212	0.14869	2.069827	3.807101	4.305293	4.511214	5.037714	5.014989
211	0.15627	2.235554	4.109299	4.817483	5.026067	5.201296	10
210	0.162089	2.367286	4.24167	4.629912	4.78249	5.039994	5.051894
209	0.169361	2.474583	4.388962	4.617496	4.703551	4.858348	4.90627
208	0.175716	2.551466	4.4371	4.722875	4.828283	5.082644	4.989107
207	0.182928	2.588531	4.565995	4.715483	4.811374	5.070168	5.069466
206	0.188025	2.591259	4.64037	4.882267	4.746224	4.930647	4.929903
205	0.19526	2.564208	4.51943	5.010899	4.929057	5.224056	10
204	0.19808	2.518012	4.313242	4.389608	4.482544	4.565109	4.618262
203	0.201322	2.469949	4.211734	4.211515	4.348977	4.519676	4.747885
202	0.209598	2.406981	3.85913	3.791775	3.985424	4.260239	4.284307
201	0.20371	2.325523	3.460264	3.404591	3.506865	3.645355	3.744905
200	0.198778	2.160366	2.800901	2.80356	2.904021	3.039932	3.237933

EXCLI Journal 2020;19:48-70 – ISSN 1611-2156
Received: October 03, 2019, accepted: December 19, 2019, published: January 03, 2020

Table 3: Absorption intensity in the wavelength range of 200-800 nm for absorption spectra of CT-DNA fixed concentration (1.77 x 10^{-5} M), in the absence (*A*) and presence of increasing concentration of *β*-hydroxyisovalerylshikonin (*B*-*G*)

Wavelength (nm)	***Absorption intensity***						
	**concentration *β*-hydroxyisovalerylshikonin (M)**						
	A	*B*	*C*	*D*	*E*	*F*	*G*
	0.00	4.0x10^{-6}	8.0x10^{-6}	1.0x10^{-5}	1.4x10^{-5}	1.6x10^{-5}	1.8x10^{-5}
800	-0.00205	0.001329	-0.00105	0.005734	0.003688	0.011573	0.012185
799	-0.00225	0.00112	-0.0012	0.005691	0.003557	0.011726	0.011868
798	-0.00198	0.001485	-0.00094	0.006278	0.004014	0.012053	0.012281
797	-0.00156	0.001632	-0.0007	0.006352	0.003661	0.012299	0.012364
796	-0.00208	0.00139	-0.00087	0.005725	0.003416	0.011891	0.011985
795	-0.00186	0.001617	-0.00105	0.006064	0.003879	0.012322	0.012455
794	-0.00203	0.001137	-0.0011	0.006122	0.003558	0.012132	0.012706
793	-0.00194	0.001366	-0.00095	0.005842	0.003795	0.012122	0.012344
792	-0.00197	0.001424	-0.00095	0.006289	0.003759	0.012393	0.012611
791	-0.00231	0.001173	-0.00106	0.005866	0.003784	0.011996	0.012264
790	-0.00194	0.001451	-0.00095	0.006411	0.003971	0.01219	0.01274
789	-0.0021	0.001591	-0.00093	0.00649	0.0039	0.012411	0.012829
788	-0.00226	0.001133	-0.00129	0.006079	0.003907	0.012155	0.01241
787	-0.00198	0.001251	-0.00107	0.006432	0.003955	0.012398	0.012803
786	-0.00228	0.001423	-0.00079	0.006356	0.003884	0.012354	0.012855
785	-0.00221	0.001433	-0.00112	0.006347	0.003878	0.012654	0.012806
784	-0.00202	0.001332	-0.00107	0.006284	0.004	0.012504	0.012829
783	-0.00225	0.001568	-0.00097	0.006159	0.004162	0.012831	0.013004
782	-0.00227	0.001465	-0.0009	0.006128	0.004271	0.01255	0.012868
781	-0.00239	0.001363	-0.00104	0.006349	0.003854	0.012758	0.013115
780	-0.00211	0.001622	-0.0012	0.006153	0.004027	0.012616	0.012811
779	-0.002	0.001348	-0.0011	0.006295	0.004088	0.01255	0.013083
778	-0.00212	0.001536	-0.0007	0.006614	0.004291	0.012995	0.013091
777	-0.00207	0.001415	-0.00088	0.006395	0.004312	0.012892	0.013274
776	-0.00212	0.001305	-0.00102	0.006404	0.004343	0.013106	0.013184
775	-0.00211	0.001586	-0.00101	0.006432	0.004202	0.012846	0.013471
774	-0.00209	0.001585	-0.00117	0.006664	0.004312	0.012993	0.013324
773	-0.00232	0.001697	-0.00081	0.00655	0.004318	0.013098	0.013428
772	-0.00195	0.001625	-0.00109	0.006523	0.004289	0.012871	0.013207
771	-0.002	0.001484	-0.00072	0.006821	0.004482	0.013059	0.013336
770	-0.00218	0.001678	-0.00097	0.006549	0.004382	0.013144	0.013813
769	-0.00231	0.001201	-0.00093	0.006639	0.00428	0.013225	0.013755
768	-0.00188	0.001557	-0.00099	0.00667	0.004406	0.013251	0.013661
767	-0.00218	0.001637	-0.00074	0.006669	0.004566	0.013415	0.013612
766	-0.0019	0.001682	-0.00084	0.006882	0.004547	0.013394	0.013969
765	-0.00208	0.001605	-0.00085	0.006677	0.004244	0.013299	0.013867
764	-0.0022	0.001774	-0.00082	0.006715	0.004501	0.013529	0.013911
763	-0.00212	0.00134	-0.00089	0.006504	0.004431	0.013267	0.013834
762	-0.00215	0.001863	-0.0006	0.007204	0.004749	0.013837	0.014147
761	-0.00216	0.001694	-0.00081	0.006939	0.004553	0.013671	0.01417
760	-0.00196	0.001837	-0.0007	0.007081	0.004853	0.013962	0.01427
759	-0.00209	0.001638	-0.00103	0.00682	0.004582	0.013679	0.014002
758	-0.00231	0.001743	-0.00078	0.006718	0.004416	0.013747	0.013858
757	-0.00209	0.001747	-0.00095	0.006951	0.004526	0.014077	0.014376

EXCLI Journal 2020;19:48-70 – ISSN 1611-2156
Received: October 03, 2019, accepted: December 19, 2019, published: January 03, 2020

	Absorption intensity						
**Wavelength (nm)**	**concentration *β*-hydroxyisovalerylshikonin (M)**						
	A	*B*	*C*	*D*	*E*	*F*	*G*
	0.00	$4.0x10^{-6}$	$8.0x10^{-6}$	$1.0x10^{-5}$	$1.4x10^{-5}$	$1.6x10^{-5}$	$1.8x10^{-5}$
756	-0.0022	0.001756	-0.00083	0.007077	0.00473	0.013979	0.014374
755	-0.00226	0.001788	-0.00099	0.007135	0.004617	0.013811	0.014398
754	-0.00204	0.001803	-0.00086	0.007151	0.004856	0.013977	0.01439
753	-0.00211	0.001681	-0.00053	0.007139	0.004931	0.014029	0.014415
752	-0.00206	0.0018	-0.00076	0.00712	0.004849	0.014162	0.014504
751	-0.002	0.001848	-0.0009	0.00722	0.005067	0.014232	0.014747
750	-0.00238	0.001681	-0.00079	0.007206	0.004816	0.014095	0.014636
749	-0.0023	0.001677	-0.00057	0.007159	0.004988	0.014335	0.014782
748	-0.00236	0.001875	-0.00079	0.007276	0.004954	0.014403	0.014756
747	-0.0021	0.001788	-0.00068	0.007367	0.004982	0.014356	0.014792
746	-0.00236	0.001727	-0.00063	0.007281	0.004878	0.014303	0.014815
745	-0.00214	0.002031	-0.00054	0.007412	0.005078	0.014899	0.015237
744	-0.00215	0.001749	-0.00055	0.007349	0.005098	0.014617	0.014934
743	-0.00222	0.001736	-0.00074	0.007438	0.005065	0.014667	0.015122
742	-0.00218	0.00181	-0.0006	0.007424	0.005187	0.014723	0.015232
741	-0.00224	0.001871	-0.00062	0.007494	0.005091	0.01462	0.015192
740	-0.00208	0.002029	-0.00067	0.007682	0.005241	0.015045	0.015389
739	-0.00208	0.002065	-0.00056	0.00769	0.005316	0.015157	0.015561
738	-0.00222	0.002087	-0.00069	0.007769	0.005459	0.014987	0.01546
737	-0.0022	0.002024	-0.00073	0.007701	0.00536	0.01505	0.015576
736	-0.00229	0.001954	-0.00052	0.007852	0.005382	0.015228	0.015818
735	-0.00204	0.002173	-0.00049	0.007761	0.00542	0.015368	0.015654
734	-0.00221	0.001793	-0.00082	0.007827	0.005401	0.01525	0.015648
733	-0.00213	0.002049	-0.00052	0.007989	0.005517	0.015363	0.015905
732	-0.00233	0.002013	-0.00067	0.007958	0.005549	0.015486	0.016106
731	-0.00228	0.001779	-0.00066	0.007895	0.005533	0.015457	0.015932
730	-0.00218	0.002158	-0.00052	0.008111	0.005563	0.01565	0.016231
729	-0.0023	0.00184	-0.00058	0.008058	0.005694	0.015596	0.016193
728	-0.00204	0.002049	-0.00052	0.008365	0.005896	0.015939	0.0165
727	-0.00224	0.002037	-0.00054	0.008029	0.005644	0.015848	0.016324
726	-0.00205	0.002055	-0.0005	0.008138	0.005742	0.015955	0.016453
725	-0.00232	0.001983	-0.00055	0.008208	0.005703	0.015897	0.016521
724	-0.00208	0.002067	-0.00052	0.008165	0.00582	0.016058	0.016716
723	-0.00213	0.002073	-0.00051	0.008228	0.005812	0.016053	0.016912
722	-0.00234	0.002023	-0.00061	0.008154	0.005787	0.016108	0.01664
721	-0.00218	0.00204	-0.00041	0.008367	0.005903	0.016277	0.016826
720	-0.00226	0.00204	-0.0005	0.008468	0.005907	0.016508	0.01685
719	-0.00234	0.001962	-0.00055	0.008338	0.00596	0.016398	0.016885
718	-0.00225	0.002282	-0.00039	0.008557	0.00596	0.016657	0.017245
717	-0.00224	0.002173	-0.00034	0.008554	0.005935	0.016572	0.017295
716	-0.00214	0.002162	-0.00017	0.008605	0.006102	0.01688	0.017584
715	-0.00211	0.002416	-0.00023	0.00884	0.006144	0.016948	0.017588
714	-0.00217	0.002295	-0.00012	0.008783	0.006149	0.017092	0.017617
713	-0.00209	0.002322	-0.00016	0.008814	0.006356	0.017205	0.017777
712	-0.00202	0.00241	-7.65E-05	0.008998	0.006308	0.017227	0.017999
711	-0.00207	0.002328	-0.00018	0.00893	0.006348	0.017392	0.017944
710	-0.00193	0.002459	-0.00011	0.009123	0.006503	0.017433	0.018088
709	-0.00234	0.002326	-0.00025	0.008863	0.006395	0.017269	0.018036

Wavelength (nm)	***Absorption intensity***						
	**concentration β-hydroxyisovalerylshikonin (M)**						
	A	*B*	*C*	*D*	*E*	*F*	*G*
	0.00	$4.0x10^{-6}$	$8.0x10^{-6}$	$1.0x10^{-5}$	$1.4x10^{-5}$	$1.6x10^{-5}$	$1.8x10^{-5}$
708	-0.00178	0.002494	-4.79E-05	0.009105	0.006619	0.017514	0.018299
707	-0.00185	0.002382	-0.00011	0.009237	0.006533	0.017678	0.01845
706	-0.00212	0.002505	-0.00014	0.009207	0.006631	0.017732	0.018587
705	-0.00213	0.002432	-4.77E-05	0.009341	0.006682	0.017811	0.018598
704	-0.002	0.002406	-5.30E-05	0.00938	0.006673	0.018008	0.018718
703	-0.00219	0.002526	-0.00027	0.009251	0.006615	0.017882	0.018657
702	-0.00211	0.002493	8.88E-05	0.009409	0.006853	0.018017	0.018913
701	-0.00201	0.002601	9.46E-05	0.009613	0.006916	0.018314	0.019091
700	-0.00202	0.002522	0.000114	0.00954	0.006764	0.018399	0.01913
699	-0.00219	0.002631	-4.19E-05	0.009782	0.006869	0.018342	0.019283
698	-0.00203	0.002578	0.000123	0.009709	0.007027	0.018595	0.019349
697	-0.00204	0.002534	3.94E-05	0.009827	0.007026	0.018622	0.019557
696	-0.00199	0.002735	0.000267	0.00992	0.00733	0.018865	0.019588
695	-0.00214	0.002707	1.35E-05	0.009761	0.007048	0.018817	0.019689
694	-0.00231	0.002554	-2.77E-05	0.00981	0.007187	0.018803	0.019825
693	-0.00211	0.002707	0.000133	0.010053	0.007404	0.019083	0.019947
692	-0.00235	0.002572	5.07E-06	0.010087	0.007259	0.019244	0.02001
691	-0.00235	0.002801	0.000126	0.010145	0.00733	0.019199	0.020184
690	-0.00219	0.002748	0.000161	0.010116	0.007545	0.019365	0.020285
689	-0.00231	0.002707	0.000225	0.01017	0.007494	0.0196	0.020605
688	-0.00232	0.002796	0.000291	0.010382	0.00769	0.019559	0.020727
687	-0.00222	0.002733	0.000299	0.010382	0.007594	0.01982	0.020751
686	-0.00228	0.002873	0.000353	0.010502	0.007764	0.019806	0.020832
685	-0.00236	0.002833	0.000149	0.010449	0.008053	0.020023	0.021033
684	-0.00201	0.003099	0.000441	0.010699	0.008097	0.020271	0.021562
683	-0.00229	0.00284	0.000353	0.010707	0.008008	0.020184	0.021407
682	-0.00228	0.002827	0.00028	0.010707	0.008081	0.020397	0.021332
681	-0.00212	0.00305	0.000471	0.010949	0.008297	0.020685	0.021734
680	-0.00219	0.002987	0.000441	0.011038	0.008124	0.020587	0.021854
679	-0.00185	0.003157	0.0005	0.011025	0.008543	0.02104	0.022227
678	-0.00221	0.003042	0.000779	0.011357	0.008391	0.020851	0.022187
677	-0.00238	0.003059	0.000476	0.011213	0.0084	0.021009	0.022388
676	-0.00232	0.002998	0.000592	0.011199	0.008603	0.021105	0.02248
675	-0.00231	0.003202	0.000572	0.011509	0.008611	0.021219	0.022782
674	-0.00228	0.003086	0.000505	0.01157	0.008749	0.021456	0.02282
673	-0.00231	0.003035	0.000654	0.011629	0.008822	0.021565	0.022914
672	-0.00239	0.003168	0.000729	0.01166	0.008793	0.021788	0.023168
671	-0.00254	0.003012	0.000555	0.011542	0.00871	0.021881	0.023341
670	-0.00237	0.003174	0.00069	0.011914	0.00905	0.022096	0.023564
669	-0.00225	0.003313	0.000712	0.011948	0.009217	0.022334	0.023657
668	-0.00239	0.003323	0.000777	0.012093	0.009432	0.022639	0.023977
667	-0.00237	0.003341	0.000785	0.012185	0.009459	0.022719	0.024189
666	-0.00238	0.003372	0.000688	0.012157	0.009566	0.022823	0.024299
665	-0.00225	0.003656	0.001052	0.012526	0.009819	0.023009	0.024739
664	-0.0022	0.003504	0.001033	0.012633	0.009913	0.023252	0.024817
663	-0.00218	0.003633	0.001002	0.012754	0.010063	0.023444	0.025057
662	-0.00231	0.003578	0.001078	0.012795	0.010073	0.023696	0.025256
661	-0.0022	0.003634	0.001217	0.013014	0.010345	0.023848	0.025562

EXCLI Journal 2020;19:48-70 – ISSN 1611-2156
Received: October 03, 2019, accepted: December 19, 2019, published: January 03, 2020

Wavelength (nm)	***Absorption intensity***						
	**concentration β-hydroxyisovalerylshikonin (M)**						
	A	*B*	*C*	*D*	*E*	*F*	*G*
	0.00	$4.0x10^{-6}$	$8.0x10^{-6}$	$1.0x10^{-5}$	$1.4x10^{-5}$	$1.6x10^{-5}$	$1.8x10^{-5}$
660	-0.00218	0.003682	0.001058	0.013017	0.010121	0.023974	0.025674
659	-0.00234	0.003599	0.001355	0.013117	0.010403	0.024186	0.025859
658	-0.00221	0.00374	0.0013	0.013286	0.010547	0.024293	0.026052
657	-0.0026	0.003574	0.001148	0.01329	0.010442	0.024537	0.026194
656	-0.00233	0.003951	0.001352	0.013594	0.010752	0.024885	0.02665
655	-0.00222	0.004011	0.001422	0.013844	0.011069	0.025078	0.026907
654	-0.00248	0.003771	0.001321	0.01366	0.011012	0.025261	0.027099
653	-0.00222	0.004096	0.001608	0.013922	0.01114	0.025555	0.027485
652	-0.00234	0.004029	0.001571	0.01401	0.011213	0.025629	0.027653
651	-0.00244	0.003994	0.001538	0.014058	0.0114	0.025815	0.027845
650	-0.00231	0.004102	0.00161	0.0143	0.011665	0.026153	0.028201
649	-0.0023	0.004158	0.001745	0.014453	0.011789	0.0263	0.02847
648	-0.00216	0.004328	0.002008	0.014739	0.011933	0.026777	0.028741
647	-0.00221	0.004248	0.001828	0.014815	0.012033	0.026734	0.028793
646	-0.00234	0.004312	0.001867	0.014816	0.012166	0.026862	0.029117
645	-0.00234	0.004399	0.00194	0.01491	0.012235	0.027312	0.029343
644	-0.00225	0.004606	0.001985	0.015281	0.012528	0.027555	0.029709
643	-0.00225	0.004563	0.002222	0.015547	0.012709	0.027841	0.030015
642	-0.00244	0.004491	0.002011	0.015416	0.012704	0.028121	0.030141
641	-0.0023	0.004532	0.002197	0.015736	0.012929	0.028366	0.030462
640	-0.00228	0.00462	0.002209	0.015903	0.013037	0.028502	0.030787
639	-0.00237	0.0047	0.002197	0.015852	0.01313	0.028765	0.031082
638	-0.00214	0.004913	0.002545	0.016307	0.013556	0.029188	0.031447
637	-0.00243	0.004722	0.002291	0.016052	0.013399	0.029121	0.031672
636	-0.00236	0.004927	0.002472	0.016434	0.013796	0.029532	0.031956
635	-0.00237	0.004718	0.002457	0.016528	0.013762	0.0297	0.032124
634	-0.00231	0.004904	0.002671	0.016736	0.014028	0.029965	0.03247
633	-0.00243	0.004886	0.002538	0.016735	0.014131	0.030233	0.032644
632	-0.00248	0.004985	0.00265	0.0168	0.01417	0.030485	0.033035
631	-0.00243	0.005015	0.002744	0.017124	0.01442	0.030616	0.033187
630	-0.00237	0.005093	0.002785	0.017399	0.014664	0.031025	0.033598
629	-0.00255	0.005132	0.002679	0.017308	0.014586	0.031226	0.03388
628	-0.00257	0.005076	0.002666	0.017496	0.014673	0.031285	0.034009
627	-0.00242	0.005297	0.002938	0.017751	0.015105	0.031797	0.034365
626	-0.00246	0.005192	0.002882	0.017792	0.015165	0.031843	0.034619
625	-0.00235	0.005252	0.002889	0.018028	0.015361	0.032109	0.034986
624	-0.00226	0.005353	0.002991	0.018159	0.015665	0.032439	0.035274
623	-0.00247	0.005345	0.003042	0.018162	0.015513	0.032654	0.035439
622	-0.00229	0.005629	0.003331	0.018572	0.015983	0.032994	0.035967
621	-0.00238	0.005478	0.003225	0.018565	0.015961	0.033107	0.036076
620	-0.0025	0.005601	0.003291	0.018617	0.016124	0.033475	0.036432
619	-0.00242	0.005792	0.0035	0.018946	0.016343	0.033883	0.036815
618	-0.00247	0.005633	0.003353	0.018912	0.016368	0.033925	0.036923
617	-0.00234	0.005876	0.003473	0.01922	0.016517	0.034241	0.037118
616	-0.00235	0.005839	0.003546	0.019315	0.016654	0.034423	0.037586
615	-0.00242	0.005766	0.003574	0.019475	0.016891	0.034827	0.037857
614	-0.00249	0.0058	0.003509	0.019682	0.016996	0.034914	0.038064
613	-0.00237	0.006119	0.003754	0.019865	0.017254	0.035237	0.038465

EXCLI Journal 2020;19:48-70 – ISSN 1611-2156
Received: October 03, 2019, accepted: December 19, 2019, published: January 03, 2020

Wavelength (nm)	***Absorption intensity***						
	**concentration *β*-hydroxyisovalerylshikonin (M)**						
	A	*B*	*C*	*D*	*E*	*F*	*G*
	0.00	$4.0x10^{-6}$	$8.0x10^{-6}$	$1.0x10^{-5}$	$1.4x10^{-5}$	$1.6x10^{-5}$	$1.8x10^{-5}$
612	-0.00231	0.005919	0.003657	0.020009	0.017472	0.035458	0.038797
611	-0.00243	0.006111	0.003882	0.019963	0.017675	0.035668	0.038922
610	-0.0023	0.006032	0.003916	0.020104	0.017771	0.036014	0.039339
609	-0.00241	0.006219	0.003943	0.020432	0.01787	0.03628	0.03968
608	-0.00241	0.006265	0.004025	0.020687	0.018116	0.036602	0.040008
607	-0.00227	0.006405	0.004177	0.020899	0.018528	0.036997	0.040505
606	-0.00234	0.006271	0.004187	0.021081	0.018532	0.037251	0.040761
605	-0.00234	0.00648	0.004323	0.021172	0.018826	0.037508	0.041047
604	-0.00228	0.006598	0.004338	0.021389	0.018917	0.037858	0.041422
603	-0.00236	0.006697	0.004527	0.021678	0.019311	0.038296	0.041811
602	-0.00231	0.006714	0.004432	0.021858	0.01942	0.038537	0.042009
601	-0.00233	0.006838	0.004574	0.02201	0.01959	0.038949	0.042505
600	-0.00241	0.006798	0.004638	0.022378	0.019877	0.039244	0.043016
599	-0.00237	0.007019	0.004679	0.022417	0.020229	0.039556	0.04329
598	-0.00232	0.007	0.004935	0.022789	0.020462	0.039998	0.043809
597	-0.00234	0.007149	0.004934	0.022918	0.020564	0.040497	0.044411
596	-0.0023	0.007142	0.005002	0.023176	0.02089	0.040775	0.044627
595	-0.0024	0.007261	0.005101	0.023423	0.021264	0.041296	0.04507
594	-0.0023	0.007219	0.005275	0.023621	0.021396	0.041644	0.045645
593	-0.0024	0.007196	0.005247	0.02366	0.021633	0.041766	0.045999
592	-0.00236	0.007427	0.005456	0.024152	0.021913	0.042362	0.046456
591	-0.00222	0.007496	0.0055	0.0244	0.022158	0.042745	0.047034
590	-0.00229	0.007585	0.005677	0.024682	0.022589	0.043309	0.04747
589	-0.0025	0.007541	0.005683	0.024676	0.022654	0.043573	0.04797
588	-0.00237	0.007699	0.005666	0.024974	0.022788	0.044037	0.048433
587	-0.0024	0.007807	0.005877	0.025239	0.023256	0.044473	0.048924
586	-0.00252	0.007813	0.005905	0.025543	0.023515	0.04492	0.049237
585	-0.00234	0.007896	0.006057	0.025883	0.023891	0.045325	0.049931
584	-0.00246	0.00794	0.006096	0.026172	0.024091	0.045567	0.050287
583	-0.00244	0.008094	0.006079	0.026109	0.024176	0.045815	0.05053
582	-0.00256	0.008124	0.006292	0.026448	0.024379	0.046225	0.050992
581	-0.00252	0.008079	0.006248	0.026662	0.0245	0.046588	0.051151
580	-0.00262	0.008164	0.006236	0.026646	0.024743	0.046847	0.051279
579	-0.00243	0.00833	0.006469	0.026975	0.025012	0.046992	0.051608
578	-0.00245	0.008441	0.006477	0.027001	0.025091	0.047012	0.051703
577	-0.00237	0.008372	0.006561	0.027122	0.025231	0.047102	0.05197
576	-0.00246	0.00832	0.006585	0.027203	0.025227	0.047372	0.052183
575	-0.00235	0.008464	0.006721	0.027287	0.025534	0.04763	0.052355
574	-0.00258	0.008212	0.006585	0.027331	0.025274	0.047573	0.052254
573	-0.00225	0.008456	0.006656	0.027418	0.025519	0.047749	0.052455
572	-0.00243	0.008318	0.006673	0.027363	0.02544	0.04769	0.052536
571	-0.0025	0.008309	0.006446	0.027303	0.025295	0.047659	0.052309
570	-0.00236	0.008519	0.006673	0.027573	0.025506	0.047803	0.052555
569	-0.00251	0.008338	0.00647	0.027311	0.025375	0.047499	0.052402
568	-0.00248	0.008482	0.00661	0.027539	0.025565	0.048108	0.052955
567	-0.00236	0.008457	0.006625	0.027292	0.025433	0.04809	0.052741
566	-0.00226	0.008452	0.006503	0.027571	0.025309	0.048003	0.052809
565	-0.00243	0.008213	0.00641	0.027329	0.02535	0.047796	0.052717

Received: October 03, 2019, accepted: December 19, 2019, published: January 03, 2020

**Absorption intensity**							
**Wavelength (nm)**	**concentration *β*-hydroxyisovalerylshikonin (M)**						
	A	*B*	*C*	*D*	*E*	*F*	*G*
	0.00	4.0x10^{-6}	8.0x10^{-6}	1.0x10^{-5}	1.4x10^{-5}	1.6x10^{-5}	1.8x10^{-5}
564	-0.00253	0.00809	0.006389	0.027122	0.025033	0.047797	0.052563
563	-0.00249	0.008381	0.006484	0.027366	0.02527	0.047988	0.05265
562	-0.00231	0.00839	0.006553	0.02746	0.025411	0.048029	0.052913
561	-0.00245	0.00834	0.006545	0.027328	0.025322	0.048018	0.052771
560	-0.00242	0.008289	0.006462	0.027391	0.025473	0.048016	0.052834
559	-0.00282	0.007955	0.006145	0.027116	0.024992	0.04778	0.052542
558	-0.00251	0.008325	0.006446	0.027315	0.025371	0.048152	0.052821
557	-0.0025	0.008439	0.006462	0.027599	0.025519	0.04843	0.053188
556	-0.00213	0.008291	0.006425	0.027614	0.025489	0.048175	0.053162
555	-0.00209	0.008385	0.006589	0.027629	0.025705	0.048477	0.053405
554	-0.00245	0.00845	0.006425	0.027712	0.025709	0.048522	0.053299
553	-0.00236	0.008513	0.006534	0.02783	0.025914	0.048816	0.053579
552	-0.0024	0.008437	0.006527	0.027871	0.025946	0.048895	0.053652
551	-0.00246	0.008486	0.006577	0.027939	0.025905	0.049094	0.053781
550	-0.00247	0.008609	0.006594	0.028095	0.02604	0.049187	0.053957
549	-0.00242	0.008636	0.006741	0.028162	0.026234	0.049212	0.054137
548	-0.00251	0.008474	0.006673	0.028163	0.026194	0.049658	0.054441
547	-0.00243	0.008677	0.006668	0.028367	0.02631	0.049543	0.054623
546	-0.00239	0.008644	0.006769	0.028538	0.02653	0.049895	0.054801
545	-0.00225	0.008657	0.006856	0.028601	0.026609	0.050042	0.054859
544	-0.00234	0.008718	0.006784	0.02884	0.02665	0.050121	0.055065
543	-0.00228	0.008782	0.00692	0.028723	0.026825	0.050481	0.05546
542	-0.00237	0.00885	0.006888	0.028893	0.026945	0.050676	0.055562
541	-0.00242	0.008958	0.006919	0.028977	0.02702	0.050741	0.055733
540	-0.00243	0.008833	0.006963	0.029067	0.02708	0.050699	0.055914
539	-0.00223	0.009111	0.007243	0.029335	0.027391	0.051178	0.056216
538	-0.00253	0.008967	0.007009	0.029172	0.027274	0.051034	0.056163
537	-0.00232	0.008967	0.00716	0.029311	0.027474	0.051268	0.056296
536	-0.00238	0.008955	0.00708	0.029435	0.027469	0.051445	0.056459
535	-0.00235	0.009024	0.007152	0.029411	0.027465	0.051492	0.056492
534	-0.0023	0.009055	0.007349	0.02951	0.027537	0.051509	0.056484
533	-0.00247	0.008866	0.006912	0.029326	0.027494	0.051463	0.056366
532	-0.00245	0.009125	0.00704	0.029369	0.027423	0.051423	0.056385
531	-0.00235	0.009091	0.007138	0.029466	0.027566	0.051519	0.056388
530	-0.00241	0.008988	0.006824	0.029208	0.027291	0.051235	0.056043
529	-0.00245	0.009004	0.007001	0.029287	0.027315	0.051251	0.056283
528	-0.00236	0.009008	0.006844	0.029327	0.027076	0.05109	0.055978
527	-0.00243	0.008942	0.006904	0.029021	0.027141	0.050968	0.055924
526	-0.00243	0.009005	0.006931	0.029083	0.026909	0.05107	0.055945
525	-0.00236	0.008857	0.006793	0.028909	0.026788	0.050691	0.055595
524	-0.00255	0.008624	0.0066	0.028684	0.026604	0.050587	0.055273
523	-0.00238	0.00877	0.006617	0.028744	0.026638	0.050483	0.055389
522	-0.00247	0.008605	0.006624	0.028561	0.026326	0.050182	0.054877
521	-0.00241	0.008904	0.006806	0.028558	0.026301	0.050021	0.054742
520	-0.00242	0.008794	0.00664	0.028466	0.02617	0.049962	0.054487
519	-0.00246	0.008592	0.006317	0.028214	0.025851	0.049617	0.054285
518	-0.00213	0.008802	0.006446	0.028297	0.026065	0.049564	0.054238
517	-0.00226	0.008497	0.006217	0.028039	0.025724	0.049384	0.053853

EXCLI Journal 2020;19:48-70 – ISSN 1611-2156
Received: October 03, 2019, accepted: December 19, 2019, published: January 03, 2020

Wavelength (nm)	***Absorption intensity***						
	**concentration β-hydroxyisovalerylshikonin (M)**						
	A	*B*	*C*	*D*	*E*	*F*	*G*
	0.00	$4.0x10^{-6}$	$8.0x10^{-6}$	$1.0x10^{-5}$	$1.4x10^{-5}$	$1.6x10^{-5}$	$1.8x10^{-5}$
516	-0.00197	0.008689	0.006309	0.028136	0.025905	0.049383	0.053916
515	-0.00211	0.008596	0.006213	0.027995	0.025581	0.049189	0.053632
514	-0.00234	0.008584	0.006057	0.02785	0.025527	0.049099	0.053623
513	-0.00257	0.008437	0.006016	0.027681	0.025374	0.048897	0.053429
512	-0.00256	0.008514	0.005953	0.027582	0.025299	0.04864	0.053157
511	-0.00241	0.008377	0.005763	0.027617	0.025091	0.048647	0.05297
510	-0.00261	0.008292	0.005759	0.027458	0.024865	0.048326	0.052874
509	-0.00264	0.008243	0.005703	0.027425	0.024976	0.048342	0.052714
508	-0.00269	0.007988	0.005621	0.027102	0.02464	0.048106	0.052523
507	-0.00286	0.008169	0.005445	0.027165	0.024541	0.048029	0.052386
506	-0.00279	0.008073	0.005577	0.026928	0.024552	0.048037	0.052226
505	-0.00285	0.007847	0.005348	0.026906	0.024339	0.047737	0.051973
504	-0.00284	0.008103	0.005306	0.026796	0.024288	0.047739	0.051974
503	-0.00286	0.007913	0.005397	0.026952	0.024373	0.047657	0.051933
502	-0.00274	0.007926	0.005195	0.026717	0.02418	0.04757	0.051772
501	-0.00275	0.00801	0.005402	0.02671	0.024091	0.047564	0.051737
500	-0.00261	0.008074	0.005285	0.02673	0.02412	0.047422	0.051733
499	-0.00282	0.007774	0.005209	0.026637	0.023954	0.047266	0.051551
498	-0.00261	0.008063	0.00544	0.02675	0.023928	0.04727	0.051478
497	-0.0026	0.008227	0.005243	0.026778	0.024011	0.047163	0.051338
496	-0.00245	0.008144	0.005491	0.026684	0.023974	0.047152	0.051312
495	-0.00249	0.008104	0.005278	0.02658	0.023711	0.047	0.051187
494	-0.00256	0.00804	0.005095	0.026353	0.023564	0.04667	0.050813
493	-0.00216	0.008275	0.005362	0.026512	0.023534	0.04679	0.050754
492	-0.0021	0.00822	0.005064	0.026268	0.023245	0.046422	0.05052
491	-0.00219	0.007934	0.004974	0.026072	0.023162	0.046288	0.050218
490	-0.00219	0.007975	0.005007	0.026215	0.023055	0.045986	0.049893
489	-0.0022	0.007932	0.004996	0.02591	0.022878	0.045856	0.04972
488	-0.00218	0.0079	0.004666	0.025787	0.022641	0.045677	0.04941
487	-0.00226	0.00781	0.0047	0.025535	0.022432	0.045293	0.049032
486	-0.00233	0.008032	0.00487	0.025455	0.022543	0.045367	0.049194
485	-0.00226	0.008073	0.00478	0.02544	0.022372	0.045113	0.048591
484	-0.00242	0.008024	0.004844	0.025153	0.022057	0.044692	0.048383
483	-0.00252	0.0081	0.004825	0.025087	0.021726	0.044637	0.048154
482	-0.00243	0.008071	0.004793	0.024894	0.021764	0.044332	0.047925
481	-0.00242	0.007971	0.004795	0.024819	0.021828	0.044317	0.047739
480	-0.00252	0.007876	0.004678	0.02457	0.021354	0.043941	0.047473
479	-0.00258	0.007942	0.004549	0.024655	0.021293	0.043829	0.04731
478	-0.00242	0.007763	0.004195	0.02451	0.021154	0.043779	0.047
477	-0.00242	0.007465	0.00402	0.024214	0.020842	0.043337	0.046736
476	-0.00214	0.007435	0.004005	0.024304	0.020964	0.043375	0.046583
475	-0.002	0.007573	0.004109	0.024271	0.020773	0.043215	0.046501
474	-0.00212	0.00751	0.00403	0.024121	0.020673	0.042981	0.046428
473	-0.00246	0.007385	0.003713	0.024023	0.020602	0.042897	0.046152
472	-0.00285	0.007135	0.003649	0.023602	0.020092	0.042475	0.045665
471	-0.00285	0.007116	0.003324	0.023362	0.019936	0.042368	0.045582
470	-0.00245	0.007397	0.003638	0.023777	0.020434	0.042511	0.045859
469	-0.00255	0.007377	0.00371	0.023727	0.01997	0.042477	0.045675

EXCLI Journal 2020;19:48-70 – ISSN 1611-2156
Received: October 03, 2019, accepted: December 19, 2019, published: January 03, 2020

Wavelength (nm)	Absorption intensity						
	concentration **β**-hydroxyisovalerylshikonin (M)						
	A	*B*	*C*	*D*	*E*	*F*	*G*
	0.00	$4.0x10^{-6}$	$8.0x10^{-6}$	$1.0x10^{-5}$	$1.4x10^{-5}$	$1.6x10^{-5}$	$1.8x10^{-5}$
468	-0.00255	0.007343	0.003473	0.023607	0.019964	0.042362	0.045573
467	-0.00251	0.00734	0.003455	0.023596	0.019921	0.042227	0.045358
466	-0.00241	0.007438	0.003308	0.023432	0.019853	0.042098	0.045255
465	-0.00245	0.007447	0.003482	0.023471	0.020018	0.042077	0.045239
464	-0.00243	0.007431	0.003494	0.023517	0.019714	0.042028	0.045028
463	-0.00252	0.007351	0.003496	0.02345	0.019593	0.041736	0.044638
462	-0.00261	0.00732	0.00354	0.023416	0.019612	0.041607	0.044552
461	-0.00239	0.007296	0.003312	0.023287	0.019355	0.041504	0.044422
460	-0.00258	0.007208	0.003288	0.023111	0.019378	0.041237	0.044312
459	-0.00253	0.007279	0.003404	0.023131	0.019292	0.041138	0.044326
458	-0.00251	0.007289	0.003243	0.022906	0.019184	0.041554	0.044554
457	-0.00245	0.007345	0.003143	0.022947	0.01926	0.041623	0.044432
456	-0.00256	0.007113	0.003047	0.023063	0.019148	0.041306	0.04427
455	-0.00258	0.00731	0.003151	0.023172	0.019355	0.041423	0.04446
454	-0.00241	0.007409	0.003237	0.023096	0.019142	0.041259	0.044432
453	-0.00249	0.007298	0.003099	0.023002	0.01914	0.04128	0.044334
452	-0.00233	0.007317	0.003092	0.023149	0.01918	0.041637	0.044373
451	-0.00241	0.007392	0.003124	0.023123	0.019105	0.041478	0.044345
450	-0.00244	0.007326	0.003127	0.023183	0.019107	0.041532	0.044555
449	-0.00252	0.007225	0.003133	0.02303	0.019009	0.041457	0.044519
448	-0.00238	0.00732	0.002933	0.0232	0.019209	0.041425	0.044562
447	-0.00239	0.00736	0.003067	0.023305	0.019243	0.041676	0.044567
446	-0.00229	0.007541	0.003098	0.023509	0.019299	0.041891	0.044764
445	-0.00236	0.007216	0.002929	0.023092	0.01931	0.041746	0.044677
444	-0.00242	0.007523	0.003174	0.023453	0.019262	0.042043	0.044908
443	-0.00228	0.007443	0.003105	0.023631	0.019304	0.042086	0.045089
442	-0.00232	0.007472	0.00333	0.023585	0.019675	0.042398	0.045347
441	-0.0024	0.007475	0.003128	0.023565	0.019434	0.042364	0.045463
440	-0.00219	0.007198	0.003077	0.023762	0.019635	0.042668	0.045481
439	-0.00253	0.007301	0.002977	0.023751	0.019565	0.042751	0.045739
438	-0.0024	0.007303	0.00292	0.023759	0.01961	0.042579	0.04575
437	-0.00243	0.007333	0.003032	0.023878	0.019745	0.042865	0.046002
436	-0.00281	0.00716	0.002944	0.02384	0.019655	0.0428	0.046021
435	-0.0028	0.00733	0.002833	0.023919	0.019645	0.042982	0.046094
434	-0.00255	0.007624	0.003202	0.024208	0.019996	0.043396	0.046515
433	-0.00275	0.007454	0.002904	0.024195	0.019848	0.043233	0.046466
432	-0.00269	0.007437	0.003088	0.02436	0.020036	0.043473	0.04663
431	-0.00225	0.007873	0.003497	0.024745	0.0203	0.043963	0.047012
430	-0.00244	0.007742	0.003232	0.024829	0.020485	0.04392	0.047253
429	-0.00246	0.007907	0.003365	0.024796	0.020434	0.04426	0.047247
428	-0.00233	0.007909	0.003523	0.024741	0.020439	0.044353	0.047401
427	-0.00251	0.00764	0.003401	0.02472	0.0203	0.044251	0.047549
426	-0.00237	0.0079	0.003447	0.024861	0.020457	0.044389	0.047514
425	-0.00234	0.008119	0.00356	0.025181	0.020698	0.04458	0.047835
424	-0.00242	0.00781	0.003169	0.02498	0.020366	0.044574	0.047673
423	-0.00229	0.008005	0.003286	0.025168	0.020633	0.044857	0.047889
422	-0.00235	0.008006	0.003419	0.025196	0.020642	0.044847	0.047897
421	-0.00234	0.008216	0.003363	0.025116	0.020617	0.044888	0.048207

EXCLI Journal 2020;19:48-70 – ISSN 1611-2156
Received: October 03, 2019, accepted: December 19, 2019, published: January 03, 2020

Wavelength (nm)	Absorption intensity						
	concentration *β*-hydroxyisovalerylshikonin (M)						
	A	*B*	*C*	*D*	*E*	*F*	*G*
	0.00	$4.0x10^{-6}$	$8.0x10^{-6}$	$1.0x10^{-5}$	$1.4x10^{-5}$	$1.6x10^{-5}$	$1.8x10^{-5}$
420	-0.00238	0.008056	0.003295	0.025315	0.020845	0.044774	0.047797
419	-0.00235	0.008181	0.0035	0.02491	0.020535	0.044992	0.048071
418	-0.00251	0.007875	0.003366	0.025044	0.020316	0.044742	0.047883
417	-0.00242	0.007934	0.003307	0.024999	0.020464	0.044911	0.048103
416	-0.00205	0.008387	0.003873	0.025439	0.020674	0.045176	0.048231
415	-0.00236	0.008082	0.003554	0.025074	0.020396	0.044841	0.048002
414	-0.00172	0.008313	0.003397	0.025293	0.020634	0.045081	0.048277
413	-0.00228	0.008144	0.003192	0.025154	0.020468	0.04488	0.048082
412	-0.00233	0.007815	0.00318	0.024947	0.020218	0.044858	0.048206
411	-0.00247	0.007909	0.003005	0.02503	0.020277	0.044625	0.048103
410	-0.00249	0.007815	0.002925	0.024822	0.020084	0.044783	0.047852
409	-0.00254	0.008186	0.002974	0.025231	0.020298	0.045009	0.048342
408	-0.0023	0.008165	0.003186	0.025262	0.020501	0.045163	0.048439
407	-0.00241	0.008142	0.00323	0.025166	0.020281	0.045351	0.048373
406	-0.00239	0.008141	0.003066	0.025196	0.020332	0.045076	0.048165
405	-0.00225	0.00774	0.002998	0.025227	0.020295	0.044853	0.048006
404	-0.00242	0.008119	0.002941	0.025228	0.020294	0.044829	0.048157
403	-0.00237	0.008235	0.003186	0.025095	0.020202	0.045136	0.048479
402	-0.00236	0.008406	0.003116	0.025634	0.020432	0.045496	0.048832
401	-0.00237	0.00805	0.002803	0.025046	0.020005	0.045179	0.048363
400	-0.0021	0.008246	0.003032	0.025334	0.020517	0.045465	0.048733
399	-0.00228	0.008146	0.002929	0.025154	0.020139	0.045413	0.048513
398	-0.00225	0.008349	0.003013	0.025229	0.020249	0.045398	0.048188
397	-0.00243	0.008092	0.002757	0.025083	0.020064	0.045245	0.048409
396	-0.00257	0.008298	0.002758	0.02518	0.020028	0.045226	0.04834
395	-0.00258	0.00779	0.002936	0.024987	0.019882	0.044975	0.048201
394	-0.00201	0.00791	0.002735	0.025204	0.020049	0.04541	0.04832
393	-0.00267	0.00813	0.002621	0.02509	0.019831	0.045153	0.048321
392	-0.00267	0.007856	0.002154	0.025102	0.019728	0.04493	0.048041
391	-0.00223	0.008133	0.002785	0.025406	0.020285	0.045734	0.048635
390	-0.00228	0.008191	0.002755	0.025546	0.019967	0.045233	0.048525
389	-0.00236	0.008048	0.002759	0.025561	0.020355	0.045622	0.048965
388	-0.00228	0.008358	0.003023	0.025325	0.020128	0.045525	0.048516
387	-0.00201	0.008206	0.002692	0.024954	0.019487	0.045235	0.04858
386	-0.00234	0.008166	0.002773	0.025245	0.01992	0.045105	0.048522
385	-0.00258	0.008155	0.00246	0.025123	0.019732	0.04508	0.048031
384	-0.00229	0.008037	0.002621	0.024869	0.019621	0.045173	0.048381
383	-0.00219	0.008106	0.002546	0.025025	0.019765	0.044958	0.048196
382	-0.00227	0.008384	0.002907	0.025161	0.019931	0.045497	0.048435
381	-0.002	0.008252	0.00265	0.025464	0.019898	0.045617	0.048683
380	-0.00218	0.008482	0.002501	0.025235	0.019568	0.045661	0.048543
379	-0.00251	0.008339	0.002434	0.025208	0.019707	0.045638	0.048759
378	-0.00182	0.009182	0.003175	0.025202	0.019584	0.045755	0.048793
377	-0.00227	0.008224	0.00249	0.025521	0.019976	0.045637	0.048806
376	-0.00333	0.007579	0.001828	0.024885	0.019113	0.045549	0.048321
375	-0.00205	0.008843	0.002815	0.026274	0.020864	0.047395	0.050143
374	-0.0023	0.008722	0.002485	0.025624	0.020112	0.046279	0.049798
373	-0.0025	0.007855	0.002135	0.02485	0.019423	0.045855	0.048411

EXCLI Journal 2020;19:48-70 – ISSN 1611-2156
Received: October 03, 2019, accepted: December 19, 2019, published: January 03, 2020

Wavelength (nm)	Absorption intensity						
	concentration **β**-hydroxyisovalerylshikonin (M)						
	A	*B*	*C*	*D*	*E*	*F*	*G*
	0.00	$4.0x10^{-6}$	$8.0x10^{-6}$	$1.0x10^{-5}$	$1.4x10^{-5}$	$1.6x10^{-5}$	$1.8x10^{-5}$
372	-0.00142	0.009087	0.003079	0.02629	0.020431	0.046476	0.049888
371	-0.00163	0.008775	0.002899	0.02611	0.02044	0.046615	0.05014
370	-0.00198	0.008166	0.002768	0.025337	0.019714	0.046262	0.049369
369	-0.00169	0.010226	0.003553	0.025875	0.019746	0.046524	0.049923
368	-0.00179	0.009201	0.002592	0.026221	0.020417	0.047224	0.050154
367	-0.00208	0.008843	0.002482	0.02576	0.020135	0.047059	0.049657
366	-0.00163	0.008931	0.003113	0.026333	0.020517	0.047373	0.050894
365	-0.0016	0.009388	0.003403	0.027096	0.021001	0.047861	0.051706
364	-0.002	0.010511	0.004125	0.026586	0.02055	0.047566	0.050739
363	-0.00163	0.009009	0.002658	0.026838	0.020746	0.04874	0.052235
362	-0.00014	0.010026	0.003648	0.029267	0.02294	0.048242	0.05177
361	-0.00399	0.008212	0.001333	0.025294	0.019198	0.046507	0.049906
360	-0.00268	0.008633	0.002882	0.026667	0.020559	0.048342	0.052008
359	-0.00146	0.010011	0.003573	0.027445	0.022018	0.049422	0.052901
358	-0.00266	0.011151	0.004982	0.027179	0.021609	0.049455	0.053184
357	-0.00079	0.009071	0.003162	0.028682	0.022628	0.050373	0.054299
356	-0.00425	0.006892	0.001927	0.026127	0.020026	0.047972	0.051351
355	-0.00168	0.009472	0.003929	0.027568	0.021656	0.050309	0.054206
354	-0.00211	0.011075	0.004354	0.026983	0.023127	0.049618	0.053672
353	-0.00289	0.009489	0.001596	0.02803	0.02293	0.051218	0.055045
352	-0.00233	0.007479	0.000638	0.027112	0.022769	0.047454	0.051268
351	0.000134	0.011588	0.005691	0.029643	0.02384	0.052323	0.056651
350	-0.00101	0.012317	0.004059	0.029608	0.023284	0.053323	0.055644
349	-0.00278	0.009532	0.002494	0.028246	0.025648	0.055688	0.057251
348	-0.00519	0.008333	0.000162	0.027604	0.020388	0.049572	0.053016
347	-0.00558	0.008276	0.000865	0.027978	0.020356	0.050086	0.054031
346	-0.00481	0.00932	0.001929	0.029103	0.021614	0.051104	0.054652
345	-0.00511	0.008643	0.000683	0.028276	0.021501	0.050775	0.054532
344	-0.00459	0.009193	0.001323	0.029414	0.021521	0.05189	0.055325
343	-0.00529	0.00827	0.001036	0.028863	0.021759	0.051487	0.055769
342	-0.0052	0.009418	0.001589	0.030051	0.022036	0.052297	0.056378
341	-0.00539	0.009017	0.001748	0.029741	0.022029	0.052789	0.057133
340	-0.00575	0.008993	0.001001	0.029555	0.02224	0.052843	0.056491
339	-0.0051	0.009382	0.002004	0.030094	0.023069	0.053841	0.058299
338	-0.00434	0.009642	0.002356	0.031156	0.023817	0.055027	0.058598
337	-0.00483	0.010028	0.001811	0.030638	0.023645	0.054532	0.059127
336	-0.00473	0.009394	0.002181	0.031742	0.023828	0.055465	0.06032
335	-0.00492	0.009553	0.001621	0.031168	0.024015	0.055551	0.060703
334	-0.00453	0.010446	0.002001	0.032137	0.024781	0.056507	0.061139
333	-0.00419	0.010381	0.00257	0.032134	0.025283	0.057405	0.062096
332	-0.00456	0.010585	0.002548	0.032582	0.025338	0.057297	0.062407
331	-0.00458	0.0108	0.002617	0.033092	0.025566	0.05775	0.06293
330	-0.00448	0.010244	0.002131	0.03311	0.025689	0.057879	0.062947
329	-0.00428	0.010671	0.002819	0.034506	0.026385	0.059032	0.064388
328	-0.00393	0.011618	0.003827	0.034385	0.027088	0.060109	0.065141
327	-0.00413	0.011212	0.002675	0.03413	0.027103	0.059739	0.065442
326	-0.00444	0.010826	0.002869	0.033848	0.026788	0.060635	0.065707
325	-0.00359	0.011465	0.003587	0.034643	0.028001	0.061322	0.066988

EXCLI Journal 2020;19:48-70 – ISSN 1611-2156
Received: October 03, 2019, accepted: December 19, 2019, published: January 03, 2020

Wavelength (nm)	***Absorption intensity***						
	**concentration *β*-hydroxyisovalerylshikonin (M)**						
	A	*B*	*C*	*D*	*E*	*F*	*G*
	0.00	$4.0x10^{-6}$	$8.0x10^{-6}$	$1.0x10^{-5}$	$1.4x10^{-5}$	$1.6x10^{-5}$	$1.8x10^{-5}$
324	-0.00428	0.011195	0.003586	0.03554	0.027874	0.061909	0.067258
323	-0.00387	0.011947	0.003839	0.036009	0.028741	0.062221	0.068115
322	-0.00326	0.012385	0.004034	0.035923	0.029049	0.06381	0.069499
321	-0.00381	0.011662	0.003672	0.03648	0.029157	0.063341	0.069735
320	-0.00311	0.01254	0.004084	0.037381	0.029884	0.064852	0.070966
319	-0.00306	0.012264	0.004123	0.037298	0.030294	0.065294	0.071713
318	-0.00304	0.012554	0.00441	0.037656	0.030445	0.065801	0.072204
317	-0.003	0.012245	0.003809	0.037417	0.030437	0.065713	0.072817
316	-0.00355	0.012665	0.004081	0.037937	0.030935	0.066876	0.073709
315	-0.00263	0.013015	0.004703	0.038857	0.031688	0.067943	0.074709
314	-0.00221	0.013593	0.004961	0.039615	0.032624	0.069181	0.075917
313	-0.00276	0.013699	0.00484	0.039953	0.032508	0.06922	0.076494
312	-0.00237	0.013831	0.004992	0.040181	0.032616	0.069742	0.077159
311	-0.00205	0.013785	0.00509	0.040645	0.033434	0.070561	0.07772
310	-0.00228	0.013982	0.005441	0.041075	0.033751	0.07106	0.07861
309	-0.00206	0.014105	0.005317	0.041303	0.034356	0.071685	0.079562
308	-0.00202	0.01436	0.005694	0.041934	0.034732	0.072596	0.080326
307	-0.00186	0.015198	0.00596	0.041903	0.035025	0.073371	0.081323
306	-0.00211	0.015493	0.006086	0.042937	0.035891	0.074067	0.082186
305	-0.00161	0.015288	0.006174	0.043357	0.035649	0.07469	0.08329
304	-0.00087	0.016247	0.007247	0.044348	0.037252	0.076857	0.084564
303	-0.00059	0.016289	0.007856	0.044567	0.037375	0.076629	0.085348
302	0.000218	0.017149	0.008021	0.04516	0.038405	0.078219	0.086471
301	-3.10E-05	0.017511	0.00843	0.045947	0.038787	0.07876	0.08795
300	0.000913	0.018745	0.009834	0.047221	0.04031	0.080477	0.089893
299	0.001784	0.019496	0.010479	0.048187	0.040848	0.081712	0.090868
298	0.002942	0.020783	0.011634	0.04969	0.042633	0.083445	0.092443
297	0.004113	0.02189	0.01327	0.050536	0.043868	0.08521	0.094444
296	0.00545	0.023942	0.015066	0.052667	0.045874	0.087564	0.096836
295	0.00727	0.025342	0.016842	0.054285	0.04777	0.089628	0.099782
294	0.00925	0.027584	0.019005	0.056486	0.049839	0.091911	0.102372
293	0.01117	0.030039	0.022003	0.05909	0.052581	0.094883	0.105822
292	0.013862	0.032548	0.025129	0.061581	0.055183	0.097886	0.108967
291	0.016338	0.03532	0.027999	0.064276	0.058482	0.100718	0.112473
290	0.018448	0.038154	0.031331	0.066698	0.061187	0.104289	0.115822
289	0.021878	0.041593	0.035149	0.069854	0.064347	0.107282	0.120156
288	0.024238	0.043986	0.037776	0.071679	0.067138	0.110304	0.123069
287	0.027943	0.048453	0.042312	0.076186	0.071271	0.114512	0.127871
286	0.030726	0.051271	0.045828	0.078563	0.074293	0.118286	0.131535
285	0.034348	0.055232	0.049833	0.082258	0.078205	0.122231	0.13569
284	0.036704	0.058248	0.053508	0.084958	0.081323	0.125555	0.139194
283	0.040967	0.062126	0.058021	0.088807	0.08539	0.12985	0.144252
282	0.044302	0.065758	0.062021	0.092172	0.089041	0.133482	0.148615
281	0.047006	0.068933	0.065506	0.094492	0.092264	0.136915	0.152167
280	0.049764	0.072247	0.069291	0.097976	0.095683	0.140331	0.155991
279	0.053461	0.075604	0.073246	0.100991	0.099016	0.144103	0.160158
278	0.055833	0.078701	0.07647	0.1037	0.10155	0.147451	0.163651
277	0.058592	0.081652	0.080014	0.106715	0.104652	0.15043	0.166884

EXCLI Journal 2020;19:48-70 – ISSN 1611-2156
Received: October 03, 2019, accepted: December 19, 2019, published: January 03, 2020

Wavelength (nm)	***Absorption intensity***						
	**concentration *β*-hydroxyisovalerylshikonin (M)**						
	A	*B*	*C*	*D*	*E*	*F*	*G*
	0.00	$4.0x10^{-6}$	$8.0x10^{-6}$	$1.0x10^{-5}$	$1.4x10^{-5}$	$1.6x10^{-5}$	$1.8x10^{-5}$
276	0.061392	0.084915	0.083731	0.109595	0.10784	0.153977	0.17058
275	0.064487	0.087953	0.087202	0.112704	0.111265	0.157048	0.174572
274	0.067152	0.091051	0.090268	0.115554	0.113999	0.160026	0.177783
273	0.070379	0.094241	0.093863	0.118082	0.117183	0.163367	0.182277
272	0.072816	0.096886	0.097028	0.12109	0.119993	0.166849	0.185454
271	0.075495	0.099892	0.1004	0.123575	0.12305	0.169841	0.188561
270	0.077832	0.102447	0.103277	0.126041	0.125957	0.172737	0.192265
269	0.080519	0.105608	0.106782	0.128728	0.128675	0.175549	0.194769
268	0.082937	0.107907	0.109377	0.131026	0.130947	0.178142	0.198005
267	0.08531	0.110258	0.111614	0.133468	0.133407	0.18086	0.200871
266	0.086812	0.112412	0.114221	0.134989	0.135825	0.182975	0.203504
265	0.089228	0.114798	0.117222	0.137224	0.138264	0.185911	0.206231
264	0.090958	0.116657	0.119212	0.139067	0.140223	0.187886	0.208658
263	0.092668	0.118754	0.121816	0.14105	0.142116	0.189838	0.211056
262	0.094166	0.120384	0.123426	0.142627	0.143856	0.192045	0.2134
261	0.09525	0.121522	0.124988	0.143956	0.145588	0.193344	0.215274
260	0.096505	0.122989	0.126373	0.14532	0.146882	0.195009	0.217329
259	0.097337	0.12369	0.127188	0.146128	0.147844	0.196125	0.21781
258	0.097545	0.123832	0.127127	0.146378	0.148073	0.196597	0.218799
257	0.097359	0.124287	0.12702	0.14692	0.148296	0.197016	0.219132
256	0.097161	0.123632	0.126646	0.146515	0.148086	0.197204	0.219043
255	0.096429	0.122878	0.125469	0.146157	0.147368	0.196925	0.21898
254	0.095511	0.122259	0.124471	0.145673	0.146645	0.19618	0.218441
253	0.093743	0.120354	0.122757	0.144446	0.145361	0.195351	0.217225
252	0.092228	0.118716	0.120795	0.143794	0.143862	0.194282	0.216364
251	0.090784	0.117172	0.118548	0.142601	0.142488	0.193324	0.215196
250	0.088173	0.114709	0.115495	0.140597	0.140341	0.191286	0.212985
249	0.085887	0.111996	0.112401	0.138545	0.138094	0.189323	0.210736
248	0.083333	0.109533	0.10913	0.136667	0.135409	0.187667	0.208868
247	0.080622	0.106478	0.105926	0.134192	0.132906	0.185115	0.20637
246	0.077512	0.103361	0.101978	0.131928	0.130067	0.182661	0.203476
245	0.074406	0.100591	0.098464	0.129359	0.127212	0.180312	0.201173
244	0.071717	0.097417	0.094726	0.12729	0.125057	0.178179	0.198731
243	0.068921	0.094395	0.091059	0.124969	0.122584	0.176155	0.196737
242	0.065782	0.091309	0.087079	0.122587	0.119995	0.174004	0.194586
241	0.063346	0.08857	0.084072	0.120814	0.118363	0.172771	0.19293
240	0.059981	0.085704	0.080516	0.119092	0.11601	0.17101	0.191395
239	0.057943	0.083452	0.078011	0.118335	0.114824	0.170621	0.190801
238	0.055584	0.081637	0.075698	0.117678	0.114446	0.170687	0.191119
237	0.053574	0.080054	0.073528	0.118139	0.114761	0.172258	0.192434
236	0.051933	0.079241	0.072514	0.119502	0.116388	0.175035	0.195485
235	0.05052	0.078988	0.072099	0.122169	0.119632	0.179909	0.200767
234	0.049093	0.079504	0.072948	0.126674	0.124917	0.187165	0.208772
233	0.048102	0.081363	0.074958	0.133767	0.133272	0.19762	0.219794
232	0.047873	0.084599	0.07914	0.144275	0.145357	0.213086	0.236604
231	0.047391	0.088685	0.085015	0.158586	0.162342	0.233701	0.258893
230	0.047858	0.095528	0.094418	0.17919	0.18612	0.261794	0.289733
229	0.048451	0.104372	0.10705	0.205975	0.21801	0.299557	0.330676

EXCLI Journal 2020;19:48-70 – ISSN 1611-2156
Received: October 03, 2019, accepted: December 19, 2019, published: January 03, 2020

Wavelength (nm)	***Absorption intensity***						
	**concentration β-hydroxyisovalerylshikonin (M)**						
	A	*B*	*C*	*D*	*E*	*F*	*G*
	0.00	$4.0x10^{-6}$	$8.0x10^{-6}$	$1.0x10^{-5}$	$1.4x10^{-5}$	$1.6x10^{-5}$	$1.8x10^{-5}$
228	0.049407	0.116019	0.124003	0.241727	0.260507	0.348888	0.38474
227	0.050614	0.131448	0.146426	0.288965	0.316121	0.413384	0.455136
226	0.052243	0.151927	0.176114	0.349481	0.388727	0.496592	0.546016
225	0.054389	0.177137	0.213541	0.42673	0.479301	0.601094	0.660418
224	0.057025	0.2097	0.261545	0.524754	0.595328	0.734287	0.804975
223	0.060047	0.249824	0.321135	0.645689	0.737778	0.897001	0.982124
222	0.062965	0.299224	0.394028	0.793628	0.91199	1.09644	1.199152
221	0.067167	0.357974	0.481203	0.970681	1.120063	1.333554	1.457956
220	0.071357	0.426699	0.584162	1.179443	1.365928	1.612998	1.761898
219	0.076636	0.506194	0.703274	1.419238	1.647673	1.932248	2.10843
218	0.081748	0.596957	0.838572	1.691376	1.969051	2.295938	2.498016
217	0.08672	0.69556	0.986532	1.991877	2.321642	2.692916	2.921738
216	0.092059	0.803134	1.148405	2.322446	2.7013	3.112355	3.371014
215	0.097179	0.914722	1.316286	2.66003	3.085425	3.534543	3.781313
214	0.103344	1.029524	1.490482	3.006719	3.472329	3.918766	4.140491
213	0.109023	1.140923	1.660606	3.359817	3.813851	4.228432	4.44397
212	0.11476	1.247122	1.819968	3.652622	4.131406	4.471111	4.815253
211	0.12031	1.344069	1.970323	3.88075	4.40679	4.655041	4.714869
210	0.125045	1.419807	2.086514	4.099201	4.455711	4.879216	4.823324
209	0.131481	1.482833	2.180276	4.280365	4.530156	4.796384	4.76187
208	0.137277	1.524212	2.240155	4.373894	4.541652	4.8181	4.782251
207	0.142533	1.547335	2.272936	4.405063	4.590304	4.737175	4.833741
206	0.148924	1.553804	2.276489	4.400499	4.567488	4.641325	4.766871
205	0.157314	1.546727	2.262802	4.290354	4.515654	4.849509	4.9735
204	0.160151	1.528833	2.219108	4.191915	4.33968	4.503073	4.535141
203	0.167487	1.515464	2.187293	4.063804	4.119167	4.162218	4.394517
202	0.175696	1.495549	2.145277	3.716444	3.826278	3.931505	3.877328
201	0.172119	1.476098	2.083886	3.362265	3.41792	3.492174	3.502906
200	0.169385	1.469421	1.981929	2.815927	2.837536	2.864536	2.874966

EXCLI Journal 2020;19:48-70 – ISSN 1611-2156
Received: October 03, 2019, accepted: December 19, 2019, published: January 03, 2020

Raw tables concerning **Figure 7.** The fluorescence emission spectra of DNA-EB fixed concentration (DNA (1.72 x 10^{-5} M) and EB (1.2 x 10^{-5} M)), in the absence and presence of increasing concentration of α-methylbutyrylshikon (***1***), acetylshikonin (***2***) and *β*-hydroxyisovalerylshikonin (***3***) (from 0 to 2.4 x 10^{-5} M). Arrow shows the intensity change upon the increase of the naphthoquinone concentration. Purple dashed line represents the emission spectra of ***1***, ***2*** and ***3*** in the absence of DNA-EB.

Table 1: Emission intensity in the wavelength range of 550-700 nm for fluorescence emission spectra of DNA-EB fixed concentration (DNA (1.72 x 10^{-5} M) and EB (1.2 x 10^{-5} M)), in the absence (*A*) and presence of increasing concentration of α-methylbutyrylshikon (*B-H*), and emission intensity of α-methylbutyrylshikon in the absence of DNA-EB (*J*)

Wavelength (nm)	***Emission intensity***								
	**concentration of α-methylbutyrylshikon (M)**								
	A	*B*	*C*	*D*	*E*	*F*	*G*	*H*	*J*
	0.00	4.0x10^{-6}	8.0x10^{-6}	1.0x10^{-5}	1.4x10^{-5}	1.6x10^{-5}	2.0x10^{-5}	2.4x10^{-5}	2.4x10^{-5}
550	2.102	2.085	2.295	2.274	2.238	2.344	2.426	2.116	0.773
551	2.205	2.159	2.352	2.331	2.312	2.397	2.487	2.158	0.767
552	2.447	2.37	2.568	2.538	2.506	2.563	2.658	2.318	0.763
553	2.732	2.628	2.837	2.797	2.738	2.772	2.859	2.52	0.768
554	3.052	2.928	3.125	3.056	2.983	3.011	3.097	2.727	0.765
555	3.413	3.245	3.451	3.381	3.29	3.287	3.38	2.987	0.772
556	3.836	3.627	3.841	3.742	3.627	3.619	3.697	3.274	0.775
557	4.272	4.057	4.266	4.138	4.001	3.973	4.057	3.596	0.785
558	4.815	4.533	4.756	4.613	4.429	4.362	4.455	3.958	0.787
559	5.393	5.07	5.292	5.136	4.915	4.822	4.907	4.363	0.788
560	6.013	5.647	5.89	5.701	5.449	5.314	5.406	4.818	0.805
561	6.736	6.332	6.56	6.331	6.053	5.886	5.944	5.325	0.815
562	7.511	7.054	7.286	7.033	6.705	6.497	6.565	5.862	0.821
563	8.37	7.865	8.107	7.818	7.434	7.186	7.261	6.467	0.838
564	9.322	8.75	9.026	8.663	8.251	7.943	8.005	7.144	0.848
565	10.281	9.704	10.001	9.592	9.087	8.74	8.791	7.858	0.852
566	11.402	10.706	11.065	10.595	10.033	9.629	9.674	8.617	0.873
567	12.577	11.857	12.159	11.646	11.028	10.577	10.645	9.452	0.879
568	13.821	13.059	13.413	12.799	12.113	11.591	11.664	10.346	0.885
569	15.263	14.373	14.748	14.074	13.292	12.734	12.776	11.343	0.9
570	16.674	15.795	16.172	15.413	14.513	13.901	13.931	12.363	0.912
571	18.239	17.321	17.698	16.875	15.953	15.176	15.213	13.507	0.916
572	19.944	18.929	19.353	18.429	17.396	16.596	16.602	14.705	0.923
573	21.675	20.609	21.057	20.036	18.875	17.999	18.051	15.988	0.937
574	23.586	22.41	22.851	21.759	20.531	19.514	19.556	17.23	0.934
575	25.511	24.321	24.805	23.54	22.176	21.152	21.122	18.639	0.95
576	27.529	26.235	26.757	25.412	23.877	22.756	22.764	20.085	0.952
577	29.649	28.305	28.809	27.404	25.769	24.467	24.472	21.577	0.96
578	31.865	30.37	31.038	29.335	27.713	26.261	26.215	23.147	0.956
579	34.114	32.56	33.249	31.485	29.657	28.165	28.115	24.771	0.967
580	36.373	34.868	35.569	33.71	31.662	30.125	30.067	26.419	0.968
581	38.766	37.119	37.921	35.834	33.769	32.03	31.994	28.069	0.974
582	41.207	39.581	40.231	38.116	35.916	33.986	33.952	29.897	0.982
583	43.639	41.95	42.743	40.419	38.05	36.06	35.975	31.628	0.976
584	45.967	44.282	45.082	42.616	40.045	37.962	37.967	33.351	0.988

EXCLI Journal 2020;19:48-70 – ISSN 1611-2156
Received: October 03, 2019, accepted: December 19, 2019, published: January 03, 2020

Wavelength (nm)	Emission intensity								
	concentration of α-methylbutyrylshikon (M)								
	A	*B*	*C*	*D*	*E*	*F*	*G*	*H*	*J*
	0.00	$4.0x10^{-6}$	$8.0x10^{-6}$	$1.0x10^{-5}$	$1.4x10^{-5}$	$1.6x10^{-5}$	$2.0x10^{-5}$	$2.4x10^{-5}$	$2.4x10^{-5}$
585	48.31	46.542	47.418	44.813	42.062	39.877	39.894	35.046	1.002
586	50.665	48.799	49.67	47.009	44.178	41.782	41.789	36.676	0.991
587	52.979	50.925	51.853	49.225	46.074	43.732	43.696	38.44	0.999
588	55.318	53.188	54.179	51.276	48.161	45.655	45.606	40.054	1.001
589	57.429	55.3	56.391	53.397	50.059	47.427	47.48	41.603	1.006
590	59.506	57.368	58.55	55.413	51.976	49.216	49.306	43.112	1.007
591	61.684	59.335	60.502	57.358	53.8	50.957	50.957	44.67	1.008
592	63.521	61.258	62.469	59.173	55.491	52.507	52.509	46.057	1.008
593	65.329	63.089	64.315	60.964	57.07	54.071	54.104	47.366	1.008
594	67.345	64.804	65.995	62.45	58.764	55.552	55.578	48.689	1.012
595	68.846	66.395	67.803	64.106	60.186	56.895	56.965	49.942	1.004
596	70.277	67.966	69.411	65.689	61.533	58.163	58.301	51.059	1.006
597	71.775	69.242	70.611	66.885	62.905	59.34	59.513	52.088	1.005
598	73.207	70.654	72.224	68.478	64.235	60.652	60.82	53.306	0.992
599	74.466	71.751	73.291	69.505	65.153	61.596	61.706	54.038	0.997
600	75.455	72.793	74.382	70.532	66.123	62.556	62.57	54.891	0.987
601	76.331	73.633	75.336	71.421	66.912	63.36	63.474	55.663	0.99
602	77.235	74.401	76.03	72.186	67.672	64.044	64.019	56.292	0.983
603	77.927	74.942	76.812	72.826	68.243	64.487	64.798	56.863	0.978
604	78.619	75.439	77.333	73.453	68.824	65.006	65.299	57.23	0.973
605	79.064	76.114	77.626	73.987	69.303	65.585	65.76	57.631	0.965
606	79.488	76.608	78.156	74.322	69.548	65.866	65.972	57.983	0.959
607	79.694	76.815	78.488	74.703	69.663	66.082	66.343	58.297	0.958
608	79.906	76.986	78.547	74.806	70.036	66.301	66.599	58.376	0.95
609	80.074	76.828	78.619	74.758	70.18	66.237	66.6	58.624	0.944
610	80.084	76.833	78.642	74.849	70.091	66.328	66.743	58.666	0.938
611	79.912	76.707	78.633	74.652	70.002	66.299	66.694	58.571	0.935
612	79.713	76.441	78.458	74.471	69.895	66.071	66.545	58.549	0.929
613	79.458	76.114	77.991	74.308	69.593	65.801	66.417	58.351	0.925
614	79.113	75.538	77.578	73.841	69.283	65.558	65.937	58.104	0.917
615	78.574	75.222	77.125	73.487	68.94	65.129	65.442	57.789	0.913
616	78.011	74.675	76.516	72.841	68.516	64.628	65.215	57.397	0.909
617	77.277	73.892	75.826	72.442	67.859	64.088	64.608	57.088	0.903
618	76.754	73.317	74.854	71.764	67.279	63.623	64.087	56.591	0.896
619	75.978	72.474	74.219	70.935	66.611	62.899	63.328	56.029	0.885
620	75.019	71.542	73.383	70.262	65.738	62.151	62.814	55.507	0.891
621	74.237	70.626	72.524	69.273	64.973	61.439	61.997	54.84	0.878
622	73.261	69.676	71.591	68.241	64.157	60.566	61.1	54.275	0.874
623	72.299	68.536	70.499	67.304	63.119	59.726	60.274	53.561	0.872
624	71.183	67.482	69.332	66.172	62.161	58.763	59.296	52.79	0.86
625	69.902	66.246	68.169	65.163	61.268	57.885	58.283	51.979	0.845
626	68.672	65.017	66.891	64.007	60.191	56.819	57.379	51.123	0.845
627	67.43	63.723	65.504	62.888	58.951	55.668	56.386	50.171	0.83
628	65.812	62.209	63.804	61.327	57.521	54.483	55.046	48.984	0.817
629	64.445	60.867	62.572	60.073	56.502	53.257	53.801	48.142	0.8
630	63.043	59.503	61.168	58.869	55.189	52.106	52.612	47.283	0.788
631	61.646	58.032	59.806	57.471	53.915	51.031	51.486	46.211	0.772
632	60.271	56.7	58.365	56.089	52.585	49.787	50.247	45.247	0.766

Wavelength (nm)	Emission intensity								
	concentration of α-methylbutyrylshikon (M)								
	A	*B*	*C*	*D*	*E*	*F*	*G*	*H*	*J*
	0.00	$4.0x10^{-6}$	$8.0x10^{-6}$	$1.0x10^{-5}$	$1.4x10^{-5}$	$1.6x10^{-5}$	$2.0x10^{-5}$	$2.4x10^{-5}$	$2.4x10^{-5}$
633	58.802	55.277	56.939	54.72	51.345	48.572	49.19	44.189	0.75
634	57.347	53.8	55.568	53.299	50.116	47.396	47.87	43.137	0.74
635	55.979	52.388	53.988	51.945	48.893	46.157	46.674	42.15	0.718
636	54.486	50.95	52.427	50.664	47.59	44.967	45.547	41.112	0.707
637	52.999	49.508	51.13	49.352	46.298	43.809	44.28	40.1	0.689
638	51.681	48.308	49.813	48.036	45.127	42.628	43.187	39.059	0.675
639	50.404	46.888	48.471	46.705	43.913	41.4	42.119	38.032	0.666
640	49.049	45.582	47.171	45.482	42.747	40.388	40.974	37.176	0.652
641	47.7	44.452	45.902	44.292	41.55	39.34	39.902	36.155	0.637
642	46.454	43.212	44.635	42.992	40.485	38.263	38.79	35.294	0.626
643	44.955	41.821	43.197	41.695	39.255	37.105	37.691	34.197	0.618
644	43.808	40.652	41.96	40.612	38.177	36.064	36.693	33.31	0.605
645	42.651	39.52	40.913	39.515	37.173	35.129	35.702	32.52	0.601
646	41.572	38.489	39.883	38.47	36.253	34.233	34.717	31.811	0.59
647	40.517	37.404	38.741	37.433	35.221	33.245	33.871	30.987	0.582
648	39.386	36.417	37.713	36.493	34.301	32.381	32.988	30.159	0.565
649	38.407	35.345	36.659	35.478	33.37	31.541	32.061	29.397	0.566
650	37.338	34.35	35.559	34.49	32.454	30.673	31.217	28.598	0.556
651	36.275	33.473	34.639	33.486	31.598	29.803	30.284	27.844	0.538
652	35.311	32.452	33.617	32.606	30.699	28.948	29.495	27.091	0.537
653	34.334	31.44	32.657	31.636	29.842	28.1	28.665	26.362	0.527
654	33.42	30.595	31.754	30.803	28.987	27.323	27.867	25.614	0.517
655	32.413	29.634	30.782	29.903	28.145	26.583	27.063	24.865	0.504
656	31.439	28.744	29.901	29.016	27.313	25.795	26.289	24.201	0.503
657	30.519	27.928	29.006	28.133	26.547	25.002	25.54	23.604	0.493
658	29.453	26.867	27.9	27.089	25.569	24.146	24.628	22.773	0.476
659	28.547	26.092	27.082	26.252	24.807	23.41	23.861	22.046	0.47
660	27.676	25.272	26.278	25.478	23.991	22.676	23.177	21.39	0.467
661	26.79	24.467	25.329	24.664	23.241	21.926	22.459	20.708	0.45
662	25.974	23.661	24.542	23.84	22.553	21.189	21.716	20.104	0.445
663	25.153	22.857	23.7	23.062	21.812	20.552	21.05	19.471	0.432
664	24.329	22.08	22.933	22.336	21.096	19.914	20.356	18.88	0.421
665	23.574	21.369	22.168	21.648	20.403	19.23	19.678	18.294	0.412
666	22.746	20.627	21.397	20.849	19.679	18.566	19.022	17.664	0.408
667	21.947	19.88	20.653	20.176	18.984	17.949	18.339	17.052	0.396
668	21.161	19.119	19.865	19.423	18.345	17.315	17.683	16.458	0.383
669	20.37	18.391	19.166	18.712	17.652	16.659	17.064	15.878	0.375
670	19.642	17.713	18.435	17.994	17.021	16.007	16.406	15.319	0.365
671	18.916	17.042	17.729	17.31	16.326	15.414	15.797	14.734	0.358
672	18.168	16.367	17.059	16.636	15.733	14.841	15.172	14.173	0.343
673	17.32	15.571	16.244	15.873	14.979	14.146	14.461	13.524	0.335
674	16.585	14.937	15.584	15.237	14.376	13.583	13.897	12.996	0.32
675	15.948	14.33	14.896	14.626	13.755	13.02	13.312	12.482	0.309
676	15.301	13.695	14.304	14.011	13.185	12.471	12.781	11.987	0.304
677	14.667	13.13	13.709	13.412	12.63	11.965	12.221	11.473	0.304
678	14.052	12.587	13.118	12.849	12.12	11.457	11.688	10.988	0.283
679	13.475	12.042	12.587	12.299	11.607	10.939	11.226	10.554	0.281
680	12.852	11.497	12.02	11.784	11.102	10.483	10.782	10.096	0.269

EXCLI Journal 2020;19:48-70 – ISSN 1611-2156
Received: October 03, 2019, accepted: December 19, 2019, published: January 03, 2020

Wavelength (nm)	***Emission intensity***								
	**concentration of α-methylbutyrylshikon (M)**								
	A	*B*	*C*	*D*	*E*	*F*	*G*	*H*	*J*
	0.00	$4.0x10^{-6}$	$8.0x10^{-6}$	$1.0x10^{-5}$	$1.4x10^{-5}$	$1.6x10^{-5}$	$2.0x10^{-5}$	$2.4x10^{-5}$	$2.4x10^{-5}$
681	12.313	10.997	11.49	11.299	10.605	10.006	10.265	9.68	0.267
682	11.757	10.489	10.946	10.763	10.144	9.56	9.818	9.242	0.259
683	11.22	10.015	10.46	10.255	9.702	9.128	9.375	8.831	0.248
684	10.613	9.47	9.886	9.712	9.176	8.654	8.881	8.342	0.234
685	10.135	9.02	9.41	9.255	8.737	8.235	8.465	7.964	0.233
686	9.65	8.597	8.986	8.801	8.341	7.864	8.068	7.6	0.227
687	9.24	8.177	8.564	8.415	7.943	7.514	7.701	7.275	0.219
688	8.812	7.802	8.149	8.006	7.567	7.153	7.33	6.942	0.212
689	8.393	7.45	7.796	7.642	7.235	6.824	7.01	6.622	0.209
690	7.999	7.098	7.426	7.305	6.897	6.487	6.695	6.324	0.198
691	7.633	6.764	7.055	6.945	6.576	6.183	6.352	6.022	0.193
692	7.286	6.453	6.734	6.629	6.272	5.893	6.081	5.746	0.194
693	6.86	6.052	6.369	6.263	5.912	5.575	5.723	5.426	0.187
694	6.551	5.758	6.04	5.96	5.618	5.314	5.463	5.182	0.184
695	6.24	5.494	5.772	5.692	5.363	5.06	5.2	4.931	0.18
696	5.93	5.228	5.494	5.394	5.108	4.813	4.935	4.712	0.178
697	5.652	4.987	5.227	5.141	4.848	4.578	4.698	4.474	0.17
698	5.382	4.739	4.968	4.886	4.619	4.365	4.486	4.27	0.162
699	5.122	4.5	4.72	4.66	4.405	4.145	4.273	4.073	0.163
700	4.84	4.26	4.465	4.393	4.155	3.919	4.033	3.843	0.157

EXCLI Journal 2020;19:48-70 – ISSN 1611-2156
Received: October 03, 2019, accepted: December 19, 2019, published: January 03, 2020

Table 2: Emission intensity in the wavelength range of 550-700 nm for fluorescence emission spectra of DNA-EB fixed concentration (DNA (1.72 x 10^{-5} M) and EB (1.2 x 10^{-5} M)), in the absence (*A*) and presence of increasing concentration of acetylshikonin (*B-H*), and emission intensity of acetylshikonin in the absence of DNA-EB (*J*)

**Wavelength (nm)**	***Emission intensity***								
	**concentration of acetylshikonin (M)**								
	A	*B*	*C*	*D*	*E*	*F*	*G*	*H*	*J*
	0.00	4.0x10^{-6}	8.0x10^{-6}	1.0x10^{-5}	1.4x10^{-5}	1.6x10^{-5}	2.0x10^{-5}	2.4x10^{-5}	2.4x10^{-5}
550	2.122	2.351	2.809	2.458	3.088	3.052	2.785	2.57	0.773
551	2.2	2.436	2.885	2.524	3.141	3.089	2.852	2.615	0.767
552	2.458	2.661	3.09	2.729	3.318	3.282	3.006	2.808	0.763
553	2.745	2.925	3.336	2.978	3.55	3.497	3.216	3.021	0.768
554	3.069	3.21	3.6	3.257	3.806	3.761	3.473	3.274	0.765
555	3.444	3.554	3.917	3.573	4.103	4.064	3.749	3.537	0.772
556	3.861	3.939	4.294	3.939	4.446	4.405	4.053	3.866	0.775
557	4.33	4.383	4.686	4.335	4.851	4.781	4.415	4.236	0.785
558	4.865	4.853	5.159	4.782	5.299	5.222	4.816	4.649	0.787
559	5.454	5.4	5.668	5.319	5.788	5.718	5.265	5.1	0.788
560	6.092	5.994	6.231	5.868	6.331	6.232	5.752	5.607	0.805
561	6.842	6.66	6.89	6.509	6.949	6.842	6.329	6.179	0.815
562	7.634	7.389	7.596	7.201	7.63	7.49	6.929	6.789	0.821
563	8.49	8.176	8.349	7.981	8.38	8.223	7.635	7.457	0.838
564	9.459	9.068	9.217	8.845	9.215	9.026	8.399	8.211	0.848
565	10.482	10.023	10.158	9.756	10.078	9.895	9.186	9.018	0.852
566	11.627	11.044	11.167	10.738	11.084	10.832	10.062	9.912	0.873
567	12.806	12.15	12.264	11.803	12.104	11.873	11.019	10.835	0.879
568	14.105	13.356	13.403	12.943	13.2	12.973	12.046	11.838	0.885
569	15.526	14.641	14.721	14.207	14.418	14.153	13.165	12.923	0.9
570	17.056	16.026	16.023	15.529	15.721	15.42	14.369	14.077	0.912
571	18.636	17.505	17.488	16.99	17.106	16.783	15.64	15.317	0.916
572	20.385	19.081	19.046	18.517	18.655	18.279	17.008	16.673	0.923
573	22.136	20.67	20.661	20.121	20.196	19.806	18.444	18.061	0.937
574	24.099	22.49	22.402	21.834	21.844	21.412	19.924	19.562	0.934
575	26.08	24.354	24.275	23.606	23.583	23.117	21.549	21.109	0.95
576	28.124	26.169	26.13	25.463	25.445	24.94	23.178	22.708	0.952
577	30.376	28.233	28.124	27.456	27.314	26.827	24.94	24.415	0.96
578	32.611	30.335	30.198	29.426	29.243	28.678	26.776	26.205	0.956
579	34.982	32.396	32.281	31.536	31.339	30.647	28.586	28.06	0.967
580	37.351	34.573	34.557	33.706	33.458	32.661	30.497	29.932	0.968
581	39.807	36.84	36.766	35.919	35.579	34.734	32.485	31.816	0.974
582	42.347	39.112	39.07	38.205	37.759	36.934	34.426	33.8	0.982
583	44.752	41.406	41.313	40.431	39.86	38.983	36.484	35.744	0.976
584	47.216	43.681	43.562	42.626	42.118	41.168	38.324	37.589	0.988
585	49.744	46.022	45.749	44.651	44.334	43.275	40.434	39.59	1.002
586	52.119	48.149	47.976	46.807	46.403	45.312	42.346	41.452	0.991
587	54.571	50.233	50.189	49.093	48.545	47.324	44.259	43.285	0.999
588	56.825	52.531	52.324	51.174	50.642	49.349	46.182	45.13	1.001
589	59.052	54.497	54.305	53.146	52.603	51.353	47.944	46.952	1.006
590	61.255	56.604	56.353	55.161	54.593	53.315	49.692	48.616	1.007
591	63.413	58.517	58.444	57.076	56.429	55.15	51.349	50.214	1.008
592	65.362	60.446	60.221	58.904	58.217	56.723	52.973	51.759	1.008

EXCLI Journal 2020;19:48-70 – ISSN 1611-2156
Received: October 03, 2019, accepted: December 19, 2019, published: January 03, 2020

Wavelength (nm)	Emission intensity								
	concentration of acetylshikonin (M)								
	A	B	C	D	E	F	G	H	J
	0.00	$4.0x10^{-6}$	$8.0x10^{-6}$	$1.0x10^{-5}$	$1.4x10^{-5}$	$1.6x10^{-5}$	$2.0x10^{-5}$	$2.4x10^{-5}$	$2.4x10^{-5}$
593	67.344	62.197	61.923	60.61	59.975	58.358	54.538	53.308	1.008
594	69.072	63.664	63.505	62.268	61.51	59.922	55.986	54.691	1.012
595	70.679	65.341	65.08	63.675	63.026	61.326	57.368	55.991	1.004
596	72.407	66.903	66.771	65.155	64.453	62.571	58.636	57.258	1.006
597	73.877	68.204	68.016	66.359	65.789	64.03	59.867	58.323	1.005
598	75.429	69.679	69.365	67.88	67.287	65.501	60.973	59.541	0.992
599	76.681	70.826	70.439	69.033	68.195	66.492	62.016	60.6	0.997
600	77.617	71.817	71.408	70.009	69.106	67.346	62.816	61.327	0.987
601	78.573	72.687	72.244	70.917	70.009	68.322	63.655	62.006	0.99
602	79.371	73.49	72.936	71.468	70.683	68.963	64.382	62.734	0.983
603	80.238	74.178	73.714	72.106	71.35	69.448	64.871	63.339	0.978
604	80.745	74.692	74.275	72.647	71.852	70.102	65.288	63.902	0.973
605	81.141	75.249	74.688	72.948	72.363	70.695	65.788	64.309	0.965
606	81.472	75.635	74.976	73.426	72.722	70.941	66.078	64.515	0.959
607	81.721	75.906	75.303	73.683	72.865	71.155	66.221	64.821	0.958
608	81.904	76.01	75.575	73.831	73.03	71.433	66.299	64.946	0.95
609	82.123	76.093	75.527	73.903	73.172	71.389	66.313	65.119	0.944
610	82.127	76.186	75.47	73.854	73.082	71.304	66.305	65.185	0.938
611	82.143	75.975	75.248	73.806	72.968	71.307	66.134	65.073	0.935
612	81.752	75.903	75.15	73.534	72.789	71.081	65.875	65.105	0.929
613	81.277	75.475	74.935	73.209	72.592	70.882	65.666	64.917	0.925
614	80.923	75.238	74.56	72.922	72.11	70.533	65.255	64.559	0.917
615	80.565	74.892	74.061	72.403	71.767	70.05	65.002	64.199	0.913
616	79.906	74.251	73.407	71.788	71.04	69.551	64.561	63.818	0.909
617	79.191	73.65	72.784	71.343	70.599	68.993	63.918	63.397	0.903
618	78.475	73.074	72.122	70.468	70.012	68.396	63.361	62.527	0.896
619	77.675	72.36	71.372	69.724	69.399	67.597	62.717	62.02	0.885
620	76.769	71.505	70.671	68.822	68.481	66.887	61.803	61.438	0.891
621	75.762	70.607	69.655	68.032	67.597	66.133	61.104	60.597	0.878
622	74.758	69.589	68.735	67.087	66.582	65.16	60.23	60.013	0.874
623	73.781	68.769	67.555	66.075	65.637	64.134	59.179	59.178	0.872
624	72.55	67.649	66.555	65.034	64.532	63.317	58.359	58.18	0.86
625	71.444	66.543	65.41	63.959	63.572	62.327	57.272	57.222	0.845
626	70.043	65.373	64.323	62.769	62.491	61.129	56.252	56.232	0.845
627	68.724	64.194	63.072	61.569	61.232	60.068	55.252	55.23	0.83
628	67.03	62.614	61.582	60.059	59.79	58.646	53.832	53.95	0.817
629	65.681	61.309	60.188	58.811	58.546	57.438	52.785	52.736	0.8
630	64.272	60.054	58.852	57.559	57.239	56.191	51.605	51.652	0.788
631	62.706	58.712	57.504	56.106	55.922	54.952	50.345	50.543	0.772
632	61.3	57.34	56.177	54.804	54.664	53.676	49.067	49.427	0.766
633	59.763	55.967	54.799	53.626	53.292	52.374	47.889	48.318	0.75
634	58.174	54.564	53.32	52.097	51.998	50.93	46.693	46.988	0.74
635	56.697	53.169	52	50.782	50.719	49.711	45.57	45.859	0.718
636	55.243	51.773	50.571	49.478	49.412	48.524	44.295	44.651	0.707
637	53.763	50.471	49.223	48.168	48.085	47.227	43.112	43.52	0.689
638	52.389	49.171	47.974	46.878	46.694	45.986	42.025	42.446	0.675
639	51.03	47.937	46.663	45.652	45.415	44.801	40.822	41.361	0.666
640	49.708	46.684	45.427	44.443	44.331	43.647	39.718	40.373	0.652

EXCLI Journal 2020;19:48-70 – ISSN 1611-2156
Received: October 03, 2019, accepted: December 19, 2019, published: January 03, 2020

Wavelength (nm)	***Emission intensity*** concentration of acetylshikonin (M)								
	A	*B*	*C*	*D*	*E*	*F*	*G*	*H*	*J*
	0.00	$4.0x10^{-6}$	$8.0x10^{-6}$	$1.0x10^{-5}$	$1.4x10^{-5}$	$1.6x10^{-5}$	$2.0x10^{-5}$	$2.4x10^{-5}$	$2.4x10^{-5}$
641	48.376	45.459	44.231	43.239	43.109	42.566	38.696	39.28	0.637
642	47.03	44.261	42.954	42.045	41.882	41.328	37.599	38.257	0.626
643	45.593	42.87	41.657	40.724	40.68	40.049	36.391	37.139	0.618
644	44.363	41.741	40.545	39.65	39.584	39.022	35.442	36.24	0.605
645	43.2	40.677	39.371	38.524	38.583	37.949	34.438	35.243	0.601
646	42.093	39.6	38.413	37.506	37.576	36.953	33.549	34.366	0.59
647	40.939	38.53	37.336	36.53	36.532	35.966	32.669	33.536	0.582
648	39.842	37.501	36.334	35.541	35.608	35.099	31.807	32.601	0.565
649	38.757	36.577	35.324	34.507	34.567	34.145	30.885	31.771	0.566
650	37.674	35.562	34.332	33.535	33.618	33.149	29.991	30.869	0.556
651	36.592	34.589	33.433	32.681	32.751	32.257	29.182	30.029	0.538
652	35.585	33.633	32.469	31.697	31.764	31.373	28.356	29.242	0.537
653	34.618	32.656	31.58	30.799	30.889	30.517	27.617	28.405	0.527
654	33.567	31.754	30.618	29.917	30.015	29.666	26.8	27.621	0.517
655	32.669	30.847	29.74	29.037	29.098	28.77	25.995	26.837	0.504
656	31.702	29.956	28.844	28.154	28.233	27.945	25.209	26.11	0.503
657	30.712	29.108	27.965	27.335	27.42	27.122	24.475	25.371	0.493
658	29.6	28.006	26.954	26.332	26.429	26.163	23.617	24.476	0.476
659	28.708	27.174	26.111	25.504	25.587	25.383	22.873	23.732	0.47
660	27.9	26.365	25.333	24.764	24.875	24.627	22.127	23.018	0.467
661	26.986	25.534	24.547	23.906	24.058	23.801	21.4	22.279	0.45
662	26.09	24.737	23.712	23.129	23.272	23.088	20.728	21.624	0.445
663	25.218	23.896	22.954	22.359	22.527	22.327	20.068	20.914	0.432
664	24.431	23.158	22.201	21.689	21.755	21.596	19.387	20.323	0.421
665	23.649	22.366	21.455	20.944	21.059	20.879	18.758	19.624	0.412
666	22.772	21.602	20.696	20.2	20.362	20.179	18.135	19.006	0.408
667	22.001	20.865	20.012	19.509	19.634	19.485	17.467	18.309	0.396
668	21.163	20.125	19.268	18.785	18.948	18.779	16.843	17.689	0.383
669	20.432	19.371	18.548	18.076	18.248	18.104	16.222	17.019	0.375
670	19.644	18.657	17.888	17.429	17.599	17.381	15.605	16.418	0.365
671	18.896	17.968	17.17	16.786	16.899	16.687	15.015	15.782	0.358
672	18.179	17.244	16.529	16.117	16.232	16.091	14.41	15.189	0.343
673	17.341	16.433	15.712	15.358	15.509	15.337	13.737	14.499	0.335
674	16.617	15.8	15.114	14.711	14.85	14.742	13.172	13.938	0.32
675	15.927	15.156	14.478	14.092	14.248	14.149	12.653	13.366	0.309
676	15.278	14.509	13.866	13.536	13.655	13.556	12.12	12.825	0.304
677	14.636	13.898	13.272	12.964	13.105	12.999	11.597	12.308	0.304
678	14.017	13.347	12.714	12.43	12.551	12.404	11.115	11.815	0.283
679	13.415	12.783	12.181	11.885	12.016	11.936	10.647	11.299	0.281
680	12.84	12.208	11.627	11.358	11.482	11.391	10.167	10.812	0.269
681	12.263	11.686	11.13	10.871	10.979	10.908	9.707	10.335	0.267
682	11.74	11.174	10.666	10.385	10.501	10.426	9.285	9.872	0.259
683	11.195	10.668	10.146	9.906	10.017	9.959	8.855	9.443	0.248
684	10.558	10.111	9.564	9.325	9.482	9.402	8.366	8.943	0.234
685	10.092	9.618	9.149	8.939	9.038	8.941	7.974	8.509	0.233
686	9.61	9.157	8.703	8.503	8.585	8.531	7.591	8.114	0.227
687	9.192	8.757	8.303	8.108	8.218	8.16	7.251	7.755	0.219
688	8.75	8.369	7.93	7.724	7.828	7.77	6.913	7.392	0.212

Wavelength (nm)	Emission intensity								
	concentration of acetylshikonin (M)								
	A	*B*	*C*	*D*	*E*	*F*	*G*	*H*	*J*
	0.00	$4.0x10^{-6}$	$8.0x10^{-6}$	$1.0x10^{-5}$	$1.4x10^{-5}$	$1.6x10^{-5}$	$2.0x10^{-5}$	$2.4x10^{-5}$	$2.4x10^{-5}$
689	8.329	7.966	7.562	7.368	7.482	7.413	6.585	7.048	0.209
690	7.948	7.595	7.187	7.009	7.13	7.066	6.288	6.726	0.198
691	7.577	7.251	6.864	6.685	6.769	6.741	5.978	6.421	0.193
692	7.233	6.894	6.547	6.37	6.466	6.423	5.701	6.122	0.194
693	6.81	6.516	6.18	5.998	6.101	6.061	5.377	5.784	0.187
694	6.492	6.205	5.879	5.72	5.804	5.762	5.134	5.505	0.184
695	6.181	5.918	5.598	5.442	5.531	5.504	4.891	5.249	0.18
696	5.874	5.629	5.307	5.179	5.268	5.236	4.643	5.003	0.178
697	5.589	5.355	5.068	4.927	5.003	4.976	4.405	4.77	0.17
698	5.311	5.09	4.808	4.672	4.765	4.734	4.204	4.533	0.162
699	5.071	4.854	4.57	4.454	4.537	4.513	3.999	4.32	0.163
700	4.778	4.577	4.312	4.209	4.285	4.262	3.781	4.077	0.157

Table 3: Emission intensity in the wavelength range of 550-700 nm for fluorescence emission spectra of DNA-EB fixed concentration (DNA (1.72 x 10^{-5} M) and EB (1.2 x 10^{-5} M)), in the absence (*A*) and presence of increasing concentration of *β*-hydroxyisovalerylshikonin (*B-H*), and emission intensity of *β*-hydroxyisovalerylshikonin in the absence of DNA-EB (*J*)

Wavelength (nm)	Emission intensity								
	concentration of *β*-hydroxyisovalerylshikonin (M)								
	A	*B*	*C*	*D*	*E*	*F*	*G*	*H*	*J*
	0.00	$4.0x10^{-6}$	$8.0x10^{-6}$	$1.0x10^{-5}$	$1.4x10^{-5}$	$1.6x10^{-5}$	$2.0x10^{-5}$	$2.4x10^{-5}$	$2.4x10^{-5}$
550	2.093	2.639	2.756	3.051	4.173	3.2	3.354	3.653	0.773
551	2.166	2.694	2.816	3.096	4.178	3.225	3.369	3.649	0.767
552	2.394	2.884	3.015	3.286	4.32	3.39	3.52	3.78	0.763
553	2.644	3.141	3.263	3.5	4.521	3.575	3.699	3.975	0.768
554	2.939	3.4	3.519	3.769	4.719	3.785	3.905	4.194	0.765
555	3.265	3.692	3.825	4.057	4.962	4.028	4.143	4.437	0.772
556	3.642	4.064	4.183	4.409	5.268	4.318	4.428	4.713	0.775
557	4.073	4.438	4.547	4.775	5.575	4.633	4.727	5.032	0.785
558	4.542	4.879	4.984	5.206	5.948	5.007	5.086	5.379	0.787
559	5.075	5.378	5.472	5.686	6.385	5.404	5.483	5.782	0.788
560	5.645	5.907	5.981	6.209	6.825	5.847	5.899	6.202	0.805
561	6.307	6.503	6.59	6.806	7.338	6.357	6.412	6.698	0.815
562	7.006	7.165	7.263	7.451	7.916	6.898	6.937	7.23	0.821
563	7.812	7.914	7.978	8.15	8.537	7.504	7.543	7.814	0.838
564	8.647	8.707	8.775	8.952	9.207	8.177	8.228	8.491	0.848
565	9.573	9.525	9.6	9.777	9.981	8.877	8.915	9.158	0.852
566	10.567	10.478	10.541	10.688	10.769	9.685	9.724	9.93	0.873
567	11.658	11.461	11.564	11.667	11.656	10.521	10.578	10.771	0.879
568	12.79	12.545	12.607	12.687	12.579	11.424	11.494	11.628	0.885
569	14.073	13.705	13.772	13.851	13.632	12.394	12.546	12.586	0.9
570	15.403	14.935	15.014	15.046	14.705	13.41	13.586	13.614	0.912
571	16.837	16.282	16.373	16.366	15.871	14.537	14.742	14.683	0.916
572	18.373	17.71	17.782	17.765	17.157	15.744	16.017	15.842	0.923
573	19.936	19.173	19.242	19.208	18.5	17.005	17.261	17.095	0.937

Wavelength (nm)	Emission intensity								
	concentration of *β*-hydroxyisovalerylshikonin (M)								
	A	*B*	*C*	*D*	*E*	*F*	*G*	*H*	*J*
	0.00	$4.0x10^{-6}$	$8.0x10^{-6}$	$1.0x10^{-5}$	$1.4x10^{-5}$	$1.6x10^{-5}$	$2.0x10^{-5}$	$2.4x10^{-5}$	$2.4x10^{-5}$
574	21.624	20.751	20.819	20.723	19.91	18.307	18.647	18.366	0.934
575	23.461	22.421	22.471	22.386	21.399	19.712	20.058	19.731	0.95
576	25.254	24.123	24.161	24.052	22.925	21.164	21.516	21.12	0.952
577	27.153	25.941	25.993	25.835	24.602	22.67	23.174	22.598	0.96
578	29.117	27.819	27.843	27.638	26.233	24.178	24.778	24.101	0.956
579	31.183	29.66	29.752	29.487	27.923	25.79	26.485	25.74	0.967
580	33.35	31.684	31.728	31.489	29.756	27.471	28.152	27.374	0.968
581	35.453	33.674	33.711	33.399	31.509	29.17	29.925	28.991	0.974
582	37.633	35.666	35.711	35.401	33.412	30.861	31.724	30.699	0.982
583	39.83	37.725	37.83	37.448	35.24	32.585	33.477	32.449	0.976
584	41.991	39.756	39.79	39.44	37.077	34.235	35.269	34.088	0.988
585	44.061	41.756	41.798	41.414	38.86	36.022	37.034	35.696	1.002
586	46.168	43.635	43.741	43.302	40.605	37.719	38.702	37.287	0.991
587	48.138	45.492	45.645	45.205	42.392	39.274	40.393	38.913	0.999
588	50.26	47.395	47.603	47.085	44.132	40.982	42.051	40.62	1.001
589	52.159	49.286	49.466	48.821	45.857	42.529	43.673	42.069	1.006
590	54.112	50.961	51.237	50.698	47.553	44.057	45.166	43.617	1.007
591	55.901	52.651	52.928	52.321	49.022	45.508	46.552	45.052	1.008
592	57.592	54.285	54.533	53.916	50.542	46.861	47.96	46.319	1.008
593	59.232	55.794	56.121	55.453	51.966	48.172	49.216	47.76	1.008
594	60.802	57.266	57.591	56.821	53.369	49.284	50.483	48.933	1.012
595	62.273	58.644	58.981	58.155	54.685	50.554	51.689	50.122	1.004
596	63.619	59.968	60.371	59.485	55.76	51.725	52.698	51.255	1.006
597	64.865	61.169	61.531	60.673	56.917	52.772	53.774	52.132	1.005
598	66.212	62.453	62.775	61.875	58.018	53.802	54.862	53.341	0.992
599	67.268	63.475	63.963	62.906	59.063	54.742	55.673	54.228	0.997
600	68.261	64.357	64.772	63.815	59.762	55.463	56.371	54.973	0.987
601	69.006	65.204	65.565	64.588	60.479	56.196	56.975	55.576	0.99
602	69.796	65.789	66.198	65.17	61.296	56.759	57.446	56.179	0.983
603	70.476	66.49	66.893	65.876	61.737	57.262	57.93	56.855	0.978
604	71.003	67.069	67.493	66.378	62.239	57.834	58.267	57.235	0.973
605	71.459	67.48	68.076	66.8	62.624	58.218	58.606	57.549	0.965
606	71.792	67.91	68.25	67.178	62.799	58.557	58.637	57.985	0.959
607	72.104	68.152	68.357	67.481	63.24	58.783	58.935	58.059	0.958
608	72.29	68.473	68.611	67.67	63.306	58.88	59.022	58.315	0.95
609	72.472	68.495	68.667	67.762	63.447	59.074	59.031	58.385	0.944
610	72.457	68.402	68.733	67.747	63.45	59.107	59.006	58.416	0.938
611	72.312	68.38	68.732	67.725	63.286	59.011	58.849	58.383	0.935
612	72.21	68.301	68.503	67.561	63.186	58.835	58.764	58.215	0.929
613	72.035	68.006	68.433	67.175	63.023	58.641	58.485	58.075	0.925
614	71.689	67.63	68.079	67.064	62.673	58.378	58.208	57.878	0.917
615	71.273	67.344	67.833	66.668	62.311	58.204	57.815	57.632	0.913
616	70.717	67.145	67.365	66.219	61.938	57.817	57.364	57.247	0.909
617	70.243	66.472	66.84	65.794	61.537	57.409	56.858	56.836	0.903
618	69.687	65.866	66.291	65.224	61.026	56.929	56.272	56.388	0.896
619	68.962	65.193	65.659	64.498	60.293	56.355	55.677	55.871	0.885
620	68.192	64.435	64.771	63.862	59.645	55.732	55.04	55.185	0.891
621	67.439	63.854	63.956	63.034	58.93	55.063	54.247	54.514	0.878

EXCLI Journal 2020;19:48-70 – ISSN 1611-2156
Received: October 03, 2019, accepted: December 19, 2019, published: January 03, 2020

Wavelength (nm)	**Emission intensity**								
	**concentration of β-hydroxyisovalerylshikonin (M)**								
	A	*B*	*C*	*D*	*E*	*F*	*G*	*H*	*J*
	0.00	$4.0x10^{-6}$	$8.0x10^{-6}$	$1.0x10^{-5}$	$1.4x10^{-5}$	$1.6x10^{-5}$	$2.0x10^{-5}$	$2.4x10^{-5}$	$2.4x10^{-5}$
622	66.635	62.982	63.284	62.266	58.043	54.417	53.539	53.856	0.874
623	65.589	62.155	62.364	61.485	57.308	53.645	52.679	53.16	0.872
624	64.513	61.123	61.388	60.489	56.448	52.871	51.864	52.388	0.86
625	63.598	59.989	60.398	59.6	55.49	52.052	50.971	51.619	0.845
626	62.457	59.118	59.379	58.534	54.502	51.203	49.951	50.748	0.845
627	61.272	58.082	58.304	57.472	53.562	50.294	49.017	49.894	0.83
628	59.747	56.746	56.916	56.096	52.281	49.08	47.9	48.72	0.817
629	58.534	55.512	55.818	55.065	51.133	48.061	46.932	47.657	0.8
630	57.359	54.369	54.671	53.892	50.004	47.031	45.914	46.654	0.788
631	56.051	53.226	53.353	52.606	48.885	45.988	44.735	45.745	0.772
632	54.747	51.913	52.121	51.45	47.778	44.948	43.706	44.657	0.766
633	53.457	50.719	50.963	50.192	46.647	43.871	42.63	43.699	0.75
634	52.078	49.483	49.686	49.059	45.503	42.89	41.664	42.607	0.74
635	50.84	48.315	48.471	47.861	44.397	41.812	40.496	41.629	0.718
636	49.478	47.045	47.16	46.641	43.302	40.762	39.444	40.596	0.707
637	48.2	45.791	46.019	45.464	42.163	39.713	38.386	39.616	0.689
638	46.979	44.669	44.805	44.266	41.043	38.624	37.38	38.476	0.675
639	45.773	43.538	43.655	43.141	39.888	37.73	36.372	37.544	0.666
640	44.528	42.378	42.5	42.054	38.939	36.814	35.39	36.627	0.652
641	43.339	41.336	41.436	40.953	37.932	35.821	34.393	35.72	0.637
642	42.181	40.153	40.333	39.875	36.905	34.892	33.534	34.744	0.626
643	40.92	39.011	39.106	38.572	35.712	33.852	32.491	33.757	0.618
644	39.901	38.02	38.114	37.66	34.825	33.009	31.639	32.898	0.605
645	38.825	36.994	37.108	36.619	33.895	32.177	30.806	32.011	0.601
646	37.846	36.066	36.169	35.641	32.999	31.308	29.981	31.222	0.59
647	36.901	35.12	35.244	34.763	32.132	30.572	29.127	30.398	0.582
648	35.914	34.184	34.269	33.851	31.266	29.752	28.363	29.586	0.565
649	34.995	33.272	33.375	33.05	30.452	28.947	27.659	28.851	0.566
650	34.047	32.434	32.476	32.093	29.591	28.229	26.87	28.098	0.556
651	33.091	31.531	31.595	31.285	28.776	27.411	26.129	27.316	0.538
652	32.193	30.685	30.731	30.439	28.006	26.667	25.404	26.628	0.537
653	31.247	29.877	29.858	29.573	27.234	26.009	24.642	25.926	0.527
654	30.401	28.977	29.043	28.787	26.464	25.285	23.935	25.238	0.517
655	29.556	28.185	28.271	27.901	25.695	24.591	23.24	24.506	0.504
656	28.657	27.397	27.47	27.126	24.953	23.846	22.52	23.796	0.503
657	27.868	26.614	26.654	26.358	24.266	23.149	21.886	23.135	0.493
658	26.841	25.687	25.654	25.397	23.374	22.4	21.018	22.326	0.476
659	26.013	24.891	24.945	24.672	22.704	21.732	20.456	21.7	0.47
660	25.216	24.167	24.147	23.92	22.038	21.086	19.849	21.044	0.467
661	24.404	23.403	23.398	23.181	21.317	20.366	19.177	20.367	0.45
662	23.643	22.674	22.65	22.476	20.669	19.719	18.599	19.752	0.445
663	22.908	21.944	21.927	21.738	19.967	19.122	17.945	19.119	0.432
664	22.168	21.244	21.262	21.055	19.345	18.519	17.36	18.518	0.421
665	21.483	20.584	20.567	20.371	18.739	17.922	16.801	17.942	0.412
666	20.722	19.866	19.846	19.728	18.075	17.363	16.223	17.343	0.408
667	20.041	19.167	19.181	19.06	17.452	16.728	15.664	16.767	0.396
668	19.285	18.527	18.475	18.374	16.846	16.185	15.071	16.175	0.383
669	18.577	17.882	17.789	17.693	16.226	15.595	14.505	15.587	0.375

Wavelength (nm)	Emission intensity								
	**concentration of β-hydroxyisovalerylshikonin (M)**								
	A	*B*	*C*	*D*	*E*	*F*	*G*	*H*	*J*
	0.00	$4.0x10^{-6}$	$8.0x10^{-6}$	$1.0x10^{-5}$	$1.4x10^{-5}$	$1.6x10^{-5}$	$2.0x10^{-5}$	$2.4x10^{-5}$	$2.4x10^{-5}$
670	17.936	17.187	17.173	17.033	15.636	15.044	13.985	14.983	0.365
671	17.25	16.554	16.508	16.419	15.053	14.512	13.473	14.452	0.358
672	16.552	15.911	15.908	15.798	14.476	13.928	12.928	13.903	0.343
673	15.803	15.185	15.156	15.042	13.788	13.296	12.323	13.272	0.335
674	15.182	14.586	14.578	14.483	13.243	12.77	11.826	12.752	0.32
675	14.577	13.992	13.991	13.895	12.677	12.242	11.354	12.258	0.309
676	13.963	13.418	13.417	13.326	12.148	11.735	10.885	11.771	0.304
677	13.428	12.888	12.858	12.779	11.674	11.263	10.413	11.277	0.304
678	12.841	12.353	12.345	12.256	11.156	10.808	9.998	10.823	0.283
679	12.318	11.817	11.818	11.741	10.727	10.373	9.571	10.371	0.281
680	11.778	11.331	11.303	11.234	10.255	9.931	9.141	9.949	0.269
681	11.26	10.802	10.82	10.761	9.798	9.489	8.752	9.508	0.267
682	10.777	10.351	10.344	10.307	9.395	9.089	8.353	9.093	0.259
683	10.296	9.889	9.895	9.83	8.951	8.693	7.974	8.697	0.248
684	9.736	9.36	9.326	9.306	8.489	8.223	7.535	8.225	0.234
685	9.29	8.933	8.894	8.871	8.087	7.844	7.186	7.836	0.233
686	8.862	8.526	8.505	8.469	7.726	7.48	6.86	7.484	0.227
687	8.467	8.17	8.118	8.084	7.381	7.148	6.54	7.158	0.219
688	8.077	7.771	7.73	7.72	7.045	6.821	6.234	6.825	0.212
689	7.712	7.433	7.378	7.373	6.72	6.513	5.946	6.509	0.209
690	7.357	7.06	7.065	7.016	6.405	6.218	5.659	6.218	0.198
691	7.009	6.752	6.733	6.721	6.101	5.941	5.398	5.929	0.193
692	6.672	6.441	6.403	6.403	5.814	5.671	5.167	5.66	0.194
693	6.32	6.077	6.045	6.062	5.496	5.353	4.892	5.36	0.187
694	6.028	5.79	5.776	5.765	5.231	5.111	4.632	5.1	0.184
695	5.739	5.531	5.507	5.485	4.981	4.873	4.433	4.86	0.18
696	5.454	5.265	5.258	5.234	4.756	4.643	4.207	4.627	0.178
697	5.204	5.009	5	4.983	4.519	4.421	4.004	4.426	0.17
698	4.954	4.784	4.755	4.751	4.307	4.226	3.814	4.211	0.162
699	4.728	4.539	4.537	4.523	4.114	4.01	3.643	4.02	0.163
700	4.447	4.3	4.293	4.267	3.877	3.788	3.424	3.802	0.157

Raw tables concerning **Figure 8.** The fluorescence emission spectra of Hoechst-DNA fixed concentration (DNA (1.66 x 10^{-5} M) and Hoechst (1.2 x 10^{-5} M)), in the absence and presence of increasing concentration of α-methylbutyrylshikon (***1***), acetylshikonin (***2***) and *β*-hydroxyisovalerylshikonin (***3***) (from 0 to 2.4 x 10^{-5} M). Arrow shows the intensity change upon the increase of the naphthoquinone concentration. Purple dashed line represents the emission spectra of ***1***, ***2*** and ***3*** in the absence of DNA-Hoechst. Right: corresponding plots of F_0/F versus [Q].

Table 1: Emission intensity in the wavelength range of 380-620 nm for fluorescence emission spectra of Hoechst-DNA fixed concentration (DNA (1.66 x 10^{-5} M) and Hoechst (1.2 x 10^{-5} M)), in the absence (*A*) and presence of increasing concentration of α-methylbutyrylshikon (*B*-*G*).

	Emission intensity						
**Wavelength (nm)**	**concentration of α-methylbutyrylshikon (M)**						
	A	*B*	*C*	*D*	*E*	*F*	*G*
	0.00	6.0x10^{-6}	1.0x10^{-5}	1.2x10^{-5}	1.4x10^{-5}	1.8x10^{-5}	2.4x10^{-5}
380	5.564	6.433	5.876	6.417	5.66	6.401	5.055
381	5.59	6.531	5.944	6.482	5.675	6.471	5.08
382	5.89	6.872	6.248	6.82	5.952	6.79	5.309
383	6.177	7.24	6.579	7.183	6.223	7.107	5.545
384	6.442	7.583	6.837	7.488	6.498	7.438	5.77
385	6.664	7.903	7.144	7.819	6.746	7.745	5.984
386	6.911	8.216	7.407	8.123	6.969	8.034	6.156
387	7.124	8.463	7.624	8.397	7.184	8.294	6.357
388	7.313	8.768	7.844	8.641	7.393	8.528	6.533
389	7.503	8.995	8.053	8.902	7.606	8.773	6.668
390	7.65	9.235	8.235	9.098	7.712	8.969	6.791
391	7.804	9.42	8.388	9.311	7.883	9.182	6.881
392	7.924	9.595	8.551	9.448	8.035	9.331	6.984
393	8.036	9.735	8.681	9.625	8.149	9.488	7.094
394	8.097	9.832	8.791	9.67	8.211	9.565	7.143
395	8.218	9.992	8.893	9.841	8.299	9.642	7.176
396	8.308	10.027	8.937	9.933	8.367	9.753	7.227
397	8.363	10.142	9.028	10.023	8.4	9.824	7.311
398	8.473	10.238	9.086	10.09	8.51	9.908	7.351
399	8.544	10.339	9.167	10.161	8.575	9.985	7.4
400	8.699	10.426	9.24	10.243	8.617	10.039	7.444
401	8.798	10.492	9.338	10.358	8.678	10.094	7.512
402	8.933	10.604	9.388	10.405	8.742	10.196	7.566
403	9.074	10.727	9.5	10.503	8.824	10.3	7.613
404	9.298	10.857	9.587	10.618	8.96	10.358	7.693
405	9.46	11.022	9.737	10.727	9.029	10.442	7.761
406	9.624	11.119	9.824	10.81	9.135	10.546	7.788
407	9.797	11.191	9.926	10.969	9.221	10.672	7.902
408	10.061	11.414	10.036	11.047	9.352	10.757	7.986
409	10.36	11.599	10.208	11.204	9.464	10.874	8.124
410	10.645	11.736	10.377	11.306	9.61	11.01	8.232
411	10.979	11.992	10.571	11.504	9.759	11.164	8.371
412	11.333	12.183	10.752	11.678	9.948	11.313	8.508
413	11.772	12.49	10.992	11.883	10.143	11.511	8.683
414	12.299	12.752	11.268	12.131	10.365	11.708	8.884
415	12.73	13.084	11.516	12.357	10.582	11.911	9.085

EXCLI Journal 2020;19:48-70 – ISSN 1611-2156
Received: October 03, 2019, accepted: December 19, 2019, published: January 03, 2020

	Emission intensity						
**Wavelength (nm)**	**concentration of α-methylbutyrylshikon (M)**						
	A	*B*	*C*	*D*	*E*	*F*	*G*
	0.00	$6.0x10^{-6}$	$1.0x10^{-5}$	$1.2x10^{-5}$	$1.4x10^{-5}$	$1.8x10^{-5}$	$2.4x10^{-5}$
416	13.343	13.484	11.847	12.621	10.932	12.201	9.343
417	13.971	13.848	12.206	12.978	11.211	12.494	9.612
418	14.644	14.37	12.631	13.325	11.585	12.825	9.93
419	15.246	14.855	12.99	13.673	11.885	13.045	10.244
420	16.135	15.403	13.516	14.052	12.284	13.487	10.613
421	17.03	15.993	14.068	14.597	12.765	13.913	11.045
422	18.038	16.708	14.648	15.116	13.334	14.43	11.514
423	19.045	17.45	15.339	15.783	13.913	14.962	12
424	20.213	18.3	16.043	16.438	14.512	15.553	12.517
425	21.401	19.158	16.758	17.087	15.188	16.156	13.086
426	22.603	20.111	17.626	17.838	15.857	16.817	13.704
427	23.929	21.067	18.436	18.681	16.601	17.472	14.431
428	25.359	22.166	19.372	19.467	17.364	18.33	15.126
429	26.765	23.27	20.361	20.396	18.255	19.133	15.839
430	28.379	24.365	21.35	21.302	19.085	19.91	16.636
431	29.915	25.632	22.469	22.376	20.013	20.79	17.396
432	31.635	26.849	23.561	23.333	20.91	21.758	18.252
433	33.248	28.193	24.663	24.389	21.879	22.678	19.161
434	34.961	29.523	25.817	25.454	22.917	23.631	20.108
435	36.812	30.958	27.132	26.599	24.036	24.617	20.96
436	38.353	32.065	28.058	27.561	24.807	25.359	21.757
437	40.143	33.412	29.275	28.757	25.967	26.469	22.684
438	41.999	34.967	30.531	29.869	27.06	27.512	23.68
439	43.994	36.523	31.763	31.121	28.23	28.609	24.753
440	46.057	38.103	33.169	32.374	29.402	29.678	25.742
441	47.857	39.399	34.567	33.665	30.554	30.826	26.937
442	49.958	41.215	35.891	34.888	31.779	31.976	27.952
443	51.924	42.797	37.409	36.226	32.936	33.012	29.066
444	53.984	44.414	38.824	37.567	34.245	34.173	30.194
445	56.087	46.051	40.333	38.969	35.581	35.428	31.274
446	58.069	47.78	41.65	40.267	36.649	36.584	32.412
447	60.337	49.293	42.946	41.701	37.919	37.646	33.475
448	61.817	51.058	44.547	42.959	39.176	38.939	34.524
449	63.889	52.496	45.852	43.942	40.383	40.157	35.527
450	66.052	54.173	47.233	45.369	41.451	41.139	36.751
451	67.892	55.824	48.657	46.702	42.575	42.291	37.761
452	69.741	57.317	50.114	47.916	43.902	43.385	38.886
453	71.758	59	51.362	49.158	44.967	44.45	40.004
454	73.043	59.995	52.491	50.16	45.873	45.24	40.877
455	74.942	61.59	53.71	51.486	47.02	46.386	41.831
456	76.73	63.083	55.044	52.565	48.225	47.489	42.751
457	78.311	64.29	56.169	53.882	49.176	48.551	43.765
458	79.879	65.902	57.39	54.807	50.324	49.394	44.892
459	81.416	67.272	58.652	56.004	51.351	50.354	45.614
460	83.08	68.522	59.906	57.108	52.193	51.226	46.481
461	84.312	69.731	60.783	57.878	53.098	52.178	47.467
462	85.457	70.972	61.608	58.996	54.034	52.96	48.158
463	86.974	72.026	62.963	59.942	54.993	53.614	48.963

EXCLI Journal 2020;19:48-70 – ISSN 1611-2156
Received: October 03, 2019, accepted: December 19, 2019, published: January 03, 2020

Wavelength (nm)	Emission intensity						
	concentration of α-methylbutyrylshikon (M)						
	A	*B*	*C*	*D*	*E*	*F*	*G*
	0.00	$6.0x10^{-6}$	$1.0x10^{-5}$	$1.2x10^{-5}$	$1.4x10^{-5}$	$1.8x10^{-5}$	$2.4x10^{-5}$
464	88.372	73.22	63.695	60.803	55.548	54.484	49.823
465	89.328	74.017	64.633	61.72	56.32	55.121	50.465
466	90.393	75.181	65.478	62.298	57.212	55.799	51.053
467	91.623	76.324	66.162	63.071	57.691	56.23	51.837
468	92.692	76.94	67.121	63.955	58.34	57.068	52.447
469	93.344	77.869	67.758	64.542	59.05	57.555	52.995
470	94.282	78.543	68.692	65.119	59.646	58.14	53.488
471	95.186	79.116	69.171	65.788	60.066	58.437	53.891
472	95.411	79.845	69.603	66.114	60.635	58.804	54.104
473	96.399	80.527	69.561	66.462	60.779	59.184	54.694
474	96.591	80.801	70.472	67.152	61.422	59.441	55.108
475	97.022	81.558	70.865	67.42	61.704	59.934	55.313
476	97.453	81.954	71.035	67.626	61.946	60.05	55.653
477	97.588	82.016	71.425	68.129	61.975	60.323	56.007
478	97.693	82.121	71.887	68.327	62.307	60.427	56.131
479	98.028	82.891	71.885	68.493	62.503	60.608	56.339
480	98.126	82.746	71.956	68.607	62.48	60.679	56.497
481	98.168	82.796	72.141	68.489	62.654	60.765	56.334
482	98.149	82.858	72.183	68.459	62.662	60.712	56.507
483	98.179	83.161	72.141	68.65	62.556	60.72	56.496
484	98.151	82.72	72.083	68.382	62.062	60.454	56.254
485	97.54	82.816	72.003	68.287	62.292	60.475	56.271
486	97.449	82.476	71.808	68.246	61.97	60.049	56.095
487	96.895	82.216	71.478	67.911	62.118	60.076	56.064
488	96.563	81.995	71.513	67.797	61.804	59.701	55.766
489	95.635	81.365	70.975	67.366	61.302	59.38	55.664
490	95.342	81.418	70.564	66.992	61.083	59.043	55.374
491	94.573	80.693	70.195	66.617	60.716	58.742	55.072
492	94.021	80.054	69.714	66.317	60.286	58.117	54.751
493	93.251	79.354	69.186	65.934	59.98	57.651	54.347
494	92.301	79.017	68.833	65.164	59.384	57.426	53.832
495	91.441	78.39	68.132	64.63	58.742	56.865	53.354
496	90.485	77.682	67.388	64.177	58.42	56.366	53.038
497	89.573	77.177	66.89	63.469	57.652	55.776	52.442
498	88.7	76.358	66.335	63.01	56.997	55.244	52.031
499	87.762	75.598	65.552	62.246	56.637	54.57	51.464
500	86.597	74.486	64.869	61.784	55.882	53.977	50.861
501	85.732	73.729	64.103	61.031	55.323	53.416	50.37
502	84.784	72.853	63.239	60.12	54.623	52.826	49.711
503	83.734	72.134	62.79	59.549	54.198	52.121	49.303
504	82.538	71.367	61.942	58.864	53.468	51.36	48.649
505	81.434	70.387	61.072	57.995	52.711	50.695	48.157
506	80.103	69.447	60.265	57.243	51.971	50.103	47.359
507	78.805	68.476	59.29	56.465	51.324	49.256	46.73
508	77.794	67.358	58.362	55.6	50.395	48.611	45.89
509	76.278	66.175	57.526	54.74	49.559	47.773	45.195
510	75.054	65.218	56.759	53.855	48.79	47.16	44.498
511	73.616	64.351	55.756	52.834	47.906	46.321	43.995

EXCLI Journal 2020;19:48-70 – ISSN 1611-2156
Received: October 03, 2019, accepted: December 19, 2019, published: January 03, 2020

Wavelength (nm)	Emission intensity						
	concentration of α-methylbutyrylshikon (M)						
	A	*B*	*C*	*D*	*E*	*F*	*G*
	0.00	$6.0x10^{-6}$	$1.0x10^{-5}$	$1.2x10^{-5}$	$1.4x10^{-5}$	$1.8x10^{-5}$	$2.4x10^{-5}$
512	72.26	63.044	54.921	52.192	47.221	45.376	43.236
513	71.049	62.134	53.823	51.341	46.456	44.693	42.523
514	69.651	60.913	53.117	50.412	45.619	43.896	41.78
515	68.449	59.844	52.05	49.416	44.848	43.062	41.171
516	67.16	58.706	51.002	48.641	44.011	42.418	40.254
517	65.571	57.588	50.194	47.692	43.167	41.496	39.644
518	64.523	56.475	49.271	46.681	42.442	40.721	38.953
519	63.074	55.413	48.269	45.744	41.576	39.859	38.31
520	61.75	54.363	47.091	44.864	40.734	39.09	37.445
521	60.561	53.114	46.406	44.039	39.926	38.328	36.832
522	59.189	52.064	45.366	43.065	39.161	37.418	36.118
523	57.915	51.076	44.342	42.28	38.339	36.674	35.387
524	56.678	49.963	43.392	41.227	37.422	35.928	34.609
525	55.312	48.799	42.381	40.508	36.636	34.949	33.772
526	53.942	47.731	41.523	39.414	35.848	34.293	33.169
527	52.745	46.795	40.555	38.549	34.882	33.568	32.494
528	51.417	45.466	39.637	37.834	34.282	32.821	31.824
529	50.315	44.347	38.771	36.913	33.442	32.106	31.046
530	48.965	43.533	37.764	36.07	32.735	31.373	30.268
531	47.763	42.356	36.92	35.214	31.955	30.643	29.576
532	46.636	41.306	36.025	34.298	31.053	29.854	28.874
533	45.332	40.352	35.03	33.369	30.271	29.153	28.272
534	44.277	39.351	34.163	32.587	29.596	28.306	27.479
535	43.148	38.315	33.483	31.832	28.821	27.597	26.901
536	41.901	37.392	32.599	30.989	28.143	26.951	26.253
537	40.794	36.347	31.721	30.151	27.432	26.247	25.628
538	39.916	35.552	30.985	29.493	26.737	25.668	24.963
539	38.748	34.602	30.19	28.703	26.107	24.959	24.436
540	37.694	33.681	29.413	27.958	25.38	24.409	23.836
541	36.793	32.921	28.631	27.234	24.833	23.748	23.302
542	35.821	32.039	28.053	26.533	24.201	23.174	22.661
543	34.992	31.252	27.361	25.935	23.624	22.65	22.153
544	34.162	30.557	26.668	25.281	23.086	22.161	21.702
545	33.363	29.886	25.99	24.824	22.485	21.586	21.25
546	32.474	29.168	25.438	24.168	21.854	21.099	20.788
547	31.719	28.54	24.913	23.679	21.56	20.677	20.225
548	31.114	27.909	24.355	23.126	21.051	20.247	19.885
549	30.361	27.288	23.936	22.61	20.588	19.772	19.454
550	29.743	26.68	23.35	22.25	20.256	19.416	19.028
551	29.088	26.119	22.878	21.747	19.797	19	18.698
552	28.392	25.487	22.372	21.208	19.415	18.532	18.356
553	27.72	24.972	21.876	20.753	18.938	18.181	17.912
554	27.059	24.48	21.516	20.383	18.584	17.778	17.582
555	26.617	23.907	20.988	19.92	18.18	17.455	17.261
556	25.906	23.543	20.627	19.541	17.788	17.078	16.924
557	25.313	22.98	20.12	19.136	17.465	16.731	16.49
558	24.751	22.506	19.678	18.697	17.041	16.356	16.191
559	24.154	21.882	19.233	18.271	16.726	16.014	15.864

Wavelength (nm)	***Emission intensity***						
	**concentration of α-methylbutyrylshikon (M)**						
	A	*B*	*C*	*D*	*E*	*F*	*G*
	0.00	$6.0x10^{-6}$	$1.0x10^{-5}$	$1.2x10^{-5}$	$1.4x10^{-5}$	$1.8x10^{-5}$	$2.4x10^{-5}$
560	23.573	21.471	18.802	17.901	16.404	15.712	15.523
561	23.035	20.937	18.403	17.495	15.992	15.371	15.19
562	22.513	20.452	17.953	17.046	15.59	15.035	14.845
563	21.936	19.953	17.533	16.727	15.25	14.68	14.495
564	21.37	19.447	17.188	16.285	14.885	14.327	14.138
565	20.906	18.996	16.689	15.9	14.544	13.956	13.787
566	20.319	18.497	16.273	15.473	14.163	13.622	13.477
567	19.683	18.079	15.871	15.145	13.809	13.342	13.138
568	19.205	17.594	15.443	14.707	13.433	12.97	12.804
569	18.7	17.108	15.071	14.349	13.139	12.641	12.426
570	18.162	16.628	14.678	13.941	12.747	12.305	12.074
571	17.592	16.176	14.194	13.542	12.42	11.958	11.803
572	17.067	15.689	13.83	13.178	12.075	11.621	11.447
573	16.598	15.23	13.436	12.792	11.703	11.286	11.064
574	15.991	14.768	12.979	12.414	11.372	10.916	10.78
575	15.529	14.274	12.592	11.989	10.986	10.609	10.421
576	15.008	13.847	12.254	11.654	10.671	10.288	10.114
577	14.464	13.354	11.831	11.301	10.361	9.996	9.802
578	13.966	12.915	11.423	10.967	10.006	9.673	9.492
579	13.505	12.484	11.081	10.578	9.652	9.318	9.21
580	12.987	12.097	10.635	10.201	9.317	9.026	8.893
581	12.529	11.632	10.314	9.843	8.994	8.695	8.596
582	12.066	11.202	9.922	9.464	8.68	8.391	8.273
583	11.606	10.784	9.534	9.115	8.35	8.076	7.974
584	11.184	10.361	9.175	8.769	8.065	7.776	7.695
585	10.721	9.955	8.827	8.453	7.749	7.478	7.381
586	10.276	9.569	8.481	8.121	7.46	7.198	7.127
587	9.842	9.157	8.141	7.804	7.165	6.915	6.801
588	9.402	8.766	7.814	7.482	6.871	6.623	6.544
589	9.037	8.394	7.457	7.135	6.578	6.35	6.286
590	8.583	8.04	7.171	6.856	6.317	6.103	6.021
591	8.226	7.667	6.855	6.54	6.014	5.822	5.775
592	7.85	7.338	6.534	6.249	5.737	5.581	5.536
593	7.476	6.971	6.258	5.982	5.512	5.34	5.26
594	7.145	6.691	5.958	5.713	5.262	5.109	5.058
595	6.785	6.35	5.676	5.482	5.02	4.89	4.816
596	6.456	6.037	5.41	5.21	4.804	4.668	4.592
597	6.139	5.747	5.171	4.954	4.577	4.441	4.37
598	5.776	5.431	4.85	4.677	4.324	4.209	4.14
599	5.487	5.15	4.627	4.458	4.127	4.02	3.944
600	5.223	4.912	4.418	4.245	3.931	3.828	3.765
601	4.97	4.687	4.234	4.061	3.777	3.68	3.607
602	4.736	4.473	4.038	3.862	3.592	3.515	3.441
603	4.5	4.256	3.838	3.699	3.438	3.346	3.287
604	4.279	4.063	3.649	3.525	3.275	3.208	3.138
605	4.067	3.846	3.501	3.366	3.14	3.065	2.996
606	3.897	3.674	3.331	3.216	2.997	2.936	2.874
607	3.713	3.526	3.187	3.068	2.875	2.806	2.752

EXCLI Journal 2020;19:48-70 – ISSN 1611-2156
Received: October 03, 2019, accepted: December 19, 2019, published: January 03, 2020

Wavelength (nm)	Emission intensity						
	concentration of α-methylbutyrylshikon (M)						
	A	*B*	*C*	*D*	*E*	*F*	*G*
	0.00	$6.0x10^{-6}$	$1.0x10^{-5}$	$1.2x10^{-5}$	$1.4x10^{-5}$	$1.8x10^{-5}$	$2.4x10^{-5}$
608	3.536	3.37	3.065	2.951	2.756	2.712	2.633
609	3.369	3.218	2.927	2.825	2.65	2.591	2.533
610	3.237	3.083	2.789	2.7	2.539	2.491	2.418
611	3.094	2.948	2.677	2.59	2.43	2.393	2.314
612	2.957	2.818	2.576	2.473	2.344	2.288	2.222
613	2.815	2.697	2.444	2.381	2.241	2.207	2.141
614	2.704	2.585	2.359	2.274	2.153	2.121	2.049
615	2.591	2.466	2.259	2.183	2.075	2.035	1.969
616	2.471	2.375	2.16	2.096	1.992	1.96	1.887
617	2.362	2.258	2.076	2.012	1.914	1.887	1.814
618	2.258	2.169	1.985	1.923	1.846	1.809	1.73
619	2.152	2.079	1.92	1.853	1.776	1.737	1.665
620	2.042	1.976	1.828	1.767	1.705	1.668	1.591

Table 2: Emission intensity in the wavelength range of 380-620 nm for fluorescence emission spectra of Hoechst-DNA fixed concentration (DNA (1.66 x 10^{-5} M) and Hoechst (1.2 x 10^{-5} M)), in the absence (*A*) and presence of increasing concentration of acetylshikonin (*B-G*).

Wavelength (nm)	Emission intensity						
	concentration of acetylshikonin (M)						
	A	*B*	*C*	*D*	*E*	*F*	*G*
	0.00	$6.0x10^{-6}$	$1.0x10^{-5}$	$1.2x10^{-5}$	$1.4x10^{-5}$	$1.8x10^{-5}$	$2.4x10^{-5}$
380	5.564	5.605	5.365	5.175	5.199	5.055	5.032
381	5.59	5.644	5.439	5.205	5.229	5.08	5.084
382	5.89	5.911	5.72	5.474	5.477	5.309	5.371
383	6.177	6.171	6.014	5.759	5.744	5.545	5.631
384	6.442	6.416	6.279	5.999	5.981	5.77	5.876
385	6.664	6.658	6.534	6.253	6.222	5.984	6.084
386	6.911	6.87	6.755	6.477	6.397	6.156	6.278
387	7.124	7.069	6.975	6.689	6.639	6.357	6.495
388	7.313	7.249	7.193	6.87	6.82	6.533	6.682
389	7.503	7.439	7.362	7.041	6.927	6.668	6.818
390	7.65	7.611	7.53	7.166	7.115	6.791	6.957
391	7.804	7.734	7.677	7.287	7.236	6.881	7.097
392	7.924	7.871	7.793	7.411	7.339	6.984	7.194
393	8.036	7.958	7.892	7.527	7.45	7.094	7.254
394	8.097	7.996	7.964	7.601	7.506	7.143	7.338
395	8.218	8.118	8.037	7.682	7.574	7.176	7.418
396	8.308	8.191	8.122	7.753	7.641	7.227	7.465
397	8.363	8.278	8.224	7.821	7.732	7.311	7.522
398	8.473	8.341	8.296	7.87	7.777	7.351	7.586
399	8.544	8.408	8.336	7.928	7.817	7.4	7.619
400	8.699	8.463	8.445	8.025	7.881	7.444	7.665
401	8.798	8.576	8.526	8.067	7.958	7.512	7.718
402	8.933	8.677	8.605	8.187	8.029	7.566	7.736
403	9.074	8.768	8.734	8.263	8.094	7.613	7.813
404	9.298	8.872	8.848	8.374	8.216	7.693	7.868

Wavelength (nm)	***Emission intensity***						
	**concentration of acetylshikonin (M)**						
	A	*B*	*C*	*D*	*E*	*F*	*G*
	0.00	$6.0x10^{-6}$	$1.0x10^{-5}$	$1.2x10^{-5}$	$1.4x10^{-5}$	$1.8x10^{-5}$	$2.4x10^{-5}$
405	9.46	9.023	8.975	8.471	8.283	7.761	7.937
406	9.624	9.133	9.034	8.546	8.343	7.788	7.993
407	9.797	9.31	9.164	8.685	8.514	7.902	8.053
408	10.061	9.462	9.358	8.853	8.63	7.986	8.11
409	10.36	9.656	9.49	8.955	8.7	8.124	8.176
410	10.645	9.896	9.626	9.138	8.879	8.232	8.29
411	10.979	10.155	9.896	9.314	9.06	8.371	8.353
412	11.333	10.429	10.072	9.531	9.225	8.508	8.484
413	11.772	10.798	10.346	9.785	9.423	8.683	8.556
414	12.299	11.151	10.629	10.009	9.639	8.884	8.698
415	12.73	11.569	10.96	10.329	9.914	9.085	8.856
416	13.343	11.986	11.286	10.635	10.221	9.343	9.011
417	13.971	12.481	11.65	11.028	10.548	9.612	9.217
418	14.644	13.013	12.112	11.406	10.902	9.93	9.398
419	15.246	13.533	12.526	11.75	11.234	10.244	9.606
420	16.135	14.22	13.051	12.28	11.671	10.613	9.89
421	17.03	14.922	13.661	12.866	12.171	11.045	10.233
422	18.038	15.69	14.264	13.441	12.727	11.514	10.536
423	19.045	16.47	14.97	14.121	13.314	12	10.939
424	20.213	17.445	15.756	14.873	13.904	12.517	11.329
425	21.401	18.396	16.525	15.57	14.634	13.086	11.812
426	22.603	19.363	17.391	16.389	15.333	13.704	12.28
427	23.929	20.503	18.305	17.22	16.113	14.431	12.779
428	25.359	21.678	19.225	18.118	16.942	15.126	13.365
429	26.765	22.807	20.297	19.08	17.774	15.839	13.919
430	28.379	24.04	21.36	20.046	18.723	16.636	14.501
431	29.915	25.437	22.454	21.128	19.581	17.396	15.187
432	31.635	26.813	23.646	22.189	20.611	18.252	15.817
433	33.248	28.254	24.839	23.377	21.735	19.161	16.466
434	34.961	29.782	26.014	24.554	22.679	20.108	17.244
435	36.812	31.349	27.394	25.707	23.877	20.96	17.972
436	38.353	32.636	28.277	26.616	24.754	21.757	18.585
437	40.143	34.1	29.791	27.923	25.887	22.684	19.357
438	41.999	35.663	31.093	29.313	27.003	23.68	20.11
439	43.994	37.339	32.483	30.522	28.274	24.753	21.001
440	46.057	39.034	34.025	31.973	29.523	25.742	21.771
441	47.857	40.779	35.416	33.351	30.814	26.937	22.619
442	49.958	42.603	36.96	34.785	32.053	27.952	23.461
443	51.924	44.27	38.402	36.131	33.352	29.066	24.312
444	53.984	46.151	39.98	37.51	34.594	30.194	25.2
445	56.087	47.946	41.591	39.002	35.982	31.274	26.108
446	58.069	49.7	43.118	40.498	37.286	32.412	26.953
447	60.337	51.393	44.63	42	38.533	33.475	27.926
448	61.817	53.013	46.212	43.389	39.932	34.524	28.786
449	63.889	54.854	47.608	44.699	41.206	35.527	29.589
450	66.052	56.66	49.217	46.127	42.565	36.751	30.533
451	67.892	58.183	50.618	47.574	43.73	37.761	31.386
452	69.741	60.045	52.129	48.827	45.124	38.886	32.237

EXCLI Journal 2020;19:48-70 – ISSN 1611-2156
Received: October 03, 2019, accepted: December 19, 2019, published: January 03, 2020

Wavelength (nm)	***Emission intensity***						
	**concentration of acetylshikonin (M)**						
	A	*B*	*C*	*D*	*E*	*F*	*G*
	0.00	$6.0x10^{-6}$	$1.0x10^{-5}$	$1.2x10^{-5}$	$1.4x10^{-5}$	$1.8x10^{-5}$	$2.4x10^{-5}$
453	71.758	61.643	53.572	50.41	46.349	40.004	33.047
454	73.043	62.905	54.615	51.451	47.253	40.877	33.739
455	74.942	64.555	56.119	52.68	48.507	41.831	34.581
456	76.73	65.769	57.522	54.103	49.747	42.751	35.415
457	78.311	67.245	58.915	55.188	50.731	43.765	36.102
458	79.879	68.855	60.239	56.556	51.75	44.892	36.961
459	81.416	69.984	61.391	57.719	52.96	45.614	37.777
460	83.08	71.552	62.832	58.943	53.956	46.481	38.42
461	84.312	72.743	63.944	60.038	54.862	47.467	39.212
462	85.457	73.753	65.218	61.081	56.032	48.158	39.947
463	86.974	74.97	66.24	62.031	56.708	48.963	40.528
464	88.372	76.099	67.455	63.093	57.616	49.823	41.284
465	89.328	77.168	68.261	64.046	58.4	50.465	41.742
466	90.393	78.137	69.192	64.785	59.304	51.053	42.279
467	91.623	78.77	70.018	65.869	60.002	51.837	42.935
468	92.692	79.91	70.942	66.568	60.691	52.447	43.317
469	93.344	80.646	71.778	67.297	61.095	52.995	43.846
470	94.282	81.378	72.539	67.827	62.071	53.488	44.244
471	95.186	82.251	73.074	68.563	62.561	53.891	44.814
472	95.411	82.642	73.683	69.052	63.112	54.104	45.053
473	96.399	83.1	74.211	69.617	63.602	54.694	45.219
474	96.591	83.52	74.848	70.149	63.873	55.108	45.599
475	97.022	84.064	75.246	70.404	64.386	55.313	45.955
476	97.453	84.542	75.827	70.867	64.729	55.653	46.15
477	97.588	84.852	75.957	71.161	65.031	56.007	46.432
478	97.693	84.952	76.302	71.306	65.23	56.131	46.59
479	98.028	85.092	76.645	71.753	65.376	56.339	46.752
480	98.126	85.233	76.686	71.847	65.58	56.497	46.833
481	98.168	85.059	76.594	71.865	65.661	56.334	47.028
482	98.149	85.3	77.011	71.946	65.961	56.507	47.062
483	98.179	85.326	76.789	72.217	65.71	56.496	47.131
484	98.151	85.135	76.93	72.117	65.532	56.254	46.963
485	97.54	84.946	76.867	72.097	65.521	56.271	47.039
486	97.449	84.558	76.622	71.802	65.3	56.095	46.824
487	96.895	84.335	76.443	71.867	65.178	56.064	46.806
488	96.563	83.944	76.119	71.464	64.875	55.766	46.709
489	95.635	83.724	75.666	71.275	64.695	55.664	46.419
490	95.342	83.303	75.41	70.84	64.52	55.374	46.31
491	94.573	82.634	75.12	70.326	64.006	55.072	46.026
492	94.021	81.907	74.219	69.942	63.693	54.751	45.836
493	93.251	81.34	73.883	69.423	63.232	54.347	45.49
494	92.301	80.727	73.615	69.079	62.666	53.832	45.212
495	91.441	79.953	73.047	68.462	62.222	53.354	44.765
496	90.485	78.968	72.426	67.778	61.72	53.038	44.476
497	89.573	78.43	71.778	67.124	61.131	52.442	44.065
498	88.7	77.47	70.969	66.446	60.601	52.031	43.703
499	87.762	76.649	70.268	66.016	60.062	51.464	43.361
500	86.597	75.575	69.648	65.274	59.477	50.861	42.822

EXCLI Journal 2020;19:48-70 – ISSN 1611-2156
Received: October 03, 2019, accepted: December 19, 2019, published: January 03, 2020

Wavelength (nm)	***Emission intensity***						
	**concentration of acetylshikonin (M)**						
	A	*B*	*C*	*D*	*E*	*F*	*G*
	0.00	$6.0x10^{-6}$	$1.0x10^{-5}$	$1.2x10^{-5}$	$1.4x10^{-5}$	$1.8x10^{-5}$	$2.4x10^{-5}$
501	85.732	74.714	68.613	64.704	58.835	50.37	42.386
502	84.784	73.53	67.945	63.769	57.763	49.711	41.939
503	83.734	72.773	67.452	63.287	57.526	49.303	41.421
504	82.538	71.796	66.536	62.451	56.838	48.649	41.139
505	81.434	70.662	65.58	61.551	56.241	48.157	40.643
506	80.103	69.972	64.635	60.613	55.5	47.359	40.104
507	78.805	68.576	63.747	59.692	54.648	46.73	39.399
508	77.794	67.454	62.843	58.864	53.934	45.89	38.878
509	76.278	66.516	61.967	57.999	53.206	45.195	38.336
510	75.054	65.278	60.9	57.193	52.37	44.498	37.778
511	73.616	64.451	60.062	56.068	51.423	43.995	37.165
512	72.26	63.281	59.032	55.311	50.796	43.236	36.684
513	71.049	62.201	58.194	54.25	49.841	42.523	36.005
514	69.651	60.727	57.158	53.271	48.967	41.78	35.34
515	68.449	59.835	56.154	52.306	48.202	41.171	34.751
516	67.16	58.754	55.081	51.237	47.246	40.254	34.157
517	65.571	57.411	54.103	50.496	46.365	39.644	33.631
518	64.523	56.351	52.996	49.317	45.504	38.953	32.973
519	63.074	55.344	52.077	48.271	44.659	38.31	32.361
520	61.75	54.112	50.902	47.542	43.847	37.445	31.685
521	60.561	52.932	49.885	46.492	42.961	36.832	31.172
522	59.189	51.772	48.964	45.46	42.017	36.118	30.535
523	57.915	50.881	48.087	44.51	41.184	35.387	29.869
524	56.678	49.422	46.858	43.505	40.31	34.609	29.355
525	55.312	48.33	46.068	42.509	39.318	33.772	28.553
526	53.942	47.294	44.796	41.716	38.465	33.169	27.959
527	52.745	46.153	43.787	40.665	37.696	32.494	27.359
528	51.417	45.234	42.94	39.813	36.833	31.824	26.717
529	50.315	44.194	41.888	38.976	35.953	31.046	26.203
530	48.965	43.142	40.894	37.986	35.21	30.268	25.624
531	47.763	42.155	39.831	36.994	34.181	29.576	25.05
532	46.636	41.029	38.925	36.077	33.566	28.874	24.442
533	45.332	40.091	38.005	35.108	32.735	28.272	23.929
534	44.277	38.886	37.046	34.376	31.841	27.479	23.242
535	43.148	37.97	36.083	33.451	31.037	26.901	22.679
536	41.901	37.043	35.163	32.619	30.26	26.253	22.212
537	40.794	36.104	34.22	31.704	29.509	25.628	21.671
538	39.916	35.195	33.425	30.964	28.731	24.963	21.124
539	38.748	34.152	32.565	30.179	28.028	24.436	20.646
540	37.694	33.281	31.658	29.384	27.403	23.836	20.19
541	36.793	32.422	30.961	28.731	26.716	23.302	19.627
542	35.821	31.592	30.149	27.942	26.057	22.661	19.187
543	34.992	30.828	29.445	27.231	25.422	22.153	18.71
544	34.162	30.06	28.791	26.734	24.784	21.702	18.328
545	33.363	29.396	28.104	26.023	24.162	21.25	17.936
546	32.474	28.747	27.415	25.452	23.642	20.788	17.528
547	31.719	28.189	26.895	24.877	23.221	20.225	17.173
548	31.114	27.467	26.239	24.381	22.633	19.885	16.81

EXCLI Journal 2020;19:48-70 – ISSN 1611-2156
Received: October 03, 2019, accepted: December 19, 2019, published: January 03, 2020

Wavelength (nm)	***Emission intensity***						
	**concentration of acetylshikonin (M)**						
	A	*B*	*C*	*D*	*E*	*F*	*G*
	0.00	$6.0x10^{-6}$	$1.0x10^{-5}$	$1.2x10^{-5}$	$1.4x10^{-5}$	$1.8x10^{-5}$	$2.4x10^{-5}$
549	30.361	26.821	25.673	23.846	22.199	19.454	16.463
550	29.743	26.256	25.189	23.282	21.673	19.028	16.138
551	29.088	25.759	24.608	22.802	21.265	18.698	15.807
552	28.392	25.122	24.065	22.295	20.839	18.356	15.515
553	27.72	24.493	23.574	21.863	20.416	17.912	15.167
554	27.059	24.039	23.063	21.377	19.968	17.582	14.905
555	26.617	23.521	22.618	20.93	19.576	17.261	14.647
556	25.906	22.956	22.055	20.489	19.161	16.924	14.302
557	25.313	22.456	21.615	19.983	18.74	16.49	14.014
558	24.751	21.936	21.09	19.523	18.347	16.191	13.732
559	24.154	21.463	20.709	19.123	17.921	15.864	13.427
560	23.573	21.055	20.217	18.728	17.556	15.523	13.149
561	23.035	20.568	19.668	18.345	17.158	15.19	12.871
562	22.513	20.103	19.336	17.881	16.787	14.845	12.592
563	21.936	19.641	18.875	17.451	16.437	14.495	12.286
564	21.37	19.083	18.385	17.046	15.971	14.138	12.035
565	20.906	18.646	17.903	16.578	15.618	13.787	11.756
566	20.319	18.161	17.437	16.232	15.274	13.477	11.477
567	19.683	17.69	17.088	15.824	14.83	13.138	11.179
568	19.205	17.18	16.567	15.382	14.45	12.804	10.975
569	18.7	16.682	16.172	14.941	14.099	12.426	10.639
570	18.162	16.239	15.698	14.569	13.702	12.074	10.385
571	17.592	15.79	15.254	14.149	13.287	11.803	10.107
572	17.067	15.268	14.764	13.751	12.903	11.447	9.835
573	16.598	14.852	14.364	13.322	12.513	11.064	9.552
574	15.991	14.355	13.925	12.941	12.111	10.78	9.292
575	15.529	13.932	13.517	12.591	11.81	10.421	8.981
576	15.008	13.431	13.085	12.148	11.439	10.114	8.737
577	14.464	12.993	12.65	11.778	11.082	9.802	8.442
578	13.966	12.555	12.244	11.396	10.742	9.492	8.181
579	13.505	12.093	11.83	11.004	10.369	9.21	7.928
580	12.987	11.708	11.395	10.627	10.009	8.893	7.643
581	12.529	11.276	11.019	10.201	9.666	8.596	7.385
582	12.066	10.848	10.599	9.861	9.29	8.273	7.118
583	11.606	10.429	10.239	9.51	8.983	7.974	6.859
584	11.184	10.028	9.834	9.139	8.629	7.695	6.636
585	10.721	9.637	9.467	8.792	8.313	7.381	6.389
586	10.276	9.24	9.078	8.433	7.989	7.127	6.146
587	9.842	8.86	8.736	8.072	7.674	6.801	5.897
588	9.402	8.499	8.367	7.744	7.357	6.544	5.655
589	9.037	8.148	8.007	7.424	7.045	6.286	5.424
590	8.583	7.798	7.658	7.121	6.771	6.021	5.214
591	8.226	7.423	7.32	6.811	6.455	5.775	5
592	7.85	7.116	7.012	6.52	6.19	5.536	4.785
593	7.476	6.783	6.686	6.212	5.916	5.26	4.583
594	7.145	6.47	6.391	5.922	5.631	5.058	4.356
595	6.785	6.163	6.093	5.657	5.372	4.816	4.156
596	6.456	5.893	5.799	5.393	5.124	4.592	3.967

EXCLI Journal 2020;19:48-70 – ISSN 1611-2156
Received: October 03, 2019, accepted: December 19, 2019, published: January 03, 2020

Emission intensity							
**Wavelength (nm)**	**concentration of acetylshikonin (M)**						
	A	*B*	*C*	*D*	*E*	*F*	*G*
	0.00	$6.0x10^{-6}$	$1.0x10^{-5}$	$1.2x10^{-5}$	$1.4x10^{-5}$	$1.8x10^{-5}$	$2.4x10^{-5}$
597	6.139	5.605	5.536	5.123	4.883	4.37	3.793
598	5.776	5.272	5.242	4.828	4.612	4.14	3.588
599	5.487	5.011	4.96	4.612	4.392	3.944	3.419
600	5.223	4.783	4.745	4.398	4.189	3.765	3.265
601	4.97	4.554	4.517	4.178	4.016	3.607	3.109
602	4.736	4.326	4.314	4.01	3.827	3.441	2.971
603	4.5	4.155	4.108	3.814	3.648	3.287	2.845
604	4.279	3.925	3.915	3.626	3.452	3.138	2.715
605	4.067	3.758	3.745	3.453	3.312	2.996	2.586
606	3.897	3.602	3.552	3.31	3.159	2.874	2.482
607	3.713	3.433	3.404	3.148	3.038	2.752	2.367
608	3.536	3.287	3.239	3.021	2.904	2.633	2.273
609	3.369	3.159	3.113	2.887	2.775	2.533	2.167
610	3.237	3.015	2.983	2.752	2.651	2.418	2.082
611	3.094	2.903	2.856	2.633	2.543	2.314	2
612	2.957	2.774	2.72	2.519	2.44	2.222	1.908
613	2.815	2.666	2.607	2.414	2.341	2.141	1.828
614	2.704	2.559	2.499	2.312	2.25	2.049	1.759
615	2.591	2.445	2.395	2.219	2.157	1.969	1.684
616	2.471	2.351	2.292	2.127	2.073	1.887	1.613
617	2.362	2.252	2.201	2.036	1.979	1.814	1.542
618	2.258	2.152	2.108	1.933	1.903	1.73	1.486
619	2.152	2.077	2.018	1.862	1.821	1.665	1.425
620	2.042	1.974	1.909	1.758	1.741	1.591	1.353

EXCLI Journal 2020;19:48-70 – ISSN 1611-2156
Received: October 03, 2019, accepted: December 19, 2019, published: January 03, 2020

Table 3: Emission intensity in the wavelength range of 380-620 nm for fluorescence emission spectra of Hoechst-DNA fixed concentration (DNA (1.66 x 10^{-5} M) and Hoechst (1.2 x 10^{-5} M)), in the absence (*A*) and presence of increasing concentration of *β*-hydroxyisovalerylshikonin (*B-G*).

Wavelength (nm)	***Emission intensity***						
	**concentration of *β*-hydroxyisovalerylshikonin (M)**						
	A	*B*	*C*	*D*	*E*	*F*	*G*
	0.00	6.0x10^{-6}	1.0x10^{-5}	1.2x10^{-5}	1.4x10^{-5}	1.8x10^{-5}	2.4x10^{-5}
380	4.881	6.25	5.999	5.818	6.969	7.232	6.698
381	4.965	6.301	6.081	5.892	7.073	7.269	6.731
382	5.235	6.556	6.305	6.172	7.408	7.582	7.043
383	5.539	6.83	6.543	6.447	7.741	7.871	7.306
384	5.753	7.091	6.757	6.702	8.095	8.132	7.562
385	6.02	7.314	6.993	6.959	8.418	8.407	7.793
386	6.274	7.532	7.178	7.172	8.701	8.655	8.042
387	6.449	7.744	7.384	7.384	8.978	8.882	8.231
388	6.66	7.931	7.522	7.584	9.269	9.063	8.464
389	6.812	8.094	7.652	7.751	9.482	9.256	8.575
390	6.983	8.238	7.791	7.9	9.734	9.397	8.74
391	7.134	8.364	7.886	8.023	9.906	9.553	8.877
392	7.255	8.473	7.996	8.14	10.056	9.702	8.936
393	7.345	8.553	8.062	8.25	10.18	9.771	9.045
394	7.404	8.621	8.122	8.305	10.291	9.831	9.104
395	7.512	8.705	8.168	8.342	10.4	9.901	9.153
396	7.62	8.726	8.197	8.414	10.472	9.96	9.189
397	7.721	8.83	8.264	8.482	10.526	9.972	9.2
398	7.799	8.882	8.272	8.552	10.604	10.027	9.239
399	7.914	8.885	8.303	8.572	10.654	9.994	9.222
400	8.037	8.959	8.325	8.645	10.654	10.035	9.225
401	8.164	9.037	8.396	8.678	10.76	10.064	9.23
402	8.296	9.116	8.432	8.743	10.84	10.072	9.259
403	8.465	9.194	8.475	8.803	10.877	10.098	9.241
404	8.654	9.284	8.542	8.862	10.951	10.163	9.278
405	8.824	9.406	8.637	8.971	11.04	10.183	9.286
406	9.044	9.57	8.706	9.067	11.098	10.24	9.321
407	9.244	9.659	8.803	9.102	11.176	10.315	9.348
408	9.475	9.849	8.955	9.287	11.323	10.414	9.367
409	9.803	10.044	9.066	9.376	11.406	10.457	9.389
410	10.063	10.249	9.216	9.503	11.526	10.609	9.446
411	10.456	10.497	9.427	9.685	11.716	10.74	9.505
412	10.852	10.816	9.646	9.85	11.877	10.86	9.576
413	11.33	11.121	9.88	10.065	12.046	11.027	9.643
414	11.776	11.468	10.15	10.319	12.271	11.287	9.79
415	12.312	11.854	10.435	10.537	12.473	11.487	9.922
416	12.976	12.295	10.786	10.846	12.744	11.722	10.054
417	13.6	12.78	11.21	11.233	13.058	12.074	10.263
418	14.37	13.408	11.636	11.56	13.395	12.37	10.444
419	15.045	13.93	12.02	11.9	13.751	12.692	10.646
420	15.866	14.557	12.577	12.366	14.193	13.145	10.912
421	16.881	15.366	13.148	12.869	14.672	13.578	11.182
422	17.9	16.109	13.789	13.412	15.205	14.079	11.552
423	19.07	16.981	14.444	14.036	15.776	14.665	11.889

Wavelength (nm)	***Emission intensity***						
	**concentration of β-hydroxyisovalerylshikonin (M)**						
	A	*B*	*C*	*D*	*E*	*F*	*G*
	0.00	$6.0x10^{-6}$	$1.0x10^{-5}$	$1.2x10^{-5}$	$1.4x10^{-5}$	$1.8x10^{-5}$	$2.4x10^{-5}$
424	20.096	17.905	15.247	14.684	16.429	15.296	12.261
425	21.397	18.885	16.045	15.361	17.073	15.934	12.711
426	22.823	19.986	16.875	16.138	17.775	16.655	13.115
427	24.164	21.091	17.751	16.912	18.607	17.376	13.665
428	25.643	22.221	18.703	17.781	19.366	18.174	14.161
429	27.266	23.566	19.769	18.634	20.251	19.052	14.779
430	28.905	24.763	20.778	19.606	21.177	19.895	15.374
431	30.485	26.238	21.883	20.576	22.112	20.727	15.939
432	32.354	27.565	23.011	21.617	23.084	21.762	16.514
433	34.151	29.117	24.272	22.656	24.14	22.753	17.216
434	35.942	30.619	25.401	23.698	25.245	23.745	17.875
435	37.941	32.203	26.727	24.838	26.4	24.863	18.563
436	39.495	33.366	27.716	25.794	27.268	25.661	19.184
437	41.55	35.055	29.002	26.874	28.396	26.793	19.908
438	43.457	36.677	30.427	28.136	29.521	27.883	20.678
439	45.554	38.311	31.799	29.367	30.793	28.909	21.417
440	47.8	40.101	33.211	30.554	31.989	30.174	22.244
441	49.726	41.795	34.686	31.886	33.259	31.377	23.038
442	52.136	43.625	36.132	33.163	34.57	32.502	23.859
443	54.168	45.35	37.517	34.398	35.615	33.699	24.725
444	56.528	47.234	39.072	35.8	37.146	35.173	25.556
445	58.645	48.999	40.721	37.263	38.427	36.242	26.479
446	60.731	50.82	42.178	38.479	39.679	37.476	27.226
447	62.976	52.741	43.562	39.81	40.965	38.755	28.069
448	65.28	54.393	45.155	41.186	42.293	39.829	28.786
449	67.236	56.227	46.524	42.432	43.699	41.119	29.725
450	69.243	57.949	47.944	43.642	45.002	42.5	30.558
451	71.339	59.542	49.413	44.942	46.085	43.707	31.475
452	73.575	61.235	50.958	46.091	47.454	44.792	32.238
453	75.572	62.969	52.18	47.583	48.676	46.03	33.098
454	77.055	64.333	53.269	48.469	49.6	46.831	33.617
455	78.846	66.161	54.793	49.778	50.818	47.943	34.498
456	80.739	67.689	55.968	50.855	52.128	48.909	35.25
457	82.521	69	57.118	52.01	53.31	50.246	35.967
458	84.13	70.714	58.471	53.159	54.221	51.139	36.647
459	86.238	72.049	59.664	54.293	55.542	52.156	37.376
460	87.502	73.257	60.972	55.319	56.391	53.175	38.088
461	89.425	74.854	62.081	56.32	57.562	54.162	38.685
462	90.43	76.059	63.112	57.41	58.445	55.021	39.461
463	92.293	77.416	63.953	58.447	59.25	55.928	40.004
464	93.738	78.282	65.298	59.373	60.271	56.609	40.494
465	94.487	79.569	65.947	60.04	61.061	57.497	41.064
466	96.007	80.65	67.035	60.991	62	58.278	41.686
467	97.121	81.458	67.897	61.563	62.596	58.877	42.044
468	98.058	82.485	68.649	62.378	63.263	59.469	42.661
469	99.104	83.175	69.441	63.202	63.969	60.094	43.037
470	99.941	83.975	70.041	63.633	64.656	60.716	43.437
471	101.129	84.572	70.824	64.426	64.981	61.172	43.759

Wavelength (nm)	***Emission intensity***						
	**concentration of β-hydroxyisovalerylshikonin (M)**						
	A	*B*	*C*	*D*	*E*	*F*	*G*
	0.00	$6.0x10^{-6}$	$1.0x10^{-5}$	$1.2x10^{-5}$	$1.4x10^{-5}$	$1.8x10^{-5}$	$2.4x10^{-5}$
472	101.37	85.376	71.2	64.739	65.485	61.682	44.022
473	102.002	85.655	71.771	64.968	66.001	62.159	44.122
474	102.716	86.289	72.058	65.54	66.335	62.21	44.436
475	103.107	86.757	72.57	65.799	66.794	62.674	44.717
476	103.517	87.099	72.864	66.336	66.996	63.066	44.932
477	104.077	87.437	73.32	66.356	67.438	63.3	45.167
478	104.387	87.776	73.513	66.693	67.447	63.506	45.201
479	104.124	87.978	73.618	67.105	67.697	63.507	45.576
480	104.739	88.093	73.781	66.926	67.92	63.746	45.608
481	104.469	88.137	73.779	67.144	67.846	63.782	45.726
482	104.828	88.175	73.928	67.031	67.917	63.888	45.692
483	104.635	87.958	73.951	67.047	67.938	63.845	45.603
484	104.197	87.851	73.619	67.152	67.735	63.589	45.495
485	104.091	87.852	73.703	67.062	67.723	63.64	45.379
486	103.51	87.281	73.577	66.718	67.492	63.459	45.315
487	103.311	87.25	73.288	66.521	67.227	63.199	45.384
488	102.861	86.655	72.956	66.301	66.973	63.13	44.92
489	102.071	86.257	72.701	66.004	66.632	62.867	44.946
490	101.586	85.642	72.229	65.871	66.353	62.382	44.588
491	100.909	85.224	71.685	65.416	66.044	62.077	44.308
492	100.126	84.655	71.389	65.017	65.43	61.557	44.068
493	99.278	83.725	70.921	64.512	65.029	61.175	43.69
494	98.342	82.947	70.533	64.052	64.405	60.607	43.342
495	97.51	82.346	69.888	63.538	63.856	60.223	42.96
496	96.766	81.557	69.354	62.982	63.468	59.844	42.681
497	95.684	80.558	68.468	62.455	62.84	59.013	42.253
498	94.905	79.857	67.782	61.943	62.099	58.32	41.903
499	93.664	79.022	67.248	61.101	61.326	57.934	41.304
500	92.766	77.88	66.481	60.732	60.908	57.247	40.919
501	91.303	77.017	65.637	59.936	60.153	56.4	40.534
502	90.44	75.952	64.724	59.211	59.454	55.754	40.04
503	89.296	75.129	64.346	58.76	58.768	55.175	39.633
504	88.244	74.032	63.449	57.849	58.189	54.462	39.139
505	86.947	73.297	62.601	57.157	57.294	53.799	38.662
506	85.576	72.017	61.804	56.51	56.608	52.869	37.982
507	84.348	70.809	60.846	55.538	55.683	52.293	37.482
508	82.826	69.969	59.983	54.786	54.959	51.337	36.78
509	81.517	68.559	58.987	53.956	54.036	50.443	36.406
510	79.919	67.727	58.021	53.14	53.172	49.651	35.655
511	78.806	66.545	56.973	52.199	52.337	48.765	35.079
512	77.379	65.52	56.18	51.465	51.47	48.048	34.652
513	75.891	64.068	55.194	50.461	50.631	47.15	33.985
514	74.573	62.908	54.211	49.518	49.687	46.465	33.383
515	73.004	61.654	53.168	48.668	48.919	45.623	32.836
516	71.567	60.623	52.253	47.828	47.902	44.647	32.281
517	70.197	59.333	51.317	46.936	46.871	43.746	31.616
518	68.798	58.212	50.149	45.975	46.107	42.914	31.199
519	67.476	57.161	49.228	45.111	45.163	42.097	30.576

EXCLI Journal 2020;19:48-70 – ISSN 1611-2156
Received: October 03, 2019, accepted: December 19, 2019, published: January 03, 2020

Wavelength (nm)	***Emission intensity***						
	**concentration of β-hydroxyisovalerylshikonin (M)**						
	A	*B*	*C*	*D*	*E*	*F*	*G*
	0.00	$6.0x10^{-6}$	$1.0x10^{-5}$	$1.2x10^{-5}$	$1.4x10^{-5}$	$1.8x10^{-5}$	$2.4x10^{-5}$
520	65.844	56.003	48.156	44.304	44.291	41.05	29.937
521	64.659	54.772	47.266	43.36	43.446	40.223	29.246
522	63.137	53.707	46.173	42.37	42.543	39.502	28.615
523	61.672	52.445	45.196	41.62	41.553	38.567	27.987
524	60.227	51.377	44.217	40.652	40.638	37.64	27.318
525	58.835	50.04	43.102	39.795	39.787	36.869	26.778
526	57.66	49.094	42.154	38.854	38.894	36.028	26.1
527	56.193	47.842	41.01	38.07	38.041	35.273	25.461
528	55.042	46.794	40.156	37.169	37.132	34.479	24.931
529	53.603	45.737	39.247	36.275	36.33	33.652	24.333
530	52.389	44.659	38.382	35.613	35.433	32.83	23.724
531	51.027	43.694	37.472	34.627	34.622	31.987	23.197
532	49.652	42.41	36.433	33.906	33.728	31.25	22.585
533	48.417	41.384	35.566	32.994	32.869	30.598	22.092
534	47.113	40.391	34.728	32.23	32.18	29.79	21.546
535	45.979	39.212	33.68	31.456	31.284	28.959	20.987
536	44.652	38.257	32.935	30.565	30.469	28.303	20.495
537	43.455	37.183	32.057	29.865	29.768	27.531	20.014
538	42.369	36.214	31.225	29.096	29.016	26.838	19.536
539	41.211	35.282	30.465	28.341	28.238	26.19	18.992
540	40.057	34.438	29.663	27.572	27.586	25.397	18.509
541	38.972	33.533	28.908	26.935	26.86	24.847	18.096
542	38.085	32.761	28.216	26.268	26.288	24.201	17.658
543	36.967	32.001	27.424	25.689	25.636	23.57	17.259
544	36.121	31.267	26.788	25.067	24.99	22.898	16.839
545	35.182	30.45	26.158	24.484	24.441	22.453	16.485
546	34.344	29.759	25.556	23.923	23.887	22.004	16.103
547	33.589	29.027	24.943	23.39	23.327	21.505	15.759
548	32.754	28.379	24.469	22.89	22.901	20.981	15.393
549	31.985	27.742	23.857	22.373	22.367	20.604	15.089
550	31.356	27.121	23.303	21.97	21.835	20.07	14.767
551	30.557	26.504	22.749	21.48	21.384	19.578	14.526
552	29.976	25.997	22.281	20.999	20.931	19.227	14.259
553	29.13	25.308	21.811	20.536	20.546	18.793	13.965
554	28.607	24.786	21.379	20.11	20.08	18.475	13.671
555	27.973	24.25	20.876	19.686	19.683	18.067	13.416
556	27.272	23.717	20.394	19.205	19.245	17.583	13.135
557	26.649	23.195	19.997	18.874	18.791	17.298	12.888
558	26.091	22.623	19.475	18.396	18.407	16.908	12.646
559	25.34	22.171	19.071	17.978	17.977	16.543	12.391
560	24.833	21.654	18.709	17.609	17.623	16.16	12.127
561	24.237	21.177	18.245	17.191	17.246	15.775	11.829
562	23.656	20.704	17.863	16.824	16.826	15.463	11.584
563	23.025	20.207	17.483	16.447	16.46	15.109	11.315
564	22.406	19.671	17.001	16.018	16.027	14.77	11.047
565	21.89	19.163	16.55	15.619	15.663	14.402	10.838
566	21.238	18.667	16.159	15.284	15.225	14.019	10.562
567	20.699	18.261	15.706	14.9	14.883	13.681	10.27

Wavelength (nm)	**Emission intensity**						
	**concentration of β-hydroxyisovalerylshikonin (M)**						
	A	*B*	*C*	*D*	*E*	*F*	*G*
	0.00	$6.0x10^{-6}$	$1.0x10^{-5}$	$1.2x10^{-5}$	$1.4x10^{-5}$	$1.8x10^{-5}$	$2.4x10^{-5}$
568	20.102	17.723	15.341	14.501	14.499	13.307	10.08
569	19.624	17.256	14.905	14.095	14.147	12.902	9.77
570	18.963	16.794	14.467	13.713	13.758	12.606	9.505
571	18.378	16.281	13.992	13.351	13.297	12.274	9.271
572	17.777	15.743	13.563	12.908	12.953	11.827	9.011
573	17.238	15.316	13.19	12.559	12.564	11.51	8.779
574	16.727	14.865	12.768	12.228	12.177	11.189	8.498
575	16.124	14.373	12.383	11.797	11.855	10.843	8.262
576	15.606	13.934	11.987	11.42	11.46	10.495	8.014
577	15.064	13.433	11.555	11.098	11.081	10.189	7.727
578	14.533	12.996	11.201	10.7	10.742	9.854	7.516
579	14.014	12.558	10.833	10.365	10.363	9.505	7.256
580	13.516	12.108	10.454	9.975	10.025	9.193	7.007
581	12.973	11.697	10.089	9.65	9.661	8.849	6.78
582	12.402	11.262	9.709	9.29	9.28	8.563	6.542
583	11.931	10.834	9.353	8.986	8.957	8.221	6.331
584	11.463	10.414	9.022	8.619	8.635	7.93	6.076
585	10.973	9.995	8.642	8.307	8.317	7.608	5.851
586	10.5	9.607	8.304	7.978	7.986	7.319	5.638
587	10.047	9.224	7.96	7.674	7.677	7.026	5.407
588	9.588	8.83	7.629	7.324	7.337	6.714	5.191
589	9.131	8.448	7.298	7.035	7.015	6.443	4.957
590	8.717	8.091	6.986	6.755	6.726	6.153	4.771
591	8.305	7.728	6.687	6.433	6.412	5.879	4.548
592	7.924	7.382	6.38	6.164	6.154	5.633	4.375
593	7.523	7.025	6.097	5.889	5.895	5.361	4.188
594	7.167	6.693	5.812	5.612	5.637	5.129	3.996
595	6.756	6.374	5.558	5.341	5.342	4.903	3.82
596	6.403	6.064	5.283	5.098	5.118	4.651	3.645
597	6.078	5.794	5.051	4.862	4.879	4.442	3.484
598	5.716	5.452	4.741	4.579	4.588	4.204	3.31
599	5.386	5.19	4.543	4.361	4.363	4.001	3.158
600	5.107	4.966	4.328	4.155	4.181	3.836	3.018
601	4.838	4.713	4.138	3.961	3.981	3.643	2.875
602	4.598	4.498	3.952	3.786	3.794	3.474	2.746
603	4.338	4.286	3.779	3.606	3.611	3.314	2.626
604	4.117	4.092	3.572	3.429	3.457	3.175	2.513
605	3.89	3.886	3.426	3.288	3.294	3.02	2.406
606	3.682	3.715	3.285	3.128	3.141	2.887	2.298
607	3.493	3.56	3.134	2.988	3.008	2.769	2.206
608	3.299	3.4	3.005	2.861	2.877	2.649	2.11
609	3.133	3.244	2.858	2.735	2.759	2.531	2.032
610	2.972	3.093	2.75	2.615	2.646	2.44	1.943
611	2.832	2.971	2.636	2.509	2.52	2.336	1.877
612	2.68	2.854	2.526	2.393	2.412	2.235	1.788
613	2.535	2.71	2.425	2.295	2.311	2.151	1.729
614	2.386	2.599	2.328	2.217	2.23	2.069	1.661
615	2.259	2.5	2.233	2.116	2.125	1.97	1.601

EXCLI Journal 2020;19:48-70 – ISSN 1611-2156
Received: October 03, 2019, accepted: December 19, 2019, published: January 03, 2020

Emission intensity							
**Wavelength (nm)**	**concentration of *β*-hydroxyisovalerylshikonin (M)**						
	A	*B*	*C*	*D*	*E*	*F*	*G*
	0.00	$6.0x10^{-6}$	$1.0x10^{-5}$	$1.2x10^{-5}$	$1.4x10^{-5}$	$1.8x10^{-5}$	$2.4x10^{-5}$
616	2.14	2.38	2.142	2.027	2.048	1.905	1.546
617	2.018	2.28	2.056	1.95	1.971	1.827	1.491
618	1.931	2.199	1.983	1.857	1.887	1.765	1.436
619	1.809	2.111	1.904	1.795	1.814	1.679	1.379
620	1.68	1.997	1.808	1.693	1.723	1.608	1.318

EXCLI Journal 2020;19:48-70 – ISSN 1611-2156
Received: October 03, 2019, accepted: December 19, 2019, published: January 03, 2020

Raw tables concerning **Figure 9.** Absorption spectra of HSA (2.00 x 10^{-6} M) before (purple dashed line) and after addition of α-methylbutyrylshikon (***1***), acetylshikonin (***2***) and *β*-hydroxyisovalerylshikonin (***3***) (0 - 1.60 x 10^{-5} M). Arrow shows the absorbance changes upon increasing concentration of α-methylbutyrylshikon (***1***), acetylshikonin (***2***) and *β*-hydroxyisovalerylshikonin (***3***).

Table 1: Absorption intensity in the wavelength range of 200-800 nm for absorption spectra of HSA fixed concentration (2.00 x 10^{-6} M), in the absence (*A*) and presence of increasing concentration of α–methylbutyrylshikon (*B*-*L*)

Wavelength (nm)	***Absorption intensity***										
	**concentration of α-methylbutyrylshikon (M)**										
	A	*B*	*C*	*D*	*E*	*F*	*G*	*I*	*J*	*K*	*L*
	0.00	8.0x10^{-7}	1.6x10^{-6}	2.0x10^{-6}	2.8x10^{-6}	4.0x10^{-6}	4.8x10^{-6}	6.0x10^{-6}	8.0x10^{-6}	1.2x10^{-5}	1.6x10^{-5}
800	0.00316	0.007383	0.01529	0.003885	0.005269	0.012191	0.003354	0.005666	0.009515	0.003937	0.007092
799	0.003996	0.00831	0.016403	0.004911	0.006372	0.012783	0.004066	0.006595	0.010371	0.005077	0.008178
798	0.003903	0.008342	0.016302	0.004757	0.00645	0.012836	0.004167	0.00649	0.010674	0.005166	0.007789
797	0.00435	0.008171	0.016081	0.004862	0.006203	0.01294	0.004374	0.00672	0.01062	0.004995	0.0079
796	0.004454	0.008332	0.016434	0.004812	0.006437	0.012859	0.004418	0.006709	0.010607	0.00516	0.008178
795	0.003996	0.008085	0.01627	0.004772	0.006498	0.012792	0.004399	0.006775	0.010824	0.005113	0.008284
794	0.00423	0.008244	0.016368	0.00492	0.006615	0.013094	0.004537	0.006585	0.010628	0.005171	0.008132
793	0.004162	0.008414	0.016125	0.004772	0.00652	0.012772	0.00447	0.006514	0.010468	0.005167	0.008188
792	0.00405	0.008155	0.016407	0.004756	0.006642	0.013086	0.004276	0.007014	0.010557	0.00535	0.008193
791	0.004161	0.008553	0.01642	0.00492	0.006212	0.013093	0.004408	0.007014	0.010731	0.005354	0.008637
790	0.004096	0.008311	0.016573	0.005112	0.006343	0.013104	0.004288	0.00668	0.010927	0.005025	0.008287
789	0.004068	0.008562	0.016523	0.004879	0.006321	0.013286	0.004458	0.006721	0.010814	0.005165	0.008229
788	0.004426	0.008683	0.016373	0.005056	0.006444	0.012897	0.004461	0.006688	0.010689	0.005304	0.008615
787	0.004495	0.00845	0.016557	0.004787	0.006393	0.013223	0.004449	0.006801	0.010872	0.005202	0.008513
786	0.004119	0.008385	0.016356	0.004667	0.006368	0.013329	0.004361	0.006807	0.010762	0.005251	0.008724
785	0.00431	0.008549	0.01662	0.004731	0.006352	0.013107	0.004619	0.00696	0.010871	0.00535	0.008252
784	0.004103	0.008303	0.016714	0.004875	0.006371	0.013148	0.00444	0.006806	0.010652	0.005196	0.008385
783	0.004103	0.008606	0.016311	0.004787	0.006485	0.013458	0.004205	0.006881	0.010951	0.005439	0.008439
782	0.0043	0.008241	0.016763	0.004831	0.006568	0.013286	0.004492	0.006682	0.011018	0.005578	0.008355
781	0.003962	0.008539	0.016599	0.004711	0.006481	0.013477	0.004316	0.006713	0.010826	0.005362	0.008533
780	0.004115	0.008424	0.016433	0.004917	0.006223	0.01348	0.004524	0.006784	0.010933	0.005443	0.008445
779	0.004133	0.008477	0.016542	0.004789	0.006297	0.0133	0.004605	0.006776	0.010711	0.005381	0.008573
778	0.004151	0.008507	0.016505	0.004799	0.006546	0.013135	0.00447	0.006815	0.010838	0.005209	0.008612
777	0.004201	0.008742	0.016361	0.005008	0.006301	0.013475	0.004253	0.007174	0.010981	0.005253	0.008746
776	0.004132	0.008547	0.016431	0.004982	0.006708	0.013292	0.004303	0.006869	0.011082	0.005129	0.008699
775	0.004125	0.008558	0.016549	0.004984	0.006377	0.013358	0.00454	0.00683	0.010932	0.005262	0.008452
774	0.004373	0.008463	0.016809	0.004951	0.006615	0.013443	0.004562	0.006919	0.011019	0.005327	0.008581
773	0.004231	0.008565	0.016658	0.005018	0.006565	0.013693	0.004521	0.006742	0.011006	0.005307	0.008818

EXCLI Journal 2020;19:48-70 – ISSN 1611-2156
Received: October 03, 2019, accepted: December 19, 2019, published: January 03, 2020

Absorption intensity											
**Wavelength (nm)**	**concentration of α-methylbutyrylshikon (M)**										
	A	*B*	*C*	*D*	*E*	*F*	*G*	*I*	*J*	*K*	*L*
	0.00	$8.0x10^{-7}$	$1.6x10^{-6}$	$2.0x10^{-6}$	$2.8x10^{-6}$	$4.0x10^{-6}$	$4.8x10^{-6}$	$6.0x10^{-6}$	$8.0x10^{-6}$	$1.2x10^{-5}$	$1.6x10^{-5}$
772	0.004196	0.008555	0.016768	0.004654	0.006281	0.013465	0.004456	0.007153	0.010928	0.005474	0.008798
771	0.004045	0.008405	0.016742	0.004823	0.006557	0.013287	0.004362	0.006939	0.010877	0.005478	0.008592
770	0.004159	0.008501	0.016564	0.004971	0.006285	0.013462	0.00456	0.006975	0.01068	0.005407	0.008758
769	0.004143	0.008719	0.016767	0.004708	0.006585	0.013588	0.004245	0.006968	0.010895	0.005364	0.008923
768	0.00406	0.008462	0.016757	0.004642	0.006531	0.013671	0.004461	0.00693	0.011055	0.00527	0.00876
767	0.004102	0.008705	0.016747	0.004909	0.006319	0.013425	0.004386	0.006892	0.011115	0.005418	0.008792
766	0.003893	0.008521	0.016689	0.004766	0.006723	0.013628	0.004331	0.006863	0.011174	0.005397	0.008836
765	0.004118	0.008258	0.016748	0.004739	0.006373	0.013727	0.004391	0.006798	0.011156	0.005365	0.008707
764	0.004166	0.008456	0.016912	0.004954	0.006471	0.01352	0.004474	0.007085	0.011258	0.005443	0.008652
763	0.004188	0.00858	0.016691	0.004907	0.006588	0.013681	0.004433	0.006758	0.011387	0.005421	0.009014
762	0.004257	0.008597	0.01686	0.004663	0.00654	0.013695	0.004407	0.007236	0.011228	0.005591	0.008859
761	0.004097	0.008421	0.016814	0.004713	0.006522	0.013711	0.004395	0.006899	0.011304	0.0054	0.008754
760	0.004062	0.008879	0.016933	0.004904	0.006513	0.013757	0.004354	0.007057	0.011259	0.005602	0.009196
759	0.004284	0.008866	0.017159	0.004729	0.006814	0.013879	0.004562	0.007045	0.011382	0.005618	0.009094
758	0.004081	0.008736	0.017095	0.00498	0.006669	0.013806	0.00441	0.006982	0.011431	0.005385	0.009184
757	0.004017	0.008422	0.016952	0.004857	0.006559	0.013835	0.004458	0.006993	0.011237	0.005448	0.008993
756	0.004012	0.008681	0.01703	0.004753	0.006584	0.013791	0.004368	0.00701	0.011319	0.005379	0.009275
755	0.003945	0.008597	0.016855	0.004715	0.006641	0.013872	0.00444	0.006903	0.0114	0.005427	0.009202
754	0.004049	0.008748	0.017063	0.004851	0.006572	0.014019	0.004639	0.006997	0.011578	0.005525	0.009244
753	0.00407	0.008453	0.01709	0.004955	0.006429	0.01389	0.004506	0.007319	0.011597	0.005629	0.009273
752	0.004295	0.008666	0.017055	0.004641	0.00674	0.013957	0.004383	0.007199	0.01137	0.005717	0.00924
751	0.004178	0.008852	0.017111	0.005022	0.00684	0.01411	0.004636	0.007325	0.011635	0.005603	0.009221
750	0.004237	0.008892	0.017033	0.004845	0.006585	0.014077	0.004591	0.007211	0.01149	0.005746	0.00938
749	0.00421	0.008614	0.017122	0.004668	0.006593	0.014046	0.004315	0.007177	0.01163	0.005708	0.009509
748	0.004111	0.00886	0.01719	0.004862	0.006792	0.013906	0.004668	0.007294	0.011625	0.005817	0.009409
747	0.004192	0.00879	0.017142	0.004917	0.00661	0.014069	0.004614	0.007335	0.011463	0.005873	0.009583
746	0.004092	0.008842	0.017235	0.004798	0.006755	0.013999	0.004568	0.007086	0.011698	0.005777	0.009571
745	0.00417	0.008739	0.017248	0.004709	0.006685	0.014102	0.004599	0.007266	0.011656	0.005875	0.009426
744	0.004017	0.008749	0.01727	0.004787	0.006591	0.01425	0.004561	0.007381	0.011625	0.005737	0.009614
743	0.004081	0.008729	0.017347	0.004677	0.00674	0.014234	0.004595	0.007309	0.01188	0.005824	0.009633
742	0.00415	0.008742	0.017213	0.004752	0.006827	0.014028	0.004593	0.007513	0.011839	0.005955	0.009668
741	0.004097	0.008715	0.017248	0.004685	0.006685	0.014098	0.004461	0.007387	0.011787	0.005731	0.009855
740	0.00401	0.008838	0.017228	0.004728	0.006625	0.014469	0.004746	0.007529	0.011882	0.005953	0.00993
739	0.004248	0.008632	0.017251	0.004619	0.006568	0.014271	0.00472	0.007339	0.011793	0.006017	0.009659
738	0.004212	0.008745	0.017193	0.00491	0.00691	0.014377	0.004612	0.007539	0.011968	0.00621	0.009969
737	0.003926	0.008764	0.017233	0.004829	0.006864	0.014322	0.004774	0.007544	0.011878	0.006192	0.010178
736	0.004092	0.008796	0.017353	0.004597	0.006819	0.014402	0.004729	0.007526	0.012028	0.006134	0.010183

**Absorption intensity**											
**Wavelength (nm)**	**concentration of α-methylbutyrylshikon (M)**										
	A	*B*	*C*	*D*	*E*	*F*	*G*	*I*	*J*	*K*	*L*
	0.00	$8.0x10^{-7}$	$1.6x10^{-6}$	$2.0x10^{-6}$	$2.8x10^{-6}$	$4.0x10^{-6}$	$4.8x10^{-6}$	$6.0x10^{-6}$	$8.0x10^{-6}$	$1.2x10^{-5}$	$1.6x10^{-5}$
735	0.004055	0.008796	0.017297	0.005008	0.006726	0.014635	0.004622	0.007522	0.01196	0.006354	0.010335
734	0.004127	0.008877	0.017561	0.004976	0.006821	0.014465	0.004694	0.007633	0.012016	0.00638	0.010436
733	0.003922	0.008678	0.017501	0.004675	0.006911	0.01465	0.004839	0.007551	0.012211	0.006385	0.01039
732	0.003998	0.008777	0.017333	0.00476	0.006757	0.014542	0.004773	0.007666	0.012063	0.006465	0.010564
731	0.003858	0.00884	0.017378	0.004646	0.006685	0.01474	0.00475	0.007748	0.012153	0.006586	0.010847
730	0.003891	0.008643	0.017487	0.00466	0.006842	0.014568	0.004677	0.00774	0.012267	0.006559	0.010873
729	0.00376	0.008654	0.017384	0.004677	0.006557	0.014648	0.004729	0.007543	0.012237	0.00641	0.010947
728	0.00385	0.008483	0.017188	0.004608	0.006713	0.014521	0.004683	0.007621	0.012275	0.006475	0.011062
727	0.003753	0.00849	0.017055	0.00449	0.00665	0.01452	0.004638	0.00751	0.012318	0.006622	0.011096
726	0.003564	0.008404	0.017143	0.004582	0.006389	0.014741	0.004589	0.007425	0.012335	0.006721	0.011028
725	0.003422	0.008346	0.016974	0.00434	0.006432	0.014477	0.004328	0.007401	0.012128	0.006442	0.010991
724	0.003255	0.008085	0.016925	0.004084	0.006157	0.014328	0.004274	0.007264	0.012035	0.006418	0.010995
723	0.003226	0.0082	0.016927	0.003986	0.006179	0.014224	0.004192	0.007309	0.011932	0.006168	0.01098
722	0.002973	0.007885	0.016695	0.003925	0.005904	0.014266	0.004006	0.007086	0.011868	0.006279	0.010711
721	0.002579	0.00766	0.016444	0.003639	0.005883	0.014068	0.003902	0.006833	0.011864	0.006061	0.010589
720	0.00276	0.007515	0.016362	0.003473	0.005766	0.013972	0.003826	0.006742	0.011563	0.006081	0.010541
719	0.002436	0.007392	0.016158	0.003388	0.005517	0.01374	0.003452	0.006715	0.011536	0.005748	0.010368
718	0.002524	0.007409	0.016066	0.003535	0.005548	0.013888	0.0037	0.006694	0.011517	0.005888	0.010875
717	0.002381	0.007449	0.016167	0.003435	0.005436	0.013797	0.003583	0.006627	0.011732	0.005864	0.010755
716	0.002246	0.007101	0.015985	0.003345	0.005394	0.013691	0.00341	0.006373	0.011454	0.005664	0.010601
715	0.00194	0.006869	0.015895	0.003096	0.005151	0.01351	0.00313	0.006255	0.011334	0.005499	0.010577
714	0.001847	0.00677	0.015721	0.002801	0.004909	0.013449	0.003143	0.006175	0.011264	0.005594	0.010386
713	0.001758	0.006687	0.015487	0.002783	0.004822	0.013266	0.003033	0.006059	0.011177	0.005498	0.010408
712	0.001347	0.006502	0.015448	0.002569	0.004751	0.013272	0.002848	0.005854	0.01091	0.005428	0.010346
711	0.001476	0.006297	0.01543	0.002381	0.004589	0.013261	0.002744	0.005926	0.010872	0.00533	0.010265
710	0.001366	0.00624	0.015245	0.00227	0.004582	0.013107	0.002485	0.005724	0.010907	0.005354	0.01028
709	0.001172	0.006009	0.01524	0.002277	0.00443	0.013169	0.00246	0.005773	0.010754	0.005199	0.01034
708	0.00112	0.00606	0.015189	0.002161	0.004424	0.013227	0.002442	0.005575	0.010959	0.00531	0.010427
707	0.000912	0.006118	0.015075	0.001964	0.004377	0.013169	0.002464	0.00568	0.010815	0.005315	0.010379
706	0.000735	0.005933	0.014897	0.002181	0.004283	0.013144	0.002308	0.005657	0.010938	0.005186	0.01066
705	0.000889	0.005821	0.014819	0.002054	0.004298	0.012833	0.002354	0.005537	0.010545	0.005278	0.010284
704	0.000956	0.006042	0.015027	0.00204	0.004294	0.013034	0.002305	0.005638	0.010889	0.005269	0.01071
703	0.000849	0.005982	0.015096	0.002171	0.004379	0.013345	0.002376	0.005708	0.011147	0.005468	0.010915
702	0.000799	0.006182	0.01526	0.002279	0.004384	0.013278	0.002467	0.005767	0.011147	0.005607	0.011191
701	0.000875	0.006305	0.015333	0.002248	0.004575	0.013419	0.002739	0.005959	0.011388	0.00569	0.011324
700	0.001037	0.006258	0.015408	0.002448	0.004526	0.013585	0.002729	0.006034	0.011321	0.005825	0.011471
699	0.001059	0.006338	0.015579	0.002342	0.004782	0.013676	0.002637	0.006118	0.011592	0.00589	0.011505

EXCLI Journal 2020;19:48-70 – ISSN 1611-2156
Received: October 03, 2019, accepted: December 19, 2019, published: January 03, 2020

Absorption intensity											
**Wavelength (nm)**	**concentration of α-methylbutyrylshikon (M)**										
	A	*B*	*C*	*D*	*E*	*F*	*G*	*I*	*J*	*K*	*L*
	0.00	$8.0x10^{-7}$	$1.6x10^{-6}$	$2.0x10^{-6}$	$2.8x10^{-6}$	$4.0x10^{-6}$	$4.8x10^{-6}$	$6.0x10^{-6}$	$8.0x10^{-6}$	$1.2x10^{-5}$	$1.6x10^{-5}$
698	0.001134	0.006312	0.015686	0.002428	0.004799	0.013867	0.002868	0.006161	0.011687	0.006193	0.011791
697	0.001251	0.006308	0.015572	0.002564	0.004928	0.013932	0.002775	0.006132	0.011715	0.006178	0.011969
696	0.001289	0.006571	0.015669	0.002408	0.004961	0.014195	0.00302	0.006515	0.012066	0.006438	0.012062
695	0.001259	0.006676	0.015961	0.002681	0.005029	0.014059	0.003184	0.006754	0.012077	0.006645	0.012297
694	0.001515	0.006833	0.016009	0.002648	0.005221	0.014348	0.003253	0.006731	0.01232	0.006621	0.012731
693	0.001345	0.00676	0.016051	0.002799	0.005265	0.014503	0.003269	0.006761	0.012286	0.006855	0.012739
692	0.001585	0.006951	0.01626	0.003	0.005461	0.014533	0.003335	0.006896	0.012458	0.007033	0.012927
691	0.001576	0.007055	0.016256	0.002961	0.005639	0.014801	0.003448	0.007056	0.01269	0.007227	0.013147
690	0.00163	0.007055	0.016316	0.003007	0.005567	0.014885	0.003524	0.007129	0.012765	0.007235	0.013539
689	0.00166	0.007121	0.016506	0.003091	0.005528	0.014924	0.003612	0.007321	0.012942	0.007534	0.013638
688	0.001676	0.00726	0.016579	0.003222	0.005789	0.014996	0.003599	0.007319	0.012892	0.007495	0.013814
687	0.00171	0.00724	0.016593	0.003198	0.005875	0.015233	0.003686	0.007493	0.013107	0.007693	0.013954
686	0.001815	0.00729	0.016602	0.003274	0.005929	0.015383	0.003904	0.007683	0.013307	0.007903	0.014135
685	0.001645	0.007494	0.016736	0.003323	0.005922	0.015323	0.00379	0.007518	0.01347	0.007974	0.014425
684	0.001703	0.00728	0.016695	0.003406	0.005969	0.277532	0.003879	0.007634	0.013619	0.008094	0.014595
683	0.001768	0.007121	0.016394	0.003276	0.005839	0.015333	0.003741	0.007611	0.01317	0.007913	0.01444
682	0.001907	0.007416	0.016536	0.003668	0.006066	0.01534	0.004008	0.00784	0.013581	0.008515	0.014736
681	0.001738	0.007432	0.016944	0.003429	0.006222	0.015687	0.004064	0.00794	0.013815	0.008488	0.015172
680	0.001812	0.007589	0.017009	0.003292	0.00622	0.015921	0.004336	0.008107	0.013976	0.008577	0.015244
679	0.001937	0.007606	0.017093	0.003548	0.00617	0.016059	0.004217	0.008199	0.014075	0.008725	0.015648
678	0.001836	0.007575	0.016909	0.003556	0.006321	0.016064	0.004175	0.00831	0.01412	0.008971	0.015753
677	0.002024	0.007673	0.017131	0.003637	0.006255	0.016165	0.004497	0.008381	0.014202	0.009066	0.015971
676	0.001887	0.007716	0.017202	0.003663	0.006371	0.01623	0.004397	0.008334	0.014446	0.009283	0.016027
675	0.001933	0.007674	0.017178	0.003604	0.006399	0.016465	0.004407	0.008496	0.014415	0.009287	0.016179
674	0.001926	0.007744	0.017178	0.003561	0.006571	0.01654	0.004476	0.008503	0.014579	0.009452	0.016561
673	0.002048	0.007669	0.017277	0.003497	0.00644	0.016648	0.004467	0.008794	0.014681	0.009639	0.016629
672	0.001913	0.007828	0.017378	0.003822	0.006756	0.016653	0.004626	0.008811	0.014864	0.009598	0.016853
671	0.002032	0.00785	0.017464	0.003799	0.006712	0.016934	0.004522	0.008754	0.015012	0.009981	0.017177
670	0.002388	0.008193	0.017821	0.004176	0.006893	0.017245	0.005048	0.00917	0.015422	0.010514	0.017652
669	0.002597	0.008445	0.018227	0.004631	0.007394	0.017594	0.005515	0.009538	0.015812	0.010832	0.018028
668	0.002816	0.008632	0.018341	0.004525	0.00753	0.018079	0.005695	0.00978	0.015997	0.011114	0.018554
667	0.002432	0.008283	0.018015	0.004177	0.007163	0.017632	0.005239	0.009511	0.015619	0.01093	0.018322
666	0.002176	0.008176	0.017739	0.004135	0.007123	0.017415	0.0051	0.009336	0.015722	0.010853	0.018296
665	0.002065	0.007872	0.017781	0.004009	0.0069	0.017425	0.005102	0.009209	0.01561	0.010831	0.018368
664	0.001974	0.007866	0.017653	0.004055	0.006786	0.017522	0.005088	0.009346	0.015782	0.010953	0.018637
663	0.001868	0.007762	0.017684	0.003844	0.006749	0.017367	0.004921	0.009202	0.015781	0.010908	0.018837
662	0.001814	0.007854	0.017668	0.003909	0.006722	0.017621	0.005106	0.009221	0.015782	0.011049	0.018905

Received: October 03, 2019, accepted: December 19, 2019, published: January 03, 2020

Absorption intensity											
**Wavelength (nm)**	**concentration of α-methylbutyrylshikon (M)**										
	A	*B*	*C*	*D*	*E*	*F*	*G*	*I*	*J*	*K*	*L*
	0.00	$8.0x10^{-7}$	$1.6x10^{-6}$	$2.0x10^{-6}$	$2.8x10^{-6}$	$4.0x10^{-6}$	$4.8x10^{-6}$	$6.0x10^{-6}$	$8.0x10^{-6}$	$1.2x10^{-5}$	$1.6x10^{-5}$
661	0.001768	0.007811	0.0176	0.003777	0.006759	0.017556	0.004804	0.009298	0.015886	0.011116	0.018918
660	0.001551	0.007731	0.017489	0.003731	0.006641	0.017629	0.004904	0.009321	0.01592	0.011161	0.019229
659	0.001544	0.007708	0.017457	0.003777	0.0067	0.017784	0.004724	0.009281	0.015861	0.011284	0.019282
658	0.001526	0.007659	0.017598	0.003674	0.006707	0.017587	0.004807	0.009493	0.016127	0.011422	0.019537
657	0.001483	0.00754	0.017548	0.0036	0.006723	0.017743	0.004888	0.009508	0.016112	0.011541	0.0195
656	0.001327	0.007477	0.017553	0.003532	0.006736	0.017846	0.004861	0.009542	0.016306	0.011671	0.019872
655	0.001298	0.007393	0.017499	0.003586	0.006601	0.017786	0.004872	0.009359	0.016199	0.011743	0.019988
654	0.001238	0.007428	0.0175	0.003605	0.006783	0.017858	0.004758	0.009487	0.016437	0.011835	0.020184
653	0.001235	0.007394	0.017617	0.00356	0.006638	0.018063	0.004749	0.009499	0.016369	0.011802	0.020435
652	0.001017	0.007237	0.017411	0.003492	0.006587	0.01807	0.004776	0.009589	0.016461	0.012108	0.020597
651	0.000984	0.007268	0.017493	0.003373	0.006529	0.018059	0.004725	0.009411	0.016567	0.012194	0.020945
650	0.000968	0.007303	0.017436	0.003295	0.006523	0.018213	0.004927	0.009449	0.016619	0.012255	0.020927
649	0.000862	0.007154	0.017385	0.003387	0.006488	0.018143	0.00485	0.00954	0.016718	0.012371	0.021129
648	0.000823	0.007149	0.017326	0.003325	0.006484	0.018314	0.004806	0.009615	0.016744	0.012521	0.02138
647	0.000756	0.006952	0.017325	0.003193	0.006381	0.018404	0.004723	0.009606	0.016777	0.01268	0.02157
646	0.000691	0.007027	0.017348	0.00319	0.006458	0.018539	0.004874	0.009816	0.01702	0.012798	0.021695
645	0.0006	0.007	0.017315	0.003105	0.006395	0.01847	0.004769	0.009682	0.017032	0.012896	0.022091
644	0.000506	0.006994	0.017313	0.003097	0.006412	0.018584	0.004714	0.009724	0.017101	0.01304	0.022168
643	0.000419	0.006866	0.017306	0.003086	0.006351	0.018674	0.004799	0.009687	0.017335	0.013093	0.022298
642	0.000279	0.006776	0.017274	0.00296	0.006418	0.018651	0.004705	0.009845	0.017325	0.013371	0.022615
641	0.000317	0.006817	0.017285	0.002923	0.006431	0.018757	0.004913	0.00983	0.017404	0.013458	0.022934
640	0.000202	0.006681	0.017342	0.003014	0.006444	0.018874	0.004737	0.009885	0.017648	0.013604	0.023088
639	0.000279	0.006827	0.017243	0.002928	0.006295	0.018894	0.004761	0.009976	0.017601	0.013715	0.023332
638	0.000233	0.006708	0.01728	0.002852	0.006411	0.018979	0.004723	0.009961	0.017611	0.013839	0.023606
637	3.89E-05	0.006695	0.017319	0.002832	0.006458	0.019148	0.00483	0.010043	0.017885	0.013987	0.023857
636	0.000125	0.006788	0.017333	0.002805	0.006312	0.019116	0.004868	0.01009	0.018081	0.014187	0.024122
635	0	0.006571	0.017357	0.002843	0.006339	0.019211	0.004934	0.010164	0.018122	0.014301	0.024472
634	-7.87E-06	0.006634	0.017384	0.002802	0.006365	0.019494	0.005085	0.010232	0.01819	0.014527	0.024744
633	5.53E-05	0.006609	0.017512	0.002824	0.006396	0.019421	0.004955	0.010375	0.018386	0.014746	0.024944
632	-0.00016	0.00662	0.017401	0.002854	0.006541	0.019666	0.004949	0.01053	0.018494	0.014884	0.025238
631	-0.0001	0.0065	0.017557	0.002997	0.006518	0.01973	0.005064	0.01059	0.018635	0.015124	0.025569
630	-0.0001	0.006755	0.01762	0.002906	0.006547	0.019778	0.005064	0.010681	0.018862	0.015354	0.025852
629	-0.00014	0.006695	0.017556	0.003071	0.006605	0.019963	0.005152	0.010847	0.019071	0.015495	0.026144
628	-0.00018	0.006628	0.017803	0.003002	0.006695	0.020181	0.005267	0.010947	0.019259	0.015838	0.026492
627	-0.00014	0.006831	0.01778	0.002955	0.006805	0.020341	0.005327	0.010951	0.01944	0.016004	0.026879
626	-0.00019	0.006775	0.017778	0.003003	0.006811	0.020453	0.005477	0.011108	0.019564	0.016315	0.027153
625	-8.05E-05	0.006792	0.017922	0.003153	0.006921	0.020747	0.005601	0.011294	0.019767	0.016534	0.027586

EXCLI Journal 2020;19:48-70 – ISSN 1611-2156
Received: October 03, 2019, accepted: December 19, 2019, published: January 03, 2020

Absorption intensity											
**Wavelength (nm)**	**concentration of α-methylbutyrylshikon (M)**										
	A	*B*	*C*	*D*	*E*	*F*	*G*	*I*	*J*	*K*	*L*
	0.00	$8.0x10^{-7}$	$1.6x10^{-6}$	$2.0x10^{-6}$	$2.8x10^{-6}$	$4.0x10^{-6}$	$4.8x10^{-6}$	$6.0x10^{-6}$	$8.0x10^{-6}$	$1.2x10^{-5}$	$1.6x10^{-5}$
624	-8.05E-05	0.006827	0.017894	0.003162	0.006988	0.020795	0.005615	0.011403	0.020002	0.016819	0.027856
623	-0.00011	0.006955	0.018	0.003187	0.007073	0.020881	0.005771	0.011576	0.020175	0.01696	0.028184
622	-4.01E-05	0.006947	0.018076	0.003342	0.007143	0.021137	0.005844	0.011747	0.020328	0.017345	0.028498
621	7.98E-05	0.006932	0.01821	0.003319	0.007229	0.021197	0.005984	0.011865	0.020595	0.017462	0.028892
620	-6.36E-05	0.007139	0.018242	0.003458	0.00726	0.02156	0.00618	0.011988	0.02075	0.017862	0.029322
619	4.74E-05	0.007166	0.018365	0.003657	0.007455	0.021546	0.006056	0.012176	0.021032	0.017946	0.029674
618	-7.87E-06	0.007211	0.018308	0.003588	0.007558	0.021756	0.006392	0.012345	0.021274	0.018444	0.029976
617	5.44E-05	0.007333	0.018595	0.003649	0.007567	0.021974	0.006387	0.012399	0.021244	0.018459	0.03034
616	0.000108	0.007236	0.018543	0.003705	0.007781	0.022111	0.006563	0.012642	0.021619	0.018861	0.030754
615	0.00029	0.007515	0.018722	0.003795	0.007842	0.022271	0.006776	0.012798	0.021748	0.019168	0.030982
614	0.000325	0.00751	0.018786	0.003883	0.008019	0.022264	0.006801	0.013013	0.022133	0.019383	0.031454
613	0.000186	0.007512	0.018716	0.004051	0.008097	0.022624	0.006933	0.013104	0.022165	0.019698	0.031719
612	0.000332	0.007619	0.018943	0.0041	0.008176	0.022703	0.006999	0.013186	0.022519	0.019879	0.032255
611	0.000247	0.007603	0.019004	0.004113	0.008118	0.022856	0.007072	0.013403	0.02255	0.020104	0.032503
610	0.000215	0.007669	0.018975	0.00414	0.008227	0.022924	0.007217	0.013613	0.022747	0.020324	0.032815
609	0.00024	0.007561	0.01913	0.004177	0.00835	0.023164	0.007217	0.013693	0.022923	0.020561	0.033177
608	0.000216	0.007588	0.019062	0.004152	0.008337	0.023359	0.007315	0.013843	0.023197	0.02088	0.033487
607	0.000227	0.007657	0.019058	0.004035	0.008404	0.023444	0.00733	0.013881	0.023191	0.020951	0.033895
606	0.000237	0.007651	0.019218	0.004095	0.008574	0.02351	0.007288	0.013923	0.02334	0.021214	0.034039
605	8.45E-05	0.007699	0.019156	0.004109	0.008392	0.023659	0.007262	0.014097	0.023418	0.021372	0.034416
604	0.000127	0.007535	0.019197	0.004004	0.008493	0.023694	0.007412	0.014129	0.023666	0.021664	0.034709
603	-0.00013	0.007429	0.019181	0.004024	0.008387	0.023705	0.007477	0.014187	0.023731	0.021851	0.034881
602	-4.47E-05	0.007379	0.019067	0.003931	0.008355	0.023808	0.007415	0.014054	0.023808	0.02186	0.035074
601	-0.00031	0.007334	0.018969	0.003798	0.008367	0.023805	0.007274	0.01402	0.023736	0.022131	0.035272
600	-0.00039	0.00712	0.01882	0.003815	0.008186	0.023822	0.007282	0.014188	0.023856	0.022092	0.03558
599	-0.00054	0.007094	0.018775	0.003638	0.008212	0.023877	0.007185	0.014155	0.023801	0.022283	0.035749
598	-0.00073	0.006929	0.018595	0.003629	0.007918	0.023911	0.007111	0.014236	0.023872	0.022242	0.036131
597	-0.00104	0.006741	0.018714	0.003521	0.008037	0.023979	0.006873	0.014066	0.023965	0.022455	0.036139
596	-0.00116	0.00654	0.01837	0.003342	0.007781	0.023829	0.006838	0.013955	0.024036	0.022369	0.03635
595	-0.00131	0.006475	0.018366	0.003177	0.007642	0.02381	0.006918	0.01399	0.02395	0.022584	0.036575
594	-0.00147	0.00627	0.018344	0.00305	0.007895	0.02388	0.006764	0.013869	0.02393	0.022715	0.036623
593	-0.0016	0.0063	0.018146	0.002895	0.007547	0.023823	0.006789	0.013869	0.0241	0.022808	0.036996
592	-0.00173	0.006288	0.018186	0.002979	0.007486	0.023815	0.006759	0.014061	0.023896	0.022974	0.036869
591	-0.002	0.006104	0.01817	0.002795	0.007552	0.023943	0.0067	0.014071	0.024122	0.023021	0.037031
590	-0.0019	0.005923	0.018017	0.002777	0.007406	0.023711	0.006785	0.014057	0.023885	0.023015	0.037322
589	-0.00185	0.006083	0.017769	0.002775	0.007552	0.023681	0.00661	0.013905	0.024056	0.023114	0.037675
588	-0.00202	0.005792	0.017836	0.002708	0.007432	0.023863	0.006446	0.01381	0.024214	0.02323	0.037935

EXCLI Journal 2020;19:48-70 – ISSN 1611-2156
Received: October 03, 2019, accepted: December 19, 2019, published: January 03, 2020

**Absorption intensity**											
**Wavelength (nm)**	**concentration of α-methylbutyrylshikon (M)**										
	A	*B*	*C*	*D*	*E*	*F*	*G*	*I*	*J*	*K*	*L*
	0.00	$8.0x10^{-7}$	$1.6x10^{-6}$	$2.0x10^{-6}$	$2.8x10^{-6}$	$4.0x10^{-6}$	$4.8x10^{-6}$	$6.0x10^{-6}$	$8.0x10^{-6}$	$1.2x10^{-5}$	$1.6x10^{-5}$
587	-0.00188	0.006013	0.018054	0.00265	0.007511	0.024205	0.006905	0.014316	0.024765	0.023835	0.038561
586	-0.00197	0.006181	0.018226	0.002774	0.007607	0.024444	0.006984	0.014547	0.024834	0.024099	0.038963
585	-0.00204	0.005974	0.018272	0.003001	0.007722	0.024761	0.006992	0.014572	0.025039	0.024431	0.039321
584	-0.00193	0.005998	0.018425	0.002936	0.007798	0.024821	0.007076	0.014786	0.025306	0.024699	0.039539
583	-0.00208	0.006253	0.018548	0.003068	0.007817	0.024943	0.007203	0.014872	0.025429	0.024852	0.040146
582	-0.0021	0.006151	0.018329	0.003029	0.008044	0.025156	0.007256	0.015082	0.02565	0.025092	0.040346
581	-0.00207	0.006243	0.018721	0.003147	0.008023	0.02546	0.007488	0.015343	0.025966	0.025391	0.040786
580	-0.00193	0.006446	0.018674	0.003295	0.008101	0.025504	0.007525	0.01552	0.026138	0.025711	0.041145
579	-0.00195	0.006424	0.018937	0.003354	0.008306	0.025855	0.007674	0.015504	0.026353	0.02613	0.041567
578	-0.00189	0.006573	0.019085	0.003441	0.008371	0.025942	0.007861	0.015755	0.026768	0.026355	0.041881
577	-0.00186	0.006618	0.019095	0.003472	0.00849	0.026205	0.007881	0.015794	0.026805	0.026646	0.042308
576	-0.00171	0.00668	0.019107	0.003671	0.008558	0.026501	0.008187	0.016266	0.026994	0.026776	0.042703
575	-0.00193	0.006776	0.019368	0.003722	0.008763	0.026722	0.008132	0.01634	0.027145	0.027037	0.042927
574	-0.00166	0.006922	0.019329	0.0037	0.008758	0.026773	0.008222	0.016305	0.027485	0.027277	0.043379
573	-0.00182	0.006773	0.019395	0.003634	0.009036	0.027031	0.008362	0.016528	0.027503	0.027541	0.043658
572	-0.00176	0.006852	0.019496	0.00373	0.00908	0.027125	0.008471	0.016701	0.0278	0.027891	0.044028
571	-0.00149	0.006926	0.019623	0.003829	0.009076	0.027298	0.008494	0.016922	0.027998	0.02806	0.044237
570	-0.0016	0.006947	0.019592	0.003985	0.009198	0.027483	0.00869	0.016996	0.028073	0.028311	0.044576
569	-0.00179	0.006907	0.01977	0.003812	0.009311	0.027692	0.008826	0.017109	0.028264	0.028429	0.044919
568	-0.00148	0.007148	0.019897	0.003989	0.009253	0.027908	0.008787	0.017378	0.028588	0.028692	0.045021
567	-0.00124	0.00774	0.020351	0.004426	0.009905	0.028501	0.009182	0.017867	0.029151	0.029374	0.045872
566	-0.00106	0.00763	0.020502	0.004498	0.010016	0.02863	0.009269	0.017999	0.029269	0.029557	0.046085
565	-0.00096	0.007681	0.020409	0.004472	0.009965	0.028772	0.009337	0.01799	0.029399	0.029776	0.046532
564	-0.00115	0.007779	0.020539	0.004543	0.010058	0.028801	0.009482	0.018218	0.029608	0.029784	0.04662
563	-0.00101	0.007679	0.020822	0.004652	0.01023	0.029118	0.00949	0.018379	0.029696	0.03006	0.046841
562	-0.00104	0.007824	0.020805	0.004627	0.010359	0.029072	0.009698	0.018468	0.029907	0.030434	0.047108
561	-0.00099	0.007875	0.020845	0.004744	0.010264	0.029378	0.009685	0.018584	0.030058	0.030402	0.047451
560	-0.00098	0.007891	0.020872	0.004853	0.010339	0.029568	0.009703	0.018585	0.03014	0.030515	0.047515
559	-0.00105	0.007855	0.020724	0.004666	0.01031	0.029426	0.00983	0.018639	0.030445	0.030724	0.047672
558	-0.00125	0.008022	0.0209	0.004832	0.010343	0.029742	0.009811	0.01884	0.030291	0.03082	0.04795
557	-0.00114	0.007792	0.021062	0.004749	0.010444	0.029772	0.009823	0.018785	0.030397	0.031038	0.048206
556	-0.00118	0.007971	0.020918	0.004899	0.010481	0.029895	0.00997	0.018895	0.03058	0.031158	0.048301
555	-0.00116	0.008042	0.020997	0.004784	0.010408	0.029973	0.009901	0.018857	0.030648	0.031285	0.048695
554	-0.00131	0.007839	0.020986	0.004799	0.010523	0.030096	0.009936	0.018872	0.030716	0.031522	0.048771
553	-0.00124	0.007824	0.020956	0.004656	0.010427	0.030086	0.009978	0.019089	0.030963	0.031683	0.048896
552	-0.00129	0.007859	0.020928	0.004681	0.010373	0.030231	0.010011	0.019108	0.030912	0.031652	0.049197
551	-0.00132	0.007915	0.021053	0.004664	0.010616	0.030346	0.009891	0.019313	0.031027	0.031796	0.049386

EXCLI Journal 2020;19:48-70 – ISSN 1611-2156
Received: October 03, 2019, accepted: December 19, 2019, published: January 03, 2020

Absorption intensity											
**Wavelength (nm)**	**concentration of α-methylbutyrylshikon (M)**										
	A	*B*	*C*	*D*	*E*	*F*	*G*	*I*	*J*	*K*	*L*
	0.00	$8.0x10^{-7}$	$1.6x10^{-6}$	$2.0x10^{-6}$	$2.8x10^{-6}$	$4.0x10^{-6}$	$4.8x10^{-6}$	$6.0x10^{-6}$	$8.0x10^{-6}$	$1.2x10^{-5}$	$1.6x10^{-5}$
550	-0.00145	0.007889	0.020925	0.004619	0.010424	0.030411	0.010058	0.019194	0.031063	0.031888	0.04944
549	-0.00147	0.00787	0.020964	0.004583	0.010481	0.030479	0.009959	0.019338	0.03129	0.032163	0.049678
548	-0.00155	0.007885	0.020969	0.004548	0.010567	0.030589	0.010013	0.019211	0.031246	0.032087	0.049838
547	-0.00159	0.007774	0.021031	0.004716	0.010568	0.03063	0.009972	0.019313	0.031381	0.032149	0.049865
546	-0.00164	0.007672	0.021023	0.004594	0.010493	0.030767	0.009958	0.01948	0.031289	0.032445	0.050119
545	-0.00188	0.007615	0.020904	0.004491	0.010463	0.030675	0.009938	0.019368	0.031415	0.032412	0.050216
544	-0.0019	0.007507	0.020975	0.004528	0.010494	0.030981	0.009873	0.019319	0.031457	0.032561	0.050486
543	-0.00181	0.00769	0.020906	0.004419	0.010381	0.030886	0.009924	0.019434	0.03159	0.032636	0.050469
542	-0.00197	0.007491	0.02093	0.004372	0.010446	0.030889	0.009862	0.019441	0.031418	0.032667	0.050801
541	-0.00204	0.007342	0.02086	0.004294	0.010313	0.030894	0.009908	0.019335	0.03165	0.032731	0.050742
540	-0.00214	0.007507	0.02101	0.004305	0.010324	0.030999	0.009804	0.019399	0.03168	0.03282	0.051044
539	-0.00231	0.007342	0.020889	0.004157	0.010272	0.030986	0.009709	0.019406	0.031787	0.032878	0.051077
538	-0.0023	0.007351	0.020804	0.00414	0.010208	0.031077	0.009748	0.019388	0.031789	0.032856	0.051271
537	-0.00243	0.0072	0.020716	0.004016	0.010086	0.031035	0.009613	0.019342	0.031827	0.03303	0.051297
536	-0.00251	0.007126	0.020739	0.004048	0.010148	0.03113	0.0097	0.019421	0.032045	0.03315	0.051405
535	-0.00264	0.007062	0.02076	0.00397	0.010107	0.03119	0.009641	0.019392	0.031823	0.033049	0.051456
534	-0.00249	0.007092	0.020629	0.003906	0.010024	0.031319	0.009671	0.019438	0.03192	0.033163	0.051605
533	-0.00269	0.006967	0.020619	0.003783	0.009974	0.031271	0.009415	0.019392	0.031956	0.03314	0.051804
532	-0.00294	0.007045	0.020683	0.003643	0.010002	0.031238	0.009512	0.019367	0.031901	0.0332	0.051901
531	-0.00286	0.006977	0.020586	0.003673	0.010018	0.031429	0.009436	0.019405	0.032062	0.033324	0.05194
530	-0.00296	0.006888	0.020503	0.003671	0.010044	0.031416	0.00936	0.019432	0.032114	0.033212	0.052094
529	-0.00309	0.006882	0.020451	0.003675	0.010114	0.031283	0.009427	0.019349	0.032064	0.03333	0.052149
528	-0.00307	0.006933	0.020556	0.003685	0.009906	0.03153	0.009292	0.019371	0.031991	0.033519	0.052206
527	-0.00316	0.006853	0.020416	0.003615	0.009945	0.031563	0.009355	0.019534	0.032183	0.033385	0.052486
526	-0.00326	0.006887	0.020723	0.003517	0.00996	0.031584	0.009254	0.019553	0.03216	0.033461	0.052459
525	-0.00324	0.006852	0.020477	0.003454	0.009808	0.03173	0.009344	0.019559	0.032297	0.033603	0.052395
524	-0.00315	0.006939	0.020592	0.003445	0.010022	0.031642	0.009389	0.019549	0.032435	0.03356	0.05273
523	-0.00331	0.00688	0.020613	0.003444	0.009958	0.031767	0.009411	0.01957	0.032371	0.033748	0.052756
522	-0.00324	0.006814	0.020646	0.003531	0.009961	0.031857	0.009225	0.019521	0.032465	0.033689	0.052807
521	-0.0033	0.007003	0.020604	0.003391	0.009921	0.031783	0.009299	0.019737	0.032568	0.033839	0.052916
520	-0.00328	0.006851	0.020681	0.003574	0.009952	0.031986	0.009389	0.019518	0.032604	0.033774	0.053084
519	-0.00333	0.006989	0.020835	0.003594	0.009988	0.031997	0.009379	0.019701	0.03271	0.033942	0.053058
518	-0.00339	0.007053	0.020669	0.003463	0.010131	0.032009	0.009465	0.019885	0.032737	0.033863	0.053146
517	-0.00343	0.006984	0.020816	0.003536	0.010073	0.032086	0.009345	0.019808	0.032642	0.033832	0.053277
516	-0.00346	0.006947	0.020845	0.003476	0.01009	0.032261	0.009508	0.019847	0.032805	0.033931	0.053307
515	-0.00348	0.007038	0.020839	0.003412	0.010061	0.032144	0.009437	0.019892	0.032801	0.034038	0.053402
514	-0.00336	0.006982	0.020954	0.00343	0.010128	0.032269	0.009495	0.020068	0.032984	0.034063	0.053469

EXCLI Journal 2020;19:48-70 – ISSN 1611-2156
Received: October 03, 2019, accepted: December 19, 2019, published: January 03, 2020

Absorption intensity											
**Wavelength (nm)**	**concentration of α-methylbutyrylshikon (M)**										
	A	*B*	*C*	*D*	*E*	*F*	*G*	*I*	*J*	*K*	*L*
	0.00	$8.0x10^{-7}$	$1.6x10^{-6}$	$2.0x10^{-6}$	$2.8x10^{-6}$	$4.0x10^{-6}$	$4.8x10^{-6}$	$6.0x10^{-6}$	$8.0x10^{-6}$	$1.2x10^{-5}$	$1.6x10^{-5}$
513	-0.00343	0.007055	0.021019	0.003441	0.010239	0.032381	0.009353	0.019855	0.0329	0.034081	0.053449
512	-0.00336	0.007251	0.02089	0.003446	0.010252	0.032398	0.009422	0.020124	0.032953	0.034204	0.05353
511	-0.00351	0.007096	0.021063	0.003554	0.010228	0.032393	0.009608	0.02002	0.033006	0.034304	0.053674
510	-0.00345	0.007303	0.021061	0.003514	0.010322	0.032415	0.009525	0.020048	0.033162	0.034322	0.053673
509	-0.00333	0.007366	0.02119	0.003559	0.010223	0.032526	0.009541	0.02019	0.033116	0.034371	0.053829
508	-0.00341	0.007277	0.021098	0.003609	0.010456	0.032622	0.009535	0.020283	0.033171	0.034285	0.053856
507	-0.00335	0.007321	0.021219	0.003546	0.010487	0.032606	0.00951	0.020077	0.033315	0.03445	0.053981
506	-0.00341	0.007424	0.021236	0.003551	0.010324	0.032657	0.009615	0.020326	0.033302	0.034519	0.054074
505	-0.00346	0.00735	0.021369	0.003559	0.010469	0.032776	0.009453	0.020289	0.033322	0.034582	0.054177
504	-0.00348	0.007471	0.021282	0.003576	0.010544	0.032959	0.00955	0.020379	0.033384	0.034578	0.054147
503	-0.00332	0.007374	0.021449	0.003531	0.010434	0.032807	0.009618	0.020288	0.033461	0.03453	0.054221
502	-0.00339	0.007579	0.021424	0.003676	0.010507	0.032799	0.009693	0.020554	0.033585	0.03453	0.05428
501	-0.0033	0.007505	0.021352	0.003601	0.010619	0.032905	0.009699	0.020479	0.033568	0.034524	0.05421
500	-0.00322	0.007606	0.021668	0.003628	0.010517	0.03291	0.009678	0.02042	0.033689	0.034711	0.054363
499	-0.00341	0.007661	0.021577	0.003593	0.010608	0.03293	0.009733	0.02053	0.033669	0.034551	0.054176
498	-0.00341	0.007674	0.021625	0.003558	0.010709	0.033064	0.009631	0.020609	0.033654	0.034616	0.054293
497	-0.00344	0.007692	0.021617	0.003539	0.010674	0.033014	0.009731	0.02053	0.033436	0.034673	0.054351
496	-0.00332	0.007638	0.021656	0.003586	0.010839	0.033046	0.009669	0.02063	0.033708	0.0347	0.054397
495	-0.0033	0.007732	0.021646	0.00369	0.010844	0.033038	0.009459	0.020522	0.03368	0.03454	0.054441
494	-0.00336	0.007786	0.021653	0.003496	0.010668	0.033137	0.009642	0.020571	0.033747	0.034561	0.054363
493	-0.00332	0.007761	0.021665	0.003589	0.010676	0.033004	0.00958	0.020604	0.033855	0.034715	0.054558
492	-0.00331	0.007731	0.021861	0.003587	0.010741	0.033194	0.009524	0.020532	0.033705	0.034672	0.054525
491	-0.00329	0.007854	0.021878	0.003469	0.010693	0.033107	0.009576	0.020519	0.033796	0.03447	0.054328
490	-0.00338	0.007964	0.021767	0.003516	0.01066	0.033148	0.009617	0.020615	0.033802	0.034601	0.054315
489	-0.0034	0.007864	0.021974	0.003458	0.01079	0.03323	0.009447	0.020586	0.03377	0.034412	0.054304
488	-0.00348	0.007984	0.021977	0.003586	0.010766	0.03325	0.009629	0.020659	0.033903	0.034498	0.054305
487	-0.00348	0.007839	0.021907	0.003562	0.01063	0.033096	0.00935	0.02053	0.033745	0.034443	0.054307
486	-0.00342	0.007881	0.021845	0.003493	0.010792	0.033228	0.009399	0.020484	0.033811	0.034329	0.054204
485	-0.00338	0.007814	0.021939	0.003379	0.010616	0.033116	0.009482	0.020332	0.033794	0.034201	0.054196
484	-0.00362	0.007818	0.021834	0.003381	0.010694	0.032942	0.009217	0.020593	0.033731	0.034241	0.053976
483	-0.00361	0.007894	0.021983	0.003273	0.010524	0.03316	0.009291	0.020225	0.033527	0.034143	0.053785
482	-0.00364	0.007821	0.021828	0.003312	0.010551	0.032968	0.009071	0.020154	0.033636	0.033951	0.053695
481	-0.00384	0.007756	0.021831	0.003082	0.010355	0.032882	0.009033	0.020226	0.03345	0.033892	0.053573
480	-0.00393	0.00769	0.021609	0.003071	0.010417	0.032757	0.008816	0.020113	0.033445	0.033614	0.053498
479	-0.00409	0.007629	0.021627	0.002931	0.01046	0.032651	0.008806	0.020069	0.033134	0.033377	0.053102
478	-0.00396	0.007512	0.0216	0.002839	0.010128	0.032496	0.008626	0.019797	0.033075	0.03326	0.053012
477	-0.00412	0.00748	0.021422	0.002642	0.01008	0.032545	0.008444	0.019666	0.032956	0.032986	0.0528

EXCLI Journal 2020;19:48-70 – ISSN 1611-2156
Received: October 03, 2019, accepted: December 19, 2019, published: January 03, 2020

Absorption intensity											
**Wavelength (nm)**	**concentration of α-methylbutyrylshikon (M)**										
	A	*B*	*C*	*D*	*E*	*F*	*G*	*I*	*J*	*K*	*L*
	0.00	$8.0x10^{-7}$	$1.6x10^{-6}$	$2.0x10^{-6}$	$2.8x10^{-6}$	$4.0x10^{-6}$	$4.8x10^{-6}$	$6.0x10^{-6}$	$8.0x10^{-6}$	$1.2x10^{-5}$	$1.6x10^{-5}$
476	-0.0042	0.007277	0.021382	0.002567	0.009939	0.032367	0.008422	0.019677	0.032948	0.03286	0.052634
475	-0.00452	0.007395	0.021345	0.002375	0.009833	0.032129	0.008074	0.019546	0.032737	0.032758	0.052376
474	-0.00449	0.007244	0.021185	0.002263	0.009819	0.032063	0.008038	0.01934	0.032611	0.032352	0.052202
473	-0.00456	0.007212	0.020994	0.00212	0.009658	0.03189	0.007837	0.019215	0.032558	0.032213	0.051969
472	-0.00472	0.007063	0.021192	0.001959	0.009581	0.031869	0.007812	0.019181	0.032344	0.032098	0.051873
471	-0.00477	0.006995	0.021007	0.001969	0.009561	0.031681	0.007648	0.018975	0.03229	0.031944	0.051773
470	-0.00494	0.006968	0.020992	0.00184	0.009421	0.031752	0.007514	0.01889	0.032327	0.031672	0.051429
469	-0.00487	0.007057	0.020952	0.001849	0.009271	0.031546	0.007448	0.018831	0.032172	0.03152	0.05138
468	-0.00494	0.007042	0.020933	0.001607	0.009271	0.0316	0.007332	0.018776	0.031964	0.031347	0.051298
467	-0.00499	0.007059	0.021045	0.00173	0.009322	0.031648	0.007268	0.018771	0.03206	0.03127	0.050677
466	-0.00497	0.00713	0.021208	0.001664	0.009285	0.031523	0.007445	0.018692	0.031926	0.031175	0.050621
465	-0.00501	0.00703	0.02099	0.001613	0.009346	0.031511	0.007234	0.018645	0.031997	0.031135	0.050433
464	-0.00495	0.007201	0.02111	0.001701	0.009298	0.031438	0.007138	0.018706	0.03176	0.031049	0.050414
463	-0.00498	0.007192	0.021006	0.001485	0.009327	0.03107	0.007243	0.018729	0.031578	0.03066	0.050413
462	-0.00509	0.007173	0.020752	0.001581	0.009374	0.031187	0.007088	0.018741	0.031474	0.030557	0.050288
461	-0.00498	0.007353	0.020843	0.001619	0.009407	0.031243	0.00704	0.018259	0.031472	0.030411	0.050194
460	-0.00484	0.007278	0.02086	0.001686	0.00943	0.031175	0.006967	0.018335	0.031706	0.030352	0.050106
459	-0.00495	0.007113	0.020895	0.001455	0.009076	0.031276	0.006868	0.018167	0.031506	0.030173	0.050052
458	-0.00491	0.007173	0.02093	0.001519	0.009247	0.031157	0.006779	0.018402	0.031488	0.030256	0.050129
457	-0.00474	0.007357	0.021271	0.001534	0.009409	0.031378	0.006918	0.018419	0.031893	0.030429	0.050103
456	-0.00474	0.007619	0.02125	0.001568	0.009377	0.031608	0.00708	0.018639	0.032044	0.030302	0.050184
455	-0.00493	0.007647	0.021391	0.001706	0.009582	0.03154	0.006923	0.018447	0.031845	0.03044	0.050157
454	-0.00483	0.007685	0.021567	0.001552	0.009467	0.031662	0.006926	0.018582	0.031984	0.03024	0.050042
453	-0.00491	0.007637	0.021495	0.001587	0.009509	0.031556	0.006998	0.018622	0.031845	0.030095	0.049993
452	-0.00463	0.007816	0.021633	0.001534	0.009465	0.031398	0.006875	0.018587	0.031983	0.030069	0.049824
451	-0.00476	0.007815	0.02163	0.001505	0.009458	0.031509	0.006836	0.018592	0.032024	0.029871	0.04973
450	-0.00478	0.007958	0.021549	0.001408	0.00962	0.031595	0.006876	0.018525	0.031934	0.029849	0.049814
449	-0.00487	0.007795	0.021781	0.001528	0.009596	0.031559	0.006715	0.018574	0.031715	0.029683	0.049432
448	-0.0049	0.007861	0.021636	0.001507	0.009548	0.031514	0.006832	0.018552	0.031808	0.029683	0.049444
447	-0.00491	0.008067	0.021599	0.001353	0.009463	0.031495	0.006606	0.018493	0.031818	0.029546	0.049357
446	-0.00495	0.008041	0.021654	0.001297	0.009401	0.031543	0.006643	0.018519	0.031691	0.029521	0.049184
445	-0.00499	0.007984	0.021721	0.001442	0.009612	0.03158	0.006702	0.01859	0.031801	0.029537	0.049155
444	-0.0049	0.007957	0.0218	0.001215	0.009589	0.031531	0.006606	0.018435	0.031764	0.029272	0.049168
443	-0.00496	0.008084	0.021709	0.001317	0.009539	0.031599	0.006536	0.018396	0.03174	0.029125	0.049181
442	-0.00496	0.008017	0.021824	0.001247	0.009684	0.031496	0.00645	0.018394	0.031844	0.029092	0.048905
441	-0.00502	0.007959	0.021784	0.001258	0.009554	0.031695	0.006402	0.018349	0.031738	0.029077	0.049037
440	-0.00487	0.008196	0.021886	0.001302	0.009625	0.031544	0.006354	0.01839	0.031726	0.029073	0.048834

Absorption intensity											
**Wavelength (nm)**	**concentration of α-methylbutyrylshikon (M)**										
	A	*B*	*C*	*D*	*E*	*F*	*G*	*I*	*J*	*K*	*L*
	0.00	$8.0x10^{-7}$	$1.6x10^{-6}$	$2.0x10^{-6}$	$2.8x10^{-6}$	$4.0x10^{-6}$	$4.8x10^{-6}$	$6.0x10^{-6}$	$8.0x10^{-6}$	$1.2x10^{-5}$	$1.6x10^{-5}$
439	-0.0049	0.008228	0.022059	0.001312	0.009698	0.031661	0.006508	0.018446	0.031884	0.028993	0.048866
438	-0.00468	0.008502	0.022179	0.001376	0.009836	0.031875	0.00652	0.018565	0.032105	0.029143	0.049121
437	-0.00472	0.008395	0.022228	0.001442	0.00975	0.031858	0.00661	0.018571	0.031832	0.029043	0.049085
436	-0.00494	0.008457	0.022172	0.001211	0.009881	0.031785	0.006323	0.018408	0.031928	0.028685	0.048734
435	-0.00494	0.008209	0.022108	0.001078	0.009547	0.031515	0.006115	0.018354	0.031728	0.028694	0.048585
434	-0.0051	0.008349	0.021833	0.001075	0.009615	0.031358	0.006221	0.018428	0.031719	0.028598	0.048617
433	-0.0051	0.008245	0.022035	0.000926	0.009458	0.031574	0.006006	0.018356	0.031647	0.028347	0.048475
432	-0.00536	0.008235	0.021966	0.000911	0.009567	0.031694	0.006155	0.018219	0.031653	0.028374	0.04855
431	-0.00521	0.008341	0.022183	0.000796	0.009568	0.031649	0.005774	0.018226	0.03163	0.028134	0.048446
430	-0.00528	0.008272	0.022237	0.000882	0.009474	0.031766	0.005905	0.018336	0.031607	0.028161	0.048233
429	-0.00507	0.008337	0.022071	0.000622	0.009485	0.03129	0.00589	0.018191	0.031625	0.028088	0.048411
428	-0.00525	0.008404	0.022059	0.000643	0.009458	0.031639	0.005806	0.018232	0.03158	0.028178	0.048272
427	-0.00552	0.008297	0.022141	0.000718	0.009393	0.031675	0.005584	0.018119	0.031473	0.027908	0.0481
426	-0.00536	0.008354	0.022199	0.000728	0.009413	0.031583	0.005593	0.018137	0.031743	0.027978	0.048128
425	-0.00547	0.008403	0.022021	0.000411	0.00931	0.031558	0.005582	0.018023	0.031742	0.027864	0.04818
424	-0.0055	0.008459	0.021999	0.000612	0.009367	0.031558	0.005501	0.018053	0.031538	0.027758	0.048114
423	-0.0055	0.008174	0.022142	0.00034	0.009299	0.03154	0.00551	0.018061	0.031457	0.027604	0.047865
422	-0.00562	0.008443	0.021989	0.000417	0.009252	0.031401	0.005368	0.017952	0.031445	0.027766	0.04793
421	-0.00579	0.008292	0.02183	0.000498	0.009275	0.031344	0.005232	0.018003	0.031442	0.027511	0.047943
420	-0.0057	0.008368	0.022037	0.000283	0.009185	0.031551	0.005383	0.01789	0.031491	0.027758	0.047989
419	-0.0059	0.008241	0.021993	0.000289	0.009081	0.031621	0.005185	0.017948	0.031577	0.027459	0.048137
418	-0.00574	0.008384	0.022163	0.000341	0.009479	0.031738	0.005346	0.01806	0.031636	0.027505	0.048075
417	-0.00571	0.008374	0.021869	0.00034	0.009012	0.031499	0.00484	0.017792	0.031298	0.027113	0.047676
416	-0.00597	0.008204	0.02214	1.47E-05	0.00911	0.03141	0.0049	0.017672	0.031331	0.027239	0.047627
415	-0.0062	0.008208	0.021979	-0.00014	0.009314	0.031472	0.004787	0.017751	0.031341	0.027092	0.047807
414	-0.00607	0.008194	0.022056	-2.30E-05	0.009175	0.031521	0.004978	0.017702	0.031728	0.027128	0.047722
413	-0.00619	0.008465	0.022146	-4.71E-05	0.009181	0.031535	0.004812	0.017813	0.031293	0.027135	0.047727
412	-0.0061	0.008407	0.022127	-0.00016	0.009374	0.031657	0.004849	0.017777	0.031515	0.026871	0.047794
411	-0.00631	0.008403	0.022115	-0.00039	0.009142	0.031679	0.005005	0.017722	0.031563	0.026963	0.047923
410	-0.00613	0.008613	0.022486	-3.35E-05	0.009309	0.031789	0.00473	0.018002	0.031613	0.027077	0.047898
409	-0.00616	0.008392	0.022167	7.71E-05	0.009201	0.031929	0.004542	0.017907	0.031544	0.026925	0.047746
408	-0.00603	0.008617	0.022488	2.62E-05	0.009494	0.031896	0.004841	0.017887	0.031712	0.02678	0.047918
407	-0.00591	0.008951	0.02232	-0.00024	0.009304	0.031793	0.004675	0.018086	0.031831	0.026632	0.047741
406	-0.00605	0.008818	0.022499	-0.00017	0.009409	0.031891	0.004764	0.018129	0.031835	0.026998	0.047764
405	-0.00613	0.009151	0.022566	-0.00021	0.009529	0.032096	0.004816	0.018221	0.031885	0.026823	0.047598
404	-0.0062	0.009032	0.022555	-0.00025	0.009491	0.031806	0.004496	0.017939	0.03147	0.026685	0.047637
403	-0.0064	0.008889	0.022262	-0.00047	0.009545	0.031713	0.004474	0.017722	0.031364	0.026168	0.047701

EXCLI Journal 2020;19:48-70 – ISSN 1611-2156
Received: October 03, 2019, accepted: December 19, 2019, published: January 03, 2020

Absorption intensity											
**Wavelength (nm)**	**concentration of α-methylbutyrylshikon (M)**										
	A	*B*	*C*	*D*	*E*	*F*	*G*	*I*	*J*	*K*	*L*
	0.00	$8.0x10^{-7}$	$1.6x10^{-6}$	$2.0x10^{-6}$	$2.8x10^{-6}$	$4.0x10^{-6}$	$4.8x10^{-6}$	$6.0x10^{-6}$	$8.0x10^{-6}$	$1.2x10^{-5}$	$1.6x10^{-5}$
402	-0.00618	0.008432	0.02214	-0.00033	0.009014	0.031642	0.004213	0.017683	0.031619	0.026418	0.047726
401	-0.00612	0.008955	0.022627	-0.00037	0.009282	0.032076	0.004453	0.018044	0.032	0.026616	0.047923
400	-0.00621	0.009104	0.022712	-0.00041	0.009605	0.032016	0.004583	0.018102	0.032087	0.026509	0.048067
399	-0.00629	0.009117	0.022759	-0.00034	0.009492	0.032129	0.004465	0.018163	0.032055	0.02648	0.048137
398	-0.00634	0.008996	0.022727	-0.00055	0.009272	0.032161	0.004389	0.018168	0.031998	0.026714	0.048171
397	-0.0066	0.008895	0.022822	-0.00071	0.009508	0.03216	0.00433	0.018091	0.032029	0.026562	0.04828
396	-0.00621	0.009011	0.022771	-0.00049	0.009541	0.032167	0.004394	0.017963	0.032067	0.026455	0.048373
395	-0.00653	0.009125	0.022773	-0.00068	0.009329	0.032279	0.004346	0.017871	0.032	0.02646	0.048107
394	-0.00638	0.009095	0.022866	-0.00074	0.009375	0.032551	0.004246	0.018321	0.032016	0.026453	0.048356
393	-0.0067	0.009082	0.022834	-0.00077	0.009304	0.03251	0.004109	0.018038	0.032204	0.0264	0.048369
392	-0.00662	0.009156	0.022896	-0.00081	0.009352	0.032474	0.004234	0.018066	0.032398	0.026294	0.048462
391	-0.0068	0.009216	0.022823	-0.00098	0.009388	0.032218	0.00403	0.018152	0.032102	0.026406	0.04851
390	-0.00706	0.009026	0.02293	-0.00108	0.009225	0.032381	0.004173	0.017842	0.032165	0.026241	0.048427
389	-0.00689	0.009051	0.022766	-0.00107	0.009231	0.032575	0.004084	0.017966	0.032283	0.026308	0.048592
388	-0.00657	0.009463	0.023027	-0.00071	0.009727	0.032991	0.004308	0.018609	0.032975	0.026653	0.049124
387	-0.00712	0.008893	0.022604	-0.00144	0.009366	0.032344	0.003511	0.017919	0.032226	0.026028	0.048653
386	-0.00736	0.00884	0.022891	-0.00144	0.009139	0.032382	0.003548	0.017666	0.032439	0.02597	0.048717
385	-0.00758	0.008798	0.022653	-0.00154	0.008998	0.032452	0.003524	0.017985	0.032287	0.026009	0.048596
384	-0.0076	0.008628	0.022615	-0.00162	0.009182	0.032488	0.003435	0.017922	0.032232	0.025893	0.04887
383	-0.00749	0.00893	0.022448	-0.00185	0.009059	0.032386	0.003313	0.01782	0.032321	0.025935	0.04835
382	-0.00764	0.008504	0.022206	-0.00209	0.008944	0.03226	0.00318	0.017672	0.032107	0.02598	0.048831
381	-0.0079	0.008901	0.022722	-0.00174	0.009042	0.032457	0.003077	0.017897	0.032466	0.026006	0.049169
380	-0.00777	0.008836	0.02256	-0.00207	0.009028	0.032408	0.003298	0.017835	0.032431	0.02609	0.049103
379	-0.008	0.008608	0.022654	-0.00222	0.008708	0.032444	0.003235	0.017714	0.032664	0.026157	0.04931
378	-0.00829	0.008939	0.022486	-0.00239	0.008586	0.032373	0.002894	0.017456	0.032581	0.025888	0.049524
377	-0.00839	0.008491	0.022354	-0.0025	0.008974	0.032298	0.002855	0.017791	0.032194	0.025847	0.049502
376	-0.00829	0.008421	0.022501	-0.00272	0.008872	0.032847	0.002799	0.017555	0.032557	0.026069	0.049507
375	-0.00872	0.009048	0.022747	-0.00212	0.0089	0.033041	0.002987	0.017958	0.032908	0.026448	0.050252
374	-0.00812	0.008126	0.021759	-0.0027	0.008199	0.032245	0.002219	0.017253	0.032313	0.025379	0.049471
373	-0.00911	0.007957	0.021894	-0.00315	0.008303	0.032405	0.002174	0.017246	0.03245	0.02545	0.049295
372	-0.00927	0.007782	0.021381	-0.0033	0.0076	0.031578	0.001769	0.016879	0.032254	0.02526	0.049773
371	-0.00927	0.00799	0.021603	-0.00348	0.008536	0.032258	0.002062	0.017075	0.032099	0.025566	0.050092
370	-0.00947	0.008032	0.021495	-0.00361	0.007953	0.032343	0.001648	0.01722	0.032529	0.025548	0.049938
369	-0.01012	0.007774	0.021696	-0.00395	0.007835	0.032123	0.001496	0.01725	0.032429	0.025775	0.050368
368	-0.00419	0.013782	0.027485	0.002153	0.014032	0.03833	0.007329	0.02317	0.038655	0.03165	0.056675
367	-0.01	0.007889	0.021815	-0.00403	0.008126	0.032366	0.001894	0.017358	0.032748	0.025714	0.05108
366	-0.00986	0.007658	0.021916	-0.00376	0.008061	0.032655	0.001496	0.017467	0.033277	0.02581	0.050731

EXCLI Journal 2020;19:48-70 – ISSN 1611-2156
Received: October 03, 2019, accepted: December 19, 2019, published: January 03, 2020

Absorption intensity											
**Wavelength (nm)**	**concentration of α-methylbutyrylshikon (M)**										
	A	*B*	*C*	*D*	*E*	*F*	*G*	*I*	*J*	*K*	*L*
	0.00	$8.0x10^{-7}$	$1.6x10^{-6}$	$2.0x10^{-6}$	$2.8x10^{-6}$	$4.0x10^{-6}$	$4.8x10^{-6}$	$6.0x10^{-6}$	$8.0x10^{-6}$	$1.2x10^{-5}$	$1.6x10^{-5}$
365	-0.01006	0.007944	0.021381	-0.0041	0.0074	0.032465	0.001574	0.01727	0.032292	0.025571	0.050882
364	-0.01042	0.007841	0.021628	-0.00422	0.007445	0.032664	0.001329	0.017275	0.032801	0.025991	0.051654
363	-0.01107	0.007209	0.021651	-0.00454	0.007936	0.033042	0.001043	0.01706	0.032931	0.02639	0.051098
362	-0.0109	0.006979	0.020914	-0.00447	0.007253	0.032557	0.000615	0.017041	0.032551	0.025659	0.051933
361	-0.01105	0.00744	0.021869	-0.00522	0.007404	0.033655	0.000835	0.018184	0.034497	0.027167	0.05352
360	-0.01001	0.008814	0.020732	-0.00356	0.009051	0.031792	0.002513	0.017165	0.033179	0.025946	0.052376
359	-0.01211	0.006263	0.020236	-0.00642	0.00649	0.032251	-0.00027	0.016133	0.032453	0.02544	0.051389
358	-0.0125	0.006616	0.020483	-0.0064	0.006415	0.032826	0.000254	0.016787	0.032602	0.025494	0.053095
357	-0.01212	0.006198	0.020676	-0.00599	0.006461	0.031793	-0.00057	0.016556	0.033561	0.025217	0.053059
356	-0.01363	0.005749	0.021493	-0.00689	0.005514	0.033235	-0.00016	0.017543	0.034429	0.026378	0.052824
355	-0.01103	0.007835	0.019294	-0.00455	0.00812	0.030741	0.001412	0.016525	0.032515	0.024858	0.051979
354	-0.01401	0.004308	0.018472	-0.0076	0.006167	0.03119	-0.00153	0.015273	0.032203	0.025963	0.051407
353	-0.01414	0.005112	0.019991	-0.00857	0.004169	0.031907	-0.00139	0.016613	0.032247	0.025069	0.052354
352	-0.01513	0.004045	0.019304	-0.00815	0.005667	0.032778	-0.00064	0.015264	0.031277	0.025854	0.052381
351	-0.0117	0.005991	0.021852	-0.00628	0.007328	0.033582	0.000171	0.017522	0.034351	0.028322	0.056695
350	-0.01534	0.004372	0.018442	-0.00892	0.004503	0.031279	-0.00282	0.015058	0.031145	0.024426	0.052353
349	-0.01634	0.002743	0.019188	-0.00972	0.005059	0.031103	-0.00259	0.015714	0.032409	0.024541	0.052859
348	-0.00234	0.017747	0.034272	0.003984	0.018346	0.044932	0.010576	0.029154	0.044436	0.039358	0.068536
347	-0.00095	0.019266	0.034867	0.00514	0.019001	0.046218	0.011416	0.030543	0.045462	0.040965	0.0697
346	-0.00132	0.0198	0.035484	0.00512	0.019021	0.045938	0.012209	0.030353	0.045853	0.04146	0.069871
345	-0.00089	0.019718	0.03631	0.006325	0.019542	0.046782	0.012566	0.031305	0.046178	0.041985	0.071232
344	-0.00067	0.019754	0.035825	0.005787	0.019817	0.046835	0.013092	0.03143	0.047463	0.042311	0.07248
343	-0.00049	0.020077	0.036727	0.006249	0.020491	0.048057	0.013447	0.032177	0.047689	0.043367	0.073258
342	-0.00056	0.020814	0.03646	0.006644	0.020729	0.048321	0.013296	0.032673	0.048528	0.044092	0.074251
341	-0.00023	0.020655	0.036854	0.0068	0.02059	0.049038	0.01347	0.032889	0.048839	0.044174	0.076081
340	7.52E-05	0.020861	0.037661	0.007251	0.021376	0.049144	0.013894	0.033518	0.049555	0.044843	0.076485
339	0.000288	0.021088	0.037926	0.006851	0.021316	0.050134	0.014496	0.03366	0.049949	0.046609	0.077538
338	3.24E-05	0.021261	0.038422	0.007255	0.021838	0.050552	0.014986	0.034889	0.050875	0.047146	0.079386
337	0.000842	0.022347	0.03836	0.007923	0.021954	0.050659	0.015057	0.035239	0.05171	0.047518	0.080402
336	0.001304	0.02161	0.039635	0.007966	0.023053	0.05132	0.01542	0.036307	0.052313	0.048771	0.08102
335	0.00095	0.022717	0.039215	0.008411	0.022831	0.05189	0.015393	0.036251	0.052725	0.048999	0.082291
334	0.001738	0.022675	0.039273	0.008361	0.023612	0.052736	0.016456	0.036893	0.053231	0.050028	0.083183
333	0.001748	0.022807	0.040249	0.008832	0.023718	0.053281	0.016459	0.037562	0.053976	0.051236	0.085231
332	0.002136	0.022994	0.0401	0.009247	0.024597	0.05421	0.017197	0.038006	0.054555	0.051498	0.086182
331	0.002392	0.023347	0.040877	0.009115	0.024704	0.054512	0.017247	0.038985	0.055521	0.051862	0.08742
330	0.002167	0.023326	0.041085	0.009631	0.025215	0.054939	0.017765	0.039167	0.0562	0.053891	0.08878
329	0.00281	0.023672	0.041779	0.009671	0.024867	0.055433	0.018126	0.039543	0.057064	0.054087	0.089636

EXCLI Journal 2020;19:48-70 – ISSN 1611-2156
Received: October 03, 2019, accepted: December 19, 2019, published: January 03, 2020

	Absorption intensity										
**Wavelength (nm)**	**concentration of α-methylbutyrylshikon (M)**										
	A	*B*	*C*	*D*	*E*	*F*	*G*	*I*	*J*	*K*	*L*
	0.00	$8.0x10^{-7}$	$1.6x10^{-6}$	$2.0x10^{-6}$	$2.8x10^{-6}$	$4.0x10^{-6}$	$4.8x10^{-6}$	$6.0x10^{-6}$	$8.0x10^{-6}$	$1.2x10^{-5}$	$1.6x10^{-5}$
328	0.002305	0.02444	0.042127	0.009995	0.025443	0.056169	0.01868	0.040939	0.057408	0.055539	0.091088
327	0.003073	0.024631	0.04202	0.010754	0.026388	0.056907	0.018876	0.041246	0.058417	0.055769	0.092316
326	0.003317	0.024972	0.042768	0.010537	0.026775	0.057973	0.019325	0.041753	0.059414	0.056959	0.093747
325	0.003554	0.025037	0.042922	0.010883	0.026585	0.058451	0.019781	0.042808	0.059879	0.058273	0.094933
324	0.003828	0.02504	0.043138	0.010714	0.027093	0.058956	0.019751	0.042785	0.060774	0.059372	0.096065
323	0.004042	0.026208	0.043859	0.011131	0.027643	0.060133	0.020552	0.043743	0.061609	0.060599	0.097816
322	0.004663	0.026457	0.044185	0.011545	0.02819	0.060559	0.02095	0.044456	0.062537	0.061293	0.099476
321	0.00401	0.026246	0.044291	0.011937	0.028316	0.061089	0.021288	0.045066	0.063394	0.062687	0.100989
320	0.00444	0.026659	0.044596	0.012036	0.0294	0.062322	0.02177	0.046098	0.064041	0.06344	0.10282
319	0.005332	0.02686	0.044911	0.01236	0.029823	0.062816	0.022662	0.046431	0.064993	0.064486	0.104294
318	0.005248	0.026914	0.045791	0.012766	0.029875	0.063484	0.022896	0.047098	0.066421	0.065639	0.105946
317	0.005446	0.027672	0.046001	0.012775	0.030516	0.064781	0.023195	0.048422	0.067075	0.066898	0.107561
316	0.005844	0.027991	0.046571	0.013064	0.031076	0.065279	0.023662	0.048996	0.067749	0.067998	0.109076
315	0.006082	0.028332	0.046969	0.013639	0.031431	0.066128	0.024406	0.049693	0.06856	0.069391	0.110866
314	0.00629	0.028619	0.048007	0.014363	0.031948	0.066793	0.025119	0.050506	0.069262	0.070244	0.112903
313	0.006728	0.028847	0.047887	0.014464	0.032452	0.067872	0.025308	0.051546	0.07089	0.07198	0.114481
312	0.00695	0.029309	0.048292	0.01477	0.032966	0.068286	0.025895	0.051908	0.071606	0.072792	0.116142
311	0.007266	0.029298	0.04911	0.015297	0.03372	0.069542	0.026469	0.052455	0.072782	0.074576	0.118122
310	0.007646	0.029835	0.049332	0.015724	0.034313	0.069965	0.027116	0.053792	0.073862	0.075713	0.119738
309	0.007682	0.03076	0.049796	0.016042	0.034688	0.071399	0.027432	0.054589	0.074772	0.076566	0.121927
308	0.007492	0.029667	0.049609	0.015877	0.034691	0.071733	0.02771	0.055005	0.075057	0.077467	0.123561
307	0.00888	0.031418	0.051236	0.016957	0.036013	0.072948	0.028857	0.056869	0.076872	0.079712	0.125668
306	0.008762	0.031936	0.05147	0.017488	0.036855	0.073851	0.029848	0.058	0.078074	0.080852	0.127677
305	0.009523	0.032355	0.052276	0.018271	0.037094	0.074765	0.030584	0.059021	0.078828	0.082726	0.129466
304	0.010069	0.033334	0.053321	0.018902	0.038227	0.076332	0.031624	0.059889	0.080864	0.084379	0.131731
303	0.010197	0.033745	0.053708	0.01866	0.039042	0.077977	0.032203	0.061105	0.081786	0.086158	0.133673
302	0.0112	0.034718	0.054586	0.020103	0.039864	0.07856	0.033345	0.062309	0.083618	0.087435	0.135804
301	0.011776	0.035322	0.055408	0.020849	0.040982	0.079988	0.034311	0.063809	0.084751	0.089317	0.137834
300	0.01249	0.036322	0.056922	0.021715	0.041757	0.081999	0.035608	0.064997	0.085978	0.091332	0.140808
299	0.013536	0.037419	0.057556	0.022742	0.043122	0.083194	0.036451	0.066521	0.088011	0.093022	0.143054
298	0.014262	0.038615	0.059037	0.024035	0.044244	0.085167	0.037814	0.068167	0.089847	0.09548	0.146137
297	0.015644	0.039815	0.060483	0.025226	0.045849	0.08625	0.039722	0.069978	0.091767	0.097586	0.148749
296	0.017313	0.041479	0.06237	0.026787	0.047632	0.088749	0.041306	0.072283	0.094365	0.100293	0.15208
295	0.019168	0.043519	0.064542	0.028714	0.049788	0.091237	0.043553	0.074522	0.09718	0.102906	0.155792
294	0.021356	0.046205	0.067025	0.031155	0.05224	0.094652	0.046355	0.077411	0.10026	0.10625	0.159734
293	0.024092	0.049328	0.070515	0.034544	0.055776	0.098152	0.04993	0.081526	0.104286	0.110916	0.164793
292	0.028009	0.053682	0.074323	0.038577	0.059761	0.102237	0.053955	0.085499	0.108518	0.115245	0.169923

EXCLI Journal 2020;19:48-70 – ISSN 1611-2156
Received: October 03, 2019, accepted: December 19, 2019, published: January 03, 2020

Absorption intensity											
**Wavelength (nm)**	**concentration of α-methylbutyrylshikon (M)**										
	A	*B*	*C*	*D*	*E*	*F*	*G*	*I*	*J*	*K*	*L*
	0.00	8.0×10^{-7}	1.6×10^{-6}	2.0×10^{-6}	2.8×10^{-6}	4.0×10^{-6}	4.8×10^{-6}	6.0×10^{-6}	8.0×10^{-6}	1.2×10^{-5}	1.6×10^{-5}
291	0.032089	0.058313	0.079081	0.043331	0.064844	0.107853	0.059207	0.090699	0.113901	0.120969	0.175931
290	0.036943	0.06369	0.084255	0.048566	0.070063	0.113409	0.06442	0.096441	0.120035	0.126827	0.182285
289	0.042875	0.069611	0.090494	0.054657	0.07625	0.119781	0.070933	0.103144	0.126179	0.133765	0.190023
288	0.048466	0.075828	0.09671	0.060585	0.082503	0.126251	0.077471	0.109664	0.133655	0.140524	0.197565
287	0.053743	0.081716	0.102779	0.066505	0.088558	0.132804	0.083505	0.115893	0.140024	0.147075	0.204671
286	0.058262	0.086291	0.107493	0.071511	0.094017	0.138228	0.088842	0.121467	0.145616	0.152904	0.210935
285	0.061811	0.090791	0.111802	0.075396	0.098025	0.142606	0.092396	0.125998	0.149951	0.157613	0.215953
284	0.064326	0.09332	0.114569	0.078308	0.100942	0.145919	0.095575	0.129467	0.153573	0.161123	0.219934
283	0.066376	0.095972	0.116952	0.080479	0.103279	0.14855	0.098309	0.13192	0.156216	0.16398	0.223716
282	0.067625	0.097791	0.119274	0.082455	0.105389	0.151353	0.100305	0.134453	0.158973	0.166649	0.22681
281	0.069861	0.100057	0.121432	0.08433	0.107366	0.153826	0.102374	0.13669	0.161381	0.169339	0.23008
280	0.071159	0.101708	0.123448	0.086185	0.109699	0.156176	0.104613	0.139052	0.164375	0.172102	0.233069
279	0.072388	0.103728	0.125053	0.087712	0.111542	0.157903	0.106099	0.140989	0.165793	0.174293	0.235581
278	0.072553	0.104211	0.125818	0.088529	0.112124	0.159358	0.106874	0.142054	0.167067	0.17589	0.237733
277	0.072605	0.104289	0.125828	0.088206	0.112182	0.159631	0.107012	0.142591	0.167773	0.175991	0.23889
276	0.071613	0.103745	0.125453	0.087518	0.111672	0.159461	0.106501	0.142013	0.167624	0.17636	0.239435
275	0.070359	0.102736	0.124544	0.08652	0.110855	0.158654	0.105539	0.141426	0.16707	0.176036	0.240192
274	0.068938	0.101445	0.12345	0.085397	0.109644	0.157997	0.104204	0.140696	0.16656	0.175858	0.240059
273	0.067274	0.100653	0.121981	0.083832	0.108747	0.157549	0.103052	0.139724	0.165736	0.17503	0.239946
272	0.065533	0.099173	0.120909	0.082348	0.10787	0.15659	0.102005	0.139002	0.165368	0.174825	0.240272
271	0.064591	0.098268	0.120318	0.081115	0.106628	0.156113	0.101299	0.138235	0.164897	0.1748	0.240906
270	0.063356	0.097537	0.119638	0.080188	0.10627	0.15612	0.100561	0.138192	0.164752	0.174971	0.241949
269	0.061869	0.096597	0.119109	0.079205	0.105572	0.156253	0.100059	0.137916	0.165061	0.175704	0.243082
268	0.060522	0.095527	0.117905	0.078218	0.105196	0.155385	0.098891	0.137903	0.164856	0.176242	0.244156
267	0.058751	0.093875	0.116623	0.076129	0.103756	0.154944	0.097531	0.137141	0.164502	0.176139	0.245575
266	0.057162	0.092592	0.115422	0.07537	0.102679	0.154885	0.096907	0.136747	0.16464	0.17659	0.246854
265	0.055781	0.091727	0.114645	0.074248	0.101958	0.154971	0.09622	0.136403	0.164675	0.177539	0.248627
264	0.054209	0.090018	0.113433	0.072516	0.101051	0.154407	0.094734	0.135953	0.164741	0.178207	0.250522
263	0.051969	0.088537	0.111903	0.071143	0.099572	0.154095	0.093681	0.135425	0.164658	0.178765	0.252058
262	0.050939	0.087274	0.111112	0.069965	0.098904	0.154337	0.093324	0.135326	0.165437	0.180623	0.25492
261	0.050011	0.087017	0.110991	0.069971	0.099025	0.15531	0.093758	0.136445	0.167051	0.183165	0.259276
260	0.049719	0.086857	0.111143	0.069828	0.099603	0.157	0.094245	0.137788	0.169127	0.18607	0.263609
259	0.048985	0.086383	0.110848	0.06948	0.099898	0.158077	0.09467	0.138867	0.17091	0.189595	0.268659
258	0.047721	0.085636	0.110545	0.068887	0.099753	0.15924	0.09475	0.140065	0.172853	0.192518	0.273742
257	0.046028	0.084082	0.10927	0.068041	0.098997	0.160226	0.094745	0.140697	0.17429	0.196098	0.27872
256	0.044216	0.082931	0.108469	0.067359	0.098626	0.161477	0.09461	0.14166	0.176486	0.19983	0.284672
255	0.043197	0.082965	0.108719	0.066887	0.099248	0.163916	0.095724	0.144092	0.179679	0.205163	0.292705

EXCLI Journal 2020;19:48-70 – ISSN 1611-2156
Received: October 03, 2019, accepted: December 19, 2019, published: January 03, 2020

Absorption intensity											
**Wavelength (nm)**	**concentration of α-methylbutyrylshikon (M)**										
	A	*B*	*C*	*D*	*E*	*F*	*G*	*I*	*J*	*K*	*L*
	0.00	$8.0x10^{-7}$	$1.6x10^{-6}$	$2.0x10^{-6}$	$2.8x10^{-6}$	$4.0x10^{-6}$	$4.8x10^{-6}$	$6.0x10^{-6}$	$8.0x10^{-6}$	$1.2x10^{-5}$	$1.6x10^{-5}$
254	0.042859	0.083117	0.109747	0.068022	0.100851	0.167219	0.097935	0.147039	0.184529	0.212361	0.302184
253	0.043	0.083611	0.110991	0.069061	0.102914	0.171882	0.100782	0.151409	0.189954	0.22077	0.31366
252	0.042863	0.083633	0.11192	0.070605	0.105066	0.176793	0.104125	0.156077	0.196407	0.229855	0.327001
251	0.042347	0.084504	0.11335	0.072107	0.108009	0.182171	0.107865	0.161224	0.203808	0.240593	0.341193
250	0.042536	0.085484	0.115719	0.074698	0.110826	0.188718	0.112668	0.167594	0.212182	0.253035	0.358238
249	0.043572	0.087329	0.118921	0.078272	0.115576	0.197673	0.118957	0.176126	0.223068	0.268798	0.37956
248	0.045288	0.090466	0.123546	0.083062	0.121676	0.20881	0.126863	0.186576	0.236685	0.287899	0.404676
247	0.047945	0.094161	0.129396	0.089558	0.129193	0.22231	0.136894	0.199833	0.253515	0.311337	0.435395
246	0.051351	0.099336	0.136453	0.097598	0.138731	0.238656	0.149406	0.216358	0.274143	0.339989	0.472721
245	0.056206	0.105954	0.145877	0.108223	0.151568	0.259925	0.165831	0.236773	0.300169	0.376231	0.520267
244	0.062741	0.114741	0.158313	0.122207	0.167632	0.286962	0.187129	0.263209	0.333235	0.422593	0.580328
243	0.071948	0.126847	0.174593	0.140454	0.189232	0.322747	0.214838	0.298131	0.376911	0.483052	0.658589
242	0.083481	0.142014	0.19639	0.164336	0.216769	0.368821	0.250919	0.343459	0.433253	0.561382	0.759889
241	0.098468	0.161507	0.224142	0.195694	0.253807	0.430375	0.298893	0.403653	0.5081	0.665611	0.893757
240	0.117361	0.186564	0.260495	0.236555	0.301475	0.510539	0.362123	0.48203	0.607303	0.803401	1.070126
239	0.140732	0.219031	0.308146	0.291201	0.365464	0.619234	0.446592	0.58853	0.740109	0.988192	1.305225
238	0.168824	0.259217	0.370235	0.361799	0.448444	0.762925	0.557839	0.728113	0.915646	1.229705	1.612708
237	0.203084	0.309823	0.450181	0.454342	0.558822	0.953966	0.706247	0.914529	1.14858	1.550401	2.012848
236	0.242746	0.371974	0.553033	0.574054	0.701685	1.20371	0.900032	1.158857	1.452846	1.961694	2.520985
235	0.28844	0.447689	0.685754	0.729262	0.887972	1.52851	1.154393	1.476567	1.847343	2.482559	3.141086
234	0.337956	0.53868	0.854362	0.926567	1.127082	1.944672	1.48014	1.883141	2.345766	3.112922	3.780955
233	0.392179	0.648838	1.068435	1.179716	1.433987	2.469525	1.896379	2.393174	2.947533	3.750787	4.247159
232	0.448482	0.780873	1.338921	1.499686	1.820462	3.091278	2.410372	3.004065	3.574753	4.130144	4.397174
231	0.507201	0.941587	1.678336	1.899153	2.301774	3.692486	3.019134	3.61314	4.017828	4.316467	4.601732
230	0.56692	1.133971	2.097841	2.390451	2.876088	4.087843	3.624646	4.02088	4.256321	4.483651	4.739552
229	0.627068	1.371453	2.609289	2.970942	3.484397	4.285311	4.021811	4.231192	4.349779	4.577842	4.950535
228	0.686578	1.659486	3.193503	3.553413	3.925211	4.44777	4.205985	4.345407	4.489796	4.808848	4.898529
227	0.746204	2.007309	3.751103	3.964369	4.186595	4.618001	4.323144	4.501102	4.652955	4.782583	4.989244
226	0.805998	2.425489	4.115396	4.207924	4.334372	4.748904	4.579246	4.711083	4.765745	4.865118	5.378005
225	0.867218	2.917196	4.300344	4.344073	4.466485	4.870963	4.624255	4.686235	4.801532	5.017131	5.129803
224	0.929842	3.44092	4.470024	4.519255	4.546402	4.990077	4.726475	4.866564	4.880361	5.097794	5.598374
223	0.997268	3.953358	4.596164	4.639909	4.642733	4.963117	4.835247	4.94039	4.919302	5.044481	5.412269
222	1.069929	4.253151	4.732453	4.620508	4.665182	5.084499	4.832541	4.883143	5.019423	5.100045	5.313781
221	1.14952	4.388629	4.856319	4.679859	4.842173	5.06373	5.063932	4.91663	5.156838	5.64355	5.342395
220	1.233891	4.582782	4.767781	4.860933	4.88091	5.313572	4.992167	4.97896	5.357774	5.034073	5.763355
219	1.321413	4.665208	4.844426	4.982776	4.907935	5.175868	4.989549	5.256851	5.047931	5.154987	5.7468
218	1.408007	4.702094	4.851301	5.006968	5.046072	5.238473	4.963833	5.097649	5.15727	5.178998	5.389555

C

EXCLI Journal 2020;19:48-70 – ISSN 1611-2156
Received: October 03, 2019, accepted: December 19, 2019, published: January 03, 2020

Absorption intensity											
**Wavelength (nm)**	**concentration of α-methylbutyrylshikon (M)**										
	A	*B*	*C*	*D*	*E*	*F*	*G*	*I*	*J*	*K*	*L*
	0.00	$8.0x10^{-7}$	$1.6x10^{-6}$	$2.0x10^{-6}$	$2.8x10^{-6}$	$4.0x10^{-6}$	$4.8x10^{-6}$	$6.0x10^{-6}$	$8.0x10^{-6}$	$1.2x10^{-5}$	$1.6x10^{-5}$
217	1.498462	4.733318	4.924173	5.042678	5.03416	5.218282	5.75022	5.231277	5.181313	5.750015	5.351801
216	1.593242	5.074935	5.125894	5.064956	5.094267	5.074403	5.613817	5.613739	5.208841	5.949483	5.183645
215	1.696832	4.806723	5.070705	5.124221	5.448731	5.527582	5.402916	5.198497	5.589735	5.402549	10
214	1.806565	5.173553	5.201497	5.216175	4.922685	4.907403	6.201091	5.56421	5.56407	5.636963	5.422653
213	1.924397	5.308602	5.203876	4.969692	5.145795	10	5.650989	5.23566	5.252604	5.747433	5.951503
212	2.051074	4.922208	5.259701	5.223109	10	10	5.07689	5.102079	5.666371	10	10
211	2.188238	5.473866	5.508704	5.225705	5.28663	5.63273	10	10	5.58651	5.586987	5.808585
210	2.344379	5.765062	10	6.13231	6.007772	10	10	10	10	10	10
209	2.501616	5.598737	5.467419	5.573478	5.65275	5.309755	10	5.74851	10	10	10
208	2.680004	5.022541	4.951037	5.292935	4.964104	5.136399	5.178617	5.337664	5.44635	5.689833	5.106231
207	2.879187	4.869788	5.082843	4.946817	5.415955	5.247072	5.41539	5.149232	5.090898	5.278256	5.330842
206	3.079413	4.810175	4.879708	5.014596	5.014982	5.014539	5.195865	4.860625	5.212079	5.638341	5.477887
205	3.305117	5.177814	5.674779	5.439642	5.564698	5.673089	10	5.173728	10	10	10
204	3.439513	4.639799	4.810343	4.997831	5.319929	5.340444	5.072232	5.524933	5.093243	5.207243	5.595912
203	3.516747	4.585402	4.418844	4.407487	4.981666	4.900469	4.937371	4.729018	5.110271	4.790544	5.130215
202	3.422569	4.102446	4.515691	4.204642	4.002168	4.383593	4.278458	4.164167	10	4.756448	5.039361
201	3.035921	3.539993	3.441703	3.500517	3.712939	3.721669	3.599674	3.723301	3.807222	3.808356	3.846478
200	2.446951	3.08038	2.997974	2.965567	2.945024	2.919963	2.81275	2.853777	2.925633	3.10287	3.11534

EXCLI Journal 2020;19:48-70 – ISSN 1611-2156
Received: October 03, 2019, accepted: December 19, 2019, published: January 03, 2020

Table 2: Absorption intensity in the wavelength range of 200-800 nm for absorption spectra of HSA fixed concentration (2.00 x 10^{-6} M), in the absence (*A*) and presence of increasing concentration of acetylshikonin (*B-L*)

Absorption intensity											
**Wavelength (nm)**	**concentration of acetylshikon (M)**										
	A	*B*	*C*	*D*	*E*	*F*	*G*	*I*	*J*	*K*	*L*
	0.00	8.0x10^{-7}	1.6x10^{-6}	2.0x10^{-6}	2.8x10^{-6}	4.0x10^{-6}	4.8x10^{-6}	6.0x10^{-6}	8.0x10^{-6}	1.2x10^{-5}	1.6x10^{-5}
800	0.002492	0.003833	0.003153	0.005876	0.005903	0.003378	0.005487	0.005541	0.004411	0.008709	0.00565
799	0.003645	0.004887	0.003951	0.006761	0.006662	0.004536	0.006348	0.006818	0.005785	0.009751	0.006722
798	0.003328	0.004747	0.003993	0.006761	0.006667	0.00449	0.006482	0.006643	0.005482	0.009764	0.006685
797	0.003317	0.004313	0.004101	0.006576	0.006952	0.004448	0.006392	0.006708	0.005391	0.009437	0.006653
796	0.003659	0.004875	0.003862	0.006522	0.006588	0.004148	0.006406	0.006718	0.005396	0.009734	0.006782
795	0.003428	0.004673	0.003959	0.006483	0.006752	0.004379	0.006549	0.00683	0.005385	0.009748	0.006585
794	0.003535	0.005115	0.004436	0.006833	0.006958	0.004453	0.006443	0.006738	0.005539	0.009582	0.006648
793	0.003516	0.004774	0.003899	0.006708	0.00669	0.004356	0.006626	0.006569	0.005065	0.009441	0.006769
792	0.00363	0.004566	0.004009	0.006606	0.006868	0.004606	0.006599	0.006539	0.005693	0.009718	0.006613
791	0.003428	0.004862	0.003896	0.006919	0.006964	0.00434	0.006715	0.006436	0.005521	0.009884	0.006684
790	0.003543	0.004619	0.004442	0.006814	0.006734	0.004532	0.006575	0.006604	0.005517	0.009771	0.006745
789	0.003516	0.004715	0.004143	0.006551	0.006764	0.004444	0.006774	0.006844	0.00545	0.009764	0.006828
788	0.003306	0.004887	0.004292	0.006929	0.006775	0.004655	0.006533	0.00668	0.005443	0.009743	0.006566
787	0.003761	0.004719	0.004112	0.00674	0.006968	0.004418	0.006602	0.006691	0.005646	0.009514	0.006724
786	0.003583	0.004585	0.004144	0.006932	0.006525	0.004308	0.006465	0.006871	0.005599	0.009789	0.006902
785	0.003559	0.004755	0.004163	0.006585	0.006696	0.004302	0.0068	0.006618	0.005515	0.009816	0.006892
784	0.00383	0.00472	0.004088	0.006538	0.006999	0.004398	0.006555	0.006952	0.005515	0.010074	0.006811
783	0.003718	0.004866	0.003998	0.006781	0.006553	0.004259	0.006725	0.006921	0.005429	0.009847	0.006641
782	0.003657	0.004738	0.004029	0.006895	0.006778	0.004438	0.006459	0.006838	0.005673	0.009981	0.006902
781	0.003533	0.004678	0.004163	0.006634	0.006768	0.004543	0.006415	0.006942	0.005646	0.009896	0.00649
780	0.003363	0.004681	0.004219	0.006798	0.006483	0.004418	0.006431	0.006896	0.005416	0.009799	0.006785
779	0.003668	0.004774	0.00396	0.006574	0.00682	0.004259	0.00639	0.006895	0.00551	0.009768	0.006985
778	0.003463	0.004782	0.004061	0.00675	0.006794	0.004159	0.006515	0.006834	0.005612	0.010074	0.006976
777	0.00369	0.004968	0.003958	0.006894	0.006933	0.004516	0.006647	0.006933	0.005501	0.009798	0.007224
776	0.003382	0.004656	0.004264	0.006798	0.006887	0.004308	0.006453	0.006921	0.005331	0.00999	0.006871
775	0.003653	0.004696	0.003929	0.006862	0.006986	0.004406	0.006577	0.006685	0.005631	0.009971	0.00703
774	0.00365	0.005149	0.004162	0.0068	0.006759	0.004297	0.006554	0.006898	0.005586	0.010177	0.007007
773	0.003581	0.004754	0.004118	0.006847	0.006728	0.004353	0.006697	0.006889	0.005617	0.009924	0.006994
772	0.00336	0.004693	0.004183	0.006838	0.006801	0.004477	0.006709	0.006811	0.005746	0.009957	0.007069
771	0.003422	0.00482	0.004077	0.006722	0.0068	0.004483	0.006533	0.006836	0.005576	0.010067	0.006795

EXCLI Journal 2020;19:48-70 – ISSN 1611-2156
Received: October 03, 2019, accepted: December 19, 2019, published: January 03, 2020

Absorption intensity											
**Wavelength (nm)**	**concentration of acetylshikon (M)**										
	A	*B*	*C*	*D*	*E*	*F*	*G*	*I*	*J*	*K*	*L*
	0.00	$8.0x10^{-7}$	$1.6x10^{-6}$	$2.0x10^{-6}$	$2.8x10^{-6}$	$4.0x10^{-6}$	$4.8x10^{-6}$	$6.0x10^{-6}$	$8.0x10^{-6}$	$1.2x10^{-5}$	$1.6x10^{-5}$
770	0.00347	0.00467	0.004165	0.006994	0.006838	0.004432	0.006609	0.006744	0.005778	0.01006	0.007132
769	0.003545	0.004676	0.004213	0.00667	0.006856	0.004542	0.006765	0.006733	0.005561	0.009893	0.006973
768	0.003322	0.004608	0.004274	0.006848	0.006818	0.004649	0.006678	0.006918	0.005559	0.010212	0.006893
767	0.003537	0.004753	0.003996	0.006694	0.007039	0.004312	0.006506	0.006734	0.005671	0.010099	0.007119
766	0.003228	0.004784	0.00411	0.006722	0.006714	0.0044	0.006583	0.006857	0.005513	0.010188	0.007104
765	0.003428	0.004805	0.003728	0.006757	0.006824	0.004492	0.006685	0.006861	0.00565	0.010022	0.007339
764	0.003465	0.004672	0.004026	0.006782	0.006786	0.004292	0.00659	0.007067	0.005643	0.010006	0.007242
763	0.003584	0.004762	0.004066	0.006675	0.006866	0.004451	0.006683	0.006824	0.00578	0.010178	0.007517
762	0.003483	0.004782	0.004093	0.006791	0.006962	0.004647	0.006867	0.006893	0.005752	0.010046	0.007283
761	0.003686	0.004669	0.004079	0.006693	0.006792	0.004671	0.00681	0.007082	0.005847	0.010071	0.007193
760	0.003453	0.004982	0.0041	0.006812	0.006868	0.004434	0.006695	0.00697	0.005496	0.010173	0.007385
759	0.003827	0.004795	0.004096	0.006878	0.006919	0.00456	0.006747	0.007171	0.005588	0.010435	0.007424
758	0.003637	0.00481	0.004105	0.006804	0.007077	0.004558	0.006841	0.007079	0.005743	0.010348	0.007471
757	0.003231	0.004813	0.0042	0.00682	0.006941	0.004573	0.00672	0.006969	0.005714	0.010175	0.007515
756	0.003437	0.004801	0.00404	0.006675	0.007052	0.004582	0.006633	0.006961	0.005677	0.010292	0.00726
755	0.003407	0.004521	0.003967	0.006815	0.006908	0.00462	0.006572	0.006877	0.005616	0.010523	0.007429
754	0.00333	0.004813	0.004184	0.006828	0.006814	0.004671	0.006645	0.00702	0.005814	0.010413	0.007463
753	0.00323	0.004689	0.004027	0.006732	0.006809	0.004741	0.006827	0.007108	0.005799	0.010399	0.007469
752	0.003531	0.00479	0.004286	0.006764	0.006978	0.004514	0.006883	0.006998	0.005685	0.010646	0.007458
751	0.003523	0.004907	0.004137	0.006885	0.006919	0.004716	0.006728	0.007208	0.005863	0.010557	0.007697
750	0.003563	0.0048	0.004219	0.006867	0.006879	0.004692	0.006918	0.007196	0.005735	0.010665	0.007723
749	0.003325	0.004833	0.004071	0.006782	0.006953	0.004693	0.006779	0.007041	0.005743	0.010795	0.007792
748	0.003454	0.004793	0.004142	0.006816	0.006983	0.004662	0.006888	0.007172	0.005936	0.01051	0.007767
747	0.003472	0.004811	0.004264	0.006835	0.006927	0.004747	0.006635	0.007145	0.005811	0.010605	0.007637
746	0.003538	0.004711	0.004145	0.006934	0.006967	0.004658	0.006919	0.00719	0.00601	0.01095	0.007946
745	0.003472	0.004721	0.004116	0.007027	0.006955	0.004624	0.006912	0.007177	0.00607	0.010619	0.007813
744	0.003403	0.004732	0.004173	0.006895	0.006992	0.004644	0.006859	0.007304	0.005935	0.010695	0.007907
743	0.00325	0.00487	0.004116	0.007001	0.00689	0.004612	0.006991	0.007115	0.006037	0.01076	0.00779
742	0.003447	0.004758	0.004057	0.006953	0.007107	0.004687	0.006791	0.007048	0.005972	0.010819	0.008026
741	0.003351	0.004751	0.004062	0.006824	0.006971	0.004587	0.00676	0.007245	0.005947	0.010828	0.007957
740	0.003562	0.004887	0.00433	0.006941	0.007141	0.004636	0.006953	0.007372	0.006004	0.010946	0.007889
739	0.003549	0.004917	0.004168	0.00693	0.007065	0.004664	0.006909	0.007398	0.006014	0.010803	0.008122
738	0.003352	0.00475	0.004168	0.006824	0.007075	0.004781	0.006933	0.007314	0.006007	0.011137	0.008215

EXCLI Journal 2020;19:48-70 – ISSN 1611-2156
Received: October 03, 2019, accepted: December 19, 2019, published: January 03, 2020

Absorption intensity											
**Wavelength (nm)**	**concentration of acetylshikon (M)**										
	A	*B*	*C*	*D*	*E*	*F*	*G*	*I*	*J*	*K*	*L*
	0.00	$8.0x10^{-7}$	$1.6x10^{-6}$	$2.0x10^{-6}$	$2.8x10^{-6}$	$4.0x10^{-6}$	$4.8x10^{-6}$	$6.0x10^{-6}$	$8.0x10^{-6}$	$1.2x10^{-5}$	$1.6x10^{-5}$
737	0.003332	0.004784	0.004094	0.006994	0.007049	0.004739	0.00701	0.007465	0.006001	0.011141	0.008394
736	0.003341	0.004593	0.004213	0.00707	0.007205	0.004872	0.007026	0.007592	0.006233	0.011189	0.008497
735	0.003362	0.004796	0.004078	0.00716	0.007054	0.004721	0.006994	0.007601	0.006239	0.01128	0.008506
734	0.003413	0.004947	0.004124	0.007012	0.006986	0.005013	0.007115	0.007642	0.006255	0.011325	0.008806
733	0.003362	0.004737	0.004018	0.0069	0.007199	0.004772	0.007195	0.007804	0.006373	0.011454	0.008837
732	0.00343	0.004681	0.004112	0.006899	0.007193	0.004797	0.007172	0.007497	0.006255	0.011582	0.008954
731	0.003267	0.004766	0.00394	0.006964	0.006952	0.004801	0.007167	0.007629	0.006293	0.01155	0.009185
730	0.00317	0.004744	0.004042	0.006855	0.007064	0.004862	0.006961	0.007798	0.006492	0.011697	0.009292
729	0.003079	0.004673	0.003897	0.006694	0.006876	0.004853	0.006996	0.007617	0.006344	0.011628	0.009226
728	0.003038	0.004613	0.00385	0.006774	0.006966	0.004777	0.007063	0.007649	0.006267	0.011906	0.009305
727	0.002972	0.004451	0.003763	0.006683	0.006741	0.004746	0.006913	0.007672	0.006245	0.01167	0.009259
726	0.0029	0.004325	0.003684	0.006673	0.00687	0.004555	0.006806	0.007603	0.006245	0.011792	0.009321
725	0.002854	0.004188	0.003686	0.0065	0.006526	0.004406	0.006859	0.007462	0.006059	0.011578	0.009208
724	0.002587	0.004011	0.003533	0.006453	0.006519	0.004386	0.006635	0.007468	0.006168	0.011689	0.00907
723	0.002398	0.003881	0.003327	0.006319	0.006406	0.004269	0.006376	0.007241	0.00605	0.011481	0.009126
722	0.002177	0.003674	0.003123	0.00627	0.00626	0.004213	0.006468	0.007275	0.005748	0.011437	0.009029
721	0.002092	0.003678	0.003048	0.006069	0.006166	0.004009	0.006201	0.006936	0.005511	0.01133	0.008989
720	0.001842	0.003283	0.002883	0.005907	0.005935	0.003916	0.006046	0.006958	0.005489	0.011178	0.008899
719	0.001753	0.003082	0.002591	0.005666	0.005818	0.00378	0.005912	0.006736	0.005281	0.011071	0.00877
718	0.00177	0.003259	0.002649	0.005572	0.005811	0.003644	0.006058	0.006923	0.005324	0.011259	0.008923
717	0.001712	0.003284	0.002649	0.005607	0.005935	0.003795	0.005889	0.006874	0.005467	0.011192	0.009008
716	0.001458	0.002908	0.002323	0.005543	0.005646	0.003447	0.005834	0.006583	0.005404	0.01095	0.008812
715	0.001307	0.00276	0.002322	0.005249	0.005463	0.003393	0.005627	0.006484	0.004998	0.011103	0.008792
714	0.001055	0.002606	0.001943	0.005144	0.005174	0.003057	0.005553	0.006288	0.004881	0.010924	0.008709
713	0.00093	0.002346	0.001873	0.00494	0.005202	0.003163	0.005231	0.006202	0.004767	0.010688	0.008608
712	0.000743	0.002204	0.001523	0.004949	0.004915	0.00305	0.00525	0.006209	0.004916	0.010731	0.008579
711	0.000524	0.002206	0.001513	0.004551	0.004837	0.003031	0.005098	0.006052	0.004482	0.010746	0.008777
710	0.000371	0.0021	0.001449	0.004578	0.004767	0.002776	0.005283	0.005926	0.004652	0.010683	0.008596
709	0.000194	0.001984	0.001302	0.004502	0.004657	0.002738	0.005053	0.00594	0.004586	0.010726	0.00871
708	0.000263	0.001854	0.001338	0.004447	0.00459	0.002707	0.004823	0.006085	0.004515	0.010669	0.008732
707	0.000134	0.001748	0.001221	0.004479	0.004569	0.002677	0.005086	0.005884	0.004493	0.010665	0.008678
706	0.000293	0.001813	0.001207	0.004315	0.004546	0.002672	0.005032	0.005926	0.004475	0.010713	0.008935
705	9.98E-05	0.00182	0.0013	0.004371	0.004595	0.002626	0.004959	0.005833	0.004475	0.010736	0.00874

EXCLI Journal 2020;19:48-70 – ISSN 1611-2156
Received: October 03, 2019, accepted: December 19, 2019, published: January 03, 2020

Absorption intensity											
**Wavelength (nm)**	**concentration of acetylshikon (M)**										
	A	*B*	*C*	*D*	*E*	*F*	*G*	*I*	*J*	*K*	*L*
	0.00	$8.0x10^{-7}$	$1.6x10^{-6}$	$2.0x10^{-6}$	$2.8x10^{-6}$	$4.0x10^{-6}$	$4.8x10^{-6}$	$6.0x10^{-6}$	$8.0x10^{-6}$	$1.2x10^{-5}$	$1.6x10^{-5}$
704	0.000173	0.00169	0.001242	0.004399	0.004574	0.002724	0.004927	0.006038	0.004606	0.010748	0.009014
703	0.000217	0.001813	0.001337	0.004384	0.004727	0.002814	0.005105	0.006053	0.004594	0.010993	0.009271
702	9.46E-05	0.001726	0.00139	0.004559	0.004717	0.002837	0.004997	0.006278	0.00474	0.011116	0.009407
701	0.000224	0.001997	0.001367	0.004552	0.004819	0.00291	0.005158	0.006344	0.004821	0.011333	0.009609
700	0.000216	0.001933	0.001276	0.00463	0.004814	0.002989	0.005199	0.006435	0.004802	0.011375	0.009652
699	0.000207	0.001899	0.001322	0.004777	0.004812	0.003197	0.005277	0.006437	0.004962	0.011583	0.009888
698	0.000407	0.002014	0.00151	0.004883	0.005118	0.003288	0.005308	0.006656	0.005203	0.011858	0.010225
697	0.000398	0.002116	0.001641	0.004903	0.005072	0.003221	0.005542	0.006766	0.005203	0.011935	0.010402
696	0.000533	0.002302	0.001825	0.004865	0.005337	0.003289	0.005665	0.007108	0.005359	0.012352	0.010639
695	0.000601	0.002242	0.001863	0.004887	0.005291	0.003555	0.005834	0.007034	0.005519	0.012311	0.010763
694	0.00079	0.002332	0.002097	0.005121	0.005293	0.003721	0.005902	0.007124	0.005686	0.012561	0.011003
693	0.000744	0.002551	0.002015	0.005203	0.005606	0.003736	0.005768	0.007334	0.005934	0.012813	0.011297
692	0.000719	0.002674	0.00222	0.005331	0.005699	0.003824	0.006105	0.007465	0.00599	0.012763	0.011643
691	0.000793	0.002548	0.002295	0.005298	0.005522	0.003891	0.006225	0.007614	0.00608	0.013166	0.011725
690	0.000809	0.002749	0.002368	0.00554	0.00585	0.004205	0.006347	0.007728	0.006202	0.013204	0.01191
689	0.000889	0.002844	0.002411	0.00546	0.006006	0.004235	0.006175	0.007798	0.006292	0.013239	0.012351
688	0.000923	0.002817	0.002432	0.005503	0.005904	0.004334	0.006504	0.008154	0.006467	0.013629	0.012419
687	0.000987	0.002914	0.002415	0.005634	0.005945	0.004466	0.006484	0.007975	0.006706	0.013907	0.012793
686	0.000943	0.003004	0.002416	0.005626	0.006258	0.004538	0.006504	0.008352	0.006381	0.014022	0.012913
685	0.000899	0.00288	0.002446	0.005812	0.005873	0.004445	0.006789	0.00853	0.006672	0.014298	0.013202
684	0.259763	0.26195	0.002522	0.005697	0.00588	0.264238	0.006782	0.008428	0.006863	0.014329	0.013405
683	0.001004	0.003109	0.002665	0.005672	0.006177	0.004579	0.006697	0.008319	0.006719	0.01406	0.013249
682	0.001264	0.00289	0.002721	0.005768	0.006325	0.004802	0.007017	0.008685	0.007017	0.014342	0.013659
681	0.001086	0.003015	0.002781	0.005875	0.006392	0.00499	0.007004	0.00881	0.007113	0.014707	0.014082
680	0.001172	0.002971	0.002723	0.005963	0.006385	0.005083	0.006949	0.009019	0.007121	0.014936	0.014184
679	0.001131	0.003132	0.002961	0.005981	0.006342	0.005141	0.006997	0.009057	0.00731	0.01522	0.014393
678	0.001192	0.00322	0.002813	0.006053	0.006501	0.005292	0.00729	0.009149	0.007404	0.015305	0.014678
677	0.001178	0.00329	0.002929	0.006013	0.006667	0.005203	0.00727	0.00937	0.007529	0.01545	0.014972
676	0.001269	0.003224	0.002968	0.006262	0.006621	0.005382	0.007269	0.009315	0.007587	0.015708	0.015333
675	0.00118	0.003016	0.003103	0.006198	0.006626	0.005424	0.007445	0.009543	0.007652	0.015877	0.015402
674	0.001139	0.003182	0.003071	0.006228	0.006767	0.005504	0.007503	0.009527	0.007805	0.016215	0.015802
673	0.001361	0.003227	0.002978	0.006311	0.006659	0.005508	0.007491	0.009725	0.007927	0.016181	0.015974
672	0.001289	0.003283	0.003041	0.006365	0.00672	0.005724	0.007695	0.009926	0.008046	0.016524	0.016168

EXCLI Journal 2020;19:48-70 – ISSN 1611-2156
Received: October 03, 2019, accepted: December 19, 2019, published: January 03, 2020

Absorption intensity											
**Wavelength (nm)**	**concentration of acetylshikon (M)**										
	A	*B*	*C*	*D*	*E*	*F*	*G*	*I*	*J*	*K*	*L*
	0.00	$8.0x10^{-7}$	$1.6x10^{-6}$	$2.0x10^{-6}$	$2.8x10^{-6}$	$4.0x10^{-6}$	$4.8x10^{-6}$	$6.0x10^{-6}$	$8.0x10^{-6}$	$1.2x10^{-5}$	$1.6x10^{-5}$
671	0.001329	0.003431	0.003345	0.006419	0.007072	0.005808	0.007912	0.010176	0.008176	0.016731	0.016425
670	0.001804	0.003481	0.003747	0.006692	0.007404	0.006096	0.008267	0.010506	0.008515	0.017199	0.016946
669	0.002011	0.004039	0.003863	0.007084	0.00764	0.006527	0.008554	0.010953	0.008847	0.017715	0.017621
668	0.001955	0.004042	0.004048	0.007181	0.007803	0.006895	0.008668	0.011285	0.00923	0.018144	0.018057
667	0.001738	0.00368	0.0037	0.006873	0.007471	0.006503	0.008432	0.010861	0.00887	0.017821	0.017932
666	0.00144	0.003522	0.003427	0.006744	0.007364	0.00642	0.008522	0.01087	0.00871	0.017819	0.017874
665	0.001315	0.003471	0.003299	0.006634	0.007387	0.006287	0.008239	0.010754	0.008762	0.01781	0.018105
664	0.001216	0.003417	0.003389	0.00665	0.007225	0.006361	0.008227	0.010822	0.0088	0.018051	0.018352
663	0.001061	0.003143	0.003328	0.006657	0.007186	0.006388	0.008355	0.010871	0.008774	0.018148	0.018414
662	0.001131	0.003254	0.003302	0.00648	0.007206	0.006355	0.008393	0.011034	0.008962	0.018233	0.018688
661	0.000912	0.00312	0.00329	0.006605	0.00708	0.006322	0.008319	0.011071	0.008861	0.01841	0.018898
660	0.000951	0.003054	0.003267	0.006412	0.007239	0.006436	0.008301	0.011119	0.008964	0.018665	0.019097
659	0.000869	0.003074	0.003173	0.00639	0.007069	0.006406	0.008442	0.011226	0.009176	0.018733	0.019503
658	0.000754	0.002968	0.003135	0.006472	0.007111	0.006539	0.008417	0.011346	0.009038	0.01907	0.019692
657	0.000734	0.002977	0.003194	0.006441	0.007056	0.006492	0.008548	0.011293	0.00914	0.019127	0.019789
656	0.000619	0.00288	0.003102	0.006454	0.007145	0.006485	0.008451	0.011538	0.009268	0.01937	0.020128
655	0.000668	0.002843	0.003097	0.006566	0.007018	0.00654	0.008508	0.011574	0.009241	0.019418	0.020437
654	0.00055	0.002776	0.003078	0.006393	0.007078	0.006724	0.008555	0.011604	0.009347	0.019673	0.020647
653	0.000543	0.002651	0.002929	0.0064	0.007073	0.006581	0.008666	0.011734	0.009326	0.019754	0.021029
652	0.000353	0.00275	0.003011	0.006402	0.007114	0.006552	0.00844	0.011804	0.00954	0.020067	0.021236
651	0.000342	0.002563	0.002981	0.006387	0.006953	0.006734	0.008761	0.012093	0.009397	0.020271	0.021431
650	0.000277	0.002604	0.002906	0.006223	0.007081	0.006764	0.00866	0.012158	0.009536	0.020375	0.02177
649	0.000206	0.002426	0.002953	0.006177	0.00711	0.006614	0.008657	0.01211	0.00956	0.0206	0.02212
648	0.000136	0.002445	0.002912	0.006345	0.007016	0.006794	0.00874	0.012079	0.009591	0.02081	0.022273
647	5.41E-05	0.002264	0.002829	0.006173	0.006989	0.006879	0.008691	0.012323	0.009821	0.020912	0.022532
646	-0.00017	0.002274	0.002923	0.006235	0.00708	0.006994	0.008792	0.012398	0.009825	0.021195	0.022964
645	-0.0003	0.002192	0.002818	0.006204	0.006995	0.007045	0.008746	0.012571	0.009867	0.021532	0.023136
644	-0.00018	0.002182	0.002828	0.006237	0.006949	0.007075	0.008933	0.012681	0.010068	0.021608	0.023568
643	-0.0003	0.002203	0.002826	0.006238	0.007055	0.006959	0.008886	0.012739	0.010175	0.021816	0.023773
642	-0.00035	0.00213	0.002786	0.00623	0.006983	0.007155	0.008935	0.012913	0.010112	0.02217	0.024085
641	-0.00042	0.002045	0.002712	0.006127	0.007003	0.007174	0.008922	0.012994	0.010199	0.022278	0.024461
640	-0.00054	0.002093	0.002763	0.00616	0.006898	0.007241	0.009	0.01309	0.010368	0.022492	0.024694
639	-0.00052	0.002058	0.00274	0.006093	0.007022	0.007266	0.009031	0.013243	0.010513	0.022817	0.025126

EXCLI Journal 2020;19:48-70 – ISSN 1611-2156
Received: October 03, 2019, accepted: December 19, 2019, published: January 03, 2020

Absorption intensity											
**Wavelength (nm)**	**concentration of acetylshikon (M)**										
	A	*B*	*C*	*D*	*E*	*F*	*G*	*I*	*J*	*K*	*L*
	0.00	$8.0x10^{-7}$	$1.6x10^{-6}$	$2.0x10^{-6}$	$2.8x10^{-6}$	$4.0x10^{-6}$	$4.8x10^{-6}$	$6.0x10^{-6}$	$8.0x10^{-6}$	$1.2x10^{-5}$	$1.6x10^{-5}$
638	-0.00064	0.002073	0.002765	0.006041	0.00706	0.007321	0.009077	0.013486	0.010434	0.023096	0.025338
637	-0.00077	0.002113	0.002698	0.006004	0.007	0.007294	0.009198	0.013543	0.010767	0.023308	0.02584
636	-0.00071	0.001954	0.002789	0.006016	0.007242	0.007429	0.009234	0.013673	0.010752	0.023533	0.026062
635	-0.00075	0.001931	0.002818	0.00614	0.007002	0.00762	0.009348	0.013926	0.010877	0.023732	0.026485
634	-0.00079	0.001903	0.002808	0.006091	0.00719	0.007569	0.009443	0.013961	0.011016	0.024022	0.026968
633	-0.00087	0.001861	0.002768	0.006114	0.007287	0.007605	0.009376	0.014103	0.011151	0.024199	0.027182
632	-0.00086	0.001758	0.002804	0.006055	0.007148	0.007915	0.009543	0.014308	0.011256	0.024505	0.027531
631	-0.00093	0.001772	0.002786	0.006259	0.007246	0.007822	0.009581	0.014527	0.011489	0.024853	0.028083
630	-0.00096	0.001808	0.002898	0.006312	0.007319	0.007956	0.009659	0.014544	0.011515	0.024964	0.028311
629	-0.00088	0.001821	0.002894	0.006371	0.007389	0.008204	0.009745	0.014867	0.011681	0.025345	0.028749
628	-0.00088	0.001912	0.003098	0.006333	0.007407	0.008356	0.009991	0.014966	0.011919	0.02572	0.029182
627	-0.0009	0.00197	0.003099	0.006416	0.007617	0.008506	0.01017	0.015361	0.012095	0.025938	0.02955
626	-0.00078	0.001908	0.003155	0.006489	0.007664	0.008534	0.01012	0.015291	0.012166	0.026277	0.029814
625	-0.00094	0.001982	0.00312	0.006676	0.007682	0.008746	0.010221	0.015556	0.012424	0.026674	0.030453
624	-0.00094	0.00202	0.003066	0.006535	0.007817	0.008744	0.010418	0.015959	0.012622	0.026865	0.030817
623	-0.00097	0.001986	0.003307	0.006678	0.007967	0.008906	0.010502	0.016015	0.01268	0.0273	0.031126
622	-0.00091	0.002124	0.003309	0.006869	0.00798	0.008993	0.010623	0.016202	0.012997	0.027658	0.03159
621	-0.00078	0.002198	0.003469	0.006803	0.008162	0.009234	0.010777	0.016457	0.013126	0.027818	0.032041
620	-0.00071	0.002255	0.003362	0.006868	0.008283	0.009454	0.010878	0.016679	0.013375	0.028186	0.032497
619	-0.00074	0.002248	0.003559	0.006922	0.008321	0.009566	0.010941	0.016846	0.01355	0.028565	0.032852
618	-0.00068	0.002281	0.00368	0.007074	0.008398	0.009668	0.011147	0.017037	0.013783	0.028845	0.03333
617	-0.00058	0.002362	0.003655	0.007237	0.008435	0.009839	0.011336	0.017269	0.013905	0.029078	0.03365
616	-0.00067	0.002315	0.003774	0.007199	0.008652	0.009982	0.011502	0.01748	0.014149	0.029368	0.034254
615	-0.00058	0.002434	0.00384	0.007253	0.008695	0.010108	0.011651	0.017704	0.014353	0.029886	0.034529
614	-0.00054	0.002417	0.004036	0.007415	0.008774	0.010254	0.011666	0.01783	0.01447	0.029969	0.035093
613	-0.00045	0.002491	0.004022	0.0075	0.008934	0.010463	0.011746	0.01811	0.014642	0.030445	0.035447
612	-0.00041	0.002643	0.004123	0.007494	0.009031	0.010593	0.012038	0.018226	0.014896	0.030635	0.035749
611	-0.00045	0.002664	0.003939	0.00769	0.009091	0.010621	0.01199	0.018462	0.01501	0.030875	0.036105
610	-0.00048	0.002597	0.004286	0.007667	0.009093	0.01088	0.012196	0.018627	0.015191	0.03123	0.036556
609	-0.00058	0.002667	0.004263	0.007757	0.00909	0.010812	0.012295	0.018762	0.015437	0.031344	0.036767
608	-0.00052	0.002633	0.00423	0.007817	0.009329	0.010964	0.012306	0.019065	0.015457	0.031764	0.037186
607	-0.00049	0.002586	0.004339	0.0078	0.00929	0.011104	0.012184	0.019075	0.015492	0.031855	0.03754
606	-0.0006	0.002715	0.004279	0.007776	0.009445	0.011061	0.012434	0.019402	0.015648	0.032255	0.037882

EXCLI Journal 2020;19:48-70 – ISSN 1611-2156
Received: October 03, 2019, accepted: December 19, 2019, published: January 03, 2020

Absorption intensity											
**Wavelength (nm)**	**concentration of acetylshikon (M)**										
	A	*B*	*C*	*D*	*E*	*F*	*G*	*I*	*J*	*K*	*L*
	0.00	8.0×10^{-7}	1.6×10^{-6}	2.0×10^{-6}	2.8×10^{-6}	4.0×10^{-6}	4.8×10^{-6}	6.0×10^{-6}	8.0×10^{-6}	1.2×10^{-5}	1.6×10^{-5}
605	-0.00056	0.002599	0.004173	0.007673	0.009459	0.011186	0.012517	0.019437	0.015746	0.0323	0.038203
604	-0.00058	0.00265	0.004298	0.007749	0.009494	0.011241	0.012527	0.019562	0.015881	0.0326	0.038517
603	-0.00085	0.002351	0.004188	0.007813	0.009329	0.011266	0.012453	0.01968	0.015904	0.032845	0.038918
602	-0.00092	0.002442	0.004148	0.007746	0.009369	0.011286	0.012447	0.019734	0.015836	0.032912	0.039114
601	-0.001	0.002104	0.004004	0.00754	0.009207	0.01132	0.012477	0.019705	0.01583	0.033084	0.039335
600	-0.00119	0.002091	0.003888	0.00745	0.009232	0.011161	0.012463	0.019747	0.015957	0.033207	0.039448
599	-0.00132	0.001868	0.003764	0.007405	0.009	0.011143	0.01227	0.01973	0.015941	0.033358	0.039723
598	-0.00142	0.00172	0.003567	0.007228	0.008949	0.011063	0.012354	0.01991	0.015966	0.033488	0.040007
597	-0.00167	0.001679	0.00348	0.007063	0.008887	0.010922	0.012239	0.019919	0.015992	0.033551	0.040226
596	-0.00185	0.001378	0.003355	0.00707	0.008818	0.010879	0.012072	0.019781	0.015821	0.033553	0.040298
595	-0.00196	0.001373	0.00329	0.006879	0.008735	0.010869	0.01221	0.019706	0.015777	0.033707	0.04065
594	-0.00227	0.001086	0.003082	0.006749	0.008522	0.010894	0.012012	0.01996	0.015852	0.033691	0.040743
593	-0.00232	0.001075	0.003092	0.006658	0.008533	0.010735	0.011931	0.019777	0.016018	0.033856	0.040895
592	-0.00246	0.000948	0.003029	0.00651	0.008488	0.010721	0.011887	0.019853	0.016041	0.033914	0.040876
591	-0.0026	0.000897	0.002913	0.006599	0.008307	0.010838	0.012001	0.019986	0.015986	0.033871	0.041208
590	-0.00268	0.000714	0.002927	0.006367	0.008352	0.010636	0.011759	0.019654	0.015996	0.033962	0.041466
589	-0.00277	0.000769	0.002867	0.006504	0.008347	0.01082	0.011753	0.019757	0.015923	0.0343	0.041734
588	-0.00282	0.000775	0.00269	0.006272	0.008143	0.01065	0.011785	0.020031	0.015983	0.034609	0.042012
587	-0.00276	0.000604	0.00296	0.006489	0.00841	0.010803	0.012043	0.020307	0.016364	0.035125	0.042683
586	-0.00288	0.00075	0.002965	0.006667	0.008541	0.011077	0.012092	0.020544	0.01649	0.03516	0.043031
585	-0.00285	0.000599	0.002897	0.006597	0.00859	0.011165	0.012284	0.020735	0.016845	0.035616	0.043605
584	-0.00293	0.000776	0.00296	0.006686	0.008656	0.011241	0.012274	0.020877	0.016877	0.035878	0.043957
583	-0.00266	0.000732	0.003083	0.006721	0.008754	0.01135	0.01253	0.020982	0.017008	0.036168	0.04424
582	-0.00281	0.000801	0.003042	0.006908	0.008825	0.011508	0.012583	0.021189	0.017255	0.0364	0.04455
581	-0.00285	0.000811	0.003072	0.006794	0.008872	0.011715	0.012648	0.021502	0.017289	0.036593	0.045112
580	-0.00276	0.000872	0.003218	0.006971	0.009097	0.011691	0.012752	0.021735	0.017527	0.03703	0.045461
579	-0.00271	0.000999	0.003218	0.007045	0.009058	0.011973	0.013082	0.021872	0.017852	0.03738	0.045806
578	-0.0027	0.001081	0.003303	0.007155	0.009189	0.012025	0.013078	0.022095	0.017936	0.037443	0.046202
577	-0.00273	0.00102	0.003392	0.007195	0.009319	0.012149	0.013238	0.022328	0.018293	0.03781	0.046583
576	-0.00265	0.001152	0.003543	0.007402	0.009494	0.012262	0.013319	0.022351	0.018397	0.03817	0.047041
575	-0.0027	0.001241	0.003374	0.007402	0.009496	0.012294	0.013508	0.022563	0.018499	0.038365	0.047349
574	-0.00268	0.001271	0.003789	0.007351	0.009592	0.012496	0.013532	0.022774	0.018632	0.038687	0.047533
573	-0.00265	0.001205	0.003638	0.007466	0.00953	0.012636	0.013539	0.022824	0.018795	0.038706	0.047893

EXCLI Journal 2020;19:48-70 – ISSN 1611-2156
Received: October 03, 2019, accepted: December 19, 2019, published: January 03, 2020

Absorption intensity											
**Wavelength (nm)**	**concentration of acetylshikon (M)**										
	A	*B*	*C*	*D*	*E*	*F*	*G*	*I*	*J*	*K*	*L*
	0.00	$8.0x10^{-7}$	$1.6x10^{-6}$	$2.0x10^{-6}$	$2.8x10^{-6}$	$4.0x10^{-6}$	$4.8x10^{-6}$	$6.0x10^{-6}$	$8.0x10^{-6}$	$1.2x10^{-5}$	$1.6x10^{-5}$
572	-0.00266	0.001379	0.003612	0.007496	0.009699	0.012564	0.013714	0.023107	0.018999	0.039307	0.048481
571	-0.00267	0.00128	0.003797	0.007646	0.009884	0.012716	0.013679	0.023138	0.019221	0.039453	0.048555
570	-0.00259	0.00136	0.003865	0.007562	0.009775	0.012847	0.0139	0.023373	0.019354	0.03962	0.048938
569	-0.00271	0.001353	0.003778	0.007781	0.009755	0.01282	0.013803	0.023433	0.019438	0.039712	0.049105
568	-0.00244	0.00136	0.003993	0.007969	0.01006	0.01304	0.013956	0.023694	0.019761	0.040045	0.049609
567	-0.00196	0.001892	0.004379	0.008301	0.010391	0.013603	0.014658	0.024236	0.020187	0.04082	0.050311
566	-0.00204	0.002018	0.004604	0.008463	0.010629	0.01374	0.014745	0.024384	0.020369	0.040916	0.050485
565	-0.00197	0.001938	0.004459	0.008428	0.010706	0.013754	0.014753	0.024583	0.020515	0.041171	0.050854
564	-0.00191	0.00199	0.004606	0.008399	0.010644	0.013918	0.014861	0.024702	0.020639	0.041398	0.051118
563	-0.00203	0.002033	0.004504	0.008535	0.010772	0.013805	0.014937	0.024768	0.020872	0.041586	0.051332
562	-0.00197	0.002159	0.004792	0.008732	0.010898	0.014041	0.015048	0.024812	0.021034	0.041876	0.051356
561	-0.00202	0.002085	0.004557	0.008596	0.010789	0.01406	0.01509	0.024931	0.021054	0.041912	0.051922
560	-0.00206	0.002173	0.004703	0.008633	0.010797	0.01411	0.015159	0.025137	0.02099	0.042263	0.052051
559	-0.0019	0.002075	0.004655	0.008681	0.010897	0.014122	0.015018	0.025142	0.021217	0.042236	0.05231
558	-0.00196	0.002153	0.004741	0.008798	0.010933	0.014203	0.015172	0.025359	0.021275	0.042336	0.052464
557	-0.00206	0.002089	0.004729	0.008608	0.010991	0.014404	0.015187	0.02536	0.021412	0.042504	0.052729
556	-0.00213	0.002121	0.004644	0.008735	0.010982	0.014112	0.015159	0.025477	0.021517	0.042589	0.053035
555	-0.00198	0.002018	0.004724	0.008645	0.011043	0.014383	0.015222	0.025438	0.021518	0.042828	0.053126
554	-0.00224	0.002026	0.004626	0.008766	0.011006	0.014301	0.015117	0.025469	0.021562	0.043017	0.053299
553	-0.00225	0.002008	0.004549	0.008659	0.011105	0.01429	0.015203	0.025657	0.021667	0.042998	0.05358
552	-0.00218	0.001912	0.004647	0.008653	0.011034	0.014458	0.015253	0.025657	0.021764	0.043244	0.053713
551	-0.00224	0.001811	0.004557	0.008682	0.011068	0.014332	0.015288	0.025715	0.02172	0.043297	0.053921
550	-0.00237	0.001892	0.004602	0.008658	0.011042	0.014285	0.015198	0.025812	0.021915	0.043562	0.054123
549	-0.00234	0.001887	0.004465	0.008729	0.010871	0.014368	0.015257	0.025797	0.021965	0.043724	0.054273
548	-0.00248	0.001746	0.004446	0.008652	0.011045	0.014335	0.015308	0.025995	0.021961	0.043637	0.054428
547	-0.00257	0.00179	0.004477	0.00865	0.011045	0.014329	0.015238	0.025917	0.022016	0.043905	0.05472
546	-0.00254	0.001722	0.004462	0.008573	0.010986	0.014441	0.015247	0.026034	0.021987	0.043866	0.054859
545	-0.00264	0.00167	0.004321	0.008455	0.011003	0.014442	0.015323	0.026	0.022164	0.044109	0.05489
544	-0.00283	0.001534	0.004449	0.008598	0.010879	0.014249	0.015243	0.026136	0.022152	0.04413	0.055233
543	-0.00283	0.001554	0.004361	0.008352	0.010863	0.014249	0.015337	0.026145	0.022129	0.044274	0.055311
542	-0.003	0.001365	0.004292	0.008374	0.010851	0.014256	0.015236	0.026172	0.022197	0.044394	0.055457
541	-0.00309	0.001395	0.004134	0.008324	0.010755	0.014274	0.015166	0.026225	0.022223	0.04437	0.055708
540	-0.00308	0.001247	0.004053	0.008377	0.010762	0.014297	0.015098	0.026166	0.022236	0.044676	0.055893

Absorption intensity											
**Wavelength (nm)**	**concentration of acetylshikon (M)**										
	A	*B*	*C*	*D*	*E*	*F*	*G*	*I*	*J*	*K*	*L*
	0.00	$8.0x10^{-7}$	$1.6x10^{-6}$	$2.0x10^{-6}$	$2.8x10^{-6}$	$4.0x10^{-6}$	$4.8x10^{-6}$	$6.0x10^{-6}$	$8.0x10^{-6}$	$1.2x10^{-5}$	$1.6x10^{-5}$
539	-0.00322	0.001244	0.003956	0.008245	0.010654	0.014264	0.015212	0.026272	0.022274	0.044786	0.056031
538	-0.00332	0.001143	0.00397	0.008344	0.010658	0.014128	0.015204	0.026225	0.022395	0.044894	0.056199
537	-0.0035	0.000962	0.00392	0.008243	0.010619	0.014198	0.015146	0.026331	0.022365	0.044964	0.056494
536	-0.0035	0.000884	0.003696	0.008098	0.010558	0.014235	0.015106	0.026413	0.022473	0.045157	0.056616
535	-0.00369	0.000959	0.003639	0.008021	0.010434	0.014177	0.014952	0.026351	0.022554	0.045191	0.056795
534	-0.00371	0.00081	0.00369	0.00794	0.010651	0.014183	0.015153	0.026507	0.022413	0.045326	0.05701
533	-0.00368	0.000623	0.003596	0.008014	0.010509	0.01419	0.01518	0.026369	0.022499	0.045306	0.057116
532	-0.00395	0.000656	0.003441	0.007984	0.010434	0.014072	0.01514	0.026614	0.022641	0.04541	0.057301
531	-0.004	0.000505	0.003565	0.007927	0.010547	0.014089	0.015037	0.026616	0.02258	0.045659	0.057536
530	-0.00417	0.000396	0.003502	0.00797	0.010376	0.014061	0.015045	0.0265	0.022734	0.045753	0.057489
529	-0.0041	0.000527	0.003355	0.007894	0.010276	0.013946	0.015142	0.026613	0.022787	0.045809	0.057717
528	-0.00418	0.000532	0.003353	0.007796	0.010409	0.014017	0.014903	0.026587	0.022849	0.045951	0.058005
527	-0.00423	0.00036	0.003327	0.007799	0.010381	0.014024	0.014992	0.026766	0.022945	0.046115	0.058103
526	-0.00419	0.000379	0.003382	0.00787	0.01042	0.014057	0.015063	0.026769	0.023046	0.046171	0.058353
525	-0.00432	0.000366	0.003311	0.00778	0.010537	0.013937	0.014883	0.026761	0.022859	0.04622	0.058328
524	-0.00429	0.000181	0.003342	0.007765	0.010333	0.014014	0.015067	0.026838	0.023099	0.046406	0.058703
523	-0.00451	0.000302	0.00333	0.007786	0.010393	0.014006	0.015043	0.026909	0.023187	0.046611	0.058818
522	-0.0044	0.00032	0.003239	0.007835	0.010509	0.01407	0.015139	0.027088	0.023273	0.046633	0.059081
521	-0.00441	0.000178	0.003332	0.007772	0.010442	0.014241	0.015124	0.027034	0.023233	0.046725	0.058958
520	-0.00431	0.000277	0.003238	0.007992	0.010475	0.014158	0.015239	0.027073	0.023497	0.046866	0.059351
519	-0.00442	0.000238	0.003197	0.007937	0.010516	0.014114	0.015176	0.027172	0.023391	0.047051	0.059457
518	-0.00459	0.000306	0.003329	0.007923	0.010653	0.014198	0.015274	0.027367	0.023546	0.047199	0.059561
517	-0.00458	0.000267	0.003218	0.007952	0.010691	0.014318	0.015258	0.027288	0.023713	0.047234	0.059867
516	-0.00448	0.00021	0.003332	0.008008	0.010731	0.014256	0.015311	0.027424	0.023723	0.047433	0.059975
515	-0.0046	0.000245	0.003438	0.008098	0.010721	0.01421	0.01543	0.027531	0.023775	0.047528	0.060173
514	-0.00461	0.000322	0.003228	0.007932	0.010576	0.014437	0.01531	0.027456	0.023928	0.047656	0.06029
513	-0.00456	0.000349	0.003312	0.008081	0.010841	0.014338	0.015531	0.027679	0.024048	0.047856	0.06054
512	-0.00476	0.000291	0.003411	0.008122	0.010774	0.014439	0.015502	0.027709	0.024016	0.047819	0.060518
511	-0.00455	0.00037	0.003303	0.008159	0.010795	0.014361	0.015515	0.027618	0.024403	0.048022	0.060878
510	-0.00458	0.000336	0.003279	0.008122	0.010869	0.014425	0.015471	0.027735	0.024445	0.048065	0.060991
509	-0.00461	0.000441	0.00359	0.008284	0.011003	0.014439	0.015808	0.027986	0.024432	0.048268	0.061032
508	-0.00455	0.000347	0.003504	0.008229	0.011012	0.0146	0.01576	0.027989	0.024549	0.048403	0.061415
507	-0.00463	0.000417	0.003449	0.008205	0.010977	0.014681	0.01561	0.028002	0.024605	0.048602	0.06148

EXCLI Journal 2020;19:48-70 – ISSN 1611-2156
Received: October 03, 2019, accepted: December 19, 2019, published: January 03, 2020

Absorption intensity											
**Wavelength (nm)**	**concentration of acetylshikon (M)**										
	A	*B*	*C*	*D*	*E*	*F*	*G*	*I*	*J*	*K*	*L*
	0.00	$8.0x10^{-7}$	$1.6x10^{-6}$	$2.0x10^{-6}$	$2.8x10^{-6}$	$4.0x10^{-6}$	$4.8x10^{-6}$	$6.0x10^{-6}$	$8.0x10^{-6}$	$1.2x10^{-5}$	$1.6x10^{-5}$
506	-0.00469	0.000452	0.003382	0.008409	0.011048	0.014477	0.015942	0.028259	0.024742	0.048675	0.061514
505	-0.00452	0.000434	0.003445	0.008316	0.011105	0.014795	0.015972	0.028112	0.024776	0.048665	0.061849
504	-0.00448	0.000547	0.003569	0.008378	0.011152	0.01478	0.016044	0.028474	0.024918	0.048849	0.061909
503	-0.00458	0.000486	0.003541	0.008415	0.011238	0.014619	0.015966	0.028307	0.024876	0.048997	0.061884
502	-0.00461	0.00053	0.003502	0.008468	0.01123	0.014761	0.016049	0.028559	0.025124	0.049057	0.06214
501	-0.00463	0.000581	0.003645	0.008432	0.011258	0.014777	0.016022	0.02851	0.025131	0.049209	0.06238
500	-0.00463	0.000536	0.00362	0.008711	0.011313	0.014789	0.016167	0.028579	0.02519	0.049294	0.062395
499	-0.0046	0.000414	0.003628	0.008637	0.01132	0.014929	0.016297	0.028543	0.025196	0.049233	0.062511
498	-0.0047	0.000525	0.0036	0.008552	0.011126	0.0149	0.016206	0.028697	0.025316	0.049479	0.062797
497	-0.00474	0.000506	0.003607	0.008641	0.011413	0.014911	0.016206	0.028722	0.025546	0.04946	0.062824
496	-0.00449	0.000615	0.003559	0.008746	0.011499	0.015015	0.016214	0.028623	0.025456	0.04959	0.062944
495	-0.00473	0.000561	0.003614	0.008693	0.011436	0.014886	0.016309	0.028828	0.025622	0.049704	0.062985
494	-0.00463	0.000491	0.003555	0.008735	0.011511	0.01501	0.016294	0.0288	0.025671	0.049859	0.063123
493	-0.00465	0.000582	0.003667	0.008896	0.01157	0.014945	0.016406	0.02874	0.025723	0.050045	0.063115
492	-0.0046	0.00052	0.00364	0.008813	0.011435	0.015033	0.016329	0.028937	0.025896	0.049971	0.06333
491	-0.00467	0.000601	0.003571	0.008802	0.011502	0.014918	0.016479	0.028924	0.025749	0.049967	0.063353
490	-0.00463	0.000498	0.003601	0.008701	0.011576	0.015068	0.01633	0.028922	0.025909	0.050047	0.063285
489	-0.00458	0.000561	0.003465	0.008844	0.011449	0.014952	0.01643	0.028909	0.025814	0.049949	0.063443
488	-0.00469	0.000682	0.003563	0.0088	0.011657	0.014961	0.016446	0.029033	0.025972	0.050072	0.063466
487	-0.0047	0.000482	0.003462	0.008822	0.011628	0.014962	0.016333	0.028853	0.025782	0.049839	0.063321
486	-0.00481	0.000658	0.003634	0.008882	0.011438	0.014938	0.016453	0.028929	0.026105	0.050043	0.063456
485	-0.00489	0.000566	0.003439	0.008704	0.011456	0.014803	0.016126	0.028733	0.02582	0.049936	0.063266
484	-0.00482	0.000443	0.003358	0.008815	0.011458	0.014665	0.016379	0.028708	0.025969	0.049966	0.063338
483	-0.0049	0.000273	0.003275	0.008677	0.011407	0.014744	0.016048	0.02867	0.025874	0.049916	0.063252
482	-0.00498	0.000424	0.003152	0.008758	0.011348	0.014664	0.016145	0.028657	0.025881	0.049861	0.063188
481	-0.00508	0.000272	0.003229	0.00862	0.011367	0.014458	0.015986	0.028486	0.025807	0.049757	0.062988
480	-0.00513	0.000328	0.003179	0.008656	0.011185	0.014327	0.015929	0.028519	0.025715	0.04963	0.062917
479	-0.00532	0.000168	0.00302	0.008453	0.011215	0.014229	0.01584	0.028282	0.025666	0.049387	0.062843
478	-0.00543	-0.0001	0.002826	0.008326	0.01111	0.014041	0.015741	0.027988	0.025495	0.049321	0.062537
477	-0.0057	-0.00023	0.00275	0.008249	0.010821	0.013921	0.01563	0.027909	0.025644	0.049242	0.0623
476	-0.00556	-0.00014	0.002708	0.008137	0.010862	0.013811	0.015487	0.027836	0.025482	0.049123	0.062208
475	-0.00581	-0.00039	0.002474	0.008056	0.010706	0.013505	0.015249	0.027554	0.025249	0.0487	0.062057
474	-0.00592	-0.00053	0.00224	0.008019	0.010492	0.01352	0.015219	0.027626	0.025284	0.048663	0.061815

Absorption intensity											
**Wavelength (nm)**	**concentration of acetylshikon (M)**										
	A	*B*	*C*	*D*	*E*	*F*	*G*	*I*	*J*	*K*	*L*
	0.00	$8.0x10^{-7}$	$1.6x10^{-6}$	$2.0x10^{-6}$	$2.8x10^{-6}$	$4.0x10^{-6}$	$4.8x10^{-6}$	$6.0x10^{-6}$	$8.0x10^{-6}$	$1.2x10^{-5}$	$1.6x10^{-5}$
473	-0.00616	-0.00064	0.002148	0.007896	0.010525	0.0133	0.015193	0.027332	0.025047	0.048608	0.06162
472	-0.00615	-0.00086	0.001945	0.007818	0.010267	0.013028	0.014946	0.027076	0.024966	0.04831	0.061482
471	-0.00626	-0.0009	0.00198	0.007814	0.010124	0.01299	0.014797	0.027071	0.024764	0.048066	0.06114
470	-0.00652	-0.00077	0.001867	0.007541	0.010095	0.013027	0.014639	0.027079	0.024862	0.048239	0.061206
469	-0.00624	-0.00119	0.001744	0.00753	0.010066	0.012927	0.014661	0.026685	0.024779	0.047996	0.060962
468	-0.00633	-0.00106	0.001761	0.007605	0.010026	0.012835	0.014601	0.026554	0.02495	0.047781	0.060453
467	-0.00645	-0.00103	0.001561	0.007392	0.010028	0.012713	0.014438	0.026713	0.024704	0.047633	0.060141
466	-0.00637	-0.00103	0.001702	0.007619	0.009918	0.012805	0.014542	0.026476	0.024665	0.047177	0.060176
465	-0.00644	-0.00113	0.001531	0.007487	0.00998	0.012625	0.014423	0.026403	0.024746	0.047077	0.059924
464	-0.00648	-0.00105	0.001598	0.007481	0.009765	0.012638	0.014479	0.026317	0.02483	0.047059	0.059847
463	-0.00643	-0.00098	0.001496	0.007613	0.009882	0.012504	0.014393	0.026142	0.024729	0.046881	0.059679
462	-0.00647	-0.001	0.001533	0.007529	0.010022	0.012586	0.0144	0.02574	0.024444	0.046746	0.05936
461	-0.00654	-0.00105	0.001542	0.007464	0.009893	0.012469	0.014326	0.025645	0.024396	0.046723	0.05926
460	-0.00659	-0.00104	0.001509	0.007503	0.009946	0.012103	0.01391	0.025708	0.024322	0.046579	0.059158
459	-0.00636	-0.00101	0.001626	0.007351	0.009537	0.011802	0.014018	0.02563	0.024458	0.046481	0.05913
458	-0.00641	-0.00087	0.001418	0.007215	0.009662	0.012026	0.014133	0.025562	0.02455	0.046566	0.058866
457	-0.00628	-0.00097	0.00136	0.00732	0.009816	0.011994	0.014241	0.025649	0.024597	0.04667	0.05905
456	-0.00614	-0.00096	0.001661	0.007768	0.009994	0.012248	0.014403	0.025771	0.024812	0.04644	0.05896
455	-0.00641	-0.001	0.001478	0.007561	0.009611	0.012126	0.014267	0.025536	0.02488	0.046493	0.0587
454	-0.00647	-0.00095	0.001468	0.007557	0.009904	0.011885	0.014074	0.025575	0.024832	0.046297	0.0586
453	-0.00647	-0.00098	0.001343	0.007632	0.009797	0.01201	0.014136	0.02544	0.024779	0.046139	0.058337
452	-0.0066	-0.00102	0.001279	0.007656	0.009861	0.011964	0.01423	0.025391	0.024821	0.046157	0.058211
451	-0.00655	-0.00092	0.001429	0.007663	0.009822	0.01163	0.014073	0.025135	0.024685	0.045889	0.057966
450	-0.00655	-0.00091	0.001332	0.007711	0.009881	0.01186	0.014163	0.025136	0.024822	0.04577	0.057833
449	-0.00663	-0.00113	0.001157	0.007644	0.009687	0.011707	0.013856	0.025087	0.024662	0.045602	0.057556
448	-0.00649	-0.00095	0.001232	0.007733	0.00967	0.011608	0.013915	0.024892	0.02476	0.045619	0.057344
447	-0.00661	-0.00109	0.00132	0.00755	0.009552	0.011534	0.01374	0.024752	0.024706	0.04528	0.057241
446	-0.00656	-0.00107	0.001006	0.007728	0.009641	0.011388	0.013845	0.024796	0.024661	0.045341	0.057129
445	-0.00671	-0.00109	0.001104	0.007419	0.009448	0.011353	0.013778	0.024565	0.024638	0.045054	0.05669
444	-0.00673	-0.00106	0.001124	0.00774	0.009623	0.011266	0.013642	0.024545	0.024685	0.044946	0.056546
443	-0.00659	-0.00116	0.000939	0.007486	0.009467	0.011217	0.013613	0.024462	0.024612	0.044834	0.056283
442	-0.00669	-0.00091	0.00097	0.007453	0.009393	0.011203	0.013739	0.024215	0.024629	0.044723	0.056149
441	-0.00679	-0.00117	0.000885	0.007645	0.009439	0.010997	0.013419	0.024093	0.02456	0.044539	0.055888

EXCLI Journal 2020;19:48-70 – ISSN 1611-2156
Received: October 03, 2019, accepted: December 19, 2019, published: January 03, 2020

Absorption intensity											
**Wavelength (nm)**	**concentration of acetylshikon (M)**										
	A	*B*	*C*	*D*	*E*	*F*	*G*	*I*	*J*	*K*	*L*
	0.00	8.0×10^{-7}	1.6×10^{-6}	2.0×10^{-6}	2.8×10^{-6}	4.0×10^{-6}	4.8×10^{-6}	6.0×10^{-6}	8.0×10^{-6}	1.2×10^{-5}	1.6×10^{-5}
440	-0.00675	-0.00112	0.00102	0.007429	0.00956	0.011085	0.013685	0.02413	0.024503	0.044418	0.055856
439	-0.00657	-0.00104	0.000957	0.007508	0.009438	0.01095	0.013597	0.02408	0.024811	0.044263	0.055732
438	-0.00656	-0.00097	0.000972	0.00787	0.009582	0.011089	0.013578	0.024074	0.024983	0.04432	0.055748
437	-0.00641	-0.0009	0.001167	0.007789	0.009531	0.010987	0.01362	0.024041	0.024819	0.044296	0.055497
436	-0.00663	-0.00106	0.000719	0.007515	0.009282	0.010771	0.013411	0.023708	0.024859	0.043921	0.055234
435	-0.00681	-0.00127	0.000581	0.007601	0.009285	0.010599	0.013107	0.023451	0.024784	0.043582	0.054737
434	-0.00688	-0.00141	0.000623	0.007484	0.009246	0.010508	0.013153	0.023204	0.024456	0.043531	0.054513
433	-0.00687	-0.00129	0.000358	0.00725	0.00903	0.0104	0.013161	0.02323	0.02446	0.043463	0.054356
432	-0.00703	-0.00131	0.000424	0.007352	0.009022	0.010219	0.01308	0.022986	0.024521	0.043216	0.054144
431	-0.00692	-0.00154	0.000354	0.007506	0.009005	0.01026	0.012959	0.023043	0.024453	0.043091	0.054138
430	-0.00708	-0.00144	0.000345	0.007283	0.008802	0.010005	0.012915	0.022768	0.024393	0.042879	0.053862
429	-0.00724	-0.00154	7.77E-05	0.007419	0.008812	0.009953	0.012757	0.022727	0.024431	0.042836	0.053551
428	-0.00706	-0.00156	0.00017	0.007367	0.008911	0.00992	0.012803	0.022528	0.024572	0.04258	0.053458
427	-0.00722	-0.00165	-5.31E-05	0.007205	0.008716	0.009739	0.012646	0.022558	0.02432	0.042681	0.05328
426	-0.00723	-0.00167	-9.44E-05	0.007171	0.008743	0.009643	0.012593	0.022345	0.024417	0.042495	0.053147
425	-0.00725	-0.00171	2.74E-05	0.007188	0.008591	0.009518	0.012543	0.022166	0.024214	0.042216	0.052656
424	-0.00741	-0.00184	-0.00021	0.007167	0.008603	0.009578	0.012688	0.021921	0.024494	0.042176	0.052737
423	-0.00755	-0.00176	-0.00014	0.007006	0.008552	0.009296	0.012324	0.02198	0.02443	0.04202	0.052668
422	-0.00761	-0.00182	-0.00033	0.007022	0.008434	0.009249	0.01225	0.02166	0.024427	0.041873	0.052412
421	-0.00743	-0.00203	-0.00037	0.007018	0.008382	0.009168	0.012297	0.021764	0.024338	0.041788	0.052183
420	-0.00767	-0.00181	-0.00031	0.006933	0.008351	0.009121	0.012097	0.02172	0.024383	0.041667	0.051985
419	-0.00773	-0.00209	-0.00038	0.00689	0.008289	0.008975	0.012118	0.021636	0.024324	0.041646	0.052077
418	-0.00765	-0.00205	-0.00059	0.00702	0.008421	0.008942	0.012118	0.021579	0.024656	0.041763	0.05169
417	-0.00776	-0.00207	-0.00038	0.006616	0.008129	0.008943	0.011747	0.020979	0.024263	0.041225	0.05171
416	-0.00753	-0.00231	-0.00072	0.006702	0.008001	0.008599	0.011726	0.021005	0.024036	0.041033	0.051292
415	-0.00804	-0.00224	-0.0008	0.006657	0.007777	0.008513	0.011734	0.020923	0.024236	0.040982	0.050983
414	-0.00814	-0.00224	-0.00099	0.006588	0.007975	0.00854	0.01166	0.020877	0.024273	0.040773	0.051127
413	-0.00821	-0.00233	-0.00076	0.006626	0.007776	0.008313	0.011477	0.020751	0.024437	0.040896	0.05076
412	-0.00821	-0.00241	-0.0007	0.006863	0.00803	0.008492	0.011603	0.020728	0.024342	0.040834	0.050648
411	-0.0083	-0.00245	-0.00101	0.006751	0.007889	0.008269	0.01162	0.020703	0.024372	0.040663	0.050881
410	-0.00823	-0.00235	-0.00105	0.006873	0.007696	0.008187	0.011544	0.020504	0.024494	0.040694	0.050608
409	-0.00805	-0.00248	-0.00119	0.006607	0.007846	0.008226	0.011516	0.02044	0.024635	0.040688	0.050503
408	-0.00807	-0.0022	-0.00098	0.006738	0.007987	0.008036	0.011613	0.020524	0.024663	0.040589	0.050364

Absorption intensity											
**Wavelength (nm)**	**concentration of acetylshikon (M)**										
	A	*B*	*C*	*D*	*E*	*F*	*G*	*I*	*J*	*K*	*L*
	0.00	$8.0x10^{-7}$	$1.6x10^{-6}$	$2.0x10^{-6}$	$2.8x10^{-6}$	$4.0x10^{-6}$	$4.8x10^{-6}$	$6.0x10^{-6}$	$8.0x10^{-6}$	$1.2x10^{-5}$	$1.6x10^{-5}$
407	-0.00818	-0.00248	-0.00098	0.006633	0.007898	0.008003	0.011478	0.020348	0.024697	0.040394	0.050181
406	-0.00805	-0.00226	-0.00107	0.006629	0.007916	0.00817	0.011504	0.02048	0.024999	0.040605	0.050151
405	-0.00806	-0.00231	-0.00106	0.006968	0.007727	0.008063	0.011627	0.020412	0.024736	0.040073	0.049804
404	-0.00827	-0.00247	-0.00107	0.006647	0.007793	0.007823	0.011287	0.020058	0.024775	0.039895	0.049882
403	-0.00819	-0.00262	-0.00149	0.006564	0.007658	0.00766	0.011204	0.019931	0.024607	0.039873	0.049617
402	-0.00832	-0.00252	-0.00141	0.006363	0.007313	0.007615	0.010824	0.019725	0.024665	0.039723	0.049567
401	-0.00823	-0.00245	-0.00124	0.006879	0.007689	0.007725	0.011206	0.01994	0.025087	0.039959	0.049859
400	-0.00836	-0.00265	-0.00134	0.006563	0.007462	0.007701	0.011165	0.01988	0.025294	0.040127	0.049802
399	-0.00838	-0.00269	-0.00148	0.006536	0.007541	0.007584	0.011101	0.019802	0.02518	0.040136	0.049733
398	-0.00828	-0.00269	-0.00159	0.006726	0.007555	0.007612	0.011045	0.019632	0.025401	0.039965	0.049838
397	-0.00843	-0.00261	-0.00153	0.006626	0.00738	0.007524	0.01099	0.019774	0.025629	0.040019	0.049965
396	-0.00867	-0.00257	-0.00173	0.006544	0.007332	0.007483	0.011088	0.019741	0.025495	0.040174	0.049748
395	-0.00877	-0.0028	-0.00187	0.006574	0.007294	0.007411	0.010917	0.019386	0.025757	0.03974	0.049649
394	-0.00879	-0.00284	-0.00191	0.006552	0.00728	0.007316	0.01094	0.019556	0.025848	0.039901	0.049833
393	-0.00874	-0.00261	-0.00189	0.006477	0.007265	0.007168	0.010926	0.019328	0.026006	0.039991	0.049799
392	-0.00911	-0.0028	-0.00168	0.00655	0.007297	0.007057	0.010903	0.019503	0.025949	0.040049	0.049772
391	-0.00888	-0.003	-0.00205	0.006233	0.007213	0.006934	0.01063	0.01937	0.025927	0.040018	0.049555
390	-0.00915	-0.003	-0.0021	0.006374	0.007216	0.00676	0.010912	0.019271	0.026342	0.03983	0.049841
389	-0.00911	-0.00319	-0.00207	0.006164	0.007072	0.006844	0.010657	0.019172	0.026475	0.039717	0.049873
388	-0.00877	-0.00273	-0.00184	0.006827	0.007424	0.007209	0.01104	0.019417	0.027011	0.040362	0.050176
387	-0.00942	-0.00341	-0.00244	0.005947	0.006648	0.006663	0.010676	0.01883	0.026391	0.039895	0.049698
386	-0.00949	-0.00363	-0.00256	0.005874	0.006656	0.006678	0.010251	0.019145	0.026463	0.039861	0.04989
385	-0.00949	-0.00355	-0.00255	0.006094	0.006723	0.006673	0.010624	0.018952	0.026505	0.039898	0.050142
384	-0.00966	-0.00375	-0.00266	0.005924	0.006701	0.006303	0.010228	0.019122	0.026601	0.040092	0.049875
383	-0.01008	-0.0038	-0.00286	0.006188	0.006551	0.006486	0.010037	0.018791	0.02674	0.039822	0.049718
382	-0.00978	-0.00383	-0.00281	0.005751	0.006359	0.006463	0.010073	0.018407	0.026713	0.039829	0.049917
381	-0.01	-0.00383	-0.00302	0.005657	0.00633	0.006242	0.009977	0.018774	0.027122	0.03995	0.050483
380	-0.01029	-0.00406	-0.00343	0.00567	0.00636	0.006105	0.010057	0.018981	0.027297	0.040398	0.050587
379	-0.01019	-0.00418	-0.00333	0.005522	0.006317	0.005993	0.010222	0.018623	0.027145	0.040357	0.050734
378	-0.01082	-0.0045	-0.00354	0.005611	0.006024	0.006031	0.009851	0.018523	0.027656	0.040053	0.050747
377	-0.01052	-0.00454	-0.0037	0.005391	0.006031	0.005618	0.009791	0.018339	0.027822	0.040232	0.05049
376	-0.01078	-0.00445	-0.00361	0.005527	0.005942	0.005692	0.009705	0.018545	0.027896	0.040542	0.050714
375	-0.01118	-0.00435	-0.00353	0.005454	0.006341	0.006202	0.010449	0.018874	0.028467	0.040828	0.051345

EXCLI Journal 2020;19:48-70 – ISSN 1611-2156
Received: October 03, 2019, accepted: December 19, 2019, published: January 03, 2020

Absorption intensity											
**Wavelength (nm)**	**concentration of acetylshikon (M)**										
	A	*B*	*C*	*D*	*E*	*F*	*G*	*I*	*J*	*K*	*L*
	0.00	8.0x10^{-7}	1.6x10^{-6}	2.0x10^{-6}	2.8x10^{-6}	4.0x10^{-6}	4.8x10^{-6}	6.0x10^{-6}	8.0x10^{-6}	1.2x10^{-5}	1.6x10^{-5}
374	-0.01067	-0.00525	-0.00455	0.005063	0.005579	0.005084	0.009073	0.018226	0.02776	0.040093	0.050715
373	-0.01154	-0.00534	-0.00437	0.004789	0.005598	0.005164	0.009481	0.017999	0.027798	0.039949	0.050545
372	-0.01162	-0.00538	-0.0044	0.004456	0.005035	0.004545	0.008813	0.017667	0.027919	0.039874	0.050171
371	-0.01205	-0.00575	-0.00475	0.004259	0.005158	0.004917	0.009107	0.018147	0.028354	0.040081	0.051128
370	-0.01212	-0.00593	-0.00489	0.004551	0.005493	0.00467	0.008632	0.017924	0.028279	0.040264	0.051063
369	-0.01274	-0.00606	-0.00471	0.004769	0.004918	0.004703	0.009024	0.017674	0.028515	0.040085	0.050846
368	-0.00663	-0.00022	0.000904	0.009935	0.010999	0.010393	0.014962	0.023487	0.034722	0.046447	0.057638
367	-0.01238	-0.00589	-0.00477	0.004371	0.005102	0.004578	0.00892	0.0181	0.028809	0.040545	0.051828
366	-0.01288	-0.0064	-0.00505	0.004162	0.005039	0.004615	0.00858	0.018062	0.029156	0.040585	0.052032
365	-0.01263	-0.00636	-0.00551	0.004536	0.004698	0.004724	0.008579	0.017565	0.028868	0.040801	0.051403
364	-0.01274	-0.00641	-0.00498	0.004187	0.004741	0.004066	0.008598	0.018233	0.029755	0.040973	0.052148
363	-0.01331	-0.00667	-0.00593	0.003866	0.004164	0.00433	0.008805	0.017685	0.029609	0.040894	0.052455
362	-0.0138	-0.007	-0.00618	0.003301	0.004061	0.003817	0.008051	0.017642	0.029755	0.040701	0.052307
361	-0.01416	-0.00693	-0.0062	0.004089	0.004242	0.004398	0.008367	0.018364	0.031047	0.041994	0.053522
360	-0.01218	-0.00589	-0.0042	0.005619	0.005677	0.005907	0.010035	0.017133	0.030017	0.041144	0.052651
359	-0.01466	-0.00856	-0.00726	0.002658	0.003396	0.00318	0.007243	0.01686	0.029079	0.040295	0.051734
358	-0.01459	-0.00825	-0.00712	0.002673	0.003227	0.003854	0.007659	0.017603	0.030337	0.040942	0.053304
357	-0.0144	-0.00823	-0.00743	0.002061	0.003383	0.003188	0.008353	0.017495	0.029648	0.041062	0.053227
356	-0.01451	-0.00856	-0.00816	0.002744	0.00293	0.002421	0.006487	0.018426	0.030391	0.042262	0.054141
355	-0.01405	-0.0076	-0.00586	0.004663	0.004339	0.005461	0.00863	0.015879	0.02927	0.040224	0.052411
354	-0.01701	-0.00979	-0.00915	0.001058	0.000987	0.002771	0.006964	0.015374	0.029932	0.040626	0.053767
353	-0.01742	-0.01019	-0.00956	0.001128	0.001157	0.002003	0.00704	0.016721	0.029742	0.041099	0.053299
352	-0.01672	-0.00995	-0.00971	0.001354	0.001562	0.002317	0.005897	0.015937	0.030479	0.040968	0.054292
351	-0.01568	-0.00803	-0.00557	0.002702	0.003676	0.005031	0.007854	0.019264	0.032702	0.044444	0.057018
350	-0.01931	-0.01314	-0.01077	0.000331	0.000208	0.000135	0.004324	0.015303	0.028874	0.04251	0.053183
349	-0.01837	-0.00955	-0.00983	0.001124	0.000719	0.002011	0.006319	0.015	0.030406	0.041912	0.053631
348	-0.00541	0.002002	0.003137	0.012944	0.014664	0.01467	0.021097	0.02919	0.044863	0.05318	0.068868
347	-0.00461	0.003397	0.003869	0.014454	0.015872	0.016107	0.02261	0.02968	0.04542	0.054076	0.070247
346	-0.00401	0.003232	0.00459	0.014204	0.016068	0.016084	0.022729	0.02976	0.046271	0.05449	0.071128
345	-0.00394	0.003495	0.004279	0.014396	0.016353	0.016851	0.023041	0.030686	0.047096	0.055291	0.072018
344	-0.00423	0.00342	0.004795	0.015156	0.016439	0.017167	0.023566	0.031348	0.047264	0.055927	0.07251
343	-0.00389	0.003663	0.004886	0.015123	0.016892	0.017249	0.023672	0.031118	0.047855	0.057463	0.074035
342	-0.00354	0.004284	0.005241	0.015463	0.017287	0.017995	0.023767	0.03192	0.048782	0.057845	0.074936

EXCLI Journal 2020;19:48-70 – ISSN 1611-2156
Received: October 03, 2019, accepted: December 19, 2019, published: January 03, 2020

Absorption intensity											
**Wavelength (nm)**	**concentration of acetylshikon (M)**										
	A	*B*	*C*	*D*	*E*	*F*	*G*	*I*	*J*	*K*	*L*
	0.00	$8.0x10^{-7}$	$1.6x10^{-6}$	$2.0x10^{-6}$	$2.8x10^{-6}$	$4.0x10^{-6}$	$4.8x10^{-6}$	$6.0x10^{-6}$	$8.0x10^{-6}$	$1.2x10^{-5}$	$1.6x10^{-5}$
341	-0.00322	0.004131	0.005857	0.015889	0.017546	0.017956	0.024482	0.032546	0.04911	0.058908	0.076032
340	-0.00336	0.004248	0.005472	0.016098	0.017861	0.018719	0.024727	0.033449	0.050899	0.059483	0.077228
339	-0.00305	0.004533	0.006296	0.016223	0.018229	0.019198	0.024942	0.034062	0.050942	0.06026	0.078237
338	-0.00339	0.004819	0.006255	0.016253	0.019161	0.019293	0.025946	0.034682	0.052274	0.061501	0.078923
337	-0.00272	0.004712	0.006193	0.016509	0.019401	0.019847	0.026002	0.035307	0.052258	0.061753	0.080204
336	-0.00301	0.005141	0.006357	0.017138	0.019394	0.019882	0.026444	0.035792	0.052857	0.062874	0.081536
335	-0.00237	0.005145	0.006931	0.017752	0.019803	0.020746	0.02712	0.036144	0.053999	0.063604	0.082697
334	-0.00243	0.00509	0.006647	0.017373	0.020105	0.021174	0.027516	0.036163	0.054487	0.064589	0.083923
333	-0.00205	0.00588	0.007375	0.017788	0.020683	0.021493	0.027849	0.037128	0.055707	0.065549	0.085035
332	-0.00249	0.006097	0.007703	0.018014	0.020278	0.021863	0.028377	0.038326	0.056443	0.066436	0.08638
331	-0.00223	0.00608	0.007867	0.018836	0.02134	0.022272	0.028872	0.038728	0.056821	0.067724	0.087778
330	-0.00165	0.005722	0.008224	0.018612	0.021607	0.022634	0.029565	0.039061	0.057685	0.068189	0.088286
329	-0.00244	0.006631	0.008709	0.019204	0.021709	0.023612	0.030008	0.039649	0.058769	0.069638	0.090344
328	-0.0018	0.006522	0.008433	0.020075	0.022421	0.023916	0.030298	0.040634	0.059531	0.070552	0.092113
327	-0.00179	0.006859	0.009384	0.019997	0.022398	0.024295	0.030269	0.041368	0.059755	0.071137	0.093114
326	-0.0018	0.006906	0.009422	0.019942	0.023181	0.025121	0.031042	0.042119	0.061656	0.072252	0.094901
325	-0.00175	0.006859	0.009779	0.0207	0.023391	0.025534	0.03161	0.043071	0.062165	0.073603	0.0963
324	-0.00212	0.007265	0.009714	0.021244	0.02389	0.026047	0.03266	0.043909	0.063317	0.075179	0.097883
323	-0.0017	0.007493	0.009987	0.021162	0.02429	0.026606	0.033043	0.044451	0.06427	0.076054	0.099433
322	-0.00143	0.007519	0.010407	0.021871	0.024802	0.027377	0.03331	0.045777	0.065437	0.077391	0.100764
321	-0.00114	0.007488	0.010953	0.021883	0.025302	0.028085	0.034011	0.046765	0.066287	0.07955	0.103266
320	-0.0017	0.007775	0.010705	0.022142	0.025782	0.028824	0.034529	0.047488	0.067561	0.080483	0.105139
319	-0.00111	0.00808	0.011838	0.022637	0.025969	0.02903	0.035036	0.048319	0.068182	0.081956	0.107079
318	-0.00118	0.00859	0.011901	0.023061	0.026213	0.029575	0.035688	0.049647	0.069981	0.083488	0.108687
317	-0.00091	0.008624	0.011843	0.023442	0.026619	0.030667	0.036283	0.050619	0.070807	0.084812	0.11151
316	-0.0009	0.008758	0.012849	0.023907	0.027529	0.031391	0.036715	0.05179	0.072302	0.086297	0.113211
315	-0.00059	0.008878	0.012542	0.024775	0.027982	0.032424	0.037852	0.052761	0.073255	0.0878	0.115585
314	-0.00053	0.009232	0.012728	0.024975	0.028755	0.032949	0.03864	0.053765	0.074693	0.089326	0.117714
313	-0.00025	0.009414	0.013866	0.025397	0.029308	0.034059	0.039144	0.055023	0.076047	0.090715	0.120095
312	-1.42E-05	0.010047	0.013675	0.026108	0.029846	0.034665	0.040237	0.055788	0.077408	0.092711	0.122274
311	2.81E-05	0.009788	0.014647	0.026572	0.030325	0.035553	0.040363	0.057039	0.078801	0.094742	0.124473
310	6.23E-05	0.010316	0.015309	0.026715	0.03075	0.036254	0.041634	0.058284	0.080259	0.096658	0.126645
309	0.000396	0.010432	0.015281	0.027158	0.031342	0.036989	0.042215	0.059663	0.081982	0.097861	0.129021

Absorption intensity											
**Wavelength (nm)**	**concentration of acetylshikon (M)**										
	A	*B*	*C*	*D*	*E*	*F*	*G*	*I*	*J*	*K*	*L*
	0.00	$8.0x10^{-7}$	$1.6x10^{-6}$	$2.0x10^{-6}$	$2.8x10^{-6}$	$4.0x10^{-6}$	$4.8x10^{-6}$	$6.0x10^{-6}$	$8.0x10^{-6}$	$1.2x10^{-5}$	$1.6x10^{-5}$
308	-0.00026	0.010331	0.015352	0.027381	0.031719	0.037218	0.042215	0.059942	0.083277	0.099534	0.131006
307	0.000633	0.011436	0.016625	0.028636	0.033088	0.038891	0.043971	0.062015	0.084893	0.101618	0.133799
306	0.000865	0.012393	0.017272	0.029661	0.033256	0.04004	0.044936	0.063611	0.086796	0.103219	0.13656
305	0.00143	0.012366	0.017417	0.029783	0.034355	0.040802	0.045826	0.064679	0.088103	0.105024	0.138737
304	0.001431	0.013195	0.018227	0.030652	0.035215	0.04207	0.046388	0.065679	0.090028	0.107255	0.141181
303	0.001875	0.013729	0.01871	0.031293	0.035496	0.042934	0.047697	0.067227	0.092404	0.109422	0.143552
302	0.002142	0.01446	0.019762	0.032578	0.036728	0.043788	0.048912	0.068991	0.094481	0.110875	0.146653
301	0.002965	0.015194	0.020322	0.033015	0.0375	0.045072	0.049908	0.070297	0.096369	0.112786	0.149308
300	0.003602	0.016062	0.02123	0.03412	0.038772	0.046534	0.051207	0.0718	0.098538	0.115019	0.151871
299	0.004064	0.016988	0.022131	0.035217	0.039772	0.047554	0.052656	0.073777	0.100959	0.117387	0.154795
298	0.005073	0.018145	0.023639	0.036556	0.04114	0.04914	0.053778	0.075234	0.10387	0.119702	0.157989
297	0.006106	0.019492	0.025034	0.037934	0.042776	0.050846	0.055616	0.077197	0.106761	0.122132	0.160587
296	0.007364	0.020701	0.026131	0.039466	0.044322	0.052756	0.057358	0.079616	0.109416	0.124702	0.164302
295	0.00889	0.022976	0.028838	0.041399	0.046438	0.055337	0.059938	0.082215	0.112997	0.128171	0.167607
294	0.011018	0.025208	0.030532	0.044058	0.048867	0.057609	0.062023	0.085413	0.117473	0.131109	0.172132
293	0.013834	0.028678	0.034079	0.047243	0.052154	0.0612	0.066022	0.088923	0.122266	0.13523	0.176227
292	0.017215	0.032159	0.037763	0.051053	0.055992	0.065075	0.070059	0.093064	0.127695	0.139834	0.181741
291	0.02088	0.036377	0.042359	0.055958	0.060845	0.070139	0.074592	0.098193	0.133288	0.145465	0.187986
290	0.025437	0.04187	0.047614	0.060923	0.065701	0.075771	0.080494	0.103906	0.140063	0.151213	0.194502
289	0.030763	0.047533	0.053329	0.066983	0.07211	0.081854	0.086886	0.110164	0.146792	0.158409	0.201577
288	0.035998	0.053503	0.058935	0.073074	0.078093	0.088364	0.093008	0.11639	0.154287	0.165772	0.209111
287	0.041077	0.059257	0.065172	0.078828	0.083636	0.094242	0.099108	0.122896	0.16105	0.172205	0.216359
286	0.045312	0.064428	0.069924	0.084107	0.088587	0.099296	0.104738	0.128231	0.167148	0.177741	0.222777
285	0.048423	0.068116	0.073806	0.087569	0.092888	0.10373	0.108571	0.132519	0.17184	0.182284	0.227537
284	0.05074	0.071088	0.07649	0.09036	0.095526	0.106443	0.111439	0.135952	0.175665	0.185634	0.231635
283	0.052935	0.073038	0.07845	0.09254	0.09748	0.108936	0.113754	0.138349	0.178834	0.188659	0.234855
282	0.054184	0.074772	0.079938	0.094421	0.099723	0.110748	0.11625	0.140583	0.181617	0.191568	0.238116
281	0.055885	0.076895	0.082348	0.09648	0.101628	0.113017	0.117969	0.142405	0.183901	0.193772	0.241467
280	0.05747	0.078307	0.084247	0.098392	0.103678	0.115095	0.120017	0.145188	0.187119	0.196312	0.244022
279	0.058841	0.079944	0.085724	0.100077	0.105225	0.116689	0.12198	0.146698	0.188607	0.198511	0.246414
278	0.059054	0.080406	0.086085	0.100207	0.105884	0.1173	0.122729	0.147686	0.190159	0.20003	0.248524
277	0.058934	0.080456	0.086029	0.100797	0.105951	0.117645	0.122593	0.148601	0.190821	0.201028	0.249677
276	0.058206	0.079839	0.085337	0.099788	0.105664	0.117275	0.122364	0.147941	0.190817	0.201239	0.250317

EXCLI Journal 2020;19:48-70 – ISSN 1611-2156
Received: October 03, 2019, accepted: December 19, 2019, published: January 03, 2020

Absorption intensity											
**Wavelength (nm)**	**concentration of acetylshikon (M)**										
	A	*B*	*C*	*D*	*E*	*F*	*G*	*I*	*J*	*K*	*L*
	0.00	$8.0x10^{-7}$	$1.6x10^{-6}$	$2.0x10^{-6}$	$2.8x10^{-6}$	$4.0x10^{-6}$	$4.8x10^{-6}$	$6.0x10^{-6}$	$8.0x10^{-6}$	$1.2x10^{-5}$	$1.6x10^{-5}$
275	0.05727	0.078754	0.084275	0.098964	0.104664	0.116197	0.121532	0.147295	0.190278	0.200905	0.250482
274	0.055834	0.077651	0.083205	0.097584	0.103553	0.11492	0.120362	0.146645	0.190031	0.20059	0.250616
273	0.05425	0.076043	0.081533	0.096378	0.102544	0.114157	0.119363	0.145853	0.189566	0.200512	0.250858
272	0.053147	0.074834	0.08047	0.095149	0.101578	0.113265	0.118026	0.145352	0.189116	0.200445	0.251399
271	0.05148	0.073684	0.079499	0.094204	0.100817	0.112489	0.117577	0.145136	0.188693	0.200338	0.252214
270	0.050713	0.072384	0.078503	0.093573	0.100127	0.112403	0.11736	0.144782	0.189013	0.201141	0.253972
269	0.049953	0.071482	0.077929	0.092713	0.099859	0.111896	0.116985	0.145371	0.189274	0.202521	0.255525
268	0.048423	0.070471	0.076668	0.091834	0.099352	0.111672	0.116459	0.14557	0.189424	0.203405	0.257677
267	0.04695	0.068527	0.075352	0.090308	0.098062	0.11075	0.115449	0.145895	0.189136	0.204565	0.259916
266	0.045348	0.067199	0.074416	0.089179	0.097138	0.110508	0.114737	0.146114	0.189652	0.205606	0.261967
265	0.04434	0.065765	0.073461	0.088318	0.096708	0.109959	0.114697	0.146555	0.189925	0.207441	0.265055
264	0.042805	0.064045	0.07214	0.087098	0.095697	0.109769	0.113587	0.14694	0.190323	0.209053	0.268108
263	0.040975	0.062479	0.070829	0.085625	0.094682	0.109143	0.113118	0.147334	0.190476	0.211084	0.271118
262	0.039979	0.061321	0.070055	0.085028	0.094351	0.109169	0.112862	0.148405	0.191211	0.213202	0.275017
261	0.039289	0.060643	0.070026	0.085143	0.094551	0.110389	0.113482	0.150279	0.193026	0.216543	0.279427
260	0.038753	0.06041	0.070248	0.085198	0.095361	0.111451	0.114538	0.152548	0.194986	0.22012	0.285101
259	0.038378	0.060076	0.069878	0.084899	0.095306	0.112239	0.114874	0.154781	0.196614	0.224168	0.290468
258	0.037272	0.058863	0.069511	0.084513	0.095435	0.112689	0.115429	0.156383	0.197537	0.227683	0.29583
257	0.035709	0.057368	0.06861	0.083216	0.094622	0.113068	0.115356	0.157971	0.198811	0.230946	0.301588
256	0.034181	0.055985	0.06799	0.08237	0.094344	0.113653	0.115354	0.16028	0.200364	0.235048	0.308071
255	0.033422	0.054965	0.068279	0.083024	0.094753	0.11527	0.116803	0.163277	0.20306	0.24068	0.315816
254	0.033	0.055273	0.069139	0.083592	0.096418	0.118065	0.118773	0.167752	0.207062	0.247691	0.325484
253	0.033156	0.055429	0.070448	0.085194	0.098687	0.121366	0.121422	0.173041	0.21156	0.25591	0.337143
252	0.032862	0.055802	0.071882	0.086125	0.100749	0.124811	0.12424	0.178927	0.216653	0.265256	0.349926
251	0.032818	0.055952	0.07339	0.087417	0.103456	0.128906	0.12768	0.185576	0.22262	0.275671	0.364235
250	0.032833	0.056853	0.075447	0.089532	0.105974	0.133804	0.131915	0.194102	0.229497	0.287483	0.380951
249	0.033905	0.058645	0.078961	0.092464	0.110738	0.140752	0.137799	0.204172	0.238813	0.302389	0.401309
248	0.03512	0.061245	0.083648	0.096584	0.116878	0.14918	0.14569	0.216919	0.250322	0.320749	0.425708
247	0.037711	0.065318	0.089416	0.102442	0.124238	0.160299	0.155058	0.232529	0.264169	0.34332	0.455994
246	0.040702	0.069876	0.097371	0.109394	0.133333	0.17371	0.166848	0.252388	0.281434	0.370618	0.492346
245	0.045282	0.076609	0.10737	0.118875	0.145614	0.191023	0.182476	0.277234	0.303307	0.405883	0.53893
244	0.051288	0.085449	0.120636	0.131249	0.161869	0.213418	0.20185	0.308943	0.330786	0.450029	0.597543
243	0.059286	0.09758	0.13841	0.147397	0.183328	0.243154	0.228432	0.351262	0.367105	0.508298	0.674822

EXCLI Journal 2020;19:48-70 – ISSN 1611-2156
Received: October 03, 2019, accepted: December 19, 2019, published: January 03, 2020

Absorption intensity											
**Wavelength (nm)**	**concentration of acetylshikon (M)**										
	A	*B*	*C*	*D*	*E*	*F*	*G*	*I*	*J*	*K*	*L*
	0.00	$8.0x10^{-7}$	$1.6x10^{-6}$	$2.0x10^{-6}$	$2.8x10^{-6}$	$4.0x10^{-6}$	$4.8x10^{-6}$	$6.0x10^{-6}$	$8.0x10^{-6}$	$1.2x10^{-5}$	$1.6x10^{-5}$
242	0.070228	0.113474	0.161013	0.168304	0.210523	0.281196	0.26244	0.405606	0.413521	0.583828	0.774755
241	0.084244	0.133644	0.191599	0.196008	0.247351	0.332765	0.307493	0.478545	0.475481	0.684925	0.907821
240	0.101401	0.159801	0.231089	0.232109	0.295216	0.400373	0.367644	0.574739	0.556237	0.818182	1.082899
239	0.123058	0.193811	0.283771	0.280003	0.359209	0.491081	0.447301	0.704461	0.664428	0.996647	1.315062
238	0.149573	0.236882	0.352245	0.341685	0.442984	0.610699	0.552361	0.875892	0.806791	1.231814	1.620629
237	0.18163	0.291361	0.441935	0.421884	0.554531	0.770381	0.692537	1.103754	0.995842	1.541667	2.01916
236	0.218755	0.359392	0.557818	0.524826	0.699561	0.979518	0.875808	1.402751	1.242687	1.945861	2.525229
235	0.261107	0.444049	0.708913	0.65778	0.889103	1.253888	1.115166	1.789391	1.564675	2.453319	3.138672
234	0.308021	0.547341	0.900879	0.826395	1.132811	1.605038	1.425132	2.279357	1.974786	3.075392	3.780293
233	0.358288	0.6755	1.14827	1.041368	1.446524	2.051705	1.818272	2.878503	2.49001	3.703269	4.219265
232	0.411037	0.831593	1.459345	1.312387	1.842614	2.602315	2.309938	3.522682	3.108318	4.090217	4.466348
231	0.465886	1.02481	1.849422	1.654248	2.330809	3.234317	2.903908	3.980885	3.698555	4.309971	4.606426
230	0.521357	1.261085	2.331411	2.076823	2.919997	3.78153	3.517099	4.210327	4.114622	4.46895	4.668761
229	0.576904	1.552979	2.901081	2.58682	3.531361	4.131958	3.977556	4.405437	4.333911	4.537117	4.930974
228	0.632766	1.910391	3.49107	3.178681	3.962839	4.296752	4.206114	4.484623	4.449704	4.645631	5.048498
227	0.688184	2.340132	3.934151	3.717875	4.184482	4.400411	4.349356	4.618156	4.618319	4.745787	5.112499
226	0.743528	2.851074	4.198541	4.086422	4.33652	4.537283	4.482097	4.793828	4.745744	4.915572	5.049989
225	0.798924	3.396345	4.332133	4.294114	4.515757	4.716468	4.592764	4.845274	4.790539	4.858156	5.306192
224	0.856403	3.873893	4.466491	4.422338	4.608018	4.723816	4.729794	4.866824	5.008596	4.99064	5.273935
223	0.917397	4.163935	4.616326	4.493466	4.76845	4.870985	4.775611	4.94064	5.051678	4.992889	5.241796
222	0.982684	4.326575	4.735853	4.612562	4.718752	5.013415	5.02642	4.95742	5.013015	5.116709	5.417457
221	1.053747	4.415432	4.699393	4.686428	4.9333	4.922418	4.980968	5.138372	5.388294	5.342932	5.196551
220	1.129431	4.49813	4.836924	4.827528	5.114919	4.954232	4.999074	5.12374	5.072833	5.171949	5.725957
219	1.20669	4.61047	4.907982	4.975663	4.949625	5.17623	5.155365	5.341466	5.311903	5.220699	5.408798
218	1.283516	4.618495	4.895218	4.889386	4.999399	4.999646	5.372398	5.226032	5.992316	5.355484	5.265071
217	1.362226	4.858595	5.025867	5.042408	5.147941	5.194232	5.017699	5.087294	5.352318	6.449275	6.14808
216	1.445438	4.80956	4.914866	5.115029	5.003448	5.10514	5.222868	5.265479	5.366182	5.281034	10
215	1.536291	4.801602	5.08072	5.272712	5.091069	5.185883	5.147839	5.499799	5.361766	5.926216	6.704196
214	1.632708	5.007155	4.922722	5.316379	5.335769	5.280863	5.532287	5.678185	5.110481	5.423494	5.5023
213	1.73808	4.961642	5.049184	5.8057	5.094603	5.74828	5.651196	5.289499	5.505223	5.350086	5.650976
212	1.848982	4.858792	5.390725	5.278561	5.444949	5.621591	5.342356	5.390689	5.922233	5.299099	5.417144
211	1.975407	5.097631	5.140515	5.140245	5.888789	5.226533	5.474027	10	5.474079	5.473608	10
210	2.112811	5.434958	5.765191	10	10	10	10	10	10	10	10

EXCLI Journal 2020;19:48-70 – ISSN 1611-2156
Received: October 03, 2019, accepted: December 19, 2019, published: January 03, 2020

Absorption intensity											
**Wavelength (nm)**	**concentration of acetylshikon (M)**										
	A	*B*	*C*	*D*	*E*	*F*	*G*	*I*	*J*	*K*	*L*
	0.00	$8.0x10^{-7}$	$1.6x10^{-6}$	$2.0x10^{-6}$	$2.8x10^{-6}$	$4.0x10^{-6}$	$4.8x10^{-6}$	$6.0x10^{-6}$	$8.0x10^{-6}$	$1.2x10^{-5}$	$1.6x10^{-5}$
209	2.261205	5.083005	5.414889	5.71502	5.829703	10	10	6.528511	10	6.13064	10
208	2.42903	4.795611	4.979332	5.038719	5.01569	5.817477	5.191881	5.179803	6.294904	5.098758	5.054745
207	2.612743	4.769678	4.941391	5.851392	5.117448	5.618268	5.178494	10	10	5.116525	5.164444
206	2.802902	4.749311	5.36766	5.643035	4.925748	5.029881	4.985003	5.296184	10	5.105617	5.692751
205	3.015334	4.728328	5.378328	6.046496	10	5.181518	5.377925	10	10	10	10
204	3.179162	4.423756	5.187636	5.069626	4.616287	5.080839	5.015731	5.948695	10	5.50087	5.153824
203	3.305161	4.268508	4.338969	6.469727	4.499712	4.611552	4.443826	4.563302	5.428847	4.731991	5.081705
202	3.286855	3.943058	4.452195	4.630305	4.062296	4.535076	4.385973	4.741271	4.960819	4.566672	4.518555
201	2.99027	3.421743	3.641335	4.12943	3.390859	3.617455	3.743146	3.548994	3.799789	3.739076	3.806996
200	2.472817	2.820551	2.884769	3.295441	2.907231	3.171117	3.215416	3.231407	3.59499	3.167404	3.01821

EXCLI Journal 2020;19:48-70 – ISSN 1611-2156
Received: October 03, 2019, accepted: December 19, 2019, published: January 03, 2020

Table 3: Absorption intensity in the wavelength range of 200-800 nm for absorption spectra of HSA fixed concentration (2.00 x 10^{-6} M), in the absence (*A*) and presence of increasing concentration of *β*-hydroxyisovalerylshikonin (*B-L*)

Absorption intensity											
**Wavelength (nm)**	**concentration of *β*-hydroxyisovalerylshikonin (M)**										
	A	*B*	*C*	*D*	*E*	*F*	*G*	*I*	*J*	*K*	*L*
	0.00	8.0x10^{-7}	1.6x10^{-6}	2.0x10^{-6}	2.8x10^{-6}	4.0x10^{-6}	4.8x10^{-6}	6.0x10^{-6}	8.0x10^{-6}	1.2x10^{-5}	1.6x10^{-5}
800	0.004447	0.003406	0.00385	0.004586	0.004051	0.004045	0.003598	0.003985	0.00345	0.003803	0.004768
799	0.005225	0.004637	0.004753	0.005295	0.004974	0.004938	0.004626	0.004658	0.00405	0.004712	0.00556
798	0.005455	0.004689	0.004916	0.005409	0.005071	0.005031	0.004499	0.004919	0.004067	0.004735	0.005929
797	0.00502	0.004541	0.004888	0.005158	0.004769	0.004988	0.004195	0.004662	0.004151	0.004921	0.005797
796	0.005167	0.004577	0.004982	0.005406	0.004585	0.005078	0.00425	0.004918	0.004144	0.004682	0.005655
795	0.005088	0.004448	0.004969	0.005287	0.004663	0.004982	0.004338	0.004662	0.003981	0.004707	0.005392
794	0.004902	0.004298	0.004893	0.005641	0.004885	0.005042	0.004526	0.004974	0.004063	0.004549	0.005726
793	0.005033	0.004249	0.004926	0.005588	0.004676	0.00513	0.004301	0.00507	0.004292	0.00485	0.005659
792	0.005273	0.004657	0.004988	0.005341	0.004774	0.004908	0.004491	0.005145	0.004301	0.004814	0.005863
791	0.00524	0.004826	0.005198	0.005509	0.004936	0.005077	0.004103	0.004851	0.004297	0.004652	0.005849
790	0.004852	0.004395	0.004701	0.005635	0.004924	0.005236	0.004291	0.004991	0.004283	0.004682	0.005638
789	0.005134	0.004632	0.005015	0.00529	0.004863	0.005246	0.004184	0.004654	0.004412	0.00448	0.005658
788	0.00509	0.004359	0.004848	0.005345	0.005064	0.004918	0.004259	0.004935	0.004084	0.00494	0.005811
787	0.005103	0.004412	0.00486	0.005442	0.004837	0.00531	0.00438	0.004911	0.004119	0.004738	0.005745
786	0.005541	0.004618	0.005025	0.005485	0.00521	0.00519	0.004561	0.004999	0.00445	0.004732	0.005797
785	0.005294	0.004724	0.004796	0.005566	0.004811	0.005042	0.004487	0.004907	0.004111	0.004898	0.005833
784	0.005188	0.004635	0.005014	0.00556	0.004548	0.005094	0.004136	0.004771	0.00435	0.00464	0.005929
783	0.005183	0.004761	0.004899	0.00552	0.004458	0.005044	0.004393	0.004791	0.004247	0.004765	0.00597
782	0.005223	0.004584	0.004983	0.005344	0.004917	0.005215	0.004569	0.004642	0.004214	0.004854	0.005968
781	0.004988	0.004578	0.004941	0.005413	0.004798	0.005332	0.004362	0.004672	0.004144	0.004965	0.005842
780	0.005395	0.004612	0.004846	0.005486	0.004989	0.005241	0.004329	0.005035	0.004386	0.004771	0.005911
779	0.005078	0.004422	0.004791	0.005561	0.004611	0.005041	0.004111	0.004864	0.00434	0.004922	0.005981
778	0.004994	0.004439	0.004893	0.005345	0.004759	0.005045	0.004383	0.005062	0.004407	0.004761	0.005965
777	0.00509	0.004561	0.005014	0.005612	0.004798	0.005304	0.004552	0.005063	0.00429	0.004752	0.005733
776	0.005278	0.004699	0.005138	0.005625	0.005017	0.00499	0.004245	0.00495	0.004211	0.004876	0.005995
775	0.005236	0.004456	0.005211	0.005482	0.004853	0.005193	0.00456	0.004949	0.004309	0.004896	0.005965
774	0.005217	0.004722	0.005044	0.005458	0.004641	0.0052	0.00443	0.004919	0.004169	0.004627	0.006073
773	0.005329	0.00465	0.004931	0.005573	0.004845	0.005232	0.004376	0.005103	0.004335	0.004793	0.005972
772	0.005287	0.004524	0.005115	0.005661	0.00488	0.004932	0.004387	0.004906	0.004291	0.004762	0.006245
771	0.005095	0.004475	0.004839	0.005534	0.004642	0.005088	0.004208	0.005124	0.004332	0.004898	0.005805

Absorption intensity											
**Wavelength (nm)**	**concentration of β-hydroxyisovalerylshikonin (M)**										
	A	*B*	*C*	*D*	*E*	*F*	*G*	*I*	*J*	*K*	*L*
	0.00	8.0×10^{-7}	1.6×10^{-6}	2.0×10^{-6}	2.8×10^{-6}	4.0×10^{-6}	4.8×10^{-6}	6.0×10^{-6}	8.0×10^{-6}	1.2×10^{-5}	1.6×10^{-5}
770	0.005247	0.004348	0.004858	0.005561	0.004765	0.005007	0.004326	0.004867	0.004368	0.005128	0.005913
769	0.005083	0.004443	0.004972	0.005254	0.004588	0.005244	0.004277	0.004785	0.004087	0.00474	0.006063
768	0.00525	0.004631	0.004734	0.005264	0.004853	0.005207	0.004366	0.004725	0.004328	0.004776	0.006167
767	0.005374	0.004646	0.005105	0.005441	0.004764	0.005209	0.004425	0.004897	0.004208	0.004982	0.00621
766	0.005115	0.004496	0.004739	0.005616	0.004836	0.005183	0.004406	0.004804	0.004334	0.005007	0.006172
765	0.005102	0.004405	0.004866	0.005569	0.004917	0.00512	0.004435	0.004845	0.004191	0.005008	0.006054
764	0.005097	0.004531	0.004954	0.005472	0.004801	0.005094	0.004428	0.004948	0.00416	0.004981	0.006253
763	0.005051	0.004565	0.004677	0.005602	0.004745	0.005255	0.004497	0.004998	0.004412	0.00505	0.00639
762	0.005275	0.004469	0.005105	0.005714	0.004809	0.00527	0.004447	0.004983	0.004345	0.005156	0.0065
761	0.005396	0.004211	0.004792	0.005581	0.004571	0.005325	0.004315	0.004841	0.004141	0.004977	0.006289
760	0.005139	0.004387	0.004879	0.005568	0.004796	0.00523	0.004341	0.005209	0.004238	0.004947	0.00617
759	0.005369	0.004536	0.005002	0.005609	0.004911	0.005213	0.004725	0.005164	0.004424	0.005276	0.006366
758	0.005204	0.004675	0.005139	0.005872	0.0049	0.00535	0.004646	0.005082	0.004558	0.005176	0.006546
757	0.005144	0.00442	0.005071	0.005596	0.004784	0.005313	0.004444	0.005094	0.004328	0.005247	0.00644
756	0.00526	0.004449	0.004904	0.005405	0.004789	0.005218	0.004533	0.004965	0.004404	0.005001	0.00636
755	0.005145	0.004592	0.004964	0.005582	0.004763	0.005043	0.004623	0.004905	0.004541	0.005086	0.006619
754	0.005296	0.004553	0.005049	0.005582	0.004862	0.005217	0.004682	0.004788	0.004195	0.005221	0.006567
753	0.005066	0.004452	0.005024	0.005788	0.004829	0.00506	0.004479	0.005069	0.004406	0.005145	0.006569
752	0.0053	0.004511	0.004932	0.00551	0.004764	0.005163	0.004483	0.005018	0.004685	0.005209	0.006551
751	0.005255	0.004544	0.004975	0.005643	0.005027	0.005359	0.004699	0.00493	0.004654	0.005307	0.006655
750	0.005424	0.00452	0.004919	0.005696	0.004986	0.005259	0.004454	0.005177	0.004657	0.005342	0.006799
749	0.005265	0.004509	0.00511	0.005815	0.004887	0.005204	0.004622	0.005236	0.004475	0.005262	0.006772
748	0.00538	0.004582	0.004989	0.005891	0.004776	0.005307	0.004677	0.005296	0.004501	0.005491	0.006835
747	0.005303	0.004485	0.004968	0.005857	0.004648	0.005313	0.004552	0.005008	0.004533	0.005539	0.00706
746	0.005498	0.004533	0.005152	0.005745	0.004821	0.005245	0.004616	0.005163	0.004738	0.005239	0.007067
745	0.005142	0.004549	0.005082	0.005868	0.004974	0.005218	0.004453	0.005321	0.004717	0.005297	0.006749
744	0.00529	0.004385	0.004884	0.005752	0.004792	0.005359	0.004745	0.005317	0.004533	0.00518	0.00693
743	0.005167	0.004642	0.004915	0.005771	0.004965	0.00539	0.004537	0.005149	0.004656	0.005326	0.007052
742	0.005289	0.004244	0.005043	0.005787	0.00489	0.005321	0.004523	0.00536	0.004485	0.005477	0.006966
741	0.005163	0.004444	0.004967	0.005863	0.004921	0.005247	0.004701	0.005249	0.004656	0.005382	0.007101
740	0.005325	0.004482	0.004924	0.005819	0.004878	0.005459	0.004639	0.005284	0.004617	0.005446	0.007306
739	0.005199	0.004417	0.004867	0.005733	0.005058	0.005224	0.004642	0.0053	0.004798	0.005686	0.007218
738	0.005154	0.004624	0.005175	0.005754	0.004821	0.005337	0.004972	0.00546	0.004832	0.00568	0.007433

EXCLI Journal 2020;19:48-70 – ISSN 1611-2156
Received: October 03, 2019, accepted: December 19, 2019, published: January 03, 2020

Wavelength (nm)	***Absorption intensity***										
	**concentration of β-hydroxyisovalerylshikonin (M)**										
	A	*B*	*C*	*D*	*E*	*F*	*G*	*I*	*J*	*K*	*L*
	0.00	$8.0x10^{-7}$	$1.6x10^{-6}$	$2.0x10^{-6}$	$2.8x10^{-6}$	$4.0x10^{-6}$	$4.8x10^{-6}$	$6.0x10^{-6}$	$8.0x10^{-6}$	$1.2x10^{-5}$	$1.6x10^{-5}$
737	0.005095	0.004702	0.004972	0.00576	0.004794	0.005329	0.004551	0.005415	0.004596	0.005696	0.007622
736	0.005091	0.004465	0.005352	0.005703	0.004862	0.00548	0.004738	0.005507	0.004791	0.005928	0.007535
735	0.005158	0.004482	0.005077	0.00595	0.004969	0.005526	0.004693	0.00549	0.004927	0.005893	0.007832
734	0.005122	0.004543	0.005044	0.005899	0.005093	0.005455	0.004809	0.00543	0.004967	0.00594	0.008068
733	0.0052	0.004548	0.005081	0.005728	0.004926	0.005498	0.004708	0.005443	0.004981	0.005975	0.008003
732	0.005219	0.004241	0.004931	0.005728	0.004829	0.005368	0.004764	0.005474	0.005127	0.006023	0.008054
731	0.005047	0.004402	0.005056	0.005759	0.00474	0.005363	0.004904	0.00543	0.005057	0.006097	0.008438
730	0.005152	0.004359	0.004897	0.005692	0.004739	0.005525	0.004731	0.005525	0.005028	0.006225	0.008431
729	0.004864	0.004241	0.004833	0.005606	0.00474	0.005512	0.004815	0.005474	0.005044	0.006131	0.008434
728	0.004979	0.00435	0.004835	0.005762	0.004788	0.005432	0.004753	0.005367	0.005092	0.006092	0.008645
727	0.004766	0.004119	0.004626	0.005515	0.004721	0.005242	0.004649	0.005576	0.004945	0.006289	0.008443
726	0.004612	0.0042	0.004656	0.005414	0.004824	0.005087	0.004712	0.005327	0.004916	0.006277	0.008632
725	0.004666	0.003936	0.004467	0.00525	0.004473	0.004992	0.004397	0.005198	0.00481	0.006135	0.008573
724	0.004516	0.003881	0.004304	0.005201	0.004274	0.004871	0.004487	0.005251	0.004565	0.006159	0.00862
723	0.004329	0.003553	0.004129	0.005042	0.004243	0.004829	0.004369	0.004933	0.00453	0.005899	0.008642
722	0.00416	0.003497	0.003993	0.004897	0.004172	0.004614	0.004212	0.004896	0.004306	0.005886	0.008664
721	0.003963	0.003338	0.003807	0.004615	0.00387	0.004454	0.003854	0.004756	0.004284	0.005631	0.00842
720	0.003796	0.0031	0.003674	0.004594	0.003861	0.00431	0.003647	0.004817	0.004168	0.005673	0.008447
719	0.003593	0.00289	0.003474	0.004485	0.003577	0.004118	0.003586	0.004413	0.00386	0.005407	0.008185
718	0.003639	0.003039	0.003633	0.004407	0.003764	0.004253	0.003766	0.004522	0.004	0.00564	0.00834
717	0.003479	0.00303	0.00365	0.004456	0.00368	0.004053	0.003623	0.004489	0.004001	0.005572	0.008419
716	0.003408	0.002637	0.00325	0.004286	0.003382	0.004076	0.003492	0.004253	0.003982	0.005423	0.008321
715	0.003202	0.002528	0.003053	0.003977	0.003321	0.003781	0.003387	0.004178	0.003644	0.005399	0.008387
714	0.002993	0.002346	0.002988	0.003868	0.003293	0.003654	0.003155	0.004031	0.003653	0.00517	0.008198
713	0.002848	0.002125	0.002697	0.003675	0.002884	0.003536	0.003065	0.003864	0.003491	0.005077	0.00828
712	0.002652	0.002059	0.002638	0.003674	0.002925	0.003367	0.002907	0.003709	0.003435	0.005188	0.008159
711	0.002482	0.001845	0.002531	0.003468	0.002779	0.003245	0.002796	0.003671	0.003244	0.004972	0.008062
710	0.0023	0.001723	0.002359	0.003421	0.002556	0.003132	0.002702	0.003602	0.003311	0.00498	0.008183
709	0.002309	0.001553	0.002378	0.003201	0.002524	0.003166	0.002653	0.003563	0.003256	0.004955	0.008414
708	0.00224	0.00165	0.002226	0.003303	0.002432	0.003077	0.002526	0.003445	0.003282	0.005137	0.008259
707	0.001977	0.001531	0.002214	0.003056	0.002417	0.003093	0.002589	0.003525	0.003139	0.005003	0.008431
706	0.002204	0.001473	0.002072	0.003087	0.002338	0.003027	0.00267	0.003545	0.003311	0.005142	0.008623
705	0.002066	0.001422	0.002244	0.003158	0.002374	0.003037	0.002622	0.003477	0.003296	0.005085	0.008482

Absorption intensity											
**Wavelength (nm)**	**concentration of β-hydroxyisovalerylshikonin (M)**										
	A	*B*	*C*	*D*	*E*	*F*	*G*	*I*	*J*	*K*	*L*
	0.00	$8.0x10^{-7}$	$1.6x10^{-6}$	$2.0x10^{-6}$	$2.8x10^{-6}$	$4.0x10^{-6}$	$4.8x10^{-6}$	$6.0x10^{-6}$	$8.0x10^{-6}$	$1.2x10^{-5}$	$1.6x10^{-5}$
704	0.002176	0.001468	0.002233	0.003193	0.002508	0.003234	0.002619	0.003469	0.003214	0.005218	0.008681
703	0.002183	0.001494	0.002169	0.003187	0.002521	0.003011	0.00252	0.003696	0.003446	0.005322	0.009058
702	0.002176	0.001559	0.002255	0.003154	0.00236	0.003091	0.002793	0.003808	0.003564	0.005703	0.00918
701	0.002121	0.001507	0.002438	0.003563	0.00262	0.003114	0.002749	0.003672	0.003703	0.005389	0.009334
700	0.002407	0.001622	0.002435	0.003401	0.002575	0.003336	0.003106	0.003888	0.003576	0.005825	0.009579
699	0.002246	0.001792	0.002527	0.003263	0.002657	0.00336	0.002953	0.003996	0.003854	0.005842	0.009615
698	0.002319	0.001928	0.002602	0.003653	0.002922	0.003498	0.003087	0.004127	0.003963	0.006243	0.010164
697	0.0025	0.001774	0.002386	0.003644	0.002862	0.003622	0.00325	0.004286	0.003954	0.006285	0.010385
696	0.002543	0.002004	0.002607	0.00378	0.002974	0.00368	0.003386	0.004313	0.004326	0.006457	0.010611
695	0.002403	0.001975	0.00267	0.003701	0.003038	0.003712	0.00346	0.004527	0.004348	0.006628	0.010918
694	0.002638	0.00193	0.002837	0.003946	0.003182	0.003767	0.00363	0.004706	0.004483	0.006806	0.010909
693	0.002632	0.002193	0.002809	0.004061	0.003198	0.004039	0.003621	0.00482	0.004698	0.007033	0.011328
692	0.002768	0.002152	0.003109	0.004078	0.003386	0.004046	0.003711	0.004968	0.004815	0.007088	0.011606
691	0.00283	0.002184	0.003102	0.004255	0.003257	0.004178	0.003898	0.004908	0.004846	0.007215	0.011874
690	0.002799	0.002399	0.003114	0.00428	0.003498	0.00425	0.004049	0.005019	0.005067	0.007629	0.012129
689	0.00284	0.002335	0.003263	0.004387	0.003469	0.00442	0.004035	0.005214	0.005098	0.007774	0.012245
688	0.00305	0.002344	0.003145	0.004397	0.003619	0.004463	0.004221	0.005314	0.005471	0.007922	0.012631
687	0.003016	0.002488	0.00328	0.004295	0.003694	0.004584	0.004312	0.005361	0.005316	0.008083	0.012952
686	0.002966	0.002614	0.0035	0.004514	0.003764	0.004653	0.004311	0.005421	0.005588	0.00835	0.013222
685	0.002989	0.002589	0.00335	0.004559	0.003766	0.004766	0.004482	0.005608	0.005771	0.008417	0.013311
684	0.0032	0.002409	0.003372	0.004399	0.003721	0.004595	0.004378	0.005424	0.005749	0.008377	0.013576
683	0.003081	0.002625	0.003605	0.004525	0.003858	0.004737	0.004735	0.005515	0.005918	0.008263	0.013264
682	0.003289	0.00273	0.003546	0.004717	0.004081	0.005017	0.004837	0.005824	0.005981	0.008986	0.013991
681	0.003147	0.002683	0.003757	0.004716	0.004	0.005009	0.004802	0.005833	0.006149	0.008952	0.014332
680	0.003338	0.00273	0.003708	0.004764	0.004063	0.004948	0.004861	0.006171	0.00628	0.009143	0.014621
679	0.003219	0.002735	0.003573	0.004854	0.004258	0.005137	0.005145	0.006129	0.006454	0.009476	0.015056
678	0.003186	0.002692	0.003716	0.004808	0.00424	0.005161	0.004936	0.00627	0.006676	0.009563	0.015284
677	0.003249	0.00281	0.003905	0.004966	0.004258	0.005328	0.005099	0.006522	0.00674	0.009762	0.015335
676	0.003351	0.002753	0.003746	0.005026	0.004447	0.005495	0.005218	0.006428	0.006765	0.009912	0.015804
675	0.003373	0.002686	0.003702	0.005042	0.004388	0.00551	0.005178	0.00662	0.00682	0.010098	0.016008
674	0.003412	0.00279	0.003852	0.005027	0.004386	0.005581	0.005298	0.006789	0.007153	0.010406	0.01633
673	0.003294	0.002744	0.003858	0.005163	0.00451	0.005638	0.005378	0.006722	0.007243	0.010503	0.016564
672	0.003348	0.002747	0.003827	0.005392	0.004645	0.005808	0.005701	0.006902	0.007268	0.010723	0.017101

EXCLI Journal 2020;19:48-70 – ISSN 1611-2156
Received: October 03, 2019, accepted: December 19, 2019, published: January 03, 2020

Absorption intensity											
**Wavelength (nm)**	**concentration of β-hydroxyisovalerylshikonin (M)**										
	A	*B*	*C*	*D*	*E*	*F*	*G*	*I*	*J*	*K*	*L*
	0.00	$8.0x10^{-7}$	$1.6x10^{-6}$	$2.0x10^{-6}$	$2.8x10^{-6}$	$4.0x10^{-6}$	$4.8x10^{-6}$	$6.0x10^{-6}$	$8.0x10^{-6}$	$1.2x10^{-5}$	$1.6x10^{-5}$
671	0.003583	0.003011	0.004169	0.005293	0.004596	0.005811	0.005518	0.007012	0.007473	0.010888	0.017232
670	0.00372	0.003063	0.004389	0.005643	0.005009	0.006028	0.006006	0.007375	0.007881	0.011338	0.017939
669	0.004088	0.003572	0.00482	0.00621	0.005405	0.006608	0.006535	0.007863	0.008372	0.012068	0.018618
668	0.00416	0.003669	0.00491	0.006247	0.005553	0.006867	0.006557	0.008112	0.008689	0.012115	0.019009
667	0.00371	0.003326	0.004553	0.005759	0.005167	0.006473	0.006425	0.007728	0.008448	0.012005	0.018818
666	0.003586	0.003237	0.004363	0.005807	0.005066	0.006315	0.006302	0.007653	0.008372	0.012	0.018842
665	0.003373	0.003199	0.00414	0.005628	0.00489	0.006222	0.006069	0.007691	0.008226	0.012086	0.019008
664	0.00347	0.002924	0.004238	0.005498	0.004989	0.006108	0.006177	0.00769	0.008371	0.012223	0.019242
663	0.003304	0.00273	0.003996	0.005462	0.004798	0.006243	0.006205	0.007626	0.008415	0.01233	0.019625
662	0.003178	0.00289	0.003998	0.00533	0.004758	0.006129	0.006088	0.007738	0.008492	0.012472	0.019866
661	0.003151	0.002708	0.004077	0.005284	0.0047	0.006179	0.006188	0.007757	0.008596	0.012652	0.020112
660	0.003005	0.002737	0.004072	0.005338	0.004899	0.006194	0.006298	0.007815	0.008759	0.012757	0.020571
659	0.003065	0.002629	0.003977	0.005298	0.004759	0.006114	0.006112	0.007846	0.008668	0.012895	0.020701
658	0.003084	0.002708	0.003895	0.005356	0.004715	0.006151	0.006173	0.007848	0.008892	0.013138	0.020965
657	0.002899	0.00253	0.003887	0.005239	0.004846	0.006232	0.006285	0.008032	0.008862	0.013287	0.021389
656	0.002826	0.002554	0.003808	0.005258	0.004835	0.006172	0.0064	0.008072	0.008949	0.013538	0.021764
655	0.002716	0.002455	0.003741	0.005332	0.004796	0.00618	0.006235	0.008182	0.009018	0.013557	0.021954
654	0.002766	0.002228	0.003924	0.005327	0.004805	0.006346	0.006452	0.008221	0.009096	0.013914	0.022365
653	0.002641	0.002483	0.003761	0.005166	0.004759	0.006071	0.006376	0.008221	0.00928	0.014039	0.022658
652	0.002563	0.002384	0.003747	0.005279	0.004842	0.006261	0.006464	0.008327	0.009379	0.014176	0.023094
651	0.002496	0.002118	0.003695	0.005285	0.004833	0.006258	0.006434	0.008453	0.009318	0.014491	0.023269
650	0.002462	0.002183	0.003713	0.00508	0.004799	0.006357	0.006493	0.008427	0.009581	0.014543	0.023651
649	0.002197	0.002147	0.003608	0.0051	0.004672	0.006272	0.006476	0.008405	0.009728	0.014654	0.023927
648	0.002272	0.001955	0.003604	0.005033	0.00473	0.006338	0.006543	0.008526	0.009728	0.014952	0.024376
647	0.002208	0.002036	0.003658	0.005014	0.004578	0.006377	0.006542	0.008504	0.009871	0.0151	0.024735
646	0.002224	0.001902	0.003506	0.005094	0.004687	0.006429	0.006504	0.008772	0.009964	0.015294	0.025217
645	0.002129	0.001885	0.003408	0.004966	0.004792	0.006362	0.006745	0.008669	0.010011	0.01559	0.02546
644	0.002031	0.001811	0.003377	0.005089	0.004634	0.00637	0.006643	0.008784	0.010227	0.015703	0.025938
643	0.002118	0.001922	0.003405	0.004964	0.004751	0.006596	0.006714	0.008828	0.010329	0.016004	0.026259
642	0.001782	0.001641	0.003342	0.004879	0.00479	0.006579	0.006825	0.008907	0.010424	0.016261	0.026741
641	0.001833	0.001616	0.003398	0.004964	0.004681	0.006527	0.006815	0.009037	0.010492	0.016332	0.027138
640	0.001814	0.001697	0.003218	0.004925	0.004589	0.006609	0.006936	0.009067	0.010741	0.016718	0.027443
639	0.001783	0.001665	0.00342	0.00488	0.004746	0.006642	0.006819	0.009153	0.010755	0.016864	0.027971

EXCLI Journal 2020;19:48-70 – ISSN 1611-2156
Received: October 03, 2019, accepted: December 19, 2019, published: January 03, 2020

Absorption intensity											
**Wavelength (nm)**	**concentration of β-hydroxyisovalerylshikonin (M)**										
	A	*B*	*C*	*D*	*E*	*F*	*G*	*I*	*J*	*K*	*L*
	0.00	$8.0x10^{-7}$	$1.6x10^{-6}$	$2.0x10^{-6}$	$2.8x10^{-6}$	$4.0x10^{-6}$	$4.8x10^{-6}$	$6.0x10^{-6}$	$8.0x10^{-6}$	$1.2x10^{-5}$	$1.6x10^{-5}$
638	0.001703	0.001569	0.003345	0.004974	0.004689	0.00668	0.00703	0.009258	0.011081	0.01721	0.028248
637	0.001596	0.001415	0.003302	0.004974	0.004664	0.006789	0.007056	0.009475	0.011064	0.017336	0.02866
636	0.001595	0.001562	0.003346	0.005032	0.004645	0.00667	0.007193	0.009581	0.011324	0.017677	0.029218
635	0.001495	0.001526	0.003371	0.004996	0.004721	0.006834	0.007247	0.009738	0.011493	0.018101	0.029717
634	0.001558	0.00147	0.003388	0.005045	0.004814	0.006945	0.007347	0.009707	0.011657	0.018127	0.030125
633	0.001554	0.001585	0.003277	0.00501	0.004955	0.006974	0.007388	0.009876	0.011782	0.018513	0.030738
632	0.00141	0.001498	0.003403	0.005105	0.004915	0.007095	0.007483	0.009966	0.012007	0.018728	0.031083
631	0.001429	0.001509	0.003323	0.005039	0.004857	0.007152	0.007499	0.010213	0.012306	0.019131	0.031435
630	0.001476	0.001508	0.003353	0.005078	0.005112	0.007202	0.00768	0.010352	0.012251	0.019342	0.031927
629	0.001486	0.001462	0.003383	0.005329	0.005159	0.007506	0.007822	0.010484	0.012625	0.019789	0.03248
628	0.001596	0.001523	0.003557	0.00526	0.005201	0.007451	0.007851	0.010624	0.012762	0.01993	0.033069
627	0.001532	0.001564	0.003584	0.005337	0.005243	0.007562	0.008082	0.010821	0.012919	0.020303	0.033434
626	0.001535	0.001568	0.003632	0.005348	0.005245	0.00766	0.008232	0.010914	0.013176	0.02063	0.034093
625	0.001469	0.001614	0.003644	0.005514	0.005372	0.007783	0.008146	0.011121	0.013348	0.020868	0.034466
624	0.001551	0.001478	0.003686	0.005557	0.005592	0.007891	0.008336	0.011316	0.01355	0.021221	0.034964
623	0.001562	0.001691	0.003751	0.005573	0.005602	0.008002	0.008614	0.011442	0.013884	0.02155	0.03538
622	0.001688	0.001584	0.003752	0.005643	0.005465	0.008014	0.008583	0.011612	0.014029	0.021931	0.036031
621	0.001576	0.00172	0.00399	0.005881	0.005666	0.008138	0.008681	0.01175	0.014121	0.022111	0.036239
620	0.001707	0.001738	0.003935	0.005861	0.00582	0.008259	0.008882	0.011988	0.014468	0.022526	0.036866
619	0.00171	0.001796	0.004	0.005885	0.005866	0.008495	0.008963	0.012104	0.014529	0.022725	0.037146
618	0.0017	0.001912	0.00408	0.006008	0.005973	0.008534	0.009108	0.012231	0.014651	0.023058	0.037691
617	0.001816	0.001824	0.004126	0.006023	0.005951	0.008575	0.009222	0.012324	0.014847	0.023225	0.037968
616	0.001987	0.001994	0.004156	0.006096	0.006245	0.008809	0.009427	0.012462	0.01515	0.023394	0.038548
615	0.00198	0.002017	0.004211	0.006206	0.00606	0.00884	0.009515	0.012641	0.015183	0.023781	0.03884
614	0.00199	0.002065	0.004319	0.006195	0.00628	0.008909	0.00954	0.012851	0.015567	0.024008	0.03932
613	0.002031	0.001956	0.00437	0.006327	0.00623	0.009042	0.009737	0.012913	0.015603	0.024187	0.039651
612	0.002007	0.002029	0.004348	0.00638	0.006386	0.009127	0.009755	0.012981	0.015812	0.024533	0.040023
611	0.002028	0.002189	0.004431	0.006565	0.006463	0.009071	0.009915	0.013053	0.015868	0.024706	0.040419
610	0.002025	0.002046	0.004381	0.006408	0.006568	0.009147	0.009944	0.013268	0.015978	0.024966	0.040765
609	0.001924	0.002115	0.0045	0.006462	0.006597	0.009229	0.009918	0.013309	0.016166	0.025289	0.041077
608	0.001921	0.00211	0.004448	0.006659	0.006596	0.009331	0.010131	0.013435	0.016217	0.025464	0.041587
607	0.002057	0.002009	0.004362	0.006492	0.00659	0.00949	0.010163	0.013525	0.016306	0.025522	0.04186
606	0.001869	0.002093	0.004478	0.006512	0.006571	0.009375	0.010046	0.013527	0.016447	0.025778	0.042096

EXCLI Journal 2020;19:48-70 – ISSN 1611-2156
Received: October 03, 2019, accepted: December 19, 2019, published: January 03, 2020

Absorption intensity											
**Wavelength (nm)**	**concentration of β-hydroxyisovalerylshikonin (M)**										
	A	*B*	*C*	*D*	*E*	*F*	*G*	*I*	*J*	*K*	*L*
	0.00	$8.0x10^{-7}$	$1.6x10^{-6}$	$2.0x10^{-6}$	$2.8x10^{-6}$	$4.0x10^{-6}$	$4.8x10^{-6}$	$6.0x10^{-6}$	$8.0x10^{-6}$	$1.2x10^{-5}$	$1.6x10^{-5}$
605	0.001876	0.001933	0.004387	0.006515	0.00653	0.00927	0.010297	0.013585	0.016583	0.025882	0.042493
604	0.001868	0.001888	0.004401	0.006517	0.006514	0.009427	0.010197	0.013701	0.016465	0.026127	0.042739
603	0.001722	0.001949	0.004318	0.006395	0.006402	0.009379	0.010089	0.013669	0.016613	0.026194	0.042965
602	0.001577	0.001711	0.004271	0.006368	0.006365	0.009212	0.010253	0.013488	0.0167	0.026249	0.043239
601	0.00161	0.00161	0.004162	0.006262	0.006368	0.009277	0.010219	0.013699	0.016597	0.026332	0.043407
600	0.001322	0.001642	0.004008	0.006199	0.006263	0.009242	0.010068	0.013529	0.016739	0.026421	0.043645
599	0.001213	0.001372	0.003963	0.00599	0.006151	0.008992	0.009976	0.013426	0.016547	0.02643	0.043913
598	0.001038	0.001234	0.003644	0.005897	0.006024	0.009076	0.009903	0.013335	0.016602	0.026506	0.044066
597	0.000863	0.00106	0.003592	0.00564	0.005925	0.008845	0.009788	0.013375	0.016587	0.026629	0.044316
596	0.000682	0.000897	0.003334	0.005618	0.005916	0.008722	0.009719	0.013261	0.016476	0.026706	0.044443
595	0.000633	0.000706	0.003323	0.005672	0.005654	0.00872	0.009577	0.013258	0.016457	0.026644	0.044657
594	0.000458	0.000632	0.003365	0.005375	0.005457	0.008529	0.009613	0.013173	0.016496	0.026916	0.044766
593	0.000287	0.000435	0.002952	0.005287	0.005395	0.008399	0.009543	0.013119	0.01643	0.026945	0.044861
592	0.000216	0.000301	0.0028	0.005371	0.005387	0.008486	0.009479	0.013301	0.016674	0.027022	0.045162
591	7.65E-05	0.000153	0.002862	0.005241	0.005343	0.008375	0.009353	0.013377	0.01653	0.026931	0.045464
590	-0.00022	0.000224	0.002787	0.005191	0.005227	0.008226	0.009545	0.013156	0.016629	0.026908	0.045784
589	-9.89E-06	0.000198	0.002677	0.005239	0.005179	0.008309	0.009466	0.013095	0.016458	0.027065	0.046073
588	-0.00011	0.000212	0.002578	0.004883	0.005004	0.007995	0.009269	0.0131	0.016431	0.027359	0.046235
587	1.03E-05	0.000205	0.002826	0.005129	0.005286	0.008438	0.009581	0.013525	0.016904	0.027953	0.047107
586	-4.18E-05	-1.05E-05	0.00267	0.005333	0.005504	0.00861	0.009569	0.013695	0.017011	0.028193	0.04745
585	-7.44E-05	8.50E-05	0.002843	0.005208	0.00539	0.008638	0.009675	0.013742	0.017224	0.028457	0.04796
584	-8.66E-05	5.41E-05	0.002882	0.005321	0.005401	0.008726	0.009938	0.013821	0.017475	0.028721	0.048446
583	-0.00011	0.000187	0.00286	0.00537	0.005526	0.008893	0.01	0.01401	0.017482	0.02893	0.04878
582	1.12E-05	0.000156	0.002873	0.005456	0.005659	0.009048	0.009954	0.014065	0.017736	0.029216	0.049221
581	2.26E-05	0.000113	0.003012	0.005538	0.005733	0.00905	0.010108	0.014148	0.017769	0.029498	0.049561
580	-6.88E-05	0.000275	0.002973	0.005605	0.005755	0.00927	0.010342	0.014367	0.018189	0.029743	0.050194
579	0.000162	0.000243	0.003156	0.005701	0.005838	0.009192	0.01055	0.014405	0.018359	0.030072	0.050527
578	0.000117	0.000351	0.00324	0.005755	0.005743	0.00927	0.010648	0.014566	0.018603	0.030399	0.050961
577	0.000165	0.000366	0.003245	0.005758	0.006032	0.009488	0.010492	0.014849	0.018747	0.03064	0.051359
576	0.000155	0.00037	0.003216	0.00592	0.006068	0.009629	0.010693	0.015041	0.018841	0.030861	0.051824
575	0.0003	0.000624	0.003394	0.005959	0.006124	0.00977	0.010887	0.015136	0.019043	0.031123	0.052369
574	0.000315	0.000447	0.003298	0.006038	0.006278	0.009648	0.011039	0.015312	0.01915	0.031289	0.052506
573	0.000268	0.000547	0.00346	0.006083	0.006289	0.00986	0.01106	0.015233	0.019345	0.03156	0.053055

EXCLI Journal 2020;19:48-70 – ISSN 1611-2156
Received: October 03, 2019, accepted: December 19, 2019, published: January 03, 2020

Wavelength (nm)	***Absorption intensity***										
	**concentration of β-hydroxyisovalerylshikonin (M)**										
	A	*B*	*C*	*D*	*E*	*F*	*G*	*I*	*J*	*K*	*L*
	0.00	$8.0x10^{-7}$	$1.6x10^{-6}$	$2.0x10^{-6}$	$2.8x10^{-6}$	$4.0x10^{-6}$	$4.8x10^{-6}$	$6.0x10^{-6}$	$8.0x10^{-6}$	$1.2x10^{-5}$	$1.6x10^{-5}$
572	0.000367	0.000563	0.00353	0.006147	0.006384	0.009756	0.011218	0.015497	0.019339	0.031955	0.053247
571	0.000356	0.000675	0.003494	0.006217	0.006294	0.009876	0.011323	0.015687	0.019464	0.031972	0.053726
570	0.000531	0.00074	0.003697	0.006299	0.006414	0.01009	0.011444	0.01562	0.019718	0.032228	0.05409
569	0.000346	0.000668	0.00357	0.006367	0.006508	0.010046	0.011426	0.015795	0.019797	0.032352	0.05434
568	0.000446	0.000679	0.003683	0.006318	0.006587	0.010285	0.011536	0.016001	0.020072	0.032625	0.054656
567	0.001071	0.001141	0.004079	0.006801	0.007153	0.010664	0.011994	0.016425	0.020563	0.033312	0.055393
566	0.001053	0.001226	0.004183	0.007066	0.007024	0.010812	0.012094	0.016588	0.020795	0.033564	0.055623
565	0.000965	0.001204	0.004274	0.006796	0.007112	0.010813	0.012159	0.016655	0.020756	0.033587	0.055949
564	0.001035	0.001224	0.004252	0.006909	0.007247	0.01084	0.012268	0.01662	0.020891	0.033821	0.056333
563	0.001033	0.001265	0.004286	0.006983	0.00719	0.010942	0.012308	0.01689	0.021206	0.034028	0.056485
562	0.00107	0.001315	0.004312	0.007158	0.00734	0.011116	0.01242	0.016937	0.021122	0.034305	0.056479
561	0.001208	0.001279	0.004307	0.007253	0.007251	0.010977	0.012524	0.016875	0.021192	0.034278	0.056993
560	0.001096	0.001263	0.004415	0.007132	0.007404	0.011135	0.012475	0.017116	0.021219	0.034466	0.057248
559	0.001046	0.001359	0.004383	0.007108	0.007225	0.011239	0.012569	0.017081	0.021307	0.034567	0.057514
558	0.001136	0.001204	0.004292	0.007298	0.007515	0.011203	0.012562	0.017078	0.02136	0.034781	0.057663
557	0.001273	0.001188	0.004343	0.007182	0.007214	0.011202	0.012537	0.017168	0.021478	0.034886	0.05797
556	0.001122	0.001248	0.004387	0.007272	0.007408	0.011206	0.012528	0.017148	0.021576	0.035017	0.058181
555	0.001033	0.001149	0.00427	0.007081	0.007319	0.011167	0.012639	0.017194	0.021698	0.035079	0.058258
554	0.001069	0.001167	0.004219	0.007046	0.007276	0.011341	0.012629	0.017299	0.02157	0.035014	0.058625
553	0.000846	0.001079	0.0044	0.007173	0.007321	0.011166	0.012634	0.017224	0.021634	0.035309	0.058683
552	0.001003	0.001122	0.004195	0.007167	0.007416	0.011249	0.012655	0.017249	0.021669	0.035252	0.05893
551	0.000826	0.001085	0.004222	0.007165	0.007246	0.011166	0.012579	0.017373	0.021644	0.035362	0.05925
550	0.000856	0.000918	0.004301	0.006994	0.007272	0.011158	0.012637	0.017233	0.021884	0.035507	0.059374
549	0.000708	0.000892	0.004138	0.007128	0.007285	0.011193	0.012668	0.017171	0.021756	0.035651	0.059517
548	0.00083	0.00083	0.004082	0.006975	0.007224	0.011217	0.01259	0.017432	0.021721	0.035644	0.059719
547	0.000822	0.000973	0.004034	0.006914	0.00729	0.011068	0.01266	0.017446	0.021881	0.03572	0.059941
546	0.000703	0.000666	0.003904	0.006902	0.007254	0.011066	0.012651	0.017277	0.021865	0.03596	0.060095
545	0.000616	0.000742	0.00394	0.006929	0.007258	0.011066	0.012613	0.017387	0.021936	0.035914	0.060259
544	0.000516	0.000531	0.00395	0.006796	0.007126	0.010896	0.012597	0.017355	0.021745	0.036019	0.060436
543	0.000491	0.000462	0.003733	0.006702	0.007154	0.011013	0.012598	0.017301	0.021892	0.036155	0.060619
542	0.000379	0.000532	0.003776	0.006765	0.006989	0.010957	0.012484	0.017344	0.021846	0.036183	0.060888
541	0.000298	0.00045	0.003688	0.006586	0.006859	0.010865	0.012403	0.017318	0.021922	0.03619	0.061022
540	0.00021	0.000304	0.003637	0.006678	0.006969	0.010917	0.01243	0.017308	0.021963	0.03613	0.061197

EXCLI Journal 2020;19:48-70 – ISSN 1611-2156
Received: October 03, 2019, accepted: December 19, 2019, published: January 03, 2020

Wavelength (nm)	Absorption intensity										
	concentration of β-hydroxyisovalerylshikonin (M)										
	A	*B*	*C*	*D*	*E*	*F*	*G*	*I*	*J*	*K*	*L*
	0.00	$8.0x10^{-7}$	$1.6x10^{-6}$	$2.0x10^{-6}$	$2.8x10^{-6}$	$4.0x10^{-6}$	$4.8x10^{-6}$	$6.0x10^{-6}$	$8.0x10^{-6}$	$1.2x10^{-5}$	$1.6x10^{-5}$
539	1.44E-05	0.000274	0.003491	0.006561	0.006794	0.010879	0.012485	0.017184	0.021905	0.036337	0.061311
538	-2.89E-05	0.000267	0.003484	0.006537	0.006764	0.010781	0.012373	0.017238	0.022036	0.036485	0.0615
537	-0.00015	5.78E-05	0.003411	0.006438	0.006735	0.010798	0.012275	0.017256	0.021815	0.036568	0.061738
536	-0.00015	3.62E-05	0.00333	0.006394	0.006708	0.010738	0.012324	0.017235	0.021926	0.03659	0.06194
535	-0.00023	7.27E-06	0.003198	0.006302	0.006576	0.010657	0.012308	0.017275	0.021952	0.036596	0.062109
534	-0.00036	-0.00012	0.003271	0.006269	0.006559	0.010644	0.012329	0.017084	0.02202	0.036625	0.062396
533	-0.00032	-0.00031	0.003079	0.006216	0.006582	0.010785	0.012161	0.017238	0.021946	0.036658	0.062408
532	-0.00028	-0.00014	0.002988	0.006224	0.006464	0.010525	0.012272	0.017178	0.021948	0.036935	0.062693
531	-0.00037	-0.00046	0.002984	0.006297	0.006364	0.010513	0.01221	0.017118	0.022065	0.036911	0.062781
530	-0.00043	-0.00046	0.002842	0.006158	0.006375	0.01054	0.012207	0.017319	0.021956	0.036931	0.063047
529	-0.00054	-0.00047	0.002803	0.006082	0.006372	0.0104	0.012256	0.017257	0.022042	0.037068	0.063321
528	-0.00062	-0.00046	0.002987	0.006105	0.006304	0.010451	0.012198	0.017204	0.022081	0.037245	0.063479
527	-0.00067	-0.00057	0.00281	0.005935	0.006358	0.010507	0.012238	0.017195	0.021956	0.037308	0.063485
526	-0.00076	-0.00059	0.002853	0.006045	0.006432	0.010502	0.012216	0.017409	0.022206	0.037266	0.063676
525	-0.00076	-0.00059	0.002714	0.006039	0.006235	0.01034	0.012285	0.017151	0.022146	0.037442	0.063928
524	-0.0007	-0.00065	0.002748	0.005994	0.006438	0.010566	0.012287	0.01733	0.022073	0.037424	0.064157
523	-0.00073	-0.00062	0.002805	0.005881	0.006199	0.010488	0.012244	0.017389	0.02224	0.03755	0.064197
522	-0.00074	-0.00073	0.002861	0.00604	0.006342	0.010373	0.012379	0.017293	0.02221	0.037555	0.064355
521	-0.00089	-0.00071	0.002658	0.006113	0.006337	0.01052	0.012523	0.017271	0.022376	0.037736	0.064592
520	-0.00077	-0.00069	0.002632	0.005964	0.006365	0.010705	0.012355	0.017449	0.022344	0.037771	0.064737
519	-0.0008	-0.00085	0.002809	0.005994	0.006431	0.010482	0.012246	0.017464	0.022384	0.037881	0.064929
518	-0.00074	-0.00069	0.002681	0.005975	0.006423	0.010578	0.012527	0.017519	0.022355	0.037952	0.065047
517	-0.00072	-0.00075	0.002707	0.005952	0.006332	0.010601	0.0124	0.017634	0.022411	0.037961	0.065412
516	-0.00085	-0.00071	0.002725	0.006036	0.006325	0.010612	0.012451	0.017542	0.022645	0.038262	0.065331
515	-0.00078	-0.00078	0.002682	0.006256	0.006508	0.010654	0.012448	0.017599	0.022603	0.03831	0.065719
514	-0.00081	-0.00064	0.002621	0.006087	0.006355	0.010729	0.012509	0.017704	0.022615	0.038237	0.065775
513	-0.00084	-0.00072	0.002788	0.006126	0.006435	0.01065	0.01248	0.017782	0.022651	0.038502	0.065706
512	-0.00067	-0.0008	0.002715	0.00617	0.006493	0.010784	0.01266	0.017757	0.022695	0.038501	0.066047
511	-0.00079	-0.0008	0.002638	0.006235	0.006368	0.010753	0.01275	0.01778	0.022736	0.038614	0.066346
510	-0.00079	-0.0007	0.002916	0.006377	0.006596	0.010823	0.012737	0.017857	0.022854	0.038658	0.066351
509	-0.00068	-0.00064	0.002774	0.006217	0.006547	0.010884	0.01291	0.017851	0.022743	0.03884	0.066691
508	-0.00063	-0.00069	0.002842	0.006281	0.006474	0.010858	0.012893	0.017947	0.022926	0.038809	0.066696
507	-0.00065	-0.00073	0.00289	0.00638	0.006537	0.010836	0.012822	0.017933	0.023023	0.03909	0.066697

Absorption intensity											
**Wavelength (nm)**	**concentration of β-hydroxyisovalerylshikonin (M)**										
	A	*B*	*C*	*D*	*E*	*F*	*G*	*I*	*J*	*K*	*L*
	0.00	$8.0x10^{-7}$	$1.6x10^{-6}$	$2.0x10^{-6}$	$2.8x10^{-6}$	$4.0x10^{-6}$	$4.8x10^{-6}$	$6.0x10^{-6}$	$8.0x10^{-6}$	$1.2x10^{-5}$	$1.6x10^{-5}$
506	-0.00073	-0.00082	0.00285	0.006307	0.006652	0.010948	0.012876	0.018005	0.023005	0.039059	0.066962
505	-0.00075	-0.00065	0.002835	0.006432	0.006559	0.010901	0.012786	0.018166	0.022963	0.038948	0.067266
504	-0.00076	-0.00062	0.002815	0.00641	0.006699	0.01106	0.013007	0.018104	0.023077	0.039501	0.067214
503	-0.0006	-0.00074	0.00291	0.006318	0.006674	0.010922	0.01316	0.018131	0.023418	0.039222	0.067302
502	-0.00062	-0.00057	0.002802	0.006458	0.006736	0.011123	0.013069	0.018146	0.023314	0.039215	0.067585
501	-0.00069	-0.00075	0.002885	0.006399	0.006735	0.011039	0.013195	0.018138	0.023204	0.03935	0.067645
500	-0.00047	-0.00071	0.002872	0.006336	0.006685	0.010914	0.013089	0.018326	0.023355	0.039566	0.067754
499	-0.00069	-0.00066	0.002813	0.006503	0.006668	0.011033	0.013155	0.018405	0.023293	0.039487	0.068018
498	-0.00047	-0.00062	0.0029	0.006501	0.006695	0.011126	0.013222	0.018581	0.023384	0.039647	0.067957
497	-0.00068	-0.00063	0.003002	0.006505	0.006701	0.01111	0.013122	0.018277	0.02349	0.03963	0.068109
496	-0.0006	-0.00061	0.002853	0.006609	0.00671	0.011142	0.013194	0.018387	0.023374	0.039752	0.068276
495	-0.00064	-0.00062	0.002978	0.00646	0.006726	0.011251	0.013158	0.018501	0.023462	0.039759	0.068311
494	-0.00062	-0.00064	0.003058	0.006587	0.006803	0.01108	0.01324	0.018451	0.023482	0.03983	0.068395
493	-0.00043	-0.00056	0.002944	0.006675	0.006697	0.011172	0.013154	0.018513	0.023519	0.039823	0.068534
492	-0.00054	-0.00061	0.002844	0.006565	0.006808	0.011316	0.013234	0.018491	0.023507	0.040017	0.068605
491	-0.00048	-0.00072	0.002914	0.006583	0.00671	0.011208	0.013243	0.018597	0.023559	0.040075	0.068751
490	-0.00043	-0.00064	0.002882	0.0065	0.006906	0.01123	0.013398	0.01852	0.023591	0.039834	0.068761
489	-0.0005	-0.00067	0.002819	0.00669	0.006845	0.011298	0.01322	0.018431	0.023553	0.03992	0.068734
488	-0.00036	-0.00071	0.002835	0.006553	0.006831	0.011224	0.013411	0.018469	0.02355	0.039967	0.068756
487	-0.00055	-0.00065	0.002845	0.006545	0.006844	0.011065	0.013408	0.018496	0.023368	0.039729	0.068574
486	-0.00045	-0.00075	0.002918	0.006486	0.006643	0.011121	0.013331	0.018484	0.023478	0.039908	0.068734
485	-0.00063	-0.00065	0.002809	0.006523	0.006661	0.011008	0.013164	0.018333	0.023347	0.039889	0.068449
484	-0.00063	-0.00083	0.002724	0.006541	0.006742	0.011044	0.013121	0.018344	0.023455	0.039748	0.068634
483	-0.00063	-0.00076	0.002594	0.006453	0.006503	0.010885	0.013111	0.018202	0.0232	0.039652	0.068341
482	-0.00069	-0.00092	0.002523	0.006284	0.006427	0.010842	0.013103	0.018198	0.023221	0.039616	0.068438
481	-0.00085	-0.00096	0.002416	0.006333	0.006586	0.010764	0.013076	0.018043	0.023	0.03945	0.068299
480	-0.00077	-0.00113	0.002494	0.00621	0.006338	0.010722	0.012843	0.018096	0.022895	0.039334	0.068186
479	-0.00089	-0.00111	0.002416	0.006153	0.006184	0.010498	0.01279	0.017809	0.022774	0.039229	0.067857
478	-0.00099	-0.00135	0.002252	0.00601	0.006087	0.010508	0.012659	0.017756	0.022635	0.038993	0.067892
477	-0.00101	-0.00146	0.002076	0.005981	0.005959	0.010202	0.012482	0.017503	0.022455	0.038775	0.067557
476	-0.00122	-0.00155	0.00195	0.00564	0.005895	0.009963	0.012283	0.017426	0.022538	0.038717	0.067248
475	-0.00132	-0.00167	0.001819	0.005629	0.005743	0.010053	0.012207	0.017294	0.02199	0.038438	0.067106
474	-0.00148	-0.00171	0.00162	0.00552	0.005602	0.009815	0.012061	0.017077	0.021932	0.038219	0.06692

Absorption intensity											
**Wavelength (nm)**	**concentration of β-hydroxyisovalerylshikonin (M)**										
	A	*B*	*C*	*D*	*E*	*F*	*G*	*I*	*J*	*K*	*L*
	0.00	8.0x10^{-7}	1.6x10^{-6}	2.0x10^{-6}	2.8x10^{-6}	4.0x10^{-6}	4.8x10^{-6}	6.0x10^{-6}	8.0x10^{-6}	1.2x10^{-5}	1.6x10^{-5}
473	-0.00158	-0.00182	0.001605	0.00543	0.005505	0.009705	0.011925	0.017105	0.021877	0.038124	0.066689
472	-0.00156	-0.00204	0.001463	0.00533	0.00551	0.009646	0.011899	0.01671	0.021632	0.037712	0.066541
471	-0.00183	-0.00209	0.00131	0.005185	0.005311	0.009575	0.011652	0.01664	0.021479	0.037596	0.066351
470	-0.00187	-0.00221	0.001345	0.005159	0.005077	0.009239	0.011663	0.016661	0.021448	0.037548	0.065914
469	-0.00182	-0.00236	0.001101	0.005121	0.005093	0.009265	0.01142	0.016497	0.021168	0.037513	0.065604
468	-0.00187	-0.00238	0.001099	0.005026	0.004988	0.009273	0.011469	0.016457	0.021239	0.037284	0.065445
467	-0.00179	-0.00215	0.001089	0.004993	0.004981	0.009151	0.011436	0.016269	0.021079	0.037264	0.065161
466	-0.00197	-0.00232	0.00127	0.005054	0.005049	0.009122	0.011299	0.016255	0.020942	0.037069	0.064876
465	-0.00185	-0.00237	0.001017	0.005044	0.004999	0.009037	0.011454	0.01615	0.020853	0.03693	0.064772
464	-0.00173	-0.00222	0.001183	0.005045	0.004745	0.009139	0.0113	0.016136	0.020843	0.036515	0.064546
463	-0.0018	-0.00234	0.001122	0.004875	0.004789	0.008913	0.011235	0.016092	0.020678	0.036298	0.06444
462	-0.00181	-0.00239	0.00099	0.005022	0.004789	0.008944	0.011214	0.016184	0.020546	0.036304	0.064291
461	-0.00196	-0.00231	0.001091	0.004934	0.004789	0.008869	0.011179	0.015969	0.020274	0.036181	0.064053
460	-0.00158	-0.00218	0.001032	0.005006	0.00494	0.008984	0.011107	0.015639	0.02018	0.036156	0.063965
459	-0.00162	-0.00235	0.001085	0.004942	0.004723	0.008607	0.010834	0.015627	0.020188	0.035922	0.063776
458	-0.00156	-0.00222	0.001155	0.00464	0.004462	0.008615	0.010988	0.015524	0.02002	0.035725	0.063703
457	-0.00154	-0.00222	0.001033	0.00486	0.004652	0.008693	0.011	0.015624	0.020196	0.036007	0.063407
456	-0.00154	-0.00234	0.001011	0.004953	0.004808	0.008863	0.011253	0.015807	0.02021	0.035939	0.06361
455	-0.00147	-0.00234	0.000978	0.004956	0.004799	0.008806	0.010917	0.015855	0.020094	0.035944	0.063287
454	-0.00154	-0.00216	0.001112	0.004995	0.004666	0.008822	0.010931	0.015686	0.020138	0.035556	0.063072
453	-0.00147	-0.00232	0.00108	0.004891	0.004792	0.008608	0.010919	0.015492	0.020122	0.035678	0.062905
452	-0.00146	-0.00226	0.001013	0.004948	0.004567	0.00874	0.010823	0.015668	0.019942	0.03547	0.062527
451	-0.00157	-0.00237	0.000925	0.004829	0.004713	0.008686	0.010999	0.01532	0.019789	0.035483	0.062336
450	-0.00155	-0.0022	0.000953	0.004876	0.004543	0.008669	0.010879	0.01536	0.019588	0.035245	0.062299
449	-0.00152	-0.00233	0.00095	0.004949	0.004451	0.00863	0.010761	0.01511	0.019623	0.035071	0.062025
448	-0.00139	-0.0022	0.000925	0.004875	0.004473	0.008315	0.010753	0.015219	0.019411	0.03498	0.061729
447	-0.00151	-0.00245	0.000711	0.004807	0.004333	0.008273	0.010734	0.015178	0.019455	0.034671	0.061482
446	-0.00147	-0.00237	0.000761	0.004865	0.004319	0.008471	0.010641	0.015037	0.019276	0.034652	0.061321
445	-0.00142	-0.00237	0.000767	0.004884	0.00439	0.008283	0.010512	0.014929	0.019179	0.03428	0.060979
444	-0.00153	-0.00247	0.00075	0.004807	0.004163	0.008324	0.010641	0.01487	0.018997	0.034422	0.060946
443	-0.00155	-0.00236	0.000654	0.004928	0.004197	0.008122	0.010436	0.014984	0.019005	0.034214	0.060471
442	-0.00126	-0.00246	0.000832	0.004744	0.004264	0.008158	0.010448	0.014772	0.019034	0.034166	0.060317
441	-0.00142	-0.00253	0.000746	0.004835	0.004266	0.008076	0.010305	0.014698	0.018733	0.034016	0.060165

Absorption intensity											
**Wavelength (nm)**	**concentration of β-hydroxyisovalerylshikonin (M)**										
	A	*B*	*C*	*D*	*E*	*F*	*G*	*I*	*J*	*K*	*L*
	0.00	$8.0x10^{-7}$	$1.6x10^{-6}$	$2.0x10^{-6}$	$2.8x10^{-6}$	$4.0x10^{-6}$	$4.8x10^{-6}$	$6.0x10^{-6}$	$8.0x10^{-6}$	$1.2x10^{-5}$	$1.6x10^{-5}$
440	-0.00142	-0.00236	0.000716	0.004881	0.004098	0.008219	0.010371	0.014879	0.018693	0.033881	0.060089
439	-0.00143	-0.00229	0.000816	0.00468	0.004153	0.008353	0.010421	0.014608	0.018756	0.033898	0.059882
438	-0.00106	-0.00209	0.000808	0.004985	0.004267	0.008207	0.010579	0.014833	0.018756	0.033931	0.05983
437	-0.00116	-0.00239	0.000846	0.004945	0.004274	0.008147	0.0105	0.014698	0.01864	0.033593	0.059435
436	-0.0012	-0.00241	0.000779	0.004698	0.004079	0.00788	0.010357	0.014561	0.018454	0.033348	0.059182
435	-0.00164	-0.00273	0.000545	0.004441	0.00396	0.007673	0.010176	0.014186	0.01835	0.033056	0.058922
434	-0.00129	-0.00277	0.000428	0.004421	0.003866	0.007832	0.010038	0.014152	0.018129	0.033093	0.058657
433	-0.00141	-0.00271	0.000482	0.004459	0.003773	0.007599	0.009962	0.014301	0.018182	0.033035	0.058625
432	-0.0014	-0.0027	0.000462	0.004592	0.003649	0.007589	0.009966	0.014071	0.01798	0.032776	0.058329
431	-0.00159	-0.00269	0.000253	0.004472	0.003452	0.007542	0.009563	0.014124	0.018011	0.032667	0.058141
430	-0.00148	-0.00281	0.000371	0.004319	0.00362	0.00749	0.009784	0.013906	0.017807	0.032555	0.058074
429	-0.00161	-0.00283	7.78E-05	0.004338	0.00355	0.007291	0.009751	0.013883	0.017708	0.032594	0.057868
428	-0.0016	-0.00289	0.000118	0.004324	0.003501	0.007476	0.009642	0.013717	0.017663	0.03248	0.057607
427	-0.0016	-0.00285	0.000133	0.004225	0.003259	0.007314	0.009567	0.013579	0.017436	0.032441	0.057549
426	-0.00171	-0.00306	0.000148	0.004353	0.003424	0.007331	0.009646	0.013697	0.01754	0.032225	0.057193
425	-0.00159	-0.00316	-1.37E-05	0.004021	0.003423	0.007196	0.009422	0.013486	0.017289	0.032138	0.057193
424	-0.00152	-0.00312	-8.35E-05	0.004041	0.003252	0.007163	0.009353	0.013508	0.017272	0.031947	0.056957
423	-0.0018	-0.00342	-2.83E-05	0.004053	0.003208	0.00707	0.009214	0.01335	0.017111	0.0319	0.056916
422	-0.00178	-0.00331	-0.00035	0.004121	0.003146	0.007074	0.009339	0.01334	0.017049	0.031817	0.05671
421	-0.00192	-0.00342	-0.00015	0.003932	0.003057	0.006925	0.009378	0.013421	0.017054	0.031812	0.056583
420	-0.00192	-0.00323	-0.00037	0.003869	0.002903	0.006852	0.009259	0.013191	0.016979	0.031733	0.056484
419	-0.00184	-0.0036	-0.00024	0.003946	0.002962	0.006759	0.009286	0.013033	0.01675	0.031744	0.056111
418	-0.00177	-0.00336	-0.00029	0.003898	0.003106	0.006954	0.009174	0.013251	0.016995	0.031658	0.056201
417	-0.00191	-0.00327	-0.00025	0.003473	0.002469	0.006455	0.008909	0.012866	0.01667	0.031187	0.055861
416	-0.00214	-0.00376	-0.00063	0.003699	0.002814	0.006426	0.009012	0.01277	0.016525	0.031208	0.055711
415	-0.00213	-0.0038	-0.00054	0.003672	0.002397	0.00638	0.008828	0.012811	0.016575	0.031029	0.055872
414	-0.00215	-0.00383	-0.00061	0.003594	0.002693	0.006496	0.009005	0.012679	0.01632	0.031024	0.055616
413	-0.00197	-0.00373	-0.00068	0.00372	0.002398	0.006485	0.008854	0.012675	0.016317	0.031206	0.055635
412	-0.00217	-0.0037	-0.00059	0.003635	0.002497	0.006443	0.00887	0.012635	0.016284	0.031113	0.055665
411	-0.00201	-0.0038	-0.00057	0.003577	0.002453	0.006257	0.008976	0.012632	0.016485	0.030878	0.055626
410	-0.00198	-0.00382	-0.00065	0.003669	0.002504	0.006444	0.008875	0.012731	0.016461	0.031025	0.055484
409	-0.00212	-0.00386	-0.00055	0.00358	0.002612	0.006351	0.008702	0.012426	0.016179	0.030917	0.055578
408	-0.00206	-0.0038	-0.00067	0.003672	0.002403	0.006324	0.00894	0.01266	0.016353	0.03085	0.055671

EXCLI Journal 2020;19:48-70 – ISSN 1611-2156
Received: October 03, 2019, accepted: December 19, 2019, published: January 03, 2020

Absorption intensity											
**Wavelength (nm)**	**concentration of β-hydroxyisovalerylshikonin (M)**										
	A	*B*	*C*	*D*	*E*	*F*	*G*	*I*	*J*	*K*	*L*
	0.00	$8.0x10^{-7}$	$1.6x10^{-6}$	$2.0x10^{-6}$	$2.8x10^{-6}$	$4.0x10^{-6}$	$4.8x10^{-6}$	$6.0x10^{-6}$	$8.0x10^{-6}$	$1.2x10^{-5}$	$1.6x10^{-5}$
407	-0.00199	-0.00388	-0.00064	0.003604	0.002369	0.006342	0.008835	0.012662	0.016332	0.030953	0.055352
406	-0.00197	-0.00377	-0.00071	0.003698	0.002411	0.006276	0.008774	0.012565	0.016236	0.030851	0.055365
405	-0.00206	-0.00377	-0.00085	0.003662	0.002222	0.006334	0.008753	0.012274	0.016137	0.03088	0.055327
404	-0.00205	-0.00389	-0.00066	0.003614	0.002446	0.006258	0.008792	0.012684	0.016061	0.030799	0.055367
403	-0.002	-0.00386	-0.00077	0.003561	0.002214	0.006313	0.008688	0.012421	0.015875	0.030444	0.055406
402	-0.00199	-0.00413	-0.00086	0.003295	0.00219	0.005937	0.00848	0.011998	0.015816	0.030383	0.055385
401	-0.00215	-0.00382	-0.0009	0.003611	0.002362	0.006063	0.008431	0.012419	0.016126	0.030867	0.055691
400	-0.00213	-0.00402	-0.00096	0.003539	0.002256	0.006035	0.008791	0.012327	0.01589	0.030836	0.055759
399	-0.00215	-0.00411	-0.00094	0.003488	0.002003	0.005982	0.008636	0.012349	0.01601	0.030743	0.056088
398	-0.0021	-0.00409	-0.00101	0.00334	0.002173	0.005903	0.008356	0.012181	0.016075	0.03093	0.055954
397	-0.00232	-0.00419	-0.00111	0.003481	0.00209	0.00589	0.008382	0.012338	0.015827	0.030989	0.05615
396	-0.00226	-0.00424	-0.00112	0.003362	0.002012	0.005893	0.008459	0.012339	0.016005	0.031024	0.056285
395	-0.00238	-0.00414	-0.00106	0.003364	0.002006	0.005736	0.008327	0.012086	0.01611	0.030892	0.056596
394	-0.00234	-0.0042	-0.00138	0.00321	0.001997	0.005774	0.008388	0.012239	0.015738	0.03113	0.05679
393	-0.00244	-0.00424	-0.00132	0.003301	0.001831	0.005814	0.008468	0.012345	0.015979	0.031002	0.05694
392	-0.00231	-0.00466	-0.00123	0.003214	0.00198	0.005834	0.008308	0.012316	0.015863	0.031192	0.057019
391	-0.00239	-0.00462	-0.00145	0.003098	0.001731	0.005733	0.008201	0.012033	0.015777	0.031115	0.057176
390	-0.00247	-0.00485	-0.00162	0.003042	0.001711	0.005678	0.008486	0.012195	0.015982	0.031375	0.05756
389	-0.00253	-0.00446	-0.00156	0.003079	0.00183	0.00567	0.008205	0.011958	0.01585	0.031168	0.057811
388	-0.00232	-0.00425	-0.00108	0.00355	0.001964	0.006013	0.008681	0.01264	0.016216	0.031691	0.057835
387	-0.00301	-0.00495	-0.00197	0.0028	0.001349	0.005298	0.008089	0.012007	0.015661	0.031206	0.057653
386	-0.00299	-0.00507	-0.00179	0.002621	0.001509	0.005244	0.008084	0.011874	0.01564	0.031117	0.05816
385	-0.00295	-0.00496	-0.00191	0.002743	0.001288	0.005155	0.007972	0.011674	0.015593	0.03149	0.058389
384	-0.00295	-0.00517	-0.00191	0.002779	0.001243	0.005458	0.008078	0.011858	0.015658	0.031487	0.058277
383	-0.00332	-0.00526	-0.00198	0.00266	0.000925	0.005394	0.00809	0.011814	0.01578	0.031258	0.058504
382	-0.00329	-0.00516	-0.00231	0.002458	0.000884	0.004863	0.007795	0.011554	0.0155	0.031057	0.059023
381	-0.00319	-0.00555	-0.0024	0.002281	0.000872	0.005098	0.007818	0.011732	0.015523	0.031582	0.059475
380	-0.00314	-0.0057	-0.0026	0.002418	0.001039	0.004823	0.007994	0.011687	0.015267	0.031576	0.059314
379	-0.00357	-0.00556	-0.00257	0.002287	0.000803	0.004936	0.007698	0.011506	0.015353	0.031818	0.059951
378	-0.00321	-0.00588	-0.00286	0.002181	0.000637	0.004644	0.007795	0.01153	0.015283	0.03183	0.059786
377	-0.00381	-0.00578	-0.00292	0.002118	0.000614	0.004603	0.007581	0.011382	0.015452	0.031948	0.060055
376	-0.00354	-0.00614	-0.0028	0.002156	0.000435	0.004556	0.007588	0.011564	0.015373	0.032195	0.060648
375	-0.00344	-0.00583	-0.0027	0.002369	0.000931	0.004965	0.007954	0.011681	0.015566	0.032241	0.060847

EXCLI Journal 2020;19:48-70 – ISSN 1611-2156
Received: October 03, 2019, accepted: December 19, 2019, published: January 03, 2020

Absorption intensity											
**Wavelength (nm)**	**concentration of β-hydroxyisovalerylshikonin (M)**										
	A	*B*	*C*	*D*	*E*	*F*	*G*	*I*	*J*	*K*	*L*
	0.00	$8.0x10^{-7}$	$1.6x10^{-6}$	$2.0x10^{-6}$	$2.8x10^{-6}$	$4.0x10^{-6}$	$4.8x10^{-6}$	$6.0x10^{-6}$	$8.0x10^{-6}$	$1.2x10^{-5}$	$1.6x10^{-5}$
374	-0.00429	-0.00661	-0.00339	0.001421	-0.00011	0.004402	0.006896	0.011179	0.015296	0.031595	0.060747
373	-0.00444	-0.00677	-0.00354	0.001495	-8.16E-05	0.004021	0.007158	0.010979	0.014975	0.031724	0.06057
372	-0.00431	-0.00676	-0.00342	0.001066	-0.00026	0.003856	0.006958	0.010808	0.014537	0.031479	0.061145
371	-0.0048	-0.00707	-0.00361	0.001006	-0.00035	0.003653	0.006497	0.010695	0.014659	0.031631	0.061222
370	-0.00508	-0.00769	-0.00423	0.001218	-0.00035	0.003463	0.006847	0.010494	0.014498	0.031626	0.061291
369	-0.00522	-0.00737	-0.00429	0.000937	-0.00061	0.003734	0.006886	0.010895	0.014454	0.031281	0.062021
368	0.001056	-0.00171	0.00159	0.006613	0.005069	0.009319	0.012955	0.016425	0.020988	0.03818	0.067553
367	-0.00447	-0.00782	-0.00406	0.00086	-0.0006	0.003798	0.006992	0.010926	0.015037	0.032417	0.062873
366	-0.00532	-0.00774	-0.00444	0.00066	-0.00095	0.003312	0.00664	0.010631	0.01526	0.03207	0.0626
365	-0.00547	-0.0077	-0.00434	0.000413	-0.00108	0.003367	0.006845	0.010902	0.014999	0.032355	0.063021
364	-0.00568	-0.00811	-0.0047	0.000325	-0.00093	0.002927	0.006847	0.010806	0.014814	0.032989	0.063858
363	-0.00516	-0.00806	-0.00475	0.000307	-0.00132	0.003584	0.006314	0.011015	0.014582	0.032583	0.063687
362	-0.00625	-0.00867	-0.00502	0.000336	-0.00128	0.002598	0.006121	0.010276	0.014291	0.03297	0.06384
361	-0.00648	-0.00848	-0.00512	-9.65E-05	-0.00135	0.002446	0.006288	0.011301	0.016198	0.033208	0.065938
360	-0.00393	-0.0072	-0.00353	0.001651	0.000275	0.004436	0.007136	0.010171	0.014232	0.032266	0.063993
359	-0.00705	-0.01016	-0.00638	-0.00128	-0.00207	0.000988	0.005305	0.009424	0.013257	0.032667	0.064154
358	-0.00773	-0.01042	-0.0068	-0.00112	-0.00296	0.002376	0.005504	0.009466	0.014511	0.032199	0.064248
357	-0.00759	-0.01057	-0.00695	-0.00208	-0.0031	0.002066	0.00492	0.0096	0.013973	0.032796	0.064801
356	-0.00855	-0.01088	-0.00675	-0.00176	-0.00307	0.000798	0.004624	0.009041	0.015257	0.033582	0.066106
355	-0.00628	-0.00838	-0.00472	-0.00063	-0.00094	0.003087	0.00697	0.011775	0.012953	0.031793	0.063846
354	-0.00824	-0.01127	-0.00836	-0.00247	-0.00417	0.001206	0.003956	0.009172	0.012721	0.032633	0.064908
353	-0.00868	-0.01111	-0.00815	-0.00196	-0.00503	0.001441	0.004645	0.008385	0.012802	0.033461	0.065452
352	-0.01016	-0.01194	-0.0078	-0.00284	-0.00454	0.000695	0.004614	0.007355	0.013677	0.03187	0.067862
351	-0.00646	-0.00811	-0.00634	-0.00039	-0.00243	0.003615	0.00671	0.012183	0.015108	0.034835	0.064515
350	-0.00946	-0.01397	-0.0085	-0.00471	-0.00536	-0.00111	0.003852	0.008126	0.013505	0.031577	0.066754
349	-0.00971	-0.01338	-0.0089	-0.00359	-0.00611	0.000744	0.004283	0.007109	0.011898	0.031811	0.068559
348	0.003445	-0.00013	0.004073	0.009615	0.007775	0.012453	0.016843	0.021895	0.026464	0.046954	0.081992
347	0.004035	0.00073	0.004392	0.010362	0.008611	0.014424	0.017569	0.023371	0.027313	0.04826	0.082905
346	0.004434	0.001062	0.004823	0.010686	0.00927	0.013643	0.018557	0.023398	0.027701	0.048764	0.084342
345	0.004098	0.000942	0.004624	0.010908	0.009158	0.014117	0.018102	0.023349	0.028931	0.049658	0.084985
344	0.004512	0.000967	0.00487	0.011301	0.010139	0.014304	0.019253	0.024234	0.028914	0.049554	0.085525
343	0.004211	0.001522	0.005562	0.011139	0.009754	0.014631	0.019793	0.025082	0.030038	0.050926	0.086905
342	0.005168	0.001517	0.005475	0.011799	0.010404	0.015611	0.02007	0.025699	0.030535	0.05205	0.08806

EXCLI Journal 2020;19:48-70 – ISSN 1611-2156
Received: October 03, 2019, accepted: December 19, 2019, published: January 03, 2020

Absorption intensity											
**Wavelength (nm)**	**concentration of β-hydroxyisovalerylshikonin (M)**										
	A	*B*	*C*	*D*	*E*	*F*	*G*	*I*	*J*	*K*	*L*
	0.00	$8.0x10^{-7}$	$1.6x10^{-6}$	$2.0x10^{-6}$	$2.8x10^{-6}$	$4.0x10^{-6}$	$4.8x10^{-6}$	$6.0x10^{-6}$	$8.0x10^{-6}$	$1.2x10^{-5}$	$1.6x10^{-5}$
341	0.005361	0.001875	0.005973	0.012445	0.010497	0.015246	0.020064	0.025781	0.031157	0.052878	0.090077
340	0.005676	0.002173	0.00605	0.011731	0.010141	0.016144	0.02085	0.026262	0.031301	0.053475	0.09105
339	0.005313	0.002187	0.006498	0.01228	0.011301	0.016446	0.021569	0.026445	0.03173	0.054035	0.092302
338	0.005528	0.002548	0.006582	0.012552	0.011709	0.016367	0.021407	0.026616	0.032719	0.054967	0.093567
337	0.005755	0.002239	0.00685	0.013034	0.012122	0.016838	0.021803	0.027535	0.03315	0.055495	0.095395
336	0.005978	0.002456	0.007035	0.013252	0.012567	0.017261	0.022285	0.028445	0.034132	0.056801	0.096419
335	0.006011	0.003001	0.007217	0.01347	0.012401	0.017752	0.022772	0.028789	0.034284	0.057548	0.097438
334	0.006118	0.002999	0.007514	0.013911	0.012604	0.018059	0.022891	0.028884	0.034697	0.058082	0.099051
333	0.006263	0.003347	0.007596	0.014127	0.013024	0.018169	0.023744	0.02981	0.035539	0.05917	0.099786
332	0.007126	0.003558	0.008025	0.014379	0.013778	0.01889	0.024246	0.030239	0.036284	0.06022	0.101782
331	0.007389	0.003698	0.008739	0.014256	0.013795	0.019175	0.024365	0.030594	0.036968	0.060878	0.102602
330	0.007585	0.004048	0.00808	0.014849	0.014244	0.019956	0.024354	0.031175	0.036806	0.061847	0.103995
329	0.007588	0.003674	0.008274	0.015031	0.013745	0.020383	0.025	0.031834	0.038113	0.062703	0.10547
328	0.007924	0.004282	0.008634	0.015494	0.014328	0.020362	0.025764	0.032619	0.038453	0.063395	0.107267
327	0.008076	0.004417	0.008822	0.015836	0.015251	0.020585	0.026371	0.032787	0.039036	0.064521	0.108463
326	0.00814	0.004482	0.009099	0.016335	0.015289	0.021391	0.026481	0.034018	0.040063	0.065646	0.110143
325	0.00813	0.004661	0.009284	0.016357	0.015759	0.021548	0.027321	0.034568	0.040648	0.067031	0.111924
324	0.008541	0.00453	0.009889	0.016643	0.016307	0.022281	0.027851	0.035024	0.041815	0.068078	0.113363
323	0.008581	0.005025	0.009875	0.016675	0.017062	0.022519	0.028342	0.035381	0.04245	0.068976	0.115034
322	0.008557	0.005019	0.009855	0.017302	0.01728	0.022715	0.028478	0.036149	0.042886	0.070171	0.117016
321	0.009016	0.005221	0.010379	0.01755	0.017131	0.023372	0.028987	0.036529	0.04415	0.071892	0.11874
320	0.008936	0.005775	0.010321	0.017867	0.018111	0.024222	0.029777	0.037472	0.045029	0.073168	0.12045
319	0.009681	0.005696	0.010811	0.018155	0.018228	0.024336	0.030591	0.038037	0.045428	0.073515	0.122664
318	0.009676	0.00551	0.011144	0.018728	0.018432	0.024624	0.03113	0.0387	0.04623	0.075297	0.124325
317	0.010113	0.006233	0.011265	0.018973	0.019189	0.025406	0.031513	0.039683	0.04721	0.076716	0.126958
316	0.010088	0.006308	0.011512	0.019091	0.019151	0.025941	0.032398	0.040236	0.047794	0.077455	0.12861
315	0.010046	0.006625	0.011891	0.019812	0.01961	0.026252	0.032946	0.041332	0.049168	0.07877	0.130895
314	0.010516	0.006447	0.012157	0.019909	0.020355	0.027122	0.033295	0.042003	0.050358	0.08029	0.133052
313	0.011246	0.006804	0.012381	0.020152	0.020916	0.028024	0.034429	0.042583	0.051111	0.081959	0.135055
312	0.010762	0.007279	0.012946	0.020539	0.021187	0.028075	0.034923	0.043431	0.051557	0.083134	0.137454
311	0.011216	0.007484	0.013139	0.021502	0.02178	0.028547	0.03554	0.044626	0.052807	0.084731	0.139719
310	0.011621	0.007735	0.013747	0.021415	0.022148	0.029495	0.03663	0.044965	0.053751	0.086307	0.142452
309	0.012041	0.007532	0.014004	0.02207	0.022723	0.030435	0.037399	0.04629	0.054802	0.087658	0.14446

EXCLI Journal 2020;19:48-70 – ISSN 1611-2156
Received: October 03, 2019, accepted: December 19, 2019, published: January 03, 2020

Absorption intensity											
**Wavelength (nm)**	**concentration of β-hydroxyisovalerylshikonin (M)**										
	A	*B*	*C*	*D*	*E*	*F*	*G*	*I*	*J*	*K*	*L*
	0.00	$8.0x10^{-7}$	$1.6x10^{-6}$	$2.0x10^{-6}$	$2.8x10^{-6}$	$4.0x10^{-6}$	$4.8x10^{-6}$	$6.0x10^{-6}$	$8.0x10^{-6}$	$1.2x10^{-5}$	$1.6x10^{-5}$
308	0.011159	0.00756	0.013491	0.021438	0.022672	0.030089	0.037543	0.046121	0.055335	0.08848	0.146087
307	0.013013	0.00857	0.014743	0.02283	0.023936	0.031574	0.038887	0.047773	0.057132	0.090403	0.148953
306	0.013063	0.00896	0.015291	0.023533	0.024765	0.032207	0.04018	0.048917	0.058124	0.092047	0.151507
305	0.013386	0.0093	0.015784	0.024062	0.025567	0.033024	0.041149	0.049537	0.059327	0.093447	0.153772
304	0.01386	0.009861	0.016415	0.024969	0.026066	0.033452	0.042254	0.050724	0.060749	0.095179	0.15657
303	0.014122	0.010126	0.016733	0.024936	0.026446	0.034126	0.043207	0.051892	0.061468	0.09692	0.158441
302	0.01507	0.01063	0.017467	0.026189	0.027588	0.035281	0.044113	0.0525	0.062839	0.099167	0.161056
301	0.015884	0.011667	0.017739	0.026789	0.02822	0.036578	0.045384	0.054258	0.064602	0.100879	0.163585
300	0.016587	0.012284	0.018697	0.02812	0.029479	0.037085	0.046698	0.055397	0.065598	0.102653	0.167095
299	0.017574	0.012601	0.019789	0.029072	0.030414	0.038148	0.048119	0.056503	0.067441	0.104778	0.169288
298	0.018955	0.014134	0.020914	0.030041	0.031396	0.039651	0.049574	0.058146	0.069275	0.106315	0.172178
297	0.019861	0.01527	0.022022	0.031175	0.032805	0.041251	0.051094	0.059953	0.070665	0.108798	0.174857
296	0.021394	0.016588	0.023809	0.032758	0.034423	0.042529	0.05325	0.06194	0.072647	0.111585	0.178468
295	0.023129	0.018517	0.025474	0.034831	0.036216	0.044729	0.055873	0.064138	0.075257	0.114418	0.182248
294	0.025348	0.020394	0.027816	0.037411	0.039071	0.047558	0.058328	0.067054	0.078625	0.117899	0.186065
293	0.02831	0.023956	0.030942	0.040782	0.041964	0.050631	0.062161	0.070488	0.081978	0.121725	0.191087
292	0.03254	0.027383	0.035029	0.044706	0.045895	0.054877	0.065767	0.074529	0.086037	0.126697	0.196135
291	0.036454	0.032183	0.039251	0.049403	0.050858	0.058989	0.070853	0.079599	0.091329	0.13181	0.202685
290	0.041377	0.037077	0.044513	0.054381	0.055523	0.064235	0.076712	0.085252	0.097123	0.137884	0.20888
289	0.047669	0.042546	0.050369	0.06002	0.061456	0.07053	0.082877	0.091507	0.103349	0.144165	0.216295
288	0.053187	0.048828	0.056362	0.066381	0.067882	0.075938	0.089004	0.098365	0.110223	0.152009	0.223831
287	0.058988	0.054405	0.062025	0.072349	0.073488	0.081895	0.095031	0.104403	0.116572	0.158375	0.230752
286	0.063643	0.05942	0.067023	0.077457	0.078851	0.086828	0.10036	0.109678	0.12195	0.164061	0.237129
285	0.067443	0.062883	0.070521	0.081501	0.082576	0.090981	0.104558	0.114166	0.12696	0.168594	0.242303
284	0.070151	0.065217	0.073848	0.084208	0.085476	0.093647	0.107672	0.117143	0.129968	0.172374	0.246196
283	0.072352	0.067411	0.075277	0.086572	0.087869	0.095818	0.109981	0.119734	0.132594	0.175439	0.250021
282	0.074103	0.069198	0.077364	0.088531	0.089605	0.097829	0.112079	0.12176	0.134839	0.178246	0.253251
281	0.076124	0.0712	0.079435	0.090437	0.091307	0.099754	0.114393	0.123996	0.137203	0.181125	0.256508
280	0.077353	0.072866	0.081294	0.092364	0.09323	0.101809	0.116307	0.126227	0.139734	0.18358	0.259929
279	0.079213	0.074129	0.082635	0.094048	0.094867	0.103796	0.118145	0.128134	0.141757	0.186021	0.262628
278	0.079426	0.074353	0.082962	0.094853	0.095487	0.103958	0.118529	0.129099	0.142811	0.187594	0.2652
277	0.07978	0.074589	0.082842	0.094708	0.095157	0.104215	0.118984	0.129079	0.143023	0.188714	0.266638
276	0.078896	0.074061	0.082061	0.094148	0.094486	0.103531	0.118437	0.129125	0.142828	0.188808	0.267752

Received: October 03, 2019, accepted: December 19, 2019, published: January 03, 2020

Wavelength (nm)	***Absorption intensity*** concentration of β-hydroxyisovalerylshikonin (M)										
	A	*B*	*C*	*D*	*E*	*F*	*G*	*I*	*J*	*K*	*L*
	0.00	8.0x10^{-7}	1.6x10^{-6}	2.0x10^{-6}	2.8x10^{-6}	4.0x10^{-6}	4.8x10^{-6}	6.0x10^{-6}	8.0x10^{-6}	1.2x10^{-5}	1.6x10^{-5}
275	0.078103	0.07283	0.081413	0.093188	0.093674	0.102869	0.117152	0.128249	0.142544	0.188786	0.268369
274	0.076357	0.071377	0.080266	0.092113	0.092221	0.101542	0.116235	0.127005	0.141496	0.188504	0.268941
273	0.075088	0.070035	0.078704	0.090907	0.091033	0.100921	0.115358	0.126575	0.141116	0.188103	0.269842
272	0.073776	0.068343	0.077668	0.089573	0.089716	0.099618	0.114031	0.125267	0.140478	0.188161	0.270659
271	0.072517	0.067118	0.076207	0.088516	0.088765	0.099048	0.113861	0.12437	0.140214	0.188495	0.272003
270	0.071567	0.066153	0.075436	0.087986	0.088092	0.097977	0.11315	0.124694	0.140085	0.189251	0.273859
269	0.070374	0.065073	0.074398	0.087289	0.087282	0.097983	0.112947	0.124643	0.140301	0.190153	0.27648
268	0.069088	0.063545	0.073556	0.086228	0.086357	0.097331	0.112147	0.123907	0.140319	0.191161	0.279494
267	0.067614	0.061984	0.071985	0.084957	0.085022	0.096412	0.111355	0.123686	0.140157	0.191893	0.28183
266	0.065996	0.060748	0.070632	0.083708	0.084158	0.095503	0.11078	0.123317	0.140271	0.192993	0.285111
265	0.064569	0.059625	0.069783	0.08286	0.083305	0.094755	0.110368	0.123342	0.140623	0.194595	0.28896
264	0.063108	0.058021	0.06822	0.081816	0.082338	0.094224	0.109636	0.122962	0.141042	0.195806	0.292679
263	0.060879	0.056282	0.066781	0.080624	0.080759	0.093338	0.10865	0.122772	0.141059	0.197095	0.29664
262	0.059676	0.054663	0.065773	0.07968	0.080587	0.093031	0.108502	0.123015	0.142199	0.199381	0.301029
261	0.059254	0.054616	0.065364	0.079806	0.080507	0.093742	0.109377	0.124143	0.14387	0.202496	0.307318
260	0.058707	0.054151	0.065434	0.080061	0.081148	0.094286	0.110366	0.125516	0.145552	0.206312	0.313873
259	0.057665	0.05384	0.065151	0.079546	0.080994	0.094625	0.110932	0.126896	0.147476	0.210093	0.320972
258	0.056599	0.052634	0.06462	0.079538	0.08087	0.095055	0.111644	0.127794	0.149284	0.213925	0.327861
257	0.054712	0.051091	0.06334	0.078451	0.080049	0.09486	0.111447	0.128327	0.150453	0.216922	0.33555
256	0.052982	0.049701	0.062323	0.078032	0.079866	0.094757	0.111825	0.129451	0.152288	0.220774	0.343737
255	0.051665	0.048947	0.062097	0.078203	0.080618	0.095779	0.113559	0.131548	0.155398	0.226658	0.35402
254	0.051682	0.049167	0.062894	0.07957	0.081758	0.097776	0.115713	0.135141	0.160179	0.233734	0.366823
253	0.051424	0.049404	0.063696	0.080598	0.083794	0.100296	0.118595	0.13896	0.16529	0.241998	0.381391
252	0.051358	0.049703	0.06455	0.082111	0.085975	0.103317	0.12214	0.143499	0.170629	0.251479	0.397739
251	0.051103	0.05011	0.065599	0.083533	0.088023	0.106549	0.125647	0.148497	0.177663	0.262223	0.415891
250	0.050933	0.050801	0.067275	0.085771	0.091165	0.110577	0.130628	0.154793	0.185228	0.274573	0.436605
249	0.051807	0.052623	0.069607	0.089366	0.09554	0.116034	0.136833	0.162697	0.195367	0.289886	0.462065
248	0.053538	0.055467	0.073609	0.094199	0.101381	0.122886	0.145133	0.173369	0.207993	0.308938	0.492773
247	0.056134	0.059268	0.079009	0.100522	0.108872	0.131857	0.15495	0.186306	0.223573	0.331894	0.530635
246	0.059465	0.064122	0.085332	0.108536	0.11802	0.142957	0.167781	0.201635	0.242699	0.359985	0.575821
245	0.064214	0.070756	0.094084	0.118781	0.130476	0.157506	0.184121	0.221999	0.267333	0.39596	0.634334
244	0.070617	0.079772	0.105457	0.13244	0.146291	0.176109	0.205275	0.247795	0.298604	0.441244	0.707796
243	0.079587	0.091861	0.120904	0.150568	0.167579	0.200745	0.233202	0.282452	0.340042	0.500742	0.803198

Absorption intensity											
**Wavelength (nm)**	**concentration of β-hydroxyisovalerylshikonin (M)**										
	A	*B*	*C*	*D*	*E*	*F*	*G*	*I*	*J*	*K*	*L*
	0.00	8.0x10^{-7}	1.6x10^{-6}	2.0x10^{-6}	2.8x10^{-6}	4.0x10^{-6}	4.8x10^{-6}	6.0x10^{-6}	8.0x10^{-6}	1.2x10^{-5}	1.6x10^{-5}
242	0.091203	0.107723	0.140715	0.173776	0.195379	0.233125	0.269223	0.327016	0.393581	0.577616	0.927263
241	0.105919	0.127877	0.166723	0.205379	0.23189	0.27599	0.317377	0.38613	0.464916	0.680049	1.092075
240	0.124362	0.153763	0.200821	0.245912	0.280051	0.332527	0.380453	0.464277	0.559736	0.815555	1.30808
239	0.147943	0.187409	0.245518	0.299701	0.343728	0.408105	0.465403	0.569217	0.686965	0.996986	1.593158
238	0.175966	0.23034	0.303452	0.369666	0.427701	0.508158	0.577051	0.708495	0.855474	1.235388	1.964248
237	0.210726	0.284526	0.378722	0.461753	0.538188	0.640832	0.725792	0.893587	1.079771	1.550776	2.441839
236	0.250736	0.351798	0.47471	0.580524	0.68272	0.814774	0.92064	1.136626	1.373501	1.957994	3.037228
235	0.296245	0.435675	0.598065	0.734945	0.87055	1.042896	1.175949	1.452936	1.751489	2.476246	3.689135
234	0.34641	0.53731	0.754054	0.931192	1.113181	1.336912	1.503261	1.8584	2.237272	3.101458	4.211637
233	0.400977	0.663156	0.951644	1.182384	1.424067	1.712183	1.921131	2.371297	2.827652	3.727044	4.418468
232	0.458407	0.816804	1.200708	1.5006	1.814502	2.185271	2.440752	2.977165	3.467493	4.140378	4.618288
231	0.518579	1.006475	1.514215	1.897186	2.299029	2.755633	3.055715	3.592463	3.941182	4.363565	4.768505
230	0.579755	1.236233	1.903386	2.384194	2.880216	3.370362	3.660314	4.007058	4.190417	4.516444	4.935167
229	0.642466	1.521781	2.378363	2.958672	3.481263	3.877918	4.048216	4.213211	4.349309	4.709617	4.993515
228	0.705106	1.872324	2.940943	3.546832	3.924066	4.121677	4.252346	4.3819	4.521544	4.760846	5.15677
227	0.769445	2.291069	3.522372	3.941813	4.174541	4.26947	4.404029	4.548143	4.5977	4.978432	5.05667
226	0.83416	2.793114	3.959662	4.141654	4.32548	4.448871	4.527903	4.627132	4.727005	5.024828	5.129351
225	0.90126	3.351621	4.206594	4.301436	4.448612	4.534855	4.648296	4.791257	4.854442	5.146618	5.229
224	0.973402	3.852112	4.412467	4.379243	4.52844	4.647909	4.801356	4.854376	4.885903	5.047873	5.406775
223	1.051547	4.197108	4.504851	4.520628	4.611875	4.70505	4.910281	4.890537	5.073768	5.088323	5.200662
222	1.136822	4.329116	4.627005	4.578192	4.800073	4.874551	4.874448	4.883648	4.969826	5.241845	6.054725
221	1.231329	4.54499	4.763853	4.741445	4.787255	4.866499	5.113109	4.933943	5.167158	5.40518	5.301672
220	1.33416	4.699789	4.833317	4.715994	4.89765	4.92544	4.919572	5.463266	4.979779	5.407622	5.726341
219	1.44412	4.733372	5.026493	4.699203	4.807168	4.983457	5.082029	5.209542	5.155781	5.198052	5.374068
218	1.554616	4.837083	4.847066	4.749496	4.857216	5.063489	5.279571	5.117482	5.168734	5.469527	5.539996
217	1.671233	4.848315	5.043761	4.864097	4.864594	5.107591	5.097439	5.709404	5.137723	5.750297	6.148297
216	1.795374	4.951444	5.10577	4.819936	5.047483	5.137857	5.313357	5.449674	5.44944	5.614246	5.582137
215	1.928391	5.082222	4.93445	5.023119	4.95708	5.136527	6.005846	5.213367	5.750112	6.227246	5.590569
214	2.07113	4.909496	5.401292	4.915178	5.077572	5.449111	5.449013	5.533013	5.147263	5.474795	6.076788
213	2.219779	5.121798	5.330452	5.119906	10	5.029202	5.205253	6.350833	5.748578	5.873311	6.049362
212	2.370366	5.225776	5.322217	5.077501	5.446964	5.280438	5.207651	10	5.174799	10	5.299502
211	2.536632	5.248353	10	5.044268	5.247212	5.209533	10	5.227372	5.358142	5.74308	6.287613
210	2.716372	10	10	5.610232	5.498538	10	10	10	10	10	10

EXCLI Journal 2020;19:48-70 – ISSN 1611-2156
Received: October 03, 2019, accepted: December 19, 2019, published: January 03, 2020

Absorption intensity											
**Wavelength (nm)**	**concentration of β-hydroxyisovalerylshikonin (M)**										
	A	*B*	*C*	*D*	*E*	*F*	*G*	*I*	*J*	*K*	*L*
	0.00	$8.0x10^{-7}$	$1.6x10^{-6}$	$2.0x10^{-6}$	$2.8x10^{-6}$	$4.0x10^{-6}$	$4.8x10^{-6}$	$6.0x10^{-6}$	$8.0x10^{-6}$	$1.2x10^{-5}$	$1.6x10^{-5}$
209	2.889804	6.231919	10	5.528996	5.602117	5.718379	6.355149	10	5.552064	10	10
208	3.069213	4.938128	5.113108	4.932914	5.034918	5.34381	5.243088	5.22961	5.160325	10	5.694117
207	3.273154	5.17213	4.896669	4.816523	4.895759	5.038775	5.496679	5.357518	5.552958	10	10
206	3.486844	5.129173	5.401081	4.865555	5.024911	5.281723	5.097998	5.300761	5.299802	10	5.0324
205	3.684689	4.955998	5.357376	4.851567	5.231459	10	10	10	10	10	10
204	3.780703	5.819227	5.071044	4.76284	5.058674	10	10	10	5.312428	10	6.654011
203	3.787485	4.957387	4.832288	4.412284	4.698429	4.951074	4.963037	5.523756	4.874654	10	4.711803
202	3.608617	4.369341	4.251998	4.03921	4.314976	4.432648	4.893312	10	4.252983	10	4.332572
201	3.252297	3.860713	3.929042	3.626342	3.485626	3.88089	4.144917	4.334074	3.663544	4.693763	3.710805
200	2.731158	3.347729	3.050546	2.942838	2.978923	3.08801	3.327185	3.162834	3.11185	3.397008	3.151285

EXCLI Journal 2020;19:48-70 – ISSN 1611-2156
Received: October 03, 2019, accepted: December 19, 2019, published: January 03, 2020

Table 4: Absorption intensity in the wavelength range of 200-800 nm for absorption spectra of different concentration of α–methylbutyrylshikon (*A-L*)

**Wavelength (nm)**	***Absorption intensity*** **concentration of α-methylbutyrylshikon (M)**										
	A	*B*	*C*	*D*	*E*	*F*	*G*	*I*	*J*	*K*	*L*
	0.00	$8.0x10^{-7}$	$1.6x10^{-6}$	$2.0x10^{-6}$	$2.8x10^{-6}$	$4.0x10^{-6}$	$4.8x10^{-6}$	$6.0x10^{-6}$	$8.0x10^{-6}$	$1.2x10^{-5}$	$1.6x10^{-5}$
800	0.002385	0.000553	0.005106	0.001881	0.000495	0.00103	0.000975	0.00012	-0.0001	0.001641	0.000521
799	0.002228	0.000401	0.004723	0.001411	0.000366	0.000992	0.000551	-0.00032	-0.00015	0.001347	0.000439
798	0.001803	0.000264	0.004502	0.001277	0.000222	0.000661	0.00074	-0.00062	-0.00025	0.001054	-0.0001
797	0.002473	0.000671	0.005031	0.001521	0.000569	0.001299	0.001166	-4.96E-05	0.000503	0.001671	0.000812
796	0.002463	0.000364	0.004727	0.001507	0.000207	0.000994	0.000991	-0.00033	9.55E-06	0.001309	0.000416
795	0.002083	7.00E-05	0.004958	0.00148	0.000158	0.000846	0.000654	-0.00059	-0.00019	0.001644	0.000161
794	0.002922	0.000852	0.005269	0.001691	0.000552	0.001157	0.001278	3.31E-05	0.000372	0.001648	0.000605
793	0.002394	0.000781	0.004933	0.001784	0.000424	0.001009	0.001254	-5.09E-05	0.000171	0.001547	0.000648
792	0.002204	0.000216	0.004554	0.001042	0.00034	0.00082	0.000659	-0.00055	-5.81E-05	0.001578	0.000153
791	0.002274	0.000459	0.004864	0.001303	0.000392	0.000977	0.000782	-0.00018	-5.78E-05	0.001469	0.000285
790	0.002115	0.000682	0.004839	0.001474	0.000332	0.001062	0.000772	-0.00043	0.000133	0.001605	0.000267
789	0.002272	0.000132	0.004418	0.000925	0.000169	0.000813	0.000781	-0.00064	-0.00022	0.001312	0.000148
788	0.002448	0.000384	0.0048	0.001212	0.000242	0.000935	0.000488	-0.00027	9.22E-05	0.001302	0.000331
787	0.00239	0.000659	0.004911	0.001504	0.000313	0.000966	0.000876	-0.00025	0.000133	0.001427	0.000277
786	0.002678	0.000793	0.004869	0.001726	0.00056	0.001207	0.001257	-0.00018	8.28E-05	0.001594	0.000719
785	0.002217	0.000497	0.004749	0.001293	0.000364	0.000831	0.000633	-0.00054	-0.00045	0.001379	7.22E-05
784	0.002213	0.00041	0.004846	0.001198	0.000256	0.000894	0.000975	-0.00046	0.00026	0.001454	0.000339
783	0.002062	9.60E-05	0.004886	0.001573	0.000382	0.000969	0.000848	-0.00032	8.73E-05	0.001402	0.00044
782	0.002412	0.000567	0.004663	0.001466	0.00056	0.001249	0.000883	-4.86E-05	0.000212	0.001592	0.000477
781	0.002008	0.000423	0.004662	0.001393	0.000286	0.000841	0.000976	-0.00037	-0.00015	0.001457	0.000106
780	0.002077	0.000378	0.004512	0.001184	0.00015	0.000724	0.000696	-0.00062	-0.00032	0.001225	0.000254
779	0.002289	0.00045	0.005031	0.001768	0.000424	0.001064	0.000824	-0.0005	0.000129	0.001823	0.000461
778	0.002097	0.000205	0.005053	0.001482	0.000429	0.000821	0.00114	-0.00029	1.61E-05	0.001647	0.000424
777	0.002406	0.000647	0.005235	0.001614	0.000611	0.000959	0.001293	-0.00018	0.000232	0.002087	0.000658
776	0.002281	0.00054	0.004653	0.001524	0.000422	0.001061	0.000847	-0.00029	0.000181	0.001689	0.000294
775	0.002279	0.00051	0.004762	0.001267	0.000409	0.00085	0.000879	-0.00041	6.14E-05	0.001588	0.000107
774	0.002497	0.000492	0.005032	0.001591	0.000324	0.00109	0.001091	-0.00018	3.50E-05	0.001847	0.000591
773	0.001882	0.000288	0.004365	0.001198	0.000173	0.000773	0.000523	-0.00045	-0.00018	0.001413	0.000119
772	0.00245	0.000374	0.005048	0.001362	0.000547	0.00138	0.001077	-0.00037	0.000173	0.001892	0.000427
771	0.002124	0.000285	0.004737	0.001396	0.000254	0.000843	0.00099	-0.00045	-0.00026	0.00157	0.000474

Absorption intensity											
**Wavelength (nm)**	**concentration of α-methylbutyrylshikon (M)**										
	A	*B*	*C*	*D*	*E*	*F*	*G*	*I*	*J*	*K*	*L*
	0.00	$8.0x10^{-7}$	$1.6x10^{-6}$	$2.0x10^{-6}$	$2.8x10^{-6}$	$4.0x10^{-6}$	$4.8x10^{-6}$	$6.0x10^{-6}$	$8.0x10^{-6}$	$1.2x10^{-5}$	$1.6x10^{-5}$
770	0.002596	0.000473	0.005152	0.001483	0.000492	0.000926	0.000883	-0.00029	-7.94E-05	0.001661	0.000384
769	0.002478	0.000325	0.004908	0.001589	0.000283	0.001291	0.001047	-0.00026	0.000253	0.001556	0.000561
768	0.002476	0.000693	0.004844	0.001629	0.000552	0.001286	0.001174	-0.00034	0.000104	0.001731	0.00057
767	0.002098	0.000406	0.004709	0.001351	0.000129	0.000853	0.000826	-0.0003	-8.86E-05	0.00165	0.000272
766	0.002486	0.000622	0.005264	0.001495	0.000424	0.001274	0.001002	-0.00024	0.000209	0.001816	0.00054
765	0.002284	0.000855	0.005202	0.00166	0.000646	0.001359	0.001052	-4.45E-06	0.00031	0.002024	0.000507
764	0.00224	0.000553	0.005274	0.001746	0.000671	0.001232	0.001095	-0.00015	0.000179	0.001875	0.000688
763	0.002236	0.000552	0.004946	0.001471	0.000353	0.000922	0.001138	-0.00032	1.97E-05	0.00165	0.000516
762	0.002221	0.000363	0.004543	0.001358	0.000314	0.000741	0.000942	-0.00023	-5.18E-05	0.00159	0.000403
761	0.002451	0.000468	0.005221	0.001822	0.000487	0.001021	0.001065	-0.00024	9.36E-05	0.001958	0.000846
760	0.002716	0.000626	0.005185	0.001729	0.000527	0.001309	0.001337	-1.59E-05	0.000243	0.002049	0.000709
759	0.002458	0.000487	0.004888	0.001571	0.000379	0.000954	0.000865	-0.00028	-2.90E-05	0.001873	0.000451
758	0.002552	0.000565	0.005091	0.001621	0.000484	0.001325	0.001022	-0.00022	0.000206	0.001876	0.000697
757	0.002098	0.000449	0.004876	0.001528	0.00063	0.000926	0.001061	-0.00053	8.55E-05	0.001949	0.000469
756	0.00257	0.000593	0.005171	0.001745	0.000585	0.001174	0.001059	-0.00015	0.000212	0.001788	0.00085
755	0.002231	0.000309	0.005056	0.001598	0.000326	0.000921	0.000802	-0.00032	5.44E-06	0.001745	0.000552
754	0.002425	0.000658	0.005104	0.00169	0.000405	0.001028	0.001015	-0.00017	0.000129	0.001892	0.000742
753	0.002417	0.000578	0.005208	0.001735	0.000667	0.001082	0.00114	-0.0003	0.000129	0.001924	0.000718
752	0.002288	0.000604	0.005228	0.001553	0.000421	0.001096	0.001257	-0.00035	9.58E-05	0.002162	0.000644
751	0.00252	0.000644	0.005182	0.001885	0.000607	0.001219	0.001211	-0.00022	0.000269	0.002216	0.000858
750	0.002349	0.000753	0.004775	0.00145	0.000402	0.001265	0.001073	-0.00032	0.000115	0.001933	0.000792
749	0.002363	0.000506	0.005058	0.001515	0.000326	0.001093	0.001172	-0.00041	0.000101	0.002095	0.000675
748	0.002634	0.000577	0.005173	0.001629	0.000485	0.001125	0.001218	-0.00014	0.000224	0.002117	0.00086
747	0.002087	0.000421	0.00485	0.001331	0.000291	0.000832	0.000832	-0.00049	-0.00022	0.002013	0.000499
746	0.002444	0.000627	0.005046	0.001619	0.000523	0.001212	0.001036	-0.00028	0.000271	0.00201	0.000977
745	0.002234	0.000433	0.004883	0.001422	0.000309	0.001087	0.00101	-0.0006	-9.35E-05	0.001913	0.000702
744	0.002245	0.000232	0.004871	0.001447	0.000431	0.000839	0.000893	-0.00026	-9.11E-05	0.001809	0.000662
743	0.002282	0.000322	0.004873	0.001534	0.000464	0.001076	0.001002	-0.0003	9.53E-05	0.002014	0.000703
742	0.002389	0.000515	0.005116	0.00153	0.000452	0.001113	0.001085	-0.0003	0.000215	0.00212	0.000864
741	0.002445	0.000495	0.005305	0.001616	0.000494	0.000936	0.001035	-0.00031	0.000259	0.002248	0.00096
740	0.002088	0.000361	0.004997	0.001561	0.000236	0.001069	0.000948	-0.00038	0.000192	0.002189	0.001046
739	0.002315	0.000515	0.005166	0.00161	0.000499	0.001056	0.001315	-0.00031	0.000214	0.002314	0.000969
738	0.002701	0.000593	0.005295	0.001926	0.000645	0.001205	0.001266	-0.00015	0.000488	0.002674	0.001341

EXCLI Journal 2020;19:48-70 – ISSN 1611-2156
Received: October 03, 2019, accepted: December 19, 2019, published: January 03, 2020

Absorption intensity											
**Wavelength (nm)**	**concentration of α-methylbutyrylshikon (M)**										
	A	*B*	*C*	*D*	*E*	*F*	*G*	*I*	*J*	*K*	*L*
	0.00	$8.0x10^{-7}$	$1.6x10^{-6}$	$2.0x10^{-6}$	$2.8x10^{-6}$	$4.0x10^{-6}$	$4.8x10^{-6}$	$6.0x10^{-6}$	$8.0x10^{-6}$	$1.2x10^{-5}$	$1.6x10^{-5}$
737	0.002488	0.000483	0.005232	0.00153	0.000484	0.001046	0.001319	-4.45E-05	0.000177	0.002538	0.001041
736	0.002013	0.000154	0.004829	0.001489	0.000202	0.000933	0.0009	-0.00047	8.76E-05	0.002271	0.000871
735	0.00238	0.000408	0.005137	0.001764	0.000625	0.001149	0.001336	3.78E-05	0.000392	0.002769	0.00139
734	0.002192	0.000289	0.005001	0.001475	0.000468	0.001009	0.001216	-0.00029	0.00024	0.002445	0.001212
733	0.002398	0.000505	0.005211	0.00166	0.000527	0.001253	0.001294	-4.73E-05	0.000382	0.002736	0.001596
732	0.002284	0.000477	0.005133	0.001807	0.000492	0.001139	0.001373	8.12E-05	0.000559	0.002787	0.001585
731	0.002102	0.000399	0.005088	0.001637	0.000434	0.001133	0.001344	-0.00018	0.000574	0.00287	0.001654
730	0.002382	0.000533	0.005425	0.001877	0.000646	0.001457	0.001577	0.000155	0.000654	0.003218	0.001894
729	0.002295	0.000557	0.005242	0.001843	0.000549	0.001446	0.001495	0.000126	0.000639	0.003103	0.001978
728	0.002464	0.000619	0.005376	0.001611	0.000511	0.00121	0.00158	9.68E-05	0.000809	0.003207	0.0021
727	0.002382	0.000517	0.00523	0.001753	0.000622	0.001306	0.001538	0.000119	0.000832	0.003163	0.002246
726	0.002662	0.000407	0.005228	0.001666	0.000657	0.001514	0.001401	4.45E-05	0.000648	0.003286	0.002101
725	0.00218	0.000346	0.005265	0.001568	0.000521	0.001315	0.00149	3.23E-05	0.00069	0.003206	0.00201
724	0.002341	0.000559	0.005339	0.00185	0.000679	0.001393	0.001557	0.00017	0.000697	0.003292	0.002233
723	0.002242	0.000501	0.005434	0.001749	0.000687	0.001237	0.001595	0.000227	0.000832	0.003396	0.002265
722	0.002496	0.000718	0.005585	0.001802	0.000758	0.001458	0.001644	0.000405	0.001089	0.003512	0.002332
721	0.002115	0.000334	0.005232	0.001602	0.000525	0.001412	0.001622	0.000158	0.000846	0.003437	0.00224
720	0.002347	0.000453	0.005286	0.001805	0.000729	0.001478	0.001664	0.000226	0.000896	0.003431	0.002429
719	0.002376	0.000496	0.005413	0.001715	0.000848	0.001582	0.001656	0.000254	0.000899	0.003584	0.002413
718	0.002168	0.000237	0.005158	0.001678	0.000553	0.001357	0.001671	0.000152	0.000714	0.003592	0.002513
717	0.002298	0.000518	0.005301	0.001849	0.000624	0.001551	0.001683	0.00019	0.000962	0.003744	0.002572
716	0.002211	0.000543	0.005427	0.001776	0.000553	0.001359	0.001762	0.000328	0.000934	0.003744	0.002717
715	0.002369	0.000579	0.005496	0.001899	0.000596	0.001503	0.001836	0.000417	0.001109	0.003877	0.002803
714	0.002241	0.000484	0.005368	0.001854	0.000708	0.001479	0.00184	0.0003	0.001067	0.003982	0.002898
713	0.002305	0.000511	0.005426	0.001971	0.000661	0.001512	0.001671	0.000355	0.00102	0.003829	0.002749
712	0.002272	0.000334	0.005424	0.001775	0.000725	0.001257	0.001675	0.000307	0.00105	0.003852	0.002946
711	0.002294	0.000323	0.005469	0.00202	0.00085	0.001652	0.001885	0.000268	0.00118	0.004026	0.003183
710	0.002382	0.000502	0.005491	0.001838	0.000654	0.001499	0.001854	0.000267	0.001108	0.004209	0.003143
709	0.002361	0.000534	0.005472	0.001956	0.000838	0.001697	0.001914	0.000423	0.001106	0.004069	0.003301
708	0.002282	0.000365	0.005455	0.001853	0.000659	0.001448	0.001898	0.000281	0.001129	0.004175	0.003254
707	0.002453	0.000316	0.005393	0.001905	0.000815	0.001493	0.001897	0.000312	0.001268	0.004153	0.003195
706	0.002506	0.000516	0.005695	0.002205	0.000848	0.00167	0.00203	0.000633	0.001397	0.00439	0.003486
705	0.002375	0.000605	0.005478	0.001991	0.000952	0.00172	0.002098	0.000568	0.001329	0.004481	0.0036

EXCLI Journal 2020;19:48-70 – ISSN 1611-2156
Received: October 03, 2019, accepted: December 19, 2019, published: January 03, 2020

Absorption intensity											
**Wavelength (nm)**	**concentration of α-methylbutyrylshikon (M)**										
	A	*B*	*C*	*D*	*E*	*F*	*G*	*I*	*J*	*K*	*L*
	0.00	$8.0x10^{-7}$	$1.6x10^{-6}$	$2.0x10^{-6}$	$2.8x10^{-6}$	$4.0x10^{-6}$	$4.8x10^{-6}$	$6.0x10^{-6}$	$8.0x10^{-6}$	$1.2x10^{-5}$	$1.6x10^{-5}$
704	0.002418	0.000542	0.005602	0.001927	0.000965	0.001679	0.001959	0.000643	0.001563	0.004574	0.003626
703	0.002368	0.000511	0.005646	0.00197	0.000792	0.00158	0.001961	0.000566	0.00142	0.004514	0.003598
702	0.002249	0.000481	0.005503	0.001842	0.000847	0.001598	0.002025	0.000444	0.001494	0.004613	0.003611
701	0.002275	0.000388	0.00537	0.001863	0.000812	0.001512	0.001948	0.000573	0.001258	0.004709	0.003777
700	0.002244	0.000497	0.005316	0.001879	0.000762	0.001493	0.002044	0.000376	0.00154	0.004535	0.003671
699	0.002338	0.000275	0.005397	0.001815	0.000698	0.001458	0.001981	0.000472	0.001544	0.004785	0.003935
698	0.002267	0.000425	0.005575	0.001884	0.000756	0.001774	0.002	0.000708	0.001569	0.004758	0.004035
697	0.002426	0.000658	0.005792	0.002209	0.001075	0.001771	0.002323	0.000702	0.00166	0.005054	0.004086
696	0.002258	0.000554	0.005462	0.001923	0.000913	0.001597	0.002166	0.000684	0.001517	0.005198	0.004309
695	0.002534	0.000736	0.00584	0.002211	0.001022	0.001897	0.002284	0.000791	0.001824	0.005343	0.00439
694	0.002332	0.000583	0.0056	0.00204	0.000871	0.001654	0.00223	0.000702	0.001803	0.005197	0.00437
693	0.002253	0.000509	0.005497	0.001877	0.000804	0.001608	0.002395	0.00056	0.001859	0.005117	0.004579
692	0.002339	0.000527	0.005588	0.001986	0.000976	0.001727	0.002313	0.000641	0.00175	0.005304	0.004439
691	0.002262	0.000326	0.005731	0.001953	0.000866	0.001706	0.002361	0.000796	0.001821	0.005321	0.004703
690	0.002289	0.000428	0.005594	0.001922	0.000935	0.001831	0.002502	0.000835	0.001936	0.005521	0.004781
689	0.002288	0.000577	0.005705	0.002027	0.000945	0.00178	0.002406	0.00095	0.001989	0.005592	0.004858
688	0.002026	0.000418	0.005333	0.00189	0.000663	0.001644	0.002171	0.000757	0.001758	0.00519	0.004545
687	0.002271	0.00028	0.005802	0.001895	0.000914	0.001732	0.002397	0.000834	0.001944	0.005812	0.005124
686	0.002305	0.000484	0.005753	0.001891	0.000992	0.001841	0.002368	0.000913	0.001932	0.005824	0.005173
685	0.002117	0.000568	0.005819	0.001962	0.000881	0.001737	0.002492	0.000919	0.002062	0.006027	0.005169
684	0.002301	0.000493	0.005778	0.002119	0.000965	0.001702	0.002436	0.000958	0.001945	0.006031	0.005457
683	0.002301	0.000552	0.005797	0.002122	0.001149	0.001933	0.002444	0.000934	0.002143	0.006132	0.005424
682	0.002174	0.000471	0.005709	0.001987	0.000849	0.001744	0.00236	0.000892	0.002091	0.00618	0.005407
681	0.002379	0.000541	0.005721	0.001931	0.001032	0.001738	0.002573	0.001066	0.002243	0.00623	0.005535
680	0.002002	0.000287	0.005514	0.001878	0.000797	0.001689	0.002367	0.000912	0.002031	0.006179	0.005541
679	0.002133	0.000425	0.005674	0.001914	0.00086	0.001843	0.002366	0.000975	0.002171	0.006299	0.005701
678	0.002373	0.000767	0.005884	0.00207	0.001071	0.00194	0.002741	0.001265	0.002532	0.006515	0.00599
677	0.002513	0.000624	0.0059	0.002131	0.001162	0.002039	0.002743	0.001236	0.002615	0.006681	0.006209
676	0.002432	0.000679	0.005962	0.00224	0.001046	0.001817	0.002784	0.001238	0.002575	0.006871	0.006114
675	0.002362	0.000483	0.00574	0.002035	0.000989	0.001899	0.002876	0.00112	0.002532	0.006728	0.006184
674	0.002216	0.000425	0.00583	0.002034	0.000961	0.002059	0.002786	0.001329	0.002535	0.006887	0.006427
673	0.00214	0.000494	0.005915	0.002104	0.001027	0.002041	0.002751	0.001284	0.00268	0.00701	0.006529
672	0.002312	0.000533	0.005952	0.002084	0.001058	0.002186	0.002887	0.001358	0.002727	0.0072	0.006753

EXCLI Journal 2020;19:48-70 – ISSN 1611-2156
Received: October 03, 2019, accepted: December 19, 2019, published: January 03, 2020

Absorption intensity											
**Wavelength (nm)**	**concentration of α-methylbutyrylshikon (M)**										
	A	*B*	*C*	*D*	*E*	*F*	*G*	*I*	*J*	*K*	*L*
	0.00	$8.0x10^{-7}$	$1.6x10^{-6}$	$2.0x10^{-6}$	$2.8x10^{-6}$	$4.0x10^{-6}$	$4.8x10^{-6}$	$6.0x10^{-6}$	$8.0x10^{-6}$	$1.2x10^{-5}$	$1.6x10^{-5}$
671	0.002181	0.00055	0.005811	0.002114	0.001135	0.00214	0.002828	0.001445	0.002712	0.007096	0.006659
670	0.002288	0.000519	0.005883	0.002099	0.001014	0.002045	0.00286	0.001433	0.002867	0.007264	0.006855
669	0.002245	0.00036	0.005767	0.002028	0.000943	0.001976	0.00288	0.001526	0.002656	0.007282	0.006873
668	0.002338	0.000568	0.00594	0.002044	0.001256	0.002069	0.002974	0.001549	0.002825	0.007642	0.007221
667	0.002335	0.000679	0.005961	0.002284	0.001277	0.002186	0.003191	0.001637	0.00326	0.007596	0.007266
666	0.002289	0.000599	0.005911	0.002131	0.001045	0.002204	0.003094	0.001507	0.003048	0.007848	0.007411
665	0.002222	0.000533	0.006045	0.002045	0.001221	0.00207	0.003021	0.001466	0.00308	0.007728	0.007668
664	0.00236	0.000561	0.005873	0.002179	0.001135	0.002113	0.003101	0.001559	0.003176	0.007991	0.007783
663	0.002466	0.000573	0.005963	0.002096	0.001044	0.002064	0.003076	0.00159	0.003126	0.008105	0.007773
662	0.002153	0.000447	0.005868	0.00217	0.000955	0.002106	0.003097	0.001553	0.003122	0.008149	0.007911
661	0.002431	0.000634	0.006022	0.002318	0.001246	0.002365	0.003418	0.001761	0.003473	0.008457	0.008169
660	0.002445	0.00067	0.006232	0.002283	0.001294	0.002338	0.003379	0.001804	0.003561	0.008622	0.008378
659	0.002357	0.000781	0.00618	0.002377	0.001431	0.002475	0.003593	0.001922	0.003689	0.008695	0.008598
658	0.00237	0.000706	0.006041	0.002256	0.001245	0.002398	0.00332	0.001824	0.003585	0.008742	0.008617
657	0.00212	0.000584	0.005931	0.002187	0.001107	0.002248	0.003556	0.001878	0.003577	0.008787	0.008549
656	0.002251	0.00059	0.005998	0.002225	0.001299	0.00234	0.003424	0.001844	0.003634	0.008975	0.008812
655	0.00241	0.00073	0.00622	0.002333	0.001302	0.00249	0.003538	0.002026	0.003866	0.009242	0.009158
654	0.002397	0.000535	0.006165	0.002311	0.001277	0.002327	0.003563	0.001929	0.003864	0.009222	0.00921
653	0.002332	0.000432	0.006052	0.002287	0.001226	0.00245	0.003546	0.002098	0.00387	0.009386	0.00934
652	0.002316	0.00048	0.005906	0.002144	0.001218	0.002412	0.003487	0.002036	0.003991	0.009448	0.009504
651	0.002401	0.00061	0.006109	0.002342	0.001338	0.002424	0.003666	0.002073	0.004135	0.009639	0.009662
650	0.002419	0.000711	0.006338	0.002485	0.001517	0.002502	0.003867	0.002426	0.004372	0.009965	0.010067
649	0.002437	0.000667	0.006127	0.002421	0.001382	0.002489	0.00378	0.002155	0.004352	0.010006	0.010071
648	0.002517	0.000759	0.006175	0.002412	0.001419	0.002595	0.003977	0.002433	0.004358	0.010101	0.010298
647	0.00246	0.000574	0.00624	0.002496	0.001433	0.002794	0.003954	0.002474	0.004551	0.010365	0.010455
646	0.002529	0.000669	0.006326	0.002548	0.001573	0.002698	0.004023	0.002508	0.004557	0.010591	0.010655
645	0.002568	0.000798	0.006283	0.00262	0.001529	0.002613	0.004011	0.002564	0.004688	0.01057	0.010809
644	0.002582	0.000818	0.006349	0.002607	0.001676	0.00286	0.004081	0.002726	0.004808	0.010756	0.01103
643	0.002478	0.000567	0.006361	0.002547	0.001549	0.002946	0.004169	0.002619	0.004887	0.010884	0.011219
642	0.002375	0.000621	0.006056	0.002486	0.001343	0.002607	0.004082	0.002637	0.004833	0.01103	0.011146
641	0.002422	0.000659	0.00614	0.002412	0.001512	0.002713	0.004134	0.002603	0.004962	0.011089	0.011476
640	0.002384	0.0007	0.006302	0.002534	0.001623	0.002914	0.004342	0.002725	0.005119	0.011371	0.011741
639	0.002421	0.000668	0.006331	0.002587	0.001678	0.002813	0.004346	0.002797	0.005228	0.011514	0.011902

EXCLI Journal 2020;19:48-70 – ISSN 1611-2156
Received: October 03, 2019, accepted: December 19, 2019, published: January 03, 2020

Wavelength (nm)	Absorption intensity										
	concentration of α-methylbutyrylshikon (M)										
	A	*B*	*C*	*D*	*E*	*F*	*G*	*I*	*J*	*K*	*L*
	0.00	$8.0x10^{-7}$	$1.6x10^{-6}$	$2.0x10^{-6}$	$2.8x10^{-6}$	$4.0x10^{-6}$	$4.8x10^{-6}$	$6.0x10^{-6}$	$8.0x10^{-6}$	$1.2x10^{-5}$	$1.6x10^{-5}$
638	0.00254	0.000615	0.00632	0.002604	0.001654	0.002886	0.004392	0.002952	0.005318	0.011591	0.012094
637	0.002495	0.000782	0.00625	0.00265	0.00156	0.002873	0.004458	0.00305	0.005395	0.01182	0.012331
636	0.002444	0.000756	0.006278	0.00261	0.00158	0.002908	0.00448	0.002981	0.00548	0.011996	0.012386
635	0.002499	0.000752	0.006334	0.00269	0.001621	0.002964	0.004558	0.003035	0.005666	0.01217	0.012702
634	0.002469	0.000742	0.006341	0.002708	0.001614	0.003111	0.004557	0.003115	0.005677	0.012416	0.012899
633	0.002574	0.000764	0.006431	0.002725	0.001664	0.003057	0.004676	0.003262	0.00587	0.012548	0.013033
632	0.002623	0.000805	0.006451	0.002753	0.001797	0.002976	0.00476	0.003313	0.005888	0.012634	0.013327
631	0.002533	0.0007	0.006378	0.002681	0.001751	0.003185	0.004733	0.003345	0.005955	0.012925	0.013485
630	0.002631	0.000761	0.006481	0.002803	0.001906	0.00316	0.005018	0.003423	0.006101	0.01315	0.013569
629	0.002662	0.000859	0.006599	0.002825	0.001783	0.003403	0.004974	0.003407	0.006335	0.013182	0.01399
628	0.002585	0.000689	0.006662	0.002741	0.001952	0.003265	0.004993	0.003347	0.006198	0.013388	0.014063
627	0.002713	0.000699	0.006514	0.002798	0.001837	0.00317	0.005033	0.003565	0.006481	0.01347	0.014155
626	0.002501	0.000796	0.006572	0.002669	0.001787	0.003256	0.004997	0.003572	0.00642	0.013625	0.014403
625	0.002571	0.000765	0.006653	0.002849	0.001879	0.003521	0.00525	0.003779	0.00669	0.013954	0.014753
624	0.002571	0.000721	0.006386	0.002689	0.001808	0.003324	0.005189	0.003574	0.00664	0.013909	0.014797
623	0.002696	0.000929	0.006575	0.002868	0.002024	0.003482	0.005326	0.003728	0.006684	0.014127	0.015166
622	0.002578	0.00078	0.006656	0.00276	0.001882	0.003378	0.005419	0.003932	0.006864	0.014389	0.01536
621	0.002558	0.000668	0.006375	0.002668	0.001911	0.003299	0.005232	0.003737	0.006833	0.014521	0.015422
620	0.002663	0.000905	0.00674	0.002939	0.002071	0.00349	0.005514	0.004013	0.007223	0.014735	0.015677
619	0.002646	0.00082	0.006781	0.002896	0.00208	0.003598	0.005589	0.003999	0.007215	0.014945	0.015979
618	0.002595	0.000764	0.006671	0.002853	0.002032	0.003708	0.005503	0.004073	0.007207	0.015273	0.016151
617	0.002729	0.00081	0.00683	0.00287	0.001946	0.003602	0.005618	0.004123	0.007404	0.015227	0.016449
616	0.00266	0.0006	0.006726	0.002869	0.001949	0.003607	0.005612	0.004231	0.007278	0.015475	0.016494
615	0.002768	0.000839	0.006763	0.003008	0.00203	0.00367	0.005705	0.004372	0.007618	0.015706	0.01679
614	0.002727	0.000853	0.006835	0.003111	0.002153	0.003778	0.005836	0.004393	0.007721	0.015973	0.017119
613	0.002702	0.000885	0.006874	0.003067	0.002162	0.003846	0.005887	0.004487	0.007825	0.0161	0.017248
612	0.002732	0.000824	0.006946	0.003109	0.002083	0.003808	0.005955	0.00448	0.00786	0.016289	0.017514
611	0.002699	0.000945	0.007004	0.003133	0.002123	0.003815	0.006035	0.004542	0.008084	0.016446	0.017645
610	0.002653	0.000888	0.006992	0.003074	0.002202	0.003963	0.005996	0.004601	0.008091	0.016631	0.017904
609	0.002599	0.000891	0.006884	0.003054	0.002275	0.00401	0.006208	0.004788	0.008148	0.016858	0.018267
608	0.00273	0.000898	0.006915	0.003145	0.002209	0.003891	0.006123	0.004681	0.008366	0.017054	0.018304
607	0.002681	0.000815	0.007012	0.003105	0.002122	0.004061	0.006323	0.004807	0.008357	0.017139	0.018578
606	0.002669	0.000933	0.007113	0.00317	0.002298	0.003912	0.006349	0.004946	0.008463	0.017491	0.018791

EXCLI Journal 2020;19:48-70 – ISSN 1611-2156
Received: October 03, 2019, accepted: December 19, 2019, published: January 03, 2020

Absorption intensity											
**Wavelength (nm)**	**concentration of α-methylbutyrylshikon (M)**										
	A	*B*	*C*	*D*	*E*	*F*	*G*	*I*	*J*	*K*	*L*
	0.00	$8.0x10^{-7}$	$1.6x10^{-6}$	$2.0x10^{-6}$	$2.8x10^{-6}$	$4.0x10^{-6}$	$4.8x10^{-6}$	$6.0x10^{-6}$	$8.0x10^{-6}$	$1.2x10^{-5}$	$1.6x10^{-5}$
605	0.002737	0.000883	0.007102	0.00328	0.00235	0.004039	0.006464	0.005004	0.008751	0.017562	0.019024
604	0.002522	0.000786	0.007028	0.003022	0.002089	0.003989	0.00632	0.00489	0.00874	0.017662	0.019149
603	0.002867	0.00104	0.007264	0.003295	0.002414	0.00432	0.006668	0.005062	0.009018	0.018121	0.019724
602	0.002759	0.000918	0.007196	0.003249	0.002362	0.004187	0.006639	0.005147	0.009148	0.01819	0.019735
601	0.002634	0.000862	0.007078	0.003235	0.002358	0.004234	0.006706	0.005303	0.009038	0.018514	0.019891
600	0.002657	0.000792	0.007155	0.003115	0.002355	0.004248	0.006779	0.005386	0.009175	0.018731	0.020311
599	0.002778	0.001012	0.007217	0.00333	0.00229	0.004265	0.006891	0.005401	0.009297	0.018911	0.020444
598	0.002859	0.001035	0.00735	0.003385	0.002533	0.004493	0.007037	0.005546	0.009475	0.019113	0.020852
597	0.002685	0.000835	0.007191	0.00317	0.002331	0.004375	0.006848	0.005484	0.009402	0.019071	0.020849
596	0.002824	0.001001	0.007354	0.003474	0.002502	0.004562	0.007118	0.005663	0.009618	0.019485	0.02121
595	0.002847	0.000905	0.007311	0.003408	0.002438	0.004454	0.007214	0.005876	0.009756	0.019582	0.021519
594	0.002885	0.001097	0.007468	0.003523	0.002628	0.004636	0.007317	0.005924	0.010009	0.020143	0.021902
593	0.00284	0.000922	0.007327	0.003351	0.002631	0.004658	0.007347	0.005839	0.009989	0.020161	0.021994
592	0.002815	0.000995	0.007297	0.003458	0.002624	0.00466	0.007407	0.006016	0.010152	0.02043	0.022159
591	0.00284	0.000923	0.007309	0.003474	0.002498	0.00463	0.007467	0.005938	0.010271	0.02058	0.022629
590	0.002832	0.000915	0.007289	0.003416	0.002483	0.004578	0.007426	0.005947	0.01028	0.020802	0.022487
589	0.002948	0.001054	0.007443	0.003528	0.002638	0.004807	0.007647	0.006224	0.010407	0.021026	0.02292
588	0.002836	0.000931	0.007441	0.003395	0.002582	0.00479	0.007606	0.006246	0.01043	0.021264	0.023218
587	0.002823	0.000806	0.007454	0.003477	0.002625	0.004863	0.007714	0.006256	0.010704	0.021467	0.023419
586	0.002909	0.001047	0.007383	0.003427	0.002683	0.004878	0.007783	0.006398	0.010743	0.021766	0.023726
585	0.002818	0.000871	0.00741	0.003544	0.002514	0.005015	0.007861	0.006401	0.010784	0.021814	0.02389
584	0.002841	0.00107	0.007542	0.003758	0.002838	0.005023	0.008071	0.006629	0.011073	0.022124	0.024203
583	0.002683	0.000886	0.007403	0.003495	0.002578	0.004878	0.007947	0.006543	0.011087	0.022159	0.024265
582	0.002792	0.000894	0.007466	0.00359	0.002762	0.004993	0.008146	0.006612	0.011191	0.022405	0.02473
581	0.002763	0.000973	0.007496	0.003625	0.002826	0.005137	0.008215	0.006699	0.011217	0.022715	0.024982
580	0.002929	0.001086	0.007552	0.003839	0.002863	0.005292	0.008205	0.006938	0.011597	0.023042	0.025302
579	0.002884	0.001098	0.007598	0.003655	0.002823	0.005143	0.008286	0.00705	0.011499	0.023115	0.025543
578	0.002997	0.001079	0.007715	0.003706	0.002931	0.00537	0.008381	0.007064	0.011728	0.023495	0.025825
577	0.002909	0.001127	0.007762	0.003767	0.002818	0.005146	0.008482	0.007135	0.011784	0.023626	0.025875
576	0.002699	0.000901	0.007639	0.003631	0.002851	0.005202	0.008432	0.007092	0.011659	0.023491	0.02602
575	0.002866	0.001019	0.007653	0.003643	0.003013	0.005346	0.008539	0.007238	0.011887	0.023881	0.026319
574	0.00279	0.000992	0.007685	0.003568	0.002856	0.005211	0.008607	0.007186	0.012118	0.023854	0.026141
573	0.002907	0.001069	0.007803	0.003845	0.003023	0.005512	0.008624	0.007449	0.012222	0.024013	0.026445

EXCLI Journal 2020;19:48-70 – ISSN 1611-2156
Received: October 03, 2019, accepted: December 19, 2019, published: January 03, 2020

Absorption intensity											
**Wavelength (nm)**	**concentration of α-methylbutyrylshikon (M)**										
	A	*B*	*C*	*D*	*E*	*F*	*G*	*I*	*J*	*K*	*L*
	0.00	8.0x10^{-7}	1.6x10^{-6}	2.0x10^{-6}	2.8x10^{-6}	4.0x10^{-6}	4.8x10^{-6}	6.0x10^{-6}	8.0x10^{-6}	1.2x10^{-5}	1.6x10^{-5}
572	0.003019	0.001127	0.007848	0.003827	0.003141	0.005474	0.008804	0.007564	0.012434	0.024165	0.026709
571	0.002862	0.000998	0.007757	0.003866	0.003075	0.00541	0.008705	0.00748	0.012331	0.024181	0.026971
570	0.002945	0.000823	0.007776	0.003927	0.002928	0.005638	0.008727	0.007538	0.012412	0.0244	0.027124
569	0.002947	0.001077	0.007794	0.003911	0.003217	0.005609	0.008898	0.007554	0.012615	0.024635	0.027246
568	0.002951	0.001184	0.008001	0.00387	0.003195	0.005735	0.009027	0.007695	0.012756	0.025405	0.027882
567	0.002908	0.001131	0.007969	0.00392	0.003257	0.00572	0.008997	0.007673	0.012726	0.025306	0.027933
566	0.002973	0.001176	0.007885	0.003927	0.003275	0.005792	0.009172	0.007934	0.012871	0.025453	0.028297
565	0.002959	0.001187	0.007906	0.004009	0.003266	0.005735	0.009198	0.007828	0.01291	0.025592	0.028384
564	0.002849	0.001168	0.007953	0.004072	0.003128	0.005835	0.009168	0.007864	0.013131	0.025841	0.028527
563	0.002889	0.000989	0.007934	0.003805	0.00311	0.005733	0.009382	0.007765	0.013095	0.025856	0.02863
562	0.002813	0.000997	0.007971	0.003785	0.003108	0.005697	0.009158	0.007879	0.012926	0.025991	0.028765
561	0.003107	0.001276	0.008153	0.004288	0.003515	0.005957	0.009314	0.008128	0.013335	0.026422	0.029133
560	0.002846	0.00108	0.008043	0.003947	0.003374	0.005972	0.009526	0.008128	0.013187	0.026433	0.029264
559	0.002969	0.001222	0.008169	0.004001	0.003297	0.005938	0.00938	0.008173	0.013512	0.026719	0.029557
558	0.002975	0.001005	0.008107	0.003928	0.003251	0.005958	0.009455	0.008252	0.013444	0.026675	0.029637
557	0.002958	0.001076	0.008223	0.004023	0.003318	0.00604	0.009559	0.008269	0.013628	0.02687	0.029854
556	0.002974	0.001089	0.008079	0.004123	0.003459	0.006026	0.009562	0.008315	0.013536	0.027054	0.029994
555	0.00306	0.001257	0.008129	0.004125	0.00334	0.006094	0.00971	0.008463	0.013605	0.027193	0.03025
554	0.003064	0.001246	0.008313	0.004224	0.003434	0.006234	0.009966	0.008659	0.013942	0.027353	0.030474
553	0.003149	0.001322	0.008381	0.004369	0.003672	0.006119	0.009896	0.008629	0.013912	0.027594	0.030701
552	0.003127	0.001385	0.00836	0.004276	0.003464	0.006328	0.009845	0.008747	0.013968	0.027738	0.030894
551	0.002897	0.001138	0.008185	0.004139	0.003417	0.006195	0.009912	0.008666	0.013978	0.027817	0.030827
550	0.003069	0.001222	0.008482	0.004304	0.003602	0.006403	0.009948	0.008623	0.014093	0.028049	0.031127
549	0.003181	0.001309	0.008459	0.00442	0.003596	0.006322	0.010099	0.008923	0.014261	0.028207	0.031452
548	0.002983	0.001252	0.008442	0.004156	0.003689	0.006386	0.010152	0.008989	0.014227	0.028292	0.031511
547	0.003134	0.001322	0.0085	0.00435	0.003675	0.006552	0.01017	0.008956	0.01451	0.028553	0.031789
546	0.00301	0.001247	0.008352	0.004328	0.003685	0.006373	0.010115	0.008994	0.014449	0.028787	0.03191
545	0.003136	0.001229	0.008441	0.004329	0.003568	0.006585	0.010352	0.009047	0.014574	0.028814	0.032249
544	0.003164	0.001311	0.008504	0.00451	0.003792	0.006464	0.010488	0.009168	0.014575	0.029019	0.032298
543	0.003072	0.001364	0.008516	0.004357	0.003734	0.006509	0.010281	0.009128	0.014678	0.029191	0.032582
542	0.002956	0.00113	0.008332	0.004405	0.003547	0.006527	0.010472	0.009273	0.014705	0.029185	0.032649
541	0.003013	0.001263	0.008454	0.00431	0.003696	0.006601	0.010361	0.009168	0.01478	0.029527	0.03289
540	0.003022	0.001313	0.008451	0.004326	0.003651	0.006641	0.010456	0.00934	0.014813	0.029572	0.032998

EXCLI Journal 2020;19:48-70 – ISSN 1611-2156
Received: October 03, 2019, accepted: December 19, 2019, published: January 03, 2020

Absorption intensity											
**Wavelength (nm)**	**concentration of α-methylbutyrylshikon (M)**										
	A	*B*	*C*	*D*	*E*	*F*	*G*	*I*	*J*	*K*	*L*
	0.00	$8.0x10^{-7}$	$1.6x10^{-6}$	$2.0x10^{-6}$	$2.8x10^{-6}$	$4.0x10^{-6}$	$4.8x10^{-6}$	$6.0x10^{-6}$	$8.0x10^{-6}$	$1.2x10^{-5}$	$1.6x10^{-5}$
539	0.003238	0.001488	0.008727	0.004652	0.00391	0.006865	0.010832	0.009557	0.015082	0.029868	0.033526
538	0.003057	0.001294	0.008501	0.004456	0.003704	0.006734	0.010593	0.009339	0.015007	0.02988	0.033349
537	0.003083	0.00138	0.008668	0.00454	0.003771	0.006735	0.010717	0.00956	0.015327	0.03005	0.03372
536	0.003264	0.001319	0.008726	0.004623	0.003944	0.006745	0.010838	0.00968	0.0153	0.030209	0.034018
535	0.003134	0.001316	0.008612	0.004449	0.003887	0.006843	0.010714	0.009575	0.015109	0.030344	0.033946
534	0.003187	0.001462	0.008717	0.004625	0.004081	0.006973	0.010908	0.009833	0.015476	0.03055	0.03422
533	0.003172	0.001374	0.008781	0.004615	0.003997	0.006991	0.010991	0.009918	0.015532	0.030795	0.034405
532	0.00321	0.001466	0.008891	0.00461	0.004244	0.00704	0.011126	0.010008	0.015628	0.03093	0.034509
531	0.002911	0.00125	0.00859	0.004547	0.004061	0.006875	0.010883	0.009704	0.015497	0.030722	0.034558
530	0.003187	0.001541	0.00885	0.00481	0.004107	0.007023	0.0112	0.01002	0.015632	0.031031	0.034751
529	0.0031	0.001279	0.008649	0.004402	0.003958	0.007118	0.010921	0.009851	0.015558	0.031076	0.034849
528	0.00311	0.001477	0.008839	0.004637	0.004139	0.007033	0.011224	0.009977	0.015721	0.031311	0.034999
527	0.003261	0.001553	0.008877	0.004855	0.00409	0.007083	0.011227	0.010075	0.015874	0.03146	0.035211
526	0.003117	0.001501	0.008765	0.00467	0.004228	0.007067	0.011195	0.010087	0.015885	0.031432	0.035384
525	0.003107	0.00145	0.008938	0.004806	0.004009	0.007183	0.011188	0.01006	0.015911	0.031738	0.035385
524	0.003054	0.001361	0.008822	0.004716	0.004117	0.007106	0.011357	0.010216	0.016108	0.031622	0.035606
523	0.003202	0.001503	0.008996	0.004851	0.004214	0.007418	0.011304	0.010319	0.016079	0.031874	0.035749
522	0.003377	0.00164	0.009004	0.005046	0.004444	0.007449	0.011378	0.010352	0.016149	0.031891	0.035903
521	0.003052	0.001505	0.009017	0.004778	0.004225	0.007202	0.011344	0.010274	0.016181	0.031851	0.035832
520	0.003116	0.001423	0.00882	0.004892	0.00424	0.007294	0.011329	0.010325	0.016106	0.03216	0.03598
519	0.003212	0.001644	0.009019	0.00488	0.004422	0.007518	0.011558	0.01047	0.016406	0.032204	0.036213
518	0.003252	0.001444	0.009083	0.00481	0.004254	0.007436	0.011463	0.010229	0.016236	0.032163	0.036061
517	0.003351	0.001568	0.009071	0.004918	0.004394	0.007557	0.011636	0.010557	0.016304	0.032383	0.03629
516	0.003219	0.001553	0.009219	0.004926	0.004367	0.007609	0.011645	0.010515	0.016294	0.032398	0.036198
515	0.00318	0.001493	0.009055	0.004874	0.004294	0.007419	0.011619	0.01056	0.016356	0.032299	0.036367
514	0.003208	0.001445	0.009256	0.005061	0.004494	0.007493	0.011695	0.010628	0.016388	0.032334	0.036553
513	0.003209	0.001554	0.009055	0.004984	0.00451	0.007609	0.011566	0.010548	0.016344	0.032628	0.036548
512	0.003326	0.001729	0.009544	0.005123	0.004767	0.007825	0.011881	0.010829	0.016699	0.032664	0.036792
511	0.003167	0.001582	0.009237	0.005265	0.004422	0.007643	0.01159	0.010723	0.016514	0.032722	0.036595
510	0.003335	0.001576	0.009184	0.005015	0.004677	0.007472	0.011776	0.010767	0.016657	0.032873	0.036994
509	0.00322	0.00165	0.009255	0.005163	0.004476	0.007731	0.011758	0.010597	0.016518	0.032733	0.036896
508	0.003316	0.001737	0.009258	0.005143	0.00445	0.007617	0.011805	0.010829	0.01672	0.032741	0.036921
507	0.003203	0.001452	0.0093	0.005152	0.004445	0.007613	0.011902	0.010653	0.016606	0.032939	0.037004

EXCLI Journal 2020;19:48-70 – ISSN 1611-2156
Received: October 03, 2019, accepted: December 19, 2019, published: January 03, 2020

Absorption intensity											
**Wavelength (nm)**	**concentration of α-methylbutyrylshikon (M)**										
	A	*B*	*C*	*D*	*E*	*F*	*G*	*I*	*J*	*K*	*L*
	0.00	$8.0x10^{-7}$	$1.6x10^{-6}$	$2.0x10^{-6}$	$2.8x10^{-6}$	$4.0x10^{-6}$	$4.8x10^{-6}$	$6.0x10^{-6}$	$8.0x10^{-6}$	$1.2x10^{-5}$	$1.6x10^{-5}$
506	0.003178	0.001541	0.00928	0.00514	0.004496	0.00767	0.011676	0.010742	0.016647	0.032974	0.037098
505	0.003464	0.001696	0.009483	0.005442	0.004689	0.007889	0.012017	0.01088	0.016799	0.033058	0.037244
504	0.0031	0.001458	0.009264	0.004999	0.004493	0.007568	0.011718	0.010624	0.016598	0.032963	0.036918
503	0.003297	0.001521	0.009317	0.005298	0.004534	0.007748	0.011807	0.010712	0.016693	0.033098	0.037231
502	0.003342	0.001752	0.009483	0.005241	0.004551	0.007765	0.011874	0.010822	0.016799	0.033175	0.037319
501	0.003273	0.001617	0.009299	0.005198	0.004518	0.007692	0.011845	0.011051	0.016566	0.033123	0.037371
500	0.003361	0.001607	0.009417	0.005267	0.004718	0.007761	0.012014	0.010846	0.016772	0.033319	0.037403
499	0.003096	0.001608	0.009456	0.005239	0.004583	0.007868	0.011867	0.01089	0.016784	0.033219	0.037303
498	0.00316	0.001488	0.009256	0.005152	0.004408	0.007716	0.011812	0.01066	0.016448	0.033036	0.037206
497	0.003248	0.001638	0.009543	0.005288	0.004672	0.00777	0.011968	0.010987	0.016891	0.033338	0.037427
496	0.003225	0.001688	0.009531	0.005329	0.004574	0.007878	0.011941	0.010835	0.016821	0.03329	0.037409
495	0.003407	0.001721	0.009557	0.005366	0.004727	0.008079	0.012153	0.010965	0.016721	0.03339	0.03757
494	0.003307	0.001798	0.009432	0.005408	0.004649	0.0079	0.012098	0.010824	0.016918	0.033426	0.037492
493	0.003273	0.001578	0.009463	0.005361	0.004579	0.007624	0.011947	0.010855	0.016831	0.033342	0.037393
492	0.003475	0.001699	0.009636	0.005227	0.004654	0.007778	0.012091	0.010865	0.017013	0.03335	0.037587
491	0.003168	0.001659	0.009524	0.005285	0.00462	0.00782	0.011933	0.010846	0.01691	0.033249	0.03744
490	0.003239	0.001704	0.009716	0.005359	0.0047	0.007878	0.011925	0.010858	0.016918	0.033371	0.037376
489	0.003351	0.001727	0.009649	0.005408	0.004605	0.007968	0.01204	0.01087	0.01707	0.033303	0.037363
488	0.003257	0.001667	0.009526	0.005403	0.004809	0.007716	0.011968	0.01075	0.016909	0.033247	0.037326
487	0.003489	0.001997	0.009687	0.005593	0.004688	0.008023	0.012037	0.010988	0.017197	0.033424	0.037428
486	0.003334	0.001661	0.009718	0.005403	0.00475	0.007888	0.011931	0.010809	0.016988	0.033387	0.037415
485	0.003333	0.001552	0.009561	0.005584	0.004665	0.00784	0.011842	0.010947	0.017105	0.033228	0.037308
484	0.003403	0.001714	0.009723	0.005491	0.004737	0.007869	0.012087	0.010994	0.017058	0.033284	0.037295
483	0.003515	0.001844	0.009756	0.005497	0.004774	0.007915	0.012072	0.010997	0.01695	0.033293	0.037156
482	0.003348	0.001611	0.009676	0.00541	0.00479	0.007956	0.011929	0.01081	0.017092	0.033037	0.037277
481	0.003576	0.001984	0.009873	0.005554	0.00483	0.007944	0.012022	0.011065	0.017203	0.033363	0.037104
480	0.003441	0.001689	0.009647	0.00532	0.004641	0.007813	0.011979	0.010748	0.017034	0.033039	0.037054
479	0.003604	0.001961	0.009957	0.005641	0.004977	0.007979	0.012175	0.010903	0.017109	0.033223	0.037264
478	0.003712	0.001869	0.009806	0.005662	0.004892	0.008024	0.012095	0.010897	0.017191	0.033097	0.037117
477	0.003488	0.001881	0.009843	0.005573	0.004755	0.007846	0.011998	0.010758	0.01704	0.032971	0.03686
476	0.003576	0.001921	0.009851	0.00555	0.004879	0.007864	0.01195	0.010824	0.017111	0.03298	0.036889
475	0.003699	0.001903	0.009924	0.005625	0.004847	0.008069	0.012151	0.010896	0.017164	0.032946	0.036952
474	0.003504	0.001914	0.009914	0.005534	0.0048	0.007866	0.011912	0.010645	0.01701	0.032914	0.036705

Received: October 03, 2019, accepted: December 19, 2019, published: January 03, 2020

Absorption intensity											
**Wavelength (nm)**	**concentration of α-methylbutyrylshikon (M)**										
	A	*B*	*C*	*D*	*E*	*F*	*G*	*I*	*J*	*K*	*L*
	0.00	8.0x10^{-7}	1.6x10^{-6}	2.0x10^{-6}	2.8x10^{-6}	4.0x10^{-6}	4.8x10^{-6}	6.0x10^{-6}	8.0x10^{-6}	1.2x10^{-5}	1.6x10^{-5}
473	0.003546	0.001831	0.009732	0.005576	0.004839	0.007954	0.01202	0.010715	0.017025	0.032876	0.036558
472	0.003648	0.001936	0.009935	0.005649	0.004797	0.007917	0.012064	0.010762	0.017062	0.032736	0.036673
471	0.003774	0.002042	0.010006	0.00577	0.004787	0.007887	0.012017	0.010716	0.017106	0.032788	0.036567
470	0.003646	0.001856	0.00999	0.00568	0.005055	0.0078	0.012029	0.010663	0.017107	0.032622	0.036483
469	0.003683	0.00187	0.009956	0.005774	0.004875	0.007937	0.011803	0.010722	0.016911	0.032635	0.036319
468	0.003861	0.002004	0.010184	0.005746	0.004972	0.00802	0.012211	0.010673	0.017111	0.032707	0.036384
467	0.003826	0.002128	0.010054	0.005743	0.00497	0.008074	0.012133	0.010609	0.01692	0.032522	0.036406
466	0.003741	0.001961	0.010053	0.005727	0.004959	0.007853	0.012006	0.010482	0.016901	0.032467	0.036022
465	0.003813	0.002049	0.010053	0.005778	0.004957	0.007918	0.012088	0.010554	0.016983	0.032424	0.036053
464	0.003701	0.001853	0.009855	0.00557	0.004836	0.007691	0.011705	0.010576	0.016792	0.031977	0.035493
463	0.003756	0.001932	0.010075	0.00579	0.004818	0.00786	0.011776	0.010432	0.016728	0.032079	0.035493
462	0.004032	0.002205	0.265228	0.00585	0.004806	0.007803	0.011999	0.010487	0.016732	0.031766	0.035368
461	0.003812	0.002096	0.010111	0.005743	0.005033	0.008047	0.012066	0.010474	0.016643	0.032032	0.035369
460	0.00394	0.00212	0.010072	0.00596	0.005034	0.007915	0.011775	0.010446	0.016334	0.031939	0.035222
459	0.003956	0.002217	0.009796	0.005705	0.004794	0.007751	0.011446	0.010192	0.016628	0.031707	0.035134
458	0.003596	0.001932	0.009743	0.005513	0.004628	0.007667	0.011631	0.010077	0.016441	0.031549	0.035128
457	0.003909	0.002147	0.010018	0.0059	0.004939	0.007858	0.011825	0.010247	0.016631	0.031744	0.03523
456	0.004121	0.00222	0.010263	0.006024	0.004956	0.008143	0.011895	0.010348	0.016951	0.032009	0.035242
455	0.003948	0.00227	0.009938	0.005758	0.004859	0.00797	0.011826	0.010403	0.016725	0.031945	0.035129
454	0.003991	0.002165	0.010219	0.005887	0.004871	0.007859	0.011756	0.01013	0.016706	0.031664	0.034988
453	0.004197	0.002227	0.010148	0.005878	0.004962	0.008117	0.011766	0.010136	0.016622	0.031691	0.034859
452	0.004057	0.002017	0.010103	0.005674	0.004821	0.007752	0.011643	0.010053	0.016574	0.031471	0.034745
451	0.004219	0.002027	0.009986	0.005648	0.004838	0.007652	0.011676	0.00991	0.016535	0.031585	0.034602
450	0.004326	0.002451	0.010224	0.006049	0.005004	0.007822	0.011824	0.010156	0.016642	0.031616	0.034642
449	0.004161	0.002194	0.010451	0.005969	0.004962	0.007865	0.011932	0.010178	0.016744	0.031282	0.034537
448	0.004122	0.002259	0.010182	0.005786	0.005109	0.007541	0.011882	0.009921	0.016416	0.031288	0.034281
447	0.004187	0.002317	0.010237	0.006091	0.004962	0.007946	0.011851	0.010083	0.016441	0.031233	0.034339
446	0.004125	0.002216	0.01022	0.005944	0.004961	0.007717	0.011602	0.010078	0.016519	0.03123	0.034065
445	0.004292	0.002394	0.010328	0.006047	0.004978	0.007912	0.011854	0.010056	0.016415	0.031195	0.03423
444	0.004271	0.002351	0.010462	0.006183	0.005	0.007765	0.011922	0.010029	0.016588	0.031199	0.03403
443	0.004346	0.002559	0.010367	0.006103	0.004964	0.007978	0.01192	0.009992	0.016434	0.031039	0.034163
442	0.004286	0.002238	0.010347	0.005863	0.004839	0.007783	0.011657	0.009814	0.016346	0.030985	0.03387
441	0.00422	0.002332	0.010206	0.006025	0.004838	0.00778	0.01176	0.009791	0.01627	0.031112	0.033791

EXCLI Journal 2020;19:48-70 – ISSN 1611-2156
Received: October 03, 2019, accepted: December 19, 2019, published: January 03, 2020

Absorption intensity											
**Wavelength (nm)**	**concentration of α-methylbutyrylshikon (M)**										
	A	*B*	*C*	*D*	*E*	*F*	*G*	*I*	*J*	*K*	*L*
	0.00	$8.0x10^{-7}$	$1.6x10^{-6}$	$2.0x10^{-6}$	$2.8x10^{-6}$	$4.0x10^{-6}$	$4.8x10^{-6}$	$6.0x10^{-6}$	$8.0x10^{-6}$	$1.2x10^{-5}$	$1.6x10^{-5}$
440	0.004507	0.002597	0.010696	0.006289	0.005056	0.008047	0.012048	0.009948	0.016615	0.031072	0.033891
439	0.004174	0.002281	0.010305	0.005979	0.004887	0.007748	0.011704	0.009725	0.016166	0.030838	0.033554
438	0.00434	0.002294	0.010439	0.006063	0.004902	0.007705	0.011728	0.00957	0.016284	0.03087	0.033439
437	0.004532	0.002407	0.010392	0.005951	0.004848	0.00766	0.011654	0.009674	0.016149	0.030747	0.033547
436	0.004457	0.002404	0.010588	0.006183	0.004811	0.007916	0.012038	0.009783	0.016311	0.030708	0.033498
435	0.004381	0.002305	0.010331	0.005924	0.004842	0.007528	0.011527	0.009569	0.015922	0.030642	0.033141
434	0.004554	0.002496	0.010486	0.006106	0.004964	0.007653	0.011775	0.009676	0.016185	0.030634	0.033352
433	0.004366	0.002378	0.010593	0.006108	0.004985	0.007743	0.01164	0.009466	0.016175	0.030505	0.033225
432	0.004445	0.002399	0.010416	0.006133	0.004866	0.00754	0.011763	0.009508	0.015996	0.030704	0.033057
431	0.004545	0.002453	0.010374	0.006024	0.004877	0.007541	0.011647	0.009409	0.015901	0.030379	0.032984
430	0.004542	0.002456	0.010531	0.006097	0.004995	0.007637	0.0118	0.009496	0.015998	0.030463	0.032978
429	0.004414	0.002333	0.010414	0.006163	0.004848	0.007694	0.011734	0.009362	0.015952	0.030575	0.032763
428	0.004818	0.002544	0.010702	0.006231	0.005139	0.007662	0.011783	0.009638	0.016025	0.030393	0.032846
427	0.004429	0.002204	0.010568	0.005924	0.004792	0.007444	0.011678	0.009229	0.015762	0.030339	0.032678
426	0.004527	0.002431	0.010439	0.006043	0.004985	0.007518	0.01157	0.009371	0.015842	0.030267	0.032384
425	0.00471	0.002495	0.010792	0.006313	0.00476	0.007588	0.011928	0.009399	0.015861	0.030417	0.032685
424	0.004506	0.002395	0.010514	0.005995	0.004742	0.007602	0.011828	0.009186	0.015655	0.030256	0.032514
423	0.004876	0.00262	0.010635	0.006424	0.005134	0.007637	0.011951	0.009398	0.015913	0.030368	0.032512
422	0.004773	0.002696	0.010724	0.00631	0.005069	0.007578	0.011706	0.009292	0.015784	0.030229	0.032356
421	0.004753	0.002524	0.010741	0.006312	0.005143	0.007668	0.011791	0.009409	0.01581	0.030211	0.032449
420	0.004736	0.00263	0.010738	0.00621	0.004959	0.007725	0.011924	0.009312	0.015676	0.030415	0.03232
419	0.004849	0.002656	0.010703	0.006265	0.004874	0.007537	0.011859	0.009193	0.015804	0.03017	0.032026
418	0.005011	0.002594	0.010832	0.006197	0.005172	0.007549	0.011774	0.009381	0.015393	0.029835	0.031928
417	0.004471	0.002487	0.010444	0.006036	0.004619	0.007267	0.011315	0.008868	0.015115	0.02994	0.031691
416	0.004933	0.002481	0.010868	0.006471	0.004893	0.007714	0.011827	0.009218	0.015682	0.030271	0.032044
415	0.00488	0.002541	0.010762	0.006472	0.00512	0.007518	0.011688	0.009148	0.015654	0.030109	0.031977
414	0.004855	0.002658	0.010675	0.006503	0.005036	0.007621	0.011896	0.009229	0.015604	0.030224	0.032128
413	0.004855	0.002531	0.010862	0.006433	0.004971	0.007546	0.011801	0.0092	0.015613	0.030279	0.032182
412	0.005059	0.002824	0.011004	0.006674	0.005121	0.007766	0.012044	0.009315	0.015873	0.030408	0.032145
411	0.004733	0.002423	0.010772	0.006548	0.004963	0.007533	0.01182	0.009197	0.015438	0.030177	0.03204
410	0.004697	0.002473	0.010696	0.006345	0.004907	0.007504	0.011681	0.008965	0.015486	0.030171	0.031836
409	0.004805	0.002636	0.010774	0.006498	0.004914	0.0076	0.011884	0.009152	0.015537	0.030111	0.03203
408	0.005046	0.002568	0.010829	0.006613	0.005108	0.007819	0.011994	0.009228	0.01547	0.030325	0.032082

Absorption intensity											
**Wavelength (nm)**	**concentration of α-methylbutyrylshikon (M)**										
	A	*B*	*C*	*D*	*E*	*F*	*G*	*I*	*J*	*K*	*L*
	0.00	$8.0x10^{-7}$	$1.6x10^{-6}$	$2.0x10^{-6}$	$2.8x10^{-6}$	$4.0x10^{-6}$	$4.8x10^{-6}$	$6.0x10^{-6}$	$8.0x10^{-6}$	$1.2x10^{-5}$	$1.6x10^{-5}$
407	0.005133	0.00273	0.010722	0.006629	0.005199	0.007782	0.012183	0.009214	0.015616	0.03052	0.032251
406	0.005177	0.002819	0.011047	0.006843	0.005128	0.007688	0.012042	0.009453	0.015647	0.03053	0.032192
405	0.005119	0.002845	0.010883	0.006716	0.005124	0.007862	0.011979	0.009116	0.015569	0.030641	0.032344
404	0.004949	0.002586	0.010796	0.006503	0.004896	0.007684	0.012028	0.00903	0.015543	0.03018	0.031861
403	0.004957	0.002736	0.010935	0.006214	0.005066	0.007753	0.011916	0.009223	0.015814	0.030079	0.031964
402	0.00506	0.002674	0.010683	0.006532	0.005306	0.00759	0.011998	0.009088	0.01534	0.030405	0.032145
401	0.004947	0.002724	0.010942	0.006964	0.005061	0.007629	0.012165	0.009349	0.01558	0.030666	0.032311
400	0.005394	0.002947	0.011192	0.007296	0.005276	0.007908	0.012297	0.009531	0.015717	0.030926	0.032574
399	0.005423	0.00288	0.011199	0.006815	0.005219	0.007925	0.012267	0.009515	0.015819	0.031068	0.032677
398	0.005146	0.002648	0.010852	0.006511	0.005061	0.007728	0.012156	0.009265	0.015816	0.031056	0.032629
397	0.005397	0.0029	0.011151	0.006795	0.005279	0.007935	0.012417	0.00945	0.015926	0.031067	0.032817
396	0.005536	0.00291	0.011239	0.006811	0.005389	0.00818	0.012692	0.009654	0.015794	0.03125	0.032902
395	0.005094	0.002714	0.011138	0.006609	0.005319	0.007743	0.012377	0.009341	0.015722	0.031165	0.03282
394	0.005629	0.003099	0.011418	0.006881	0.005372	0.008089	0.012569	0.009759	0.016182	0.031457	0.033264
393	0.005421	0.002841	0.011148	0.006995	0.005234	0.008036	0.012547	0.009486	0.016049	0.031477	0.033195
392	0.005514	0.00286	0.011254	0.006881	0.005388	0.007995	0.012782	0.009727	0.016437	0.031654	0.033411
391	0.005562	0.002829	0.011298	0.007288	0.005255	0.008156	0.012207	0.009711	0.016126	0.031856	0.033495
390	0.005908	0.003222	0.011637	0.007412	0.005792	0.0085	0.013359	0.010246	0.016608	0.032357	0.034275
389	0.005446	0.003211	0.01167	0.007202	0.0055	0.008355	0.013302	0.010057	0.016804	0.032061	0.033917
388	0.005639	0.003094	0.010663	0.006935	0.005551	0.008298	0.012446	0.009945	0.016131	0.031654	0.033316
387	0.005559	0.002981	0.011267	0.007302	0.005386	0.008151	0.012749	0.009859	0.016269	0.032428	0.033941
386	0.005735	0.002715	0.011577	0.007343	0.005535	0.00833	0.013077	0.010095	0.01674	0.032785	0.034363
385	0.005954	0.002841	0.011468	0.007556	0.005539	0.008286	0.012898	0.00994	0.016714	0.032868	0.034482
384	0.005831	0.002968	0.011423	0.007403	0.005695	0.008342	0.013125	0.010101	0.016761	0.033128	0.034605
383	0.006095	0.003305	0.011648	0.007779	0.006045	0.008559	0.013599	0.01022	0.016995	0.03302	0.034872
382	0.00543	0.002953	0.011168	0.007403	0.005388	0.008138	0.013063	0.010258	0.016841	0.033112	0.034707
381	0.005825	0.003015	0.011646	0.007353	0.005517	0.008469	0.013356	0.010279	0.017034	0.033228	0.034861
380	0.006247	0.002943	0.011657	0.008481	0.00613	0.008748	0.013635	0.010508	0.017071	0.03386	0.035705
379	0.006223	0.003437	0.01186	0.007989	0.006332	0.008815	0.014061	0.010736	0.017682	0.034007	0.035844
378	0.006318	0.00353	0.011821	0.007917	0.006294	0.008782	0.013964	0.011067	0.017464	0.033997	0.036059
377	0.006369	0.003536	0.01192	0.00817	0.00625	0.009096	0.014214	0.01091	0.017655	0.03457	0.036118
376	0.006473	0.003491	0.012055	0.008216	0.005944	0.009264	0.014103	0.011065	0.018042	0.035362	0.03695
375	0.006599	0.003688	0.01248	0.007826	0.006323	0.009193	0.014529	0.010943	0.018612	0.033731	0.036031

EXCLI Journal 2020;19:48-70 – ISSN 1611-2156
Received: October 03, 2019, accepted: December 19, 2019, published: January 03, 2020

Absorption intensity											
**Wavelength (nm)**	**concentration of α-methylbutyrylshikon (M)**										
	A	*B*	*C*	*D*	*E*	*F*	*G*	*I*	*J*	*K*	*L*
	0.00	$8.0x10^{-7}$	$1.6x10^{-6}$	$2.0x10^{-6}$	$2.8x10^{-6}$	$4.0x10^{-6}$	$4.8x10^{-6}$	$6.0x10^{-6}$	$8.0x10^{-6}$	$1.2x10^{-5}$	$1.6x10^{-5}$
374	0.00649	0.003409	0.011728	0.008188	0.006264	0.009475	0.014229	0.011209	0.017834	0.035186	0.036492
373	0.006652	0.003635	0.012409	0.008424	0.00649	0.009567	0.014545	0.01136	0.018349	0.035578	0.037051
372	0.006389	0.003545	0.011758	0.008192	0.006428	0.009159	0.014546	0.010755	0.01808	0.035165	0.036746
371	0.007071	0.003392	0.011808	0.00846	0.006462	0.009477	0.014628	0.011241	0.018691	0.035769	0.03731
370	0.006657	0.003521	0.011752	0.009377	0.006243	0.009518	0.014268	0.011525	0.018575	0.035594	0.037282
369	0.007167	0.004153	0.01264	0.009154	0.00697	0.009746	0.014896	0.011615	0.019149	0.036314	0.03827
368	0.007395	0.003972	0.012559	0.00916	0.006723	0.010176	0.01555	0.012087	0.019258	0.036811	0.03847
367	0.0069	0.0039	0.012602	0.008618	0.006791	0.009394	0.015004	0.011919	0.018859	0.035819	0.037612
366	0.007038	0.004004	0.012604	0.009488	0.006706	0.009867	0.015512	0.01202	0.019058	0.036967	0.038482
365	0.007118	0.004267	0.012649	0.009041	0.006898	0.009764	0.015125	0.012035	0.019249	0.037556	0.038901
364	0.005561	0.002371	0.010435	0.007585	0.005255	0.00818	0.013776	0.010327	0.01826	0.03549	0.037498
363	0.0071	0.003316	0.01237	0.0091	0.006439	0.009545	0.015263	0.011867	0.01948	0.037152	0.038961
362	0.009118	0.005919	0.014514	0.01151	0.008943	0.012049	0.018085	0.014209	0.021563	0.038459	0.039857
361	0.007269	0.004147	0.012679	0.009204	0.00706	0.010144	0.015855	0.012489	0.020127	0.037929	0.039785
360	0.007821	0.004574	0.012902	0.009771	0.007362	0.010675	0.016751	0.013098	0.020378	0.040719	0.042027
359	0.005492	0.002128	0.01103	0.007561	0.005247	0.008634	0.013779	0.011078	0.01842	0.036645	0.038504
358	0.008188	0.00415	0.013281	0.009974	0.006982	0.010663	0.016677	0.013344	0.020836	0.038983	0.041322
357	0.008865	0.004543	0.013885	0.010923	0.008243	0.011341	0.017542	0.013884	0.022684	0.038579	0.040519
356	0.007434	0.004167	0.012584	0.010121	0.007517	0.010618	0.017088	0.012516	0.020428	0.039883	0.041502
355	0.007377	0.00433	0.012188	0.010122	0.007592	0.009851	0.016416	0.01255	0.021065	0.041551	0.043795
354	0.004294	0.001811	0.009495	0.007113	0.004128	0.00705	0.013925	0.010522	0.017689	0.037791	0.038798
353	0.008947	0.004981	0.013625	0.011367	0.008481	0.011372	0.016906	0.013256	0.024074	0.041955	0.043839
352	0.011925	0.006986	0.017316	0.014476	0.011023	0.014218	0.019508	0.016247	0.020851	0.041095	0.042477
351	0.008077	0.004288	0.01129	0.008784	0.007259	0.010945	0.017345	0.012657	0.020682	0.042979	0.045514
350	0.004163	0.000131	0.010061	0.008004	0.003409	0.007068	0.013205	0.009286	0.016847	0.039596	0.040396
349	0.006763	0.003038	0.014867	0.011766	0.007049	0.012296	0.018205	0.014498	0.023899	0.041497	0.042976
348	0.011377	0.004863	0.010333	0.012221	0.007823	0.012894	0.019834	0.015138	0.023555	0.045642	0.045601
347	0.011453	0.004166	0.010482	0.01177	0.00817	0.012998	0.019766	0.014792	0.023901	0.045847	0.045654
346	0.011095	0.004794	0.010626	0.012288	0.008581	0.012988	0.020392	0.015097	0.024066	0.046284	0.045997
345	0.010328	0.00451	0.009591	0.011603	0.007342	0.0125	0.019862	0.014726	0.023919	0.045998	0.045962
344	0.01149	0.005094	0.010232	0.012413	0.008655	0.013324	0.020644	0.015779	0.024759	0.047017	0.046873
343	0.011799	0.004529	0.011226	0.012845	0.008576	0.013512	0.02101	0.015944	0.024676	0.047397	0.048003
342	0.011686	0.004929	0.010231	0.012383	0.008038	0.013039	0.020957	0.015738	0.024891	0.048116	0.047926

Absorption intensity											
**Wavelength (nm)**	**concentration of α-methylbutyrylshikon (M)**										
	A	*B*	*C*	*D*	*E*	*F*	*G*	*I*	*J*	*K*	*L*
	0.00	$8.0x10^{-7}$	$1.6x10^{-6}$	$2.0x10^{-6}$	$2.8x10^{-6}$	$4.0x10^{-6}$	$4.8x10^{-6}$	$6.0x10^{-6}$	$8.0x10^{-6}$	$1.2x10^{-5}$	$1.6x10^{-5}$
341	0.011381	0.004983	0.0111	0.012929	0.008256	0.014003	0.021261	0.016047	0.02558	0.048149	0.048956
340	0.012093	0.005297	0.011381	0.012336	0.008803	0.013931	0.021679	0.016394	0.025686	0.048752	0.049097
339	0.012595	0.005403	0.011664	0.013756	0.009278	0.014685	0.022255	0.01739	0.026599	0.049951	0.049872
338	0.012107	0.005137	0.011306	0.013316	0.008494	0.014188	0.022309	0.016918	0.026114	0.050461	0.050608
337	0.012242	0.005111	0.01098	0.013646	0.008784	0.014644	0.022123	0.017057	0.026589	0.050741	0.05086
336	0.012344	0.004579	0.011255	0.013967	0.008944	0.014338	0.022728	0.01692	0.026742	0.051192	0.051357
335	0.012854	0.00512	0.011486	0.014216	0.261599	0.014487	0.022788	0.017333	0.02718	0.052144	0.051878
334	0.012999	0.005088	0.01165	0.014088	0.009559	0.01508	0.023365	0.017514	0.028134	0.052587	0.052969
333	0.013044	0.005568	0.011808	0.014633	0.009719	0.014879	0.023697	0.017909	0.028222	0.053366	0.054342
332	0.012623	0.005366	0.011325	0.013877	0.009154	0.015335	0.023504	0.017719	0.028392	0.053623	0.054513
331	0.013264	0.005835	0.012094	0.015017	0.00984	0.016071	0.024397	0.018379	0.029291	0.054887	0.055407
330	0.013854	0.006025	0.012067	0.014867	0.009939	0.015982	0.024611	0.018691	0.029572	0.056128	0.05669
329	0.013836	0.005574	0.01217	0.015421	0.009965	0.015517	0.02462	0.018843	0.029724	0.056281	0.056621
328	0.013957	0.005623	0.012324	0.015943	0.009883	0.016703	0.025397	0.019597	0.030661	0.056688	0.057744
327	0.014519	0.006199	0.01251	0.015706	0.010612	0.016275	0.025649	0.019687	0.031439	0.057894	0.058725
326	0.014323	0.005985	0.012348	0.01553	0.010161	0.01658	0.025635	0.019805	0.031299	0.058295	0.059256
325	0.01469	0.006518	0.012479	0.016703	0.010633	0.017064	0.026412	0.020349	0.03187	0.059732	0.060298
324	0.014523	0.006093	0.012366	0.016357	0.010502	0.017425	0.026884	0.020138	0.032134	0.06011	0.061061
323	0.015233	0.006527	0.013006	0.016425	0.011078	0.017832	0.027099	0.021121	0.032937	0.061342	0.062131
322	0.014798	0.006624	0.01266	0.01681	0.010929	0.017378	0.026907	0.02092	0.032974	0.06169	0.063061
321	0.015138	0.006579	0.012822	0.017122	0.011252	0.018053	0.027539	0.021617	0.034008	0.062794	0.06422
320	0.015081	0.006357	0.01305	0.017549	0.011233	0.018138	0.027874	0.021815	0.034761	0.064058	0.065367
319	0.015678	0.006412	0.013394	0.017365	0.011645	0.018772	0.028342	0.022132	0.034933	0.064754	0.066109
318	0.015552	0.006659	0.013291	0.017412	0.011649	0.018136	0.028454	0.022284	0.035628	0.065516	0.066869
317	0.016417	0.007112	0.013853	0.018254	0.012171	0.019453	0.029386	0.022834	0.036148	0.066672	0.068214
316	0.016928	0.006826	0.013874	0.018557	0.01249	0.019631	0.02945	0.023302	0.036067	0.06809	0.069083
315	0.016372	0.006802	0.013768	0.018404	0.012179	0.019922	0.02982	0.023865	0.03731	0.06866	0.070434
314	0.017045	0.007506	0.014116	0.019013	0.012405	0.020275	0.030455	0.024339	0.037363	0.069969	0.071955
313	0.017428	0.007671	0.013986	0.019322	0.01243	0.020439	0.030611	0.024399	0.038284	0.070925	0.072706
312	0.01712	0.007278	0.014282	0.018811	0.01223	0.02059	0.03079	0.024609	0.038926	0.071568	0.07391
311	0.017609	0.007703	0.014127	0.019833	0.013116	0.020717	0.031343	0.025264	0.039264	0.072475	0.07521
310	0.018346	0.007986	0.015208	0.020329	0.013548	0.021319	0.032226	0.025681	0.040201	0.074287	0.07684
309	0.01797	0.007716	0.01462	0.020305	0.013097	0.021594	0.032431	0.026058	0.040086	0.074944	0.0779

EXCLI Journal 2020;19:48-70 – ISSN 1611-2156
Received: October 03, 2019, accepted: December 19, 2019, published: January 03, 2020

Absorption intensity											
**Wavelength (nm)**	**concentration of α-methylbutyrylshikon (M)**										
	A	*B*	*C*	*D*	*E*	*F*	*G*	*I*	*J*	*K*	*L*
	0.00	$8.0x10^{-7}$	$1.6x10^{-6}$	$2.0x10^{-6}$	$2.8x10^{-6}$	$4.0x10^{-6}$	$4.8x10^{-6}$	$6.0x10^{-6}$	$8.0x10^{-6}$	$1.2x10^{-5}$	$1.6x10^{-5}$
308	0.01892	0.008161	0.014926	0.020886	0.013712	0.021854	0.033102	0.026889	0.041609	0.076859	0.079441
307	0.01895	0.008404	0.01528	0.021354	0.014106	0.022466	0.033956	0.027389	0.041848	0.077814	0.080126
306	0.019517	0.008013	0.015267	0.02112	0.013716	0.022457	0.033865	0.027247	0.042202	0.078971	0.081394
305	0.019691	0.008512	0.015313	0.021604	0.013819	0.023179	0.034598	0.027868	0.043081	0.080799	0.083108
304	0.020119	0.008786	0.015858	0.021821	0.014784	0.023515	0.03498	0.028742	0.043688	0.0817	0.084758
303	0.020126	0.008778	0.01585	0.021779	0.014476	0.023758	0.035632	0.028928	0.044474	0.082378	0.085548
302	0.02058	0.00872	0.015775	0.021939	0.01491	0.023625	0.035854	0.029521	0.044811	0.083637	0.086572
301	0.020925	0.00902	0.015829	0.022331	0.014619	0.023972	0.036307	0.029335	0.045397	0.084595	0.08786
300	0.021628	0.009436	0.016424	0.022783	0.015447	0.0246	0.037082	0.030279	0.046	0.08629	0.089854
299	0.021943	0.00999	0.01667	0.023582	0.016059	0.025273	0.037341	0.030775	0.046807	0.087096	0.090671
298	0.022091	0.00943	0.016388	0.023333	0.015741	0.025303	0.037906	0.031124	0.047252	0.088008	0.091755
297	0.02215	0.009555	0.0167	0.023391	0.015566	0.025077	0.038252	0.031168	0.047793	0.088736	0.09288
296	0.022732	0.010042	0.016648	0.02411	0.016156	0.025742	0.038671	0.031999	0.048867	0.090683	0.094177
295	0.023384	0.00992	0.0171	0.02405	0.016435	0.025459	0.039282	0.032531	0.049385	0.091645	0.095471
294	0.023474	0.0102	0.016707	0.023779	0.016377	0.026265	0.039814	0.032645	0.049767	0.092049	0.096341
293	0.023826	0.010263	0.017212	0.024511	0.016712	0.026291	0.040019	0.032683	0.050532	0.093508	0.097897
292	0.024707	0.011068	0.017771	0.025121	0.017182	0.026892	0.040847	0.033788	0.051735	0.09528	0.099412
291	0.024821	0.010505	0.017463	0.024613	0.017076	0.026947	0.040988	0.03388	0.051308	0.096081	0.10031
290	0.025099	0.011187	0.018055	0.02513	0.017175	0.027134	0.041659	0.034192	0.052153	0.096866	0.101633
289	0.025758	0.01167	0.018382	0.025876	0.017788	0.027854	0.042293	0.034676	0.053337	0.098478	0.102905
288	0.02583	0.011483	0.018117	0.025477	0.018031	0.027728	0.042479	0.035209	0.0534	0.099023	0.103914
287	0.026225	0.011451	0.017985	0.025873	0.018302	0.027917	0.042888	0.035483	0.053846	0.100231	0.105066
286	0.026579	0.011742	0.01845	0.026111	0.018427	0.028379	0.043397	0.036105	0.054729	0.101409	0.106192
285	0.02681	0.012076	0.018309	0.026074	0.018267	0.028591	0.04339	0.036451	0.05498	0.101958	0.107248
284	0.027291	0.012316	0.018668	0.026724	0.018708	0.028973	0.044188	0.036812	0.055876	0.103327	0.108459
283	0.027695	0.01233	0.018839	0.026622	0.018749	0.028937	0.044561	0.037007	0.056501	0.104345	0.109559
282	0.027279	0.012114	0.018632	0.026614	0.01896	0.02914	0.044873	0.036983	0.056509	0.104803	0.110013
281	0.028214	0.012545	0.019636	0.027286	0.01966	0.029504	0.045544	0.03792	0.057706	0.106484	0.11166
280	0.028536	0.01292	0.019672	0.027612	0.019947	0.030166	0.04606	0.038369	0.058162	0.107212	0.112851
279	0.028352	0.012945	0.01978	0.027461	0.019925	0.030112	0.046224	0.038427	0.058758	0.107782	0.113954
278	0.02874	0.013125	0.019803	0.027606	0.019963	0.030678	0.046621	0.039037	0.059502	0.109057	0.114897
277	0.028866	0.013163	0.020137	0.027746	0.02035	0.030466	0.046687	0.039235	0.059864	0.110015	0.11597
276	0.028868	0.013119	0.020147	0.028055	0.020423	0.03092	0.047426	0.039786	0.060558	0.110959	0.117358

EXCLI Journal 2020;19:48-70 – ISSN 1611-2156
Received: October 03, 2019, accepted: December 19, 2019, published: January 03, 2020

Absorption intensity											
**Wavelength (nm)**	**concentration of α-methylbutyrylshikon (M)**										
	A	*B*	*C*	*D*	*E*	*F*	*G*	*I*	*J*	*K*	*L*
	0.00	$8.0x10^{-7}$	$1.6x10^{-6}$	$2.0x10^{-6}$	$2.8x10^{-6}$	$4.0x10^{-6}$	$4.8x10^{-6}$	$6.0x10^{-6}$	$8.0x10^{-6}$	$1.2x10^{-5}$	$1.6x10^{-5}$
275	0.028893	0.013131	0.01991	0.027992	0.020517	0.030937	0.04787	0.04023	0.061109	0.11183	0.118443
274	0.029262	0.013683	0.020726	0.028768	0.021064	0.031843	0.048514	0.041136	0.06219	0.113335	0.120605
273	0.029479	0.013707	0.020863	0.028614	0.021391	0.032069	0.048653	0.041597	0.062883	0.114563	0.121634
272	0.02992	0.013672	0.021003	0.028785	0.021628	0.032756	0.049817	0.04214	0.063455	0.115792	0.123111
271	0.030027	0.014049	0.021274	0.02959	0.021859	0.032702	0.049829	0.042695	0.064361	0.117345	0.124864
270	0.029748	0.014116	0.021433	0.029794	0.022439	0.033403	0.050501	0.043393	0.065322	0.118767	0.126643
269	0.030174	0.01423	0.021664	0.030017	0.023101	0.033875	0.051312	0.04436	0.066662	0.120644	0.129089
268	0.029933	0.014166	0.021787	0.030249	0.023213	0.034093	0.051502	0.044953	0.067542	0.122215	0.131144
267	0.03014	0.014271	0.022262	0.030796	0.023577	0.034764	0.052852	0.046063	0.068869	0.124288	0.133424
266	0.030557	0.014761	0.022634	0.031229	0.024213	0.03569	0.05366	0.047254	0.07064	0.126984	0.136898
265	0.030212	0.014955	0.02304	0.031997	0.024637	0.036675	0.054658	0.048329	0.072066	0.129461	0.13987
264	0.030336	0.014947	0.023563	0.032668	0.025501	0.037485	0.055857	0.049582	0.073946	0.13209	0.143234
263	0.030231	0.015164	0.023946	0.033091	0.026343	0.038354	0.056566	0.051	0.075852	0.135145	0.146895
262	0.030557	0.015471	0.024339	0.033842	0.027146	0.03956	0.058275	0.052559	0.077931	0.138474	0.151092
261	0.030713	0.015445	0.024541	0.034674	0.027599	0.040548	0.059492	0.054172	0.080065	0.14194	0.155307
260	0.030728	0.0159	0.025354	0.035665	0.028825	0.041754	0.061258	0.056181	0.082647	0.145913	0.160129
259	0.031166	0.015978	0.02589	0.036248	0.030029	0.043218	0.062889	0.057802	0.085323	0.14998	0.165393
258	0.031358	0.016846	0.026866	0.037807	0.031441	0.045237	0.06503	0.060634	0.088757	0.154841	0.171499
257	0.031339	0.017006	0.027519	0.038774	0.032621	0.046778	0.066934	0.063134	0.0921	0.159988	0.177972
256	0.031491	0.01741	0.028312	0.040208	0.033586	0.048591	0.069285	0.066	0.095947	0.165582	0.185341
255	0.031538	0.017901	0.02929	0.041609	0.035342	0.050951	0.071796	0.069312	0.100569	0.172328	0.193954
254	0.031386	0.017902	0.030015	0.042947	0.03695	0.053288	0.074562	0.072956	0.105481	0.179665	0.203153
253	0.032064	0.018986	0.031531	0.045127	0.039325	0.056414	0.078482	0.077574	0.111344	0.188968	0.214564
252	0.032261	0.019637	0.033012	0.046943	0.041539	0.059415	0.082169	0.081988	0.117795	0.198433	0.22682
251	0.03271	0.020302	0.034708	0.049661	0.044542	0.063445	0.086598	0.087579	0.125512	0.209732	0.241223
250	0.03279	0.021079	0.036276	0.051975	0.047216	0.067291	0.091326	0.093916	0.13361	0.222028	0.256763
249	0.032886	0.022168	0.03841	0.054734	0.050815	0.072052	0.097066	0.100939	0.143408	0.236526	0.275861
248	0.033437	0.023024	0.040845	0.058091	0.055163	0.07806	0.103626	0.10974	0.154997	0.253863	0.297997
247	0.034275	0.025215	0.044137	0.062764	0.060184	0.08497	0.112088	0.120297	0.168977	0.274667	0.324855
246	0.034642	0.026589	0.04768	0.067142	0.066084	0.093257	0.1214	0.132543	0.185364	0.299228	0.357103
245	0.035478	0.028885	0.052043	0.073493	0.073611	0.103609	0.133188	0.148449	0.205733	0.330126	0.39775
244	0.035827	0.031341	0.05729	0.080467	0.083054	0.116276	0.147816	0.167566	0.231593	0.369028	0.448512
243	0.036326	0.034471	0.064341	0.089794	0.094968	0.13272	0.166283	0.192804	0.265052	0.41928	0.514193

EXCLI Journal 2020;19:48-70 – ISSN 1611-2156
Received: October 03, 2019, accepted: December 19, 2019, published: January 03, 2020

Wavelength (nm)	***Absorption intensity***										
	**concentration of α-methylbutyrylshikon (M)**										
	A	*B*	*C*	*D*	*E*	*F*	*G*	*I*	*J*	*K*	*L*
	0.00	$8.0x10^{-7}$	$1.6x10^{-6}$	$2.0x10^{-6}$	$2.8x10^{-6}$	$4.0x10^{-6}$	$4.8x10^{-6}$	$6.0x10^{-6}$	$8.0x10^{-6}$	$1.2x10^{-5}$	$1.6x10^{-5}$
242	0.037038	0.038789	0.073552	0.102	0.110431	0.154503	0.191148	0.225824	0.308734	0.484356	0.600145
241	0.03799	0.044214	0.086149	0.118144	0.131971	0.183788	0.223882	0.270861	0.367454	0.57205	0.715021
240	0.039094	0.051961	0.10347	0.140316	0.161005	0.223312	0.268628	0.331462	0.446607	0.68895	0.867908
239	0.039818	0.062437	0.126749	0.170189	0.200463	0.278022	0.329761	0.414368	0.554721	0.84703	1.072937
238	0.040616	0.076634	0.159102	0.211191	0.255528	0.352447	0.413541	0.527516	0.699789	1.058331	1.344371
237	0.042097	0.097236	0.204554	0.26924	0.331819	0.455887	0.52897	0.682318	0.89836	1.341386	1.704158
236	0.043654	0.12586	0.267509	0.348493	0.437161	0.597082	0.685793	0.89136	1.163414	1.712965	2.167712
235	0.045414	0.164993	0.354788	0.457925	0.581123	0.789024	0.898163	1.171264	1.511898	2.19103	2.745156
234	0.047304	0.219237	0.474207	0.606082	0.774878	1.044734	1.180197	1.538316	1.964843	2.789234	3.415629
233	0.049516	0.29447	0.635682	0.805909	1.034568	1.381654	1.549523	2.008734	2.534549	3.456255	3.955588
232	0.052474	0.395334	0.851903	1.069165	1.372958	1.817387	2.022923	2.597025	3.193355	3.960741	4.257185
231	0.056048	0.532354	1.135615	1.413144	1.807973	2.361282	2.608562	3.250955	3.757592	4.229599	4.463652
230	0.06053	0.713025	1.500651	1.85033	2.351363	2.998365	3.255291	3.762657	4.08051	4.446676	4.600076
229	0.066454	0.948647	1.962602	2.397222	2.984504	3.599764	3.783841	4.064929	4.299298	4.579648	4.833808
228	0.073389	1.249692	2.530075	3.023343	3.568652	3.941874	4.073914	4.215128	4.44102	4.695044	4.881345
227	0.082141	1.630219	3.150126	3.5882	3.912672	4.152328	4.26616	4.387807	4.570608	4.811844	4.901384
226	0.092786	2.098511	3.679486	3.926287	4.109106	4.299622	4.391284	4.533691	4.72168	4.898835	5.204142
225	0.106883	2.649681	3.950736	4.10563	4.297762	4.47155	4.581357	4.611723	4.850034	5.053899	5.183201
224	0.123824	3.239927	4.159302	4.300529	4.44596	4.561124	4.660881	4.698497	4.92403	4.92391	5.013981
223	0.144599	3.698575	4.291384	4.447254	4.483927	4.6597	4.749985	4.723515	4.949922	5.092264	5.024351
222	0.170334	3.962106	4.378939	4.514871	4.673804	4.754391	4.845287	4.876671	5.001705	5.076971	5.30886
221	0.200813	4.096882	4.500057	4.646598	4.655367	4.80489	4.809134	4.949892	5.269183	5.140914	5.414902
220	0.236657	4.286068	4.671651	4.818747	4.769616	4.832532	4.924818	4.968879	5.128496	5.118841	5.109556
219	0.277615	4.413021	4.621923	4.745594	4.853373	4.918946	5.012914	4.841969	4.996614	4.881194	5.514296
218	0.324144	4.445864	4.648059	4.718092	4.736174	4.829108	5.102119	5.04111	5.112894	5.060318	5.800506
217	0.375157	4.539652	4.700862	4.700842	4.784247	5.015917	4.866886	4.81994	5.025329	5.127216	5.167176
216	0.43044	4.589721	4.696661	4.862388	4.797145	4.817545	5.10473	5.029812	5.162339	5.090755	5.104151
215	0.48847	4.817172	4.896063	4.818378	4.990109	4.859437	4.990133	4.967016	5.195875	5.14255	5.177196
214	0.547227	4.607341	4.936671	4.659761	4.756623	5.142478	4.972925	5.181684	5.600963	5.462408	10
213	0.606551	4.610386	4.859788	4.959441	5.220139	5.672119	5.149133	5.127263	5.245255	10	5.494881
212	0.665299	4.930456	5.354463	4.82736	4.851568	4.948112	4.890219	5.491316	5.917424	5.792065	10
211	0.71148	4.824073	4.821861	4.771995	4.924706	5.060546	5.494301	4.923785	5.037684	5.162319	5.560506
210	0.755709	4.937213	5.233347	5.443851	10	10	10	10	10	10	10

EXCLI Journal 2020;19:48-70 – ISSN 1611-2156
Received: October 03, 2019, accepted: December 19, 2019, published: January 03, 2020

Absorption intensity											
**Wavelength (nm)**	**concentration of α-methylbutyrylshikon (M)**										
	A	*B*	*C*	*D*	*E*	*F*	*G*	*I*	*J*	*K*	*L*
	0.00	$8.0x10^{-7}$	$1.6x10^{-6}$	$2.0x10^{-6}$	$2.8x10^{-6}$	$4.0x10^{-6}$	$4.8x10^{-6}$	$6.0x10^{-6}$	$8.0x10^{-6}$	$1.2x10^{-5}$	$1.6x10^{-5}$
209	0.789838	4.609134	5.601531	4.987496	5.300737	5.174777	10	10	10	10	10
208	0.815979	4.761627	4.729502	4.606461	4.901498	4.923581	5.093925	5.092529	4.985572	5.074979	5.128195
207	0.829612	4.530591	4.973493	4.767044	4.620809	4.756221	4.850497	4.970164	5.055745	4.804561	10
206	0.838372	4.599471	4.640162	4.677423	4.717485	4.93083	5.181735	4.863775	4.879621	5.004202	4.848727
205	0.833498	5.028859	5.366153	5.013347	10	5.03644	5.035663	10	10	10	10
204	0.831946	4.285131	5.078649	4.852486	4.593724	4.886053	5.462179	10	5.510708	5.267742	10
203	0.826342	4.517328	4.33767	4.391688	4.379986	4.376959	4.602442	5.349849	4.683738	4.394969	10
202	0.82316	3.871326	4.1668	3.999378	4.205573	3.884262	4.454732	10	4.33198	5.210792	5.533957
201	0.789697	3.4528	3.539756	3.474044	3.484585	3.579777	3.879521	3.679053	3.629205	3.722429	3.646401
200	0.797277	3.018141	3.249737	2.86275	3.046617	2.967701	3.08756	3.093437	3.120656	2.98169	3.144303

Table 6: Absorption intensity in the wavelength range of 200-800 nm for absorption spectra of different concentration of acetylshikon (*A*-*L*)

Absorption intensity											
**Wavelength (nm)**	**concentration of acetylshikonin (M)**										
	A	*B*	*C*	*D*	*E*	*F*	*G*	*I*	*J*	*K*	*L*
	0.00	8.0x10^{-7}	1.6x10^{-6}	2.0x10^{-6}	2.8x10^{-6}	4.0x10^{-6}	4.8x10^{-6}	6.0x10^{-6}	8.0x10^{-6}	1.2x10^{-5}	1.6x10^{-5}
800	0.000995	-3.47E-06	0.001038	0.00063	-0.00045	0.000382	0.000237	0.000152	0.000885	0.002264	0.000856
799	0.000844	-0.00047	0.000898	0.000381	-0.00051	0.000334	0.000252	-0.00018	0.000991	0.001554	0.000635
798	0.000699	-0.00083	0.000653	0.000215	-0.00081	-0.00018	3.55E-05	-0.00011	0.000576	0.001557	0.000411
797	0.000992	-0.0001	0.001081	0.000756	-0.00032	0.000524	0.000537	6.55E-05	0.00129	0.002058	0.000867
796	0.001085	-0.00042	0.001311	0.000638	-0.00052	0.000354	0.000255	0.00013	0.00098	0.001916	0.000372
795	0.000623	-0.00061	0.000788	0.000266	-0.0007	-4.21E-05	-0.00033	-0.00023	0.000621	0.001786	0.0004
794	0.001354	3.74E-05	0.001175	0.000714	-0.00038	0.000559	0.00052	0.000168	0.001522	0.002169	0.001058
793	0.00139	-0.0001	0.00125	0.000611	-0.00059	0.000451	0.000393	0.000248	0.001111	0.001988	0.001004
792	0.000636	-0.00023	0.000825	0.000375	-0.00093	-7.01E-05	-0.00018	-0.00011	0.000995	0.001405	0.0005
791	0.000955	-0.00026	0.000829	0.000616	-0.00055	0.000126	6.27E-05	-3.42E-05	0.001129	0.001607	0.000663
790	0.000999	-0.00019	0.000558	0.000289	-0.0004	0.000211	-0.00016	0.00021	0.000998	0.00187	0.000781
789	0.000478	-0.00062	0.000649	9.20E-05	-0.0009	8.49E-05	-0.00027	-0.00036	0.000794	0.00175	0.000765
788	0.00101	-0.00036	0.000848	0.000378	-0.00072	0.000396	0.000197	-0.0002	0.000863	0.001749	0.000902
787	0.000923	-0.0002	0.000848	0.000728	-0.00037	9.84E-05	0.000442	5.36E-05	0.001003	0.001789	0.000793
786	0.001541	0.00021	0.001206	0.000877	-0.00041	0.000413	0.000353	-0.0001	0.00122	0.001884	0.000882
785	0.000951	-0.00027	0.00083	0.000348	-0.00076	5.31E-05	9.65E-05	0.000202	0.000796	0.00179	0.000825
784	0.000957	-0.0004	0.000947	0.000138	-0.00073	0.000178	0.000213	-1.88E-05	0.000954	0.001725	0.000605
783	0.001046	-0.00019	0.000576	0.00032	-0.00067	0.00026	0.000271	-2.69E-06	0.001097	0.00173	0.000724
782	0.0011	-0.00027	0.000805	0.000526	-0.00059	0.000292	0.000182	-5.81E-05	0.001225	0.001912	0.000837
781	0.000952	-0.00019	0.000906	0.000574	-0.00075	0.000395	0.000149	6.15E-05	0.000869	0.001814	0.000724
780	0.000724	-0.00033	0.00073	0.000186	-0.00084	-0.00011	-0.00011	-0.00047	0.000771	0.001554	0.00042
779	0.001083	-0.00022	0.001074	0.000466	-0.00065	0.000479	0.000179	5.14E-05	0.001116	0.001883	0.00107
778	0.001033	-0.00035	0.000727	0.00022	-0.0008	0.000165	0.000204	-3.16E-05	0.001183	0.001809	0.000917
777	0.001161	0.00012	0.000936	0.000467	-0.00038	0.000378	0.00051	7.62E-05	0.001225	0.002044	0.001027
776	0.00085	-0.00011	0.001086	0.000396	-0.00048	0.000269	-3.42E-06	0.000108	0.00082	0.001819	0.000933
775	0.001207	-0.00042	0.00097	0.000713	-0.00081	0.000146	-7.61E-05	-0.00015	0.000899	0.001567	0.000763
774	0.001174	-9.60E-05	0.001121	0.0004	-0.0006	0.000168	0.000156	2.90E-05	0.001155	0.001912	0.000918
773	0.000747	-0.00045	0.000703	0.000189	-0.00095	0.000106	-1.58E-05	-0.00046	0.000491	0.001686	0.000512
772	0.001216	-0.00017	0.00109	0.000441	-0.00053	0.000392	3.50E-05	7.05E-05	0.000967	0.001938	0.001068
771	0.001029	-0.00023	0.000857	0.000513	-0.00074	0.000205	3.34E-05	-0.00024	0.000984	0.001678	0.000908
770	0.001123	-0.00014	0.001018	0.00064	-0.00068	0.000341	0.000175	4.19E-05	0.001144	0.001846	0.000817

EXCLI Journal 2020;19:48-70 – ISSN 1611-2156
Received: October 03, 2019, accepted: December 19, 2019, published: January 03, 2020

Absorption intensity											
Wavelength (nm)	concentration of acetylshikonin (M)										
	A	B	C	D	E	F	G	I	J	K	L
	0.00	$8.0x10^{-7}$	$1.6x10^{-6}$	$2.0x10^{-6}$	$2.8x10^{-6}$	$4.0x10^{-6}$	$4.8x10^{-6}$	$6.0x10^{-6}$	$8.0x10^{-6}$	$1.2x10^{-5}$	$1.6x10^{-5}$
769	0.001222	-0.00026	0.001188	0.000399	-0.00065	0.000349	0.000354	0.000195	0.001349	0.00206	0.000884
768	0.001082	-0.00013	0.000982	0.000357	-0.00043	0.000497	0.000376	7.69E-05	0.000928	0.002135	0.001061
767	0.000838	-0.00039	0.001129	0.000341	-0.00078	0.000223	0.000206	-2.76E-05	0.001018	0.001904	0.000573
766	0.001216	-0.00019	0.001085	0.000465	-0.00056	0.000577	0.000273	3.11E-07	0.001178	0.002058	0.000885
765	0.001437	6.75E-05	0.001147	0.000716	-0.00039	0.000452	0.000359	0.000149	0.001405	0.002069	0.001134
764	0.001231	-0.00012	0.000948	0.00067	-0.00048	0.000626	0.000413	5.47E-05	0.001249	0.001905	0.001156
763	0.000886	-0.00035	0.000898	0.000468	-0.00072	0.000436	0.00018	-0.00016	0.001139	0.001869	0.001123
762	0.000956	-0.00055	0.000745	0.000357	-0.00103	0.00017	3.27E-05	-0.00025	0.000943	0.001873	0.000849
761	0.001079	-0.0003	0.00088	0.000583	-0.00041	0.000426	0.000208	0.000131	0.00111	0.002231	0.001116
760	0.001182	-0.00016	0.001248	0.000696	-0.00067	0.00056	0.000363	0.000212	0.001279	0.002164	0.001209
759	0.001011	-0.00035	0.000985	0.000405	-0.0006	0.000414	0.000122	0.000164	0.001172	0.002101	0.000839
758	0.001196	-0.00034	0.001033	0.000437	-0.00057	0.000387	0.000185	0.000138	0.001361	0.002267	0.001189
757	0.000909	-0.00027	0.00086	0.00052	-0.00089	0.000229	-2.42E-05	-0.00014	0.001055	0.001945	0.001144
756	0.001194	-0.00022	0.001178	0.000708	-0.00058	0.00043	0.00023	0.000185	0.001321	0.002091	0.001155
755	0.000821	-0.00043	0.000693	0.000191	-0.00068	0.000413	0.000171	3.73E-05	0.0012	0.001741	0.001165
754	0.001092	-0.00024	0.001113	0.000615	-0.0006	0.000408	0.000261	-6.71E-05	0.001235	0.001998	0.001076
753	0.001144	-0.00013	0.001152	0.00078	-0.00025	0.000541	0.00022	-5.18E-07	0.001305	0.002149	0.0013
752	0.001061	-0.00029	0.001004	0.000607	-0.00046	0.000596	0.000242	0.00011	0.001287	0.001901	0.001094
751	0.001183	-5.20E-05	0.001284	0.000754	-0.00048	0.000661	0.000459	0.000174	0.001429	0.002212	0.00154
750	0.001116	-0.00027	0.001001	0.000627	-0.00062	0.00042	0.000277	2.58E-05	0.001268	0.001923	0.001001
749	0.000902	-0.00032	0.001005	0.000428	-0.00069	0.000373	0.000207	6.12E-05	0.001305	0.001986	0.001188
748	0.001118	-0.00023	0.001038	0.000754	-0.00044	0.000567	0.000345	4.91E-05	0.001382	0.002139	0.001217
747	0.000766	-0.00043	0.000862	0.000272	-0.00081	0.000212	0.000137	-0.00026	0.001155	0.001803	0.000912
746	0.001194	-0.00029	0.000832	0.000475	-0.00054	0.00048	0.000311	0.000208	0.001399	0.002159	0.001378
745	0.000853	-0.00036	0.000866	0.000378	-0.00086	0.000509	0.000172	-8.48E-05	0.001105	0.002003	0.001092
744	0.001085	-0.00046	0.000897	0.000315	-0.00085	0.000473	-6.52E-06	-0.00018	0.001192	0.001974	0.000965
743	0.001035	-0.00034	0.000849	0.000441	-0.00069	0.000414	0.000221	-0.00018	0.001264	0.002055	0.001021
742	0.00117	-0.00024	0.001038	0.000406	-0.00059	0.000455	0.000294	7.68E-05	0.001325	0.002081	0.001407
741	0.001182	-8.19E-05	0.000876	0.000515	-0.00072	0.000407	0.000306	-1.75E-05	0.001331	0.002152	0.001397
740	0.000965	-0.00032	0.000964	0.000447	-0.00087	0.000258	4.43E-05	5.24E-05	0.001332	0.002014	0.001217
739	0.001018	-0.00015	0.001048	0.000546	-0.00068	0.000415	0.000281	0.000114	0.001353	0.002224	0.001394
738	0.001142	-8.80E-05	0.001285	0.000467	-0.00053	0.000761	0.000467	0.000511	0.001707	0.002563	0.001718
737	0.001064	-0.00017	0.00112	0.000593	-0.00067	0.000599	0.000366	0.00029	0.001613	0.002261	0.00172

EXCLI Journal 2020;19:48-70 – ISSN 1611-2156
Received: October 03, 2019, accepted: December 19, 2019, published: January 03, 2020

Absorption intensity											
**Wavelength (nm)**	**concentration of acetylshikonin (M)**										
	A	*B*	*C*	*D*	*E*	*F*	*G*	*I*	*J*	*K*	*L*
	0.00	$8.0x10^{-7}$	$1.6x10^{-6}$	$2.0x10^{-6}$	$2.8x10^{-6}$	$4.0x10^{-6}$	$4.8x10^{-6}$	$6.0x10^{-6}$	$8.0x10^{-6}$	$1.2x10^{-5}$	$1.6x10^{-5}$
736	0.0007	-0.00049	0.000973	0.000167	-0.0008	0.000281	0.000189	-0.00019	0.001202	0.002025	0.001456
735	0.001026	-0.00019	0.001066	0.000551	-0.0007	0.000702	0.00037	0.000264	0.001428	0.002498	0.001781
734	0.00089	-0.00042	0.00104	0.000356	-0.0008	0.000401	0.000276	0.000244	0.001574	0.002386	0.001641
733	0.001232	-4.32E-05	0.001059	0.000499	-0.00066	0.000599	0.000453	0.000359	0.001664	0.002531	0.002037
732	0.00096	-0.00036	0.001145	0.000674	-0.00064	0.000478	0.000419	0.000244	0.001758	0.002626	0.002152
731	0.001176	-0.00028	0.001057	0.000505	-0.00052	0.000531	0.000378	0.000245	0.001705	0.002544	0.001993
730	0.00121	-0.00034	0.001189	0.000606	-0.00058	0.000804	0.00039	0.000411	0.001844	0.002921	0.00234
729	0.000988	-0.00038	0.001037	0.000587	-0.00058	0.000807	0.000459	0.000471	0.001831	0.002756	0.002427
728	0.001238	-8.92E-05	0.001343	0.000744	-0.00052	0.000748	0.000448	0.000469	0.001893	0.002951	0.00247
727	0.00114	-0.00012	0.001175	0.000567	-0.00031	0.000854	0.000559	0.000419	0.00205	0.002939	0.002622
726	0.001136	-0.00028	0.001452	0.000677	-0.00058	0.000922	0.000691	0.000517	0.002007	0.003003	0.002757
725	0.000948	-0.00044	0.000952	0.000516	-0.00059	0.000654	0.000417	0.000408	0.001931	0.002859	0.002576
724	0.001206	-7.70E-05	0.001233	0.000665	-0.00042	0.000827	0.000663	0.000594	0.002034	0.003132	0.002662
723	0.001194	-0.00015	0.001286	0.000766	-0.00042	0.000926	0.000695	0.00062	0.002163	0.003117	0.002688
722	0.001216	-7.42E-05	0.001251	0.000922	-0.00026	0.000994	0.00087	0.000683	0.002154	0.003321	0.002937
721	0.001125	-0.00016	0.001292	0.000568	-0.00056	0.000944	0.000577	0.000567	0.002103	0.003163	0.002865
720	0.001218	-0.00028	0.001339	0.00077	-0.00035	0.000927	0.00077	0.000583	0.002065	0.003207	0.002996
719	0.001171	-0.00019	0.001338	0.000831	-0.00042	0.000962	0.000635	0.000668	0.00216	0.003198	0.003054
718	0.000949	-0.00025	0.001124	0.00063	-0.00045	0.000857	0.000437	0.000395	0.002005	0.003295	0.002864
717	0.001226	-0.00011	0.001216	0.000644	-0.00037	0.000996	0.00065	0.0006	0.002288	0.003392	0.003112
716	0.00123	-9.86E-05	0.001222	0.00059	-0.00044	0.000926	0.000651	0.000539	0.002193	0.003428	0.002966
715	0.001256	-8.10E-05	0.001356	0.000786	-0.00027	0.001058	0.000791	0.000678	0.002413	0.00351	0.003278
714	0.00122	-4.80E-05	0.001322	0.000721	-0.00036	0.001087	0.000833	0.000881	0.002367	0.003596	0.003385
713	0.001198	-6.36E-05	0.001328	0.000721	-0.00042	0.000968	0.000717	0.000814	0.002285	0.003522	0.003307
712	0.001271	-0.00036	0.001363	0.000753	-0.00053	0.000989	0.000584	0.000691	0.002384	0.003473	0.003289
711	0.001247	-8.42E-05	0.001449	0.000653	-0.0003	0.001161	0.0008	0.00074	0.002468	0.003564	0.003504
710	0.001196	-0.00011	0.001516	0.000814	-0.00039	0.000904	0.000757	0.000832	0.002485	0.0038	0.003557
709	0.001136	4.59E-05	0.00132	0.000755	-0.0003	0.001318	0.000839	0.000773	0.00248	0.00389	0.003696
708	0.001052	-0.00015	0.001443	0.000762	-0.00036	0.001034	0.000765	0.000698	0.00245	0.003777	0.003874
707	0.001324	6.37E-05	0.001326	0.000748	-0.00026	0.001007	0.000833	0.000955	0.002466	0.003745	0.003881
706	0.001315	2.38E-05	0.001647	0.000885	-0.00022	0.001251	0.000867	0.000967	0.002857	0.004069	0.003954
705	0.001418	-7.75E-05	0.001588	0.000977	-0.00018	0.001368	0.000922	0.001008	0.002665	0.004031	0.004038
704	0.001372	-0.00012	0.001486	0.000774	-0.00027	0.001293	0.001089	0.001071	0.002637	0.003999	0.004182

EXCLI Journal 2020;19:48-70 – ISSN 1611-2156
Received: October 03, 2019, accepted: December 19, 2019, published: January 03, 2020

Absorption intensity											
**Wavelength (nm)**	**concentration of acetylshikonin (M)**										
	A	*B*	*C*	*D*	*E*	*F*	*G*	*I*	*J*	*K*	*L*
	0.00	$8.0x10^{-7}$	$1.6x10^{-6}$	$2.0x10^{-6}$	$2.8x10^{-6}$	$4.0x10^{-6}$	$4.8x10^{-6}$	$6.0x10^{-6}$	$8.0x10^{-6}$	$1.2x10^{-5}$	$1.6x10^{-5}$
703	0.001306	-0.00016	0.001501	0.000905	-0.00027	0.001167	0.001028	0.00104	0.00268	0.004086	0.003996
702	0.001159	-0.00019	0.001519	0.000812	-0.00012	0.001278	0.000917	0.000946	0.002619	0.004107	0.004106
701	0.00135	-0.0001	0.001403	0.000769	-0.00032	0.001137	0.001005	0.000852	0.002675	0.003995	0.004248
700	0.001241	-0.00022	0.001315	0.000849	-0.00033	0.001285	0.000977	0.00085	0.002596	0.003985	0.0042
699	0.001153	-0.00013	0.001378	0.000866	-0.00036	0.001109	0.000925	0.001048	0.002671	0.004189	0.004437
698	0.001163	-7.73E-05	0.001516	0.000844	-0.00036	0.001404	0.001091	0.000963	0.002865	0.00422	0.004616
697	0.001538	0.000153	0.001627	0.000963	-0.00017	0.001539	0.000982	0.001286	0.003017	0.00444	0.004723
696	0.001205	-7.05E-05	0.001583	0.000891	-0.00038	0.001229	0.000931	0.00109	0.002981	0.004252	0.004525
695	0.001453	8.89E-05	0.001683	0.000968	-0.00014	0.001592	0.001221	0.001358	0.003185	0.004618	0.004978
694	0.001344	-9.29E-05	0.001469	0.000872	-0.00011	0.001401	0.001026	0.001134	0.002973	0.004525	0.004854
693	0.001349	9.92E-05	0.001592	0.000951	-0.00045	0.001387	0.001065	0.001205	0.003144	0.004542	0.004849
692	0.001174	-9.16E-05	0.001438	0.001054	-0.00022	0.001411	0.001037	0.001187	0.003134	0.004657	0.005141
691	0.001345	2.62E-05	0.001512	0.000964	-0.00026	0.001579	0.001136	0.001255	0.003158	0.004626	0.005108
690	0.001525	0.000128	0.001753	0.00101	-0.0002	0.001402	0.001141	0.001341	0.003306	0.00485	0.005382
689	0.0014	0.000135	0.001685	0.000928	-9.29E-05	0.001626	0.00116	0.001422	0.00325	0.004938	0.005391
688	0.001297	4.02E-05	0.001466	0.000829	-0.00031	0.001494	0.000998	0.001157	0.002942	0.004491	0.004991
687	0.001368	0.000197	0.001761	0.001067	-0.00029	0.001593	0.001216	0.001331	0.003316	0.005071	0.005535
686	0.001389	3.61E-05	0.001771	0.000923	-0.00019	0.001662	0.001112	0.00128	0.003277	0.00506	0.00562
685	0.001421	8.61E-05	0.00152	0.000969	-0.00026	0.001641	0.001302	0.001412	0.003347	0.005216	0.005708
684	0.001584	4.74E-05	0.001615	0.000986	-0.00023	0.001509	0.001285	0.001462	0.003457	0.005201	0.005765
683	0.001521	0.00014	0.001749	0.000892	-0.0001	0.00178	0.001274	0.001464	0.003628	0.00544	0.006226
682	0.001329	-0.00021	0.001524	0.000859	-0.0003	0.001466	0.001148	0.001359	0.003395	0.005188	0.005943
681	0.001449	7.04E-05	0.001632	0.000986	-0.00015	0.001481	0.001262	0.00155	0.003671	0.005339	0.006201
680	0.001198	-0.0001	0.001491	0.000905	-0.00032	0.001526	0.001234	0.00148	0.003272	0.005367	0.006223
679	0.001351	5.11E-05	0.001607	0.00096	-0.0001	0.001577	0.001343	0.001371	0.003582	0.005423	0.006262
678	0.00152	0.000408	0.001928	0.001099	-3.11E-06	0.001672	0.001397	0.001672	0.003744	0.005734	0.00669
677	0.001454	0.000223	0.001719	0.001111	-8.11E-05	0.002004	0.001377	0.001669	0.004	0.005847	0.006706
676	0.001487	0.000101	0.00177	0.001142	-0.0001	0.001908	0.001419	0.001688	0.00373	0.005759	0.006846
675	0.001417	0.000202	0.001664	0.001059	-2.86E-05	0.00192	0.001395	0.001739	0.003991	0.006027	0.006958
674	0.001297	0.000124	0.00169	0.000896	-0.0002	0.001887	0.001343	0.001734	0.003925	0.006047	0.007073
673	0.001532	0.000208	0.001778	0.001082	-0.00013	0.001865	0.001481	0.001873	0.004187	0.006214	0.007311
672	0.00145	0.000231	0.001876	0.001066	-0.00024	0.00179	0.001607	0.001923	0.004007	0.006321	0.007409
671	0.001529	0.000234	0.00174	0.001014	-0.00011	0.002158	0.001513	0.001911	0.00417	0.006321	0.007526

EXCLI Journal 2020;19:48-70 – ISSN 1611-2156
Received: October 03, 2019, accepted: December 19, 2019, published: January 03, 2020

**Absorption intensity**											
**Wavelength (nm)**	**concentration of acetylshikonin (M)**										
	A	*B*	*C*	*D*	*E*	*F*	*G*	*I*	*J*	*K*	*L*
	0.00	8.0x10^{-7}	1.6x10^{-6}	2.0x10^{-6}	2.8x10^{-6}	4.0x10^{-6}	4.8x10^{-6}	6.0x10^{-6}	8.0x10^{-6}	1.2x10^{-5}	1.6x10^{-5}
670	0.00156	0.000156	0.001829	0.001088	3.20E-05	0.002217	0.001596	0.002078	0.004178	0.006292	0.007698
669	0.001242	0.000255	0.00185	0.000962	5.71E-05	0.002024	0.001502	0.002008	0.004123	0.006345	0.007658
668	0.001395	0.000224	0.0018	0.001116	-7.81E-05	0.002135	0.001661	0.002155	0.004447	0.006612	0.007985
667	0.001607	0.000382	0.002036	0.001162	0.000109	0.002261	0.001859	0.002186	0.004561	0.006744	0.007991
666	0.001495	7.05E-05	0.00187	0.001144	-8.82E-05	0.002144	0.001748	0.002169	0.004424	0.006809	0.008235
665	0.001575	0.000219	0.001905	0.001105	-9.51E-05	0.002233	0.001634	0.00206	0.004638	0.006707	0.008344
664	0.001457	0.000172	0.001796	0.001046	-9.29E-05	0.002178	0.001763	0.002169	0.004507	0.007002	0.008544
663	0.001628	0.000259	0.001787	0.001159	3.13E-05	0.002191	0.0017	0.002272	0.004531	0.007147	0.008621
662	0.001321	0.000233	0.001833	0.001104	5.32E-05	0.002277	0.001724	0.002182	0.004609	0.007227	0.008902
661	0.001458	0.000477	0.002011	0.001133	9.77E-05	0.002468	0.001896	0.002429	0.004852	0.007414	0.009123
660	0.001636	0.000463	0.002081	0.001281	0.000187	0.002432	0.001975	0.002432	0.004862	0.007575	0.009285
659	0.001616	0.000472	0.002128	0.001308	0.000263	0.002543	0.002095	0.002755	0.005127	0.007683	0.009631
658	0.001581	0.000328	0.001947	0.001189	0.000175	0.002421	0.001924	0.002556	0.005078	0.007651	0.009759
657	0.001475	0.000399	0.002025	0.001189	8.25E-05	0.002415	0.001967	0.002517	0.005152	0.007846	0.00967
656	0.001431	0.000486	0.001995	0.001049	0.000209	0.002462	0.001924	0.002591	0.00513	0.007842	0.010118
655	0.001582	0.000553	0.002114	0.001337	0.00035	0.002783	0.00217	0.00279	0.005409	0.008294	0.010366
654	0.001551	0.00047	0.002074	0.001288	0.000164	0.002602	0.002194	0.002669	0.005337	0.008343	0.010554
653	0.00148	0.000596	0.002132	0.001247	7.82E-05	0.002634	0.002089	0.002733	0.005445	0.008321	0.010585
652	0.001509	0.000519	0.002018	0.001334	0.000167	0.002683	0.002158	0.002812	0.005496	0.008293	0.010755
651	0.001582	0.00059	0.002088	0.001332	0.0003	0.002646	0.002287	0.002916	0.005524	0.008721	0.010932
650	0.001796	0.000765	0.002303	0.001457	0.00041	0.002904	0.002396	0.003099	0.005724	0.009006	0.011299
649	0.001669	0.000684	0.002099	0.001361	0.000285	0.002913	0.002303	0.00301	0.005752	0.008903	0.011508
648	0.001719	0.000593	0.002192	0.001528	0.000373	0.003064	0.002536	0.003203	0.005874	0.009192	0.011799
647	0.001815	0.000635	0.002308	0.001365	0.000482	0.003125	0.002487	0.003123	0.006019	0.00926	0.011991
646	0.001783	0.000774	0.002344	0.001629	0.000474	0.003049	0.002654	0.003409	0.006185	0.009455	0.012387
645	0.001816	0.00077	0.002308	0.001542	0.000541	0.003178	0.002585	0.003391	0.006178	0.009633	0.012568
644	0.001789	0.000907	0.002362	0.001525	0.000506	0.003305	0.002714	0.003495	0.006244	0.009883	0.012804
643	0.001723	0.000888	0.00236	0.001449	0.000356	0.003338	0.002635	0.003483	0.006464	0.010014	0.012953
642	0.001629	0.000792	0.002246	0.001465	0.000265	0.003275	0.002686	0.003517	0.006411	0.010029	0.013169
641	0.001718	0.000814	0.002274	0.001609	0.000349	0.003122	0.002749	0.003468	0.006486	0.010178	0.013253
640	0.001817	0.000966	0.002439	0.001522	0.00043	0.003362	0.002755	0.003788	0.00675	0.010423	0.013724
639	0.001765	0.000928	0.002434	0.001628	0.000431	0.003441	0.003061	0.003809	0.006788	0.010462	0.013885
638	0.001802	0.000961	0.002474	0.001673	0.000576	0.003518	0.002955	0.003764	0.006928	0.010744	0.014153

Absorption intensity											
**Wavelength (nm)**	**concentration of acetylshikonin (M)**										
	A	*B*	*C*	*D*	*E*	*F*	*G*	*I*	*J*	*K*	*L*
	0.00	$8.0x10^{-7}$	$1.6x10^{-6}$	$2.0x10^{-6}$	$2.8x10^{-6}$	$4.0x10^{-6}$	$4.8x10^{-6}$	$6.0x10^{-6}$	$8.0x10^{-6}$	$1.2x10^{-5}$	$1.6x10^{-5}$
637	0.00191	0.001003	0.00252	0.001562	0.000628	0.003594	0.003004	0.003883	0.007051	0.010989	0.01451
636	0.001831	0.000973	0.002509	0.001651	0.000532	0.003524	0.003011	0.003906	0.007039	0.010949	0.01469
635	0.001825	0.001101	0.002548	0.001681	0.000643	0.003625	0.003084	0.003966	0.007271	0.011316	0.014855
634	0.001826	0.001004	0.00268	0.001752	0.000657	0.003762	0.003181	0.004146	0.007255	0.011399	0.015172
633	0.001875	0.001051	0.002556	0.001678	0.000784	0.003683	0.003358	0.004228	0.007391	0.011592	0.015546
632	0.002	0.001142	0.00263	0.001779	0.000665	0.003869	0.003197	0.004248	0.007639	0.011816	0.015702
631	0.001816	0.001135	0.002634	0.001756	0.000647	0.003911	0.003342	0.00432	0.007693	0.011961	0.01604
630	0.001903	0.001208	0.002603	0.001683	0.000743	0.004104	0.003452	0.004477	0.007759	0.011984	0.016334
629	0.001988	0.001253	0.002705	0.00197	0.000822	0.004054	0.003461	0.004536	0.008012	0.012311	0.016574
628	0.001952	0.001225	0.002628	0.001757	0.000821	0.004053	0.003382	0.004501	0.007999	0.012414	0.016839
627	0.001936	0.00122	0.002704	0.001803	0.000793	0.004086	0.003482	0.004659	0.008185	0.012705	0.017118
626	0.001881	0.00117	0.002708	0.001829	0.00079	0.004253	0.003534	0.004803	0.00821	0.012741	0.017347
625	0.002065	0.001278	0.002817	0.001844	0.000979	0.004326	0.003555	0.00493	0.008488	0.013092	0.017735
624	0.001923	0.001243	0.002674	0.00187	0.000791	0.00434	0.003593	0.004806	0.008339	0.013307	0.017976
623	0.002087	0.001443	0.00286	0.002068	0.000957	0.00437	0.003822	0.005132	0.008585	0.013497	0.018251
622	0.002096	0.001337	0.002812	0.001944	0.000926	0.004319	0.00371	0.005106	0.008771	0.013622	0.018548
621	0.001846	0.001181	0.002693	0.001861	0.00094	0.004441	0.003753	0.005128	0.008678	0.013661	0.018687
620	0.002057	0.001411	0.003008	0.001966	0.001024	0.004645	0.003961	0.005239	0.008937	0.014121	0.019132
619	0.002096	0.001425	0.002923	0.002095	0.001111	0.004593	0.004053	0.005269	0.009114	0.014218	0.01938
618	0.002093	0.001461	0.003026	0.001978	0.000869	0.00473	0.004057	0.005378	0.009139	0.014206	0.019687
617	0.002098	0.001405	0.002826	0.002132	0.001087	0.004855	0.004133	0.005669	0.009293	0.014519	0.019902
616	0.002062	0.001424	0.002946	0.001989	0.000961	0.004864	0.004086	0.005543	0.009352	0.014634	0.020125
615	0.002048	0.001402	0.00306	0.002131	0.00105	0.004954	0.004297	0.005516	0.009704	0.014865	0.020621
614	0.002016	0.001378	0.003063	0.002176	0.001049	0.00502	0.00435	0.005762	0.009795	0.015218	0.020784
613	0.002115	0.001481	0.003051	0.002131	0.001213	0.00505	0.004372	0.005813	0.009882	0.015256	0.021137
612	0.002114	0.001536	0.003088	0.002048	0.001167	0.005095	0.004455	0.005947	0.01	0.01548	0.02139
611	0.002052	0.001549	0.003053	0.002132	0.001185	0.005177	0.004473	0.006156	0.010081	0.015829	0.021735
610	0.002153	0.001591	0.0032	0.002222	0.001294	0.005188	0.00455	0.0061	0.010183	0.016006	0.021899
609	0.002075	0.001648	0.003194	0.002296	0.001325	0.005286	0.004636	0.006163	0.010464	0.016108	0.022321
608	0.002083	0.001586	0.00311	0.002271	0.001189	0.005402	0.004704	0.006265	0.010402	0.016251	0.022501
607	0.002189	0.001512	0.003215	0.00222	0.001193	0.005319	0.004688	0.006147	0.010495	0.016416	0.022891
606	0.002176	0.001704	0.003234	0.002296	0.001402	0.005491	0.004878	0.006414	0.01075	0.016767	0.023061
605	0.002124	0.001699	0.003261	0.002485	0.00138	0.00563	0.004958	0.00664	0.010866	0.016832	0.023497

EXCLI Journal 2020;19:48-70 – ISSN 1611-2156
Received: October 03, 2019, accepted: December 19, 2019, published: January 03, 2020

Wavelength (nm)	***Absorption intensity*** **concentration of acetylshikonin (M)**										
	A	*B*	*C*	*D*	*E*	*F*	*G*	*I*	*J*	*K*	*L*
	0.00	$8.0x10^{-7}$	$1.6x10^{-6}$	$2.0x10^{-6}$	$2.8x10^{-6}$	$4.0x10^{-6}$	$4.8x10^{-6}$	$6.0x10^{-6}$	$8.0x10^{-6}$	$1.2x10^{-5}$	$1.6x10^{-5}$
604	0.002013	0.00155	0.003228	0.002167	0.001291	0.005511	0.004698	0.006593	0.01078	0.016882	0.023647
603	0.002283	0.001751	0.003346	0.002389	0.001583	0.005883	0.0052	0.006607	0.011191	0.017236	0.024009
602	0.002139	0.001645	0.003315	0.002544	0.001433	0.00587	0.005141	0.006873	0.011314	0.01739	0.024269
601	0.002157	0.001601	0.003375	0.002539	0.001387	0.005834	0.005065	0.006959	0.011225	0.017476	0.024457
600	0.002266	0.001669	0.003396	0.002384	0.001467	0.005913	0.005121	0.006869	0.011398	0.01764	0.024838
599	0.002139	0.00165	0.003345	0.002435	0.001421	0.006	0.005224	0.00704	0.011537	0.01802	0.024982
598	0.002253	0.001839	0.003512	0.002609	0.00168	0.006114	0.005411	0.007179	0.011815	0.018218	0.02545
597	0.002086	0.001598	0.003554	0.002477	0.001505	0.006081	0.005196	0.007154	0.011807	0.018346	0.025574
596	0.002295	0.001736	0.003467	0.002608	0.001573	0.006236	0.005541	0.007383	0.011936	0.018523	0.026025
595	0.002347	0.001698	0.003616	0.002646	0.001727	0.006289	0.005543	0.007357	0.012093	0.018784	0.026307
594	0.002342	0.00174	0.003615	0.002815	0.001851	0.006418	0.005671	0.007521	0.012374	0.019074	0.026741
593	0.002289	0.001745	0.003688	0.002739	0.001734	0.006407	0.005494	0.007699	0.012422	0.01918	0.026887
592	0.002274	0.001819	0.003647	0.002695	0.001798	0.006361	0.005676	0.007763	0.012547	0.019456	0.027244
591	0.00225	0.001596	0.003617	0.00284	0.001793	0.006524	0.005764	0.007674	0.012759	0.019509	0.027469
590	0.002146	0.001613	0.003607	0.002568	0.001704	0.006479	0.005677	0.007692	0.012662	0.019653	0.027583
589	0.002383	0.001798	0.003817	0.00286	0.001901	0.006652	0.005884	0.007837	0.012845	0.01999	0.028081
588	0.002217	0.001739	0.003675	0.002885	0.001766	0.006579	0.005839	0.007966	0.013197	0.020177	0.028231
587	0.002297	0.001559	0.003841	0.002705	0.001886	0.006892	0.005922	0.008181	0.013184	0.020202	0.028665
586	0.002349	0.001759	0.0037	0.002895	0.00183	0.006795	0.006092	0.00814	0.013246	0.020492	0.028982
585	0.002321	0.001637	0.003751	0.002823	0.002062	0.006906	0.006046	0.008145	0.013374	0.020783	0.029165
584	0.002374	0.001805	0.00393	0.002864	0.002028	0.007055	0.00629	0.008448	0.013736	0.021087	0.029565
583	0.002303	0.001601	0.003702	0.002797	0.001977	0.006869	0.006201	0.008329	0.013549	0.021233	0.029766
582	0.002283	0.001625	0.003952	0.002914	0.002046	0.007106	0.006272	0.008382	0.013821	0.02126	0.030043
581	0.00228	0.001682	0.003861	0.002836	0.002035	0.007199	0.006452	0.008573	0.014041	0.021525	0.03038
580	0.002573	0.001813	0.004006	0.003105	0.002272	0.007297	0.006427	0.008718	0.014301	0.021939	0.030789
579	0.002363	0.001738	0.004064	0.003068	0.002193	0.007273	0.006565	0.008815	0.014337	0.02209	0.030954
578	0.002418	0.001829	0.004127	0.003042	0.002271	0.00745	0.006782	0.008936	0.014417	0.022226	0.031431
577	0.002441	0.00171	0.004034	0.003039	0.002174	0.007496	0.006879	0.008946	0.01445	0.022442	0.031481
576	0.002365	0.001676	0.0039	0.003037	0.002114	0.007378	0.006677	0.008962	0.014508	0.022597	0.031467
575	0.002354	0.001608	0.004109	0.002985	0.002241	0.007519	0.006779	0.00916	0.014809	0.022767	0.031852
574	0.002247	0.001543	0.004043	0.002993	0.002215	0.007577	0.006815	0.009023	0.014803	0.022622	0.032007
573	0.002395	0.001622	0.004084	0.003085	0.002421	0.007665	0.006969	0.009148	0.015071	0.022865	0.032273
572	0.00254	0.001748	0.00425	0.00325	0.002372	0.007809	0.006999	0.009452	0.01519	0.023004	0.032599

EXCLI Journal 2020;19:48-70 – ISSN 1611-2156
Received: October 03, 2019, accepted: December 19, 2019, published: January 03, 2020

Absorption intensity											
**Wavelength (nm)**	**concentration of acetylshikonin (M)**										
	A	*B*	*C*	*D*	*E*	*F*	*G*	*I*	*J*	*K*	*L*
	0.00	8.0x10^{-7}	1.6x10^{-6}	2.0x10^{-6}	2.8x10^{-6}	4.0x10^{-6}	4.8x10^{-6}	6.0x10^{-6}	8.0x10^{-6}	1.2x10^{-5}	1.6x10^{-5}
571	0.002288	0.001708	0.004095	0.003121	0.002201	0.007886	0.007034	0.009478	0.015212	0.023067	0.032717
570	0.002318	0.001698	0.004244	0.003194	0.002431	0.007954	0.007179	0.009493	0.015184	0.023428	0.033055
569	0.002398	0.001786	0.004522	0.003237	0.002624	0.008114	0.007172	0.009535	0.015336	0.023454	0.033525
568	0.002387	0.001565	0.004429	0.003337	0.002621	0.008085	0.007429	0.009762	0.015711	0.02406	0.034063
567	0.002572	0.001448	0.004216	0.003301	0.002458	0.008066	0.007403	0.009748	0.015748	0.024193	0.034247
566	0.002438	0.001459	0.004303	0.003392	0.002639	0.00831	0.007289	0.009967	0.016092	0.024409	0.034452
565	0.002419	0.001506	0.004425	0.003422	0.002567	0.008354	0.007466	0.009992	0.016036	0.024648	0.03469
564	0.002561	0.001534	0.004298	0.003412	0.00253	0.008291	0.007414	0.010028	0.016096	0.024767	0.034937
563	0.002392	0.001431	0.004411	0.003275	0.0026	0.008344	0.007469	0.009872	0.016181	0.024796	0.035018
562	0.002367	0.001361	0.004371	0.003208	0.002459	0.008377	0.007505	0.010057	0.016251	0.024945	0.03518
561	0.002639	0.001578	0.00474	0.003783	0.002849	0.008522	0.007753	0.010382	0.016493	0.025344	0.035606
560	0.002436	0.001493	0.004453	0.003471	0.002612	0.008769	0.007756	0.010342	0.016603	0.025413	0.0358
559	0.002586	0.001574	0.004457	0.003514	0.00284	0.008769	0.007849	0.010527	0.016756	0.025484	0.036149
558	0.002502	0.001384	0.004496	0.003431	0.002642	0.008673	0.007843	0.010489	0.01672	0.025662	0.036128
557	0.002674	0.001455	0.004671	0.003531	0.002773	0.008841	0.008041	0.0105	0.016895	0.025787	0.036513
556	0.00245	0.001441	0.004598	0.003517	0.00289	0.008802	0.007996	0.010692	0.016945	0.026104	0.036653
555	0.002673	0.001495	0.004759	0.003612	0.002967	0.009013	0.008064	0.010808	0.017247	0.026281	0.036884
554	0.002724	0.001594	0.004868	0.00376	0.002986	0.009067	0.008215	0.010856	0.017377	0.026387	0.037169
553	0.002788	0.001575	0.004881	0.003707	0.003028	0.009291	0.008374	0.011009	0.01743	0.026674	0.037448
552	0.002679	0.001583	0.004696	0.003819	0.00309	0.009138	0.008329	0.010991	0.017646	0.026833	0.037597
551	0.002521	0.001541	0.004719	0.003739	0.002996	0.009107	0.00835	0.011028	0.017626	0.026876	0.037731
550	0.002577	0.001537	0.004855	0.00369	0.003053	0.009363	0.008428	0.011191	0.017789	0.027167	0.038118
549	0.002718	0.001594	0.004865	0.003872	0.003089	0.009354	0.008565	0.011319	0.017928	0.027255	0.03836
548	0.002804	0.001574	0.004977	0.003919	0.003264	0.009357	0.008618	0.011345	0.018145	0.027463	0.0385
547	0.002706	0.001609	0.005057	0.003881	0.003154	0.009656	0.008674	0.011436	0.018283	0.0278	0.038898
546	0.002859	0.001478	0.004931	0.00404	0.003257	0.009511	0.00876	0.011533	0.018372	0.027755	0.039106
545	0.002744	0.00152	0.004907	0.003899	0.003182	0.009745	0.008798	0.011628	0.018488	0.027998	0.039325
544	0.0028	0.001631	0.005015	0.004052	0.003477	0.009739	0.00893	0.011801	0.018724	0.02816	0.039681
543	0.00275	0.001524	0.005128	0.00401	0.003435	0.009748	0.008966	0.011783	0.018922	0.028195	0.040046
542	0.002545	0.001462	0.005015	0.003994	0.003442	0.009815	0.009012	0.011823	0.018833	0.028512	0.040156
541	0.002524	0.001558	0.005059	0.004069	0.003338	0.009947	0.009173	0.011887	0.018921	0.028667	0.040357
540	0.002728	0.001364	0.005085	0.004091	0.003492	0.010036	0.009174	0.011995	0.019052	0.028861	0.04063
539	0.002879	0.001721	0.005131	0.0044	0.003698	0.010248	0.009471	0.012271	0.019404	0.029327	0.040961

EXCLI Journal 2020;19:48-70 – ISSN 1611-2156
Received: October 03, 2019, accepted: December 19, 2019, published: January 03, 2020

Absorption intensity											
**Wavelength (nm)**	**concentration of acetylshikonin (M)**										
	A	*B*	*C*	*D*	*E*	*F*	*G*	*I*	*J*	*K*	*L*
	0.00	8.0x10^{-7}	1.6x10^{-6}	2.0x10^{-6}	2.8x10^{-6}	4.0x10^{-6}	4.8x10^{-6}	6.0x10^{-6}	8.0x10^{-6}	1.2x10^{-5}	1.6x10^{-5}
538	0.002777	0.001533	0.005333	0.004132	0.003508	0.01027	0.009336	0.012248	0.019563	0.029383	0.041229
537	0.002864	0.001461	0.005294	0.004232	0.003694	0.010281	0.009427	0.012417	0.019651	0.029419	0.041569
536	0.002821	0.001667	0.005398	0.004378	0.003829	0.010485	0.009605	0.012399	0.019953	0.029878	0.041821
535	0.002786	0.001486	0.005319	0.004317	0.003738	0.010417	0.009665	0.012541	0.019825	0.030072	0.042013
534	0.002968	0.001569	0.005588	0.004565	0.003981	0.010559	0.009813	0.012737	0.020167	0.030237	0.042436
533	0.00282	0.001752	0.005513	0.004469	0.003913	0.0107	0.009928	0.01285	0.020361	0.030515	0.042614
532	0.002977	0.001737	0.00556	0.004346	0.004094	0.010675	0.009992	0.013037	0.020555	0.030714	0.042907
531	0.002877	0.001591	0.005583	0.00439	0.003817	0.010715	0.009901	0.012983	0.02054	0.030664	0.043209
530	0.002796	0.00179	0.005563	0.00457	0.004094	0.01096	0.010107	0.013215	0.020819	0.031077	0.04345
529	0.002816	0.001666	0.005541	0.00458	0.003961	0.010804	0.010109	0.013075	0.020951	0.031088	0.043541
528	0.003022	0.001704	0.005662	0.004491	0.004125	0.010949	0.010247	0.013279	0.020995	0.03132	0.043908
527	0.00289	0.001858	0.005721	0.004784	0.004245	0.011198	0.010425	0.013443	0.021194	0.03159	0.044158
526	0.002823	0.001806	0.005805	0.004733	0.0041	0.011203	0.010338	0.013341	0.021321	0.031684	0.044275
525	0.002931	0.001624	0.005856	0.004699	0.004224	0.011194	0.010527	0.013548	0.02147	0.031857	0.044639
524	0.002955	0.001623	0.005805	0.004709	0.004267	0.011239	0.010671	0.013511	0.021482	0.032042	0.044712
523	0.002988	0.001776	0.005911	0.00487	0.004361	0.011475	0.010645	0.013806	0.021783	0.032401	0.045115
522	0.003149	0.001899	0.006082	0.004938	0.004481	0.011642	0.010906	0.013855	0.021957	0.032428	0.04541
521	0.003038	0.001782	0.005939	0.004822	0.004416	0.011612	0.010701	0.014012	0.022032	0.032624	0.045471
520	0.002906	0.001795	0.005956	0.004878	0.004394	0.011632	0.010704	0.013867	0.02204	0.032751	0.045584
519	0.003081	0.001891	0.006078	0.004913	0.004465	0.011916	0.011029	0.014029	0.022247	0.032927	0.045874
518	0.003087	0.00165	0.00588	0.004878	0.004507	0.011712	0.010999	0.01403	0.022346	0.033168	0.046017
517	0.003126	0.001961	0.006099	0.005068	0.004528	0.011912	0.011213	0.014291	0.022622	0.033303	0.046334
516	0.003089	0.001933	0.006157	0.005161	0.004774	0.012136	0.011105	0.01431	0.022683	0.033496	0.046425
515	0.003006	0.001784	0.006138	0.004947	0.00475	0.012042	0.011312	0.014316	0.022618	0.033496	0.046641
514	0.003125	0.001792	0.006171	0.005202	0.004749	0.012042	0.011351	0.014451	0.022919	0.03382	0.046868
513	0.003063	0.001952	0.006223	0.005187	0.00482	0.012223	0.011541	0.01453	0.023025	0.034114	0.047133
512	0.003111	0.002008	0.006391	0.005463	0.004956	0.012363	0.011758	0.014939	0.023404	0.034199	0.047482
511	0.003165	0.001886	0.006266	0.005267	0.005061	0.012394	0.011671	0.014767	0.023344	0.034159	0.047358
510	0.003238	0.002069	0.006486	0.005157	0.004984	0.012541	0.011681	0.014815	0.023401	0.034454	0.047503
509	0.003084	0.001922	0.006196	0.005368	0.004982	0.012429	0.011725	0.014887	0.023451	0.034551	0.047796
508	0.003098	0.002026	0.006424	0.005305	0.005063	0.01252	0.011716	0.014959	0.023543	0.03472	0.048006
507	0.003035	0.001931	0.006502	0.005396	0.00487	0.012617	0.011813	0.014987	0.023742	0.034858	0.048118
506	0.003076	0.001914	0.006576	0.005324	0.005076	0.012653	0.011911	0.014953	0.02379	0.03494	0.048305

EXCLI Journal 2020;19:48-70 – ISSN 1611-2156
Received: October 03, 2019, accepted: December 19, 2019, published: January 03, 2020

Absorption intensity											
**Wavelength (nm)**	**concentration of acetylshikonin (M)**										
	A	*B*	*C*	*D*	*E*	*F*	*G*	*I*	*J*	*K*	*L*
	0.00	$8.0x10^{-7}$	$1.6x10^{-6}$	$2.0x10^{-6}$	$2.8x10^{-6}$	$4.0x10^{-6}$	$4.8x10^{-6}$	$6.0x10^{-6}$	$8.0x10^{-6}$	$1.2x10^{-5}$	$1.6x10^{-5}$
505	0.003327	0.002041	0.006654	0.005625	0.005261	0.012867	0.012132	0.015267	0.024166	0.035237	0.048696
504	0.003035	0.001867	0.006355	0.005383	0.004959	0.012647	0.01183	0.015029	0.023782	0.035134	0.048452
503	0.003088	0.001848	0.006588	0.005275	0.005056	0.012835	0.012026	0.015324	0.024119	0.035379	0.048845
502	0.003348	0.002092	0.006644	0.005508	0.005195	0.0128	0.012264	0.015477	0.024327	0.035596	0.048846
501	0.00322	0.002008	0.006525	0.005342	0.00516	0.012914	0.012102	0.015282	0.024263	0.035476	0.049044
500	0.003386	0.002097	0.006851	0.005407	0.00534	0.013046	0.012235	0.015454	0.024476	0.03583	0.049419
499	0.003287	0.002126	0.006718	0.005476	0.005312	0.012959	0.012193	0.015487	0.024506	0.035925	0.049402
498	0.00303	0.001843	0.006645	0.005252	0.005263	0.013029	0.01221	0.015423	0.024618	0.035744	0.049578
497	0.003246	0.002009	0.006726	0.005544	0.005447	0.013287	0.012389	0.015736	0.024741	0.036144	0.049701
496	0.003247	0.00203	0.006884	0.005579	0.005386	0.013115	0.012362	0.015768	0.024951	0.036344	0.049877
495	0.003311	0.002058	0.006827	0.005651	0.005388	0.013268	0.01251	0.015788	0.025155	0.036424	0.05003
494	0.003146	0.002109	0.006967	0.005743	0.005455	0.013363	0.012559	0.015777	0.025157	0.036512	0.050142
493	0.003242	0.001996	0.00681	0.005605	0.005483	0.013345	0.012623	0.015818	0.025063	0.036618	0.05022
492	0.0033	0.002054	0.006888	0.005786	0.005622	0.013543	0.012618	0.015786	0.025304	0.03673	0.050342
491	0.003285	0.002053	0.006899	0.005663	0.005455	0.01338	0.012554	0.015872	0.025196	0.036726	0.050391
490	0.003214	0.002137	0.007029	0.005623	0.005551	0.013461	0.012791	0.016006	0.025378	0.036734	0.050456
489	0.003403	0.002003	0.00703	0.005703	0.005599	0.013504	0.012681	0.015974	0.02554	0.036708	0.05039
488	0.003361	0.002052	0.007055	0.005611	0.005488	0.013445	0.012689	0.01605	0.025501	0.036835	0.050519
487	0.003543	0.00234	0.007289	0.005942	0.005765	0.013752	0.01287	0.016232	0.025646	0.037146	0.050666
486	0.003328	0.001944	0.00709	0.005725	0.005624	0.013617	0.012691	0.016036	0.025565	0.03693	0.050597
485	0.003202	0.002024	0.007153	0.005766	0.005535	0.01369	0.012813	0.015954	0.025441	0.03691	0.050678
484	0.003371	0.001991	0.00695	0.005778	0.005651	0.0137	0.012893	0.016063	0.02561	0.037024	0.050619
483	0.003506	0.002094	0.007172	0.00586	0.005844	0.013797	0.012832	0.016117	0.025785	0.037109	0.050752
482	0.003332	0.002017	0.007185	0.005895	0.005718	0.013679	0.012885	0.01616	0.025792	0.037176	0.050643
481	0.003471	0.002147	0.007182	0.005862	0.005774	0.013954	0.013103	0.016239	0.025798	0.037311	0.050799
480	0.003378	0.001896	0.007231	0.005753	0.005752	0.013728	0.012852	0.016138	0.025786	0.0371	0.050516
479	0.003545	0.002155	0.007481	0.006037	0.005912	0.013984	0.01304	0.01632	0.025972	0.037239	0.050783
478	0.003481	0.0021	0.007491	0.006009	0.005972	0.014033	0.013053	0.016285	0.026022	0.037211	0.050927
477	0.003451	0.002131	0.007419	0.006038	0.005782	0.013777	0.012977	0.016247	0.025873	0.03704	0.050464
476	0.003521	0.002049	0.007597	0.006039	0.005977	0.013812	0.012896	0.016162	0.025993	0.037172	0.050635
475	0.003673	0.002196	0.007486	0.005881	0.006025	0.014028	0.013053	0.0163	0.026024	0.037095	0.050602
474	0.003553	0.002096	0.007346	0.005955	0.005869	0.013844	0.013007	0.015897	0.026009	0.03711	0.05041
473	0.003576	0.002152	0.007599	0.005828	0.005797	0.01392	0.013115	0.01617	0.02597	0.03695	0.050296

Received: October 03, 2019, accepted: December 19, 2019, published: January 03, 2020

Absorption intensity											
**Wavelength (nm)**	**concentration of acetylshikonin (M)**										
	A	*B*	*C*	*D*	*E*	*F*	*G*	*I*	*J*	*K*	*L*
	0.00	$8.0x10^{-7}$	$1.6x10^{-6}$	$2.0x10^{-6}$	$2.8x10^{-6}$	$4.0x10^{-6}$	$4.8x10^{-6}$	$6.0x10^{-6}$	$8.0x10^{-6}$	$1.2x10^{-5}$	$1.6x10^{-5}$
472	0.003532	0.002072	0.007502	0.005995	0.00589	0.013971	0.013115	0.016267	0.025988	0.036922	0.050317
471	0.003659	0.002014	0.007559	0.006043	0.005966	0.013988	0.013161	0.01615	0.026042	0.03707	0.050314
470	0.003734	0.002254	0.007554	0.005919	0.00592	0.014118	0.013025	0.016238	0.025839	0.036896	0.050166
469	0.003533	0.001883	0.007432	0.005797	0.005749	0.013978	0.01296	0.015983	0.025971	0.036718	0.05004
468	0.003885	0.002208	0.007727	0.006178	0.005956	0.014142	0.013185	0.016215	0.026057	0.03689	0.049964
467	0.003859	0.002154	0.007665	0.005932	0.005884	0.014	0.013095	0.016068	0.026076	0.036779	0.04984
466	0.003775	0.002173	0.007731	0.005856	0.005829	0.014081	0.012867	0.016182	0.02594	0.03678	0.049416
465	0.003771	0.002088	0.007632	0.006225	0.005917	0.014037	0.013103	0.016243	0.025876	0.036603	0.049315
464	0.003825	0.002004	0.007657	0.005864	0.005747	0.013966	0.012839	0.015846	0.025809	0.036125	0.049125
463	0.003652	0.002003	0.007699	0.005925	0.005791	0.013933	0.012914	0.015978	0.025658	0.036064	0.049022
462	0.003864	0.002146	0.007741	0.00607	0.005794	0.01391	0.012871	0.015871	0.02538	0.036017	0.048886
461	0.003879	0.002181	0.007653	0.006014	0.005841	0.013877	0.012861	0.01569	0.025487	0.036001	0.048837
460	0.004037	0.002161	0.007858	0.006024	0.005818	0.01352	0.012574	0.015567	0.025432	0.035848	0.048801
459	0.004109	0.002147	0.007545	0.005873	0.005734	0.013584	0.012445	0.015489	0.025499	0.035899	0.048594
458	0.00364	0.002093	0.007617	0.005646	0.00563	0.013526	0.012331	0.015397	0.025421	0.035696	0.048495
457	0.003916	0.002032	0.007881	0.005886	0.005626	0.013948	0.012665	0.015612	0.0256	0.035889	0.048629
456	0.004059	0.002143	0.007961	0.006142	0.005887	0.013899	0.012827	0.015654	0.025591	0.035847	0.048428
455	0.003948	0.002165	0.007966	0.005942	0.00583	0.013713	0.012641	0.015614	0.025563	0.035655	0.048265
454	0.004026	0.002059	0.007787	0.005807	0.005704	0.013843	0.012675	0.015554	0.025385	0.035505	0.048179
453	0.004051	0.002196	0.007912	0.005916	0.005739	0.013814	0.012639	0.015441	0.025438	0.035525	0.04776
452	0.004007	0.002052	0.007901	0.005773	0.005562	0.01364	0.012388	0.015292	0.02529	0.035155	0.047596
451	0.003863	0.001902	0.007904	0.005706	0.005602	0.013689	0.012362	0.015288	0.0253	0.035116	0.047397
450	0.004283	0.002212	0.008133	0.005929	0.005679	0.013885	0.01268	0.015614	0.025234	0.035122	0.047322
449	0.004213	0.002011	0.007899	0.005721	0.005677	0.013738	0.012387	0.015366	0.025243	0.034952	0.047203
448	0.004103	0.002169	0.007976	0.005625	0.005505	0.013624	0.012368	0.015013	0.025114	0.034762	0.046825
447	0.004396	0.002192	0.007993	0.005936	0.005494	0.013789	0.012312	0.015236	0.025094	0.034824	0.046973
446	0.004323	0.001953	0.008081	0.005947	0.005528	0.013604	0.012311	0.015073	0.025079	0.034605	0.046433
445	0.004296	0.002211	0.008062	0.005814	0.00579	0.013749	0.012182	0.015087	0.024926	0.034413	0.0464
444	0.004446	0.002221	0.00812	0.005955	0.005531	0.013663	0.012361	0.015227	0.025121	0.034246	0.046289
443	0.004376	0.002181	0.008201	0.005884	0.0056	0.013568	0.012102	0.014944	0.024809	0.03413	0.045997
442	0.004134	0.001889	0.00815	0.005561	0.005444	0.01348	0.011926	0.014888	0.024704	0.033973	0.045832
441	0.004439	0.001964	0.00819	0.005705	0.005442	0.013501	0.012092	0.014878	0.024622	0.033845	0.045495
440	0.004516	0.002062	0.008271	0.006008	0.005565	0.013628	0.012048	0.014859	0.02476	0.033881	0.0455

EXCLI Journal 2020;19:48-70 – ISSN 1611-2156
Received: October 03, 2019, accepted: December 19, 2019, published: January 03, 2020

Absorption intensity											
**Wavelength (nm)**	**concentration of acetylshikonin (M)**										
	A	*B*	*C*	*D*	*E*	*F*	*G*	*I*	*J*	*K*	*L*
	0.00	$8.0x10^{-7}$	$1.6x10^{-6}$	$2.0x10^{-6}$	$2.8x10^{-6}$	$4.0x10^{-6}$	$4.8x10^{-6}$	$6.0x10^{-6}$	$8.0x10^{-6}$	$1.2x10^{-5}$	$1.6x10^{-5}$
439	0.00425	0.00197	0.008065	0.005744	0.005397	0.013321	0.011755	0.014722	0.024381	0.0334	0.045046
438	0.004334	0.001953	0.00814	0.005612	0.005395	0.013388	0.01183	0.014521	0.024261	0.033241	0.044995
437	0.004465	0.002136	0.007995	0.005631	0.005294	0.013334	0.011903	0.014501	0.024283	0.033167	0.044678
436	0.004562	0.001961	0.008223	0.005643	0.005508	0.01352	0.011881	0.01459	0.024482	0.033187	0.044693
435	0.004364	0.00171	0.00811	0.005527	0.005268	0.013176	0.01168	0.014364	0.024027	0.032792	0.044423
434	0.004345	0.001878	0.008217	0.005616	0.005191	0.013193	0.011606	0.014461	0.024191	0.03288	0.044209
433	0.004531	0.001985	0.00829	0.005561	0.005392	0.013421	0.0116	0.01419	0.024178	0.032564	0.044008
432	0.004555	0.001923	0.007988	0.005503	0.005257	0.013205	0.011519	0.014221	0.023965	0.032595	0.043776
431	0.00453	0.00181	0.008253	0.005516	0.005222	0.01322	0.01154	0.014093	0.023948	0.032424	0.043441
430	0.004719	0.002024	0.008214	0.005679	0.005129	0.013207	0.011441	0.014106	0.023955	0.032364	0.043536
429	0.004577	0.00186	0.008158	0.005356	0.005116	0.013302	0.011427	0.01409	0.023847	0.032072	0.04335
428	0.004787	0.00204	0.008307	0.005768	0.005294	0.013345	0.01139	0.014005	0.023816	0.032078	0.043044
427	0.004747	0.001967	0.008198	0.005354	0.005299	0.012988	0.011022	0.01377	0.023591	0.031797	0.042723
426	0.004607	0.001782	0.008218	0.005374	0.005023	0.013023	0.01118	0.01366	0.023637	0.03173	0.04251
425	0.004775	0.00199	0.008189	0.00561	0.005139	0.013314	0.011418	0.014003	0.023589	0.031934	0.042582
424	0.004639	0.001919	0.008238	0.005401	0.005127	0.01281	0.01107	0.013579	0.023529	0.031393	0.042163
423	0.005014	0.002119	0.008518	0.005635	0.005057	0.013139	0.01126	0.013666	0.023636	0.031415	0.042188
422	0.004803	0.001937	0.008365	0.005399	0.005209	0.013097	0.011067	0.013805	0.023474	0.031384	0.042028
421	0.00498	0.002121	0.008463	0.005688	0.005021	0.013058	0.011053	0.013716	0.023347	0.031224	0.041883
420	0.004879	0.001843	0.008529	0.005586	0.005105	0.013195	0.011113	0.013417	0.023431	0.031211	0.041959
419	0.005021	0.002021	0.008388	0.00535	0.005019	0.013255	0.011095	0.013763	0.02302	0.030691	0.041423
418	0.005129	0.001875	0.008455	0.005531	0.00514	0.012786	0.011084	0.013034	0.02302	0.030696	0.041213
417	0.004684	0.001882	0.00818	0.005092	0.004566	0.012561	0.01054	0.013077	0.022979	0.030092	0.040815
416	0.005057	0.002061	0.008485	0.005277	0.005065	0.012886	0.010835	0.013395	0.023187	0.030614	0.041174
415	0.004969	0.001854	0.00863	0.005337	0.004936	0.012773	0.010668	0.013306	0.023091	0.030561	0.041004
414	0.005091	0.001847	0.008405	0.00547	0.005103	0.013019	0.010835	0.013417	0.023187	0.030521	0.041102
413	0.005032	0.001825	0.008557	0.005263	0.004848	0.012848	0.010936	0.013264	0.02297	0.030448	0.040821
412	0.005276	0.001876	0.008752	0.005729	0.005176	0.012919	0.010803	0.013327	0.02335	0.030617	0.040908
411	0.00492	0.001652	0.008553	0.005287	0.00487	0.01299	0.010629	0.013077	0.023134	0.030439	0.040655
410	0.005065	0.00178	0.008333	0.005099	0.00472	0.012838	0.010428	0.013077	0.022845	0.029975	0.04041
409	0.005221	0.001851	0.008636	0.00533	0.004807	0.01296	0.010347	0.01298	0.022821	0.030028	0.040495
408	0.005271	0.00186	0.008712	0.005321	0.004798	0.013042	0.010562	0.013028	0.023209	0.030294	0.040622
407	0.005224	0.001795	0.008646	0.005218	0.004868	0.013092	0.010789	0.013166	0.023252	0.030089	0.040541

EXCLI Journal 2020;19:48-70 – ISSN 1611-2156
Received: October 03, 2019, accepted: December 19, 2019, published: January 03, 2020

Absorption intensity											
**Wavelength (nm)**	**concentration of acetylshikonin (M)**										
	A	*B*	*C*	*D*	*E*	*F*	*G*	*I*	*J*	*K*	*L*
	0.00	$8.0x10^{-7}$	$1.6x10^{-6}$	$2.0x10^{-6}$	$2.8x10^{-6}$	$4.0x10^{-6}$	$4.8x10^{-6}$	$6.0x10^{-6}$	$8.0x10^{-6}$	$1.2x10^{-5}$	$1.6x10^{-5}$
406	0.005463	0.001808	0.008786	0.005467	0.005146	0.013158	0.010632	0.013077	0.023108	0.030247	0.040491
405	0.005409	0.001969	0.008838	0.005379	0.004989	0.013151	0.010549	0.013146	0.023219	0.030185	0.040486
404	0.005164	0.001541	0.008683	0.005184	0.004746	0.013089	0.010582	0.012883	0.023143	0.029856	0.039987
403	0.0056	0.00168	0.008726	0.00532	0.004818	0.013147	0.010502	0.012961	0.022753	0.029716	0.039987
402	0.005402	0.001833	0.008727	0.005254	0.004965	0.012815	0.010378	0.012786	0.023002	0.030058	0.04027
401	0.005513	0.001632	0.008837	0.005062	0.00468	0.013229	0.010421	0.013092	0.023214	0.030112	0.040492
400	0.005653	0.002117	0.009127	0.005409	0.004971	0.013327	0.010794	0.013186	0.023338	0.030388	0.040828
399	0.005867	0.001759	0.009083	0.005454	0.005074	0.013231	0.010602	0.01327	0.023376	0.030368	0.040793
398	0.00555	0.001682	0.009098	0.005007	0.004894	0.013206	0.010539	0.013064	0.023186	0.030227	0.040663
397	0.005645	0.001983	0.009013	0.005381	0.004954	0.0135	0.010809	0.013091	0.02355	0.030566	0.040867
396	0.005864	0.002094	0.009357	0.005634	0.004966	0.013382	0.01104	0.013295	0.023861	0.030607	0.041243
395	0.0058	0.001543	0.00902	0.005187	0.004702	0.013329	0.01077	0.01307	0.023283	0.030317	0.041163
394	0.005926	0.001971	0.009471	0.005595	0.005228	0.013627	0.010907	0.013576	0.023935	0.030649	0.041461
393	0.005597	0.00181	0.009215	0.005237	0.004872	0.01341	0.010674	0.013319	0.023736	0.030793	0.041594
392	0.005961	0.001828	0.009427	0.005502	0.005086	0.01371	0.011083	0.013275	0.02402	0.030799	0.041538
391	0.005869	0.001791	0.009436	0.005389	0.005108	0.013751	0.011028	0.013302	0.023815	0.031009	0.04176
390	0.00592	0.002398	0.009758	0.005823	0.005316	0.013937	0.011261	0.013803	0.024483	0.031718	0.042615
389	0.005948	0.001876	0.009695	0.005653	0.005413	0.014119	0.011465	0.014067	0.024784	0.031249	0.041851
388	0.005995	0.001963	0.009703	0.005696	0.005322	0.013481	0.010713	0.013045	0.024081	0.031181	0.041953
387	0.005944	0.001826	0.009702	0.005648	0.005049	0.013869	0.010991	0.013628	0.024289	0.031621	0.042347
386	0.006062	0.002093	0.010124	0.005507	0.005336	0.014256	0.011278	0.013706	0.024677	0.031907	0.042938
385	0.006219	0.001679	0.009721	0.005614	0.005245	0.014492	0.011273	0.013938	0.024914	0.032119	0.042912
384	0.006536	0.002025	0.009922	0.005582	0.005464	0.014336	0.011173	0.013832	0.02486	0.032112	0.04311
383	0.006504	0.002183	0.010098	0.005757	0.005536	0.014522	0.0115	0.014196	0.025207	0.031856	0.043302
382	0.006158	0.001698	0.009739	0.005468	0.005404	0.014321	0.011464	0.013898	0.025033	0.032202	0.043273
381	0.00635	0.00179	0.010078	0.00573	0.005315	0.014455	0.011294	0.014277	0.025239	0.032836	0.04372
380	0.006371	0.002028	0.010396	0.005766	0.005883	0.014752	0.011858	0.014412	0.02584	0.032743	0.044245
379	0.006729	0.00247	0.010406	0.006148	0.006094	0.01529	0.011748	0.014709	0.02591	0.033396	0.044543
378	0.00664	0.002288	0.010409	0.006028	0.005957	0.015174	0.011584	0.014786	0.026283	0.033334	0.044616
377	0.006878	0.001983	0.010798	0.006062	0.006015	0.015044	0.011884	0.014588	0.026203	0.033368	0.044743
376	0.006828	0.00251	0.010917	0.006098	0.006158	0.015522	0.011776	0.014941	0.026411	0.034167	0.045728
375	0.007237	0.002464	0.011104	0.005978	0.005773	0.015752	0.013152	0.015262	0.027005	0.032954	0.044823
374	0.006731	0.002321	0.010795	0.006123	0.005723	0.015627	0.01198	0.0149	0.026616	0.033946	0.045921

EXCLI Journal 2020;19:48-70 – ISSN 1611-2156
Received: October 03, 2019, accepted: December 19, 2019, published: January 03, 2020

Absorption intensity											
**Wavelength (nm)**	**concentration of acetylshikonin (M)**										
	A	*B*	*C*	*D*	*E*	*F*	*G*	*I*	*J*	*K*	*L*
	0.00	$8.0x10^{-7}$	$1.6x10^{-6}$	$2.0x10^{-6}$	$2.8x10^{-6}$	$4.0x10^{-6}$	$4.8x10^{-6}$	$6.0x10^{-6}$	$8.0x10^{-6}$	$1.2x10^{-5}$	$1.6x10^{-5}$
373	0.006828	0.002435	0.010961	0.006241	0.006358	0.015803	0.012436	0.015325	0.027167	0.034055	0.046414
372	0.007053	0.002254	0.010812	0.006294	0.006397	0.015476	0.011995	0.014881	0.026813	0.033826	0.045511
371	0.007245	0.002592	0.011345	0.006591	0.00632	0.015839	0.012507	0.015729	0.027265	0.034555	0.04651
370	0.006899	0.002273	0.011173	0.006234	0.005981	0.01588	0.012229	0.015	0.027003	0.034418	0.046663
369	0.007835	0.003261	0.011804	0.006468	0.006551	0.016413	0.012639	0.016051	0.027633	0.035012	0.047583
368	0.008203	0.00267	0.012289	0.006733	0.006747	0.016737	0.013101	0.015919	0.028242	0.035446	0.047859
367	0.006922	0.002395	0.011146	0.006591	0.007002	0.016142	0.012745	0.015707	0.02763	0.034414	0.047321
366	0.007676	0.002783	0.012072	0.0067	0.006546	0.01637	0.012395	0.015701	0.028186	0.035116	0.047599
365	0.007785	0.002641	0.011427	0.006554	0.006703	0.016843	0.012426	0.016083	0.027821	0.035085	0.047878
364	0.005962	0.001314	0.01039	0.005147	0.00505	0.015081	0.010952	0.014427	0.026552	0.034011	0.046568
363	0.007364	0.002286	0.01152	0.006636	0.006936	0.016447	0.013107	0.01583	0.028399	0.035474	0.048112
362	0.009661	0.004917	0.013815	0.009013	0.008435	0.019449	0.015166	0.017769	0.029263	0.036294	0.049327
361	0.007791	0.002738	0.012068	0.006567	0.006839	0.017376	0.013098	0.016019	0.028895	0.037058	0.049013
360	0.008065	0.002886	0.012042	0.007031	0.007289	0.017114	0.013398	0.017118	0.031123	0.038324	0.051795
359	0.006463	0.000951	0.010823	0.004912	0.005482	0.01586	0.012136	0.0147	0.027973	0.034741	0.047754
358	0.008462	0.003585	0.011844	0.006762	0.006706	0.017968	0.014206	0.016972	0.029791	0.037241	0.05038
357	0.009155	0.003717	0.013788	0.007558	0.007587	0.02033	0.014635	0.017996	0.03004	0.036604	0.049899
356	0.008912	0.00351	0.012415	0.007711	0.007176	0.017609	0.014091	0.017383	0.030269	0.037455	0.051032
355	0.008139	0.002937	0.012323	0.006472	0.007262	0.017267	0.01267	0.017572	0.032199	0.0399	0.053614
354	0.006049	0.001391	0.010062	0.003977	0.004953	0.014936	0.011477	0.014015	0.02852	0.034709	0.048185
353	0.008774	0.004806	0.014429	0.008096	0.007642	0.019469	0.014823	0.018682	0.031698	0.038977	0.053527
352	0.013014	0.006762	0.016849	0.010745	0.01031	0.022468	0.017587	0.022155	0.031196	0.038877	0.052886
351	0.01011	0.002234	0.012878	0.006998	0.007639	0.016862	0.013749	0.017573	0.034021	0.041324	0.054845
350	0.004584	1.03E-05	0.008698	0.002853	0.004326	0.014506	0.010677	0.014872	0.028069	0.036127	0.050643
349	0.009479	0.004605	0.014185	0.006157	0.007223	0.019978	0.015325	0.019325	0.031218	0.037621	0.053259
348	0.011468	0.003615	0.015085	0.007987	0.008119	0.020361	0.014496	0.019007	0.033512	0.040758	0.055355
347	0.011169	0.003045	0.015361	0.008511	0.007797	0.020321	0.015633	0.018629	0.032851	0.041187	0.055849
346	0.011387	0.004453	0.015151	0.00818	0.008316	0.021291	0.015817	0.019246	0.034485	0.041573	0.055556
345	0.011023	0.003209	0.015096	0.007644	0.00781	0.020138	0.014817	0.01848	0.033442	0.041107	0.055454
344	0.011495	0.003903	0.015658	0.008252	0.008338	0.021148	0.015684	0.019552	0.034633	0.042001	0.056932
343	0.011889	0.003982	0.015437	0.008975	0.008787	0.021674	0.015396	0.019331	0.035622	0.042297	0.057622
342	0.011499	0.004095	0.015594	0.008388	0.008414	0.021137	0.015771	0.019894	0.0348	0.04235	0.058006
341	0.011419	0.004153	0.016029	0.008451	0.008863	0.022278	0.016315	0.019706	0.03541	0.043143	0.058765

EXCLI Journal 2020;19:48-70 – ISSN 1611-2156
Received: October 03, 2019, accepted: December 19, 2019, published: January 03, 2020

Absorption intensity											
**Wavelength (nm)**	**concentration of acetylshikonin (M)**										
	A	*B*	*C*	*D*	*E*	*F*	*G*	*I*	*J*	*K*	*L*
	0.00	$8.0x10^{-7}$	$1.6x10^{-6}$	$2.0x10^{-6}$	$2.8x10^{-6}$	$4.0x10^{-6}$	$4.8x10^{-6}$	$6.0x10^{-6}$	$8.0x10^{-6}$	$1.2x10^{-5}$	$1.6x10^{-5}$
340	0.012021	0.004321	0.016137	0.008868	0.008536	0.021929	0.016347	0.020429	0.035854	0.043676	0.059683
339	0.012592	0.004449	0.016457	0.009554	0.009055	0.022867	0.017355	0.020524	0.036666	0.04436	0.060679
338	0.01245	0.004208	0.016391	0.008884	0.008628	0.022441	0.016353	0.020056	0.03649	0.04421	0.060554
337	0.012268	0.004445	0.016812	0.009131	0.009102	0.022307	0.016491	0.020479	0.037026	0.045116	0.061061
336	0.012164	0.004569	0.016517	0.009138	0.00891	0.022698	0.016448	0.020591	0.036889	0.045028	0.061884
335	0.012598	0.004516	0.016525	0.008961	0.008853	0.023033	0.017421	0.021011	0.037633	0.045931	0.063001
334	0.012494	0.004485	0.017455	0.009494	0.009369	0.023496	0.017445	0.021439	0.038152	0.046688	0.063761
333	0.013155	0.004762	0.017128	0.009706	0.009769	0.024105	0.017053	0.021458	0.038981	0.04725	0.064412
332	0.012417	0.00433	0.017244	0.009254	0.009545	0.02389	0.017443	0.021824	0.03899	0.047767	0.065305
331	0.013167	0.005203	0.018322	0.0099	0.010197	0.024555	0.018025	0.022151	0.039889	0.049032	0.066357
330	0.013866	0.005481	0.018342	0.010593	0.010212	0.025037	0.018213	0.022723	0.040703	0.049176	0.067341
329	0.013254	0.005551	0.017783	0.010317	0.010543	0.02467	0.018739	0.023525	0.040598	0.049493	0.068208
328	0.013415	0.005408	0.018345	0.010533	0.010623	0.025881	0.018556	0.023659	0.04163	0.050323	0.06922
327	0.013724	0.005096	0.018877	0.010634	0.010852	0.026246	0.019672	0.024458	0.042254	0.051962	0.070708
326	0.014028	0.005683	0.018709	0.010985	0.011019	0.025594	0.018804	0.024475	0.042359	0.051918	0.071121
325	0.013977	0.005673	0.018931	0.010797	0.011017	0.026566	0.020047	0.025163	0.043188	0.052891	0.072631
324	0.014133	0.006001	0.019297	0.010826	0.011	0.026686	0.020153	0.024973	0.043814	0.054095	0.073797
323	0.014419	0.005508	0.019325	0.011264	0.011805	0.027705	0.020694	0.025775	0.044811	0.055169	0.075384
322	0.014106	0.005875	0.018823	0.011115	0.011374	0.027811	0.020482	0.025797	0.045348	0.055349	0.076217
321	0.014589	0.006354	0.019729	0.011504	0.011827	0.028541	0.020864	0.027103	0.046064	0.056545	0.077565
320	0.014686	0.005944	0.01976	0.012116	0.012289	0.028497	0.021294	0.027283	0.046867	0.05792	0.079335
319	0.01522	0.006218	0.020427	0.011945	0.012434	0.029443	0.022138	0.027692	0.047813	0.058511	0.080884
318	0.015101	0.00673	0.020313	0.01178	0.012475	0.029802	0.021887	0.027789	0.048031	0.059537	0.082111
317	0.015717	0.007262	0.020917	0.012688	0.013247	0.030127	0.023019	0.029007	0.04932	0.061295	0.083855
316	0.015385	0.006675	0.020863	0.012883	0.013438	0.03068	0.02323	0.029348	0.049809	0.062018	0.08572
315	0.015639	0.007191	0.020949	0.01328	0.013676	0.031313	0.023772	0.029516	0.050848	0.06314	0.087087
314	0.01602	0.007233	0.021689	0.013348	0.014062	0.031782	0.024126	0.030916	0.052099	0.064764	0.088938
313	0.016156	0.006855	0.021618	0.013936	0.014137	0.032344	0.024124	0.031678	0.052711	0.06556	0.090245
312	0.016199	0.006979	0.021771	0.013122	0.014159	0.032635	0.024707	0.031611	0.053471	0.066735	0.091796
311	0.016434	0.007659	0.022374	0.014199	0.014753	0.033293	0.02475	0.032357	0.054247	0.067973	0.093805
310	0.0172	0.008291	0.023233	0.014357	0.015401	0.034514	0.025965	0.033477	0.055726	0.069333	0.095644
309	0.017256	0.007848	0.022812	0.01422	0.01503	0.03458	0.026134	0.033467	0.056114	0.070355	0.097139
308	0.017528	0.008436	0.023485	0.015304	0.015824	0.035472	0.026959	0.034699	0.057687	0.072248	0.099573

EXCLI Journal 2020;19:48-70 – ISSN 1611-2156
Received: October 03, 2019, accepted: December 19, 2019, published: January 03, 2020

Absorption intensity											
**Wavelength (nm)**	**concentration of acetylshikonin (M)**										
	A	*B*	*C*	*D*	*E*	*F*	*G*	*I*	*J*	*K*	*L*
	0.00	$8.0x10^{-7}$	$1.6x10^{-6}$	$2.0x10^{-6}$	$2.8x10^{-6}$	$4.0x10^{-6}$	$4.8x10^{-6}$	$6.0x10^{-6}$	$8.0x10^{-6}$	$1.2x10^{-5}$	$1.6x10^{-5}$
307	0.017507	0.007971	0.023725	0.015266	0.016042	0.035615	0.027319	0.035483	0.058859	0.073765	0.100893
306	0.017497	0.008045	0.023879	0.015205	0.016371	0.036246	0.027605	0.035349	0.059134	0.074781	0.102704
305	0.017839	0.008488	0.02444	0.015903	0.01655	0.037339	0.028322	0.036115	0.060366	0.076105	0.104535
304	0.018871	0.009062	0.02449	0.01616	0.017077	0.037852	0.028799	0.036861	0.061455	0.076985	0.106405
303	0.018693	0.009116	0.025264	0.016471	0.016928	0.037793	0.029225	0.037396	0.062003	0.078392	0.108089
302	0.019	0.008982	0.025319	0.016391	0.017353	0.03871	0.029522	0.037906	0.063199	0.079739	0.109709
301	0.019064	0.009004	0.025498	0.016137	0.017823	0.039077	0.029676	0.038612	0.063969	0.080576	0.111106
300	0.019993	0.009316	0.025684	0.017225	0.018718	0.039615	0.030612	0.03914	0.065216	0.082122	0.113207
299	0.020246	0.009493	0.026596	0.017654	0.01886	0.04031	0.031051	0.039767	0.065764	0.082996	0.114301
298	0.020331	0.009743	0.026391	0.017743	0.018393	0.040057	0.031225	0.039906	0.066203	0.084082	0.115564
297	0.020372	0.00956	0.02651	0.01735	0.018788	0.040996	0.031677	0.040831	0.066794	0.084795	0.117185
296	0.020772	0.010111	0.026801	0.017804	0.01923	0.041806	0.032106	0.041007	0.068114	0.086109	0.118608
295	0.021062	0.01008	0.027207	0.018196	0.019311	0.042199	0.032301	0.041535	0.068483	0.086879	0.120028
294	0.021145	0.009821	0.027341	0.018255	0.019389	0.042247	0.032838	0.042053	0.069536	0.088052	0.121575
293	0.021797	0.009992	0.027911	0.018675	0.019576	0.043216	0.033229	0.042651	0.070114	0.089061	0.122787
292	0.022632	0.010492	0.028231	0.018935	0.019945	0.043997	0.033808	0.043567	0.071294	0.090264	0.124007
291	0.022409	0.01048	0.028177	0.018859	0.019785	0.043491	0.033447	0.043066	0.071589	0.090702	0.124658
290	0.022858	0.010599	0.028868	0.019277	0.020167	0.044111	0.034161	0.043931	0.072092	0.091542	0.125588
289	0.023511	0.010734	0.028966	0.019739	0.020602	0.044744	0.034753	0.044827	0.073064	0.09269	0.128079
288	0.023802	0.010912	0.029007	0.019595	0.02077	0.044795	0.03474	0.044599	0.073497	0.093483	0.128918
287	0.024149	0.010964	0.029333	0.019924	0.020811	0.045259	0.035219	0.04505	0.074183	0.094142	0.129909
286	0.024296	0.01089	0.029372	0.020127	0.021078	0.045691	0.03544	0.045441	0.074604	0.095001	0.131496
285	0.023933	0.010889	0.029504	0.020218	0.021293	0.046114	0.035439	0.045784	0.075055	0.095878	0.131942
284	0.025101	0.011355	0.029981	0.02061	0.021479	0.046755	0.036079	0.046276	0.076395	0.096794	0.13318
283	0.025014	0.011349	0.030174	0.020823	0.02175	0.046859	0.036053	0.046375	0.076433	0.097219	0.134569
282	0.025088	0.011341	0.030074	0.020532	0.021654	0.046951	0.036398	0.046472	0.077037	0.098033	0.135149
281	0.025808	0.011803	0.03086	0.021591	0.022098	0.048033	0.037203	0.047591	0.077933	0.099305	0.136691
280	0.025859	0.011707	0.031051	0.021551	0.02225	0.047691	0.037235	0.047616	0.078359	0.099747	0.137429
279	0.026099	0.011742	0.031351	0.021759	0.022434	0.048417	0.037836	0.048145	0.078893	0.100591	0.138646
278	0.026353	0.012196	0.03156	0.021927	0.022859	0.048774	0.038281	0.048635	0.079817	0.101411	0.139437
277	0.026671	0.011916	0.031638	0.022196	0.022913	0.049349	0.038465	0.049189	0.080362	0.102635	0.140988
276	0.026627	0.012108	0.031653	0.02231	0.023097	0.049192	0.038633	0.04951	0.081208	0.103327	0.142032
275	0.026613	0.012226	0.031967	0.022268	0.023321	0.049947	0.039098	0.049811	0.081922	0.104145	0.143153

EXCLI Journal 2020;19:48-70 – ISSN 1611-2156
Received: October 03, 2019, accepted: December 19, 2019, published: January 03, 2020

Absorption intensity											
**Wavelength (nm)**	**concentration of acetylshikonin (M)**										
	A	*B*	*C*	*D*	*E*	*F*	*G*	*I*	*J*	*K*	*L*
	0.00	8.0x10^{-7}	1.6x10^{-6}	2.0x10^{-6}	2.8x10^{-6}	4.0x10^{-6}	4.8x10^{-6}	6.0x10^{-6}	8.0x10^{-6}	1.2x10^{-5}	1.6x10^{-5}
274	0.027241	0.012604	0.032441	0.023078	0.023922	0.050803	0.039821	0.05079	0.083002	0.1061	0.145341
273	0.02733	0.012671	0.032879	0.023084	0.024261	0.050919	0.040572	0.051326	0.083691	0.10733	0.146788
272	0.02755	0.012685	0.033073	0.023449	0.024426	0.051981	0.041217	0.051947	0.085093	0.108775	0.149239
271	0.027337	0.012849	0.033622	0.023676	0.024956	0.052406	0.041837	0.052691	0.086167	0.110236	0.151354
270	0.027755	0.013312	0.034036	0.024201	0.025647	0.053164	0.04252	0.053793	0.087224	0.112389	0.153885
269	0.027933	0.013371	0.03452	0.024716	0.026155	0.054214	0.043469	0.055012	0.08923	0.114768	0.156662
268	0.027848	0.013435	0.034634	0.024835	0.02666	0.054918	0.044045	0.055756	0.090675	0.116675	0.160012
267	0.027945	0.013637	0.035012	0.025098	0.02712	0.055951	0.045056	0.057364	0.092474	0.119254	0.163291
266	0.028389	0.014111	0.035964	0.026117	0.028286	0.05722	0.046599	0.058903	0.094765	0.122723	0.167578
265	0.028344	0.014305	0.036606	0.026739	0.028941	0.058551	0.048161	0.060534	0.096912	0.125634	0.171829
264	0.028593	0.014789	0.037141	0.027229	0.02975	0.060001	0.049363	0.062139	0.099266	0.12909	0.17648
263	0.028657	0.015065	0.037952	0.027751	0.030608	0.061408	0.050563	0.064194	0.101954	0.132799	0.181388
262	0.028745	0.015554	0.038581	0.028349	0.031726	0.062915	0.052531	0.065885	0.104688	0.136622	0.18657
261	0.028989	0.015517	0.039215	0.029334	0.032701	0.064258	0.05375	0.06743	0.106988	0.140217	0.191065
260	0.029268	0.016077	0.040221	0.030008	0.033731	0.066021	0.055498	0.069917	0.110257	0.14451	0.197106
259	0.029349	0.01659	0.040887	0.030557	0.034785	0.06784	0.057187	0.072163	0.112986	0.148337	0.202093
258	0.029957	0.017411	0.04237	0.031873	0.036225	0.069961	0.059253	0.074472	0.11643	0.153724	0.208815
257	0.030127	0.017598	0.04326	0.032691	0.037487	0.071907	0.061403	0.077016	0.120168	0.158787	0.215356
256	0.030215	0.018243	0.044118	0.033685	0.039005	0.074396	0.063889	0.079612	0.123819	0.164395	0.223153
255	0.030738	0.018797	0.045297	0.034954	0.040455	0.07691	0.065964	0.08304	0.128817	0.17073	0.231954
254	0.030863	0.019346	0.046693	0.035714	0.042056	0.079548	0.068964	0.086104	0.133585	0.177799	0.241352
253	0.031378	0.020026	0.048092	0.037622	0.044339	0.083045	0.072185	0.090488	0.139249	0.186194	0.252232
252	0.03164	0.020815	0.049752	0.039215	0.046605	0.086436	0.075986	0.095127	0.145547	0.195363	0.264304
251	0.032057	0.021645	0.051764	0.04091	0.049094	0.090607	0.080117	0.100011	0.152646	0.205837	0.278313
250	0.032408	0.022403	0.053567	0.04268	0.051799	0.094986	0.08456	0.105535	0.160205	0.217134	0.293784
249	0.032716	0.023293	0.055642	0.044607	0.055076	0.099518	0.090042	0.112095	0.168997	0.230511	0.31189
248	0.033408	0.024782	0.058521	0.047576	0.058893	0.105513	0.096031	0.120066	0.180018	0.246721	0.333372
247	0.033862	0.026648	0.061705	0.051102	0.063963	0.113097	0.103946	0.129747	0.193266	0.266075	0.359501
246	0.034563	0.028173	0.06536	0.055125	0.06949	0.121477	0.113298	0.140679	0.208088	0.289545	0.39107
245	0.035158	0.030473	0.069666	0.060017	0.076682	0.132225	0.124947	0.154791	0.227773	0.318983	0.4304
244	0.035421	0.032921	0.075376	0.066079	0.085479	0.145441	0.139138	0.172343	0.251828	0.355814	0.480053
243	0.036402	0.036262	0.081914	0.073913	0.096823	0.162372	0.157828	0.195327	0.283216	0.40366	0.544492
242	0.036757	0.040583	0.090821	0.084037	0.111651	0.184587	0.18276	0.225204	0.324333	0.466899	0.628342

EXCLI Journal 2020;19:48-70 – ISSN 1611-2156
Received: October 03, 2019, accepted: December 19, 2019, published: January 03, 2020

Absorption intensity											
**Wavelength (nm)**	**concentration of acetylshikonin (M)**										
	A	*B*	*C*	*D*	*E*	*F*	*G*	*I*	*J*	*K*	*L*
	0.00	$8.0x10^{-7}$	$1.6x10^{-6}$	$2.0x10^{-6}$	$2.8x10^{-6}$	$4.0x10^{-6}$	$4.8x10^{-6}$	$6.0x10^{-6}$	$8.0x10^{-6}$	$1.2x10^{-5}$	$1.6x10^{-5}$
241	0.037427	0.046433	0.102977	0.09834	0.131956	0.214347	0.215975	0.265674	0.379739	0.551157	0.74127
240	0.038428	0.054731	0.119445	0.117461	0.159685	0.255324	0.261575	0.320954	0.454223	0.665161	0.892073
239	0.03911	0.065472	0.141517	0.143671	0.197383	0.311198	0.324393	0.396256	0.556061	0.819096	1.094318
238	0.039601	0.080758	0.171702	0.180084	0.250097	0.38767	0.41017	0.498927	0.694022	1.026393	1.361406
237	0.040891	0.102441	0.214173	0.230852	0.323418	0.494025	0.528999	0.640859	0.881862	1.302879	1.714925
236	0.04179	0.132266	0.27273	0.301416	0.424213	0.638051	0.69022	0.832467	1.13359	1.666183	2.174962
235	0.042756	0.173732	0.354432	0.399008	0.562575	0.833909	0.908669	1.089607	1.46609	2.137224	2.747175
234	0.043574	0.230649	0.465179	0.53189	0.749382	1.094396	1.19901	1.427627	1.899272	2.72435	3.4155
233	0.044603	0.309696	0.616143	0.711589	0.998818	1.438167	1.578229	1.866821	2.446607	3.396613	3.97455
232	0.045747	0.416284	0.817066	0.949736	1.324841	1.878591	2.061686	2.416785	3.092529	3.93202	4.26125
231	0.046854	0.559664	1.081421	1.26253	1.744216	2.431368	2.656469	3.061384	3.700804	4.195142	4.461719
230	0.048086	0.748075	1.424197	1.662342	2.273823	3.071016	3.310098	3.650273	4.058748	4.371791	4.621953
229	0.049454	0.994639	1.859298	2.166298	2.89546	3.6587	3.80459	3.985298	4.253575	4.529222	4.797188
228	0.050233	1.309398	2.393079	2.763653	3.502073	3.977164	4.058162	4.181785	4.422592	4.694867	4.858359
227	0.051559	1.705864	3.005571	3.373301	3.875502	4.214276	4.244109	4.343701	4.534686	4.829043	4.992587
226	0.052632	2.191792	3.580099	3.828154	4.089678	4.336477	4.377662	4.491812	4.720993	4.902843	4.938063
225	0.054158	2.759304	3.931683	4.029515	4.247637	4.475837	4.480951	4.602346	4.771504	4.995928	5.113086
224	0.0554	3.33787	4.113474	4.197752	4.37511	4.564888	4.625558	4.712688	4.857136	4.928716	5.037319
223	0.056492	3.767295	4.272168	4.331486	4.516704	4.742583	4.687209	4.806043	4.93861	5.190909	5.114508
222	0.058271	3.98802	4.407368	4.440458	4.565964	4.779536	4.86714	4.844418	5.047618	5.007735	5.206413
221	0.059271	4.151614	4.557677	4.555075	4.695837	4.840025	4.904161	4.926235	5.041883	5.096681	5.335345
220	0.060928	4.243774	4.602623	4.585141	4.664256	4.760172	4.832053	5.118649	5.017657	4.981857	5.236989
219	0.061972	4.355046	4.669218	4.718837	4.875319	4.841339	4.911805	4.95199	4.931334	5.20016	5.252667
218	0.063679	4.457825	4.764201	4.694811	4.996018	4.995879	5.050457	4.947565	5.069852	5.256153	5.123457
217	0.06628	4.567653	4.795297	4.760608	4.934218	5.045606	4.98627	5.482172	5.180893	5.181238	5.226766
216	0.067669	4.541896	4.722898	4.770232	4.965781	4.927829	4.909957	5.146758	5.089884	5.131662	5.177375
215	0.070378	4.665956	4.718512	4.841252	4.876155	4.779197	4.885054	5.05243	4.866421	5.515617	10
214	0.072845	4.721344	4.860165	4.732336	4.890402	5.105146	5.105223	4.770793	10	5.201888	5.011395
213	0.075772	4.724882	5.001889	4.749892	4.917501	5.448724	5.1935	5.302589	5.00122	5.086928	5.334606
212	0.07883	4.627699	4.770884	4.750329	5.11524	5.032108	5.439179	5.032081	5.279219	10	10
211	0.082006	4.934863	4.875872	4.647594	5.082484	4.714128	6.036609	5.16175	5.559095	6.337413	10
210	0.088544	4.765277	5.867688	5.389953	10	10	10	5.389909	10	10	10
209	0.091474	4.528656	5.473759	5.121169	5.511068	10	5.773441	5.348239	10	10	10

EXCLI Journal 2020;19:48-70 – ISSN 1611-2156
Received: October 03, 2019, accepted: December 19, 2019, published: January 03, 2020

Absorption intensity											
**Wavelength (nm)**	**concentration of acetylshikonin (M)**										
	A	*B*	*C*	*D*	*E*	*F*	*G*	*I*	*J*	*K*	*L*
	0.00	$8.0x10^{-7}$	$1.6x10^{-6}$	$2.0x10^{-6}$	$2.8x10^{-6}$	$4.0x10^{-6}$	$4.8x10^{-6}$	$6.0x10^{-6}$	$8.0x10^{-6}$	$1.2x10^{-5}$	$1.6x10^{-5}$
208	0.097115	4.587406	4.921319	4.650075	4.668984	4.834745	4.763501	4.844811	5.373573	4.78831	5.231777
207	0.103229	4.619186	4.616401	4.698661	4.666477	5.183623	4.73213	5.414184	4.642201	4.965752	4.96543
206	0.110065	4.387969	4.861994	4.436341	4.875328	4.789959	4.639466	5.021065	4.889596	4.99796	4.666622
205	0.114858	5.24621	10	4.753162	10	6.257208	4.653922	4.859632	5.301689	10	10
204	0.121698	4.448349	4.702156	4.838328	5.150629	4.972658	4.76928	4.750981	5.147317	10	10
203	0.124462	4.454919	4.264249	4.173515	4.403061	4.352596	4.574395	4.381596	4.789635	4.787808	4.463588
202	0.15226	3.856807	3.956817	3.839756	4.119326	4.21839	3.960944	4.287444	4.329987	4.427933	4.526482
201	0.120127	3.337156	3.381941	3.604744	3.796047	3.500993	3.421122	3.425857	3.72781	3.656421	3.968008
200	0.170178	2.881635	3.092948	2.868058	2.940796	3.06822	2.994173	2.866024	3.171747	3.156736	3.469814

EXCLI Journal 2020;19:48-70 – ISSN 1611-2156
Received: October 03, 2019, accepted: December 19, 2019, published: January 03, 2020

Table 5: Absorption intensity in the wavelength range of 200-800 nm for absorption spectra of different concentration of *β*-hydroxyisovalerylshikonin (*A*-*L*)

Wavelength (nm)	***Absorption intensity*** **concentration of *β*-hydroxyisovalerylshikonin (M)**										
	A	*B*	*C*	*D*	*E*	*F*	*G*	*I*	*J*	*K*	*L*
	0.00	$8.0x10^{-7}$	$1.6x10^{-6}$	$2.0x10^{-6}$	$2.8x10^{-6}$	$4.0x10^{-6}$	$4.8x10^{-6}$	$6.0x10^{-6}$	$8.0x10^{-6}$	$1.2x10^{-5}$	$1.6x10^{-5}$
800	9.62E-05	-0.00033	0.000371	5.47E-05	-0.00038	0.000306	-7.95E-05	-0.00029	0.000924	-4.89E-05	0.00091
799	-7.90E-05	-0.00043	0.00019	-0.0003	-0.0005	0.000157	-1.86E-05	-0.00043	0.000892	-0.00045	0.001081
798	-0.00047	-0.00074	-0.00013	-0.00082	-0.00081	-0.00013	-0.00078	-0.00091	0.00086	-0.00042	0.000548
797	0.000216	-0.00028	0.000213	-0.00035	-0.0005	0.000412	-9.99E-06	-4.54E-05	0.001128	-0.00042	0.00113
796	-0.00018	-0.00036	0.000349	-0.00045	-0.00069	0.000127	-0.00025	-0.00037	0.001084	-0.00021	0.000924
795	-0.00014	-0.00063	8.86E-05	-0.00076	-0.00052	0.000232	-0.00033	-0.0007	0.000869	-0.00057	0.000632
794	0.000197	-0.00024	0.000258	-0.00046	-7.41E-05	0.000383	2.60E-05	-0.00014	0.00161	6.03E-05	0.001228
793	-0.00011	-0.00013	0.000564	-0.00033	-0.00043	0.000409	-8.39E-05	-0.00029	0.001432	-0.00013	0.001114
792	-0.00025	-0.00059	-2.38E-05	-0.00041	-0.00055	0.000367	-0.00058	-0.00041	0.000797	-0.00037	0.00084
791	-3.57E-05	-0.00053	1.85E-05	-0.00042	-0.00069	0.000519	-0.00042	-0.00025	0.00093	-0.00026	0.00102
790	9.53E-05	-0.00027	0.000117	-0.00036	-0.00027	0.000326	-0.00032	-0.00061	0.000864	3.23E-05	0.001075
789	-0.00031	-0.00074	0.000233	-0.00066	-0.00088	-2.11E-05	-0.00039	-0.00056	0.000763	-0.00068	0.000904
788	-1.10E-05	-0.00055	-7.50E-05	-0.00058	-0.0007	0.00037	-0.00044	-0.00038	0.000917	-0.0004	0.000893
787	-2.79E-05	-0.00035	0.00011	-0.00038	-0.00034	0.000313	-6.46E-05	-0.00031	0.001166	-0.00025	0.001092
786	0.000379	-0.00034	8.13E-05	-7.24E-05	-0.00015	0.00056	2.51E-06	-0.00017	0.001411	-0.00024	0.001053
785	-0.00016	-0.00049	-1.35E-06	-0.00046	-0.00077	0.00023	-0.00033	-0.00052	0.000975	-0.00032	0.000762
784	9.03E-05	-0.00048	3.28E-05	-0.00046	-0.00074	0.000381	-0.00022	-0.00025	0.000918	-0.00031	0.001045
783	-0.0001	-0.00077	0.000123	-0.00045	-0.00054	0.000251	-0.00034	-0.00065	0.000976	-0.00036	0.000778
782	5.59E-05	-0.00029	0.00012	-0.00028	-0.00044	0.000252	-0.00013	-0.00027	0.00109	-0.00017	0.001116
781	-0.00011	-0.00045	0.000214	-0.0007	-0.00029	0.000285	-0.00014	-0.00062	0.000979	-0.00024	0.000997
780	-0.00024	-0.00078	-5.03E-05	-0.00065	-0.00094	0.00012	-0.00056	-0.00057	0.000765	-0.00056	0.000514
779	-0.00017	-0.00051	6.37E-05	-0.00039	-0.00034	0.000535	-0.00012	-0.00017	0.00128	-0.00021	0.001056
778	0.000114	-0.00054	0.000168	-0.00054	-0.00037	0.00024	-0.00015	-0.00023	0.000984	-0.00042	0.000896
777	8.49E-05	-0.00042	0.000287	-0.00023	-0.00013	0.000615	-9.36E-05	-7.15E-05	0.001452	-0.00018	0.001352
776	-8.57E-05	-0.00061	0.000168	-0.00032	-0.00057	0.000534	-5.39E-05	-0.00053	0.001148	-8.31E-05	0.00113
775	-5.63E-05	-0.00049	-2.46E-05	-0.0005	-0.0006	0.000287	-0.00044	-0.00055	0.001104	-0.00034	0.000886
774	8.99E-05	-0.00043	0.000284	-0.00057	-0.00051	0.000512	-0.00031	-0.00027	0.00101	-0.00015	0.000961
773	-0.00025	-0.00064	-0.00013	-0.00068	-0.00089	1.37E-05	-0.00077	-0.00081	0.000663	-0.0007	0.000824
772	0.000101	-0.00031	0.000123	-0.00028	-0.00042	0.000408	-1.48E-05	-0.00054	0.00116	-0.00016	0.001127
771	0.000163	-0.00046	2.44E-05	-0.0006	-0.00081	0.000543	-0.00029	-0.00057	0.001142	-0.00033	0.000966

EXCLI Journal 2020;19:48-70 – ISSN 1611-2156
Received: October 03, 2019, accepted: December 19, 2019, published: January 03, 2020

Wavelength (nm)	***Absorption intensity*** **concentration of β-hydroxyisovalerylshikonin (M)**									
	A	*B*	*C*	*D*	*E*	*F*	*G*	*I*	*J*	*K*	*L*
	0.00	8.0x10^{-7}	1.6x10^{-6}	2.0x10^{-6}	2.8x10^{-6}	4.0x10^{-6}	4.8x10^{-6}	6.0x10^{-6}	8.0x10^{-6}	1.2x10^{-5}	1.6x10^{-5}
770	-0.0002	-0.00046	0.000298	-0.00016	-0.00035	0.000318	-0.00023	-0.00036	0.00114	-0.00032	0.001061
769	0.000193	-0.00029	8.39E-05	-0.00018	-0.00037	0.000472	-0.00021	-0.00016	0.000982	6.73E-05	0.001131
768	0.00021	-0.00052	0.000179	-0.00038	-0.00049	0.00031	-0.00026	-0.00041	0.001102	-7.72E-05	0.001241
767	-5.77E-05	-0.00064	-0.00013	-0.00046	-0.00079	7.59E-05	-0.00032	-0.00056	0.000895	-0.0003	0.000823
766	0.000224	-0.00042	0.000135	-0.00031	-0.00054	0.000523	0.000122	-0.00034	0.001221	-0.0003	0.001128
765	0.000218	-0.00032	0.000351	-0.00039	-0.0003	0.000519	-0.00015	-0.00022	0.001533	-1.48E-05	0.001252
764	0.000198	-0.00051	0.000322	-0.00027	-0.0005	0.000663	-6.96E-05	-0.00025	0.001229	-0.00024	0.001175
763	1.79E-05	-0.00065	0.000246	-0.00041	-0.00061	0.000324	-0.0002	-0.00038	0.001195	-0.00022	0.001095
762	2.15E-06	-0.00041	3.12E-05	-0.00072	-0.0007	0.00023	-0.00039	-0.0007	0.000923	-0.00023	0.000925
761	0.000192	-0.00038	0.000144	-0.00048	-0.00048	0.000678	-0.00021	-0.00038	0.001201	-8.85E-05	0.001133
760	0.000304	-0.00021	0.000429	-0.00021	-0.00028	0.000687	-5.96E-05	-0.00043	0.001266	-9.64E-05	0.001505
759	0.00013	-0.00042	0.000227	-0.00051	-0.00067	0.000347	-0.00024	-0.00052	0.00116	-0.0003	0.000953
758	0.000328	-0.00027	0.000286	-0.00025	-0.00064	0.00067	-0.00017	-0.00014	0.001213	-0.00018	0.001373
757	-3.98E-05	-0.00043	0.000115	-0.0005	-0.00062	0.000441	-0.00042	-0.00057	0.001019	-0.00027	0.001159
756	0.00042	-0.00037	0.000264	-0.00025	-0.0004	0.000409	-0.00031	-0.00059	0.001379	-7.87E-05	0.001231
755	0.000112	-0.00059	8.70E-05	-0.00057	-0.0006	0.000503	-0.00036	-0.00059	0.001045	-0.00044	0.000917
754	0.000156	-0.00045	0.000142	-0.00058	-0.00053	0.000505	-0.00015	-0.00033	0.001289	-0.00021	0.001201
753	0.000246	-0.00036	0.000474	-0.00022	-0.00051	0.000671	-3.64E-05	-0.00027	0.001329	4.32E-06	0.001315
752	6.44E-05	-0.00062	-3.31E-06	-0.00051	-0.00058	0.000341	-0.00029	-0.00049	0.001338	-0.00039	0.001323
751	0.000294	-0.00022	0.000435	-0.00017	-0.00027	0.000617	-0.00021	-0.00027	0.001352	-4.61E-05	0.001533
750	7.90E-05	-0.00046	8.23E-05	-0.00028	-0.00066	0.000284	-0.00032	-0.00056	0.001313	-0.00033	0.001185
749	0.00019	-0.00071	5.83E-05	-0.00056	-0.00053	0.000514	-0.00018	-0.00061	0.001135	-0.00025	0.001125
748	1.48E-05	-0.00035	0.000244	-0.00031	-0.0005	0.0005	-5.03E-05	-0.00035	0.001394	-0.00017	0.001282
747	-8.44E-05	-0.00065	-1.41E-05	-0.00056	-0.00083	0.000264	-0.00048	-0.00056	0.001027	-0.00046	0.001051
746	0.000198	-0.00035	0.000459	-0.0003	-0.00044	0.000594	-0.00038	-0.00027	0.001281	-0.00017	0.001402
745	-5.54E-05	-0.00079	0.00019	-0.00062	-0.00067	0.0004	-0.00042	-0.00046	0.001195	-0.00037	0.001187
744	6.52E-06	-0.00051	0.000251	-0.0007	-0.00086	0.000397	-0.00024	-0.00058	0.000958	-0.00028	0.001117
743	0.000138	-0.0006	0.00012	-0.00052	-0.00047	0.000513	-0.00025	-0.00044	0.00114	-0.00013	0.001332
742	0.000314	-0.00037	0.000389	-0.00036	-0.00051	0.000565	-0.00029	-0.00041	0.001247	-9.85E-05	0.001507
741	0.000128	-0.0005	0.000166	-0.00065	-0.00066	0.000485	-6.73E-05	-0.00053	0.001179	-0.00027	0.001425
740	7.15E-05	-0.00064	9.71E-05	-0.00051	-0.00074	0.000267	-0.00037	-0.00067	0.00119	-0.00029	0.001291
739	-7.31E-05	-0.00036	0.000187	-0.00045	-0.00052	0.000531	-0.00033	-0.00049	0.001462	-0.00019	0.001567
738	0.000419	-0.00035	0.000311	-0.00038	-0.00051	0.000695	-5.80E-06	-0.00011	0.001686	0.00019	0.00196

EXCLI Journal 2020;19:48-70 – ISSN 1611-2156
Received: October 03, 2019, accepted: December 19, 2019, published: January 03, 2020

Absorption intensity											
**Wavelength (nm)**	**concentration of β-hydroxyisovalerylshikonin (M)**										
	A	*B*	*C*	*D*	*E*	*F*	*G*	*I*	*J*	*K*	*L*
	0.00	$8.0x10^{-7}$	$1.6x10^{-6}$	$2.0x10^{-6}$	$2.8x10^{-6}$	$4.0x10^{-6}$	$4.8x10^{-6}$	$6.0x10^{-6}$	$8.0x10^{-6}$	$1.2x10^{-5}$	$1.6x10^{-5}$
737	0.00018	-0.00055	0.000402	-0.00048	-0.00082	0.000523	-0.00022	-0.00021	0.001291	-2.13E-05	0.001633
736	-0.00012	-0.00065	-0.00012	-0.0007	-0.0008	0.000358	-0.00041	-0.00065	0.001123	-0.00025	0.001648
735	0.00028	-0.00029	0.000235	-0.00042	-0.00061	0.000525	-5.14E-05	-0.00029	0.00157	0.000203	0.001821
734	2.75E-05	-0.00059	7.52E-05	-0.00052	-0.00078	0.000275	-0.00013	-0.00044	0.001412	-7.02E-05	0.001878
733	0.000111	-0.00037	0.000439	-0.00029	-0.00046	0.000716	-3.83E-06	-0.00025	0.001614	0.000341	0.002122
732	0.000101	-0.00054	0.000416	-0.00049	-0.00055	0.000713	-0.0002	-0.00034	0.001671	0.000302	0.002195
731	6.14E-05	-0.00046	0.000359	-0.00058	-0.0006	0.000541	-0.00022	-0.00016	0.001517	0.000395	0.002264
730	0.000303	-0.00048	0.000404	-0.00039	-0.00048	0.00072	5.85E-05	-3.85E-05	0.001754	0.000536	0.002547
729	0.000213	-0.0004	0.000467	-0.00039	-0.00042	0.000746	8.73E-05	-0.00013	0.001764	0.000644	0.002686
728	0.000221	-0.00049	0.000428	-0.0004	-0.00037	0.000764	3.65E-05	8.05E-05	0.001842	0.000732	0.002723
727	0.000197	-0.00048	0.000367	-0.00028	-0.0004	0.000795	4.33E-05	1.53E-06	0.001945	0.000636	0.002608
726	0.000277	-0.00045	0.000446	-0.00021	-0.00039	0.000805	0.000193	7.17E-05	0.002097	0.000919	0.002858
725	0.00017	-0.00046	0.000338	-0.0005	-0.00054	0.000719	-7.57E-05	-0.00017	0.001769	0.000643	0.002655
724	0.000254	-0.00027	0.000373	-0.00036	-0.00032	0.000822	0.00016	-7.46E-06	0.002066	0.000796	0.002974
723	0.000284	-0.00034	0.000445	-0.00028	-0.00044	0.000775	0.000303	-4.31E-05	0.002072	0.000879	0.0031
722	0.000298	-0.00023	0.000508	-7.39E-05	-0.00029	0.001127	0.000147	0.000109	0.002285	0.000982	0.003158
721	0.000133	-0.00051	0.000407	-0.00028	-0.00025	0.000805	8.61E-05	0.00019	0.001965	0.00083	0.002947
720	0.000379	-0.0004	0.000436	-0.00038	-0.00047	0.000937	0.000144	7.24E-05	0.002257	0.000954	0.003297
719	0.000472	-0.00042	0.000476	-0.00022	-0.00036	0.001055	0.000136	0.000132	0.002132	0.001074	0.003213
718	0.000191	-0.00053	0.000483	-0.00053	-0.00054	0.000741	-1.69E-05	0.000132	0.001917	0.000897	0.003141
717	0.000379	-0.00047	0.000504	-0.0003	-0.00026	0.000896	0.000218	0.000206	0.002276	0.001101	0.003549
716	0.000307	-0.00039	0.000466	-0.00026	-0.00038	0.000979	0.000227	0.000112	0.00223	0.001113	0.003467
715	0.000494	-0.00027	0.000546	-0.00019	-0.00032	0.000881	0.000326	0.000276	0.002434	0.001144	0.00375
714	0.000405	-0.00033	0.000586	-0.0002	-0.00039	0.001003	0.000354	0.000231	0.002469	0.001189	0.003691
713	0.000467	-0.00043	0.000478	-0.00021	-0.00034	0.000989	0.000298	0.000452	0.002322	0.001218	0.003855
712	0.000392	-0.0004	0.00045	-0.00019	-0.00043	0.000985	0.000202	0.000176	0.002322	0.001166	0.003719
711	0.00044	-0.00037	0.00053	-0.00027	-0.00023	0.001088	0.000337	0.000477	0.002617	0.001344	0.00401
710	0.000474	-0.00025	0.000541	-0.00022	-0.00027	0.001031	0.000241	0.000428	0.002524	0.001362	0.003941
709	0.000515	-0.00041	0.0006	-0.00015	-0.00032	0.001012	0.000293	0.000413	0.002372	0.00155	0.004037
708	0.000453	-0.00032	0.000647	-0.00027	-0.0004	0.001178	0.000334	0.000482	0.002557	0.001455	0.004221
707	0.000407	-0.00029	0.00062	-0.00022	-0.00036	0.001271	0.000357	0.000382	0.002539	0.001508	0.004079
706	0.000543	-0.00026	0.000857	-8.67E-05	-0.00031	0.001361	0.000474	0.000447	0.002648	0.001643	0.004483
705	0.000472	-0.00044	0.000646	-0.00019	-0.00016	0.001257	0.000436	0.000503	0.002763	0.001589	0.004436

EXCLI Journal 2020;19:48-70 – ISSN 1611-2156
Received: October 03, 2019, accepted: December 19, 2019, published: January 03, 2020

Absorption intensity											
**Wavelength (nm)**	**concentration of β-hydroxyisovalerylshikonin (M)**										
	A	*B*	*C*	*D*	*E*	*F*	*G*	*I*	*J*	*K*	*L*
	0.00	$8.0x10^{-7}$	$1.6x10^{-6}$	$2.0x10^{-6}$	$2.8x10^{-6}$	$4.0x10^{-6}$	$4.8x10^{-6}$	$6.0x10^{-6}$	$8.0x10^{-6}$	$1.2x10^{-5}$	$1.6x10^{-5}$
704	0.000754	-4.71E-05	0.000727	-0.00012	-0.00033	0.0013	0.000577	0.000575	0.002864	0.001837	0.004595
703	0.000454	-0.00033	0.000643	-9.91E-05	-0.00034	0.001185	0.000656	0.000545	0.002871	0.001829	0.004614
702	0.000512	-0.00048	0.000804	-0.00017	-0.00038	0.001225	0.000378	0.000587	0.00272	0.001841	0.004604
701	0.000594	-0.00026	0.00078	-0.0003	-0.00039	0.001391	0.000533	0.00055	0.002841	0.001791	0.00458
700	0.000419	-0.00056	0.000625	-0.00038	-0.00034	0.001119	0.000406	0.000524	0.00283	0.001649	0.004619
699	0.000428	-0.00038	0.000786	-0.00019	-0.0003	0.001202	0.000339	0.000653	0.002752	0.001768	0.004891
698	0.000366	-0.00026	0.000628	-0.00025	-0.00011	0.001316	0.000527	0.000701	0.002822	0.001901	0.004717
697	0.000693	1.28E-05	0.000943	6.30E-05	-9.47E-05	0.00159	0.000654	0.00082	0.003149	0.002109	0.005194
696	0.000599	-0.00045	0.000821	-0.00036	-0.00034	0.001279	0.000626	0.000603	0.002837	0.001908	0.005169
695	0.000802	-0.00021	0.000919	7.65E-05	6.63E-06	0.001597	0.000883	0.00093	0.0032	0.002219	0.005484
694	0.000471	-0.00019	0.000768	-0.00012	-0.00025	0.001353	0.000573	0.000641	0.003192	0.002132	0.005445
693	0.000427	-0.00034	0.000777	-8.71E-05	-0.00035	0.001137	0.000624	0.000745	0.003053	0.002062	0.005471
692	0.000445	-0.00037	0.000836	-4.56E-05	-0.00013	0.001342	0.000631	0.000613	0.003161	0.002306	0.005626
691	0.000549	-0.00027	0.000863	-7.48E-05	-0.00039	0.001554	0.000805	0.00083	0.003518	0.002387	0.005748
690	0.000567	-0.00023	0.000899	-3.40E-05	-3.07E-05	0.00147	0.000551	0.000942	0.003332	0.002515	0.00585
689	0.000612	-0.00015	0.000724	-2.51E-05	-0.00015	0.001498	0.000707	0.001055	0.003483	0.002399	0.005876
688	0.000433	-0.00041	0.000689	-0.00034	-0.00042	0.001324	0.000541	0.00076	0.002844	0.002169	0.005596
687	0.000727	-0.00022	0.000809	0.000133	-0.00014	0.001588	0.000784	0.258327	0.003502	0.002675	0.006164
686	0.000448	-0.00023	0.000949	-8.80E-05	-0.00011	0.001636	0.000705	0.001073	0.00359	0.002663	0.006362
685	0.000621	-0.00025	0.000885	-0.00013	-0.00021	0.001624	0.000695	0.001011	0.00353	0.002712	0.006261
684	0.000487	-0.00032	0.00094	-8.69E-05	-0.00016	0.001631	0.000693	0.000979	0.00352	0.002762	0.006644
683	0.000642	-0.00036	0.001067	0.000105	0.000188	0.001814	0.000795	0.001276	0.003614	0.002917	0.006782
682	0.000577	-0.00058	0.000762	-0.00025	-0.00034	0.001514	0.000803	0.0009	0.003494	0.002732	0.006698
681	0.000515	-0.00026	0.000828	-0.00021	-0.00016	0.001583	0.000851	0.001193	0.003667	0.002835	0.006887
680	0.000486	-0.00051	0.000759	-0.00013	-0.00029	0.001637	0.000625	0.001108	0.003557	0.002767	0.006917
679	0.000407	-0.00036	0.000958	-0.00029	-0.00016	0.001712	0.000952	0.001087	0.003765	0.003007	0.00713
678	0.00054	-0.00011	0.001036	-7.90E-05	6.68E-06	0.00197	0.001094	0.001303	0.004089	0.003292	0.007393
677	0.000614	-5.27E-05	0.000999	6.84E-05	3.11E-05	0.001911	0.001103	0.0013	0.004021	0.003344	0.007585
676	0.000511	-0.00019	0.001073	-7.30E-05	-0.00018	0.001791	0.001127	0.001341	0.003935	0.00343	0.007624
675	0.000533	-7.82E-05	0.000833	8.36E-06	-0.00014	0.001816	0.001037	0.001246	0.004135	0.003371	0.007645
674	0.000534	-0.00028	0.001026	0.000183	-1.90E-05	0.001785	0.000991	0.001291	0.004129	0.003373	0.007956
673	0.000599	-0.00015	0.001055	0.000115	-0.0001	0.001986	0.001177	0.001623	0.004387	0.003568	0.008198
672	0.000628	-0.00011	0.00116	3.47E-05	-2.04E-05	0.00198	0.001058	0.001387	0.004079	0.003703	0.008223

EXCLI Journal 2020;19:48-70 – ISSN 1611-2156
Received: October 03, 2019, accepted: December 19, 2019, published: January 03, 2020

Absorption intensity											
**Wavelength (nm)**	**concentration of β-hydroxyisovalerylshikonin (M)**										
	A	*B*	*C*	*D*	*E*	*F*	*G*	*I*	*J*	*K*	*L*
	0.00	8.0x10^{-7}	1.6x10^{-6}	2.0x10^{-6}	2.8x10^{-6}	4.0x10^{-6}	4.8x10^{-6}	6.0x10^{-6}	8.0x10^{-6}	1.2x10^{-5}	1.6x10^{-5}
671	0.000515	-0.00018	0.001102	4.05E-05	4.60E-05	0.001938	0.001083	0.001462	0.004322	0.003722	0.008456
670	0.00067	-0.00026	0.001005	1.10E-05	-1.27E-05	0.002057	0.001308	0.001589	0.004334	0.003933	0.008523
669	0.000503	-0.00035	0.001101	-4.14E-05	-6.32E-05	0.002073	0.001279	0.001523	0.004288	0.003852	0.008525
668	0.000504	-0.00023	0.001242	0.000206	6.03E-05	0.002056	0.001297	0.00166	0.004551	0.003908	0.008887
667	0.000662	-0.00017	0.001303	0.000227	5.39E-05	0.002391	0.001594	0.001845	0.004517	0.004176	0.008981
666	0.000575	-0.00016	0.001173	0.000151	2.42E-05	0.002206	0.001501	0.001662	0.004645	0.0042	0.009264
665	0.000526	-0.00019	0.001227	0.000262	-2.51E-05	0.002139	0.001411	0.00172	0.004626	0.004311	0.009406
664	0.000578	-0.00018	0.001101	3.47E-05	4.23E-05	0.002156	0.001354	0.00183	0.00481	0.004336	0.009666
663	0.000574	-0.00017	0.001163	3.47E-05	2.60E-05	0.002049	0.001416	0.001811	0.004819	0.004381	0.009905
662	0.000503	-0.00016	0.001215	8.67E-05	-3.25E-05	0.002144	0.001399	0.001699	0.004843	0.004453	0.009849
661	0.000756	-0.0001	0.001243	0.000208	0.000268	0.00237	0.001665	0.002057	0.005104	0.004839	0.010381
660	0.000777	-7.39E-05	0.00134	0.000278	0.000115	0.002387	0.001712	0.002094	0.005131	0.004864	0.010526
659	0.000704	1.66E-05	0.001424	0.000454	0.000245	0.002562	0.001748	0.002252	0.005459	0.005183	0.010712
658	0.000647	-3.07E-05	0.001345	0.000125	0.000226	0.002418	0.001654	0.002143	0.005391	0.005148	0.010874
657	0.000663	-0.00018	0.001305	0.000147	0.000134	0.00244	0.001565	0.002105	0.005337	0.00512	0.010838
656	0.000618	-0.00014	0.001207	0.000225	0.000103	0.00241	0.00162	0.002132	0.005462	0.005141	0.011076
655	0.000718	7.39E-05	0.001362	0.000376	0.000316	0.00269	0.001778	0.002518	0.005706	0.005481	0.011459
654	0.00063	-0.00021	0.001395	0.000197	0.000266	0.002483	0.001783	0.002402	0.0057	0.00547	0.011696
653	0.000599	-0.00015	0.001347	0.00022	0.000249	0.002532	0.001873	0.002148	0.005715	0.005434	0.011746
652	0.000652	-0.00011	0.001451	0.000162	0.000332	0.002589	0.001784	0.002394	0.005674	0.005551	0.011966
651	0.000683	-1.44E-05	0.001391	0.000388	0.00032	0.00283	0.00201	0.002551	0.005912	0.005903	0.012281
650	0.00076	5.31E-05	0.001713	0.000403	0.000535	0.002896	0.00214	0.002675	0.006164	0.006107	0.01272
649	0.000696	-6.05E-05	0.001584	0.000243	0.000357	0.002746	0.002051	0.002732	0.006071	0.006176	0.012862
648	0.000798	0.00011	0.001484	0.000436	0.000496	0.002861	0.002329	0.002794	0.00635	0.006446	0.013207
647	0.000717	1.82E-05	0.001593	0.000508	0.000568	0.002994	0.002406	0.002927	0.006484	0.006508	0.013441
646	0.000884	0.000161	0.001666	0.00056	0.000596	0.003071	0.002309	0.002941	0.006524	0.006716	0.013816
645	0.000944	0.000122	0.001736	0.00047	0.000582	0.003022	0.00244	0.002995	0.006716	0.006907	0.013957
644	0.000837	0.000133	0.001734	0.000673	0.000609	0.003138	0.002409	0.003138	0.00678	0.00704	0.014242
643	0.000858	5.39E-05	0.001734	0.000506	0.000436	0.003109	0.002477	0.003262	0.006765	0.007076	0.014591
642	0.000761	1.14E-05	0.001459	0.000428	0.000393	0.003049	0.002503	0.003072	0.006937	0.007118	0.014738
641	0.000758	-2.32E-05	0.00175	0.000459	0.000503	0.003057	0.002513	0.00323	0.006959	0.007331	0.015003
640	0.00075	0.0001	0.001834	0.000634	0.000628	0.003237	0.002728	0.003319	0.007077	0.007528	0.015354
639	0.000728	9.49E-05	0.001743	0.000523	0.0007	0.00338	0.002741	0.003442	0.00728	0.007713	0.015614

Wavelength (nm)	***Absorption intensity***										
	**concentration of β-hydroxyisovalerylshikonin (M)**										
	A	*B*	*C*	*D*	*E*	*F*	*G*	*I*	*J*	*K*	*L*
	0.00	8.0x10^{-7}	1.6x10^{-6}	2.0x10^{-6}	2.8x10^{-6}	4.0x10^{-6}	4.8x10^{-6}	6.0x10^{-6}	8.0x10^{-6}	1.2x10^{-5}	1.6x10^{-5}
638	0.000832	3.53E-05	0.001988	0.000628	0.000668	0.003222	0.002702	0.003423	0.00757	0.007838	0.015896
637	0.000748	0.000165	0.001947	0.000682	0.000698	0.003487	0.002763	0.003689	0.007546	0.007971	0.016392
636	0.00073	0.000263	0.001887	0.00075	0.000626	0.0034	0.002851	0.003558	0.007677	0.008078	0.016493
635	0.00081	0.000153	0.001983	0.000688	0.000744	0.003507	0.002942	0.003789	0.007673	0.008348	0.016671
634	0.000827	0.000271	0.001991	0.00078	0.000814	0.003633	0.002977	0.00388	0.007818	0.008514	0.017032
633	0.000971	0.000307	0.001967	0.000712	0.000928	0.003643	0.00316	0.003917	0.008017	0.008618	0.017271
632	0.000798	0.00031	0.002066	0.000759	0.000925	0.003704	0.003194	0.004066	0.008099	0.008944	0.017779
631	0.000772	0.000168	0.002061	0.000759	0.000855	0.003793	0.003199	0.004004	0.00827	0.009065	0.018014
630	0.000923	0.00027	0.001998	0.000858	0.001032	0.003976	0.003283	0.004303	0.008401	0.009271	0.018283
629	0.000977	0.000403	0.002247	0.000958	0.000916	0.003981	0.003394	0.004327	0.008594	0.009611	0.018691
628	0.000861	0.000292	0.00205	0.000856	0.000959	0.003838	0.003427	0.004409	0.008529	0.00963	0.019015
627	0.000916	0.000298	0.002178	0.000821	0.001028	0.004039	0.003599	0.0045	0.008815	0.009873	0.019436
626	0.000753	0.000319	0.002217	0.000874	0.001095	0.004072	0.003503	0.004519	0.008841	0.009928	0.019611
625	0.000873	0.000375	0.002255	0.000986	0.001108	0.00417	0.003665	0.004728	0.009119	0.010197	0.019959
624	0.000752	0.000305	0.002166	0.000975	0.001092	0.004065	0.003605	0.004621	0.009088	0.010375	0.020168
623	0.001055	0.000438	0.002346	0.001007	0.001039	0.00423	0.003718	0.004814	0.009315	0.010455	0.020558
622	0.000935	0.000384	0.002299	0.001001	0.001228	0.004352	0.00393	0.005006	0.009491	0.010765	0.020799
621	0.00077	0.000222	0.002145	0.000862	0.001059	0.004171	0.003706	0.004813	0.009539	0.010916	0.021021
620	0.000871	0.000423	0.002483	0.001133	0.001229	0.004412	0.003878	0.005022	0.00976	0.011159	0.021597
619	0.000908	0.000471	0.002447	0.001119	0.0013	0.004477	0.003964	0.005276	0.009753	0.011312	0.021812
618	0.001006	0.000365	0.00237	0.001144	0.001186	0.004433	0.004065	0.00528	0.009907	0.011493	0.022142
617	0.001003	0.000472	0.002442	0.001103	0.001363	0.004628	0.004102	0.005336	0.010204	0.011566	0.02242
616	0.000819	0.000272	0.002462	0.000997	0.001284	0.004484	0.004159	0.005421	0.010203	0.011851	0.022695
615	0.001032	0.000382	0.00251	0.001195	0.001446	0.004783	0.004404	0.005481	0.010479	0.011997	0.023043
614	0.001077	0.00052	0.002575	0.001242	0.001387	0.00479	0.004386	0.00564	0.010528	0.012277	0.023337
613	0.001056	0.000444	0.002725	0.001208	0.001328	0.004805	0.004424	0.005653	0.010605	0.012326	0.023547
612	0.000971	0.000483	0.002624	0.001197	0.001547	0.004943	0.004624	0.00587	0.010782	0.012583	0.023833
611	0.001079	0.000662	0.002557	0.001164	0.001487	0.004865	0.004642	0.005949	0.010966	0.012827	0.024129
610	0.000987	0.000608	0.002729	0.001386	0.00144	0.004847	0.004597	0.005934	0.011066	0.012926	0.024451
609	0.000975	0.000513	0.002698	0.001425	0.001607	0.005157	0.004676	0.00612	0.011182	0.013127	0.024834
608	0.001029	0.000597	0.002645	0.001371	0.00161	0.00507	0.00469	0.006114	0.011228	0.013259	0.024979
607	0.001003	0.000591	0.00268	0.001346	0.001571	0.005055	0.004733	0.006169	0.011248	0.013427	0.025176
606	0.001065	0.00065	0.002851	0.001321	0.001617	0.005165	0.00484	0.00634	0.01144	0.013571	0.025524

Wavelength (nm)	***Absorption intensity***										
	**concentration of β-hydroxyisovalerylshikonin (M)**										
	A	*B*	*C*	*D*	*E*	*F*	*G*	*I*	*J*	*K*	*L*
	0.00	$8.0x10^{-7}$	$1.6x10^{-6}$	$2.0x10^{-6}$	$2.8x10^{-6}$	$4.0x10^{-6}$	$4.8x10^{-6}$	$6.0x10^{-6}$	$8.0x10^{-6}$	$1.2x10^{-5}$	$1.6x10^{-5}$
605	0.001059	0.000646	0.002904	0.001391	0.001672	0.00524	0.004894	0.006407	0.011586	0.013787	0.025856
604	0.00084	0.000534	0.002749	0.001357	0.001541	0.005125	0.004776	0.006383	0.011634	0.013812	0.025938
603	0.001078	0.00076	0.002964	0.001679	0.001883	0.005433	0.005133	0.006555	0.011925	0.014125	0.026468
602	0.001094	0.000788	0.00303	0.001617	0.001887	0.00535	0.005111	0.006711	0.012043	0.014288	0.026636
601	0.001044	0.000697	0.003	0.001514	0.001702	0.005527	0.005188	0.00664	0.012014	0.01447	0.026848
600	0.001029	0.000568	0.002838	0.001484	0.001891	0.005536	0.005131	0.006747	0.012141	0.014452	0.027122
599	0.001017	0.000743	0.003081	0.001435	0.001851	0.005586	0.005276	0.006802	0.012372	0.014801	0.02736
598	0.001113	0.00083	0.003127	0.001706	0.001946	0.00568	0.005492	0.006942	0.012565	0.015051	0.027735
597	0.000979	0.000644	0.003056	0.001557	0.001893	0.005621	0.005356	0.006948	0.012486	0.015018	0.02781
596	0.001117	0.000752	0.003232	0.001545	0.002022	0.005715	0.005667	0.007085	0.012775	0.015421	0.028335
595	0.001101	0.000832	0.003203	0.001679	0.001888	0.005963	0.005693	0.007225	0.01282	0.015605	0.028497
594	0.001086	0.000845	0.00325	0.001717	0.002103	0.005804	0.005682	0.007449	0.013028	0.015773	0.028877
593	0.001045	0.000823	0.003168	0.001749	0.002045	0.005921	0.00573	0.007299	0.013229	0.01595	0.029213
592	0.001144	0.000831	0.003269	0.001827	0.002114	0.006027	0.005911	0.007435	0.013366	0.016207	0.029348
591	0.000977	0.000901	0.00311	0.001907	0.002096	0.005989	0.005893	0.007512	0.01334	0.016248	0.02976
590	0.000976	0.000796	0.003187	0.00171	0.002124	0.006023	0.00576	0.00763	0.013469	0.016477	0.029775
589	0.001086	0.000829	0.003372	0.001906	0.002198	0.00615	0.006107	0.00768	0.01365	0.016645	0.030237
588	0.001	0.000856	0.003483	0.001894	0.002093	0.006075	0.005989	0.007846	0.013819	0.016798	0.030603
587	0.001076	0.000749	0.003342	0.001797	0.002304	0.006305	0.006218	0.007922	0.014019	0.017117	0.030817
586	0.001113	0.000852	0.003657	0.001908	0.002245	0.006407	0.006291	0.007883	0.014191	0.017323	0.031313
585	0.000984	0.000935	0.003485	0.001891	0.0022	0.006269	0.006311	0.008188	0.014145	0.017348	0.031363
584	0.00114	0.000942	0.003557	0.002086	0.002386	0.006573	0.006369	0.008456	0.014485	0.017827	0.031986
583	0.001	0.000689	0.003454	0.001807	0.002192	0.006607	0.00638	0.008187	0.014526	0.017962	0.03207
582	0.000917	0.000915	0.003645	0.001944	0.002276	0.006584	0.00659	0.008372	0.014658	0.018103	0.032417
581	0.000979	0.000974	0.003632	0.001966	0.002465	0.00677	0.006582	0.00849	0.014768	0.018299	0.03289
580	0.001212	0.000976	0.003746	0.00212	0.002638	0.006806	0.006961	0.008672	0.015135	0.018772	0.03332
579	0.001117	0.000999	0.003751	0.002079	0.002625	0.006967	0.006867	0.008778	0.015124	0.018954	0.033568
578	0.001124	0.000967	0.003812	0.002214	0.002721	0.007008	0.006974	0.008965	0.015445	0.019087	0.033967
577	0.001051	0.00105	0.003649	0.002203	0.002646	0.00704	0.0069	0.008868	0.015506	0.019234	0.033997
576	0.000889	0.000868	0.003619	0.002054	0.002373	0.006723	0.006962	0.008804	0.015457	0.019311	0.033971
575	0.001155	0.000915	0.003843	0.002251	0.00263	0.00713	0.006999	0.009159	0.015636	0.019709	0.034443
574	0.00094	0.000915	0.003699	0.002145	0.002517	0.007179	0.007195	0.009122	0.015806	0.019882	0.034729
573	0.001098	0.000956	0.003896	0.002252	0.002665	0.007225	0.007201	0.00926	0.016091	0.019958	0.035119

EXCLI Journal 2020;19:48-70 – ISSN 1611-2156
Received: October 03, 2019, accepted: December 19, 2019, published: January 03, 2020

Absorption intensity											
**Wavelength (nm)**	**concentration of β-hydroxyisovalerylshikonin (M)**										
	A	*B*	*C*	*D*	*E*	*F*	*G*	*I*	*J*	*K*	*L*
	0.00	$8.0x10^{-7}$	$1.6x10^{-6}$	$2.0x10^{-6}$	$2.8x10^{-6}$	$4.0x10^{-6}$	$4.8x10^{-6}$	$6.0x10^{-6}$	$8.0x10^{-6}$	$1.2x10^{-5}$	$1.6x10^{-5}$
572	0.001259	0.001228	0.003979	0.002475	0.002878	0.007424	0.007391	0.009532	0.016236	0.020022	0.035453
571	0.000934	0.000963	0.003854	0.002261	0.002826	0.007351	0.007369	0.009458	0.01621	0.020005	0.035628
570	0.001094	0.001163	0.004035	0.002318	0.002879	0.007417	0.007515	0.009664	0.016168	0.020338	0.035934
569	0.001211	0.001203	0.004011	0.259531	0.003003	0.007554	0.007592	0.009797	0.016395	0.020522	0.036294
568	0.001146	0.001249	0.004151	0.002483	0.00289	0.007634	0.007767	0.009877	0.016774	0.021144	0.037026
567	0.00101	0.001213	0.004037	0.002451	0.002982	0.007713	0.007791	0.010154	0.016851	0.021357	0.037195
566	0.001141	0.001069	0.004181	0.002489	0.003078	0.007708	0.007924	0.010153	0.01712	0.021462	0.037384
565	0.001063	0.001187	0.004104	0.002624	0.002939	0.007795	0.007824	0.010118	0.017202	0.021702	0.037604
564	0.001098	0.00126	0.004097	0.002525	0.003078	0.007875	0.007947	0.010224	0.017255	0.021734	0.037851
563	0.00086	0.001062	0.003988	0.002437	0.003084	0.007845	0.00792	0.010225	0.017347	0.02188	0.037844
562	0.000895	0.001099	0.004048	0.002566	0.002933	0.007831	0.007912	0.010317	0.017353	0.022067	0.038264
561	0.001171	0.001385	0.004541	0.00279	0.003397	0.008058	0.008251	0.01051	0.017622	0.022362	0.038591
560	0.000977	0.001167	0.004344	0.002535	0.00323	0.008172	0.008126	0.010663	0.017774	0.022442	0.038843
559	0.001173	0.001187	0.004258	0.002719	0.003503	0.008156	0.008379	0.01078	0.017995	0.022567	0.039017
558	0.001043	0.001188	0.004299	0.002533	0.003279	0.008211	0.008307	0.010818	0.01788	0.022812	0.039211
557	0.001003	0.001215	0.004472	0.002695	0.003318	0.008246	0.008499	0.010905	0.018208	0.023103	0.039501
556	0.001074	0.0012	0.004269	0.002638	0.003299	0.008309	0.008413	0.010769	0.018181	0.023194	0.039655
555	0.001162	0.001404	0.004474	0.002766	0.003413	0.008567	0.008483	0.011039	0.01827	0.023411	0.040073
554	0.0011	0.001342	0.00451	0.002858	0.003581	0.008601	0.008779	0.011102	0.018455	0.023675	0.040182
553	0.001218	0.001503	0.004525	0.002943	0.003681	0.008538	0.00884	0.011334	0.01874	0.023842	0.040597
552	0.001225	0.001504	0.004563	0.002869	0.003604	0.008617	0.008881	0.011371	0.018999	0.024057	0.040724
551	0.001124	0.001243	0.004517	0.002897	0.003513	0.00865	0.008811	0.011363	0.018806	0.024055	0.040715
550	0.001037	0.001367	0.004593	0.003084	0.003724	0.008766	0.008948	0.011553	0.019037	0.024342	0.040988
549	0.001188	0.001427	0.004768	0.002988	0.003666	0.00889	0.009079	0.011575	0.019188	0.024486	0.041324
548	0.001048	0.0014	0.004692	0.002936	0.003634	0.009028	0.009057	0.011725	0.019233	0.024754	0.041478
547	0.001139	0.001362	0.004787	0.003167	0.003833	0.008938	0.009195	0.011821	0.019439	0.024913	0.041774
546	0.001003	0.001433	0.004676	0.003087	0.003768	0.009079	0.009264	0.011875	0.019628	0.025089	0.041989
545	0.001057	0.001391	0.004858	0.003259	0.003803	0.009046	0.009399	0.011935	0.019773	0.025305	0.042281
544	0.00127	0.001315	0.004953	0.003241	0.003947	0.009067	0.009456	0.012176	0.019886	0.025413	0.042664
543	0.001163	0.001493	0.004873	0.003127	0.003907	0.009279	0.009509	0.012147	0.020025	0.025553	0.042773
542	0.001001	0.00132	0.00484	0.003096	0.003871	0.00925	0.009567	0.012209	0.020076	0.025734	0.042871
541	0.000943	0.001363	0.004813	0.003264	0.003919	0.009233	0.009571	0.01228	0.020197	0.026094	0.043205
540	0.000955	0.001332	0.004972	0.003236	0.004006	0.009361	0.009697	0.012507	0.020312	0.026186	0.043502

EXCLI Journal 2020;19:48-70 – ISSN 1611-2156
Received: October 03, 2019, accepted: December 19, 2019, published: January 03, 2020

Absorption intensity											
**Wavelength (nm)**	**concentration of β-hydroxyisovalerylshikonin (M)**										
	A	*B*	*C*	*D*	*E*	*F*	*G*	*I*	*J*	*K*	*L*
	0.00	$8.0x10^{-7}$	$1.6x10^{-6}$	$2.0x10^{-6}$	$2.8x10^{-6}$	$4.0x10^{-6}$	$4.8x10^{-6}$	$6.0x10^{-6}$	$8.0x10^{-6}$	$1.2x10^{-5}$	$1.6x10^{-5}$
539	0.001212	0.001708	0.005169	0.003542	0.00419	0.009825	0.009887	0.012752	0.020653	0.026553	0.043752
538	0.001061	0.00136	0.004999	0.003519	0.004094	0.00938	0.009865	0.012774	0.020707	0.026639	0.043946
537	0.001149	0.001526	0.005171	0.003247	0.004137	0.009617	0.009959	0.012875	0.020927	0.027002	0.044303
536	0.001166	0.001648	0.005072	0.003562	0.004276	0.009839	0.010282	0.012978	0.02108	0.027255	0.044495
535	0.001073	0.001422	0.005197	0.003536	0.004372	0.009696	0.010128	0.013038	0.021056	0.027357	0.044716
534	0.001221	0.001711	0.005228	0.003517	0.004352	0.009933	0.010405	0.01329	0.02131	0.02758	0.045215
533	0.001317	0.001641	0.005345	0.003558	0.004442	0.01004	0.010431	0.013245	0.021446	0.027773	0.04527
532	0.001158	0.001633	0.005389	0.003743	0.004482	0.010051	0.010525	0.013465	0.021678	0.027957	0.045693
531	0.001049	0.001482	0.0052	0.003625	0.004498	0.010131	0.010417	0.013444	0.021569	0.028074	0.045752
530	0.001369	0.001675	0.005331	0.003715	0.004443	0.01021	0.010683	0.013625	0.021853	0.028296	0.046043
529	0.000995	0.001548	0.005395	0.003682	0.004483	0.010237	0.010689	0.013638	0.021986	0.0285	0.046194
528	0.00106	0.001649	0.005468	0.003767	0.004596	0.010297	0.010742	0.0138	0.022035	0.028678	0.046385
527	0.00108	0.001886	0.00554	0.003841	0.004567	0.010453	0.011029	0.013785	0.022473	0.028964	0.046626
526	0.001084	0.001547	0.005621	0.003744	0.00469	0.010395	0.010841	0.013985	0.022328	0.029029	0.046871
525	0.001029	0.001542	0.005436	0.003933	0.00472	0.010615	0.011179	0.014108	0.022651	0.029234	0.047149
524	0.001193	0.001726	0.005565	0.003791	0.004709	0.010665	0.011046	0.014017	0.022714	0.029456	0.047197
523	0.001179	0.001735	0.005722	0.003998	0.004932	0.010657	0.011189	0.014343	0.022746	0.029601	0.047539
522	0.001161	0.001925	0.005876	0.004129	0.004869	0.010834	0.011247	0.014421	0.023063	0.029942	0.047858
521	0.001122	0.001706	0.00574	0.003938	0.004793	0.010853	0.011245	0.014355	0.023062	0.029965	0.04775
520	0.001009	0.00171	0.005694	0.003918	0.004839	0.010758	0.011268	0.014417	0.023173	0.030003	0.047866
519	0.001087	0.001657	0.005905	0.004135	0.005051	0.010921	0.011368	0.014721	0.023429	0.030389	0.048289
518	0.001034	0.001703	0.005858	0.004136	0.004981	0.011068	0.011649	0.014624	0.02345	0.030407	0.048348
517	0.001046	0.001853	0.005958	0.004274	0.005081	0.010988	0.011598	0.014723	0.02357	0.030618	0.048619
516	0.001252	0.001832	0.00599	0.00408	0.005201	0.011237	0.011679	0.014859	0.023672	0.030775	0.048906
515	0.001245	0.001771	0.006066	0.004104	0.00504	0.011176	0.011689	0.014878	0.023742	0.030821	0.048719
514	0.001082	0.001799	0.005991	0.004261	0.005165	0.011284	0.011639	0.015	0.023835	0.031066	0.049042
513	0.00119	0.001827	0.006064	0.00421	0.005249	0.011375	0.01197	0.015011	0.023951	0.031212	0.04935
512	0.001443	0.001882	0.006158	0.004554	0.005418	0.011491	0.012126	0.015198	0.024291	0.031532	0.049534
511	0.001141	0.001746	0.006052	0.004264	0.005276	0.011403	0.012059	0.015295	0.024169	0.031368	0.049498
510	0.001189	0.0019	0.006103	0.004239	0.005376	0.011633	0.011927	0.01511	0.024438	0.031661	0.049627
509	0.001097	0.001849	0.006216	0.004282	0.005322	0.011619	0.012008	0.0154	0.024435	0.031685	0.049955
508	0.001215	0.001894	0.006238	0.0044	0.005398	0.011523	0.012235	0.015445	0.024559	0.031888	0.049896
507	0.001007	0.001836	0.006109	0.004378	0.005401	0.011708	0.012159	0.015493	0.024665	0.031971	0.050137

EXCLI Journal 2020;19:48-70 – ISSN 1611-2156
Received: October 03, 2019, accepted: December 19, 2019, published: January 03, 2020

Absorption intensity											
**Wavelength (nm)**	**concentration of β-hydroxyisovalerylshikonin (M)**										
	A	*B*	*C*	*D*	*E*	*F*	*G*	*I*	*J*	*K*	*L*
	0.00	8.0x10^{-7}	1.6x10^{-6}	2.0x10^{-6}	2.8x10^{-6}	4.0x10^{-6}	4.8x10^{-6}	6.0x10^{-6}	8.0x10^{-6}	1.2x10^{-5}	1.6x10^{-5}
506	0.001114	0.001882	0.006282	0.004423	0.005416	0.01175	0.012411	0.015514	0.024737	0.03225	0.050183
505	0.001235	0.001936	0.006381	0.004643	0.005539	0.011936	0.012481	0.015693	0.024969	0.032402	0.050673
504	0.000848	0.001636	0.0063	0.004211	0.005263	0.011832	0.012188	0.015528	0.024723	0.032124	0.050448
503	0.000964	0.001803	0.006322	0.004476	0.005562	0.011847	0.012527	0.015578	0.024978	0.032426	0.050666
502	0.001036	0.001829	0.006423	0.004498	0.005527	0.011915	0.012729	0.015956	0.025169	0.032705	0.050995
501	0.001056	0.001758	0.006305	0.004593	0.005502	0.011931	0.012601	0.015806	0.025144	0.032633	0.051017
500	0.000927	0.001844	0.006574	0.004489	0.005568	0.012129	0.01266	0.01586	0.025387	0.032961	0.051125
499	0.001042	0.001665	0.006496	0.004476	0.005586	0.011945	0.01261	0.015928	0.02535	0.032841	0.051228
498	0.000758	0.001756	0.006262	0.004363	0.005426	0.01198	0.012419	0.015936	0.025299	0.032949	0.051327
497	0.001069	0.001838	0.006514	0.004648	0.005734	0.01226	0.012678	0.015915	0.025587	0.033112	0.051583
496	0.001012	0.001819	0.006473	0.004652	0.005639	0.012145	0.012776	0.016054	0.025669	0.033239	0.051648
495	0.001039	0.001908	0.006697	0.004689	0.005665	0.012196	0.012985	0.016161	0.025784	0.033496	0.051832
494	0.001092	0.001897	0.006601	0.004642	0.005812	0.012402	0.01287	0.016245	0.025801	0.033377	0.051902
493	0.00091	0.001878	0.00665	0.004746	0.005768	0.012354	0.012846	0.016249	0.025847	0.033597	0.051984
492	0.001007	0.001949	0.006663	0.004719	0.005842	0.012296	0.012925	0.016277	0.025979	0.033723	0.05194
491	0.000876	0.001594	0.006624	0.004686	0.005772	0.01234	0.012901	0.016247	0.02584	0.033645	0.052106
490	0.000957	0.001879	0.006772	0.004752	0.005834	0.01243	0.012998	0.016336	0.026004	0.03378	0.052213
489	0.000853	0.001761	0.006654	0.004783	0.005764	0.012451	0.013102	0.016405	0.025944	0.033844	0.052275
488	0.00093	0.001844	0.006802	0.004697	0.005921	0.012461	0.01315	0.016445	0.026117	0.033798	0.052313
487	0.001086	0.001936	0.006884	0.004846	0.005979	0.012728	0.013153	0.016573	0.026376	0.034032	0.052518
486	0.000877	0.001943	0.006849	0.004666	0.005903	0.01259	0.01317	0.01646	0.02619	0.033868	0.05245
485	0.000783	0.001677	0.006696	0.004685	0.005903	0.012619	0.013169	0.016571	0.026165	0.033957	0.052427
484	0.000915	0.00182	0.006808	0.004806	0.005832	0.012692	0.013198	0.016601	0.026314	0.034088	0.052587
483	0.000881	0.001981	0.006896	0.004925	0.006039	0.01276	0.013364	0.016529	0.026521	0.034222	0.052661
482	0.000926	0.001773	0.006794	0.004791	0.005807	0.012721	0.013195	0.01668	0.026326	0.034072	0.052507
481	0.001028	0.001943	0.006905	0.004929	0.006039	0.012749	0.013395	0.016658	0.026634	0.034193	0.052757
480	0.000737	0.001801	0.006844	0.004809	0.005892	0.012622	0.013188	0.016548	0.026503	0.034106	0.052569
479	0.001082	0.002174	0.007042	0.005119	0.006166	0.012949	0.013384	0.016672	0.026598	0.034166	0.052678
478	0.000933	0.001997	0.007018	0.004961	0.005953	0.012907	0.013398	0.016686	0.026594	0.034247	0.052731
477	0.000983	0.001845	0.006909	0.004752	0.005975	0.012733	0.013265	0.016506	0.026408	0.034061	0.052409
476	0.000879	0.001858	0.007092	0.005027	0.006124	0.012905	0.013179	0.016701	0.026624	0.034083	0.052532
475	0.001053	0.002113	0.006964	0.004975	0.006133	0.012966	0.01346	0.016615	0.026575	0.034111	0.052658
474	0.000947	0.001967	0.00707	0.004951	0.006018	0.012764	0.013219	0.016601	0.026541	0.033929	0.052443

EXCLI Journal 2020;19:48-70 – ISSN 1611-2156
Received: October 03, 2019, accepted: December 19, 2019, published: January 03, 2020

	Absorption intensity										
**Wavelength (nm)**	**concentration of β-hydroxyisovalerylshikonin (M)**										
	A	*B*	*C*	*D*	*E*	*F*	*G*	*I*	*J*	*K*	*L*
	0.00	$8.0x10^{-7}$	$1.6x10^{-6}$	$2.0x10^{-6}$	$2.8x10^{-6}$	$4.0x10^{-6}$	$4.8x10^{-6}$	$6.0x10^{-6}$	$8.0x10^{-6}$	$1.2x10^{-5}$	$1.6x10^{-5}$
473	0.000967	0.001858	0.006998	0.004814	0.006027	0.012891	0.013159	0.016674	0.02651	0.033958	0.052406
472	0.001049	0.001836	0.007078	0.004902	0.006102	0.012895	0.013389	0.016661	0.026506	0.033968	0.05245
471	0.000946	0.002045	0.007124	0.005035	0.00616	0.012985	0.013358	0.016575	0.026636	0.033983	0.052212
470	0.00099	0.001947	0.007234	0.005101	0.006151	0.012899	0.013299	0.016559	0.026557	0.033881	0.052242
469	0.000693	0.001981	0.006951	0.004848	0.005983	0.012799	0.013178	0.016593	0.026589	0.033716	0.052214
468	0.001135	0.001885	0.007278	0.004956	0.006154	0.013048	0.013406	0.016592	0.026535	0.03397	0.052206
467	0.001069	0.002017	0.007203	0.00499	0.006209	0.012994	0.013349	0.016545	0.026623	0.033775	0.051748
466	0.000911	0.001989	0.007147	0.004966	0.005934	0.012967	0.013329	0.01653	0.026456	0.033706	0.051563
465	0.001012	0.002183	0.007299	0.004937	0.006073	0.012962	0.013232	0.016578	0.02644	0.033564	0.051543
464	0.000794	0.001818	0.007125	0.004887	0.005867	0.012757	0.013107	0.016274	0.026297	0.033175	0.051456
463	0.001011	0.00181	0.007232	0.00492	0.006104	0.012814	0.013071	0.016402	0.026153	0.033003	0.051396
462	0.001012	0.002062	0.007197	0.00486	0.005841	0.01287	0.013046	0.01643	0.026018	0.033133	0.051308
461	0.001122	0.001824	0.007257	0.005036	0.005976	0.012977	0.013097	0.016165	0.026011	0.033062	0.051115
460	0.000964	0.002114	0.007257	0.004887	0.006037	0.012549	0.012848	0.015996	0.025855	0.032845	0.050923
459	0.001171	0.001953	0.006998	0.004932	0.005822	0.012637	0.012758	0.015921	0.025835	0.032804	0.050901
458	0.000928	0.001727	0.006961	0.004711	0.005654	0.012441	0.012638	0.01577	0.025875	0.03279	0.050735
457	0.00107	0.001872	0.007284	0.004934	0.005913	0.012859	0.012928	0.016136	0.026175	0.032876	0.050726
456	0.001204	0.002143	0.007411	0.005007	0.005871	0.012966	0.013011	0.016076	0.026049	0.032895	0.050877
455	0.000959	0.001926	0.007087	0.004741	0.005854	0.012761	0.012895	0.016071	0.026078	0.032796	0.050636
454	0.001005	0.001812	0.007255	0.004997	0.006083	0.01283	0.012719	0.016003	0.026004	0.032599	0.050519
453	0.001072	0.001988	0.007261	0.004768	0.005911	0.012933	0.012923	0.015902	0.026008	0.032529	0.050444
452	0.000824	0.001733	0.00718	0.004715	0.005753	0.01269	0.012554	0.015724	0.025695	0.032288	0.050101
451	0.000817	0.00183	0.007292	0.00495	0.006021	0.012643	0.012708	0.015837	0.025652	0.032201	0.050057
450	0.001071	0.001966	0.007478	0.004763	0.005845	0.013068	0.012778	0.015734	0.025839	0.032371	0.049941
449	0.000979	0.001831	0.007238	0.004952	0.00573	0.01284	0.012641	0.015787	0.02576	0.032107	0.049658
448	0.00103	0.001705	0.007297	0.00471	0.005745	0.012633	0.012394	0.01562	0.02552	0.031868	0.049551
447	0.001281	0.001892	0.007465	0.00473	0.005826	0.012973	0.012537	0.015569	0.025524	0.031847	0.049473
446	0.000967	0.001821	0.00729	0.004798	0.005714	0.012678	0.012539	0.015514	0.025408	0.031748	0.049188
445	0.001177	0.002	0.007364	0.004892	0.005749	0.012838	0.012451	0.015506	0.025517	0.03155	0.049233
444	0.00121	0.002157	0.007512	0.004899	0.005795	0.012703	0.012579	0.015555	0.025548	0.031523	0.048994
443	0.001079	0.001839	0.007488	0.004929	0.005736	0.012732	0.012326	0.015322	0.025409	0.031359	0.048829
442	0.000982	0.00174	0.007349	0.004736	0.005511	0.01262	0.012177	0.015296	0.025208	0.031147	0.048476
441	0.001164	0.001999	0.007273	0.004679	0.005599	0.012745	0.01226	0.015275	0.025155	0.031043	0.048432

EXCLI Journal 2020;19:48-70 – ISSN 1611-2156
Received: October 03, 2019, accepted: December 19, 2019, published: January 03, 2020

Absorption intensity											
**Wavelength (nm)**	**concentration of β-hydroxyisovalerylshikonin (M)**										
	A	*B*	*C*	*D*	*E*	*F*	*G*	*I*	*J*	*K*	*L*
	0.00	$8.0x10^{-7}$	$1.6x10^{-6}$	$2.0x10^{-6}$	$2.8x10^{-6}$	$4.0x10^{-6}$	$4.8x10^{-6}$	$6.0x10^{-6}$	$8.0x10^{-6}$	$1.2x10^{-5}$	$1.6x10^{-5}$
440	0.001128	0.001996	0.007492	0.004926	0.005836	0.012706	0.012282	0.015243	0.025471	0.031106	0.048466
439	0.000924	0.001704	0.007326	0.004597	0.005466	0.01249	0.012036	0.01512	0.024923	0.030607	0.048006
438	0.001095	0.001648	0.007314	0.004466	0.005441	0.012578	0.011952	0.014909	0.02488	0.030597	0.047921
437	0.000876	0.001757	0.007315	0.004775	0.005401	0.012479	0.012217	0.014806	0.024967	0.030526	0.047741
436	0.001125	0.001922	0.007516	0.004786	0.005653	0.012596	0.01207	0.014973	0.024916	0.030356	0.047584
435	0.000842	0.001696	0.007304	0.004555	0.005308	0.012408	0.011798	0.014691	0.024631	0.030005	0.047488
434	0.000888	0.001784	0.007256	0.004676	0.005376	0.012543	0.012029	0.0146	0.024762	0.030211	0.047312
433	0.000984	0.00175	0.00742	0.004708	0.005414	0.012456	0.011847	0.014598	0.02456	0.030051	0.046864
432	0.00091	0.001784	0.007276	0.004523	0.005435	0.012539	0.011845	0.014688	0.024679	0.029951	0.046903
431	0.001047	0.001685	0.007335	0.004549	0.005295	0.012385	0.011763	0.0144	0.024536	0.029775	0.046818
430	0.001132	0.001904	0.007506	0.004731	0.005327	0.012377	0.011812	0.014631	0.02447	0.029526	0.046791
429	0.001025	0.001746	0.007351	0.004426	0.005412	0.012404	0.011654	0.014376	0.024422	0.02941	0.046554
428	0.001267	0.001903	0.007436	0.004675	0.005353	0.012495	0.011876	0.014513	0.024482	0.029492	0.046389
427	0.001033	0.001808	0.007228	0.004415	0.005079	0.012114	0.011375	0.014159	0.024349	0.029178	0.046315
426	0.000751	0.001726	0.007249	0.004455	0.005191	0.012335	0.011509	0.014244	0.024192	0.029004	0.046238
425	0.00117	0.001774	0.007371	0.004565	0.005277	0.012389	0.011634	0.014267	0.024283	0.029295	0.046088
424	0.000892	0.001697	0.007408	0.004467	0.005088	0.012219	0.011436	0.014222	0.023985	0.029013	0.045854
423	0.001081	0.001995	0.007588	0.004694	0.005258	0.012613	0.011566	0.014441	0.024322	0.029042	0.045938
422	0.001131	0.001844	0.007533	0.004301	0.00518	0.012476	0.011389	0.014154	0.024065	0.028881	0.045601
421	0.001059	0.001921	0.00745	0.004741	0.005074	0.012427	0.011548	0.014274	0.02418	0.028806	0.04554
420	0.001014	0.001808	0.00755	0.00434	0.005043	0.012571	0.011372	0.01402	0.024196	0.028438	0.045641
419	0.001102	0.001738	0.007422	0.004362	0.004943	0.012545	0.011423	0.01407	0.023903	0.028504	0.045276
418	0.001026	0.001681	0.0076	0.004716	0.005228	0.012036	0.01111	0.013705	0.023712	0.028167	0.045165
417	0.001064	0.001503	0.007301	0.003964	0.004603	0.012046	0.010943	0.013509	0.023533	0.028144	0.044895
416	0.001157	0.001708	0.007502	0.00422	0.004943	0.012447	0.011313	0.013986	0.024155	0.028544	0.045276
415	0.000949	0.001695	0.007566	0.004402	0.004958	0.012213	0.011173	0.013702	0.023969	0.028435	0.0451
414	0.00106	0.001838	0.007684	0.00447	0.005104	0.012333	0.011396	0.013938	0.023986	0.028537	0.04516
413	0.001218	0.001701	0.007726	0.004389	0.005095	0.012534	0.01122	0.013858	0.023991	0.028431	0.045229
412	0.001277	0.001903	0.007588	0.00439	0.005266	0.012426	0.011194	0.01404	0.024073	0.028426	0.045241
411	0.001044	0.00143	0.007479	0.004354	0.004995	0.012372	0.011111	0.013884	0.023752	0.02825	0.045103
410	0.000982	0.001799	0.00744	0.004267	0.004772	0.012284	0.010986	0.013711	0.023828	0.028095	0.044863
409	0.000946	0.001688	0.007538	0.004201	0.004782	0.01222	0.011094	0.013821	0.024024	0.028102	0.045226
408	0.001111	0.001747	0.007548	0.004329	0.005176	0.01253	0.011087	0.013885	0.024052	0.028157	0.04531

Absorption intensity											
**Wavelength (nm)**	**concentration of β-hydroxyisovalerylshikonin (M)**										
	A	*B*	*C*	*D*	*E*	*F*	*G*	*I*	*J*	*K*	*L*
	0.00	$8.0x10^{-7}$	$1.6x10^{-6}$	$2.0x10^{-6}$	$2.8x10^{-6}$	$4.0x10^{-6}$	$4.8x10^{-6}$	$6.0x10^{-6}$	$8.0x10^{-6}$	$1.2x10^{-5}$	$1.6x10^{-5}$
407	0.001019	0.001844	0.007675	0.004496	0.005005	0.012603	0.011154	0.013939	0.024208	0.028414	0.045261
406	0.001085	0.001979	0.007905	0.004454	0.005089	0.012821	0.011334	0.013833	0.024334	0.028275	0.045476
405	0.001178	0.001895	0.007841	0.004382	0.005029	0.012822	0.011277	0.013894	0.024208	0.028337	0.045061
404	0.000951	0.001576	0.007493	0.004334	0.004885	0.012761	0.011196	0.013782	0.024226	0.028219	0.045139
403	0.001167	0.00168	0.007572	0.00464	0.004807	0.01265	0.011258	0.013943	0.023882	0.02811	0.045365
402	0.001172	0.001919	0.007864	0.004454	0.005205	0.012499	0.011041	0.013714	0.024107	0.028476	0.045344
401	0.000863	0.001722	0.007867	0.004323	0.005203	0.013111	0.011281	0.013939	0.024392	0.02841	0.045888
400	0.001396	0.002044	0.008091	0.004641	0.005199	0.013051	0.011647	0.014109	0.024638	0.028661	0.046076
399	0.001184	0.001803	0.007989	0.00456	0.005304	0.013003	0.011538	0.014282	0.024703	0.028599	0.046431
398	0.001027	0.001687	0.007833	0.004377	0.005069	0.013009	0.011371	0.01418	0.024647	0.028645	0.046374
397	0.000991	0.001903	0.008074	0.00457	0.005199	0.013157	0.011612	0.014244	0.024896	0.028869	0.046651
396	0.001213	0.001701	0.008241	0.004692	0.005236	0.013205	0.011687	0.014436	0.024948	0.028971	0.046984
395	0.000957	0.001656	0.007876	0.00438	0.00497	0.01314	0.011584	0.014444	0.024899	0.028976	0.047008
394	0.001495	0.001968	0.008394	0.004707	0.005115	0.013458	0.011638	0.014708	0.0253	0.029371	0.04738
393	0.001032	0.001652	0.008132	0.00447	0.005106	0.013266	0.011651	0.014455	0.025318	0.029173	0.047491
392	0.001123	0.001921	0.008356	0.004534	0.005285	0.013443	0.011726	0.01462	0.025661	0.029547	0.047997
391	0.001277	0.001698	0.008199	0.004639	0.005155	0.013335	0.011989	0.014652	0.025685	0.029623	0.047949
390	0.001446	0.002075	0.008872	0.004973	0.005604	0.013967	0.01224	0.015153	0.025959	0.030391	0.048992
389	0.00119	0.002157	0.008579	0.005072	0.005544	0.014171	0.012196	0.015378	0.026695	0.030364	0.048428
388	0.001055	0.002037	0.008791	0.005025	0.005533	0.013431	0.011663	0.014612	0.025772	0.03019	0.048536
387	0.000988	0.001921	0.008335	0.004632	0.005382	0.013834	0.012067	0.01502	0.026307	0.030424	0.049017
386	0.001148	0.001927	0.008552	0.004952	0.005831	0.014094	0.012175	0.015408	0.026546	0.030853	0.049683
385	0.001248	0.002154	0.008977	0.005077	0.005746	0.014246	0.012069	0.015693	0.026666	0.031312	0.050025
384	0.001206	0.002152	0.008897	0.004935	0.005751	0.014318	0.012712	0.015496	0.026671	0.031331	0.05002
383	0.001488	0.002397	0.009063	0.005024	0.006059	0.014603	0.012791	0.015762	0.026937	0.031648	0.050677
382	0.001197	0.001893	0.008481	0.004879	0.005603	0.014489	0.012217	0.015308	0.026944	0.031591	0.051096
381	0.000924	0.001877	0.008869	0.004818	0.005808	0.014479	0.012764	0.015595	0.027389	0.032671	0.051487
380	0.001203	0.002225	0.009359	0.005435	0.005769	0.015078	0.01309	0.016335	0.027426	0.032324	0.051883
379	0.001438	0.002569	0.009434	0.005417	0.006194	0.015182	0.013444	0.016467	0.028164	0.032941	0.052582
378	0.001372	0.002301	0.009432	0.005453	0.006162	0.015352	0.013246	0.01638	0.028308	0.032966	0.052587
377	0.001371	0.0024	0.009325	0.005448	0.006432	0.015309	0.013667	0.016639	0.02817	0.28743	0.053049
376	0.001375	0.002407	0.009842	0.005545	0.006259	0.015543	0.013578	0.016628	0.028701	0.033084	0.054132
375	0.001433	0.002725	0.009639	0.005543	0.006283	0.015803	0.014155	0.017101	0.029354	0.03285	0.053493

EXCLI Journal 2020;19:48-70 – ISSN 1611-2156
Received: October 03, 2019, accepted: December 19, 2019, published: January 03, 2020

Wavelength (nm)	***Absorption intensity***										
	**concentration of β-hydroxyisovalerylshikonin (M)**										
	A	*B*	*C*	*D*	*E*	*F*	*G*	*I*	*J*	*K*	*L*
	0.00	8.0x10^{-7}	1.6x10^{-6}	2.0x10^{-6}	2.8x10^{-6}	4.0x10^{-6}	4.8x10^{-6}	6.0x10^{-6}	8.0x10^{-6}	1.2x10^{-5}	1.6x10^{-5}
374	0.00155	0.00227	0.009765	0.005545	0.006398	0.01554	0.013569	0.017013	0.028639	0.033913	0.054098
373	0.001896	0.00286	0.009798	0.006225	0.007004	0.015919	0.013985	0.016924	0.029722	0.034434	0.054879
372	0.001414	0.002528	0.009385	0.005719	0.006399	0.015885	0.013877	0.017385	0.028936	0.034071	0.05474
371	0.001721	0.002698	0.009813	0.005703	0.006358	0.015916	0.014121	0.01755	0.029413	0.034807	0.055174
370	0.001109	0.002554	0.009873	0.006088	0.006483	0.015718	0.013775	0.017559	0.029689	0.034112	0.055707
369	0.002251	0.00316	0.01058	0.006311	0.006951	0.016925	0.014674	0.017618	0.030204	0.034756	0.056545
368	0.002193	0.003248	0.010632	0.006172	0.0071	0.016678	0.014668	0.018077	0.030468	0.035368	0.056976
367	0.001654	0.003404	0.010394	0.006147	0.006715	0.016892	0.014785	0.017906	0.030013	0.034658	0.056493
366	0.001548	0.003164	0.010541	0.006313	0.006547	0.016979	0.014416	0.017916	0.030303	0.035422	0.057394
365	0.0016	0.00289	0.010636	0.006117	0.006702	0.016954	0.01492	0.018184	0.031031	0.035749	0.058069
364	0.000157	0.00143	0.008897	0.004763	0.005296	0.015052	0.013245	0.016676	0.029601	0.034463	0.056945
363	0.000899	0.00274	0.010517	0.005696	0.006449	0.016795	0.014319	0.018311	0.030815	0.03589	0.058288
362	0.003879	0.004635	0.012856	0.00788	0.008676	0.019345	0.016785	0.020553	0.03156	0.036319	0.059287
361	0.001875	0.003081	0.011418	0.006157	0.007328	0.017424	0.015021	0.01899	0.031737	0.03737	0.059878
360	0.002289	0.003999	0.010948	0.006874	0.007158	0.018251	0.015314	0.019403	0.034162	0.039326	0.06231
359	-0.00015	0.00186	0.00939	0.004862	0.005874	0.016334	0.013837	0.017553	0.029907	0.035466	0.058368
358	0.002102	0.003738	0.012051	0.006738	0.007363	0.018557	0.015904	0.019117	0.031744	0.038884	0.061137
357	0.002934	0.004685	0.012654	0.008038	0.008605	0.01939	0.016642	0.021207	0.031716	0.037247	0.060347
356	0.002287	0.003527	0.011457	0.007088	0.007242	0.018842	0.015363	0.019889	0.032314	0.03803	0.063389
355	0.002008	0.00356	0.010265	0.005746	0.00755	0.01752	0.014724	0.018909	0.034735	0.040502	0.062118
354	-0.00113	0.00081	0.008306	0.003794	0.004705	0.016073	0.011938	0.016458	0.029967	0.035659	0.05946
353	0.003944	0.004609	0.012515	0.007285	0.009363	0.019306	0.018061	0.020035	0.033837	0.039182	0.065589
352	0.005262	0.00766	0.014928	0.010004	0.011737	0.022155	0.019187	0.023285	0.034805	0.039223	0.065653
351	0.000268	0.003489	0.012516	0.007622	0.008316	0.017424	0.014897	0.019787	0.035777	0.041331	0.068603
350	-0.00131	0.000536	0.008212	0.002058	0.003132	0.015151	0.012765	0.015952	0.030816	0.034574	0.060882
349	0.00173	0.0026	0.012559	0.007117	0.007448	0.021806	0.016794	0.02138	0.033254	0.038233	0.064557
348	0.0025	0.003795	0.013747	0.008018	0.008638	0.020873	0.018072	0.021362	0.035986	0.041082	0.068596
347	0.002601	0.003833	0.013718	0.008488	0.008645	0.020858	0.016934	0.021018	0.036495	0.041093	0.069226
346	0.003034	0.004051	0.014706	0.008505	0.008743	0.021244	0.017534	0.021729	0.036827	0.041602	0.070028
345	0.002696	0.004213	0.013635	0.007595	0.007952	0.020569	0.016925	0.021151	0.036964	0.041574	0.069809
344	0.003001	0.004507	0.01389	0.007656	0.008642	0.021094	0.018393	0.022006	0.037183	0.042472	0.071217
343	0.002879	0.004823	0.014743	0.00826	0.008986	0.02147	0.018026	0.022572	0.037644	0.043465	0.072078
342	0.003009	0.004527	0.014756	0.008286	0.008967	0.021428	0.018474	0.022422	0.037755	0.04303	0.072645

EXCLI Journal 2020;19:48-70 – ISSN 1611-2156
Received: October 03, 2019, accepted: December 19, 2019, published: January 03, 2020

Absorption intensity											
**Wavelength (nm)**	**concentration of β-hydroxyisovalerylshikonin (M)**										
	A	*B*	*C*	*D*	*E*	*F*	*G*	*I*	*J*	*K*	*L*
	0.00	$8.0x10^{-7}$	$1.6x10^{-6}$	$2.0x10^{-6}$	$2.8x10^{-6}$	$4.0x10^{-6}$	$4.8x10^{-6}$	$6.0x10^{-6}$	$8.0x10^{-6}$	$1.2x10^{-5}$	$1.6x10^{-5}$
341	0.003193	0.004248	0.014511	0.008419	0.009028	0.02194	0.01876	0.022885	0.038927	0.043634	0.073518
340	0.003517	0.00435	0.014606	0.008452	0.009518	0.022268	0.018956	0.022632	0.03874	0.044335	0.074565
339	0.003176	0.005071	0.015339	0.009415	0.009756	0.023057	0.019352	0.023511	0.039904	0.044817	0.075873
338	0.003284	0.004742	0.014689	0.008804	0.009337	0.022511	0.018928	0.023586	0.039443	0.044863	0.076803
337	0.004031	0.005094	0.015013	0.008571	0.009543	0.023076	0.019764	0.023647	0.040068	0.045381	0.077531
336	0.003445	0.004834	0.01602	0.008941	0.009896	0.022985	0.019172	0.023258	0.040353	0.045431	0.07775
335	0.00354	0.004759	0.015579	0.009326	0.010027	0.023582	0.019602	0.023961	0.04115	0.046376	0.079485
334	0.003566	0.005168	0.015949	0.009349	0.010014	0.024437	0.020114	0.024444	0.041662	0.047014	0.080406
333	0.003751	0.004721	0.015944	0.009735	0.009953	0.024285	0.020755	0.024836	0.042032	0.048391	0.081567
332	0.003273	0.004801	0.015934	0.008605	0.010359	0.023603	0.020831	0.02444	0.042591	0.04849	0.081913
331	0.004167	0.005777	0.01618	0.009814	0.010696	0.024908	0.021296	0.025287	0.043389	0.0494	0.0839
330	0.004245	0.005528	0.017013	0.010439	0.011039	0.025363	0.021486	0.025849	0.043853	0.049932	0.084838
329	0.004196	0.005492	0.01645	0.01039	0.010325	0.025606	0.022308	0.026082	0.044428	0.050826	0.086161
328	0.00474	0.005737	0.016953	0.010631	0.011023	0.026078	0.022459	0.026742	0.045008	0.051401	0.087018
327	0.004406	0.006002	0.017213	0.010366	0.011525	0.026186	0.022616	0.027117	0.045839	0.052069	0.088997
326	0.003905	0.005589	0.016992	0.01043	0.011316	0.026087	0.022433	0.027173	0.046094	0.052553	0.090105
325	0.004533	0.0061	0.017883	0.010822	0.011293	0.026992	0.023141	0.027975	0.046824	0.053843	0.091457
324	0.004494	0.006027	0.01794	0.010872	0.011759	0.027363	0.023431	0.027813	0.047286	0.053991	0.092667
323	0.004722	0.006458	0.018274	0.011341	0.01178	0.027866	0.024201	0.028758	0.048181	0.055305	0.094914
322	0.003953	0.005946	0.01796	0.01106	0.012172	0.02755	0.024327	0.028873	0.048411	0.055676	0.095213
321	0.004977	0.006464	0.018524	0.011669	0.011886	0.028419	0.024725	0.029661	0.049503	0.056863	0.097011
320	0.004752	0.007217	0.019012	0.011434	0.012394	0.029167	0.025039	0.029892	0.049969	0.057857	0.098647
319	0.004543	0.006731	0.019315	0.011763	0.012888	0.029332	0.025724	0.030413	0.050791	0.058862	0.100474
318	0.004547	0.006479	0.019364	0.011999	0.013303	0.029753	0.025537	0.030878	0.05151	0.059566	0.101558
317	0.004988	0.00682	0.019561	0.012904	0.013702	0.030521	0.026844	0.031718	0.052722	0.060383	0.103879
316	0.005085	0.007154	0.020067	0.012816	0.013695	0.030577	0.027191	0.031872	0.053194	0.06187	0.105327
315	0.005296	0.007122	0.0205	0.012582	0.014033	0.030829	0.027734	0.032384	0.054067	0.062514	0.107113
314	0.005279	0.007433	0.020625	0.013126	0.014229	0.031477	0.027815	0.033583	0.054863	0.06415	0.10904
313	0.005696	0.007853	0.020793	0.013162	0.014648	0.03195	0.028564	0.033838	0.056142	0.064345	0.110757
312	0.00549	0.007245	0.021045	0.013155	0.014227	0.032347	0.028631	0.033981	0.05609	0.065841	0.112257
311	0.005723	0.007889	0.021315	0.01379	0.01534	0.0329	0.029412	0.035045	0.057531	0.066667	0.114312
310	0.006221	0.008666	0.021887	0.014168	0.015624	0.0341	0.029959	0.035748	0.05893	0.06846	0.116323
309	0.005674	0.008464	0.022182	0.014052	0.015445	0.033794	0.030365	0.036141	0.059282	0.069374	0.118575

EXCLI Journal 2020;19:48-70 – ISSN 1611-2156
Received: October 03, 2019, accepted: December 19, 2019, published: January 03, 2020

Absorption intensity											
**Wavelength (nm)**	**concentration of β-hydroxyisovalerylshikonin (M)**										
	A	*B*	*C*	*D*	*E*	*F*	*G*	*I*	*J*	*K*	*L*
	0.00	$8.0x10^{-7}$	$1.6x10^{-6}$	$2.0x10^{-6}$	$2.8x10^{-6}$	$4.0x10^{-6}$	$4.8x10^{-6}$	$6.0x10^{-6}$	$8.0x10^{-6}$	$1.2x10^{-5}$	$1.6x10^{-5}$
308	0.006096	0.008587	0.022698	0.014879	0.015952	0.034461	0.03117	0.03711	0.060578	0.071002	0.121225
307	0.00625	0.008636	0.023064	0.015063	0.016442	0.035551	0.031714	0.037109	0.061158	0.071398	0.122928
306	0.006046	0.008556	0.022896	0.014788	0.016152	0.035269	0.031749	0.037839	0.062166	0.072486	0.124657
305	0.006477	0.00896	0.023574	0.015628	0.016678	0.036276	0.032846	0.038841	0.063061	0.074131	0.127348
304	0.006653	0.009316	0.023871	0.015959	0.016854	0.037239	0.033216	0.039209	0.063991	0.07543	0.128873
303	0.006661	0.009431	0.02413	0.016038	0.017248	0.037148	0.033373	0.039341	0.065085	0.076112	0.131023
302	0.006699	0.009233	0.024441	0.016148	0.017699	0.037547	0.033699	0.040481	0.065327	0.077548	0.13324
301	0.006929	0.009619	0.024532	0.016261	0.01755	0.038021	0.034251	0.040832	0.066428	0.078361	0.135259
300	0.007319	0.010083	0.025315	0.017181	0.018285	0.038839	0.035183	0.041604	0.06759	0.079535	0.137772
299	0.007526	0.010551	0.026061	0.017353	0.018625	0.038931	0.035581	0.041852	0.068376	0.080956	0.139384
298	0.007137	0.010292	0.025618	0.017077	0.018194	0.03988	0.035735	0.042335	0.068798	0.081375	0.140964
297	0.00759	0.010351	0.026093	0.017273	0.01878	0.039915	0.036142	0.042748	0.069455	0.082147	0.142951
296	0.007743	0.010961	0.02647	0.017609	0.019289	0.040402	0.037197	0.043665	0.070385	0.083336	0.144812
295	0.007586	0.010993	0.026632	0.017936	0.019319	0.041228	0.03726	0.043969	0.071082	0.084612	0.146886
294	0.007874	0.010867	0.026613	0.017823	0.01937	0.041011	0.037423	0.044117	0.072127	0.084984	0.148842
293	0.008266	0.011382	0.027166	0.018397	0.019442	0.041606	0.037917	0.044883	0.072778	0.08639	0.150507
292	0.008906	0.012081	0.02799	0.018907	0.020201	0.042414	0.038545	0.045777	0.073852	0.08764	0.152629
291	0.008594	0.011971	0.027933	0.018679	0.019895	0.04275	0.038829	0.045434	0.074137	0.087631	0.15402
290	0.009157	0.012204	0.028322	0.019041	0.020076	0.043057	0.039199	0.046201	0.075033	0.089037	0.155932
289	0.009662	0.012514	0.02896	0.019803	0.020552	0.043603	0.039951	0.046986	0.075702	0.090315	0.158142
288	0.009372	0.012311	0.028738	0.019455	0.020559	0.043729	0.039755	0.047226	0.076102	0.090658	0.159161
287	0.009271	0.012872	0.029271	0.020029	0.020764	0.044408	0.040356	0.047362	0.076723	0.091532	0.161343
286	0.009994	0.01313	0.029943	0.020375	0.020993	0.044902	0.040868	0.047635	0.077771	0.092355	0.162924
285	0.009845	0.013137	0.029616	0.020432	0.020893	0.044646	0.041087	0.048053	0.078227	0.093106	0.164834
284	0.00992	0.01364	0.029931	0.020847	0.021251	0.045671	0.041442	0.048816	0.078887	0.094152	0.167173
283	0.010177	0.01346	0.030126	0.020935	0.021381	0.045684	0.042013	0.048731	0.079808	0.095076	0.168417
282	0.010269	0.013687	0.030164	0.020956	0.021526	0.045999	0.042327	0.04959	0.079992	0.095877	0.169953
281	0.011297	0.01406	0.030859	0.021659	0.022126	0.046792	0.042995	0.050261	0.081411	0.097019	0.171982
280	0.010738	0.014241	0.031082	0.022068	0.022431	0.046901	0.043319	0.050648	0.081786	0.097815	0.173647
279	0.011118	0.014357	0.031449	0.022566	0.022799	0.047377	0.044181	0.051033	0.082167	0.098626	0.174923
278	0.011245	0.014306	0.031698	0.022335	0.022552	0.047933	0.044481	0.051646	0.083638	0.099805	0.176734
277	0.011371	0.014635	0.032054	0.022599	0.023032	0.048369	0.044913	0.052301	0.083879	0.100852	0.178577
276	0.01125	0.014652	0.032055	0.02298	0.022959	0.04843	0.045245	0.052797	0.084713	0.102121	0.180115

EXCLI Journal 2020;19:48-70 – ISSN 1611-2156
Received: October 03, 2019, accepted: December 19, 2019, published: January 03, 2020

Absorption intensity											
**Wavelength (nm)**	**concentration of β-hydroxyisovalerylshikonin (M)**										
	A	*B*	*C*	*D*	*E*	*F*	*G*	*I*	*J*	*K*	*L*
	0.00	8.0×10^{-7}	1.6×10^{-6}	2.0×10^{-6}	2.8×10^{-6}	4.0×10^{-6}	4.8×10^{-6}	6.0×10^{-6}	8.0×10^{-6}	1.2×10^{-5}	1.6×10^{-5}
275	0.011018	0.014227	0.032361	0.023168	0.023226	0.048936	0.045747	0.053334	0.085658	0.103334	0.182051
274	0.012079	0.014869	0.033198	0.024007	0.023699	0.050196	0.046989	0.054297	0.087205	0.104852	0.184413
273	0.01203	0.015214	0.033442	0.024139	0.024255	0.050186	0.047487	0.054864	0.087882	0.106348	0.186135
272	0.012519	0.015401	0.033801	0.024582	0.024848	0.051092	0.048016	0.055717	0.089228	0.107923	0.188903
271	0.012226	0.015566	0.034057	0.024613	0.025045	0.05169	0.048665	0.056484	0.09043	0.109643	0.191398
270	0.012564	0.01601	0.034635	0.025443	0.025467	0.052339	0.049656	0.057559	0.091849	0.111489	0.194298
269	0.0125	0.015929	0.035358	0.026034	0.026391	0.053484	0.050704	0.058921	0.093475	0.113919	0.197629
268	0.012484	0.015964	0.035399	0.026172	0.026374	0.053865	0.051652	0.059891	0.094961	0.115896	0.20065
267	0.012601	0.016236	0.03576	0.026766	0.026965	0.055109	0.05291	0.061108	0.096809	0.118784	0.204409
266	0.013247	0.016645	0.036878	0.027676	0.028073	0.056306	0.054081	0.062924	0.09904	0.122295	0.208406
265	0.012906	0.016841	0.03729	0.028045	0.028565	0.057448	0.05524	0.064511	0.101334	0.125767	0.212623
264	0.01314	0.017255	0.038042	0.028694	0.029465	0.058522	0.056894	0.066016	0.103528	0.129159	0.216923
263	0.012957	0.017215	0.038965	0.029601	0.03031	0.059814	0.058181	0.067917	0.106025	0.132899	0.221999
262	0.013088	0.017532	0.03986	0.030171	0.031274	0.06163	0.059956	0.069818	0.108934	0.136914	0.227138
261	0.013048	0.01796	0.040325	0.030993	0.032062	0.062724	0.061512	0.071707	0.111426	0.141191	0.232216
260	0.013085	0.018207	0.041402	0.031624	0.033068	0.064545	0.063207	0.07418	0.114599	0.145523	0.238069
259	0.013556	0.018636	0.042344	0.03215	0.03406	0.066314	0.06525	0.076232	0.117337	0.150048	0.244163
258	0.01385	0.019302	0.043311	0.033457	0.035613	0.068198	0.067494	0.079129	0.12101	0.155628	0.251141
257	0.014011	0.019903	0.044567	0.034574	0.036944	0.070154	0.069458	0.081826	0.124672	0.160964	0.258375
256	0.013789	0.02019	0.045976	0.035601	0.038084	0.072483	0.071991	0.084759	0.128466	0.167389	0.26635
255	0.014012	0.021028	0.047089	0.036854	0.039628	0.074828	0.074999	0.08827	0.133433	0.174477	0.276067
254	0.013845	0.021258	0.04848	0.037984	0.041516	0.077656	0.077692	0.091798	0.138657	0.182101	0.286255
253	0.013961	0.022108	0.050234	0.03947	0.043757	0.080941	0.081387	0.096599	0.144799	0.191013	0.298271
252	0.013993	0.022595	0.052192	0.041536	0.045892	0.084336	0.085443	0.101368	0.151393	0.200482	0.311778
251	0.014156	0.023626	0.054316	0.043506	0.048372	0.088298	0.089981	0.107152	0.159121	0.211828	0.326932
250	0.014132	0.024374	0.056327	0.04538	0.051004	0.092956	0.094628	0.113545	0.167334	0.223706	0.343965
249	0.013662	0.025538	0.058836	0.047773	0.054568	0.09776	0.10023	0.120464	0.177054	0.237743	0.363848
248	0.014368	0.027073	0.062043	0.050743	0.058151	0.103521	0.107384	0.129478	0.188852	0.254723	0.387809
247	0.014708	0.029	0.0657	0.054876	0.063497	0.111079	0.115429	0.140019	0.202625	0.2748	0.416517
246	0.01466	0.03061	0.070025	0.058799	0.069094	0.119351	0.125052	0.152404	0.219255	0.298953	0.450827
245	0.015105	0.032856	0.075154	0.06456	0.076294	0.130071	0.137551	0.168043	0.239983	0.329198	0.493759
244	0.0151	0.035541	0.081621	0.071146	0.085114	0.14277	0.152591	0.18751	0.265875	0.367341	0.547272
243	0.01532	0.039025	0.0899	0.07969	0.096384	0.15982	0.172716	0.212804	0.299913	0.416998	0.61689

Absorption intensity											
**Wavelength (nm)**	**concentration of β-hydroxyisovalerylshikonin (M)**										
	A	*B*	*C*	*D*	*E*	*F*	*G*	*I*	*J*	*K*	*L*
	0.00	$8.0x10^{-7}$	$1.6x10^{-6}$	$2.0x10^{-6}$	$2.8x10^{-6}$	$4.0x10^{-6}$	$4.8x10^{-6}$	$6.0x10^{-6}$	$8.0x10^{-6}$	$1.2x10^{-5}$	$1.6x10^{-5}$
242	0.015321	0.043709	0.100129	0.091191	0.111485	0.181944	0.198165	0.246305	0.343882	0.481845	0.707833
241	0.015684	0.049829	0.114736	0.106492	0.131886	0.211346	0.233302	0.29127	0.403242	0.569885	0.828861
240	0.01581	0.058685	0.134234	0.127662	0.159518	0.251305	0.281552	0.352165	0.483573	0.687361	0.990356
239	0.016061	0.069551	0.16074	0.156236	0.197772	0.30657	0.347036	0.435784	0.592681	0.846584	1.206043
238	0.01614	0.085153	0.196939	0.195728	0.250596	0.382141	0.436953	0.549445	0.74028	1.059674	1.490623
237	0.016861	0.107197	0.248138	0.251462	0.324214	0.486827	0.560724	0.705965	0.941527	1.344419	1.864751
236	0.01729	0.137592	0.318154	0.328255	0.425447	0.629041	0.729479	0.916013	1.209428	1.718553	2.348984
235	0.017639	0.179854	0.415199	0.433738	0.564546	0.822752	0.956899	1.197597	1.563829	2.200904	2.946806
234	0.018067	0.237289	0.547031	0.577391	0.751734	1.080168	1.258608	1.565564	2.020718	2.805718	3.621366
233	0.018633	0.316817	0.724841	0.771345	1.002204	1.4188	1.650777	2.040818	2.591745	3.468006	4.118772
232	0.018938	0.424392	0.961084	1.027355	1.330627	1.856414	2.153788	2.630804	3.252779	3.973518	4.361423
231	0.019969	0.568312	1.269774	1.362228	1.75079	2.403178	2.7623	3.282951	3.829034	4.243033	4.533151
230	0.020748	0.758222	1.666581	1.789593	2.283257	3.043615	3.410268	3.810254	4.1241	4.409414	4.763544
229	0.021433	1.004955	2.163646	2.321486	2.904716	3.637045	3.870306	4.106353	4.297093	4.599035	4.830419
228	0.022535	1.320112	2.756387	2.944487	3.518231	3.973351	4.115086	4.253042	4.47435	4.713133	5.055563
227	0.023565	1.717231	3.377483	3.536167	3.890823	4.178853	4.279938	4.449972	4.615171	4.893128	5.159113
226	0.024696	2.201359	3.819615	3.892382	4.10539	4.354267	4.415994	4.651703	4.723915	4.898591	5.112189
225	0.026258	2.771201	4.047601	4.105662	4.240832	4.487152	4.5428	4.694779	4.774991	5.059889	5.245719
224	0.028032	3.350581	4.216122	4.268871	4.407008	4.647069	4.712671	4.818565	4.874268	5.192343	5.119293
223	0.030007	3.767377	4.378639	4.411438	4.528637	4.739012	4.739222	4.838003	4.965506	5.099478	5.16392
222	0.03239	3.998841	4.482084	4.517765	4.614873	4.866856	4.844444	4.942445	5.054905	5.132457	5.424942
221	0.03466	4.182382	4.54544	4.600522	4.763349	4.791388	4.863355	5.0806	4.909718	5.210669	5.307442
220	0.037815	4.247887	4.61994	4.650798	4.720627	4.798925	4.894798	5.010546	4.968365	5.178861	5.040853
219	0.041116	4.396998	4.766715	4.790082	4.825199	4.852115	4.893103	5.036983	4.981117	5.346289	5.329227
218	0.044814	4.496663	4.758957	4.716808	4.65802	4.81712	4.81696	5.004599	4.870914	6.004396	5.079953
217	0.048875	4.637671	4.801101	4.766368	4.75528	4.977045	4.825684	4.977245	5.090375	5.511971	5.454058
216	0.052949	4.6407	4.946157	4.838327	4.789251	4.823487	4.901598	4.88486	5.146591	10	10
215	0.057869	4.660015	4.913536	4.786323	4.849246	4.885276	4.885065	10	5.000674	5.557042	5.326397
214	0.061738	4.594032	4.830816	5.141484	5.160305	4.859412	5.105061	5.055733	6.502908	6.201965	5.326973
213	0.067125	4.707366	5.126378	4.749972	5.01713	4.757959	5.24435	6.147852	5.749532	5.545428	5.169902
212	0.071627	4.660606	4.99549	4.677709	4.780353	5.092233	5.694225	5.439268	5.050897	10	5.216947
211	0.076558	5.587042	4.531139	4.75761	4.845696	5.014547	5.336751	5.258147	5.492011	5.734745	10
210	0.080782	4.886519	5.64454	4.787277	5.389273	10	10	10	10	10	10

EXCLI Journal 2020;19:48-70 – ISSN 1611-2156
Received: October 03, 2019, accepted: December 19, 2019, published: January 03, 2020

Absorption intensity											
**Wavelength (nm)**	**concentration of *β*-hydroxyisovalerylshikonin (M)**										
	A	*B*	*C*	*D*	*E*	*F*	*G*	*I*	*J*	*K*	*L*
	0.00	$8.0x10^{-7}$	$1.6x10^{-6}$	$2.0x10^{-6}$	$2.8x10^{-6}$	$4.0x10^{-6}$	$4.8x10^{-6}$	$6.0x10^{-6}$	$8.0x10^{-6}$	$1.2x10^{-5}$	$1.6x10^{-5}$
209	0.085663	4.453422	5.007297	4.811263	5.948949	5.471482	5.228432	10	6.249764	10	10
208	0.08963	4.534987	4.5839	4.606485	4.884237	4.862939	4.769915	5.088737	4.873263	4.85297	5.486033
207	0.093544	4.615639	4.965024	4.688216	4.831802	4.996098	4.919641	5.265876	4.787718	5.265232	5.410805
206	0.099975	4.933413	4.800812	4.61222	4.454799	4.65479	5.203637	4.920094	4.995023	5.41487	4.812325
205	0.098788	4.709844	5.666008	4.737453	4.819495	5.697502	10	10	10	10	10
204	0.105932	4.338265	4.504426	4.489998	4.605135	4.366878	4.526171	10	4.557048	5.175374	10
203	0.111076	4.217411	4.448154	4.400108	4.371381	4.45353	4.51097	10	4.174517	6.262936	4.682421
202	0.119004	3.77382	4.375599	3.902612	4.026714	4.165159	4.101524	4.255195	4.812798	4.102826	4.428938
201	0.097058	3.621506	3.463068	3.330431	3.411933	3.446056	3.272557	3.623302	3.510684	4.377361	3.961016
200	0.147145	2.968267	2.870187	2.984442	2.82176	2.881893	2.967229	3.084734	2.934941	3.288682	3.275478

EXCLI Journal 2020;19:48-70 – ISSN 1611-2156
Received: October 03, 2019, accepted: December 19, 2019, published: January 03, 2020

Raw tables concerning **Figure 10.** The fluorescence emission spectra of HSA fixed concentration (2.00 x 10^{-6} M), in the absence (blue line) and presence of increasing concentration of α-methylbutyrylshikon (***1***), acetylshikonin (***2***) and *β*-hydroxyisovalerylshikonin (***3***) (from 0 to 1.6 x 10^{-5} M) Insets: plots of F_0/F versus [naphthoquinone].

Table 1: Emission intensity in the wavelength range of 310-460 nm for fluorescence emission spectra of HSA fixed concentration (2.00 x 10^{-6} M), in the absence (*A*) and presence of increasing concentration of α-methylbutyrylshikon (*B-L*)

	Emission intensity										
**Wavelength (nm)**	**concentration of α-methylbutyrylshikon (M)**										
	A	*B*	*C*	*D*	*E*	*F*	*G*	*I*	*J*	*K*	*L*
	0.00	8.0x10^{-7}	1.6x10^{-6}	2.0x10^{-6}	2.8x10^{-6}	4.0x10^{-6}	4.8x10^{-6}	6.0x10^{-6}	8.0x10^{-6}	1.2x10^{-5}	1.6x10^{-5}
310	22.824	23.615	22.3	22.613	21.59	19.979	20.594	20.881	21.64	22.351	26.968
311	22.15	22.968	21.476	21.752	20.806	19.056	19.832	19.771	20.541	20.942	24.765
312	20.934	21.519	19.937	20.055	19.234	17.183	18.211	17.591	17.798	17.377	19.629
313	20.699	21.082	19.391	19.423	18.611	16.184	17.49	16.543	16.239	15.134	16.039
314	21.2	21.45	19.606	19.523	18.725	16.072	17.522	16.28	15.637	13.9	13.906
315	22.306	22.56	20.515	20.39	19.566	16.538	18.265	16.773	15.811	13.651	12.988
316	23.86	24.058	21.876	21.739	20.844	17.455	19.392	17.691	16.483	14.037	12.925
317	25.643	25.943	23.546	23.329	22.388	18.747	20.785	18.958	17.587	14.843	13.527
318	27.29	27.534	25.076	24.794	23.84	19.942	22.109	20.167	18.661	15.762	14.323
319	29.243	29.522	26.855	26.539	25.535	21.381	23.74	21.574	20.028	16.968	15.418
320	31.19	31.535	28.664	28.298	27.395	22.916	25.391	23.141	21.471	18.268	16.633
321	33.103	33.471	30.475	30.098	28.982	24.363	26.92	24.554	22.831	19.445	17.813
322	34.822	35.296	32.225	31.721	30.543	25.659	28.36	25.938	24.125	20.641	18.934
323	36.517	36.933	33.827	33.225	31.99	26.965	29.831	27.186	25.306	21.764	20.063
324	38.005	38.503	35.257	34.578	33.377	28.103	31.048	28.341	26.372	22.694	21.053
325	39.468	39.897	36.553	35.844	34.672	29.219	32.193	29.46	27.377	23.578	21.959
326	40.856	41.368	37.809	37.115	35.793	30.196	33.373	30.434	28.26	24.394	22.77
327	42.102	42.643	38.961	38.284	36.904	31.016	34.263	31.286	29.104	25.09	23.546
328	43.028	43.539	39.886	39.102	37.774	31.631	35.066	31.916	29.643	25.593	24.046
329	44.135	44.705	40.888	40.062	38.596	32.394	35.93	32.646	30.316	26.148	24.613
330	45.228	45.792	41.845	41.001	39.539	32.993	36.686	33.265	30.846	26.55	25.078
331	46.225	46.851	42.744	41.831	40.32	33.523	37.428	33.888	31.339	26.943	25.487
332	47.225	47.859	43.543	42.69	41.022	34.06	38.047	34.33	31.796	27.318	25.83
333	48.136	48.847	44.354	43.331	41.75	34.511	38.666	34.819	32.232	27.564	26.141
334	49.176	49.776	45.191	44.159	42.483	34.922	39.274	35.327	32.541	27.793	26.361

EXCLI Journal 2020;19:48-70 – ISSN 1611-2156
Received: October 03, 2019, accepted: December 19, 2019, published: January 03, 2020

**Emission intensity**											
**Wavelength (nm)**	**concentration of α-methylbutyrylshikon (M)**										
	A	*B*	*C*	*D*	*E*	*F*	*G*	*I*	*J*	*K*	*L*
	0.00	$8.0x10^{-7}$	$1.6x10^{-6}$	$2.0x10^{-6}$	$2.8x10^{-6}$	$4.0x10^{-6}$	$4.8x10^{-6}$	$6.0x10^{-6}$	$8.0x10^{-6}$	$1.2x10^{-5}$	$1.6x10^{-5}$
335	50.077	50.64	46.088	45.023	43.349	35.272	39.934	35.75	32.888	28.02	26.648
336	51.26	51.789	46.894	45.889	43.928	35.699	40.478	36.211	33.182	28.144	26.903
337	52.264	52.686	47.683	46.657	44.574	36.053	41.096	36.64	33.468	28.318	27.089
338	53.081	53.459	48.501	47.254	45.276	36.39	41.572	37.025	33.687	28.412	27.302
339	54.184	54.654	49.229	48.121	46.126	36.832	42.224	37.373	34.065	28.547	27.525
340	55.342	55.81	50.157	48.979	46.88	37.214	42.873	37.833	34.314	28.658	27.695
341	56.337	56.933	51.085	49.874	47.673	37.569	43.509	38.276	34.672	28.74	27.876
342	57.475	57.886	51.935	50.613	48.464	38.031	44.156	38.727	34.847	28.823	28.041
343	58.504	58.981	52.84	51.48	49.125	38.406	44.804	39.158	35.109	28.911	28.127
344	59.653	60.076	53.771	52.355	49.894	38.738	45.378	39.472	35.481	28.904	28.208
345	60.668	61.189	54.636	53.226	50.638	39.071	45.876	39.959	35.696	28.921	28.18
346	61.726	62.173	55.472	53.983	51.288	39.343	46.487	40.154	35.852	28.836	28.21
347	62.857	63.153	56.275	54.742	52.04	39.581	47.045	40.581	36.079	28.78	28.108
348	63.739	64.108	57.057	55.494	52.771	39.777	47.637	40.957	36.23	28.747	28
349	64.434	64.867	57.557	56.023	53.223	39.991	48.092	41.133	36.354	28.722	27.905
350	65.32	65.609	58.127	56.526	53.78	40.241	48.395	41.326	36.451	28.574	27.761
351	66.162	66.405	58.786	57.15	54.246	40.32	48.812	41.589	36.541	28.449	27.524
352	66.875	66.987	59.32	57.621	54.719	40.481	49.196	41.746	36.639	28.211	27.415
353	67.645	67.517	59.701	58.115	55.057	40.498	49.432	41.831	36.634	28.073	27.205
354	68.21	67.993	60.127	58.458	55.463	40.539	49.757	41.937	36.599	27.848	26.941
355	68.586	68.396	60.465	58.88	55.64	40.515	49.856	42.033	36.521	27.59	26.724
356	68.934	68.793	60.731	58.978	55.855	40.451	49.948	41.963	36.426	27.378	26.453
357	69.207	68.987	60.835	59.065	55.963	40.369	49.999	41.924	36.406	27.13	26.157
358	69.254	69.017	60.743	59.207	55.986	40.254	49.978	41.843	36.194	26.886	25.934
359	69.349	69.18	60.802	59.158	55.918	40.102	49.893	41.757	36.04	26.656	25.722
360	69.262	69.032	60.708	59.014	55.876	39.945	49.787	41.652	35.918	26.426	25.423
361	69.128	68.774	60.425	58.901	55.646	39.747	49.508	41.449	35.699	26.269	25.204
362	68.899	68.64	60.266	58.718	55.476	39.5	49.41	41.119	35.508	26.011	24.976
363	68.623	68.084	60.005	58.421	55.15	39.195	49.104	40.986	35.251	25.794	24.784
364	68.29	67.684	59.49	58.025	54.726	38.897	48.743	40.723	35.006	25.56	24.567
365	67.776	67.292	59.161	57.572	54.236	38.515	48.447	40.352	34.787	25.324	24.331
366	67.159	66.628	58.575	56.935	53.75	38.263	48.067	40.036	34.441	25.18	24.174
367	66.47	65.979	58.089	56.398	53.274	37.789	47.516	39.625	34.132	24.936	23.998

EXCLI Journal 2020;19:48-70 – ISSN 1611-2156
Received: October 03, 2019, accepted: December 19, 2019, published: January 03, 2020

Emission intensity											
**Wavelength (nm)**	**concentration of α-methylbutyrylshikon (M)**										
	A	*B*	*C*	*D*	*E*	*F*	*G*	*I*	*J*	*K*	*L*
	0.00	$8.0x10^{-7}$	$1.6x10^{-6}$	$2.0x10^{-6}$	$2.8x10^{-6}$	$4.0x10^{-6}$	$4.8x10^{-6}$	$6.0x10^{-6}$	$8.0x10^{-6}$	$1.2x10^{-5}$	$1.6x10^{-5}$
368	65.633	65.21	57.316	55.689	52.661	37.476	47.081	39.292	33.77	24.692	23.859
369	65.006	64.463	56.682	55.212	52.162	37.108	46.619	38.762	33.456	24.532	23.628
370	64.151	63.603	55.951	54.524	51.545	36.579	46.03	38.33	33.125	24.262	23.451
371	63.184	62.767	55.192	53.648	50.859	36.085	45.313	37.808	32.64	23.937	23.154
372	62.202	61.657	54.228	52.854	50.052	35.53	44.674	37.273	32.111	23.608	22.822
373	61.116	60.479	53.316	51.96	49.063	34.914	44.004	36.656	31.636	23.285	22.511
374	60.059	59.534	52.429	51.037	48.261	34.234	43.17	35.949	31.142	22.842	22.118
375	58.883	58.351	51.311	49.999	47.38	33.668	42.36	35.308	30.522	22.408	21.687
376	57.731	57.238	50.203	48.992	46.527	32.945	41.523	34.684	29.866	21.988	21.23
377	56.507	55.915	49.197	47.874	45.54	32.213	40.626	33.913	29.281	21.503	20.752
378	55.057	54.662	48.156	46.852	44.415	31.531	39.71	33.217	28.629	21.04	20.315
379	53.773	53.345	47.126	45.806	43.386	30.832	38.845	32.376	27.946	20.561	19.858
380	52.606	52.047	45.915	44.715	42.354	30.056	37.996	31.502	27.333	20.081	19.432
381	51.639	51.031	45.089	43.789	41.517	29.509	37.165	30.985	26.84	19.732	19.084
382	50.34	49.817	43.84	42.73	40.506	28.749	36.245	30.233	26.205	19.296	18.716
383	48.973	48.498	42.726	41.642	39.5	28.067	35.372	29.504	25.635	18.896	18.343
384	47.724	47.168	41.623	40.43	38.495	27.376	34.49	28.813	24.999	18.473	17.998
385	46.378	45.892	40.473	39.403	37.401	26.677	33.633	28.055	24.408	18.074	17.665
386	45.088	44.596	39.348	38.405	36.382	25.962	32.645	27.314	23.8	17.778	17.349
387	43.71	43.314	38.157	37.243	35.36	25.267	31.787	26.58	23.18	17.369	17.024
388	42.329	41.95	37.059	36.07	34.264	24.611	30.815	25.913	22.595	16.99	16.645
389	41.106	40.697	35.879	35.011	33.317	23.909	29.926	25.158	21.967	16.608	16.273
390	39.733	39.284	34.759	33.854	32.24	23.239	29.012	24.394	21.333	16.182	15.909
391	38.438	38.021	33.606	32.784	31.277	22.527	28.151	23.697	20.74	15.696	15.487
392	37.122	36.788	32.497	31.674	30.171	21.833	27.205	22.91	20.094	15.228	15.018
393	35.818	35.453	31.31	30.629	29.227	21.05	26.324	22.091	19.385	14.778	14.591
394	34.831	34.444	30.504	29.755	28.429	20.515	25.672	21.514	18.865	14.387	14.206
395	33.502	33.126	29.413	28.645	27.389	19.769	24.638	20.703	18.157	13.845	13.663
396	32.27	31.891	28.293	27.561	26.311	19.052	23.716	19.961	17.527	13.328	13.169
397	30.995	30.702	27.167	26.52	25.393	18.344	22.825	19.2	16.879	12.838	12.664
398	29.823	29.483	26.076	25.53	24.38	17.649	21.958	18.501	16.243	12.362	12.205
399	28.559	28.329	25.053	24.504	23.377	16.953	21.123	17.796	15.641	11.868	11.724
400	27.365	27.138	24.066	23.544	22.504	16.289	20.312	17.068	15.016	11.394	11.252

Emission intensity											
**Wavelength (nm)**	**concentration of α-methylbutyrylshikon (M)**										
	A	*B*	*C*	*D*	*E*	*F*	*G*	*I*	*J*	*K*	*L*
	0.00	$8.0x10^{-7}$	$1.6x10^{-6}$	$2.0x10^{-6}$	$2.8x10^{-6}$	$4.0x10^{-6}$	$4.8x10^{-6}$	$6.0x10^{-6}$	$8.0x10^{-6}$	$1.2x10^{-5}$	$1.6x10^{-5}$
401	26.281	26.029	23.078	22.582	21.564	15.647	19.446	16.396	14.424	10.965	10.844
402	25.177	24.936	22.107	21.582	20.68	15.009	18.656	15.735	13.87	10.581	10.451
403	24.071	23.846	21.145	20.638	19.752	14.407	17.829	15.07	13.287	10.175	10.077
404	23.079	22.826	20.256	19.797	18.935	13.802	17.096	14.466	12.787	9.804	9.744
405	22.085	21.844	19.351	18.935	18.188	13.26	16.434	13.875	12.289	9.461	9.395
406	21.091	20.895	18.518	18.122	17.445	12.749	15.725	13.325	11.789	9.119	9.072
407	20.35	20.141	17.865	17.503	16.817	12.342	15.202	12.907	11.418	8.85	8.841
408	19.42	19.259	17.107	16.752	16.137	11.844	14.618	12.386	10.978	8.566	8.572
409	18.594	18.439	16.379	16.048	15.467	11.388	14.009	11.898	10.578	8.248	8.291
410	17.763	17.603	15.655	15.353	14.765	10.964	13.419	11.412	10.177	7.943	7.992
411	16.947	16.855	14.947	14.694	14.191	10.505	12.85	10.975	9.792	7.679	7.723
412	16.221	16.08	14.342	14.063	13.55	10.115	12.324	10.534	9.42	7.413	7.427
413	15.501	15.386	13.724	13.488	13.003	9.686	11.812	10.098	9.059	7.139	7.177
414	14.815	14.736	13.13	12.913	12.452	9.309	11.325	9.712	8.689	6.87	6.936
415	14.191	14.136	12.59	12.386	11.985	8.969	10.912	9.332	8.372	6.615	6.689
416	13.618	13.543	12.064	11.89	11.503	8.627	10.454	8.988	8.067	6.382	6.453
417	13.071	12.982	11.582	11.416	11.06	8.311	10.066	8.67	7.775	6.154	6.227
418	12.579	12.48	11.13	10.958	10.611	8.027	9.704	8.345	7.484	5.966	6.021
419	12.156	12.085	10.769	10.652	10.316	7.805	9.414	8.097	7.289	5.804	5.859
420	11.688	11.626	10.405	10.24	9.962	7.545	9.08	7.811	7.057	5.619	5.676
421	11.231	11.185	9.994	9.859	9.586	7.267	8.751	7.558	6.819	5.458	5.519
422	10.819	10.812	9.637	9.52	9.248	7.037	8.458	7.305	6.623	5.3	5.361
423	10.421	10.423	9.293	9.174	8.944	6.814	8.168	7.083	6.413	5.152	5.233
424	10.039	10.01	8.962	8.842	8.631	6.611	7.907	6.865	6.235	5.025	5.104
425	9.667	9.662	8.641	8.543	8.338	6.427	7.675	6.668	6.065	4.902	4.982
426	9.319	9.324	8.35	8.27	8.078	6.239	7.427	6.473	5.889	4.816	4.902
427	8.988	8.999	8.068	7.985	7.815	6.086	7.204	6.288	5.751	4.713	4.806
428	8.659	8.691	7.787	7.722	7.578	5.929	6.994	6.115	5.593	4.633	4.718
429	8.351	8.379	7.537	7.45	7.332	5.742	6.78	5.954	5.47	4.537	4.671
430	8.049	8.047	7.248	7.208	7.118	5.615	6.594	5.798	5.329	4.454	4.586
431	7.76	7.767	7.001	6.984	6.876	5.485	6.377	5.649	5.215	4.373	4.519
432	7.462	7.499	6.794	6.749	6.657	5.339	6.208	5.497	5.092	4.317	4.444
433	7.189	7.247	6.555	6.517	6.461	5.2	6.03	5.363	4.972	4.249	4.396

Received: October 03, 2019, accepted: December 19, 2019, published: January 03, 2020

Emission intensity											
**Wavelength (nm)**	**concentration of α-methylbutyrylshikon (M)**										
	A	*B*	*C*	*D*	*E*	*F*	*G*	*I*	*J*	*K*	*L*
	0.00	$8.0x10^{-7}$	$1.6x10^{-6}$	$2.0x10^{-6}$	$2.8x10^{-6}$	$4.0x10^{-6}$	$4.8x10^{-6}$	$6.0x10^{-6}$	$8.0x10^{-6}$	$1.2x10^{-5}$	$1.6x10^{-5}$
434	6.929	6.99	6.327	6.327	6.231	5.076	5.837	5.221	4.857	4.157	4.33
435	6.636	6.711	6.118	6.114	6.057	4.949	5.67	5.077	4.762	4.104	4.275
436	6.439	6.537	5.94	5.942	5.909	4.851	5.552	4.974	4.675	4.052	4.213
437	6.224	6.283	5.759	5.743	5.71	4.73	5.394	4.846	4.583	3.995	4.168
438	5.989	6.052	5.571	5.56	5.54	4.624	5.24	4.736	4.486	3.941	4.109
439	5.744	5.833	5.369	5.359	5.378	4.518	5.087	4.628	4.383	3.877	4.05
440	5.519	5.601	5.182	5.192	5.201	4.397	4.953	4.508	4.289	3.826	3.98
441	5.309	5.409	5.006	5.022	5.026	4.301	4.825	4.403	4.203	3.778	3.941
442	5.09	5.209	4.824	4.835	4.866	4.211	4.668	4.302	4.104	3.717	3.889
443	4.881	5	4.655	4.686	4.712	4.106	4.548	4.198	4.027	3.661	3.86
444	4.695	4.793	4.501	4.533	4.575	4.016	4.412	4.089	3.929	3.63	3.804
445	4.508	4.627	4.344	4.369	4.437	3.921	4.299	4.004	3.856	3.586	3.777
446	4.322	4.445	4.205	4.241	4.286	3.836	4.189	3.909	3.793	3.556	3.732
447	4.148	4.28	4.06	4.104	4.162	3.76	4.069	3.838	3.723	3.521	3.708
448	3.983	4.129	3.92	3.968	4.034	3.683	3.972	3.769	3.662	3.494	3.686
449	3.825	3.978	3.795	3.852	3.918	3.62	3.868	3.689	3.601	3.477	3.67
450	3.69	3.831	3.68	3.714	3.807	3.554	3.78	3.624	3.559	3.449	3.659
451	3.542	3.696	3.558	3.602	3.701	3.489	3.687	3.543	3.491	3.432	3.638
452	3.399	3.564	3.457	3.504	3.621	3.427	3.617	3.494	3.446	3.421	3.622
453	3.285	3.433	3.348	3.38	3.505	3.382	3.528	3.435	3.415	3.404	3.61
454	3.173	3.333	3.27	3.318	3.441	3.341	3.471	3.391	3.389	3.395	3.615
455	3.043	3.217	3.163	3.225	3.344	3.283	3.396	3.335	3.333	3.37	3.615
456	2.938	3.107	3.071	3.141	3.263	3.236	3.339	3.284	3.305	3.372	3.614
457	2.82	3.004	2.987	3.065	3.189	3.201	3.258	3.235	3.254	3.35	3.593
458	2.72	2.9	2.904	2.973	3.109	3.149	3.201	3.185	3.234	3.33	3.588
459	2.615	2.792	2.818	2.903	3.032	3.115	3.134	3.148	3.193	3.327	3.583
460	2.511	2.698	2.728	2.804	2.947	3.06	3.077	3.085	3.14	3.313	3.559

EXCLI Journal 2020;19:48-70 – ISSN 1611-2156
Received: October 03, 2019, accepted: December 19, 2019, published: January 03, 2020

Table 2: Emission intensity in the wavelength range of 310-460 nm for fluorescence emission spectra of HSA fixed concentration (2.00 x 10^{-6} M), in the absence (*A*) and presence of increasing concentration of acetylshikonin (*B-L*)

**Wavelength (nm)**	***Emission intensity***										
	**concentration of acetylshikonin (M)**										
	A	*B*	*C*	*D*	*E*	*F*	*G*	*I*	*J*	*K*	*L*
	0.00	8.0x10^{-7}	1.6x10^{-6}	2.0x10^{-6}	2.8x10^{-6}	4.0x10^{-6}	4.8x10^{-6}	6.0x10^{-6}	8.0x10^{-6}	1.2x10^{-5}	1.6x10^{-5}
310	22.824	22.58	21.258	20.904	20.004	18.956	20.236	19.59	17.702	20.687	21.903
311	22.15	21.945	20.557	20.044	19.239	18.213	19.313	18.501	16.784	19.786	19.928
312	20.934	20.594	19.023	18.393	17.616	16.509	17.05	15.962	14.63	16.614	15.728
313	20.699	20.233	18.404	17.608	16.813	15.739	15.8	14.436	13.382	14.199	12.787
314	21.2	20.569	18.604	17.669	16.777	15.688	15.378	13.791	12.798	12.745	11.04
315	22.306	21.594	19.459	18.436	17.404	16.242	15.718	13.844	12.938	12.045	10.216
316	23.86	23.029	20.707	19.542	18.419	17.168	16.533	14.446	13.516	12.033	10.2
317	25.643	24.737	22.208	20.97	19.726	18.387	17.703	15.407	14.435	12.462	10.622
318	27.29	26.332	23.615	22.208	20.927	19.519	18.789	16.371	15.341	13.053	11.219
319	29.243	28.159	25.314	23.868	22.4	20.913	20.148	17.561	16.448	13.899	12.1
320	31.19	30.11	27.02	25.407	23.985	22.353	21.521	18.834	17.617	14.903	13.015
321	33.103	31.883	28.617	26.939	25.358	23.71	22.831	20.019	18.697	15.861	13.992
322	34.822	33.539	30.096	28.415	26.792	24.992	24.08	21.147	19.73	16.789	14.878
323	36.517	35.172	31.524	29.801	28.024	26.148	25.182	22.216	20.726	17.731	15.771
324	38.005	36.609	32.81	31.048	29.108	27.141	26.199	23.122	21.618	18.526	16.524
325	39.468	38.037	34.027	32.182	30.19	28.124	27.161	23.917	22.429	19.232	17.222
326	40.856	39.24	35.206	33.222	31.136	29.035	28.015	24.662	23.086	19.818	17.866
327	42.102	40.409	36.221	34.137	32.053	29.867	28.735	25.269	23.734	20.422	18.397
328	43.028	41.339	37.005	34.853	32.686	30.451	29.287	25.709	24.127	20.832	18.77
329	44.135	42.362	37.895	35.646	33.36	31.038	29.942	26.096	24.598	21.259	19.197
330	45.228	43.263	38.642	36.394	34.006	31.631	30.448	26.433	24.995	21.585	19.53
331	46.225	44.221	39.461	37.08	34.64	32.023	30.918	26.754	25.291	21.834	19.822
332	47.225	45.105	40.185	37.808	35.22	32.467	31.314	26.925	25.525	22.026	20.034
333	48.136	45.977	40.949	38.393	35.748	32.922	31.682	27.119	25.804	22.197	20.199
334	49.176	46.833	41.64	39.036	36.228	33.336	32.065	27.214	25.992	22.356	20.31
335	50.077	47.757	42.274	39.638	36.746	33.706	32.365	27.3	26.152	22.423	20.443
336	51.26	48.69	42.972	40.238	37.216	34.102	32.737	27.43	26.286	22.505	20.555
337	52.264	49.575	43.667	40.829	37.793	34.499	33.028	27.394	26.415	22.618	20.661
338	53.081	50.335	44.306	41.331	38.226	34.871	33.23	27.326	26.553	22.661	20.73
339	54.184	51.206	45.033	41.993	38.753	35.237	33.609	27.365	26.65	22.688	20.772

EXCLI Journal 2020;19:48-70 – ISSN 1611-2156
Received: October 03, 2019, accepted: December 19, 2019, published: January 03, 2020

Emission intensity											
**Wavelength (nm)**	**concentration of acetylshikonin (M)**										
	A	*B*	*C*	*D*	*E*	*F*	*G*	*I*	*J*	*K*	*L*
	0.00	$8.0x10^{-7}$	$1.6x10^{-6}$	$2.0x10^{-6}$	$2.8x10^{-6}$	$4.0x10^{-6}$	$4.8x10^{-6}$	$6.0x10^{-6}$	$8.0x10^{-6}$	$1.2x10^{-5}$	$1.6x10^{-5}$
340	55.342	52.171	45.78	42.638	39.265	35.691	33.903	27.285	26.84	22.755	20.8
341	56.337	53.227	46.611	43.215	39.719	36.014	34.28	27.22	26.89	22.775	20.864
342	57.475	54.188	47.365	43.906	40.324	36.39	34.601	27.104	27.036	22.868	20.837
343	58.504	55.141	48.201	44.575	40.9	36.762	34.898	27.081	27.134	22.839	20.847
344	59.653	56.022	48.898	45.271	41.344	37.237	35.166	26.893	27.265	22.814	20.801
345	60.668	57.108	49.671	45.9	41.86	37.586	35.441	26.788	27.347	22.82	20.713
346	61.726	57.985	50.38	46.465	42.491	37.936	35.782	26.579	27.393	22.729	20.602
347	62.857	59.003	51.163	47.09	42.856	38.303	35.999	26.345	27.469	22.647	20.455
348	63.739	59.851	51.882	47.589	43.266	38.608	36.262	26.198	27.474	22.516	20.28
349	64.434	60.592	52.244	47.981	43.741	38.846	36.345	26.036	27.503	22.409	20.146
350	65.32	61.344	52.856	48.53	44.138	39.121	36.589	25.839	27.501	22.323	19.923
351	66.162	61.931	53.538	48.893	44.448	39.207	36.704	25.556	27.455	22.137	19.726
352	66.875	62.576	53.987	49.294	44.776	39.45	36.779	25.303	27.454	21.921	19.522
353	67.645	63.177	54.318	49.499	45.025	39.623	36.788	25.068	27.314	21.793	19.254
354	68.21	63.591	54.589	49.841	45.228	39.755	36.865	24.71	27.236	21.614	19.048
355	68.586	64.039	54.967	50.077	45.385	39.767	36.86	24.393	27.139	21.417	18.832
356	68.934	64.334	55.163	50.138	45.449	39.777	36.823	24.117	26.972	21.199	18.64
357	69.207	64.52	55.307	50.28	45.446	39.746	36.761	23.769	26.844	20.974	18.454
358	69.254	64.629	55.411	50.284	45.471	39.735	36.674	23.464	26.678	20.751	18.201
359	69.349	64.7	55.313	50.353	45.375	39.687	36.598	23.174	26.572	20.604	18.026
360	69.262	64.726	55.335	50.16	45.366	39.541	36.385	22.769	26.394	20.413	17.809
361	69.128	64.478	55.182	49.947	45.062	39.303	36.159	22.431	26.16	20.193	17.686
362	68.899	64.309	54.887	49.775	44.912	39.123	35.899	22.088	25.969	20.023	17.522
363	68.623	64.002	54.755	49.494	44.68	38.904	35.705	21.734	25.781	19.894	17.376
364	68.29	63.538	54.391	49.121	44.428	38.551	35.446	21.383	25.52	19.723	17.299
365	67.776	63.164	54.028	48.825	44.114	38.218	35.053	21.011	25.323	19.565	17.219
366	67.159	62.634	53.559	48.469	43.612	37.967	34.764	20.69	25.058	19.424	17.133
367	66.47	62.066	53.061	48.067	43.265	37.58	34.376	20.323	24.866	19.267	17.041
368	65.633	61.392	52.499	47.386	42.811	37.137	33.963	19.956	24.676	19.077	17.019
369	65.006	60.825	52.016	46.917	42.38	36.793	33.656	19.618	24.425	18.946	16.964
370	64.151	60.044	51.354	46.446	41.865	36.236	33.179	19.254	24.099	18.785	16.841
371	63.184	59.195	50.504	45.687	41.23	35.72	32.711	18.856	23.803	18.556	16.726
372	62.202	58.273	49.78	44.964	40.602	35.229	32.19	18.428	23.488	18.262	16.527

EXCLI Journal 2020;19:48-70 – ISSN 1611-2156
Received: October 03, 2019, accepted: December 19, 2019, published: January 03, 2020

Emission intensity											
**Wavelength (nm)**	**concentration of acetylshikonin (M)**										
	A	*B*	*C*	*D*	*E*	*F*	*G*	*I*	*J*	*K*	*L*
	0.00	8.0×10^{-7}	1.6×10^{-6}	2.0×10^{-6}	2.8×10^{-6}	4.0×10^{-6}	4.8×10^{-6}	6.0×10^{-6}	8.0×10^{-6}	1.2×10^{-5}	1.6×10^{-5}
373	61.116	57.225	49.055	44.227	39.983	34.636	31.683	18.015	23.097	18.007	16.332
374	60.059	56.229	48.201	43.326	39.3	34.044	31.07	17.54	22.683	17.721	16.063
375	58.883	55.267	47.281	42.662	38.529	33.38	30.475	17.134	22.264	17.351	15.783
376	57.731	54.153	46.389	41.735	37.823	32.763	29.879	16.725	21.789	17.034	15.528
377	56.507	53.041	45.417	40.903	36.969	32	29.162	16.278	21.329	16.674	15.201
378	55.057	51.888	44.488	39.966	36.141	31.312	28.515	15.857	20.827	16.314	14.88
379	53.773	50.855	43.471	39.066	35.384	30.626	27.787	15.42	20.371	15.96	14.552
380	52.606	49.61	42.392	38.127	34.5	29.859	27.203	14.982	19.95	15.595	14.265
381	51.639	48.646	41.572	37.411	33.817	29.29	26.681	14.692	19.626	15.322	14.056
382	50.34	47.535	40.627	36.479	33.043	28.635	25.935	14.297	19.109	15.014	13.805
383	48.973	46.238	39.667	35.675	32.267	27.949	25.424	13.913	18.699	14.713	13.55
384	47.724	45.109	38.584	34.711	31.464	27.22	24.782	13.556	18.247	14.438	13.404
385	46.378	43.946	37.543	33.757	30.652	26.511	24.138	13.192	17.859	14.153	13.208
386	45.088	42.718	36.513	32.815	29.824	25.788	23.483	12.864	17.475	13.878	13.021
387	43.71	41.486	35.453	31.958	29.031	25.07	22.888	12.49	17.05	13.592	12.822
388	42.329	40.267	34.457	31.014	28.103	24.399	22.207	12.112	16.628	13.325	12.603
389	41.106	38.985	33.491	30.141	27.291	23.755	21.56	11.776	16.198	13.008	12.348
390	39.733	37.809	32.439	29.205	26.477	23.069	20.917	11.392	15.763	12.721	12.119
391	38.438	36.592	31.394	28.202	25.731	22.288	20.256	11.046	15.325	12.389	11.862
392	37.122	35.414	30.417	27.328	24.851	21.577	19.659	10.69	14.84	12.042	11.532
393	35.818	34.196	29.37	26.412	24.042	20.865	19.044	10.342	14.34	11.66	11.195
394	34.831	33.253	28.525	25.659	23.384	20.328	18.489	10.015	13.997	11.363	10.919
395	33.502	32.076	27.503	24.756	22.569	19.605	17.81	9.677	13.508	10.953	10.545
396	32.27	30.838	26.572	23.807	21.728	18.874	17.141	9.293	13.007	10.56	10.122
397	30.995	29.649	25.552	22.919	20.94	18.192	16.515	8.912	12.53	10.181	9.773
398	29.823	28.597	24.57	22.059	20.144	17.454	15.903	8.584	12.043	9.779	9.392
399	28.559	27.452	23.663	21.163	19.368	16.832	15.291	8.272	11.605	9.432	9.035
400	27.365	26.368	22.713	20.352	18.583	16.171	14.684	7.981	11.177	9.061	8.721
401	26.281	25.349	21.797	19.523	17.838	15.5	14.07	7.656	10.749	8.72	8.394
402	25.177	24.241	20.921	18.704	17.112	14.933	13.483	7.378	10.296	8.413	8.108
403	24.071	23.195	20.027	17.957	16.383	14.27	12.916	7.103	9.914	8.114	7.806
404	23.079	22.24	19.199	17.166	15.75	13.707	12.406	6.84	9.532	7.822	7.568
405	22.085	21.322	18.401	16.481	15.123	13.15	11.916	6.59	9.182	7.558	7.328

EXCLI Journal 2020;19:48-70 – ISSN 1611-2156
Received: October 03, 2019, accepted: December 19, 2019, published: January 03, 2020

Wavelength (nm)	***Emission intensity***										
	**concentration of acetylshikonin (M)**										
	A	*B*	*C*	*D*	*E*	*F*	*G*	*I*	*J*	*K*	*L*
	0.00	$8.0x10^{-7}$	$1.6x10^{-6}$	$2.0x10^{-6}$	$2.8x10^{-6}$	$4.0x10^{-6}$	$4.8x10^{-6}$	$6.0x10^{-6}$	$8.0x10^{-6}$	$1.2x10^{-5}$	$1.6x10^{-5}$
406	21.091	20.401	17.613	15.782	14.481	12.593	11.464	6.326	8.848	7.301	7.117
407	20.35	19.722	17.071	15.295	13.99	12.211	11.106	6.158	8.573	7.118	6.961
408	19.42	18.818	16.326	14.647	13.413	11.699	10.651	5.952	8.27	6.888	6.742
409	18.594	18.011	15.653	14.045	12.859	11.256	10.21	5.742	7.993	6.675	6.538
410	17.763	17.268	14.97	13.452	12.359	10.804	9.819	5.527	7.679	6.453	6.349
411	16.947	16.518	14.386	12.89	11.842	10.346	9.404	5.328	7.387	6.208	6.118
412	16.221	15.805	13.774	12.357	11.373	9.94	9.049	5.145	7.119	6.006	5.908
413	15.501	15.113	13.193	11.841	10.906	9.527	8.691	4.959	6.864	5.804	5.717
414	14.815	14.461	12.683	11.39	10.464	9.177	8.35	4.806	6.589	5.617	5.53
415	14.191	13.89	12.173	10.909	10.055	8.818	8.045	4.636	6.361	5.418	5.349
416	13.618	13.322	11.684	10.457	9.67	8.483	7.752	4.517	6.118	5.221	5.159
417	13.071	12.816	11.235	10.079	9.332	8.188	7.453	4.377	5.915	5.055	4.989
418	12.579	12.316	10.814	9.699	8.961	7.878	7.194	4.232	5.729	4.906	4.82
419	12.156	11.939	10.484	9.41	8.699	7.649	7.01	4.147	5.57	4.784	4.69
420	11.688	11.512	10.092	9.071	8.422	7.383	6.754	4.038	5.393	4.65	4.552
421	11.231	11.092	9.734	8.737	8.107	7.14	6.532	3.942	5.224	4.513	4.417
422	10.819	10.678	9.409	8.427	7.837	6.905	6.33	3.857	5.072	4.406	4.307
423	10.421	10.276	9.087	8.138	7.577	6.691	6.138	3.774	4.934	4.293	4.206
424	10.039	9.92	8.743	7.874	7.311	6.492	5.944	3.688	4.807	4.207	4.117
425	9.667	9.595	8.481	7.61	7.102	6.295	5.778	3.624	4.691	4.106	4.034
426	9.319	9.253	8.209	7.387	6.886	6.11	5.629	3.56	4.581	4.039	3.957
427	8.988	8.941	7.924	7.138	6.687	5.933	5.477	3.514	4.478	3.966	3.9
428	8.659	8.633	7.675	6.922	6.499	5.768	5.326	3.459	4.397	3.9	3.861
429	8.351	8.324	7.417	6.713	6.304	5.614	5.2	3.402	4.304	3.858	3.803
430	8.049	8.036	7.205	6.515	6.104	5.454	5.068	3.346	4.211	3.813	3.741
431	7.76	7.756	6.952	6.303	5.927	5.314	4.945	3.316	4.131	3.76	3.709
432	7.462	7.496	6.745	6.095	5.764	5.177	4.808	3.269	4.057	3.7	3.663
433	7.189	7.215	6.534	5.921	5.593	5.04	4.707	3.246	3.987	3.647	3.629
434	6.929	6.968	6.322	5.731	5.394	4.898	4.589	3.202	3.906	3.615	3.576
435	6.636	6.728	6.103	5.566	5.265	4.782	4.468	3.167	3.853	3.559	3.536
436	6.439	6.542	5.951	5.426	5.152	4.683	4.396	3.138	3.791	3.529	3.51
437	6.224	6.3	5.761	5.266	5.001	4.566	4.299	3.114	3.734	3.489	3.472
438	5.989	6.086	5.584	5.114	4.861	4.443	4.198	3.077	3.665	3.443	3.442

EXCLI Journal 2020;19:48-70 – ISSN 1611-2156
Received: October 03, 2019, accepted: December 19, 2019, published: January 03, 2020

Emission intensity											
**Wavelength (nm)**	**concentration of acetylshikonin (M)**										
	A	*B*	*C*	*D*	*E*	*F*	*G*	*I*	*J*	*K*	*L*
	0.00	$8.0x10^{-7}$	$1.6x10^{-6}$	$2.0x10^{-6}$	$2.8x10^{-6}$	$4.0x10^{-6}$	$4.8x10^{-6}$	$6.0x10^{-6}$	$8.0x10^{-6}$	$1.2x10^{-5}$	$1.6x10^{-5}$
439	5.744	5.853	5.399	4.951	4.731	4.343	4.109	3.062	3.614	3.418	3.41
440	5.519	5.662	5.24	4.797	4.595	4.223	4.008	3.039	3.552	3.367	3.368
441	5.309	5.445	5.064	4.673	4.456	4.131	3.931	3.013	3.493	3.333	3.324
442	5.09	5.249	4.895	4.509	4.349	4.034	3.844	2.987	3.435	3.299	3.309
443	4.881	5.047	4.739	4.37	4.222	3.927	3.767	2.962	3.402	3.289	3.279
444	4.695	4.87	4.598	4.247	4.105	3.845	3.695	2.943	3.355	3.258	3.254
445	4.508	4.696	4.434	4.13	3.978	3.769	3.632	2.935	3.306	3.232	3.242
446	4.322	4.523	4.299	4.016	3.888	3.675	3.557	2.93	3.267	3.221	3.22
447	4.148	4.366	4.18	3.898	3.782	3.607	3.495	2.918	3.245	3.194	3.203
448	3.983	4.2	4.052	3.787	3.702	3.521	3.431	2.908	3.201	3.177	3.203
449	3.825	4.056	3.926	3.691	3.608	3.448	3.375	2.901	3.184	3.176	3.197
450	3.69	3.903	3.826	3.587	3.518	3.397	3.318	2.895	3.158	3.162	3.187
451	3.542	3.79	3.717	3.505	3.442	3.328	3.279	2.891	3.133	3.15	3.187
452	3.399	3.656	3.61	3.418	3.366	3.261	3.23	2.883	3.115	3.156	3.208
453	3.285	3.514	3.494	3.33	3.283	3.219	3.183	2.895	3.091	3.164	3.203
454	3.173	3.431	3.427	3.263	3.236	3.168	3.157	2.892	3.083	3.162	3.209
455	3.043	3.309	3.34	3.181	3.173	3.124	3.105	2.878	3.07	3.156	3.203
456	2.938	3.215	3.257	3.103	3.107	3.073	3.078	2.881	3.053	3.16	3.221
457	2.82	3.108	3.172	3.036	3.054	3.031	3.044	2.875	3.028	3.155	3.21
458	2.72	3.014	3.088	2.979	2.983	2.983	3.003	2.879	3.021	3.156	3.228
459	2.615	2.905	3.014	2.907	2.937	2.944	2.97	2.873	2.993	3.151	3.24
460	2.511	2.796	2.934	2.83	2.867	2.905	2.926	2.875	2.976	3.163	3.241

EXCLI Journal 2020;19:48-70 – ISSN 1611-2156
Received: October 03, 2019, accepted: December 19, 2019, published: January 03, 2020

Table 3: Emission intensity in the wavelength range of 310-460 nm for fluorescence emission spectra of HSA fixed concentration (2.00 x 10^{-6} M), in the absence (*A*) and presence of increasing concentration of *β*-hydroxyisovalerylshikonin (*B-L*)

Emission intensity											
**Wavelength (nm)**	**concentration of *β*-hydroxyisovalerylshikonin (M)**										
	A	*B*	*C*	*D*	*E*	*F*	*G*	*I*	*J*	*K*	*L*
	0.00	8.0x10^{-7}	1.6x10^{-6}	2.0x10^{-6}	2.8x10^{-6}	4.0x10^{-6}	4.8x10^{-6}	6.0x10^{-6}	8.0x10^{-6}	1.2x10^{-5}	1.6x10^{-5}
310	22.824	22.586	21.69	21.387	20.373	19.371	19.095	18.477	17.337	15.251	12.907
311	22.15	21.919	20.988	20.6	19.731	18.656	18.287	17.573	16.508	14.309	12.11
312	20.934	20.627	19.636	19.113	18.159	16.893	16.718	15.808	14.716	12.46	10.126
313	20.699	20.199	19.186	18.459	17.445	16.092	15.963	14.83	13.728	11.352	8.922
314	21.2	20.561	19.431	18.577	17.489	15.979	15.887	14.593	13.437	10.894	8.321
315	22.306	21.573	20.341	19.394	18.109	16.513	16.528	14.981	13.76	11.007	8.241
316	23.86	22.993	21.684	20.62	19.183	17.468	17.52	15.753	14.465	11.522	8.631
317	25.643	24.692	23.332	22.116	20.584	18.749	18.802	16.852	15.475	12.284	9.305
318	27.29	26.305	24.685	23.451	21.827	19.901	19.993	17.905	16.459	13.04	9.969
319	29.243	28.178	26.503	25.118	23.364	21.338	21.39	19.204	17.647	13.986	10.908
320	31.19	30.049	28.254	26.835	24.984	22.801	22.887	20.519	18.925	14.955	11.89
321	33.103	31.854	29.957	28.463	26.492	24.197	24.293	21.733	20.08	15.935	12.88
322	34.822	33.554	31.59	29.983	27.9	25.502	25.57	22.945	21.169	16.827	13.858
323	36.517	35.147	33.084	31.373	29.251	26.65	26.825	24.085	22.278	17.687	14.831
324	38.005	36.646	34.461	32.674	30.53	27.708	27.926	25.029	23.183	18.433	15.693
325	39.468	38.004	35.653	33.842	31.574	28.761	29.027	25.917	24.046	19.148	16.476
326	40.856	39.275	36.882	34.979	32.546	29.653	29.93	26.708	24.757	19.71	17.233
327	42.102	40.479	37.988	36.01	33.474	30.504	30.757	27.512	25.423	20.217	17.878
328	43.028	41.379	38.83	36.833	34.141	31.048	31.373	27.995	25.926	20.574	18.264
329	44.135	42.352	39.795	37.752	34.915	31.726	32.014	28.516	26.417	20.99	18.78
330	45.228	43.463	40.664	38.577	35.71	32.31	32.636	28.987	26.866	21.24	19.302
331	46.225	44.293	41.52	39.322	36.416	32.876	33.192	29.411	27.204	21.423	19.607
332	47.225	45.211	42.399	40.084	37.042	33.327	33.664	29.825	27.512	21.626	19.892
333	48.136	46.149	43.211	40.763	37.561	33.761	34.212	30.124	27.768	21.753	20.104
334	49.176	46.971	44.021	41.393	38.193	34.108	34.61	30.469	27.923	21.846	20.369
335	50.077	47.868	44.677	42.019	38.695	34.53	34.974	30.75	28.14	21.873	20.568
336	51.26	48.782	45.439	42.76	39.283	35.023	35.498	31.095	28.319	21.859	20.749
337	52.264	49.666	46.146	43.383	39.842	35.416	35.962	31.326	28.503	21.977	20.942
338	53.081	50.447	46.859	43.925	40.291	35.811	36.295	31.552	28.602	21.953	21.081
339	54.184	51.389	47.822	44.705	40.857	36.17	36.771	31.76	28.859	21.944	21.238

Emission intensity											
**Wavelength (nm)**	**concentration of β-hydroxyisovalerylshikonin (M)**										
	A	*B*	*C*	*D*	*E*	*F*	*G*	*I*	*J*	*K*	*L*
	0.00	$8.0x10^{-7}$	$1.6x10^{-6}$	$2.0x10^{-6}$	$2.8x10^{-6}$	$4.0x10^{-6}$	$4.8x10^{-6}$	$6.0x10^{-6}$	$8.0x10^{-6}$	$1.2x10^{-5}$	$1.6x10^{-5}$
340	55.342	52.383	48.591	45.329	41.472	36.553	37.098	32.012	28.93	21.965	21.34
341	56.337	53.386	49.51	46.08	42.061	36.999	37.559	32.306	29.092	21.961	21.503
342	57.475	54.172	50.411	46.909	42.765	37.325	38.084	32.546	29.227	21.911	21.619
343	58.504	55.194	51.147	47.723	43.369	37.643	38.587	32.912	29.39	21.933	21.614
344	59.653	56.227	52.016	48.449	43.985	38.272	39.021	33.172	29.537	21.848	21.635
345	60.668	57.224	52.84	49.101	44.551	38.686	39.443	33.346	29.59	21.867	21.578
346	61.726	58.085	53.702	49.791	45.021	39.046	39.816	33.621	29.675	21.829	21.468
347	62.857	58.941	54.336	50.407	45.622	39.415	40.267	33.851	29.795	21.737	21.28
348	63.739	59.917	55.006	51.004	46.172	39.769	40.553	34.024	29.903	21.627	21.143
349	64.434	60.603	55.64	51.557	46.537	39.991	40.822	34.096	29.883	21.558	20.979
350	65.32	61.277	56.239	51.995	46.958	40.238	41.092	34.206	29.842	21.446	20.775
351	66.162	62.016	56.862	52.577	47.292	40.368	41.441	34.393	29.904	21.344	20.608
352	66.875	62.665	57.429	52.97	47.728	40.607	41.567	34.52	29.885	21.192	20.379
353	67.645	63.409	57.847	53.332	48.038	40.704	41.672	34.525	29.836	21.044	20.13
354	68.21	63.761	58.287	53.63	48.216	40.86	41.905	34.506	29.811	20.931	19.848
355	68.586	63.955	58.661	53.841	48.354	40.845	41.932	34.578	29.692	20.773	19.664
356	68.934	64.294	58.772	54.081	48.489	40.926	41.99	34.548	29.495	20.68	19.388
357	69.207	64.41	58.885	54.145	48.603	40.901	41.952	34.355	29.363	20.498	19.174
358	69.254	64.529	58.983	54.062	48.495	40.767	41.946	34.322	29.216	20.324	18.952
359	69.349	64.631	58.954	54.179	48.568	40.692	41.882	34.251	29.049	20.226	18.812
360	69.262	64.705	58.93	54.141	48.496	40.55	41.687	34.105	28.877	20.107	18.66
361	69.128	64.48	58.731	53.992	48.364	40.295	41.549	33.92	28.695	19.912	18.52
362	68.899	64.202	58.564	53.686	48.102	40.12	41.332	33.733	28.529	19.82	18.352
363	68.623	63.979	58.333	53.375	47.881	39.938	40.986	33.497	28.242	19.729	18.288
364	68.29	63.638	57.869	53.042	47.581	39.608	40.774	33.231	28.013	19.537	18.254
365	67.776	63.07	57.443	52.661	47.076	39.264	40.441	32.945	27.829	19.478	18.23
366	67.159	62.618	57.068	52.059	46.771	38.848	40.145	32.728	27.6	19.37	18.232
367	66.47	61.997	56.382	51.652	46.244	38.433	39.705	32.435	27.323	19.272	18.239
368	65.633	61.266	55.707	51.012	45.789	38.07	39.351	31.978	27.088	19.122	18.284
369	65.006	60.659	55.203	50.473	45.316	37.596	38.818	31.716	26.873	18.994	18.288
370	64.151	60.001	54.418	49.912	44.775	37.094	38.371	31.295	26.5	18.887	18.255
371	63.184	59.048	53.545	49.236	44.224	36.547	37.771	30.85	26.122	18.66	18.175
372	62.202	58.116	52.792	48.297	43.5	36.05	37.227	30.425	25.771	18.474	18.033

EXCLI Journal 2020;19:48-70 – ISSN 1611-2156
Received: October 03, 2019, accepted: December 19, 2019, published: January 03, 2020

Emission intensity											
**Wavelength (nm)**	**concentration of β-hydroxyisovalerylshikonin (M)**										
	A	*B*	*C*	*D*	*E*	*F*	*G*	*I*	*J*	*K*	*L*
	0.00	$8.0x10^{-7}$	$1.6x10^{-6}$	$2.0x10^{-6}$	$2.8x10^{-6}$	$4.0x10^{-6}$	$4.8x10^{-6}$	$6.0x10^{-6}$	$8.0x10^{-6}$	$1.2x10^{-5}$	$1.6x10^{-5}$
373	61.116	57.199	51.9	47.534	42.633	35.486	36.643	29.917	25.33	18.196	17.879
374	60.059	56.1	51.103	46.704	41.986	34.83	35.931	29.364	24.851	17.967	17.625
375	58.883	55.058	50.1	45.87	41.181	34.128	35.287	28.899	24.41	17.667	17.312
376	57.731	54.091	49.113	44.842	40.397	33.335	34.565	28.367	23.919	17.366	17.063
377	56.507	52.893	48.076	43.919	39.605	32.677	33.804	27.717	23.384	16.968	16.699
378	55.057	51.722	46.968	42.9	38.671	32	33.106	27.117	22.895	16.613	16.376
379	53.773	50.63	45.801	41.915	37.839	31.232	32.343	26.491	22.397	16.271	16.1
380	52.606	49.451	44.746	40.911	36.895	30.489	31.567	25.914	21.847	15.92	15.826
381	51.639	48.518	43.921	40.051	36.151	29.912	30.996	25.478	21.445	15.65	15.591
382	50.34	47.312	42.846	39.066	35.343	29.187	30.21	24.827	20.988	15.353	15.356
383	48.973	46.117	41.728	38.075	34.459	28.455	29.455	24.22	20.532	15.092	15.167
384	47.724	44.971	40.634	37.063	33.549	27.672	28.678	23.666	20.029	14.833	14.996
385	46.378	43.652	39.501	36.095	32.636	26.976	27.888	23.118	19.562	14.535	14.8
386	45.088	42.472	38.462	35.109	31.785	26.273	27.201	22.511	19.092	14.277	14.701
387	43.71	41.257	37.373	34.129	30.896	25.521	26.437	21.934	18.639	13.955	14.55
388	42.329	40.026	36.26	33.057	30	24.817	25.637	21.354	18.111	13.717	14.375
389	41.106	38.821	35.17	32.067	29.074	24.06	24.939	20.763	17.635	13.394	14.144
390	39.733	37.598	34.016	31.074	28.253	23.351	24.192	20.169	17.172	13.071	13.904
391	38.438	36.291	32.9	30.069	27.395	22.583	23.411	19.588	16.693	12.783	13.57
392	37.122	35.208	31.809	29.031	26.464	21.873	22.697	18.91	16.216	12.439	13.262
393	35.818	33.975	30.77	28.022	25.614	21.145	21.931	18.324	15.68	12.023	12.924
394	34.831	33.105	29.831	27.283	24.936	20.597	21.292	17.847	15.223	11.742	12.587
395	33.502	31.777	28.77	26.265	24.003	19.865	20.521	17.227	14.678	11.313	12.151
396	32.27	30.653	27.691	25.218	23.122	19.1	19.797	16.623	14.118	10.925	11.743
397	30.995	29.494	26.644	24.323	22.248	18.395	19.017	16.014	13.627	10.522	11.312
398	29.823	28.345	25.621	23.395	21.407	17.684	18.303	15.406	13.134	10.143	10.866
399	28.559	27.245	24.629	22.491	20.59	17.007	17.585	14.842	12.655	9.777	10.472
400	27.365	26.171	23.601	21.545	19.765	16.35	16.9	14.224	12.14	9.428	10.091
401	26.281	25.125	22.642	20.669	18.983	15.661	16.202	13.69	11.68	9.075	9.739
402	25.177	24.115	21.701	19.878	18.183	15.042	15.562	13.175	11.198	8.754	9.411
403	24.071	23.051	20.793	19.022	17.449	14.445	14.936	12.657	10.79	8.428	9.12
404	23.079	22.102	19.898	18.171	16.73	13.839	14.325	12.156	10.369	8.138	8.821
405	22.085	21.127	19.043	17.427	16.027	13.276	13.73	11.681	9.946	7.865	8.576

EXCLI Journal 2020;19:48-70 – ISSN 1611-2156
Received: October 03, 2019, accepted: December 19, 2019, published: January 03, 2020

Emission intensity											
**Wavelength (nm)**	**concentration of *β*-hydroxyisovalerylshikonin (M)**										
	A	*B*	*C*	*D*	*E*	*F*	*G*	*I*	*J*	*K*	*L*
	0.00	$8.0x10^{-7}$	$1.6x10^{-6}$	$2.0x10^{-6}$	$2.8x10^{-6}$	$4.0x10^{-6}$	$4.8x10^{-6}$	$6.0x10^{-6}$	$8.0x10^{-6}$	$1.2x10^{-5}$	$1.6x10^{-5}$
406	21.091	20.272	18.262	16.677	15.358	12.731	13.188	11.233	9.604	7.606	8.328
407	20.35	19.556	17.653	16.109	14.847	12.369	12.768	10.9	9.327	7.441	8.128
408	19.42	18.733	16.912	15.416	14.25	11.847	12.237	10.482	8.975	7.174	7.901
409	18.594	17.907	16.168	14.763	13.693	11.367	11.742	10.081	8.661	6.956	7.678
410	17.763	17.137	15.5	14.164	13.122	10.928	11.282	9.691	8.334	6.723	7.48
411	16.947	16.36	14.842	13.558	12.547	10.458	10.823	9.312	8.016	6.496	7.244
412	16.221	15.712	14.19	12.982	12.038	10.038	10.39	8.966	7.72	6.279	7.03
413	15.501	15.021	13.568	12.401	11.543	9.647	9.978	8.637	7.415	6.061	6.792
414	14.815	14.396	13.018	11.892	11.077	9.277	9.599	8.31	7.182	5.86	6.565
415	14.191	13.837	12.472	11.465	10.676	8.936	9.249	8.011	6.93	5.678	6.353
416	13.618	13.244	11.984	11.006	10.28	8.595	8.894	7.739	6.68	5.489	6.145
417	13.071	12.736	11.484	10.595	9.905	8.278	8.596	7.47	6.476	5.315	5.944
418	12.579	12.205	11.096	10.176	9.536	7.969	8.276	7.223	6.267	5.156	5.75
419	12.156	11.86	10.756	9.868	9.252	7.741	8.049	7.038	6.086	5.034	5.613
420	11.688	11.443	10.357	9.527	8.916	7.495	7.788	6.805	5.908	4.89	5.429
421	11.231	11.003	9.974	9.172	8.62	7.245	7.531	6.617	5.732	4.773	5.269
422	10.819	10.619	9.618	8.838	8.335	7.021	7.314	6.434	5.58	4.655	5.148
423	10.421	10.247	9.271	8.535	8.08	6.805	7.089	6.265	5.46	4.555	5.037
424	10.039	9.893	8.978	8.273	7.815	6.615	6.896	6.101	5.325	4.472	4.938
425	9.667	9.56	8.674	8.008	7.587	6.408	6.704	5.951	5.193	4.385	4.853
426	9.319	9.217	8.364	7.738	7.36	6.241	6.538	5.799	5.096	4.333	4.783
427	8.988	8.913	8.089	7.506	7.155	6.092	6.369	5.67	4.997	4.251	4.725
428	8.659	8.605	7.815	7.281	6.964	5.92	6.206	5.572	4.911	4.202	4.678
429	8.351	8.326	7.606	7.058	6.77	5.78	6.066	5.452	4.832	4.168	4.627
430	8.049	8.044	7.371	6.835	6.576	5.647	5.916	5.347	4.753	4.126	4.584
431	7.76	7.76	7.117	6.647	6.393	5.521	5.768	5.258	4.694	4.082	4.553
432	7.462	7.51	6.902	6.45	6.237	5.404	5.653	5.16	4.624	4.062	4.518
433	7.189	7.257	6.664	6.251	6.081	5.277	5.534	5.075	4.567	4.024	4.484
434	6.929	7.007	6.444	6.063	5.915	5.162	5.407	4.99	4.505	3.978	4.449
435	6.636	6.754	6.24	5.892	5.773	5.042	5.3	4.926	4.454	3.971	4.421
436	6.439	6.559	6.104	5.765	5.647	4.946	5.226	4.862	4.415	3.951	4.401
437	6.224	6.321	5.907	5.596	5.521	4.867	5.105	4.777	4.371	3.93	4.365
438	5.989	6.127	5.731	5.446	5.386	4.756	5.02	4.719	4.322	3.918	4.355

EXCLI Journal 2020;19:48-70 – ISSN 1611-2156
Received: October 03, 2019, accepted: December 19, 2019, published: January 03, 2020

Emission intensity											
**Wavelength (nm)**	**concentration of β-hydroxyisovalerylshikonin (M)**										
	A	*B*	*C*	*D*	*E*	*F*	*G*	*I*	*J*	*K*	*L*
	0.00	$8.0x10^{-7}$	$1.6x10^{-6}$	$2.0x10^{-6}$	$2.8x10^{-6}$	$4.0x10^{-6}$	$4.8x10^{-6}$	$6.0x10^{-6}$	$8.0x10^{-6}$	$1.2x10^{-5}$	$1.6x10^{-5}$
439	5.744	5.919	5.542	5.279	5.25	4.681	4.926	4.651	4.281	3.892	4.324
440	5.519	5.723	5.369	5.138	5.128	4.584	4.839	4.611	4.245	3.879	4.293
441	5.309	5.512	5.222	5.011	5.005	4.493	4.775	4.537	4.229	3.864	4.265
442	5.09	5.333	5.054	4.871	4.901	4.423	4.674	4.479	4.189	3.867	4.241
443	4.881	5.158	4.907	4.738	4.788	4.338	4.597	4.439	4.157	3.848	4.242
444	4.695	4.961	4.742	4.611	4.686	4.265	4.524	4.396	4.139	3.86	4.22
445	4.508	4.783	4.59	4.481	4.581	4.204	4.464	4.349	4.123	3.853	4.226
446	4.322	4.627	4.453	4.371	4.482	4.137	4.418	4.307	4.097	3.845	4.226
447	4.148	4.478	4.328	4.267	4.401	4.08	4.345	4.283	4.088	3.861	4.221
448	3.983	4.321	4.219	4.176	4.321	4.037	4.294	4.257	4.079	3.872	4.23
449	3.825	4.178	4.105	4.072	4.245	3.978	4.257	4.218	4.073	3.881	4.252
450	3.69	4.042	3.986	3.983	4.171	3.927	4.209	4.204	4.058	3.894	4.265
451	3.542	3.918	3.893	3.899	4.095	3.888	4.159	4.185	4.053	3.915	4.295
452	3.399	3.795	3.787	3.814	4.021	3.844	4.117	4.169	4.061	3.926	4.324
453	3.285	3.676	3.674	3.751	3.957	3.816	4.08	4.154	4.07	3.944	4.344
454	3.173	3.591	3.611	3.693	3.922	3.788	4.054	4.133	4.067	3.965	4.371
455	3.043	3.474	3.516	3.61	3.871	3.759	4.029	4.121	4.073	4.008	4.385
456	2.938	3.369	3.438	3.564	3.817	3.721	3.989	4.111	4.085	4.02	4.424
457	2.82	3.272	3.356	3.473	3.777	3.687	3.962	4.107	4.08	4.051	4.454
458	2.72	3.173	3.281	3.411	3.713	3.645	3.946	4.098	4.089	4.059	4.465
459	2.615	3.092	3.208	3.354	3.66	3.632	3.919	4.092	4.088	4.076	4.503
460	2.511	2.998	3.129	3.294	3.609	3.601	3.875	4.073	4.091	4.085	4.529

EXCLI Journal 2020;19:48-70 – ISSN 1611-2156
Received: October 03, 2019, accepted: December 19, 2019, published: January 03, 2020

Table 4: Emission intensity in the wavelength range of 310-460 nm for fluorescence emission spectra of different concentration of α-methylbutyrylshikon (*A*-*L*)

Emission intensity											
**Wavelength (nm)**	**concentration of α-methylbutyrylshikon (M)**										
	A	*B*	*C*	*D*	*E*	*F*	*G*	*I*	*J*	*K*	*L*
	0.00	$8.0x10^{-7}$	$1.6x10^{-6}$	$2.0x10^{-6}$	$2.8x10^{-6}$	$4.0x10^{-6}$	$4.8x10^{-6}$	$6.0x10^{-6}$	$8.0x10^{-6}$	$1.2x10^{-5}$	$1.6x10^{-5}$
310	7.369	7.406	5.342	6.748	5.684	5.734	6.363	9.72	7.049	8.596	9.134
311	6.393	6.472	4.717	6.066	5.124	5.083	5.649	8.06	6.236	7.609	8.069
312	4.404	4.502	3.295	4.062	3.652	3.593	3.984	5.286	4.366	5.302	5.771
313	2.995	2.993	2.258	2.711	2.551	2.464	2.726	3.28	3.037	3.74	4.063
314	2.03	1.976	1.576	1.878	1.812	1.721	1.914	2.071	2.168	2.734	2.96
315	1.43	1.408	1.178	1.376	1.363	1.284	1.43	1.564	1.676	2.156	2.395
316	1.13	1.147	1.006	1.138	1.141	1.101	1.237	1.433	1.5	1.975	2.225
317	1.024	1.078	0.993	1.101	1.093	1.071	1.223	1.485	1.535	2.063	2.366
318	1.035	1.111	1.058	1.139	1.148	1.136	1.318	1.625	1.671	2.275	2.642
319	1.112	1.191	1.187	1.251	1.243	1.264	1.474	1.861	1.902	2.645	3.088
320	1.213	1.296	1.329	1.375	1.371	1.423	1.676	2.16	2.19	3.083	3.614
321	1.31	1.416	1.484	1.508	1.514	1.587	1.89	2.474	2.493	3.55	4.207
322	1.411	1.528	1.641	1.654	1.651	1.751	2.104	2.781	2.8	4.051	4.819
323	1.507	1.63	1.794	1.791	1.777	1.9	2.314	3.102	3.105	4.547	5.452
324	1.583	1.728	1.927	1.893	1.892	2.035	2.507	3.398	3.391	5.031	6.046
325	1.636	1.788	2.052	1.986	1.989	2.162	2.688	3.681	3.664	5.497	6.629
326	1.678	1.839	2.136	2.064	2.063	2.256	2.835	3.928	3.903	5.932	7.181
327	1.707	1.87	2.204	2.111	2.107	2.325	2.963	4.133	4.107	6.319	7.677
328	1.699	1.876	2.244	2.14	2.137	2.369	3.042	4.289	4.247	6.59	8.038
329	1.679	1.858	2.27	2.147	2.144	2.405	3.105	4.449	4.383	6.883	8.44
330	1.63	1.819	2.27	2.134	2.13	2.408	3.152	4.577	4.506	7.179	8.818
331	1.571	1.764	2.246	2.092	2.085	2.384	3.177	4.666	4.595	7.448	9.144
332	1.5	1.699	2.201	2.035	2.018	2.345	3.178	4.743	4.644	7.644	9.45
333	1.404	1.614	2.137	1.965	1.953	2.297	3.159	4.809	4.694	7.831	9.717
334	1.309	1.513	2.088	1.888	1.873	2.237	3.13	4.854	4.722	8.051	9.98
335	1.208	1.426	2.025	1.811	1.786	2.184	3.083	4.926	4.76	8.242	10.256
336	1.115	1.329	1.951	1.726	1.709	2.121	3.051	4.983	4.768	8.445	10.538
337	1.026	1.237	1.898	1.644	1.626	2.066	3.006	5.045	4.784	8.64	10.813
338	0.967	1.17	1.844	1.583	1.559	2.018	2.971	5.095	4.803	8.799	11.041
339	0.897	1.091	1.795	1.51	1.49	1.966	2.943	5.174	4.81	8.975	11.284

EXCLI Journal 2020;19:48-70 – ISSN 1611-2156
Received: October 03, 2019, accepted: December 19, 2019, published: January 03, 2020

Emission intensity											
**Wavelength (nm)**	**concentration of α-methylbutyrylshikon (M)**										
	A	*B*	*C*	*D*	*E*	*F*	*G*	*I*	*J*	*K*	*L*
	0.00	$8.0x10^{-7}$	$1.6x10^{-6}$	$2.0x10^{-6}$	$2.8x10^{-6}$	$4.0x10^{-6}$	$4.8x10^{-6}$	$6.0x10^{-6}$	$8.0x10^{-6}$	$1.2x10^{-5}$	$1.6x10^{-5}$
340	0.831	1.035	1.763	1.452	1.428	1.933	2.914	5.22	4.821	9.195	11.526
341	0.787	0.982	1.725	1.398	1.389	1.89	2.896	5.285	4.831	9.339	11.73
342	0.759	0.949	1.694	1.372	1.337	1.875	2.875	5.328	4.833	9.461	11.887
343	0.738	0.922	1.679	1.337	1.312	1.86	2.871	5.384	4.824	9.59	12.024
344	0.721	0.909	1.658	1.315	1.279	1.851	2.85	5.407	4.831	9.668	12.125
345	0.725	0.896	1.653	1.299	1.26	1.835	2.832	5.427	4.821	9.727	12.213
346	0.72	0.879	1.632	1.285	1.249	1.839	2.802	5.435	4.791	9.722	12.175
347	0.731	0.882	1.638	1.273	1.235	1.843	2.78	5.415	4.779	9.697	12.188
348	0.735	0.878	1.624	1.261	1.23	1.848	2.771	5.392	4.748	9.68	12.119
349	0.747	0.876	1.619	1.252	1.226	1.838	2.76	5.381	4.742	9.64	12.075
350	0.765	0.879	1.606	1.254	1.227	1.842	2.746	5.318	4.696	9.559	11.991
351	0.773	0.88	1.587	1.254	1.226	1.845	2.714	5.292	4.675	9.494	11.851
352	0.781	0.884	1.577	1.243	1.229	1.851	2.69	5.249	4.626	9.409	11.758
353	0.797	0.895	1.568	1.249	1.232	1.853	2.68	5.186	4.611	9.312	11.619
354	0.809	0.888	1.557	1.241	1.242	1.855	2.654	5.149	4.573	9.236	11.491
355	0.819	0.894	1.55	1.238	1.237	1.868	2.646	5.09	4.542	9.148	11.396
356	0.831	0.903	1.535	1.237	1.253	1.888	2.62	5.046	4.532	9.036	11.292
357	0.842	0.913	1.532	1.249	1.265	1.903	2.612	4.999	4.507	8.955	11.172
358	0.851	0.913	1.522	1.256	1.277	1.917	2.613	4.964	4.515	8.887	11.094
359	0.86	0.922	1.519	1.266	1.299	1.935	2.604	4.94	4.513	8.84	11.017
360	0.875	0.93	1.523	1.287	1.323	1.967	2.627	4.885	4.523	8.796	10.966
361	0.879	0.941	1.53	1.314	1.356	2.01	2.654	4.877	4.567	8.738	10.906
362	0.881	0.951	1.53	1.331	1.397	2.05	2.684	4.883	4.603	8.715	10.918
363	0.891	0.965	1.547	1.36	1.439	2.102	2.727	4.882	4.685	8.723	10.983
364	0.89	0.976	1.557	1.396	1.487	2.156	2.772	4.894	4.754	8.755	11.027
365	0.901	0.988	1.582	1.43	1.541	2.22	2.84	4.928	4.854	8.803	11.109
366	0.9	0.999	1.595	1.473	1.591	2.283	2.89	4.969	4.94	8.871	11.159
367	0.898	1.013	1.614	1.507	1.651	2.341	2.96	4.989	5.064	8.927	11.265
368	0.904	1.032	1.631	1.549	1.696	2.404	3.011	5.002	5.154	9.006	11.375
369	0.894	1.038	1.643	1.582	1.736	2.458	3.047	5.026	5.216	9.063	11.464
370	0.906	1.05	1.652	1.606	1.773	2.499	3.098	5.034	5.282	9.074	11.494
371	0.899	1.05	1.655	1.623	1.804	2.529	3.111	5.034	5.311	9.064	11.511
372	0.89	1.057	1.659	1.638	1.819	2.552	3.13	4.996	5.344	9.019	11.477

EXCLI Journal 2020;19:48-70 – ISSN 1611-2156
Received: October 03, 2019, accepted: December 19, 2019, published: January 03, 2020

Wavelength (nm)	*Emission intensity*										
	**concentration of α-methylbutyrylshikon (M)**										
	A	*B*	*C*	*D*	*E*	*F*	*G*	*I*	*J*	*K*	*L*
	0.00	$8.0x10^{-7}$	$1.6x10^{-6}$	$2.0x10^{-6}$	$2.8x10^{-6}$	$4.0x10^{-6}$	$4.8x10^{-6}$	$6.0x10^{-6}$	$8.0x10^{-6}$	$1.2x10^{-5}$	$1.6x10^{-5}$
373	0.887	1.055	1.643	1.637	1.845	2.559	3.13	4.949	5.33	8.929	11.372
374	0.889	1.053	1.636	1.651	1.848	2.564	3.106	4.894	5.295	8.828	11.249
375	0.884	1.049	1.609	1.636	1.855	2.555	3.091	4.831	5.247	8.676	11.06
376	0.873	1.039	1.595	1.635	1.843	2.54	3.051	4.743	5.196	8.532	10.871
377	0.865	1.026	1.572	1.622	1.842	2.51	3.022	4.639	5.134	8.375	10.673
378	0.858	1.023	1.548	1.604	1.836	2.5	2.983	4.561	5.08	8.218	10.488
379	0.85	1.012	1.531	1.598	1.832	2.486	2.95	4.492	5.02	8.083	10.32
380	0.848	1.009	1.511	1.588	1.818	2.467	2.93	4.43	4.99	7.947	10.165
381	0.833	0.999	1.502	1.588	1.825	2.466	2.92	4.382	4.95	7.882	10.065
382	0.827	0.994	1.494	1.584	1.818	2.464	2.908	4.34	4.936	7.777	9.976
383	0.816	0.992	1.486	1.585	1.825	2.464	2.902	4.3	4.919	7.73	9.892
384	0.807	0.995	1.478	1.584	1.838	2.452	2.891	4.266	4.907	7.693	9.873
385	0.792	0.991	1.475	1.596	1.829	2.467	2.887	4.257	4.906	7.648	9.814
386	0.792	0.981	1.466	1.595	1.845	2.469	2.892	4.226	4.9	7.605	9.773
387	0.776	0.97	1.462	1.591	1.851	2.468	2.885	4.205	4.893	7.532	9.673
388	0.773	0.973	1.439	1.588	1.835	2.459	2.855	4.17	4.853	7.462	9.61
389	0.757	0.96	1.433	1.572	1.826	2.442	2.835	4.107	4.796	7.355	9.489
390	0.743	0.943	1.415	1.558	1.81	2.408	2.797	4.029	4.729	7.215	9.307
391	0.73	0.932	1.385	1.538	1.798	2.367	2.734	3.935	4.644	7.069	9.092
392	0.72	0.916	1.361	1.5	1.764	2.336	2.678	3.834	4.538	6.886	8.877
393	0.715	0.9	1.326	1.474	1.723	2.284	2.622	3.723	4.43	6.695	8.634
394	0.702	0.885	1.292	1.438	1.696	2.232	2.564	3.626	4.326	6.522	8.41
395	0.686	0.872	1.262	1.408	1.643	2.181	2.464	3.5	4.189	6.282	8.11
396	0.676	0.853	1.218	1.376	1.605	2.111	2.389	3.381	4.054	6.047	7.806
397	0.669	0.824	1.179	1.339	1.566	2.055	2.32	3.252	3.917	5.823	7.509
398	0.651	0.815	1.138	1.299	1.533	1.996	2.242	3.134	3.776	5.607	7.225
399	0.642	0.794	1.109	1.269	1.488	1.941	2.177	3.029	3.682	5.409	6.98
400	0.633	0.774	1.08	1.229	1.444	1.884	2.11	2.935	3.558	5.22	6.711
401	0.614	0.763	1.049	1.2	1.411	1.833	2.046	2.834	3.451	5.038	6.478
402	0.602	0.749	1.016	1.174	1.37	1.798	1.987	2.75	3.346	4.904	6.28
403	0.592	0.728	1	1.144	1.34	1.745	1.942	2.672	3.26	4.753	6.101
404	0.588	0.72	0.97	1.117	1.32	1.707	1.889	2.614	3.166	4.613	5.927
405	0.579	0.705	0.95	1.096	1.29	1.666	1.844	2.544	3.104	4.49	5.759

EXCLI Journal 2020;19:48-70 – ISSN 1611-2156
Received: October 03, 2019, accepted: December 19, 2019, published: January 03, 2020

Emission intensity											
**Wavelength (nm)**	**concentration of α-methylbutyrylshikon (M)**										
	A	*B*	*C*	*D*	*E*	*F*	*G*	*I*	*J*	*K*	*L*
	0.00	$8.0x10^{-7}$	$1.6x10^{-6}$	$2.0x10^{-6}$	$2.8x10^{-6}$	$4.0x10^{-6}$	$4.8x10^{-6}$	$6.0x10^{-6}$	$8.0x10^{-6}$	$1.2x10^{-5}$	$1.6x10^{-5}$
406	0.565	0.694	0.932	1.076	1.258	1.628	1.799	2.482	3.031	4.376	5.609
407	0.553	0.684	0.919	1.065	1.246	1.599	1.772	2.422	2.971	4.296	5.501
408	0.547	0.673	0.9	1.041	1.211	1.568	1.72	2.364	2.903	4.169	5.374
409	0.537	0.661	0.884	1.016	1.2	1.537	1.685	2.294	2.827	4.059	5.215
410	0.531	0.65	0.864	0.998	1.178	1.494	1.636	2.236	2.751	3.951	5.051
411	0.52	0.637	0.84	0.976	1.145	1.456	1.585	2.17	2.675	3.813	4.89
412	0.513	0.626	0.823	0.953	1.118	1.429	1.55	2.09	2.596	3.706	4.722
413	0.508	0.615	0.801	0.928	1.091	1.39	1.503	2.04	2.52	3.572	4.571
414	0.5	0.608	0.777	0.911	1.062	1.358	1.466	1.969	2.447	3.449	4.42
415	0.497	0.597	0.755	0.892	1.051	1.32	1.421	1.91	2.365	3.336	4.254
416	0.484	0.584	0.747	0.871	1.021	1.284	1.376	1.847	2.296	3.21	4.091
417	0.489	0.575	0.722	0.847	0.997	1.258	1.334	1.788	2.229	3.11	3.944
418	0.479	0.577	0.708	0.833	0.976	1.226	1.302	1.732	2.171	3.008	3.82
419	0.486	0.565	0.7	0.82	0.962	1.212	1.269	1.682	2.117	2.927	3.726
420	0.475	0.565	0.687	0.804	0.947	1.187	1.236	1.642	2.067	2.844	3.608
421	0.479	0.559	0.669	0.788	0.932	1.159	1.206	1.606	2.022	2.772	3.489
422	0.473	0.549	0.665	0.771	0.912	1.148	1.188	1.581	1.973	2.696	3.404
423	0.477	0.549	0.652	0.762	0.897	1.126	1.169	1.544	1.928	2.653	3.32
424	0.469	0.545	0.645	0.76	0.889	1.115	1.15	1.519	1.9	2.588	3.269
425	0.475	0.535	0.639	0.751	0.886	1.1	1.132	1.495	1.871	2.558	3.198
426	0.473	0.534	0.634	0.743	0.87	1.09	1.121	1.469	1.854	2.512	3.144
427	0.474	0.534	0.623	0.738	0.872	1.076	1.111	1.458	1.815	2.48	3.098
428	0.469	0.523	0.626	0.73	0.859	1.081	1.1	1.442	1.807	2.437	3.06
429	0.466	0.53	0.618	0.728	0.848	1.066	1.083	1.422	1.784	2.424	3.017
430	0.459	0.531	0.617	0.721	0.85	1.06	1.072	1.414	1.772	2.399	2.982
431	0.458	0.526	0.609	0.714	0.843	1.053	1.06	1.398	1.741	2.366	2.947
432	0.468	0.523	0.614	0.709	0.827	1.048	1.047	1.371	1.74	2.343	2.903
433	0.462	0.519	0.602	0.7	0.826	1.035	1.045	1.365	1.711	2.311	2.863
434	0.469	0.518	0.592	0.703	0.835	1.028	1.023	1.348	1.697	2.275	2.833
435	0.469	0.515	0.594	0.694	0.816	1.019	1.016	1.327	1.684	2.244	2.786
436	0.464	0.516	0.591	0.685	0.815	1.014	1.015	1.315	1.657	2.225	2.747
437	0.464	0.516	0.591	0.685	0.815	1.014	1.015	1.315	1.657	2.225	2.717
438	0.452	0.506	0.578	0.672	0.804	1.002	0.985	1.293	1.623	2.159	2.672

EXCLI Journal 2020;19:48-70 – ISSN 1611-2156
Received: October 03, 2019, accepted: December 19, 2019, published: January 03, 2020

Emission intensity											
**Wavelength (nm)**	**concentration of α-methylbutyrylshikon (M)**										
	A	*B*	*C*	*D*	*E*	*F*	*G*	*I*	*J*	*K*	*L*
	0.00	$8.0x10^{-7}$	$1.6x10^{-6}$	$2.0x10^{-6}$	$2.8x10^{-6}$	$4.0x10^{-6}$	$4.8x10^{-6}$	$6.0x10^{-6}$	$8.0x10^{-6}$	$1.2x10^{-5}$	$1.6x10^{-5}$
439	0.454	0.506	0.577	0.666	0.797	0.986	0.974	1.272	1.598	2.137	2.629
440	0.456	0.501	0.569	0.654	0.79	0.974	0.969	1.257	1.58	2.112	2.603
441	0.454	0.496	0.564	0.65	0.785	0.972	0.959	1.253	1.563	2.087	2.562
442	0.451	0.495	0.557	0.649	0.779	0.956	0.949	1.233	1.546	2.056	2.521
443	0.448	0.491	0.554	0.642	0.773	0.947	0.93	1.216	1.532	2.031	2.49
444	0.442	0.492	0.551	0.636	0.764	0.944	0.927	1.21	1.516	2.012	2.456
445	0.449	0.488	0.547	0.63	0.757	0.933	0.917	1.193	1.501	1.991	2.434
446	0.447	0.485	0.538	0.627	0.753	0.924	0.909	1.181	1.488	1.97	2.411
447	0.446	0.48	0.537	0.622	0.749	0.921	0.899	1.178	1.477	1.956	2.393
448	0.444	0.476	0.53	0.62	0.755	0.908	0.892	1.168	1.47	1.949	2.378
449	0.443	0.476	0.53	0.613	0.745	0.906	0.885	1.165	1.459	1.931	2.358
450	0.444	0.472	0.524	0.607	0.745	0.904	0.886	1.162	1.455	1.931	2.348
451	0.44	0.468	0.522	0.608	0.747	0.901	0.885	1.157	1.444	1.913	2.338
452	0.442	0.467	0.524	0.607	0.745	0.901	0.881	1.153	1.443	1.907	2.337
453	0.442	0.467	0.524	0.6	0.738	0.89	0.876	1.156	1.443	1.9	2.335
454	0.439	0.465	0.522	0.595	0.738	0.887	0.881	1.151	1.438	1.902	2.326
455	0.443	0.462	0.517	0.596	0.742	0.884	0.875	1.147	1.432	1.916	2.321
456	0.439	0.464	0.516	0.592	0.74	0.885	0.872	1.141	1.429	1.905	2.329
457	0.44	0.462	0.512	0.59	0.737	0.878	0.872	1.15	1.422	1.898	2.325
458	0.436	0.454	0.514	0.588	0.733	0.878	0.872	1.14	1.422	1.891	2.322
459	0.434	0.452	0.515	0.586	0.734	0.881	0.866	1.141	1.416	1.899	2.316
460	0.435	0.454	0.511	0.585	0.731	0.873	0.861	1.134	1.415	1.888	2.313

EXCLI Journal 2020;19:48-70 – ISSN 1611-2156
Received: October 03, 2019, accepted: December 19, 2019, published: January 03, 2020

Table 5: Emission intensity in the wavelength range of 310-460 nm for fluorescence emission spectra of different concentration of acetylshikonin (*A*-*L*)

Wavelength (nm)	***Emission intensity***										
	**concentration of acetylshikonin (M)**										
	A	*B*	*C*	*D*	*E*	*F*	*G*	*I*	*J*	*K*	*L*
	0.00	$8.0x10^{-7}$	$1.6x10^{-6}$	$2.0x10^{-6}$	$2.8x10^{-6}$	$4.0x10^{-6}$	$4.8x10^{-6}$	$6.0x10^{-6}$	$8.0x10^{-6}$	$1.2x10^{-5}$	$1.6x10^{-5}$
310	7.406	5.746	4.979	4.131	4.367	5.142	4.655	5.366	7.911	8.315	10.173
311	6.442	5.063	4.46	3.653	3.88	4.61	4.097	4.751	6.931	7.463	9.005
312	4.042	3.496	3.153	2.58	2.771	3.281	2.896	3.371	4.804	5.338	6.366
313	2.465	2.371	2.155	1.819	1.957	2.301	2.056	2.374	3.226	3.759	4.365
314	1.573	1.646	1.501	1.3	1.435	1.663	1.516	1.709	2.195	2.729	3.062
315	1.158	1.213	1.116	1.027	1.143	1.284	1.248	1.34	1.63	2.115	2.271
316	0.962	1.019	0.953	0.939	1.049	1.158	1.171	1.198	1.441	1.91	1.968
317	0.902	0.99	0.943	0.967	1.094	1.184	1.229	1.23	1.479	1.969	1.949
318	0.934	1.03	0.999	1.038	1.196	1.279	1.348	1.333	1.639	2.172	2.097
319	1.013	1.126	1.114	1.182	1.354	1.455	1.545	1.522	1.886	2.519	2.385
320	1.109	1.247	1.244	1.354	1.555	1.667	1.764	1.738	2.191	2.935	2.757
321	1.197	1.359	1.377	1.521	1.746	1.884	2.018	1.969	2.502	3.391	3.156
322	1.292	1.485	1.517	1.686	1.957	2.103	2.263	2.201	2.833	3.863	3.564
323	1.38	1.592	1.648	1.83	2.15	2.317	2.495	2.448	3.172	4.351	3.983
324	1.445	1.695	1.761	1.966	2.321	2.515	2.718	2.661	3.469	4.781	4.389
325	1.498	1.771	1.865	2.093	2.491	2.685	2.926	2.854	3.754	5.208	4.774
326	1.53	1.814	1.928	2.182	2.614	2.844	3.106	3.029	4.025	5.621	5.12
327	1.538	1.856	1.979	2.247	2.738	2.956	3.252	3.166	4.225	5.998	5.435
328	1.528	1.858	2.003	2.284	2.802	3.032	3.351	3.261	4.385	6.262	5.689
329	1.504	1.857	2.008	2.314	2.857	3.122	3.444	3.354	4.53	6.54	5.939
330	1.454	1.821	1.987	2.311	2.895	3.154	3.504	3.421	4.68	6.801	6.155
331	1.38	1.774	1.944	2.294	2.907	3.161	3.539	3.448	4.783	7.025	6.341
332	1.296	1.7	1.89	2.242	2.888	3.161	3.562	3.451	4.86	7.206	6.494
333	1.203	1.623	1.824	2.203	2.872	3.142	3.576	3.45	4.909	7.397	6.621
334	1.105	1.537	1.742	2.143	2.84	3.126	3.57	3.433	4.955	7.553	6.748
335	0.996	1.443	1.671	2.074	2.825	3.082	3.569	3.418	4.989	7.727	6.848
336	0.895	1.347	1.593	2.001	2.796	3.048	3.546	3.403	5.052	7.901	6.955
337	0.797	1.272	1.509	1.946	2.761	3.022	3.54	3.378	5.069	8.066	7.039
338	0.721	1.206	1.455	1.9	2.756	2.986	3.535	3.348	5.087	8.203	7.118
339	0.65	1.136	1.404	1.845	2.755	2.958	3.547	3.332	5.125	8.37	7.179

Emission intensity											
**Wavelength (nm)**	**concentration of acetylshikonin (M)**										
	A	*B*	*C*	*D*	*E*	*F*	*G*	*I*	*J*	*K*	*L*
	0.00	$8.0x10^{-7}$	$1.6x10^{-6}$	$2.0x10^{-6}$	$2.8x10^{-6}$	$4.0x10^{-6}$	$4.8x10^{-6}$	$6.0x10^{-6}$	$8.0x10^{-6}$	$1.2x10^{-5}$	$1.6x10^{-5}$
340	0.581	1.071	1.354	1.784	2.741	2.925	3.554	3.316	5.143	8.544	7.277
341	0.529	1.02	1.315	1.744	2.725	2.905	3.553	3.296	5.173	8.671	7.344
342	0.491	0.985	1.283	1.71	2.731	2.884	3.549	3.281	5.199	8.786	7.397
343	0.462	0.961	1.261	1.685	2.737	2.882	3.558	3.277	5.199	8.88	7.421
344	0.434	0.933	1.238	1.661	2.737	2.859	3.545	3.269	5.204	8.946	7.434
345	0.423	0.914	1.235	1.631	2.737	2.835	3.534	3.26	5.177	8.973	7.449
346	0.414	0.897	1.23	1.604	2.724	2.824	3.521	3.246	5.176	9.008	7.435
347	0.412	0.892	1.218	1.597	2.713	2.811	3.525	3.227	5.143	8.984	7.415
348	0.41	0.883	1.219	1.578	2.706	2.788	3.496	3.197	5.127	8.926	7.366
349	0.416	0.872	1.218	1.565	2.69	2.761	3.477	3.19	5.085	8.887	7.348
350	0.413	0.86	1.21	1.559	2.674	2.737	3.451	3.184	5.046	8.843	7.292
351	0.423	0.864	1.205	1.547	2.649	2.725	3.418	3.166	5.004	8.745	7.236
352	0.432	0.856	1.202	1.531	2.628	2.693	3.397	3.143	4.941	8.65	7.186
353	0.429	0.856	1.201	1.524	2.611	2.665	3.353	3.123	4.897	8.555	7.149
354	0.445	0.848	1.191	1.516	2.583	2.637	3.335	3.099	4.861	8.486	7.119
355	0.453	0.851	1.19	1.504	2.569	2.621	3.317	3.084	4.802	8.393	7.045
356	0.455	0.849	1.188	1.495	2.55	2.594	3.293	3.061	4.761	8.304	7.038
357	0.456	0.853	1.178	1.49	2.536	2.602	3.255	3.05	4.724	8.231	7.022
358	0.464	0.853	1.174	1.475	2.514	2.582	3.246	3.056	4.72	8.185	6.993
359	0.468	0.846	1.177	1.468	2.501	2.58	3.235	3.056	4.687	8.14	7.021
360	0.47	0.852	1.19	1.475	2.491	2.584	3.229	3.068	4.686	8.071	7.036
361	0.468	0.851	1.203	1.483	2.474	2.617	3.235	3.118	4.695	8.049	7.116
362	0.481	0.852	1.209	1.487	2.488	2.623	3.253	3.163	4.718	8.055	7.192
363	0.474	0.866	1.23	1.499	2.492	2.646	3.278	3.197	4.753	8.052	7.321
364	0.478	0.867	1.273	1.51	2.492	2.692	3.315	3.264	4.816	8.079	7.478
365	0.485	0.877	1.292	1.523	2.508	2.722	3.339	3.33	4.892	8.132	7.651
366	0.476	0.882	1.318	1.54	2.523	2.778	3.394	3.399	4.954	8.191	7.826
367	0.484	0.9	1.343	1.566	2.547	2.831	3.438	3.473	5.039	8.261	7.982
368	0.474	0.909	1.357	1.574	2.557	2.875	3.476	3.553	5.092	8.327	8.142
369	0.479	0.919	1.378	1.588	2.574	2.901	3.506	3.591	5.159	8.371	8.288
370	0.472	0.924	1.384	1.605	2.578	2.933	3.533	3.649	5.198	8.394	8.403
371	0.477	0.922	1.406	1.602	2.564	2.949	3.537	3.676	5.222	8.369	8.474
372	0.473	0.93	1.412	1.602	2.553	2.946	3.518	3.695	5.208	8.351	8.542

EXCLI Journal 2020;19:48-70 – ISSN 1611-2156
Received: October 03, 2019, accepted: December 19, 2019, published: January 03, 2020

Wavelength (nm)	***Emission intensity*** **concentration of acetylshikonin (M)**										
	A	*B*	*C*	*D*	*E*	*F*	*G*	*I*	*J*	*K*	*L*
	0.00	$8.0x10^{-7}$	$1.6x10^{-6}$	$2.0x10^{-6}$	$2.8x10^{-6}$	$4.0x10^{-6}$	$4.8x10^{-6}$	$6.0x10^{-6}$	$8.0x10^{-6}$	$1.2x10^{-5}$	$1.6x10^{-5}$
373	0.471	0.933	1.405	1.589	2.528	2.936	3.505	3.691	5.193	8.261	8.529
374	0.458	0.929	1.402	1.579	2.502	2.919	3.477	3.665	5.147	8.157	8.522
375	0.462	0.922	1.386	1.565	2.456	2.887	3.431	3.627	5.091	8.015	8.44
376	0.461	0.913	1.375	1.544	2.421	2.851	3.377	3.6	5.001	7.897	8.359
377	0.453	0.912	1.36	1.525	2.376	2.82	3.326	3.553	4.926	7.737	8.245
378	0.446	0.91	1.338	1.5	2.344	2.787	3.28	3.513	4.86	7.613	8.163
379	0.445	0.91	1.326	1.486	2.302	2.76	3.231	3.485	4.792	7.459	8.083
380	0.441	0.902	1.31	1.475	2.279	2.736	3.191	3.455	4.752	7.365	8.009
381	0.438	0.899	1.294	1.468	2.261	2.722	3.177	3.443	4.714	7.304	7.98
382	0.437	0.9	1.288	1.462	2.246	2.696	3.158	3.431	4.684	7.221	7.958
383	0.432	0.897	1.278	1.454	2.234	2.69	3.125	3.434	4.68	7.173	7.933
384	0.426	0.9	1.26	1.457	2.222	2.694	3.125	3.417	4.661	7.143	7.93
385	0.416	0.904	1.258	1.45	2.214	2.675	3.139	3.42	4.664	7.098	7.936
386	0.416	0.907	1.233	1.438	2.201	2.675	3.109	3.407	4.645	7.077	7.926
387	0.411	0.901	1.222	1.433	2.179	2.671	3.086	3.408	4.631	7.01	7.908
388	0.408	0.886	1.214	1.419	2.159	2.647	3.054	3.382	4.576	6.939	7.864
389	0.406	0.884	1.208	1.413	2.133	2.612	3.015	3.347	4.535	6.826	7.778
390	0.399	0.883	1.189	1.384	2.1	2.576	2.971	3.295	4.452	6.717	7.674
391	0.393	0.877	1.161	1.36	2.042	2.514	2.906	3.246	4.367	6.585	7.542
392	0.393	0.862	1.146	1.324	2.008	2.473	2.842	3.172	4.258	6.414	7.37
393	0.395	0.833	1.12	1.299	1.947	2.399	2.745	3.088	4.142	6.207	7.213
394	0.391	0.826	1.099	1.276	1.91	2.351	2.695	3.022	4.053	6.068	7.038
395	0.388	0.81	1.063	1.23	1.846	2.277	2.603	2.939	3.918	5.842	6.785
396	0.376	0.787	1.042	1.198	1.787	2.201	2.521	2.838	3.786	5.654	6.573
397	0.382	0.758	1.015	1.171	1.737	2.135	2.427	2.739	3.651	5.422	6.345
398	0.373	0.742	0.978	1.132	1.685	2.066	2.365	2.66	3.522	5.227	6.126
399	0.369	0.723	0.959	1.113	1.637	2.003	2.276	2.576	3.415	5.05	5.933
400	0.367	0.71	0.932	1.078	1.582	1.946	2.198	2.498	3.305	4.861	5.764
401	0.357	0.686	0.907	1.059	1.544	1.894	2.136	2.42	3.202	4.732	5.571
402	0.362	0.664	0.888	1.029	1.511	1.84	2.083	2.368	3.118	4.581	5.41
403	0.359	0.633	0.874	1.005	1.473	1.789	2.021	2.304	3.041	4.445	5.272
404	0.35	0.609	0.853	0.985	1.437	1.756	1.977	2.252	2.957	4.341	5.138
405	0.345	0.599	0.839	0.969	1.405	1.713	1.935	2.2	2.891	4.213	5.015

EXCLI Journal 2020;19:48-70 – ISSN 1611-2156
Received: October 03, 2019, accepted: December 19, 2019, published: January 03, 2020

Emission intensity											
**Wavelength (nm)**	**concentration of acetylshikonin (M)**										
	A	*B*	*C*	*D*	*E*	*F*	*G*	*I*	*J*	*K*	*L*
	0.00	$8.0x10^{-7}$	$1.6x10^{-6}$	$2.0x10^{-6}$	$2.8x10^{-6}$	$4.0x10^{-6}$	$4.8x10^{-6}$	$6.0x10^{-6}$	$8.0x10^{-6}$	$1.2x10^{-5}$	$1.6x10^{-5}$
406	0.343	0.585	0.82	0.944	1.377	1.677	1.882	2.152	2.824	4.112	4.895
407	0.342	0.576	0.807	0.926	1.365	1.65	1.854	2.121	2.778	4.029	4.816
408	0.338	0.571	0.784	0.914	1.335	1.606	1.808	2.058	2.709	3.929	4.709
409	0.33	0.562	0.769	0.897	1.3	1.575	1.755	2.015	2.639	3.824	4.59
410	0.332	0.55	0.754	0.878	1.277	1.529	1.711	1.968	2.575	3.724	4.482
411	0.325	0.541	0.741	0.858	1.237	1.495	1.673	1.905	2.498	3.597	4.347
412	0.319	0.532	0.719	0.84	1.212	1.457	1.615	1.851	2.417	3.492	4.222
413	0.319	0.523	0.711	0.812	1.18	1.411	1.568	1.804	2.346	3.372	4.082
414	0.315	0.513	0.69	0.799	1.146	1.366	1.526	1.753	2.283	3.265	3.957
415	0.312	0.5	0.676	0.788	1.124	1.333	1.476	1.703	2.207	3.156	3.831
416	0.314	0.499	0.656	0.765	1.087	1.295	1.429	1.659	2.148	3.054	3.712
417	0.313	0.484	0.641	0.75	1.062	1.26	1.391	1.611	2.076	2.962	3.617
418	0.312	0.478	0.634	0.738	1.039	1.235	1.35	1.566	2.021	2.854	3.504
419	0.312	0.475	0.624	0.729	1.028	1.209	1.323	1.54	1.98	2.797	3.423
420	0.31	0.479	0.617	0.713	1.012	1.178	1.303	1.507	1.938	2.725	3.319
421	0.303	0.47	0.602	0.701	0.99	1.158	1.275	1.473	1.878	2.66	3.259
422	0.304	0.465	0.6	0.696	0.981	1.138	1.239	1.455	1.85	2.593	3.179
423	0.297	0.46	0.594	0.686	0.967	1.12	1.224	1.427	1.824	2.539	3.118
424	0.306	0.457	0.584	0.686	0.959	1.11	1.204	1.403	1.792	2.502	3.082
425	0.298	0.463	0.582	0.679	0.952	1.099	1.194	1.389	1.762	2.456	3.036
426	0.302	0.459	0.583	0.672	0.945	1.089	1.181	1.371	1.758	2.417	3.001
427	0.303	0.457	0.578	0.672	0.94	1.076	1.174	1.367	1.737	2.403	2.966
428	0.294	0.456	0.573	0.663	0.931	1.068	1.165	1.345	1.716	2.371	2.937
429	0.302	0.457	0.579	0.671	0.933	1.06	1.148	1.343	1.698	2.341	2.906
430	0.298	0.46	0.568	0.662	0.931	1.057	1.137	1.329	1.689	2.325	2.888
431	0.299	0.453	0.566	0.654	0.92	1.05	1.125	1.321	1.675	2.307	2.85
432	0.294	0.453	0.561	0.656	0.917	1.05	1.13	1.317	1.658	2.28	2.838
433	0.301	0.451	0.562	0.655	0.905	1.038	1.111	1.302	1.648	2.252	2.806
434	0.297	0.447	0.553	0.65	0.908	1.025	1.11	1.294	1.626	2.239	2.772
435	0.288	0.444	0.557	0.647	0.907	1.017	1.096	1.286	1.606	2.209	2.742
436	0.292	0.443	0.551	0.643	0.903	1.014	1.092	1.277	1.598	2.193	2.736
437	0.292	0.443	0.551	0.643	0.903	1.014	1.092	1.277	1.598	2.193	2.736
438	0.291	0.438	0.541	0.633	0.887	0.991	1.067	1.259	1.572	2.146	2.669

EXCLI Journal 2020;19:48-70 – ISSN 1611-2156
Received: October 03, 2019, accepted: December 19, 2019, published: January 03, 2020

Wavelength (nm)	***Emission intensity***										
	**concentration of acetylshikonin (M)**										
	A	*B*	*C*	*D*	*E*	*F*	*G*	*I*	*J*	*K*	*L*
	0.00	$8.0x10^{-7}$	$1.6x10^{-6}$	$2.0x10^{-6}$	$2.8x10^{-6}$	$4.0x10^{-6}$	$4.8x10^{-6}$	$6.0x10^{-6}$	$8.0x10^{-6}$	$1.2x10^{-5}$	$1.6x10^{-5}$
439	0.288	0.432	0.538	0.635	0.884	0.99	1.058	1.242	1.555	2.136	2.645
440	0.282	0.432	0.54	0.625	0.883	0.968	1.057	1.23	1.542	2.115	2.619
441	0.286	0.428	0.54	0.623	0.882	0.968	1.048	1.221	1.525	2.108	2.592
442	0.28	0.426	0.536	0.618	0.88	0.959	1.037	1.21	1.523	2.101	2.584
443	0.275	0.423	0.527	0.612	0.873	0.954	1.028	1.207	1.517	2.086	2.552
444	0.278	0.42	0.527	0.613	0.868	0.945	1.021	1.197	1.501	2.075	2.537
445	0.273	0.419	0.522	0.613	0.867	0.94	1.014	1.192	1.489	2.08	2.527
446	0.269	0.416	0.528	0.603	0.872	0.937	1.018	1.196	1.48	2.069	2.522
447	0.268	0.415	0.521	0.604	0.868	0.933	1.013	1.181	1.483	2.06	2.511
448	0.271	0.415	0.519	0.606	0.861	0.929	1.006	1.19	1.484	2.063	2.489
449	0.267	0.413	0.516	0.604	0.866	0.926	1.004	1.188	1.481	2.067	2.503
450	0.267	0.412	0.516	0.603	0.863	0.927	1.005	1.188	1.484	2.069	2.507
451	0.266	0.414	0.512	0.604	0.872	0.932	1.012	1.187	1.491	2.078	2.513
452	0.264	0.407	0.517	0.608	0.867	0.925	1.011	1.197	1.489	2.079	2.515
453	0.265	0.411	0.517	0.607	0.873	0.934	1.018	1.192	1.494	2.097	2.523
454	0.264	0.408	0.519	0.606	0.874	0.932	1.02	1.189	1.496	2.097	2.532
455	0.258	0.409	0.518	0.606	0.872	0.938	1.014	1.19	1.498	2.1	2.543
456	0.258	0.409	0.511	0.608	0.877	0.935	1.02	1.198	1.502	2.112	2.553
457	0.26	0.403	0.518	0.604	0.864	0.94	1.023	1.202	1.502	2.102	2.555
458	0.254	0.403	0.513	0.6	0.867	0.945	1.024	1.199	1.503	2.11	2.56
459	0.257	0.406	0.52	0.601	0.875	0.944	1.023	1.2	1.507	2.107	2.577
460	0.248	0.403	0.515	0.608	0.869	0.945	1.023	1.201	1.519	2.114	2.579

EXCLI Journal 2020;19:48-70 – ISSN 1611-2156
Received: October 03, 2019, accepted: December 19, 2019, published: January 03, 2020

Table 6: Emission intensity in the wavelength range of 310-460 nm for fluorescence emission spectra of different concentration of β-hydroxy-isovalerylshikonin (*A-L*)

Wavelength (nm)	***Emission intensity***										
	**concentration of β-hydroxyisovalerylshikonin (M)**										
	A	*B*	*C*	*D*	*E*	*F*	*G*	*I*	*J*	*K*	*L*
	0.00	$8.0x10^{-7}$	$1.6x10^{-6}$	$2.0x10^{-6}$	$2.8x10^{-6}$	$4.0x10^{-6}$	$4.8x10^{-6}$	$6.0x10^{-6}$	$8.0x10^{-6}$	$1.2x10^{-5}$	$1.6x10^{-5}$
310	6.233	5.393	4.217	4.337	3.798	5.159	7.549	7.363	6.65	7.531	10.485
311	5.773	4.792	3.754	3.86	3.412	4.584	6.694	6.562	5.929	6.743	9.34
312	4.165	3.392	2.637	2.737	2.43	3.238	4.496	4.624	4.229	4.76	6.515
313	2.892	2.363	1.851	1.913	1.733	2.276	2.87	3.154	2.993	3.311	4.444
314	1.971	1.664	1.319	1.376	1.295	1.648	1.917	2.15	2.179	2.371	3.055
315	1.35	1.235	1.029	1.061	1.041	1.293	1.455	1.541	1.728	1.856	2.233
316	1.045	1.033	0.912	0.935	0.958	1.18	1.327	1.29	1.573	1.688	1.871
317	0.959	0.972	0.92	0.92	0.999	1.198	1.366	1.281	1.625	1.773	1.797
318	0.97	1.007	0.994	0.966	1.094	1.297	1.48	1.371	1.777	1.943	1.886
319	1.037	1.088	1.113	1.069	1.223	1.481	1.662	1.528	2.042	2.241	2.067
320	1.127	1.195	1.258	1.183	1.411	1.683	1.9	1.733	2.361	2.59	2.322
321	1.214	1.306	1.405	1.3	1.573	1.901	2.13	1.932	2.699	2.973	2.596
322	1.313	1.408	1.549	1.427	1.759	2.123	2.374	2.144	3.069	3.378	2.874
323	1.392	1.504	1.685	1.525	1.924	2.334	2.626	2.357	3.412	3.765	3.148
324	1.454	1.586	1.808	1.611	2.083	2.534	2.845	2.53	3.747	4.135	3.4
325	1.499	1.652	1.91	1.683	2.219	2.711	3.045	2.712	4.065	4.504	3.66
326	1.543	1.689	1.992	1.741	2.321	2.857	3.228	2.858	4.344	4.845	3.872
327	1.56	1.709	2.048	1.769	2.416	2.972	3.372	2.966	4.583	5.142	4.073
328	1.555	1.718	2.075	1.777	2.466	3.059	3.468	3.048	4.768	5.361	4.196
329	1.523	1.699	2.087	1.772	2.489	3.13	3.56	3.105	4.959	5.593	4.343
330	1.483	1.666	2.066	1.737	2.505	3.187	3.618	3.147	5.12	5.774	4.461
331	1.412	1.609	2.033	1.681	2.486	3.193	3.659	3.176	5.253	5.952	4.55
332	1.323	1.534	1.986	1.613	2.465	3.192	3.698	3.159	5.356	6.078	4.605
333	1.237	1.453	1.924	1.522	2.422	3.187	3.694	3.146	5.435	6.198	4.671
334	1.139	1.365	1.858	1.439	2.363	3.173	3.713	3.118	5.498	6.313	4.694
335	1.042	1.277	1.783	1.351	2.304	3.138	3.709	3.076	5.598	6.404	4.705
336	0.941	1.192	1.712	1.259	2.242	3.102	3.711	3.046	5.677	6.497	4.752
337	0.842	1.118	1.644	1.183	2.191	3.072	3.71	3.007	5.752	6.581	4.765
338	0.776	1.056	1.6	1.119	2.145	3.056	3.733	2.982	5.813	6.644	4.78
339	0.7	0.986	1.532	1.055	2.089	3.023	3.736	2.959	5.864	6.726	4.802

EXCLI Journal 2020;19:48-70 – ISSN 1611-2156
Received: October 03, 2019, accepted: December 19, 2019, published: January 03, 2020

Wavelength (nm)	*Emission intensity* concentration of *β*-hydroxyisovalerylshikonin (M)										
	A	*B*	*C*	*D*	*E*	*F*	*G*	*I*	*J*	*K*	*L*
	0.00	$8.0x10^{-7}$	$1.6x10^{-6}$	$2.0x10^{-6}$	$2.8x10^{-6}$	$4.0x10^{-6}$	$4.8x10^{-6}$	$6.0x10^{-6}$	$8.0x10^{-6}$	$1.2x10^{-5}$	$1.6x10^{-5}$
340	0.635	0.941	1.489	1.003	2.051	3.006	3.753	2.934	5.94	6.786	4.827
341	0.586	0.907	1.455	0.958	2.022	2.988	3.781	2.909	6.003	6.854	4.836
342	0.548	0.893	1.423	0.932	1.992	2.982	3.783	2.899	6.046	6.899	4.865
343	0.525	0.88	1.399	0.904	1.965	2.966	3.806	2.886	6.085	6.932	4.879
344	0.501	0.876	1.387	0.897	1.954	2.95	3.811	2.866	6.125	6.952	4.892
345	0.488	0.878	1.367	0.893	1.929	2.937	3.819	2.863	6.143	6.95	4.908
346	0.477	0.887	1.346	0.896	1.913	2.93	3.818	2.85	6.115	6.919	4.921
347	0.475	0.903	1.347	0.899	1.913	2.897	3.788	2.83	6.094	6.906	4.933
348	0.478	0.915	1.338	0.902	1.888	2.891	3.795	2.83	6.083	6.86	4.926
349	0.482	0.938	1.324	0.918	1.881	2.865	3.773	2.823	6.045	6.815	4.942
350	0.489	0.953	1.329	0.929	1.871	2.847	3.745	2.803	6.013	6.745	4.938
351	0.497	0.969	1.32	0.935	1.85	2.814	3.732	2.798	5.959	6.686	4.944
352	0.504	0.99	1.312	0.949	1.837	2.785	3.695	2.792	5.898	6.628	4.955
353	0.506	1.015	1.299	0.964	1.833	2.761	3.672	2.79	5.827	6.559	4.98
354	0.513	1.034	1.301	0.974	1.819	2.744	3.657	2.796	5.783	6.489	5.006
355	0.518	1.05	1.294	0.992	1.801	2.71	3.628	2.788	5.725	6.446	5.028
356	0.529	1.068	1.297	1.005	1.796	2.695	3.6	2.786	5.677	6.401	5.077
357	0.533	1.08	1.287	1.028	1.782	2.684	3.594	2.791	5.622	6.362	5.149
358	0.533	1.099	1.285	1.051	1.782	2.672	3.561	2.819	5.591	6.335	5.211
359	0.54	1.119	1.285	1.07	1.785	2.661	3.552	2.84	5.563	6.321	5.297
360	0.544	1.131	1.292	1.098	1.788	2.65	3.547	2.865	5.534	6.343	5.404
361	0.548	1.137	1.292	1.125	1.803	2.68	3.553	2.913	5.546	6.344	5.556
362	0.548	1.151	1.31	1.145	1.825	2.7	3.57	2.983	5.525	6.401	5.729
363	0.549	1.161	1.319	1.181	1.849	2.711	3.571	3.057	5.551	6.468	5.922
364	0.553	1.176	1.334	1.201	1.862	2.734	3.589	3.129	5.594	6.566	6.14
365	0.552	1.188	1.352	1.237	1.898	2.773	3.622	3.213	5.65	6.67	6.374
366	0.554	1.192	1.363	1.261	1.941	2.816	3.664	3.305	5.706	6.82	6.626
367	0.553	1.201	1.387	1.299	1.978	2.854	3.68	3.389	5.762	6.94	6.885
368	0.562	1.194	1.403	1.324	2.005	2.892	3.718	3.485	5.818	7.05	7.123
369	0.558	1.202	1.422	1.349	2.029	2.919	3.737	3.551	5.85	7.125	7.313
370	0.548	1.208	1.422	1.374	2.037	2.936	3.748	3.613	5.886	7.209	7.509
371	0.554	1.205	1.431	1.388	2.06	2.942	3.754	3.656	5.891	7.281	7.677
372	0.553	1.205	1.426	1.399	2.061	2.952	3.727	3.699	5.871	7.284	7.802

EXCLI Journal 2020;19:48-70 – ISSN 1611-2156
Received: October 03, 2019, accepted: December 19, 2019, published: January 03, 2020

Wavelength (nm)	Emission intensity										
	concentration of β-hydroxyisovalerylshikonin (M)										
	A	B	C	D	E	F	G	I	J	K	L
	0.00	8.0x10^{-7}	1.6x10^{-6}	2.0x10^{-6}	2.8x10^{-6}	4.0x10^{-6}	4.8x10^{-6}	6.0x10^{-6}	8.0x10^{-6}	1.2x10^{-5}	1.6x10^{-5}
373	0.551	1.189	1.414	1.411	2.061	2.932	3.698	3.716	5.818	7.259	7.857
374	0.552	1.187	1.414	1.407	2.052	2.909	3.659	3.714	5.754	7.204	7.919
375	0.545	1.175	1.394	1.409	2.025	2.87	3.616	3.672	5.666	7.118	7.937
376	0.543	1.155	1.383	1.4	2.002	2.847	3.568	3.677	5.565	7.032	7.908
377	0.536	1.142	1.364	1.395	1.985	2.803	3.52	3.63	5.479	6.935	7.845
378	0.531	1.13	1.346	1.387	1.959	2.767	3.459	3.602	5.393	6.86	7.846
379	0.532	1.12	1.34	1.378	1.936	2.733	3.407	3.576	5.312	6.761	7.785
380	0.524	1.099	1.314	1.373	1.919	2.705	3.364	3.554	5.251	6.684	7.774
381	0.52	1.098	1.314	1.368	1.905	2.692	3.343	3.542	5.204	6.651	7.758
382	0.52	1.077	1.311	1.361	1.912	2.683	3.302	3.53	5.152	6.637	7.741
383	0.506	1.07	1.304	1.369	1.914	2.678	3.295	3.536	5.115	6.606	7.754
384	0.511	1.046	1.305	1.359	1.907	2.679	3.271	3.537	5.088	6.577	7.794
385	0.496	1.032	1.305	1.349	1.9	2.668	3.249	3.539	5.07	6.575	7.826
386	0.495	1.022	1.294	1.359	1.909	2.646	3.233	3.537	5.025	6.556	7.857
387	0.491	1.009	1.292	1.345	1.887	2.639	3.222	3.547	4.993	6.541	7.857
388	0.487	0.994	1.277	1.339	1.88	2.61	3.185	3.524	4.959	6.505	7.85
389	0.484	0.98	1.267	1.336	1.853	2.585	3.154	3.488	4.881	6.43	7.828
390	0.479	0.964	1.239	1.321	1.84	2.547	3.094	3.45	4.785	6.338	7.742
391	0.472	0.942	1.227	1.3	1.808	2.5	3.045	3.388	4.685	6.227	7.665
392	0.465	0.93	1.2	1.288	1.776	2.444	2.979	3.325	4.569	6.085	7.531
393	0.459	0.91	1.179	1.259	1.733	2.383	2.897	3.238	4.441	5.899	7.357
394	0.452	0.894	1.157	1.236	1.697	2.319	2.834	3.173	4.339	5.765	7.228
395	0.449	0.875	1.119	1.21	1.655	2.249	2.759	3.077	4.175	5.581	7.014
396	0.444	0.854	1.096	1.184	1.61	2.178	2.651	2.98	4.024	5.393	6.829
397	0.438	0.835	1.068	1.148	1.564	2.113	2.568	2.9	3.898	5.195	6.615
398	0.432	0.812	1.032	1.123	1.521	2.045	2.488	2.803	3.753	5.028	6.401
399	0.421	0.796	1.011	1.095	1.477	1.994	2.408	2.715	3.63	4.847	6.218
400	0.417	0.769	0.981	1.078	1.442	1.934	2.345	2.646	3.508	4.691	6.041
401	0.411	0.759	0.954	1.047	1.408	1.875	2.28	2.557	3.399	4.55	5.855
402	0.401	0.744	0.935	1.03	1.37	1.825	2.214	2.499	3.302	4.414	5.69
403	0.401	0.729	0.918	0.998	1.348	1.788	2.162	2.427	3.205	4.294	5.547
404	0.396	0.704	0.893	0.987	1.313	1.739	2.109	2.371	3.117	4.173	5.418
405	0.39	0.692	0.875	0.962	1.3	1.7	2.055	2.324	3.035	4.077	5.294

EXCLI Journal 2020;19:48-70 – ISSN 1611-2156
Received: October 03, 2019, accepted: December 19, 2019, published: January 03, 2020

Emission intensity											
**Wavelength (nm)**	**concentration of β-hydroxyisovalerylshikonin (M)**										
	A	*B*	*C*	*D*	*E*	*F*	*G*	*I*	*J*	*K*	*L*
	0.00	8.0x10^{-7}	1.6x10^{-6}	2.0x10^{-6}	2.8x10^{-6}	4.0x10^{-6}	4.8x10^{-6}	6.0x10^{-6}	8.0x10^{-6}	1.2x10^{-5}	1.6x10^{-5}
406	0.383	0.685	0.865	0.947	1.279	1.668	2.017	2.264	2.967	3.974	5.184
407	0.388	0.673	0.847	0.935	1.25	1.644	1.982	2.233	2.912	3.894	5.098
408	0.377	0.659	0.837	0.919	1.232	1.608	1.943	2.169	2.839	3.814	4.982
409	0.374	0.643	0.815	0.901	1.206	1.57	1.895	2.126	2.764	3.724	4.868
410	0.369	0.637	0.805	0.887	1.181	1.534	1.851	2.074	2.683	3.616	4.755
411	0.369	0.622	0.789	0.863	1.159	1.482	1.804	2.009	2.605	3.512	4.629
412	0.37	0.613	0.767	0.853	1.129	1.45	1.764	1.963	2.517	3.395	4.507
413	0.364	0.604	0.753	0.831	1.106	1.408	1.707	1.912	2.442	3.289	4.394
414	0.36	0.591	0.741	0.817	1.076	1.363	1.669	1.859	2.361	3.187	4.272
415	0.357	0.584	0.723	0.807	1.058	1.336	1.623	1.809	2.29	3.073	4.164
416	0.354	0.577	0.703	0.784	1.032	1.301	1.579	1.757	2.209	2.971	4.036
417	0.349	0.566	0.69	0.776	1.015	1.26	1.542	1.702	2.139	2.873	3.918
418	0.353	0.557	0.683	0.767	0.99	1.232	1.509	1.673	2.065	2.784	3.811
419	0.357	0.553	0.668	0.756	0.978	1.219	1.482	1.635	2.021	2.724	3.741
420	0.35	0.547	0.658	0.741	0.965	1.188	1.453	1.593	1.976	2.653	3.654
421	0.348	0.547	0.654	0.736	0.952	1.169	1.429	1.568	1.925	2.573	3.575
422	0.353	0.545	0.645	0.727	0.939	1.147	1.409	1.541	1.892	2.53	3.5
423	0.356	0.544	0.634	0.725	0.924	1.133	1.388	1.517	1.845	2.476	3.44
424	0.353	0.533	0.635	0.716	0.921	1.126	1.376	1.491	1.82	2.428	3.387
425	0.356	0.53	0.63	0.714	0.904	1.109	1.357	1.478	1.791	2.388	3.345
426	0.356	0.524	0.627	0.709	0.899	1.098	1.357	1.471	1.769	2.355	3.31
427	0.36	0.527	0.624	0.706	0.899	1.095	1.334	1.459	1.76	2.328	3.271
428	0.36	0.532	0.62	0.703	0.891	1.085	1.335	1.438	1.735	2.32	3.248
429	0.357	0.528	0.616	0.701	0.893	1.078	1.326	1.44	1.709	2.287	3.22
430	0.356	0.524	0.615	0.701	0.885	1.072	1.321	1.43	1.712	2.26	3.202
431	0.36	0.526	0.616	0.7	0.881	1.063	1.314	1.424	1.691	2.248	3.176
432	0.361	0.517	0.61	0.687	0.883	1.06	1.305	1.419	1.683	2.229	3.159
433	0.364	0.52	0.604	0.694	0.875	1.048	1.302	1.408	1.662	2.211	3.149
434	0.365	0.52	0.605	0.7	0.872	1.042	1.291	1.396	1.642	2.186	3.121
435	0.361	0.515	0.609	0.685	0.863	1.035	1.282	1.385	1.635	2.163	3.105
436	0.362	0.516	0.602	0.691	0.862	1.033	1.28	1.376	1.625	2.139	3.09
437	0.362	0.516	0.602	0.691	0.862	1.033	1.28	1.376	1.607	2.139	3.09
438	0.364	0.512	0.589	0.68	0.85	1.017	1.266	1.361	1.589	2.109	3.054

Emission intensity											
**Wavelength (nm)**	**concentration of β-hydroxyisovalerylshikonin (M)**										
	A	*B*	*C*	*D*	*E*	*F*	*G*	*I*	*J*	*K*	*L*
	0.00	$8.0x10^{-7}$	$1.6x10^{-6}$	$2.0x10^{-6}$	$2.8x10^{-6}$	$4.0x10^{-6}$	$4.8x10^{-6}$	$6.0x10^{-6}$	$8.0x10^{-6}$	$1.2x10^{-5}$	$1.6x10^{-5}$
439	0.358	0.517	0.589	0.683	0.844	1.011	1.248	1.354	1.577	2.087	3.033
440	0.363	0.506	0.587	0.683	0.836	1.001	1.247	1.342	1.56	2.07	3.015
441	0.359	0.507	0.581	0.677	0.834	0.997	1.246	1.336	1.558	2.056	2.995
442	0.352	0.502	0.582	0.676	0.827	0.994	1.242	1.333	1.538	2.044	2.99
443	0.362	0.507	0.573	0.67	0.821	0.993	1.228	1.325	1.536	2.028	2.976
444	0.351	0.504	0.571	0.675	0.823	0.978	1.229	1.325	1.534	2.022	2.974
445	0.355	0.5	0.572	0.669	0.818	0.98	1.226	1.322	1.528	2.023	2.966
446	0.35	0.492	0.572	0.671	0.809	0.982	1.228	1.316	1.526	2.027	2.966
447	0.347	0.493	0.575	0.665	0.817	0.978	1.223	1.316	1.528	2.021	2.971
448	0.346	0.495	0.572	0.677	0.815	0.974	1.213	1.325	1.53	2.03	2.974
449	0.35	0.498	0.569	0.675	0.812	0.982	1.221	1.33	1.532	2.034	2.992
450	0.344	0.496	0.574	0.67	0.812	0.983	1.221	1.326	1.535	2.045	2.997
451	0.343	0.488	0.574	0.676	0.809	0.983	1.226	1.334	1.543	2.047	3.024
452	0.339	0.486	0.572	0.676	0.813	0.981	1.224	1.333	1.554	2.064	3.048
453	0.342	0.492	0.571	0.682	0.81	0.983	1.231	1.341	1.558	2.087	3.055
454	0.335	0.484	0.573	0.681	0.812	0.99	1.234	1.353	1.563	2.091	3.069
455	0.334	0.488	0.572	0.678	0.812	0.991	1.24	1.359	1.579	2.109	3.094
456	0.335	0.487	0.572	0.679	0.812	0.999	1.244	1.366	1.586	2.12	3.12
457	0.324	0.477	0.572	0.679	0.815	1.006	1.238	1.374	1.591	2.138	3.136
458	0.327	0.478	0.575	0.685	0.818	1.003	1.25	1.38	1.608	2.16	3.157
459	0.32	0.479	0.567	0.69	0.822	1.01	1.247	1.384	1.607	2.169	3.18
460	0.321	0.476	0.569	0.686	0.816	1.01	1.247	1.386	1.624	2.181	3.209

EXCLI Journal 2020;19:48-70 – ISSN 1611-2156
Received: October 03, 2019, accepted: December 19, 2019, published: January 03, 2020

Raw tables concerning **Supplementary Figure 1**. Absorption spectra of of α-methylbutyrylshikon (***1***), acetylshikonin (***2***) and *β*-hydroxyisovalerylshikonin (***3***) (concentration of each was 8.00 x 10^{-5} M) before (blue line) and after addition of CT-DNA (0.00-1.73 x 10^{-4} M). Arrow shows the absorbance changes upon increasing concentration of CT-DNA.

Table 1: Absorption intensity in the wavelength range of 200-800 nm for absorption spectra of α-methylbutyrylshikonin fixed concentration (8.00 x 10^{-5} M), in the absence (*A*) and presence of increasing concentration of CT-DNA (*B-F*)

	Absorption intensity					
**Wavelength (nm)**	**concentration of CT-DNA (M)**					
	A	*B*	*C*	*D*	*E*	*F*
	0.00	2.66x10^{-5}	6.21x10^{-5}	7.98x10^{-5}	1.33x10^{-4}	1.73x10^{-4}
800	0.002551	0.000885	0.002165	0.001607	0.003029	0.002269
799	0.002167	0.001441	0.00228	0.001717	0.003153	0.00253
798	0.002208	0.001049	0.002076	0.001767	0.003084	0.002806
797	0.002654	0.001578	0.002668	0.002096	0.003196	0.002764
796	0.002415	0.001231	0.002456	0.002071	0.003332	0.002894
795	0.002219	0.001291	0.002086	0.001743	0.003079	0.002813
794	0.001917	0.001256	0.002209	0.001747	0.002898	0.002585
793	0.002243	0.001566	0.002561	0.001844	0.002944	0.002613
792	0.002448	0.00167	0.002694	0.002242	0.003617	0.002998
791	0.002166	0.001086	0.002283	0.001848	0.003163	0.002663
790	0.002589	0.001364	0.002155	0.001947	0.003178	0.002988
789	0.002018	0.001236	0.002287	0.001555	0.003189	0.002755
788	0.001945	0.001024	0.002133	0.001809	0.003001	0.002616
787	0.002159	0.00121	0.002099	0.001591	0.002966	0.002557
786	0.002273	0.001258	0.002454	0.001622	0.003189	0.002879
785	0.001906	0.001175	0.002331	0.001536	0.002862	0.002553
784	0.002477	0.001495	0.002583	0.001821	0.003255	0.002708
783	0.002006	0.001429	0.00244	0.00181	0.003338	0.002878
782	0.002293	0.00116	0.00209	0.001567	0.003021	0.002728
781	0.002353	0.001473	0.002624	0.001898	0.003349	0.002813
780	0.002336	0.001088	0.00214	0.001629	0.002997	0.002735
779	0.002161	0.001068	0.002274	0.001831	0.003137	0.002654
778	0.002174	0.001079	0.002098	0.001581	0.003241	0.002632
777	0.002355	0.001208	0.002349	0.001842	0.003118	0.002805
776	0.001872	0.001425	0.002346	0.001616	0.002948	0.002893
775	0.002478	0.001142	0.002472	0.001733	0.003211	0.00283
774	0.002123	0.001328	0.002131	0.001815	0.00305	0.002874
773	0.002301	0.001377	0.002178	0.001776	0.003139	0.002964
772	0.002318	0.001115	0.002174	0.001893	0.003423	0.002833
771	0.001998	0.001143	0.002252	0.00178	0.003146	0.002642
770	0.001868	0.000959	0.002096	0.001333	0.002852	0.002708
769	0.002397	0.001389	0.002458	0.001983	0.00317	0.003014
768	0.002337	0.001417	0.002405	0.001823	0.003134	0.002804
767	0.002092	0.00139	0.002232	0.001918	0.003255	0.002698
766	0.002259	0.001242	0.002401	0.001999	0.00333	0.003126
765	0.002306	0.001388	0.00221	0.001751	0.003447	0.002626
764	0.002287	0.001399	0.00261	0.001905	0.003364	0.002888
763	0.002204	0.001222	0.002421	0.001964	0.003331	0.003081
762	0.002171	0.001121	0.002156	0.001845	0.003171	0.002592
761	0.002332	0.001338	0.002703	0.001913	0.003314	0.002997

EXCLI Journal 2020;19:48-70 – ISSN 1611-2156
Received: October 03, 2019, accepted: December 19, 2019, published: January 03, 2020

Absorption intensity						
**Wavelength (nm)**	**concentration of CT-DNA (M)**					
	A	*B*	*C*	*D*	*E*	*F*
	0.00	$2.66x10^{-5}$	$6.21x10^{-5}$	$7.98x10^{-5}$	$1.33x10^{-4}$	$1.73x10^{-4}$
760	0.002087	0.001272	0.002302	0.001984	0.003405	0.002897
759	0.002184	0.001567	0.002582	0.0018	0.003458	0.002921
758	0.00219	0.001346	0.002338	0.001728	0.003262	0.003045
757	0.002343	0.001221	0.002503	0.002106	0.003356	0.003018
756	0.002531	0.001531	0.002458	0.002104	0.003595	0.002988
755	0.00231	0.001483	0.002473	0.002022	0.003612	0.003052
754	0.002125	0.001502	0.002563	0.002019	0.003518	0.003065
753	0.002236	0.001222	0.002507	0.001884	0.003468	0.002935
752	0.002273	0.001285	0.002541	0.001864	0.003444	0.002913
751	0.002218	0.001179	0.002437	0.002	0.003402	0.003081
750	0.001984	0.001077	0.002439	0.001731	0.003363	0.002861
749	0.002265	0.001529	0.002455	0.001945	0.003553	0.003064
748	0.00227	0.001345	0.002687	0.001974	0.003587	0.003081
747	0.002273	0.001126	0.00264	0.001946	0.003377	0.003131
746	0.002223	0.00122	0.002463	0.001962	0.003343	0.002913
745	0.002396	0.00114	0.002562	0.002068	0.003498	0.003105
744	0.002087	0.001216	0.002388	0.001879	0.003381	0.003053
743	0.002162	0.001404	0.002565	0.002104	0.003368	0.003123
742	0.002181	0.001396	0.002565	0.002055	0.00351	0.003239
741	0.001962	0.001128	0.002385	0.001846	0.003436	0.002991
740	0.002195	0.001216	0.002356	0.00198	0.003318	0.003164
739	0.002355	0.001297	0.002594	0.002238	0.003662	0.003332
738	0.002207	0.001524	0.002534	0.002154	0.003549	0.003324
737	0.002371	0.001415	0.002606	0.002258	0.003727	0.003399
736	0.00239	0.00143	0.002733	0.002222	0.00361	0.003384
735	0.002577	0.001524	0.002821	0.002252	0.003904	0.003558
734	0.002454	0.001573	0.002628	0.002251	0.003741	0.003533
733	0.002473	0.001836	0.002951	0.002541	0.004101	0.003639
732	0.002762	0.001889	0.003069	0.002574	0.004044	0.003764
731	0.002728	0.001866	0.003019	0.00269	0.003968	0.00384
730	0.002624	0.001678	0.002934	0.002378	0.003955	0.003727
729	0.002905	0.002038	0.003193	0.002819	0.004294	0.004025
728	0.002837	0.001987	0.003191	0.002733	0.0041	0.003949
727	0.002895	0.001979	0.003085	0.002817	0.004091	0.003894
726	0.002826	0.001984	0.003158	0.002774	0.004265	0.00403
725	0.002978	0.00222	0.003342	0.003045	0.00439	0.004207
724	0.003027	0.002188	0.003163	0.003	0.004289	0.004182
723	0.002959	0.002239	0.003402	0.003033	0.004352	0.004283
722	0.003069	0.002324	0.003523	0.003034	0.00435	0.004168
721	0.00314	0.002158	0.003348	0.00312	0.004431	0.004269
720	0.003086	0.002318	0.003484	0.002983	0.004379	0.004184
719	0.003243	0.002204	0.00349	0.003135	0.004462	0.00429
718	0.003281	0.00227	0.003478	0.00321	0.004506	0.004357
717	0.003217	0.002376	0.003562	0.003258	0.004575	0.004425
716	0.003234	0.002477	0.003595	0.003184	0.004461	0.004327
715	0.003445	0.002497	0.003751	0.003501	0.004743	0.004595
714	0.00329	0.002527	0.003708	0.003455	0.004696	0.004668
713	0.003297	0.0026	0.003751	0.003195	0.004749	0.00465

EXCLI Journal 2020;19:48-70 – ISSN 1611-2156
Received: October 03, 2019, accepted: December 19, 2019, published: January 03, 2020

Absorption intensity						
**Wavelength (nm)**	**concentration of CT-DNA (M)**					
	A	*B*	*C*	*D*	*E*	*F*
	0.00	2.66x10^{-5}	6.21x10^{-5}	7.98x10^{-5}	1.33x10^{-4}	1.73x10^{-4}
712	0.003337	0.002638	0.003842	0.003425	0.004833	0.004672
711	0.003426	0.002723	0.003961	0.003675	0.005021	0.004909
710	0.003598	0.002851	0.003994	0.003803	0.005081	0.005043
709	0.003369	0.002483	0.00385	0.003547	0.004997	0.004865
708	0.003421	0.002732	0.003911	0.003745	0.004849	0.004923
707	0.003667	0.00285	0.004118	0.003957	0.005157	0.0052
706	0.003775	0.002908	0.004144	0.003957	0.005296	0.005306
705	0.003602	0.003016	0.004063	0.003888	0.005219	0.00517
704	0.003757	0.00297	0.004219	0.003894	0.005293	0.005129
703	0.003882	0.003037	0.004282	0.004093	0.005226	0.005298
702	0.003848	0.00307	0.004237	0.004079	0.005232	0.005312
701	0.003983	0.003041	0.004351	0.004108	0.005177	0.00536
700	0.003896	0.003208	0.004406	0.004107	0.005463	0.005437
699	0.003889	0.003046	0.004422	0.004129	0.00536	0.005371
698	0.003943	0.003296	0.004609	0.004324	0.005471	0.00541
697	0.003854	0.003165	0.004336	0.004077	0.005354	0.005395
696	0.004031	0.003123	0.004374	0.004303	0.005455	0.005606
695	0.003821	0.003299	0.004439	0.004226	0.005551	0.005427
694	0.004113	0.003593	0.004657	0.004539	0.005797	0.005713
693	0.004094	0.003451	0.004862	0.004282	0.005775	0.005757
692	0.004083	0.003412	0.004623	0.004343	0.005712	0.005794
691	0.004051	0.00369	0.00481	0.004655	0.005853	0.005899
690	0.004172	0.003592	0.0049	0.004688	0.00588	0.006037
689	0.004326	0.003531	0.004826	0.0046	0.005994	0.006123
688	0.004373	0.003671	0.005035	0.004739	0.006044	0.0062
687	0.004431	0.003709	0.005038	0.0048	0.006028	0.006176
686	0.00451	0.003808	0.005039	0.004794	0.006062	0.006429
685	0.004509	0.003858	0.005081	0.004957	0.006188	0.006284
684	0.004599	0.003854	0.005086	0.00511	0.006295	0.006427
683	0.004652	0.004062	0.005182	0.005206	0.006397	0.006578
682	0.004752	0.004138	0.005426	0.005102	0.006611	0.006547
681	0.0047	0.004089	0.005427	0.005233	0.006438	0.006766
680	0.004593	0.004099	0.005423	0.005164	0.006275	0.006568
679	0.004804	0.004192	0.005528	0.005468	0.006731	0.006893
678	0.004792	0.00405	0.005611	0.005442	0.006558	0.006753
677	0.004747	0.004211	0.005557	0.005422	0.006658	0.006957
676	0.004684	0.004239	0.005743	0.005374	0.006697	0.006955
675	0.004816	0.004329	0.005678	0.005515	0.006859	0.006876
674	0.005004	0.004558	0.005877	0.005725	0.007162	0.007168
673	0.005056	0.004532	0.005955	0.005721	0.006908	0.007203
672	0.005038	0.004415	0.005786	0.005653	0.006919	0.007229
671	0.005032	0.004645	0.006072	0.005819	0.007146	0.007471
670	0.005205	0.004782	0.006013	0.006124	0.007313	0.007538
669	0.005238	0.004845	0.006102	0.006089	0.007315	0.007742
668	0.005316	0.00481	0.006156	0.006167	0.007449	0.007719
667	0.005394	0.004852	0.00639	0.006163	0.007476	0.007818
666	0.00537	0.005113	0.006407	0.006339	0.007487	0.007892
665	0.00553	0.005042	0.00649	0.006341	0.007661	0.008027

EXCLI Journal 2020;19:48-70 – ISSN 1611-2156
Received: October 03, 2019, accepted: December 19, 2019, published: January 03, 2020

Absorption intensity						
**Wavelength (nm)**	**concentration of CT-DNA (M)**					
	A	*B*	*C*	*D*	*E*	*F*
	0.00	2.66×10^{-5}	6.21×10^{-5}	7.98×10^{-5}	1.33×10^{-4}	1.73×10^{-4}
664	0.005584	0.005087	0.006487	0.006393	0.007771	0.008142
663	0.005562	0.005231	0.006587	0.006512	0.007863	0.008185
662	0.005831	0.005345	0.006687	0.006816	0.008014	0.008468
661	0.005619	0.005232	0.00676	0.006619	0.007885	0.008336
660	0.005882	0.005327	0.006738	0.00681	0.008175	0.008466
659	0.005873	0.005362	0.006882	0.006973	0.008149	0.008575
658	0.005879	0.005494	0.006925	0.00688	0.008162	0.00857
657	0.006062	0.005642	0.007157	0.007155	0.008477	0.008862
656	0.006013	0.005667	0.007084	0.007271	0.008351	0.008846
655	0.00617	0.005779	0.007327	0.007375	0.008421	0.008952
654	0.006092	0.00591	0.007329	0.007493	0.008626	0.009193
653	0.006302	0.005975	0.007463	0.007551	0.008738	0.009334
652	0.006337	0.00619	0.007705	0.007664	0.008906	0.009448
651	0.006351	0.006125	0.007525	0.007757	0.009025	0.009355
650	0.006484	0.006316	0.007713	0.007891	0.009094	0.009694
649	0.006484	0.006293	0.007654	0.007903	0.009007	0.009688
648	0.006604	0.006493	0.008054	0.008191	0.009248	0.009971
647	0.006533	0.006423	0.007955	0.008139	0.009225	0.009865
646	0.006771	0.006583	0.008095	0.008231	0.009414	0.010051
645	0.006754	0.006548	0.008105	0.008397	0.009459	0.010221
644	0.006983	0.006869	0.00824	0.008634	0.009717	0.010373
643	0.007217	0.006997	0.008477	0.008718	0.009829	0.010629
642	0.007055	0.006927	0.008437	0.00873	0.009737	0.010531
641	0.007167	0.007006	0.008616	0.00885	0.009867	0.010669
640	0.007086	0.007146	0.008701	0.008978	0.009964	0.010732
639	0.007225	0.007201	0.008712	0.009034	0.010142	0.010957
638	0.007334	0.007251	0.008781	0.00908	0.010253	0.011003
637	0.007176	0.007229	0.008971	0.009224	0.010321	0.011106
636	0.007473	0.007498	0.00899	0.009342	0.010516	0.011231
635	0.007518	0.0075	0.009266	0.009432	0.010669	0.011465
634	0.00759	0.007495	0.009206	0.009491	0.01071	0.011484
633	0.007556	0.007676	0.009317	0.009618	0.010796	0.011536
632	0.007957	0.007951	0.009501	0.009823	0.011032	0.011941
631	0.007742	0.007909	0.009448	0.00996	0.011121	0.011958
630	0.00785	0.008111	0.009784	0.010154	0.011298	0.012179
629	0.007973	0.00825	0.009707	0.010179	0.011311	0.012203
628	0.008139	0.008158	0.00978	0.010343	0.011432	0.012486
627	0.008341	0.008579	0.010202	0.010618	0.011724	0.012747
626	0.008363	0.00847	0.010323	0.010659	0.011815	0.012794
625	0.008216	0.008556	0.010297	0.010737	0.011823	0.012816
624	0.008632	0.008852	0.01049	0.011044	0.012152	0.013182
623	0.008607	0.008695	0.010581	0.011084	0.012228	0.013289
622	0.008877	0.008992	0.010758	0.011355	0.012392	0.013388
621	0.008806	0.008992	0.01067	0.011294	0.012337	0.013398
620	0.008879	0.009181	0.010899	0.011449	0.012503	0.013672
619	0.008984	0.009332	0.011007	0.011553	0.012548	0.013805
618	0.008908	0.009225	0.01105	0.011658	0.012634	0.013716
617	0.009115	0.009444	0.011232	0.011874	0.012898	0.013961

EXCLI Journal 2020;19:48-70 – ISSN 1611-2156
Received: October 03, 2019, accepted: December 19, 2019, published: January 03, 2020

Absorption intensity						
Wavelength (nm)	concentration of CT-DNA (M)					
	A	*B*	*C*	*D*	*E*	*F*
	0.00	$2.66x10^{-5}$	$6.21x10^{-5}$	$7.98x10^{-5}$	$1.33x10^{-4}$	$1.73x10^{-4}$
616	0.00929	0.009609	0.011346	0.011998	0.013106	0.014104
615	0.009203	0.009646	0.011445	0.01212	0.013142	0.014122
614	0.009429	0.009845	0.011602	0.012254	0.013455	0.014502
613	0.009369	0.009905	0.01176	0.012136	0.013399	0.014473
612	0.009594	0.009912	0.011918	0.012392	0.013628	0.014574
611	0.009696	0.01018	0.01209	0.012782	0.013774	0.014977
610	0.009836	0.010258	0.01221	0.012809	0.013984	0.01506
609	0.009915	0.010474	0.012318	0.013051	0.014156	0.015301
608	0.009898	0.010505	0.01247	0.013046	0.014137	0.015402
607	0.00996	0.010653	0.012365	0.013124	0.014283	0.015424
606	0.010119	0.010774	0.012728	0.013404	0.014503	0.015725
605	0.010353	0.011054	0.012941	0.013602	0.014706	0.016039
604	0.010478	0.011165	0.013053	0.013655	0.014801	0.016148
603	0.010626	0.011188	0.013115	0.013794	0.014958	0.016222
602	0.010686	0.011319	0.01317	0.013962	0.015052	0.016415
601	0.010778	0.011502	0.013462	0.01419	0.015258	0.016583
600	0.010792	0.011494	0.013437	0.014291	0.015331	0.016601
599	0.011051	0.011795	0.01372	0.014535	0.015601	0.016948
598	0.011008	0.011652	0.013607	0.014534	0.015572	0.016909
597	0.011165	0.011971	0.013972	0.0148	0.015797	0.01713
596	0.011125	0.012105	0.013981	0.014904	0.016076	0.017194
595	0.011267	0.012189	0.014317	0.014998	0.016113	0.017388
594	0.011442	0.012377	0.01441	0.015232	0.016388	0.017584
593	0.01162	0.012629	0.014516	0.015559	0.016653	0.017859
592	0.01174	0.012675	0.014615	0.015576	0.016624	0.017943
591	0.011527	0.012714	0.014719	0.015754	0.016758	0.018045
590	0.01189	0.012915	0.014922	0.015877	0.01699	0.018332
589	0.012064	0.013167	0.015088	0.016007	0.017178	0.018584
588	0.012176	0.013191	0.015346	0.016275	0.017341	0.018562
587	0.011764	0.013026	0.014963	0.015917	0.017002	0.018368
586	0.012144	0.013165	0.01531	0.016222	0.017173	0.018785
585	0.012426	0.013568	0.015596	0.016667	0.01758	0.019081
584	0.012631	0.013762	0.01596	0.016932	0.017838	0.019443
583	0.012796	0.013979	0.015976	0.01693	0.01808	0.019529
582	0.012894	0.014037	0.016236	0.017245	0.018315	0.019746
581	0.012928	0.014173	0.016338	0.017425	0.018282	0.01986
580	0.013049	0.014238	0.016332	0.017505	0.018394	0.019987
579	0.01305	0.014265	0.01644	0.017569	0.018514	0.019937
578	0.013161	0.014527	0.016721	0.017751	0.018716	0.020225
577	0.013338	0.014775	0.0169	0.018004	0.018929	0.020379
576	0.013413	0.014837	0.016935	0.018104	0.019147	0.02045
575	0.013568	0.014982	0.017059	0.018198	0.019287	0.020659
574	0.013768	0.015194	0.017274	0.018438	0.019464	0.021027
573	0.013695	0.015214	0.017315	0.018438	0.019465	0.020989
572	0.013814	0.015321	0.017554	0.018527	0.019629	0.021211
571	0.013899	0.015411	0.017726	0.018835	0.019841	0.021351
570	0.014055	0.015543	0.017781	0.018764	0.019992	0.021467
569	0.014036	0.015754	0.017876	0.018967	0.020057	0.021628

EXCLI Journal 2020;19:48-70 – ISSN 1611-2156
Received: October 03, 2019, accepted: December 19, 2019, published: January 03, 2020

Absorption intensity						
Wavelength (nm)	concentration of CT-DNA (M)					
	A	B	C	D	E	F
	0.00	2.66x10^{-5}	6.21x10^{-5}	7.98x10^{-5}	1.33x10^{-4}	1.73x10^{-4}
568	0.014246	0.015804	0.018005	0.019212	0.020212	0.021774
567	0.014318	0.015992	0.018172	0.019302	0.020446	0.021931
566	0.014413	0.016057	0.018255	0.019444	0.02054	0.022055
565	0.014475	0.016239	0.018397	0.019551	0.020612	0.022002
564	0.014555	0.016264	0.018474	0.019692	0.020691	0.022258
563	0.01478	0.016306	0.018501	0.019881	0.020797	0.022462
562	0.014846	0.01627	0.018704	0.019942	0.020925	0.022488
561	0.014978	0.016624	0.018785	0.019977	0.021173	0.022633
560	0.015193	0.016717	0.018881	0.020222	0.021146	0.022708
559	0.014952	0.016655	0.018946	0.020252	0.021192	0.02268
558	0.015216	0.017039	0.019155	0.020412	0.021413	0.022967
557	0.015225	0.016773	0.019066	0.020412	0.02133	0.02301
556	0.01509	0.016758	0.019044	0.020401	0.021261	0.022864
555	0.015005	0.016766	0.019037	0.020309	0.021243	0.02285
554	0.015336	0.016972	0.018995	0.020427	0.021332	0.022824
553	0.015507	0.017114	0.019465	0.020794	0.02166	0.023175
552	0.015653	0.017445	0.019821	0.021084	0.02209	0.02352
551	0.015694	0.017507	0.01976	0.02112	0.02197	0.023499
550	0.015933	0.017685	0.020044	0.02137	0.022296	0.023879
549	0.015992	0.017676	0.020032	0.021385	0.022504	0.023931
548	0.015994	0.017824	0.020077	0.021451	0.022472	0.024038
547	0.016107	0.017825	0.02021	0.021446	0.022572	0.024113
546	0.016156	0.018015	0.020402	0.021637	0.02274	0.024203
545	0.016394	0.018203	0.02042	0.021864	0.022935	0.024436
544	0.0165	0.018356	0.020652	0.022107	0.022979	0.024595
543	0.016644	0.018444	0.020777	0.022228	0.023176	0.024783
542	0.016688	0.018446	0.020822	0.022177	0.02318	0.024792
541	0.016598	0.018675	0.020909	0.02227	0.023205	0.024828
540	0.016974	0.018924	0.021196	0.022544	0.023587	0.025114
539	0.016974	0.018939	0.021159	0.02268	0.023608	0.025188
538	0.017089	0.018974	0.02118	0.022646	0.023528	0.025228
537	0.017296	0.019179	0.02135	0.022801	0.023743	0.025131
536	0.017269	0.019029	0.02148	0.022854	0.023718	0.025359
535	0.017268	0.019207	0.021436	0.022979	0.023855	0.025524
534	0.01749	0.019524	0.021696	0.023175	0.024045	0.025636
533	0.017492	0.019482	0.021703	0.023268	0.024094	0.025655
532	0.017634	0.019432	0.02186	0.02341	0.024193	0.025643
531	0.017664	0.01973	0.021962	0.023417	0.024383	0.025976
530	0.01765	0.019667	0.021953	0.023433	0.024403	0.025949
529	0.017861	0.019794	0.021991	0.023597	0.024392	0.02605
528	0.017982	0.019846	0.022199	0.023717	0.024581	0.026106
527	0.01796	0.019902	0.02227	0.023679	0.024609	0.026151
526	0.018004	0.019997	0.022359	0.023774	0.024708	0.026263
525	0.018286	0.02039	0.022553	0.023999	0.024882	0.026501
524	0.018221	0.020307	0.022444	0.023984	0.024942	0.026478
523	0.01834	0.020346	0.022609	0.024043	0.025075	0.026614
522	0.018411	0.020339	0.022615	0.024125	0.025083	0.026556
521	0.018435	0.02057	0.022681	0.024298	0.025026	0.02667

EXCLI Journal 2020;19:48-70 – ISSN 1611-2156
Received: October 03, 2019, accepted: December 19, 2019, published: January 03, 2020

Absorption intensity						
Wavelength (nm)	concentration of CT-DNA (M)					
	A	B	C	D	E	F
	0.00	2.66x10^{-5}	6.21x10^{-5}	7.98x10^{-5}	1.33x10^{-4}	1.73x10^{-4}
520	0.018428	0.020432	0.022678	0.02414	0.025035	0.026426
519	0.018584	0.020651	0.022638	0.024075	0.024871	0.026438
518	0.018686	0.020583	0.022608	0.024061	0.02505	0.02659
517	0.018425	0.020286	0.022485	0.023967	0.024776	0.026418
516	0.018239	0.020179	0.022382	0.023979	0.024855	0.026325
515	0.018484	0.020455	0.022636	0.024243	0.02502	0.026565
514	0.018591	0.020432	0.022475	0.024138	0.024763	0.026466
513	0.018533	0.020558	0.022661	0.024274	0.025115	0.026605
512	0.018646	0.020493	0.02266	0.024319	0.025031	0.026699
511	0.018745	0.0202	0.02276	0.02428	0.025194	0.026735
510	0.018804	0.020638	0.022848	0.024695	0.025048	0.026737
509	0.018896	0.020799	0.023128	0.024557	0.025402	0.026912
508	0.018907	0.020798	0.022969	0.024554	0.025461	0.026843
507	0.018878	0.020784	0.022926	0.024515	0.025351	0.026878
506	0.018866	0.020749	0.023006	0.024481	0.02537	0.026898
505	0.018934	0.020719	0.022962	0.024524	0.025318	0.026862
504	0.019018	0.020935	0.022996	0.024555	0.025459	0.027052
503	0.019032	0.020968	0.023067	0.024714	0.025482	0.02703
502	0.019194	0.021122	0.023208	0.024793	0.025616	0.027087
501	0.019193	0.021163	0.023187	0.02474	0.02558	0.027137
500	0.01949	0.021183	0.023312	0.024861	0.025667	0.027365
499	0.01938	0.021279	0.023578	0.024808	0.02564	0.027295
498	0.019307	0.021161	0.023249	0.024821	0.025736	0.027359
497	0.019352	0.021256	0.023314	0.024811	0.025407	0.027177
496	0.019377	0.021199	0.023147	0.024785	0.025561	0.027106
495	0.019248	0.020972	0.022983	0.024619	0.025298	0.026976
494	0.019376	0.021095	0.023104	0.0247	0.025525	0.027064
493	0.019251	0.021043	0.02311	0.024598	0.025356	0.02701
492	0.019476	0.021208	0.023155	0.024843	0.025527	0.027132
491	0.019475	0.021065	0.02329	0.02469	0.025517	0.027107
490	0.019556	0.021253	0.023196	0.02474	0.025442	0.027095
489	0.019758	0.021523	0.023386	0.024884	0.025685	0.027139
488	0.019729	0.021454	0.023536	0.024971	0.025703	0.027102
487	0.019769	0.021464	0.023577	0.025012	0.025925	0.027397
486	0.019742	0.021409	0.023377	0.025	0.025901	0.02742
485	0.019798	0.021531	0.023438	0.02512	0.025865	0.027429
484	0.01991	0.021527	0.023515	0.024977	0.025847	0.027409
483	0.019923	0.021479	0.02353	0.025118	0.025954	0.027532
482	0.019788	0.021431	0.023404	0.024863	0.025665	0.027469
481	0.019837	0.021469	0.023491	0.024885	0.025751	0.027503
480	0.019972	0.021391	0.023524	0.025038	0.025844	0.027357
479	0.019872	0.021457	0.023382	0.024915	0.025675	0.027426
478	0.019912	0.021517	0.023509	0.024998	0.025676	0.02753
477	0.019888	0.021383	0.023194	0.024886	0.025604	0.027264
476	0.019877	0.021423	0.023418	0.024837	0.025623	0.027231
475	0.019815	0.021367	0.023294	0.024818	0.025539	0.027174
474	0.019763	0.021131	0.023227	0.024681	0.025495	0.026942
473	0.019933	0.021281	0.022943	0.02459	0.025301	0.026692

EXCLI Journal 2020;19:48-70 – ISSN 1611-2156
Received: October 03, 2019, accepted: December 19, 2019, published: January 03, 2020

Absorption intensity						
Wavelength (nm)	concentration of CT-DNA (M)					
	A	*B*	*C*	*D*	*E*	*F*
	0.00	$2.66x10^{-5}$	$6.21x10^{-5}$	$7.98x10^{-5}$	$1.33x10^{-4}$	$1.73x10^{-4}$
472	0.019457	0.020831	0.022856	0.024266	0.024942	0.026618
471	0.019217	0.020716	0.022809	0.024204	0.024967	0.026671
470	0.019318	0.02081	0.022711	0.02426	0.024975	0.026642
469	0.019161	0.020662	0.022731	0.024249	0.024939	0.026415
468	0.019479	0.020899	0.022808	0.02429	0.025124	0.026746
467	0.019643	0.020986	0.022986	0.02449	0.025317	0.026765
466	0.019491	0.020914	0.022776	0.024244	0.025121	0.026844
465	0.019538	0.020881	0.022892	0.024249	0.02526	0.026867
464	0.019332	0.020826	0.022828	0.024174	0.025038	0.026654
463	0.019444	0.020787	0.022792	0.024242	0.025052	0.026807
462	0.01958	0.020821	0.022755	0.024101	0.02504	0.026801
461	0.019546	0.020775	0.022664	0.024167	0.025058	0.026625
460	0.019397	0.020709	0.022573	0.024101	0.024867	0.026676
459	0.019431	0.020704	0.022616	0.024066	0.024968	0.026531
458	0.019443	0.020651	0.022461	0.023994	0.024821	0.026496
457	0.019328	0.02053	0.022465	0.023967	0.024674	0.026409
456	0.019419	0.020664	0.022433	0.024125	0.024857	0.026488
455	0.019115	0.020333	0.022309	0.023702	0.024695	0.026261
454	0.019214	0.020493	0.022211	0.023864	0.024631	0.026269
453	0.019217	0.020395	0.022274	0.023762	0.024625	0.026331
452	0.019109	0.020236	0.022195	0.023753	0.024546	0.026174
451	0.018923	0.020122	0.021932	0.023582	0.02436	0.025969
450	0.019052	0.020187	0.022219	0.023507	0.024502	0.026204
449	0.018898	0.020243	0.021882	0.023418	0.024373	0.025957
448	0.019084	0.020128	0.022205	0.023558	0.024538	0.026151
447	0.018885	0.020069	0.021861	0.023512	0.024434	0.02591
446	0.018929	0.020044	0.022088	0.023367	0.024374	0.026087
445	0.018843	0.020045	0.021836	0.023382	0.024302	0.025981
444	0.018784	0.020036	0.021928	0.023419	0.02446	0.025979
443	0.018861	0.019877	0.021799	0.02329	0.024145	0.025963
442	0.018827	0.019687	0.021788	0.023178	0.024103	0.025914
441	0.018789	0.019985	0.021798	0.023134	0.02412	0.025928
440	0.018814	0.019894	0.021753	0.02327	0.024074	0.025766
439	0.018632	0.019734	0.021579	0.022989	0.023954	0.025803
438	0.018537	0.019646	0.021537	0.023027	0.024019	0.025814
437	0.018696	0.019747	0.021577	0.023203	0.024149	0.025924
436	0.018551	0.0196	0.021602	0.023056	0.023967	0.025724
435	0.01858	0.019551	0.021542	0.022997	0.023755	0.025765
434	0.018338	0.019506	0.021353	0.022877	0.023935	0.025527
433	0.018373	0.019439	0.021418	0.022987	0.023678	0.025566
432	0.018488	0.019402	0.021344	0.022861	0.023719	0.025446
431	0.01857	0.019527	0.021402	0.022891	0.023839	0.025643
430	0.018306	0.019344	0.021213	0.022715	0.023583	0.025504
429	0.018239	0.019329	0.02112	0.0227	0.023605	0.0254
428	0.018313	0.019356	0.021287	0.022688	0.023667	0.025556
427	0.018171	0.019156	0.021223	0.022609	0.023724	0.025483
426	0.018333	0.019229	0.021232	0.022613	0.023634	0.025577
425	0.018108	0.019163	0.021159	0.022663	0.023714	0.025366

EXCLI Journal 2020;19:48-70 – ISSN 1611-2156
Received: October 03, 2019, accepted: December 19, 2019, published: January 03, 2020

Absorption intensity						
Wavelength (nm)	concentration of CT-DNA (M)					
	A	*B*	*C*	*D*	*E*	*F*
	0.00	2.66x10^{-5}	6.21x10^{-5}	7.98x10^{-5}	1.33x10^{-4}	1.73x10^{-4}
424	0.017981	0.018872	0.020961	0.022379	0.02373	0.025365
423	0.01827	0.019091	0.021118	0.022565	0.023939	0.025412
422	0.018082	0.01901	0.021117	0.02248	0.023639	0.025474
421	0.018366	0.019234	0.021192	0.022492	0.023894	0.025613
420	0.018091	0.019059	0.021226	0.022655	0.023731	0.025484
419	0.018292	0.019111	0.02123	0.022625	0.023907	0.025632
418	0.01826	0.019115	0.020923	0.022494	0.023818	0.025559
417	0.018048	0.018875	0.021027	0.022525	0.023705	0.025475
416	0.018146	0.019128	0.021115	0.022422	0.023812	0.02553
415	0.018272	0.018952	0.021197	0.022574	0.023802	0.025508
414	0.018133	0.018951	0.021031	0.022515	0.023721	0.025457
413	0.018116	0.01905	0.02108	0.022419	0.023612	0.025414
412	0.01807	0.018938	0.020987	0.022603	0.023689	0.025495
411	0.017908	0.018777	0.020817	0.022264	0.023499	0.025319
410	0.01813	0.018815	0.021087	0.022605	0.023668	0.025394
409	0.018041	0.018949	0.021081	0.022481	0.023736	0.025401
408	0.017642	0.018673	0.02084	0.022295	0.023708	0.025098
407	0.017544	0.01864	0.020575	0.022153	0.02348	0.025084
406	0.017777	0.018652	0.02084	0.022401	0.023546	0.02533
405	0.017924	0.018884	0.02113	0.02254	0.023967	0.025753
404	0.018237	0.019285	0.021409	0.022853	0.023908	0.02601
403	0.018091	0.019107	0.021168	0.022603	0.023741	0.025766
402	0.01821	0.01922	0.021287	0.022792	0.02382	0.025895
401	0.018287	0.019271	0.021312	0.022645	0.023878	0.025867
400	0.018138	0.018954	0.021071	0.022546	0.023841	0.025743
399	0.018337	0.018989	0.021316	0.022624	0.023925	0.026015
398	0.018284	0.01933	0.021428	0.022865	0.023955	0.026036
397	0.018232	0.019134	0.02126	0.022672	0.023937	0.026083
396	0.018534	0.019463	0.021487	0.023202	0.024133	0.02621
395	0.018478	0.019296	0.021576	0.023143	0.024193	0.026267
394	0.01841	0.019501	0.02168	0.023131	0.024124	0.02626
393	0.018928	0.019591	0.021786	0.023167	0.024225	0.02663
392	0.018906	0.01962	0.021894	0.023236	0.024575	0.026557
391	0.018427	0.020086	0.022154	0.023618	0.024423	0.026867
390	0.018068	0.019048	0.021449	0.022722	0.024347	0.026009
389	0.018865	0.019644	0.022081	0.023578	0.02481	0.026692
388	0.018836	0.019708	0.022208	0.023558	0.024749	0.026851
387	0.018979	0.019685	0.021917	0.023497	0.02515	0.026948
386	0.018738	0.01973	0.022039	0.023572	0.025101	0.02698
385	0.019166	0.020158	0.022415	0.023652	0.025329	0.027329
384	0.018871	0.019829	0.022251	0.023459	0.025425	0.027028
383	0.019257	0.020476	0.022748	0.024051	0.025823	0.027576
382	0.019639	0.020528	0.022611	0.024203	0.025534	0.027857
381	0.019729	0.020198	0.022911	0.024259	0.025866	0.028233
380	0.019746	0.020584	0.023072	0.024352	0.025772	0.028004
379	0.019843	0.020712	0.02302	0.024328	0.025898	0.028161
378	0.020125	0.020747	0.023174	0.024707	0.02586	0.028499
377	0.019972	0.020718	0.0231	0.024693	0.026082	0.028335

EXCLI Journal 2020;19:48-70 – ISSN 1611-2156
Received: October 03, 2019, accepted: December 19, 2019, published: January 03, 2020

Wavelength (nm)	***Absorption intensity***					
	**concentration of CT-DNA (M)**					
	A	*B*	*C*	*D*	*E*	*F*
	0.00	$2.66x10^{-5}$	$6.21x10^{-5}$	$7.98x10^{-5}$	$1.33x10^{-4}$	$1.73x10^{-4}$
376	0.0201	0.021119	0.023764	0.024807	0.025884	0.0284
375	0.020024	0.021134	0.023395	0.024881	0.026555	0.02826
374	0.020125	0.020793	0.023192	0.024793	0.026147	0.028463
373	0.020394	0.021217	0.023693	0.025164	0.026984	0.028852
372	0.020644	0.021257	0.024	0.025341	0.027651	0.028882
371	0.020257	0.021034	0.023999	0.025026	0.026638	0.028845
370	0.020488	0.021488	0.023941	0.025026	0.027118	0.028869
369	0.020598	0.021408	0.024007	0.025342	0.026739	0.029235
368	0.02134	0.022307	0.024559	0.02598	0.027731	0.029853
367	0.020786	0.021396	0.024228	0.025686	0.027432	0.029289
366	0.021309	0.022067	0.02472	0.026208	0.028048	0.029768
365	0.020968	0.022169	0.025004	0.026202	0.027827	0.030274
364	0.020704	0.022478	0.025171	0.02615	0.027917	0.029968
363	0.021311	0.022405	0.025097	0.02655	0.028322	0.030588
362	0.022712	0.022801	0.026234	0.027076	0.029124	0.031368
361	0.020025	0.020561	0.023639	0.025143	0.026528	0.028972
360	0.021934	0.022545	0.025361	0.026948	0.029224	0.03115
359	0.021886	0.022822	0.025537	0.027534	0.028603	0.031385
358	0.022594	0.023095	0.026023	0.027085	0.028947	0.032429
357	0.023652	0.024444	0.027248	0.028333	0.030477	0.032905
356	0.019993	0.020965	0.023947	0.024752	0.026832	0.02951
355	0.023273	0.023918	0.026937	0.028392	0.030085	0.032656
354	0.022644	0.023418	0.025814	0.028254	0.029901	0.032657
353	0.023062	0.023565	0.02637	0.028346	0.0303	0.032703
352	0.022512	0.02367	0.026883	0.02818	0.030649	0.032569
351	0.023789	0.025045	0.025919	0.02965	0.030511	0.032413
350	0.023923	0.023278	0.027295	0.028973	0.030816	0.034556
349	0.023883	0.025717	0.029122	0.029714	0.031667	0.036294
348	0.022764	0.024552	0.026947	0.029027	0.030474	0.034303
347	0.023971	0.02482	0.027155	0.02944	0.030596	0.034219
346	0.023781	0.02489	0.027948	0.029459	0.031163	0.035008
345	0.024224	0.025275	0.027969	0.030531	0.031777	0.03544
344	0.024292	0.025802	0.028433	0.030806	0.032333	0.035781
343	0.024733	0.025802	0.029255	0.030784	0.032266	0.035916
342	0.02448	0.025985	0.02866	0.030733	0.032151	0.036066
341	0.024591	0.025797	0.028691	0.031229	0.032431	0.036605
340	0.025277	0.026307	0.02934	0.031493	0.032767	0.036892
339	0.025001	0.02688	0.029667	0.031725	0.033216	0.03745
338	0.024823	0.0268	0.029452	0.031786	0.033414	0.037851
337	0.025259	0.026732	0.02995	0.031887	0.034281	0.037627
336	0.025737	0.027583	0.030264	0.032411	0.034343	0.038633
335	0.025632	0.027606	0.030991	0.033031	0.034244	0.039062
334	0.026211	0.02861	0.031042	0.033662	0.035394	0.039484
333	0.02643	0.028525	0.031037	0.033935	0.035785	0.039708
332	0.027347	0.029762	0.03219	0.034661	0.036113	0.04079
331	0.027192	0.029359	0.032415	0.034969	0.036082	0.041313
330	0.02797	0.029621	0.032547	0.035659	0.037398	0.041721
329	0.028647	0.030249	0.033472	0.035617	0.037773	0.042361

EXCLI Journal 2020;19:48-70 – ISSN 1611-2156
Received: October 03, 2019, accepted: December 19, 2019, published: January 03, 2020

Wavelength (nm)	Absorption intensity					
	concentration of CT-DNA (M)					
	A	*B*	*C*	*D*	*E*	*F*
	0.00	$2.66x10^{-5}$	$6.21x10^{-5}$	$7.98x10^{-5}$	$1.33x10^{-4}$	$1.73x10^{-4}$
328	0.028078	0.030206	0.033496	0.036116	0.037492	0.042583
327	0.029455	0.031737	0.03455	0.037402	0.03901	0.043704
326	0.0292	0.031244	0.034779	0.037158	0.039001	0.043632
325	0.029933	0.031995	0.035753	0.038732	0.039819	0.044436
324	0.029901	0.032278	0.036005	0.038724	0.040468	0.045567
323	0.030343	0.032984	0.035969	0.039105	0.041015	0.045813
322	0.03133	0.03337	0.036896	0.039772	0.041348	0.046454
321	0.032059	0.034024	0.037505	0.040266	0.042376	0.047739
320	0.032164	0.034693	0.038142	0.041126	0.042872	0.048269
319	0.03284	0.035366	0.038614	0.041933	0.043614	0.04905
318	0.033373	0.035557	0.039612	0.041988	0.04408	0.049849
317	0.033317	0.036054	0.040073	0.042915	0.044875	0.050332
316	0.034082	0.036637	0.040473	0.043821	0.045487	0.050919
315	0.0348	0.037393	0.041088	0.044334	0.046666	0.05231
314	0.034586	0.037901	0.041462	0.04508	0.047072	0.052886
313	0.035412	0.038486	0.042212	0.045851	0.047517	0.053363
312	0.036416	0.038881	0.043607	0.046895	0.049033	0.054932
311	0.036566	0.039429	0.043695	0.047478	0.049766	0.05534
310	0.037218	0.040183	0.04473	0.04851	0.050866	0.056816
309	0.03804	0.041372	0.04603	0.05014	0.052634	0.059055
308	0.039166	0.042514	0.047651	0.051471	0.054034	0.060638
307	0.039499	0.043579	0.048718	0.052743	0.055557	0.062312
306	0.040997	0.044796	0.05003	0.054812	0.057838	0.065184
305	0.041914	0.045672	0.052098	0.057207	0.060966	0.067611
304	0.042299	0.047094	0.053559	0.058309	0.063002	0.070458
303	0.043607	0.049009	0.055884	0.061652	0.066263	0.074477
302	0.045088	0.050892	0.059066	0.065104	0.071215	0.079152
301	0.046484	0.05311	0.062442	0.068726	0.076298	0.084829
300	0.047351	0.055452	0.065943	0.07366	0.082016	0.092245
299	0.048888	0.058475	0.070429	0.079797	0.089879	0.10085
298	0.05123	0.062592	0.077213	0.087353	0.09948	0.11141
297	0.053577	0.067276	0.08417	0.09572	0.110883	0.1244
296	0.056186	0.071801	0.09238	0.106072	0.124151	0.139223
295	0.058972	0.077477	0.102071	0.118011	0.139747	0.156642
294	0.061962	0.084204	0.113279	0.131453	0.157882	0.176597
293	0.065854	0.092051	0.125655	0.147307	0.178218	0.199237
292	0.069608	0.099827	0.139259	0.163964	0.199347	0.223965
291	0.0737	0.108201	0.153253	0.181623	0.223492	0.250641
290	0.078065	0.117949	0.16916	0.200393	0.248328	0.278752
289	0.08354	0.128516	0.185693	0.220769	0.275226	0.30847
288	0.088071	0.138341	0.202645	0.24126	0.302207	0.339075
287	0.09298	0.149595	0.220301	0.263353	0.331178	0.371257
286	0.098541	0.160521	0.238892	0.285705	0.360179	0.40393
285	0.10353	0.171811	0.25762	0.308794	0.391685	0.438595
284	0.109288	0.183842	0.276853	0.332878	0.42236	0.473179
283	0.11414	0.195407	0.296161	0.355911	0.452959	0.508138
282	0.120423	0.207391	0.315107	0.379086	0.483598	0.541531
281	0.125604	0.218956	0.333907	0.401747	0.512839	0.574753

EXCLI Journal 2020;19:48-70 – ISSN 1611-2156
Received: October 03, 2019, accepted: December 19, 2019, published: January 03, 2020

Absorption intensity						
Wavelength (nm)	concentration of CT-DNA (M)					
	A	B	C	D	E	F
	0.00	$2.66x10^{-5}$	$6.21x10^{-5}$	$7.98x10^{-5}$	$1.33x10^{-4}$	$1.73x10^{-4}$
280	0.13045	0.230145	0.351481	0.422959	0.541343	0.606319
279	0.135567	0.241141	0.368688	0.443706	0.568509	0.636435
278	0.141057	0.2526	0.386005	0.464094	0.595457	0.666755
277	0.145772	0.26349	0.403152	0.485587	0.623445	0.697961
276	0.150886	0.274441	0.420608	0.506904	0.650895	0.728301
275	0.155857	0.285189	0.438256	0.52752	0.678694	0.759475
274	0.160986	0.295726	0.455712	0.548906	0.70677	0.790087
273	0.166467	0.306377	0.472787	0.570299	0.734308	0.822026
272	0.171368	0.31712	0.489882	0.590557	0.76135	0.851595
271	0.176102	0.327559	0.506295	0.610718	0.787208	0.880595
270	0.180938	0.33701	0.521978	0.63024	0.81223	0.908003
269	0.185659	0.34595	0.536497	0.647396	0.835399	0.934284
268	0.189464	0.354647	0.550659	0.664329	0.857747	0.958926
267	0.194332	0.363332	0.564573	0.681122	0.879446	0.983298
266	0.198561	0.371203	0.57685	0.69712	0.900059	1.006053
265	0.202921	0.379058	0.589953	0.712704	0.920463	1.029032
264	0.206779	0.386511	0.602445	0.727667	0.93946	1.04986
263	0.211376	0.393676	0.613704	0.741496	0.957724	1.070079
262	0.215185	0.400406	0.624525	0.754884	0.973842	1.08882
261	0.218934	0.406295	0.633703	0.76574	0.987784	1.103421
260	0.222711	0.411732	0.64118	0.774352	0.999089	1.116426
259	0.226314	0.416103	0.647313	0.781567	1.007117	1.124967
258	0.229488	0.419774	0.651059	0.786183	1.012089	1.130438
257	0.232328	0.422036	0.653848	0.788076	1.014004	1.13245
256	0.234812	0.423855	0.654747	0.789	1.013677	1.131897
255	0.238083	0.425679	0.655001	0.788696	1.011225	1.127975
254	0.241137	0.42647	0.653705	0.785965	1.006953	1.122768
253	0.244639	0.42767	0.652086	0.782557	1.000016	1.114752
252	0.248136	0.428372	0.649067	0.777377	0.991128	1.103561
251	0.252728	0.42914	0.644976	0.770677	0.979946	1.089806
250	0.257788	0.430251	0.640678	0.762678	0.966453	1.074618
249	0.263125	0.431081	0.634778	0.753676	0.950979	1.056071
248	0.270379	0.432508	0.62979	0.745631	0.935462	1.038025
247	0.279839	0.436435	0.626624	0.737669	0.921175	1.019255
246	0.291594	0.442399	0.625229	0.732435	0.90806	1.003333
245	0.30754	0.452786	0.627668	0.730326	0.898861	0.990547
244	0.3289	0.467731	0.635936	0.734426	0.895647	0.983405
243	0.357426	0.490546	0.651383	0.746095	0.899465	0.984205
242	0.396898	0.523911	0.677469	0.768098	0.914397	0.995386
241	0.452016	0.57238	0.720622	0.807257	0.94583	1.023943
240	0.526939	0.642561	0.784356	0.867822	0.999194	1.074354
239	0.632377	0.742479	0.87907	0.960216	1.085064	1.157449
238	0.775372	0.880095	1.012903	1.09176	1.210516	1.281066
237	0.974409	1.073457	1.203649	1.280456	1.393494	1.462514
236	1.238456	1.33232	1.461021	1.537126	1.643549	1.712887
235	1.591617	1.682324	1.811269	1.886587	1.987496	2.056301
234	2.0486	2.134266	2.263277	2.338571	2.438615	2.503866
233	2.624259	2.706567	2.840475	2.921109	3.010827	3.076553

EXCLI Journal 2020;19:48-70 – ISSN 1611-2156
Received: October 03, 2019, accepted: December 19, 2019, published: January 03, 2020

Absorption intensity						
**Wavelength (nm)**	**concentration of CT-DNA (M)**					
	A	*B*	*C*	*D*	*E*	*F*
	0.00	$2.66x10^{-5}$	$6.21x10^{-5}$	$7.98x10^{-5}$	$1.33x10^{-4}$	$1.73x10^{-4}$
232	3.28683	3.370326	3.509165	3.5892	3.6813	3.746334
231	3.860149	3.954405	4.104144	4.174507	4.31553	4.303805
230	4.162339	4.283252	4.401635	4.515609	4.603689	4.588943
229	4.304737	4.506245	4.586563	4.690869	4.81039	4.80024
228	4.518479	4.621821	4.75509	4.874072	4.962668	4.962934
227	4.662835	4.766567	4.878093	4.886517	5.016925	5.005764
226	4.790772	4.872741	4.915738	5.318159	5.15745	4.973785
225	4.973036	4.923509	5.099469	5.210915	5.135952	5.312388
224	5.120623	5.352689	5.327682	5.18519	5.7079	5.503747
223	5.043415	5.105309	5.043218	5.413438	5.127712	5.413627
222	5.052739	5.222112	5.297204	5.434446	5.450387	5.285692
221	5.055247	5.191719	5.456385	6.155715	5.422987	5.423021
220	5.207856	5.540415	5.070227	5.117122	5.762094	5.887119
219	5.224245	5.020088	5.871762	5.349071	5.411436	6.525318
218	5.394481	5.378242	5.768124	6.031443	5.411509	5.633444
217	5.559766	5.535373	5.375468	10	5.644333	5.41039
216	5.307217	5.447431	5.371654	5.491049	10	5.565786
215	5.571307	6.446771	5.601484	5.332746	5.270574	10
214	4.967853	10	5.945644	10	5.343592	10
213	6.220434	10	5.697536	10	10	10
212	10	10	10	10	6.668649	10
211	10	6.035494	5.596108	10	10	5.734368
210	10	10	10	10	10	10
209	5.313125	5.467277	5.302106	5.363249	5.325335	5.927847
208	5.331749	10	5.568372	5.677462	5.647372	5.823696
207	5.107348	5.195361	5.848338	5.496029	5.906382	5.709809
206	10	5.541281	5.207545	10	5.148933	5.42381
205	10	10	10	10	10	10
204	4.908333	5.276762	10	5.630797	5.828331	5.14163
203	5.011732	5.413074	10	10	10	10
202	4.434312	4.712498	4.819783	4.401416	4.372936	4.459036
201	3.904566	4.248269	4.080481	4.47736	4.230413	4.161228
200	3.219299	3.256006	3.456336	3.60441	3.148582	3.542021

EXCLI Journal 2020;19:48-70 – ISSN 1611-2156
Received: October 03, 2019, accepted: December 19, 2019, published: January 03, 2020

Table 2: Absorption intensity in the wavelength range of 200-800 nm for absorption spectra acetylshikonin fixed concentration (8.00 x 10^{-5} M), in the absence (*A*) and presence of increasing concentration of CT-DNA (*B-F*)

	Absorption intensity					
**Wavelength (nm)**	**concentration of CT-DNA (M)**					
	A	*B*	*C*	*D*	*E*	*F*
	0.00	2.66x10^{-5}	6.21x10^{-5}	7.98x10^{-5}	1.33x10^{-4}	1.73x10^{-4}
800	0.001541	0.001528	0.001404	0.001684	0.002039	0.003523
799	0.001094	0.001381	0.001511	0.001566	0.001771	0.00347
798	0.001416	0.001403	0.001718	0.001754	0.002099	0.003307
797	0.001549	0.001777	0.00179	0.00196	0.002203	0.003711
796	0.001344	0.001606	0.002083	0.001882	0.002392	0.003425
795	0.001344	0.001674	0.001842	0.001882	0.002007	0.003494
794	0.001356	0.001278	0.0014	0.001841	0.001893	0.003082
793	0.001519	0.001655	0.001632	0.001745	0.002216	0.003614
792	0.001815	0.001884	0.002167	0.002079	0.002441	0.003854
791	0.001008	0.001544	0.001479	0.001559	0.002217	0.00344
790	0.001689	0.00191	0.001803	0.001887	0.00242	0.003422
789	0.00141	0.00123	0.001847	0.001703	0.001969	0.003229
788	0.001498	0.001274	0.001849	0.001694	0.002169	0.003214
787	0.001311	0.001159	0.001402	0.001711	0.00199	0.00327
786	0.001485	0.001537	0.00164	0.001976	0.0024	0.003266
785	0.0012	0.00149	0.001527	0.001737	0.001936	0.003267
784	0.001589	0.001591	0.001726	0.00166	0.002362	0.003474
783	0.001446	0.001582	0.001811	0.001945	0.00218	0.003621
782	0.00147	0.001458	0.001765	0.002007	0.002264	0.003443
781	0.001642	0.001602	0.001838	0.001868	0.002401	0.003611
780	0.001426	0.001541	0.001824	0.001883	0.002156	0.003472
779	0.001182	0.001452	0.001521	0.00163	0.001949	0.00333
778	0.001124	0.001393	0.001751	0.001612	0.001916	0.003351
777	0.001335	0.001319	0.001815	0.001722	0.002047	0.0033
776	0.001307	0.001472	0.001867	0.001491	0.002225	0.003656
775	0.001454	0.00191	0.001962	0.001942	0.002312	0.003683
774	0.00129	0.001634	0.001703	0.002043	0.002241	0.00352
773	0.001514	0.001559	0.00157	0.001705	0.002308	0.003398
772	0.0014	0.001556	0.00178	0.001901	0.002335	0.003582
771	0.001356	0.001364	0.001641	0.001765	0.002075	0.00332
770	0.001324	0.001275	0.001604	0.001689	0.002141	0.003119
769	0.001697	0.001682	0.001956	0.001782	0.002311	0.003762
768	0.001399	0.001509	0.001803	0.002002	0.002385	0.003742
767	0.001364	0.001296	0.001681	0.001892	0.002296	0.003384
766	0.001542	0.001543	0.001938	0.002111	0.002315	0.00358
765	0.001423	0.001253	0.001631	0.001996	0.002113	0.003562
764	0.001786	0.001714	0.001922	0.001995	0.002373	0.003652
763	0.001394	0.001609	0.001995	0.002086	0.002207	0.003438
762	0.001443	0.001493	0.001797	0.001943	0.002342	0.003527
761	0.001316	0.00156	0.001929	0.002156	0.002454	0.003646
760	0.001685	0.00157	0.001686	0.001907	0.002244	0.003784
759	0.001619	0.001689	0.001934	0.002037	0.002494	0.003766
758	0.001624	0.00164	0.001822	0.001886	0.002294	0.00374
757	0.001591	0.001885	0.002091	0.002126	0.002588	0.003985

EXCLI Journal 2020;19:48-70 – ISSN 1611-2156
Received: October 03, 2019, accepted: December 19, 2019, published: January 03, 2020

Wavelength (nm)	***Absorption intensity*** concentration of CT-DNA (M)					
	A	*B*	*C*	*D*	*E*	*F*
	0.00	$2.66x10^{-5}$	$6.21x10^{-5}$	$7.98x10^{-5}$	$1.33x10^{-4}$	$1.73x10^{-4}$
756	0.001497	0.00171	0.002043	0.002032	0.002422	0.003996
755	0.001538	0.001683	0.002028	0.002096	0.002361	0.003798
754	0.001677	0.001782	0.002024	0.002003	0.002455	0.003925
753	0.001622	0.00177	0.00185	0.002029	0.002413	0.003691
752	0.001574	0.001703	0.001848	0.001996	0.002518	0.003736
751	0.001584	0.001844	0.001951	0.002054	0.002454	0.003843
750	0.001473	0.001612	0.001894	0.001883	0.002417	0.00378
749	0.001749	0.001742	0.001767	0.002213	0.002686	0.003927
748	0.001651	0.001931	0.001998	0.00221	0.002702	0.003958
747	0.001654	0.001761	0.001904	0.002163	0.002582	0.003903
746	0.001416	0.001544	0.001644	0.001929	0.002444	0.003723
745	0.001661	0.001829	0.001989	0.002167	0.002564	0.00386
744	0.001537	0.001699	0.001856	0.002037	0.00229	0.00385
743	0.001571	0.001749	0.001933	0.002193	0.002495	0.003849
742	0.001546	0.001718	0.001843	0.002094	0.002477	0.003858
741	0.00149	0.00164	0.001829	0.002092	0.002403	0.003792
740	0.00166	0.001763	0.00192	0.002171	0.002365	0.003969
739	0.001665	0.001929	0.002001	0.002424	0.002785	0.004159
738	0.001718	0.001902	0.002082	0.002232	0.002503	0.004161
737	0.001787	0.002029	0.002205	0.002273	0.002579	0.004102
736	0.001807	0.001877	0.002019	0.002408	0.002783	0.004151
735	0.002015	0.002129	0.002304	0.002555	0.002977	0.004267
734	0.001913	0.002118	0.002267	0.002442	0.002913	0.004316
733	0.002031	0.002333	0.002463	0.002671	0.002984	0.004519
732	0.002127	0.002299	0.002583	0.002724	0.003225	0.004703
731	0.002315	0.002413	0.002488	0.002902	0.003342	0.004709
730	0.002071	0.002249	0.002415	0.0029	0.003023	0.004509
729	0.002296	0.002428	0.002656	0.002892	0.003466	0.004833
728	0.00229	0.002376	0.002717	0.002963	0.003426	0.004748
727	0.002424	0.002401	0.002627	0.002904	0.003373	0.004618
726	0.002451	0.002582	0.00275	0.003107	0.003416	0.00483
725	0.002458	0.002572	0.002909	0.003144	0.003626	0.004939
724	0.002447	0.002622	0.002759	0.003122	0.003561	0.004975
723	0.002601	0.002574	0.002841	0.003246	0.003531	0.005141
722	0.002585	0.002732	0.002929	0.003298	0.00359	0.005128
721	0.00275	0.002548	0.002955	0.003238	0.003665	0.005046
720	0.002387	0.0026	0.002894	0.003184	0.003691	0.00511
719	0.002687	0.002675	0.003028	0.003296	0.00378	0.005131
718	0.002581	0.002748	0.003087	0.003452	0.003783	0.005199
717	0.002764	0.002809	0.003071	0.003465	0.003844	0.005305
716	0.002675	0.002884	0.003204	0.003447	0.003992	0.00523
715	0.002909	0.003085	0.003428	0.003674	0.004077	0.005497
714	0.002823	0.003007	0.003389	0.003702	0.004006	0.005525
713	0.002898	0.003005	0.003347	0.003634	0.004059	0.005396
712	0.002904	0.003116	0.003329	0.003664	0.004199	0.005549
711	0.003001	0.003216	0.003455	0.003894	0.004387	0.005831
710	0.003186	0.003359	0.003618	0.00392	0.004435	0.005784
709	0.002942	0.003125	0.003303	0.00372	0.00423	0.005643

EXCLI Journal 2020;19:48-70 – ISSN 1611-2156
Received: October 03, 2019, accepted: December 19, 2019, published: January 03, 2020

Wavelength (nm)	Absorption intensity					
	concentration of CT-DNA (M)					
	A	B	C	D	E	F
	0.00	2.66×10^{-5}	6.21×10^{-5}	7.98×10^{-5}	1.33×10^{-4}	1.73×10^{-4}
708	0.003009	0.003308	0.003449	0.003922	0.004363	0.005784
707	0.003279	0.003583	0.00376	0.004121	0.004571	0.006027
706	0.003364	0.003534	0.003638	0.004124	0.004747	0.005968
705	0.00347	0.003371	0.003725	0.004026	0.004704	0.005987
704	0.003296	0.003386	0.003606	0.004156	0.004674	0.006051
703	0.003508	0.003557	0.003898	0.004349	0.004799	0.00613
702	0.003603	0.003553	0.004033	0.00425	0.004742	0.006221
701	0.003553	0.003593	0.003917	0.004481	0.004754	0.006226
700	0.003719	0.003529	0.003931	0.004501	0.004806	0.00627
699	0.003542	0.003527	0.00399	0.004371	0.004836	0.006231
698	0.003723	0.003811	0.004182	0.00454	0.004916	0.006435
697	0.003529	0.003636	0.00385	0.004248	0.004825	0.006265
696	0.00364	0.003804	0.004102	0.00452	0.004987	0.006531
695	0.003684	0.003762	0.004186	0.004577	0.004987	0.006549
694	0.004054	0.003904	0.004412	0.004776	0.005242	0.006808
693	0.004007	0.004091	0.004487	0.004879	0.005304	0.006914
692	0.003987	0.004043	0.00438	0.00475	0.005204	0.006853
691	0.004168	0.004195	0.004504	0.004815	0.005521	0.006992
690	0.004131	0.004215	0.004622	0.005016	0.005438	0.007087
689	0.004056	0.004332	0.004635	0.004979	0.00549	0.007116
688	0.004146	0.004472	0.004855	0.005023	0.005783	0.007175
687	0.004403	0.004286	0.004761	0.00509	0.005851	0.007218
686	0.004207	0.004396	0.00483	0.005171	0.005778	0.007227
685	0.004462	0.004412	0.004875	0.005257	0.005792	0.007377
684	0.004502	0.00459	0.004892	0.005448	0.005901	0.007497
683	0.004651	0.004802	0.005238	0.00558	0.006165	0.007496
682	0.004835	0.004774	0.005136	0.005633	0.006266	0.007662
681	0.004707	0.004665	0.005176	0.005657	0.006154	0.007616
680	0.004664	0.004643	0.005159	0.005734	0.006054	0.007703
679	0.005064	0.005041	0.005309	0.005881	0.006406	0.007871
678	0.004915	0.004948	0.005398	0.005834	0.006463	0.0079
677	0.004991	0.005037	0.005484	0.005998	0.006538	0.008082
676	0.005071	0.005152	0.005507	0.005983	0.006511	0.008167
675	0.005311	0.005267	0.005528	0.005993	0.006648	0.008217
674	0.005348	0.005376	0.005844	0.006369	0.006912	0.008478
673	0.005371	0.00533	0.005915	0.006247	0.00691	0.008357
672	0.005318	0.005333	0.005939	0.006188	0.006974	0.008528
671	0.005416	0.005564	0.005935	0.006418	0.006994	0.008743
670	0.005566	0.005699	0.006205	0.006605	0.007289	0.008898
669	0.005789	0.00579	0.006318	0.006761	0.007356	0.00903
668	0.005751	0.005683	0.006176	0.00669	0.007304	0.009145
667	0.005812	0.005994	0.006477	0.006833	0.007527	0.009156
666	0.006018	0.00598	0.00644	0.006901	0.007672	0.009246
665	0.006078	0.005956	0.006563	0.007018	0.007669	0.009468
664	0.006092	0.006119	0.006683	0.007131	0.007895	0.009404
663	0.006315	0.0063	0.006743	0.007262	0.008023	0.009714
662	0.006554	0.006342	0.006903	0.007495	0.008227	0.009849
661	0.006437	0.006509	0.00701	0.007476	0.008224	0.009771

EXCLI Journal 2020;19:48-70 – ISSN 1611-2156
Received: October 03, 2019, accepted: December 19, 2019, published: January 03, 2020

Absorption intensity						
**Wavelength (nm)**	**concentration of CT-DNA (M)**					
	A	*B*	*C*	*D*	*E*	*F*
	0.00	$2.66x10^{-5}$	$6.21x10^{-5}$	$7.98x10^{-5}$	$1.33x10^{-4}$	$1.73x10^{-4}$
660	0.006476	0.006472	0.006909	0.007604	0.008324	0.009943
659	0.006643	0.006596	0.007083	0.007755	0.008358	0.010143
658	0.006664	0.006717	0.007197	0.007763	0.008365	0.010229
657	0.006944	0.006919	0.007435	0.00807	0.008608	0.010476
656	0.006831	0.006894	0.007347	0.008061	0.008695	0.010485
655	0.00712	0.007064	0.007628	0.008211	0.008895	0.010783
654	0.00714	0.007222	0.007776	0.008324	0.009018	0.01079
653	0.007189	0.007421	0.007857	0.008561	0.009248	0.011072
652	0.007392	0.007425	0.008141	0.008564	0.009423	0.011199
651	0.0074	0.007512	0.008054	0.008721	0.009442	0.011256
650	0.007644	0.007717	0.008204	0.008823	0.009617	0.011371
649	0.007686	0.00777	0.008274	0.00895	0.009696	0.011501
648	0.007948	0.007973	0.008558	0.009178	0.010023	0.011841
647	0.007913	0.008062	0.008434	0.009041	0.009934	0.011801
646	0.008086	0.008061	0.008643	0.009295	0.010244	0.012025
645	0.008216	0.008202	0.008759	0.009469	0.010329	0.012124
644	0.008333	0.008428	0.008914	0.009594	0.010534	0.0124
643	0.008636	0.008696	0.009189	0.00992	0.010727	0.01262
642	0.008688	0.008526	0.009041	0.009857	0.010769	0.012602
641	0.008764	0.008697	0.00918	0.010041	0.010938	0.012801
640	0.008817	0.00881	0.009384	0.010186	0.010992	0.012886
639	0.008964	0.008744	0.009472	0.010359	0.011101	0.013049
638	0.009037	0.008999	0.009576	0.01039	0.011256	0.013268
637	0.009172	0.00913	0.009717	0.010537	0.011421	0.013395
636	0.009318	0.009337	0.009848	0.010692	0.011667	0.013634
635	0.009483	0.009428	0.010112	0.0109	0.011869	0.01386
634	0.009483	0.009506	0.010313	0.01091	0.011827	0.014076
633	0.009653	0.009686	0.010256	0.011067	0.012011	0.014125
632	0.009933	0.009977	0.010589	0.011334	0.012411	0.014572
631	0.010013	0.01008	0.010812	0.011393	0.012323	0.014523
630	0.010138	0.010293	0.010909	0.011596	0.012666	0.014751
629	0.010314	0.010256	0.010946	0.01163	0.012779	0.014788
628	0.010353	0.010566	0.011148	0.011994	0.012964	0.015125
627	0.010658	0.010725	0.011372	0.012245	0.013195	0.015425
626	0.010851	0.011024	0.01151	0.012419	0.013376	0.015625
625	0.010834	0.010937	0.011422	0.012361	0.013416	0.015512
624	0.01119	0.01118	0.011803	0.012794	0.013801	0.015964
623	0.011323	0.011317	0.011911	0.012759	0.013849	0.01598
622	0.011479	0.011431	0.012031	0.012938	0.013967	0.016199
621	0.011414	0.011476	0.012093	0.013085	0.014113	0.016313
620	0.011728	0.011612	0.01231	0.013252	0.014234	0.016586
619	0.011851	0.011795	0.012441	0.013329	0.014396	0.016764
618	0.0119	0.011823	0.012569	0.013554	0.014478	0.016804
617	0.012109	0.011945	0.012735	0.013719	0.014775	0.017193
616	0.012323	0.012249	0.012866	0.013941	0.014898	0.017393
615	0.012436	0.012337	0.013017	0.014011	0.015041	0.017536
614	0.012566	0.012465	0.013399	0.014247	0.015332	0.017782
613	0.012644	0.012723	0.013312	0.014145	0.015357	0.017823

EXCLI Journal 2020;19:48-70 – ISSN 1611-2156
Received: October 03, 2019, accepted: December 19, 2019, published: January 03, 2020

Wavelength (nm)	Absorption intensity					
	concentration of CT-DNA (M)					
	A	*B*	*C*	*D*	*E*	*F*
	0.00	$2.66x10^{-5}$	$6.21x10^{-5}$	$7.98x10^{-5}$	$1.33x10^{-4}$	$1.73x10^{-4}$
612	0.012717	0.01283	0.013491	0.0144	0.015442	0.018027
611	0.01298	0.013012	0.013691	0.01466	0.015789	0.01832
610	0.013215	0.013265	0.013833	0.014804	0.01592	0.018557
609	0.013329	0.013379	0.014021	0.014984	0.01611	0.01864
608	0.013414	0.013556	0.014175	0.015091	0.016352	0.018788
607	0.013497	0.013631	0.014368	0.015094	0.016484	0.01886
606	0.013799	0.013736	0.014349	0.015382	0.016632	0.019208
605	0.014047	0.014059	0.014813	0.015675	0.016928	0.019452
604	0.014235	0.014152	0.014829	0.015791	0.017067	0.019524
603	0.014296	0.014261	0.014927	0.015951	0.017148	0.019807
602	0.014389	0.014437	0.014969	0.016054	0.017241	0.019947
601	0.014576	0.014537	0.01525	0.016309	0.017596	0.020116
600	0.014732	0.014671	0.015348	0.016395	0.017677	0.019988
599	0.015096	0.014951	0.015566	0.016842	0.017947	0.020489
598	0.01502	0.014863	0.015632	0.016645	0.017976	0.020591
597	0.015142	0.015107	0.015962	0.017059	0.018119	0.020754
596	0.015312	0.015328	0.015989	0.017232	0.018348	0.020927
595	0.015462	0.015373	0.016172	0.017364	0.018427	0.021097
594	0.015644	0.015688	0.016487	0.017532	0.018593	0.021426
593	0.015904	0.01593	0.016708	0.017824	0.018961	0.021685
592	0.015907	0.016003	0.016767	0.017813	0.019047	0.021801
591	0.016133	0.016185	0.016835	0.017924	0.019162	0.021866
590	0.016295	0.016316	0.017104	0.018234	0.019439	0.022291
589	0.016642	0.016573	0.017333	0.018343	0.019632	0.0225
588	0.0168	0.016694	0.017341	0.018507	0.019576	0.022369
587	0.016317	0.016476	0.017105	0.018576	0.019501	0.02232
586	0.016686	0.016693	0.017454	0.018922	0.019878	0.022608
585	0.017096	0.017112	0.017833	0.018879	0.020317	0.023077
584	0.017439	0.01743	0.018058	0.01915	0.020597	0.023442
583	0.017678	0.017543	0.018235	0.019465	0.0208	0.023577
582	0.017746	0.017737	0.018519	0.019589	0.020996	0.023737
581	0.017966	0.017848	0.018565	0.019809	0.021099	0.024048
580	0.0181	0.017954	0.018742	0.019935	0.021234	0.023976
579	0.018032	0.018055	0.018828	0.019952	0.02134	0.024169
578	0.018208	0.018303	0.01896	0.02029	0.021557	0.024449
577	0.018567	0.018455	0.019261	0.020512	0.021833	0.024702
576	0.018552	0.018576	0.019385	0.020457	0.021839	0.024731
575	0.018835	0.018717	0.019492	0.020379	0.021877	0.024905
574	0.019086	0.018954	0.019794	0.020656	0.022234	0.025284
573	0.019145	0.019061	0.019822	0.020649	0.022407	0.025316
572	0.019266	0.019319	0.020114	0.020805	0.022609	0.025667
571	0.019368	0.01949	0.020353	0.021041	0.022855	0.025777
570	0.019612	0.019635	0.020442	0.021126	0.022868	0.025939
569	0.019618	0.019754	0.020544	0.021265	0.023009	0.026135
568	0.019863	0.01982	0.020712	0.02184	0.023225	0.026319
567	0.020046	0.020053	0.020936	0.022004	0.023399	0.02637
566	0.020069	0.02016	0.020985	0.022239	0.023495	0.026621
565	0.020326	0.020207	0.021084	0.022337	0.023641	0.026783

Wavelength (nm)	Absorption intensity					
	concentration of CT-DNA (M)					
	A	*B*	*C*	*D*	*E*	*F*
	0.00	$2.66x10^{-5}$	$6.21x10^{-5}$	$7.98x10^{-5}$	$1.33x10^{-4}$	$1.73x10^{-4}$
564	0.020451	0.02041	0.021246	0.02251	0.023972	0.026862
563	0.02056	0.020617	0.021378	0.022678	0.023957	0.02702
562	0.02066	0.020686	0.021432	0.022805	0.024218	0.027179
561	0.02086	0.020808	0.021617	0.022855	0.024152	0.027237
560	0.020928	0.021058	0.021717	0.023039	0.024442	0.027592
559	0.020946	0.021101	0.021804	0.023106	0.024437	0.027591
558	0.02136	0.02121	0.022175	0.023366	0.024756	0.027734
557	0.021188	0.021092	0.022092	0.023422	0.024757	0.027814
556	0.02128	0.021297	0.022041	0.02341	0.024714	0.027715
555	0.021273	0.021123	0.021909	0.023336	0.024472	0.027723
554	0.021357	0.021199	0.022126	0.023365	0.02464	0.027827
553	0.021788	0.021619	0.022474	0.023732	0.024912	0.027996
552	0.022007	0.022044	0.022795	0.024252	0.025457	0.028458
551	0.021967	0.021932	0.022916	0.024242	0.025355	0.028718
550	0.022376	0.022301	0.023227	0.024514	0.025764	0.029003
549	0.022434	0.022403	0.023298	0.024654	0.025805	0.029072
548	0.02241	0.022559	0.023486	0.024884	0.025922	0.029155
547	0.022585	0.022702	0.023607	0.024703	0.025934	0.029437
546	0.02281	0.022962	0.023783	0.025063	0.026264	0.029477
545	0.022983	0.0231	0.023992	0.025222	0.026492	0.029707
544	0.023099	0.023354	0.024226	0.025487	0.026794	0.03005
543	0.023327	0.02348	0.024336	0.025604	0.0269	0.030168
542	0.023462	0.023544	0.024364	0.025685	0.026962	0.030138
541	0.023551	0.023743	0.024486	0.025888	0.027175	0.030243
540	0.023851	0.024164	0.024934	0.026164	0.027509	0.030722
539	0.024174	0.024191	0.025062	0.026281	0.027611	0.030735
538	0.024182	0.02433	0.025069	0.026389	0.027747	0.030851
537	0.024351	0.024353	0.025327	0.026589	0.027951	0.030927
536	0.024564	0.024566	0.025489	0.026722	0.028044	0.031212
535	0.024588	0.024648	0.025541	0.026859	0.028094	0.031344
534	0.02482	0.025009	0.025803	0.027229	0.028257	0.031669
533	0.024836	0.025004	0.025862	0.027362	0.028428	0.031675
532	0.02504	0.025121	0.026057	0.027548	0.028692	0.031916
531	0.025278	0.025449	0.026372	0.02775	0.028748	0.03217
530	0.025252	0.025417	0.026492	0.027792	0.028853	0.032214
529	0.02551	0.025614	0.026495	0.027976	0.029012	0.032339
528	0.02559	0.025881	0.026783	0.027984	0.029236	0.032599
527	0.025774	0.025851	0.02687	0.028243	0.029317	0.032745
526	0.025868	0.026146	0.027049	0.028313	0.029403	0.032782
525	0.026235	0.026575	0.027403	0.028696	0.029827	0.033175
524	0.026183	0.026569	0.027473	0.028628	0.029908	0.033207
523	0.026404	0.026713	0.027605	0.02891	0.030063	0.03332
522	0.026399	0.026893	0.027645	0.028967	0.030209	0.033252
521	0.026633	0.027071	0.027891	0.029091	0.030165	0.033116
520	0.026495	0.026777	0.027682	0.028789	0.030002	0.033262
519	0.026534	0.026788	0.02779	0.028967	0.030126	0.033339
518	0.026797	0.027219	0.027955	0.029303	0.030416	0.033704
517	0.026787	0.027201	0.027941	0.029295	0.030409	0.033625

EXCLI Journal 2020;19:48-70 – ISSN 1611-2156
Received: October 03, 2019, accepted: December 19, 2019, published: January 03, 2020

Absorption intensity						
**Wavelength (nm)**	**concentration of CT-DNA (M)**					
	A	*B*	*C*	*D*	*E*	*F*
	0.00	$2.66x10^{-5}$	$6.21x10^{-5}$	$7.98x10^{-5}$	$1.33x10^{-4}$	$1.73x10^{-4}$
516	0.026805	0.027086	0.027941	0.029301	0.030411	0.033614
515	0.027282	0.027383	0.028277	0.029671	0.030699	0.033978
514	0.027074	0.027361	0.028261	0.029581	0.030623	0.034004
513	0.027243	0.027577	0.028482	0.029848	0.030773	0.03419
512	0.027252	0.027744	0.028529	0.029781	0.0309	0.034145
511	0.027452	0.027883	0.028841	0.030098	0.031059	0.034404
510	0.02744	0.027992	0.028863	0.030283	0.031186	0.034623
509	0.027886	0.028237	0.029156	0.03039	0.031342	0.034827
508	0.027765	0.02821	0.029239	0.030489	0.031424	0.034876
507	0.027837	0.028345	0.02928	0.030522	0.031537	0.034877
506	0.027926	0.028416	0.029285	0.030597	0.031581	0.034909
505	0.027974	0.028493	0.029357	0.030588	0.03159	0.035131
504	0.028217	0.028676	0.029652	0.030727	0.03186	0.035139
503	0.028316	0.028867	0.029701	0.030958	0.031926	0.035277
502	0.028446	0.029072	0.029884	0.031061	0.032122	0.035484
501	0.028588	0.029075	0.029961	0.031334	0.03229	0.035609
500	0.02876	0.029465	0.030315	0.031494	0.032509	0.035841
499	0.028794	0.029373	0.030238	0.031519	0.032549	0.035856
498	0.028932	0.029464	0.030342	0.031507	0.032652	0.035901
497	0.02898	0.029439	0.030314	0.031626	0.032635	0.035942
496	0.029036	0.029554	0.03032	0.03171	0.03264	0.036138
495	0.028981	0.029521	0.030252	0.031531	0.032707	0.035799
494	0.02904	0.029618	0.030425	0.031876	0.0327	0.035928
493	0.028956	0.029631	0.030535	0.031785	0.032875	0.036034
492	0.029192	0.029809	0.030622	0.032123	0.033007	0.036165
491	0.029218	0.029918	0.030642	0.032004	0.032733	0.036309
490	0.029238	0.029836	0.030787	0.032084	0.032889	0.036253
489	0.029359	0.030022	0.030946	0.032237	0.033018	0.036391
488	0.029358	0.03017	0.031011	0.032189	0.032849	0.036376
487	0.02943	0.030071	0.031023	0.032231	0.032967	0.036452
486	0.029735	0.030344	0.031047	0.032158	0.033078	0.036469
485	0.029841	0.030526	0.031265	0.032448	0.033267	0.036473
484	0.029815	0.030637	0.031478	0.032674	0.033582	0.03659
483	0.02991	0.030723	0.031572	0.03266	0.033598	0.036712
482	0.029838	0.030581	0.031293	0.032671	0.033596	0.036728
481	0.02987	0.03066	0.031388	0.032733	0.033614	0.03686
480	0.030035	0.030729	0.031396	0.032762	0.033672	0.037011
479	0.029874	0.03065	0.031395	0.032688	0.033643	0.036832
478	0.029997	0.030728	0.031418	0.03284	0.033682	0.036915
477	0.02994	0.030595	0.031277	0.032727	0.033621	0.036609
476	0.029943	0.030663	0.03146	0.032826	0.033606	0.03654
475	0.029877	0.030581	0.031136	0.032466	0.03317	0.036461
474	0.029413	0.030113	0.031023	0.032195	0.033166	0.036281
473	0.029464	0.030181	0.030982	0.032381	0.033095	0.036409
472	0.029364	0.029944	0.030895	0.032094	0.0331	0.036244
471	0.029317	0.029963	0.030823	0.03216	0.032946	0.036212
470	0.029355	0.03019	0.030821	0.03237	0.033035	0.036409
469	0.029215	0.03006	0.030818	0.032096	0.032941	0.036127

EXCLI Journal 2020;19:48-70 – ISSN 1611-2156
Received: October 03, 2019, accepted: December 19, 2019, published: January 03, 2020

Absorption intensity						
**Wavelength (nm)**	**concentration of CT-DNA (M)**					
	A	*B*	*C*	*D*	*E*	*F*
	0.00	$2.66x10^{-5}$	$6.21x10^{-5}$	$7.98x10^{-5}$	$1.33x10^{-4}$	$1.73x10^{-4}$
468	0.029523	0.03013	0.031116	0.032256	0.033199	0.03641
467	0.029562	0.030357	0.031028	0.03248	0.033204	0.036682
466	0.029359	0.030217	0.03108	0.032253	0.033129	0.036469
465	0.02935	0.030293	0.030884	0.032297	0.03301	0.036455
464	0.029319	0.030173	0.030815	0.032145	0.033003	0.036242
463	0.029132	0.030263	0.030944	0.032101	0.033114	0.036285
462	0.029287	0.030117	0.030711	0.031979	0.032937	0.036246
461	0.029085	0.030033	0.030585	0.031929	0.032773	0.036047
460	0.029013	0.029881	0.030492	0.031843	0.032797	0.035958
459	0.028973	0.029943	0.030534	0.031959	0.032832	0.035878
458	0.028914	0.029698	0.030339	0.031741	0.032608	0.03584
457	0.028817	0.029749	0.030246	0.031602	0.032512	0.035741
456	0.028916	0.029746	0.030233	0.031664	0.032508	0.035633
455	0.028752	0.029372	0.030022	0.031364	0.032232	0.035399
454	0.028497	0.029367	0.029975	0.031526	0.032274	0.035539
453	0.028437	0.029206	0.029886	0.031297	0.032153	0.035389
452	0.028303	0.029236	0.029918	0.031154	0.031956	0.035234
451	0.02816	0.02893	0.029645	0.030969	0.03181	0.034996
450	0.028103	0.028975	0.029698	0.030983	0.031761	0.035143
449	0.02787	0.028631	0.029369	0.0306	0.031572	0.034866
448	0.028018	0.028884	0.029577	0.030966	0.031809	0.035101
447	0.027833	0.028675	0.029409	0.030595	0.031464	0.034807
446	0.027675	0.028702	0.029204	0.030693	0.03161	0.034727
445	0.027591	0.028625	0.029183	0.030281	0.031353	0.034669
444	0.027564	0.028612	0.029092	0.030395	0.031254	0.03475
443	0.027275	0.028334	0.028785	0.030177	0.031232	0.034278
442	0.027317	0.028131	0.028755	0.03003	0.031062	0.0342
441	0.027111	0.02809	0.028758	0.029945	0.031088	0.03412
440	0.027138	0.027946	0.028531	0.029781	0.030809	0.034043
439	0.027017	0.027857	0.028341	0.029678	0.03077	0.033815
438	0.026671	0.027638	0.028314	0.029479	0.030636	0.033751
437	0.026972	0.027646	0.028237	0.029565	0.030685	0.033881
436	0.026727	0.027439	0.028017	0.029469	0.030289	0.033342
435	0.026569	0.027419	0.02806	0.029279	0.030163	0.033394
434	0.026376	0.02721	0.027755	0.029182	0.030025	0.03318
433	0.026227	0.027105	0.027671	0.029106	0.029877	0.033175
432	0.026261	0.026984	0.027515	0.028978	0.029982	0.033147
431	0.026005	0.027152	0.027599	0.028845	0.02989	0.033071
430	0.025744	0.02668	0.0274	0.028609	0.029654	0.032769
429	0.025668	0.026626	0.027004	0.028379	0.029463	0.03269
428	0.025596	0.026563	0.02718	0.028464	0.029561	0.032671
427	0.025501	0.026509	0.027064	0.028288	0.029363	0.032532
426	0.025358	0.026401	0.026953	0.028218	0.029182	0.032488
425	0.02553	0.026369	0.026824	0.02803	0.029125	0.03229
424	0.025192	0.026029	0.026564	0.027811	0.028963	0.032196
423	0.025195	0.026259	0.026883	0.027977	0.028959	0.032352
422	0.025057	0.026037	0.026346	0.027846	0.02888	0.032033
421	0.025295	0.025975	0.02663	0.027789	0.028881	0.032182

EXCLI Journal 2020;19:48-70 – ISSN 1611-2156
Received: October 03, 2019, accepted: December 19, 2019, published: January 03, 2020

Wavelength (nm)	***Absorption intensity***					
	**concentration of CT-DNA (M)**					
	A	*B*	*C*	*D*	*E*	*F*
	0.00	$2.66x10^{-5}$	$6.21x10^{-5}$	$7.98x10^{-5}$	$1.33x10^{-4}$	$1.73x10^{-4}$
420	0.025136	0.025868	0.026282	0.027782	0.028819	0.031933
419	0.025019	0.025801	0.026447	0.027763	0.028895	0.031961
418	0.024873	0.025798	0.026285	0.027696	0.028752	0.031849
417	0.024866	0.025539	0.026244	0.027537	0.028599	0.031715
416	0.024695	0.025597	0.026178	0.027578	0.028614	0.031658
415	0.024985	0.025483	0.025961	0.027377	0.028609	0.03155
414	0.024581	0.02529	0.025844	0.027392	0.028445	0.03151
413	0.024568	0.025094	0.025965	0.02741	0.028395	0.031486
412	0.024605	0.025257	0.025813	0.02733	0.028398	0.031505
411	0.024188	0.024967	0.025553	0.026897	0.028035	0.031288
410	0.024432	0.025076	0.025653	0.027037	0.028122	0.031192
409	0.024212	0.024819	0.025525	0.026744	0.027867	0.031048
408	0.023903	0.024636	0.025314	0.026655	0.027775	0.030975
407	0.023747	0.024542	0.025009	0.026386	0.027517	0.030849
406	0.02399	0.024764	0.025341	0.026846	0.027756	0.031003
405	0.024286	0.025003	0.025637	0.026802	0.028053	0.031263
404	0.024297	0.025318	0.025786	0.026882	0.028198	0.031408
403	0.02417	0.025076	0.025611	0.026736	0.028133	0.031187
402	0.024345	0.024991	0.025697	0.026897	0.028357	0.03149
401	0.024296	0.024762	0.025517	0.026946	0.028151	0.031254
400	0.024243	0.024848	0.025213	0.026644	0.028064	0.031048
399	0.024311	0.024832	0.025453	0.026902	0.028322	0.031117
398	0.024453	0.024936	0.02541	0.02693	0.0282	0.031315
397	0.024222	0.024753	0.02539	0.026876	0.028233	0.030993
396	0.024574	0.025035	0.025692	0.027238	0.028268	0.031393
395	0.024617	0.025016	0.025845	0.027131	0.028602	0.031357
394	0.024675	0.025133	0.025896	0.027171	0.028595	0.031401
393	0.024823	0.025175	0.02592	0.027483	0.028644	0.031598
392	0.024849	0.02528	0.025952	0.027295	0.028773	0.031503
391	0.025131	0.025538	0.026329	0.027631	0.029104	0.031811
390	0.024359	0.024792	0.025481	0.026848	0.028144	0.031284
389	0.025112	0.025358	0.026248	0.027588	0.028681	0.032059
388	0.024951	0.025671	0.025913	0.027718	0.028854	0.031991
387	0.025138	0.025634	0.02624	0.027666	0.028881	0.032187
386	0.025021	0.025445	0.026428	0.027804	0.029049	0.032136
385	0.025372	0.025799	0.026635	0.027935	0.029039	0.032201
384	0.02519	0.025558	0.026225	0.027764	0.029067	0.031998
383	0.025738	0.026262	0.026706	0.028168	0.029476	0.032788
382	0.025715	0.026395	0.027052	0.028238	0.029886	0.032941
381	0.026017	0.02631	0.026937	0.028626	0.030047	0.032875
380	0.026239	0.026766	0.026993	0.028639	0.029992	0.033147
379	0.025994	0.026344	0.027084	0.028657	0.030266	0.033272
378	0.02648	0.026735	0.027504	0.029059	0.0305	0.033257
377	0.026534	0.026728	0.027402	0.028846	0.03034	0.033271
376	0.026629	0.026962	0.027894	0.029191	0.030605	0.033697
375	0.026824	0.02668	0.027903	0.029037	0.030686	0.033393
374	0.026492	0.026745	0.027658	0.029043	0.030702	0.033459
373	0.02706	0.027276	0.02798	0.029424	0.030948	0.034037

EXCLI Journal 2020;19:48-70 – ISSN 1611-2156
Received: October 03, 2019, accepted: December 19, 2019, published: January 03, 2020

Wavelength (nm)	***Absorption intensity***					
	**concentration of CT-DNA (M)**					
	A	*B*	*C*	*D*	*E*	*F*
	0.00	$2.66x10^{-5}$	$6.21x10^{-5}$	$7.98x10^{-5}$	$1.33x10^{-4}$	$1.73x10^{-4}$
372	0.027028	0.027271	0.02849	0.029955	0.0314	0.034127
371	0.026467	0.027107	0.027866	0.029553	0.031011	0.033886
370	0.02706	0.027317	0.028154	0.029666	0.031102	0.033999
369	0.026888	0.027392	0.028249	0.029732	0.031352	0.034158
368	0.027792	0.027903	0.029174	0.030373	0.031766	0.034725
367	0.027517	0.027158	0.028667	0.029875	0.031284	0.034748
366	0.027553	0.027758	0.028449	0.030374	0.031764	0.035055
365	0.027567	0.027849	0.029403	0.03088	0.032035	0.034888
364	0.027942	0.028265	0.029239	0.030734	0.031861	0.035278
363	0.028142	0.028521	0.029618	0.030567	0.032546	0.035104
362	0.028699	0.028862	0.030312	0.03145	0.033235	0.036818
361	0.026802	0.02762	0.027943	0.029317	0.030909	0.034185
360	0.028317	0.028524	0.030158	0.031514	0.032728	0.035723
359	0.028422	0.029	0.030014	0.031424	0.032985	0.036256
358	0.028862	0.02943	0.030569	0.031551	0.033928	0.036466
357	0.029975	0.029618	0.031482	0.032619	0.034431	0.03702
356	0.02615	0.027129	0.027706	0.029064	0.031047	0.034266
355	0.029825	0.029744	0.030542	0.031458	0.034096	0.037829
354	0.029498	0.029066	0.031555	0.031875	0.034388	0.037021
353	0.029387	0.029702	0.031015	0.03266	0.033981	0.036491
352	0.030253	0.028792	0.030814	0.03257	0.034869	0.037161
351	0.029439	0.029399	0.031577	0.032413	0.034268	0.036554
350	0.030538	0.028759	0.030753	0.032828	0.03394	0.038902
349	0.033506	0.031304	0.032908	0.03732	0.038791	0.040315
348	0.030609	0.030199	0.031692	0.032949	0.034886	0.038542
347	0.030699	0.030949	0.031486	0.033793	0.035846	0.039362
346	0.031061	0.030988	0.032277	0.033998	0.035824	0.039607
345	0.031889	0.031462	0.032448	0.033869	0.036877	0.040181
344	0.032147	0.031963	0.032744	0.034584	0.036777	0.040571
343	0.031918	0.032112	0.033378	0.034653	0.036601	0.041241
342	0.031971	0.031641	0.033009	0.034924	0.037393	0.040637
341	0.032247	0.031807	0.032906	0.035471	0.037025	0.040669
340	0.032737	0.032997	0.033787	0.035846	0.03828	0.041883
339	0.032279	0.032484	0.033472	0.036009	0.037759	0.042057
338	0.032753	0.032802	0.03462	0.036272	0.03878	0.042415
337	0.033088	0.033346	0.034467	0.036191	0.039131	0.042014
336	0.034155	0.033587	0.034964	0.036983	0.039134	0.043081
335	0.033853	0.033851	0.03537	0.037361	0.039523	0.043779
334	0.034472	0.034689	0.035576	0.037646	0.039752	0.043602
333	0.03502	0.034825	0.035788	0.038	0.040267	0.044791
332	0.035969	0.035467	0.037012	0.039195	0.041394	0.046005
331	0.035755	0.036174	0.037125	0.039591	0.041988	0.045768
330	0.036871	0.036308	0.037967	0.040086	0.042178	0.046616
329	0.037778	0.036943	0.03863	0.040571	0.043235	0.047169
328	0.036845	0.037103	0.03873	0.040511	0.043259	0.047247
327	0.038898	0.038339	0.040008	0.042168	0.044279	0.049031
326	0.038523	0.038186	0.039591	0.041894	0.044616	0.049099
325	0.039835	0.039449	0.040617	0.043188	0.045519	0.050275

EXCLI Journal 2020;19:48-70 – ISSN 1611-2156
Received: October 03, 2019, accepted: December 19, 2019, published: January 03, 2020

Absorption intensity						
Wavelength (nm)	concentration of CT-DNA (M)					
	A	*B*	*C*	*D*	*E*	*F*
	0.00	2.66x10^{-5}	6.21x10^{-5}	7.98x10^{-5}	1.33x10^{-4}	1.73x10^{-4}
324	0.039965	0.039775	0.041531	0.043703	0.04633	0.05095
323	0.040918	0.040575	0.041384	0.04385	0.04688	0.052076
322	0.041843	0.041251	0.042974	0.045147	0.048387	0.053046
321	0.042406	0.042286	0.043655	0.046409	0.049058	0.05341
320	0.043011	0.042965	0.044845	0.046735	0.0501	0.054282
319	0.044123	0.043965	0.045512	0.047931	0.050946	0.055392
318	0.044708	0.044525	0.04633	0.049018	0.051722	0.056276
317	0.045656	0.045197	0.04665	0.049487	0.052481	0.057116
316	0.046463	0.046141	0.047876	0.050418	0.053596	0.058028
315	0.047365	0.047582	0.048988	0.051508	0.055092	0.059984
314	0.047982	0.047647	0.049695	0.051986	0.055439	0.060859
313	0.049055	0.049221	0.050324	0.053547	0.056747	0.06189
312	0.0502	0.049966	0.051635	0.054654	0.058336	0.062869
311	0.050467	0.050683	0.052423	0.055888	0.059498	0.064694
310	0.051809	0.051673	0.053843	0.056625	0.060564	0.065849
309	0.052844	0.053369	0.055227	0.058098	0.062672	0.068465
308	0.054371	0.054807	0.057086	0.060231	0.064562	0.070216
307	0.054858	0.055429	0.058067	0.061823	0.066267	0.072165
306	0.056643	0.057337	0.059587	0.063714	0.069379	0.074938
305	0.057789	0.058389	0.061765	0.066014	0.071863	0.078182
304	0.058259	0.059703	0.063158	0.067527	0.074471	0.080983
303	0.059664	0.061164	0.065845	0.070671	0.078674	0.085337
302	0.060505	0.062944	0.067809	0.073846	0.083121	0.090557
301	0.061058	0.064415	0.070781	0.077317	0.088851	0.096417
300	0.062222	0.06607	0.073773	0.081457	0.095247	0.10338
299	0.063029	0.068098	0.077468	0.086483	0.102809	0.112008
298	0.063791	0.070557	0.082164	0.092966	0.112977	0.12319
297	0.064976	0.07303	0.087709	0.099832	0.124654	0.136023
296	0.065545	0.076381	0.093696	0.108438	0.138232	0.151174
295	0.066257	0.079142	0.100616	0.117983	0.154115	0.168729
294	0.067185	0.082786	0.108832	0.129318	0.172407	0.189032
293	0.067907	0.086876	0.118462	0.142076	0.193012	0.211963
292	0.068607	0.091142	0.128268	0.155473	0.214773	0.236536
291	0.0692	0.095429	0.138767	0.169543	0.238976	0.263209
290	0.069904	0.100332	0.150116	0.18566	0.264502	0.291805
289	0.070869	0.105888	0.162052	0.202275	0.291757	0.322003
288	0.071647	0.110804	0.174563	0.218782	0.319113	0.352777
287	0.072075	0.116193	0.18768	0.236835	0.348321	0.384851
286	0.07268	0.122174	0.201123	0.254763	0.378382	0.418647
285	0.073712	0.127933	0.21497	0.274031	0.409529	0.452675
284	0.074116	0.133673	0.22922	0.293256	0.441432	0.487847
283	0.074308	0.13944	0.243227	0.312213	0.471862	0.522592
282	0.075309	0.145599	0.257586	0.331574	0.503613	0.556955
281	0.075688	0.151243	0.271474	0.349572	0.532968	0.589779
280	0.076608	0.156585	0.285098	0.367355	0.562081	0.622007
279	0.076943	0.161841	0.298038	0.384924	0.589687	0.652916
278	0.078	0.167885	0.311224	0.401854	0.617473	0.68358
277	0.077879	0.172942	0.324739	0.419377	0.646067	0.715213

EXCLI Journal 2020;19:48-70 – ISSN 1611-2156
Received: October 03, 2019, accepted: December 19, 2019, published: January 03, 2020

Wavelength (nm)	***Absorption intensity***					
	**concentration of CT-DNA (M)**					
	A	*B*	*C*	*D*	*E*	*F*
	0.00	$2.66x10^{-5}$	$6.21x10^{-5}$	$7.98x10^{-5}$	$1.33x10^{-4}$	$1.73x10^{-4}$
276	0.079278	0.178443	0.33737	0.436831	0.673988	0.746324
275	0.08003	0.183934	0.350949	0.454291	0.702679	0.777954
274	0.080901	0.189469	0.36428	0.471682	0.730679	0.809585
273	0.081997	0.195111	0.377209	0.489544	0.759304	0.84074
272	0.083277	0.200545	0.390126	0.50681	0.786986	0.871913
271	0.084215	0.206066	0.402658	0.523179	0.813783	0.901039
270	0.086144	0.212183	0.415043	0.539401	0.839556	0.929202
269	0.08718	0.217056	0.426383	0.554496	0.863735	0.956303
268	0.088838	0.222435	0.436953	0.56904	0.886097	0.981787
267	0.091355	0.227851	0.448333	0.583712	0.909389	1.007145
266	0.093387	0.232979	0.458342	0.596981	0.930812	1.03059
265	0.095864	0.238416	0.468613	0.610724	0.95136	1.054453
264	0.098342	0.243615	0.478592	0.624128	0.971884	1.076334
263	0.100824	0.248448	0.487815	0.636023	0.990659	1.097459
262	0.103517	0.253724	0.495661	0.647349	1.007718	1.116385
261	0.106181	0.257803	0.503904	0.656882	1.02334	1.132608
260	0.109332	0.262465	0.510685	0.665326	1.035308	1.145102
259	0.112012	0.266179	0.516011	0.671243	1.043355	1.154478
258	0.115349	0.270063	0.519881	0.676442	1.049165	1.160293
257	0.118478	0.272683	0.522447	0.678894	1.051003	1.162806
256	0.12245	0.276078	0.524565	0.680563	1.050992	1.161796
255	0.126663	0.279299	0.526295	0.680756	1.048791	1.158514
254	0.13132	0.282291	0.526223	0.679941	1.043561	1.152602
253	0.137374	0.285911	0.526835	0.677727	1.036019	1.144948
252	0.143227	0.289595	0.5263	0.67504	1.027208	1.133397
251	0.150363	0.293747	0.525003	0.670504	1.014971	1.119712
250	0.158679	0.298435	0.524334	0.66604	1.001924	1.103034
249	0.167911	0.304009	0.522818	0.660618	0.985763	1.085198
248	0.178488	0.31047	0.522599	0.655083	0.968708	1.066031
247	0.192147	0.319917	0.524162	0.651965	0.953832	1.047782
246	0.207839	0.330941	0.527334	0.650043	0.940159	1.030517
245	0.227867	0.346452	0.534716	0.652837	0.929256	1.017399
244	0.253683	0.367322	0.547617	0.660285	0.924613	1.009448
243	0.286962	0.395668	0.568169	0.675761	0.925819	1.008923
242	0.329696	0.435015	0.598855	0.701786	0.939289	1.019897
241	0.388616	0.489874	0.646149	0.744754	0.968309	1.047628
240	0.467266	0.565237	0.71395	0.808053	1.019242	1.097758
239	0.575809	0.670692	0.81217	0.903644	1.100619	1.17924
238	0.721399	0.815057	0.948715	1.037503	1.220661	1.301259
237	0.921401	1.013855	1.142327	1.228172	1.397814	1.482406
236	1.185946	1.279319	1.401611	1.486907	1.641933	1.730218
235	1.539525	1.634689	1.75137	1.83551	1.975097	2.073493
234	1.992937	2.09299	2.207138	2.288986	2.414632	2.51969
233	2.568442	2.671152	2.784346	2.865845	2.97459	3.091741
232	3.226138	3.342097	3.449931	3.534099	3.649149	3.767519
231	3.788607	3.895082	4.019025	4.13518	4.228977	4.357332
230	4.090559	4.215494	4.303824	4.485368	4.583159	4.621447
229	4.287682	4.386189	4.54911	4.665634	4.758565	4.81046

EXCLI Journal 2020;19:48-70 – ISSN 1611-2156
Received: October 03, 2019, accepted: December 19, 2019, published: January 03, 2020

Absorption intensity						
**Wavelength (nm)**	**concentration of CT-DNA (M)**					
	A	*B*	*C*	*D*	*E*	*F*
	0.00	$2.66x10^{-5}$	$6.21x10^{-5}$	$7.98x10^{-5}$	$1.33x10^{-4}$	$1.73x10^{-4}$
228	4.458736	4.59366	4.719708	4.780195	4.977771	4.873893
227	4.63866	4.710401	4.873849	4.93012	5.022459	5.155314
226	4.761343	4.85667	4.963235	5.134474	5.406517	5.078522
225	4.798546	5.005779	5.258414	5.121428	5.477108	5.269159
224	4.959839	5.014879	5.105683	5.282024	5.21197	5.421949
223	5.006666	4.983975	5.04369	5.307654	5.285081	5.213364
222	5.133408	5.184542	5.203006	5.252753	5.4507	6.020707
221	5.076552	5.211215	5.492921	5.678371	5.298356	5.423224
220	5.035284	5.364278	5.762197	5.426304	5.323027	5.310061
219	5.193179	5.680153	5.182833	5.503961	5.411599	5.395245
218	5.119575	5.278362	5.906662	5.530915	5.291529	5.531161
217	5.313811	5.222654	5.945666	5.147213	6.189193	5.299603
216	6.770102	5.427632	5.389894	5.924847	10	10
215	5.301168	5.270787	5.271036	5.469057	5.544088	10
214	10	10	5.610525	5.308941	10	10
213	5.619053	5.919674	10	5.852872	10	5.283424
212	10	10	10	5.237737	10	10
211	5.637806	5.939007	10	5.407472	10	10
210	10	10	10	10	10	10
209	5.259217	5.450841	5.737033	5.539919	6.183815	5.627522
208	5.40897	5.180542	5.377921	10	5.979795	10
207	5.274879	5.289453	5.711343	5.371888	5.639178	5.150227
206	10	5.540804	5.377423	5.25695	6.656183	5.701165
205	10	10	10	10	10	10
204	5.082975	4.815794	5.229589	10	10	5.094937
203	5.615692	10	5.61903	10	10	10
202	4.260777	4.575839	4.331821	4.234318	4.589335	4.58594
201	3.900676	4.016118	4.205883	4.203389	4.107603	4.114995
200	3.318591	3.381732	3.266143	3.839047	3.324218	3.245551

EXCLI Journal 2020;19:48-70 – ISSN 1611-2156
Received: October 03, 2019, accepted: December 19, 2019, published: January 03, 2020

Table 3: Absorption intensity in the wavelength range of 200-800 nm for absorption spectra *β*-hydroxy-isovalerylshikonin fixed concentration (8.00 x 10^{-5} M), in the absence (*A*) and presence of increasing concentration of CT-DNA (*B-F*)

Wavelength (nm)	***Absorption intensity***					
	**concentration of CT-DNA (M)**					
	A	*B*	*C*	*D*	*E*	*F*
	0.00	2.66x10^{-5}	6.21x10^{-5}	7.98x10^{-5}	1.33x10^{-4}	1.73x10^{-4}
800	0.001104	0.001812	0.001203	0.002431	0.003925	0.003752
799	0.00114	0.001825	0.001586	0.002026	0.004054	0.00385
798	0.00116	0.001875	0.001528	0.002434	0.004036	0.003756
797	0.001469	0.002203	0.001772	0.002497	0.004141	0.004084
796	0.001184	0.00224	0.001936	0.002262	0.004191	0.004023
795	0.000835	0.001784	0.001599	0.002351	0.004334	0.00378
794	0.000897	0.001705	0.001519	0.002275	0.003857	0.003757
793	0.001317	0.001826	0.001798	0.0021	0.004068	0.003752
792	0.001709	0.002326	0.002049	0.00271	0.004416	0.004149
791	0.001121	0.002129	0.001484	0.002155	0.004087	0.003666
790	0.001332	0.002011	0.001525	0.002678	0.00444	0.00404
789	0.001384	0.001745	0.001566	0.002436	0.00396	0.003779
788	0.001022	0.001664	0.00137	0.002054	0.003933	0.004074
787	0.001162	0.001804	0.001353	0.00189	0.003797	0.003817
786	0.001266	0.002023	0.001609	0.002496	0.004193	0.003988
785	0.001123	0.001872	0.00146	0.002276	0.003905	0.003927
784	0.00103	0.001912	0.001727	0.002645	0.004314	0.00387
783	0.001304	0.002071	0.001787	0.00274	0.00449	0.003947
782	0.001337	0.001946	0.001433	0.002374	0.004169	0.004018
781	0.001358	0.002073	0.001753	0.002696	0.004017	0.003831
780	0.001085	0.001788	0.001415	0.002275	0.004164	0.00405
779	0.001108	0.001841	0.00156	0.002364	0.004107	0.003628
778	0.001025	0.0021	0.001382	0.00241	0.004105	0.003835
777	0.000892	0.001899	0.001349	0.002252	0.003993	0.003731
776	0.001035	0.00195	0.001531	0.002228	0.003862	0.0037
775	0.001099	0.002179	0.001706	0.002605	0.00418	0.003919
774	0.00111	0.00221	0.0016	0.002363	0.004075	0.003969
773	0.001261	0.001865	0.001451	0.002383	0.004217	0.004113
772	0.001233	0.002139	0.001681	0.002474	0.004225	0.003974
771	0.000907	0.001845	0.001354	0.002246	0.004068	0.003811
770	0.000942	0.001846	0.001236	0.002016	0.003844	0.003665
769	0.001036	0.001872	0.001715	0.0028	0.004224	0.004103
768	0.00141	0.001981	0.001636	0.002505	0.004119	0.004212
767	0.000787	0.001832	0.001417	0.00238	0.00408	0.003837
766	0.00095	0.002175	0.001627	0.002709	0.004387	0.004179
765	0.00105	0.002006	0.001567	0.002493	0.004213	0.004019
764	0.001302	0.002063	0.0017	0.002515	0.004157	0.004142
763	0.001044	0.002365	0.001567	0.002418	0.004262	0.004077
762	0.001101	0.002068	0.001442	0.002571	0.00423	0.004128
761	0.0015	0.002328	0.001651	0.002498	0.00445	0.004412
760	0.001127	0.002151	0.001668	0.00254	0.004413	0.004132
759	0.001255	0.002178	0.001638	0.002777	0.004473	0.004082
758	0.001247	0.002074	0.001652	0.002409	0.004471	0.004144
757	0.001196	0.002267	0.001768	0.002716	0.004407	0.004272

EXCLI Journal 2020;19:48-70 – ISSN 1611-2156
Received: October 03, 2019, accepted: December 19, 2019, published: January 03, 2020

Wavelength (nm)	Absorption intensity					
	concentration of CT-DNA (M)					
	A	*B*	*C*	*D*	*E*	*F*
	0.00	$2.66x10^{-5}$	$6.21x10^{-5}$	$7.98x10^{-5}$	$1.33x10^{-4}$	$1.73x10^{-4}$
756	0.001319	0.002122	0.001663	0.002749	0.004236	0.004271
755	0.001094	0.002153	0.001719	0.002671	0.004449	0.004426
754	0.001076	0.002069	0.001814	0.002583	0.004322	0.00426
753	0.001227	0.001916	0.001612	0.002633	0.004386	0.004196
752	0.001054	0.002163	0.00164	0.002534	0.004431	0.004241
751	0.00111	0.002422	0.001757	0.002737	0.004405	0.004455
750	0.00125	0.002097	0.001542	0.002516	0.004157	0.004116
749	0.001131	0.002153	0.001714	0.00272	0.004494	0.004303
748	0.001248	0.002403	0.001687	0.002685	0.004494	0.004508
747	0.001061	0.002185	0.001846	0.002669	0.004422	0.004103
746	0.0011	0.002008	0.001603	0.002541	0.00435	0.004115
745	0.001141	0.002203	0.001745	0.002819	0.004312	0.004362
744	0.001043	0.002239	0.001633	0.002673	0.004556	0.00425
743	0.001158	0.002169	0.001667	0.002793	0.00446	0.004374
742	0.00124	0.002139	0.001775	0.002765	0.00436	0.004314
741	0.000951	0.002165	0.001627	0.002555	0.004229	0.004341
740	0.00115	0.002153	0.00174	0.002736	0.004538	0.004392
739	0.001422	0.002467	0.001824	0.003024	0.004653	0.004513
738	0.001124	0.002375	0.001832	0.002911	0.004649	0.004434
737	0.001286	0.002489	0.00199	0.00291	0.004609	0.004562
736	0.001303	0.002438	0.001853	0.002992	0.004732	0.004525
735	0.00159	0.002587	0.001995	0.00319	0.004846	0.004816
734	0.001436	0.002769	0.002136	0.003044	0.004829	0.004713
733	0.0014	0.002793	0.002189	0.003222	0.005039	0.005068
732	0.001577	0.002979	0.002328	0.003471	0.005031	0.005073
731	0.001639	0.002676	0.002346	0.003551	0.005289	0.005165
730	0.00155	0.002817	0.002215	0.003342	0.005094	0.004942
729	0.001896	0.002941	0.002411	0.00359	0.005302	0.005223
728	0.001906	0.002985	0.002427	0.003555	0.005332	0.005278
727	0.001731	0.002868	0.002355	0.003598	0.005291	0.00527
726	0.001778	0.00311	0.002472	0.003579	0.005345	0.00533
725	0.001923	0.003243	0.002714	0.003788	0.005534	0.005528
724	0.001974	0.003295	0.002543	0.003642	0.005378	0.005341
723	0.002081	0.003341	0.002656	0.003919	0.005659	0.005476
722	0.002161	0.003378	0.002822	0.00406	0.005652	0.005553
721	0.002229	0.00326	0.002646	0.003844	0.005557	0.005482
720	0.002094	0.003479	0.002691	0.003939	0.005634	0.005504
719	0.002109	0.003392	0.002699	0.00403	0.005497	0.005747
718	0.002265	0.003343	0.002962	0.003979	0.005806	0.005671
717	0.002186	0.003491	0.002912	0.00395	0.005753	0.005689
716	0.002194	0.003439	0.002809	0.004057	0.005808	0.005733
715	0.002342	0.003759	0.003032	0.00441	0.005999	0.005993
714	0.00235	0.00362	0.003005	0.004314	0.005903	0.005867
713	0.002217	0.003817	0.002967	0.004312	0.006015	0.006002
712	0.002412	0.003769	0.003	0.004357	0.005929	0.006089
711	0.002547	0.004008	0.003384	0.00456	0.006096	0.006008
710	0.002562	0.003996	0.003309	0.004642	0.006184	0.006286
709	0.002385	0.00386	0.003177	0.004411	0.00602	0.006092

EXCLI Journal 2020;19:48-70 – ISSN 1611-2156
Received: October 03, 2019, accepted: December 19, 2019, published: January 03, 2020

Wavelength (nm)	***Absorption intensity*** concentration of CT-DNA (M)					
	A	*B*	*C*	*D*	*E*	*F*
	0.00	2.66x10^{-5}	6.21x10^{-5}	7.98x10^{-5}	1.33x10^{-4}	1.73x10^{-4}
708	0.002434	0.003987	0.003169	0.004427	0.006107	0.006213
707	0.002758	0.004218	0.003573	0.004759	0.006383	0.006457
706	0.002781	0.004247	0.003474	0.004648	0.00673	0.006424
705	0.002716	0.004151	0.003472	0.00478	0.006437	0.00648
704	0.002682	0.004189	0.003539	0.004681	0.006459	0.006572
703	0.002955	0.004286	0.003714	0.004902	0.006676	0.00672
702	0.002745	0.004455	0.003836	0.004866	0.006757	0.006811
701	0.003091	0.004498	0.003726	0.004833	0.006642	0.00677
700	0.00293	0.004447	0.00372	0.005106	0.006792	0.006866
699	0.002876	0.004465	0.003757	0.005082	0.006824	0.006905
698	0.003087	0.004691	0.003735	0.005191	0.006845	0.007012
697	0.002983	0.004445	0.003701	0.005063	0.006878	0.006763
696	0.003186	0.004673	0.00399	0.005229	0.006915	0.007028
695	0.003172	0.004756	0.003909	0.00539	0.006901	0.006956
694	0.003181	0.00491	0.00428	0.005616	0.007262	0.007241
693	0.003361	0.004944	0.004137	0.005577	0.007225	0.007232
692	0.00336	0.004805	0.004133	0.005464	0.007171	0.007279
691	0.003396	0.004928	0.004146	0.005776	0.00744	0.007471
690	0.003596	0.004951	0.004231	0.005644	0.007457	0.007467
689	0.003453	0.005246	0.004377	0.005784	0.007381	0.007692
688	0.003627	0.005288	0.004461	0.005994	0.007611	0.007628
687	0.003686	0.005289	0.004363	0.005907	0.007568	0.007722
686	0.003827	0.005385	0.004409	0.005861	0.007601	0.007825
685	0.00387	0.005342	0.00453	0.006083	0.007783	0.007828
684	0.003829	0.005457	0.004699	0.00604	0.008001	0.007965
683	0.003979	0.005674	0.00486	0.006359	0.008084	0.008126
682	0.00409	0.005766	0.004966	0.006375	0.008137	0.008063
681	0.003984	0.005696	0.004999	0.006442	0.008247	0.00827
680	0.003989	0.005549	0.004808	0.006403	0.008097	0.008215
679	0.004209	0.005906	0.005118	0.006635	0.008506	0.008415
678	0.004208	0.005976	0.00506	0.00678	0.008345	0.00846
677	0.004146	0.00602	0.005315	0.006732	0.008626	0.008544
676	0.004338	0.005968	0.005365	0.006718	0.008499	0.0085
675	0.004438	0.006115	0.005365	0.006945	0.00879	0.008507
674	0.004715	0.006372	0.00555	0.007216	0.008912	0.008892
673	0.004545	0.006462	0.005548	0.00716	0.008828	0.008984
672	0.004607	0.006325	0.005559	0.007096	0.008985	0.008927
671	0.004822	0.006544	0.005614	0.007384	0.009083	0.009062
670	0.004896	0.006632	0.005746	0.007452	0.009258	0.009312
669	0.005049	0.006903	0.005913	0.007704	0.00944	0.009503
668	0.005011	0.006846	0.005966	0.007616	0.009381	0.009445
667	0.005187	0.006969	0.006053	0.007775	0.00952	0.009655
666	0.005222	0.007203	0.006182	0.007794	0.00958	0.009671
665	0.005236	0.007158	0.00625	0.008016	0.009818	0.009824
664	0.00529	0.007236	0.006448	0.008154	0.009983	0.009852
663	0.005418	0.007355	0.006422	0.008128	0.010094	0.009924
662	0.005542	0.007547	0.006671	0.008408	0.010277	0.010163
661	0.005598	0.007504	0.00664	0.008415	0.010097	0.010256

EXCLI Journal 2020;19:48-70 – ISSN 1611-2156
Received: October 03, 2019, accepted: December 19, 2019, published: January 03, 2020

Wavelength (nm)	Absorption intensity					
	concentration of CT-DNA (M)					
	A	B	C	D	E	F
	0.00	2.66x10^{-5}	6.21x10^{-5}	7.98x10^{-5}	1.33x10^{-4}	1.73x10^{-4}
660	0.005628	0.007647	0.006675	0.008537	0.010476	0.010344
659	0.005686	0.00766	0.006795	0.008659	0.010563	0.010449
658	0.005874	0.007823	0.006893	0.008672	0.010668	0.010479
657	0.006066	0.008178	0.007102	0.009033	0.010812	0.010802
656	0.006129	0.008089	0.007072	0.00907	0.010837	0.010854
655	0.006249	0.00823	0.007266	0.009214	0.011049	0.011054
654	0.006274	0.008429	0.007402	0.009379	0.011272	0.011209
653	0.006564	0.0085	0.007571	0.009532	0.011486	0.011373
652	0.00666	0.008702	0.007696	0.009703	0.01161	0.011426
651	0.006662	0.00872	0.007782	0.009742	0.011615	0.011493
650	0.006762	0.009017	0.007983	0.009941	0.011873	0.011712
649	0.006777	0.00896	0.008009	0.009964	0.011976	0.011905
648	0.007238	0.009158	0.008313	0.010281	0.012176	0.012194
647	0.007106	0.009239	0.008213	0.01037	0.012141	0.012168
646	0.007306	0.009375	0.008375	0.010374	0.012316	0.012406
645	0.007328	0.009557	0.008517	0.010536	0.012515	0.01247
644	0.007612	0.009686	0.008727	0.010881	0.01273	0.012825
643	0.007876	0.009934	0.008918	0.011104	0.012942	0.012984
642	0.00778	0.009808	0.008932	0.011088	0.01309	0.01292
641	0.007839	0.010141	0.009099	0.01132	0.013184	0.013244
640	0.007874	0.010227	0.009196	0.011503	0.013487	0.013246
639	0.008118	0.010273	0.009343	0.011538	0.013581	0.013483
638	0.008241	0.010329	0.009383	0.0117	0.01379	0.013593
637	0.008301	0.010552	0.009581	0.011903	0.013915	0.013744
636	0.008522	0.010672	0.009719	0.012136	0.014248	0.013921
635	0.008787	0.010957	0.009904	0.012314	0.014403	0.014123
634	0.008758	0.010976	0.010011	0.012398	0.014459	0.014182
633	0.008833	0.011129	0.010118	0.012458	0.014491	0.014352
632	0.00931	0.01139	0.010509	0.012931	0.015057	0.014812
631	0.00928	0.011582	0.010419	0.012999	0.01508	0.014757
630	0.009393	0.011844	0.010678	0.013208	0.015273	0.01503
629	0.009511	0.011845	0.01088	0.013288	0.015434	0.015238
628	0.009651	0.012085	0.01115	0.013639	0.015694	0.015424
627	0.009959	0.012399	0.011361	0.013752	0.016037	0.015825
626	0.010119	0.012471	0.011464	0.01389	0.016155	0.015892
625	0.010057	0.012523	0.011629	0.014097	0.016263	0.016048
624	0.010394	0.012815	0.011826	0.014493	0.016546	0.016373
623	0.010493	0.012957	0.011906	0.014558	0.016628	0.016423
622	0.010741	0.013155	0.012139	0.014781	0.016926	0.016709
621	0.010808	0.013173	0.012208	0.014997	0.01705	0.016795
620	0.011049	0.013389	0.012465	0.015176	0.017347	0.017062
619	0.011127	0.013606	0.012609	0.015315	0.017519	0.017144
618	0.011198	0.013615	0.012676	0.015393	0.017556	0.017312
617	0.011455	0.013946	0.012917	0.01569	0.017937	0.017517
616	0.011558	0.014021	0.013029	0.015892	0.018053	0.017788
615	0.011714	0.014099	0.013156	0.015966	0.018162	0.017741
614	0.011893	0.014354	0.013442	0.016349	0.018456	0.017986
613	0.011966	0.014341	0.013359	0.016298	0.018503	0.017956

Absorption intensity						
**Wavelength (nm)**	**concentration of CT-DNA (M)**					
	A	*B*	*C*	*D*	*E*	*F*
	0.00	$2.66x10^{-5}$	$6.21x10^{-5}$	$7.98x10^{-5}$	$1.33x10^{-4}$	$1.73x10^{-4}$
612	0.011921	0.01455	0.01358	0.016442	0.018587	0.01825
611	0.012346	0.014805	0.013869	0.016671	0.018936	0.018431
610	0.012439	0.014948	0.014061	0.016901	0.01908	0.018655
609	0.012556	0.015141	0.014147	0.017079	0.019166	0.018884
608	0.012689	0.015162	0.014282	0.017185	0.019384	0.019044
607	0.012781	0.015344	0.014375	0.01731	0.019504	0.019142
606	0.012935	0.015645	0.014665	0.017513	0.019684	0.019384
605	0.013253	0.015754	0.014768	0.017744	0.019892	0.019683
604	0.013232	0.015898	0.015029	0.0179	0.020046	0.019745
603	0.013434	0.016206	0.014999	0.018021	0.02008	0.019848
602	0.013511	0.016136	0.015129	0.018047	0.020327	0.019892
601	0.013815	0.016363	0.015406	0.018334	0.020537	0.020213
600	0.013672	0.016434	0.015558	0.018454	0.020637	0.020132
599	0.014112	0.016666	0.015863	0.018795	0.020922	0.020536
598	0.014085	0.016727	0.015731	0.018723	0.020948	0.020482
597	0.014385	0.016948	0.015941	0.019083	0.021178	0.020759
596	0.014406	0.017032	0.016078	0.019298	0.021278	0.020886
595	0.014545	0.017181	0.01634	0.019392	0.021462	0.021026
594	0.014731	0.017349	0.016538	0.019539	0.021647	0.021301
593	0.014981	0.017747	0.016833	0.019956	0.021964	0.021583
592	0.015159	0.017767	0.016881	0.019936	0.021979	0.021672
591	0.01524	0.01792	0.016948	0.020087	0.02213	0.021818
590	0.015404	0.018076	0.017261	0.020388	0.022331	0.022076
589	0.015591	0.018298	0.017466	0.020472	0.022536	0.0223
588	0.01583	0.018335	0.017512	0.020594	0.022552	0.022461
587	0.015471	0.018105	0.017276	0.020697	0.022366	0.022555
586	0.015838	0.018589	0.017826	0.021034	0.022815	0.022967
585	0.016191	0.018915	0.018059	0.021086	0.023244	0.022945
584	0.016508	0.019253	0.018306	0.021417	0.023527	0.023292
583	0.016697	0.019378	0.018459	0.021594	0.02358	0.023524
582	0.01688	0.019494	0.018794	0.021899	0.023984	0.023832
581	0.017005	0.019834	0.018855	0.022037	0.02406	0.023922
580	0.017033	0.019745	0.019049	0.022134	0.024192	0.023993
579	0.017251	0.019932	0.019084	0.0224	0.024463	0.024255
578	0.017429	0.020142	0.019472	0.022523	0.024652	0.024392
577	0.017707	0.02031	0.019639	0.022768	0.024871	0.024343
576	0.017809	0.020558	0.019694	0.02253	0.02505	0.024332
575	0.018105	0.02064	0.019919	0.022876	0.025194	0.024571
574	0.018256	0.020795	0.020292	0.023145	0.025582	0.024855
573	0.018235	0.020967	0.020371	0.023117	0.025625	0.024901
572	0.018489	0.021112	0.020431	0.02341	0.025987	0.025141
571	0.018644	0.02138	0.020733	0.023648	0.026006	0.025388
570	0.018849	0.021485	0.020877	0.023789	0.026258	0.025652
569	0.018975	0.021592	0.021011	0.02389	0.026367	0.02576
568	0.019183	0.021742	0.021325	0.024505	0.02652	0.026474
567	0.01933	0.021854	0.021483	0.02461	0.02681	0.026651
566	0.019409	0.021978	0.021575	0.024879	0.026845	0.02673
565	0.019574	0.022161	0.021765	0.025049	0.027056	0.026869

Wavelength (nm)	***Absorption intensity***					
	**concentration of CT-DNA (M)**					
	A	*B*	*C*	*D*	*E*	*F*
	0.00	$2.66x10^{-5}$	$6.21x10^{-5}$	$7.98x10^{-5}$	$1.33x10^{-4}$	$1.73x10^{-4}$
564	0.01973	0.022243	0.02187	0.025264	0.027185	0.02724
563	0.019894	0.022482	0.021971	0.025455	0.027332	0.02736
562	0.01994	0.022483	0.02219	0.02544	0.027465	0.027463
561	0.020115	0.022614	0.02233	0.025555	0.027567	0.02754
560	0.020238	0.022735	0.022482	0.025714	0.027791	0.027927
559	0.020381	0.022755	0.022523	0.025784	0.027842	0.02778
558	0.020595	0.023261	0.022919	0.026156	0.028159	0.02812
557	0.020558	0.023066	0.022862	0.026	0.028089	0.028033
556	0.020537	0.023046	0.022799	0.025916	0.028	0.027972
555	0.020417	0.022944	0.02267	0.02602	0.028039	0.028101
554	0.020526	0.023005	0.02275	0.026064	0.028093	0.028035
553	0.020963	0.023447	0.023128	0.026279	0.028125	0.028155
552	0.021363	0.023787	0.023557	0.026722	0.028649	0.028725
551	0.021366	0.0237	0.023482	0.026884	0.028835	0.028784
550	0.021533	0.023996	0.023819	0.027091	0.029079	0.029076
549	0.021651	0.024219	0.023987	0.027157	0.029225	0.029242
548	0.021738	0.024317	0.02404	0.027312	0.029277	0.029147
547	0.021875	0.024345	0.024254	0.027441	0.029272	0.029368
546	0.021976	0.024559	0.02432	0.027581	0.029558	0.029572
545	0.022257	0.024809	0.024508	0.027678	0.029764	0.029894
544	0.02247	0.024992	0.024829	0.027997	0.029925	0.030066
543	0.022692	0.025155	0.024852	0.028073	0.03012	0.030195
542	0.022527	0.0251	0.025024	0.028136	0.02995	0.030251
541	0.022684	0.025229	0.025173	0.028276	0.030196	0.030363
540	0.023078	0.025653	0.025516	0.028574	0.030514	0.030624
539	0.023155	0.025649	0.025485	0.028768	0.030683	0.030729
538	0.023315	0.025616	0.025681	0.028758	0.030721	0.03092
537	0.023508	0.025862	0.025847	0.028891	0.030859	0.031058
536	0.023568	0.025951	0.025972	0.029055	0.031013	0.031188
535	0.02372	0.026119	0.02598	0.029089	0.031115	0.031249
534	0.023967	0.026328	0.026206	0.029365	0.03144	0.031503
533	0.02414	0.026356	0.026371	0.0295	0.031479	0.031542
532	0.024135	0.026559	0.026485	0.029852	0.031701	0.031656
531	0.024246	0.026764	0.026724	0.029864	0.031819	0.031846
530	0.02439	0.026748	0.026868	0.029923	0.031892	0.031868
529	0.024657	0.026782	0.026958	0.030059	0.032041	0.032165
528	0.024749	0.027006	0.027175	0.030286	0.032244	0.032248
527	0.024897	0.027119	0.027323	0.030256	0.032257	0.032361
526	0.024986	0.027339	0.027327	0.030398	0.032247	0.032456
525	0.025186	0.027569	0.027619	0.030739	0.032485	0.0328
524	0.025175	0.027485	0.027615	0.030722	0.032553	0.032752
523	0.025416	0.027759	0.027859	0.030864	0.032783	0.03299
522	0.025436	0.027817	0.027845	0.030808	0.032632	0.032948
521	0.025747	0.027862	0.028025	0.03089	0.032649	0.032914
520	0.025649	0.027636	0.027827	0.030657	0.03266	0.032945
519	0.025421	0.027806	0.0279	0.030869	0.032777	0.033145
518	0.025687	0.027931	0.028138	0.031003	0.03295	0.033193
517	0.025769	0.027934	0.027995	0.030871	0.032842	0.033297

Wavelength (nm)	***Absorption intensity***					
	**concentration of CT-DNA (M)**					
	A	*B*	*C*	*D*	*E*	*F*
	0.00	$2.66x10^{-5}$	$6.21x10^{-5}$	$7.98x10^{-5}$	$1.33x10^{-4}$	$1.73x10^{-4}$
516	0.025599	0.027839	0.027992	0.031009	0.032884	0.033382
515	0.026031	0.028143	0.028451	0.031306	0.033146	0.033505
514	0.025946	0.028103	0.028284	0.031092	0.033051	0.033416
513	0.026139	0.028267	0.028418	0.031458	0.033378	0.033658
512	0.02622	0.02832	0.028442	0.031546	0.033307	0.033765
511	0.026454	0.028517	0.028667	0.031663	0.033629	0.033681
510	0.026441	0.028559	0.028785	0.031684	0.033537	0.033862
509	0.026722	0.028704	0.029108	0.032013	0.033716	0.034084
508	0.026756	0.028695	0.028928	0.031901	0.033808	0.034003
507	0.026764	0.028654	0.028971	0.031985	0.033872	0.033986
506	0.026696	0.028724	0.029004	0.032048	0.033732	0.034227
505	0.026888	0.028805	0.029104	0.031968	0.033767	0.034174
504	0.026942	0.029097	0.029171	0.03214	0.033894	0.034418
503	0.027015	0.029139	0.029347	0.03221	0.034025	0.034392
502	0.027295	0.029187	0.029374	0.032375	0.034241	0.034645
501	0.027201	0.029304	0.029446	0.032378	0.034209	0.03479
500	0.02744	0.02966	0.029688	0.032659	0.034462	0.035024
499	0.027472	0.029515	0.029652	0.032597	0.034371	0.034961
498	0.027524	0.029602	0.029757	0.032676	0.034559	0.03503
497	0.027506	0.02961	0.029613	0.032465	0.034529	0.035017
496	0.027544	0.029546	0.029894	0.032637	0.034575	0.035075
495	0.027549	0.029513	0.029715	0.032559	0.034343	0.034877
494	0.027551	0.02958	0.029792	0.032639	0.03446	0.035099
493	0.027639	0.029699	0.029833	0.032679	0.034503	0.03503
492	0.027744	0.029705	0.029972	0.032923	0.034712	0.035143
491	0.02785	0.029805	0.029946	0.03291	0.034721	0.035264
490	0.028012	0.029772	0.030021	0.032891	0.03466	0.035206
489	0.028077	0.029926	0.030046	0.033115	0.034803	0.035465
488	0.028099	0.029934	0.030064	0.033008	0.034768	0.03524
487	0.028423	0.029856	0.030136	0.033022	0.034801	0.035401
486	0.028428	0.030124	0.030355	0.032973	0.034773	0.035443
485	0.028477	0.030334	0.030463	0.033188	0.034849	0.035498
484	0.028434	0.030459	0.030604	0.033299	0.035048	0.035545
483	0.028538	0.030599	0.030517	0.033337	0.035229	0.035919
482	0.028487	0.03048	0.030314	0.033303	0.035132	0.035854
481	0.028514	0.030401	0.030511	0.033401	0.035195	0.035885
480	0.028508	0.030512	0.030481	0.033297	0.035165	0.036052
479	0.028479	0.030367	0.030317	0.033346	0.035123	0.036037
478	0.028572	0.030461	0.030486	0.033371	0.035283	0.035983
477	0.028486	0.030393	0.030398	0.033234	0.035135	0.035777
476	0.028415	0.03046	0.030362	0.033336	0.03495	0.035584
475	0.028469	0.030226	0.030388	0.032946	0.034798	0.035597
474	0.028015	0.029877	0.02988	0.032832	0.03468	0.035352
473	0.028039	0.029969	0.029974	0.032867	0.034708	0.035541
472	0.02786	0.029763	0.029752	0.032869	0.034658	0.035335
471	0.027967	0.029752	0.029829	0.03278	0.034693	0.035411
470	0.027908	0.029233	0.029746	0.032676	0.034636	0.035335
469	0.027777	0.029595	0.02966	0.032598	0.034481	0.035197

Wavelength (nm)	Absorption intensity					
	concentration of CT-DNA (M)					
	A	*B*	*C*	*D*	*E*	*F*
	0.00	$2.66x10^{-5}$	$6.21x10^{-5}$	$7.98x10^{-5}$	$1.33x10^{-4}$	$1.73x10^{-4}$
468	0.02802	0.030028	0.029781	0.032916	0.03464	0.035489
467	0.028325	0.030032	0.02994	0.032917	0.034902	0.035758
466	0.028077	0.029952	0.02981	0.032918	0.034568	0.035456
465	0.028076	0.02991	0.02975	0.032742	0.034616	0.035434
464	0.027904	0.029811	0.029715	0.03271	0.03446	0.035575
463	0.027921	0.029748	0.02963	0.032658	0.034483	0.035354
462	0.027919	0.029865	0.029606	0.032508	0.034418	0.035353
461	0.027871	0.029799	0.029494	0.03249	0.034229	0.035315
460	0.027725	0.029665	0.029499	0.03242	0.034186	0.035346
459	0.027718	0.029628	0.029328	0.032425	0.034324	0.035231
458	0.027531	0.029538	0.02939	0.032349	0.034051	0.035229
457	0.027497	0.029492	0.029114	0.03222	0.033907	0.035077
456	0.027665	0.02951	0.029067	0.032223	0.03412	0.035212
455	0.027351	0.029281	0.028889	0.032004	0.033978	0.034929
454	0.027132	0.029254	0.028892	0.031951	0.033827	0.034853
453	0.027249	0.029136	0.028874	0.032055	0.0338	0.034813
452	0.027182	0.02919	0.028675	0.031846	0.033549	0.034794
451	0.026824	0.028829	0.028459	0.03166	0.033318	0.034479
450	0.027038	0.028922	0.028668	0.031626	0.033666	0.034523
449	0.026808	0.028946	0.028257	0.031511	0.033212	0.034412
448	0.02683	0.028922	0.02832	0.031502	0.033412	0.034404
447	0.026663	0.028774	0.028254	0.03137	0.033272	0.034262
446	0.026558	0.028734	0.028141	0.031445	0.033118	0.034279
445	0.026476	0.028667	0.027978	0.031273	0.033058	0.034213
444	0.026496	0.028584	0.028118	0.031187	0.032968	0.034197
443	0.02632	0.028476	0.02783	0.031006	0.032845	0.03411
442	0.026146	0.028352	0.027677	0.030951	0.032767	0.034057
441	0.026027	0.028206	0.02757	0.030815	0.032671	0.033848
440	0.026011	0.028155	0.027416	0.030726	0.032462	0.033925
439	0.025759	0.028197	0.027274	0.030483	0.032513	0.033753
438	0.025699	0.028044	0.027154	0.030326	0.032369	0.033645
437	0.025865	0.02805	0.027109	0.030428	0.032374	0.033749
436	0.025456	0.027817	0.026869	0.030437	0.032301	0.033424
435	0.025472	0.027721	0.026924	0.030153	0.032095	0.033568
434	0.025238	0.027475	0.026667	0.030031	0.032035	0.033269
433	0.025181	0.027443	0.026526	0.029959	0.031951	0.033289
432	0.025041	0.027395	0.026588	0.029858	0.031822	0.033186
431	0.024946	0.027422	0.026581	0.029969	0.031835	0.033206
430	0.024738	0.027172	0.026315	0.029608	0.031565	0.032916
429	0.024635	0.027002	0.026147	0.0296	0.031544	0.032967
428	0.024641	0.027018	0.026088	0.029577	0.031476	0.032794
427	0.024602	0.027021	0.02606	0.029442	0.031588	0.03271
426	0.024397	0.026913	0.025884	0.029338	0.031289	0.032715
425	0.024361	0.026937	0.025896	0.029334	0.031138	0.032608
424	0.024263	0.026687	0.025722	0.0292	0.031025	0.032685
423	0.024297	0.026945	0.025803	0.029289	0.031109	0.032783
422	0.024191	0.026646	0.025694	0.029089	0.030973	0.032493
421	0.024248	0.026692	0.025603	0.029229	0.030993	0.032716

Wavelength (nm)	Absorption intensity					
	concentration of CT-DNA (M)					
	A	*B*	*C*	*D*	*E*	*F*
	0.00	$2.66x10^{-5}$	$6.21x10^{-5}$	$7.98x10^{-5}$	$1.33x10^{-4}$	$1.73x10^{-4}$
420	0.023968	0.026658	0.025655	0.029079	0.030908	0.032628
419	0.024004	0.026596	0.02551	0.02884	0.031032	0.032637
418	0.023809	0.026598	0.025366	0.028909	0.030944	0.032659
417	0.023886	0.026506	0.025225	0.028745	0.030809	0.032579
416	0.023789	0.026471	0.025292	0.028788	0.030746	0.032494
415	0.023778	0.026528	0.025315	0.028804	0.031078	0.032503
414	0.023454	0.026305	0.02513	0.028658	0.030776	0.032516
413	0.023521	0.026563	0.025224	0.028607	0.030649	0.032409
412	0.023508	0.026342	0.025061	0.028571	0.030789	0.032464
411	0.023438	0.026144	0.024814	0.028522	0.03056	0.032271
410	0.023384	0.026153	0.024998	0.028576	0.030639	0.03235
409	0.023461	0.025952	0.024972	0.028365	0.030458	0.03184
408	0.023033	0.02586	0.024498	0.028211	0.030159	0.032225
407	0.022788	0.025714	0.024602	0.028236	0.030135	0.031782
406	0.023122	0.026141	0.024615	0.02845	0.030363	0.032153
405	0.023316	0.026281	0.025034	0.02865	0.03059	0.032505
404	0.023536	0.026646	0.025202	0.028833	0.030732	0.032842
403	0.023401	0.026521	0.025062	0.028847	0.030753	0.032667
402	0.023593	0.026503	0.025269	0.028899	0.030914	0.03293
401	0.023383	0.026461	0.025132	0.028777	0.030909	0.032849
400	0.023306	0.026279	0.025029	0.028732	0.030696	0.032768
399	0.023514	0.02651	0.025251	0.02884	0.030879	0.032958
398	0.023535	0.026796	0.025187	0.029028	0.031073	0.033162
397	0.02342	0.026683	0.025131	0.028808	0.0311	0.033039
396	0.023835	0.026974	0.025553	0.029272	0.031413	0.033426
395	0.023691	0.026867	0.025545	0.029261	0.031373	0.033406
394	0.023969	0.027005	0.025738	0.029413	0.031518	0.033554
393	0.024154	0.027272	0.025778	0.02949	0.031746	0.033802
392	0.024064	0.027407	0.025865	0.029593	0.031535	0.034006
391	0.024666	0.027124	0.026124	0.029759	0.032061	0.033708
390	0.023571	0.026775	0.025395	0.029187	0.031318	0.033535
389	0.024175	0.027605	0.026152	0.030004	0.032227	0.034193
388	0.02438	0.027565	0.026409	0.029974	0.032243	0.034289
387	0.02474	0.027714	0.026194	0.030195	0.032185	0.034487
386	0.024591	0.027798	0.02625	0.030281	0.032401	0.034535
385	0.024934	0.028068	0.026865	0.030228	0.03265	0.034857
384	0.024568	0.028016	0.026528	0.030294	0.032512	0.034919
383	0.025009	0.028355	0.026988	0.030807	0.033014	0.035085
382	0.025528	0.028685	0.027206	0.031078	0.033163	0.035826
381	0.025531	0.028887	0.027281	0.031428	0.033577	0.035937
380	0.0257	0.029058	0.027333	0.031403	0.03366	0.036084
379	0.025714	0.028811	0.027611	0.031533	0.033657	0.036446
378	0.025989	0.02954	0.027908	0.032021	0.033895	0.036432
377	0.025946	0.029214	0.027704	0.031997	0.033683	0.036646
376	0.026262	0.029535	0.028164	0.031981	0.034316	0.036748
375	0.026082	0.029583	0.027995	0.032051	0.034361	0.036827
374	0.026183	0.029354	0.027987	0.032211	0.034301	0.036727
373	0.026657	0.029929	0.028304	0.032721	0.034885	0.037357

EXCLI Journal 2020;19:48-70 – ISSN 1611-2156
Received: October 03, 2019, accepted: December 19, 2019, published: January 03, 2020

Wavelength (nm)	Absorption intensity					
	concentration of CT-DNA (M)					
	A	*B*	*C*	*D*	*E*	*F*
	0.00	2.66x10^{-5}	6.21x10^{-5}	7.98x10^{-5}	1.33x10^{-4}	1.73x10^{-4}
372	0.026692	0.03027	0.028921	0.032821	0.035639	0.037587
371	0.026355	0.029686	0.028356	0.032873	0.034735	0.037739
370	0.026552	0.030236	0.028305	0.033096	0.035291	0.037727
369	0.027	0.030029	0.028473	0.032965	0.035377	0.038057
368	0.027583	0.030988	0.029817	0.033592	0.035833	0.038495
367	0.026904	0.030456	0.029014	0.033424	0.035514	0.038235
366	0.027243	0.031095	0.029224	0.033874	0.036019	0.038753
365	0.027402	0.030976	0.02971	0.033847	0.036376	0.038962
364	0.027287	0.031564	0.029628	0.033846	0.03664	0.039047
363	0.027621	0.031452	0.030234	0.034119	0.036522	0.038934
362	0.02853	0.032266	0.031279	0.035355	0.037511	0.040228
361	0.026385	0.030259	0.028499	0.032994	0.035441	0.038038
360	0.027961	0.031819	0.030508	0.034322	0.037194	0.039572
359	0.028494	0.032277	0.030859	0.035324	0.037216	0.040082
358	0.028509	0.033124	0.030783	0.03577	0.03813	0.040271
357	0.028864	0.033309	0.031966	0.036186	0.038865	0.041032
356	0.02622	0.03077	0.029297	0.032805	0.035091	0.038556
355	0.029122	0.032784	0.031286	0.035889	0.038469	0.041088
354	0.028874	0.032926	0.031713	0.035926	0.038251	0.041825
353	0.029965	0.033677	0.032737	0.036226	0.038852	0.040887
352	0.029836	0.033564	0.031366	0.036773	0.03939	0.041096
351	0.02895	0.033745	0.032092	0.037153	0.040638	0.041869
350	0.029534	0.034393	0.033026	0.037275	0.03947	0.042118
349	0.030952	0.034988	0.036458	0.038655	0.043705	0.04464
348	0.030212	0.034487	0.032916	0.037188	0.039378	0.042309
347	0.030263	0.034829	0.033369	0.037696	0.040286	0.043081
346	0.0302	0.035325	0.033845	0.038033	0.040543	0.043412
345	0.031443	0.035886	0.034177	0.038554	0.041394	0.044348
344	0.031674	0.036165	0.034155	0.03944	0.041695	0.044904
343	0.031668	0.036497	0.034407	0.038926	0.041975	0.044797
342	0.03143	0.036194	0.034795	0.039464	0.041721	0.045011
341	0.031779	0.036695	0.034843	0.039702	0.04248	0.045305
340	0.032485	0.037226	0.034944	0.040093	0.042722	0.046174
339	0.032645	0.037168	0.035536	0.040231	0.04295	0.045917
338	0.032852	0.037868	0.035961	0.040802	0.043597	0.046556
337	0.032486	0.038439	0.036244	0.040742	0.043604	0.046845
336	0.033639	0.038844	0.036586	0.041403	0.04458	0.047899
335	0.033758	0.039296	0.036543	0.042054	0.044972	0.04787
334	0.034122	0.039221	0.037026	0.042099	0.045157	0.04816
333	0.035002	0.039999	0.038018	0.042797	0.045535	0.048772
332	0.035441	0.040359	0.038654	0.044146	0.046814	0.05018
331	0.035492	0.040747	0.038717	0.044261	0.046856	0.050287
330	0.036397	0.041687	0.039423	0.045015	0.047845	0.051079
329	0.036445	0.041994	0.039757	0.045093	0.048558	0.051305
328	0.036803	0.042501	0.039648	0.045821	0.04856	0.051585
327	0.037901	0.043965	0.041498	0.047055	0.049862	0.053176
326	0.038168	0.043719	0.041323	0.047161	0.050301	0.05339
325	0.038905	0.044736	0.041848	0.048263	0.050697	0.054405

Wavelength (nm)	***Absorption intensity***					
	**concentration of CT-DNA (M)**					
	A	*B*	*C*	*D*	*E*	*F*
	0.00	$2.66x10^{-5}$	$6.21x10^{-5}$	$7.98x10^{-5}$	$1.33x10^{-4}$	$1.73x10^{-4}$
324	0.039425	0.04513	0.042414	0.04845	0.051558	0.055006
323	0.039927	0.045114	0.043342	0.049239	0.052293	0.055193
322	0.040617	0.046484	0.044483	0.049947	0.053296	0.056324
321	0.041596	0.046658	0.044529	0.050913	0.054229	0.057453
320	0.04177	0.048064	0.045748	0.051872	0.055201	0.058056
319	0.043243	0.048678	0.045989	0.052538	0.05571	0.059167
318	0.043362	0.049122	0.046887	0.053334	0.056588	0.059864
317	0.04398	0.049827	0.04763	0.053911	0.057392	0.060618
316	0.0443	0.050553	0.048233	0.054778	0.058335	0.061706
315	0.04561	0.05145	0.049101	0.056032	0.059526	0.062843
314	0.046221	0.05213	0.049839	0.056635	0.060246	0.063846
313	0.046716	0.052934	0.050546	0.057632	0.061271	0.064757
312	0.047929	0.054036	0.051674	0.058635	0.06224	0.065763
311	0.048141	0.054828	0.052388	0.059583	0.063859	0.067155
310	0.049222	0.055564	0.053458	0.061073	0.064458	0.068637
309	0.050705	0.056535	0.054906	0.062632	0.066959	0.071227
308	0.051819	0.057655	0.055789	0.064426	0.068651	0.072539
307	0.052898	0.0584	0.056811	0.066115	0.070463	0.074782
306	0.053921	0.059828	0.059056	0.068567	0.073037	0.077492
305	0.05547	0.060881	0.060253	0.070975	0.076273	0.080753
304	0.056353	0.061494	0.061745	0.073163	0.079235	0.084012
303	0.057505	0.062325	0.063368	0.076866	0.083485	0.088677
302	0.059142	0.063218	0.06555	0.080527	0.088035	0.094374
301	0.060627	0.064305	0.067923	0.085059	0.094117	0.101007
300	0.062512	0.064595	0.069765	0.090673	0.100772	0.109008
299	0.063963	0.06543	0.072844	0.097402	0.109719	0.118752
298	0.06706	0.066436	0.077186	0.105684	0.120518	0.131277
297	0.06904	0.067332	0.08105	0.115538	0.133031	0.145703
296	0.072697	0.067975	0.085582	0.127681	0.148063	0.162365
295	0.075704	0.068882	0.09175	0.140977	0.16522	0.182492
294	0.07953	0.069309	0.097607	0.156223	0.184962	0.205594
293	0.084043	0.070536	0.104913	0.174072	0.207617	0.231807
292	0.08845	0.071261	0.112626	0.192598	0.232676	0.259189
291	0.09298	0.071827	0.120759	0.213168	0.258852	0.290502
290	0.098448	0.072807	0.129929	0.235141	0.286898	0.322561
289	0.104031	0.073709	0.139493	0.258044	0.3171	0.356945
288	0.109728	0.074581	0.149174	0.281525	0.347398	0.39189
287	0.115725	0.074916	0.1597	0.306037	0.379755	0.429099
286	0.122024	0.075833	0.170346	0.33203	0.412684	0.466006
285	0.128258	0.076165	0.180176	0.358519	0.447349	0.506522
284	0.134996	0.077054	0.192072	0.385287	0.481975	0.545869
283	0.141066	0.077609	0.20255	0.412104	0.516803	0.585865
282	0.148	0.078775	0.21409	0.43887	0.550998	0.62461
281	0.15434	0.079323	0.225117	0.464108	0.584528	0.662823
280	0.160602	0.07972	0.23576	0.489012	0.616255	0.69864
279	0.166658	0.080657	0.246588	0.512947	0.647551	0.733625
278	0.173006	0.081537	0.257072	0.537261	0.677743	0.769243
277	0.178749	0.081747	0.267373	0.561552	0.70948	0.804534

Wavelength (nm)	***Absorption intensity***					
	**concentration of CT-DNA (M)**					
	A	*B*	*C*	*D*	*E*	*F*
	0.00	$2.66x10^{-5}$	$6.21x10^{-5}$	$7.98x10^{-5}$	$1.33x10^{-4}$	$1.73x10^{-4}$
276	0.184894	0.082708	0.277598	0.585445	0.740702	0.840023
275	0.191653	0.08373	0.288436	0.610438	0.772568	0.876503
274	0.197619	0.084908	0.298732	0.634865	0.80351	0.912164
273	0.203731	0.085947	0.309256	0.659024	0.835251	0.948383
272	0.21013	0.087263	0.319196	0.683094	0.865544	0.982856
271	0.216007	0.088283	0.329333	0.705562	0.895456	1.016344
270	0.222013	0.089853	0.339308	0.727712	0.923912	1.04906
269	0.227701	0.09114	0.348305	0.748163	0.9503	1.078141
268	0.233253	0.092739	0.356994	0.767555	0.975547	1.107287
267	0.238633	0.094684	0.365563	0.787418	1.001087	1.135174
266	0.243974	0.096376	0.373482	0.805565	1.023903	1.162562
265	0.249602	0.09868	0.381954	0.823607	1.04682	1.188389
264	0.255129	0.101275	0.389743	0.840893	1.069314	1.213619
263	0.259909	0.103376	0.396803	0.856931	1.089301	1.235727
262	0.264749	0.10578	0.403885	0.871966	1.107731	1.258677
261	0.269648	0.108318	0.410043	0.884374	1.123618	1.276243
260	0.273863	0.111294	0.415552	0.895282	1.135823	1.289846
259	0.277907	0.114004	0.420301	0.90231	1.145694	1.29998
258	0.281462	0.116722	0.424166	0.907324	1.151431	1.306298
257	0.284446	0.120034	0.426526	0.909809	1.153244	1.307638
256	0.287632	0.123762	0.42888	0.910501	1.153317	1.306913
255	0.290615	0.128151	0.430758	0.90934	1.149382	1.30211
254	0.293416	0.13274	0.432311	0.905867	1.144108	1.295463
253	0.296982	0.138529	0.433862	0.901209	1.13604	1.285768
252	0.300573	0.144537	0.434659	0.894138	1.124597	1.272536
251	0.304378	0.151184	0.435965	0.88576	1.111021	1.2558
250	0.308759	0.15976	0.437249	0.876134	1.095303	1.236063
249	0.314025	0.169093	0.438822	0.864152	1.076748	1.21361
248	0.320291	0.179657	0.441317	0.852607	1.058622	1.190533
247	0.32867	0.193348	0.445823	0.84233	1.040863	1.167782
246	0.339536	0.208714	0.45231	0.833668	1.024434	1.146984
245	0.354748	0.228565	0.462624	0.828597	1.011999	1.128818
244	0.374943	0.253687	0.478989	0.828956	1.004417	1.117151
243	0.403148	0.286248	0.502728	0.837096	1.005764	1.112704
242	0.441405	0.328639	0.53656	0.856677	1.017593	1.120713
241	0.495102	0.386545	0.587127	0.892544	1.046789	1.145852
240	0.56967	0.46404	0.658008	0.950236	1.098495	1.193522
239	0.674575	0.570841	0.759577	1.040143	1.182508	1.273335
238	0.817544	0.71409	0.89967	1.169373	1.307695	1.395604
237	1.015799	0.911942	1.09628	1.357377	1.492748	1.578788
236	1.279725	1.173735	1.358565	1.612595	1.74542	1.830461
235	1.634377	1.523364	1.713663	1.961464	2.094651	2.177525
234	2.090257	1.973063	2.169008	2.415295	2.547332	2.630786
233	2.671529	2.544373	2.750385	2.994664	3.132935	3.217333
232	3.337032	3.202805	3.419721	3.662337	3.79848	3.894347
231	3.896622	3.790447	4.006674	4.215291	4.393512	4.435766
230	4.197876	4.111913	4.255548	4.537149	4.641823	4.767568
229	4.390282	4.282135	4.494154	4.72946	4.810856	4.991961

EXCLI Journal 2020;19:48-70 – ISSN 1611-2156
Received: October 03, 2019, accepted: December 19, 2019, published: January 03, 2020

Wavelength (nm)	***Absorption intensity***					
	**concentration of CT-DNA (M)**					
	A	*B*	*C*	*D*	*E*	*F*
	0.00	$2.66x10^{-5}$	$6.21x10^{-5}$	$7.98x10^{-5}$	$1.33x10^{-4}$	$1.73x10^{-4}$
228	4.58995	4.451343	4.635842	4.832185	4.9992	5.226411
227	4.681111	4.624986	4.825832	5.084306	5.163124	5.195923
226	4.868775	4.758604	4.768928	5.098868	5.296166	5.254354
225	4.901201	4.809526	4.982412	5.258967	5.661831	5.549585
224	5.058378	4.955067	5.120461	5.452831	5.365791	5.167914
223	5.12045	4.962563	5.333961	5.461137	10	5.357339
222	5.095037	4.968455	5.269636	5.212405	5.635222	5.26353
221	5.084231	5.130528	5.614175	5.575775	10	5.900236
220	5.035283	5.022135	5.285655	5.410039	6.063098	6.063089
219	6.048215	5.981443	5.506639	5.295033	5.712262	5.680011
218	5.633462	5.253579	5.422777	5.663742	5.663163	5.855
217	5.468562	5.28576	5.470586	5.392947	6.188342	10
216	5.116684	5.014415	5.62545	5.815487	5.371754	5.37143
215	5.793527	4.999887	5.302686	10	6.270225	6.747462
214	5.21879	5.292801	5.404017	6.723894	5.468602	10
213	5.355489	6.698282	5.854958	5.317556	10	5.521363
212	10	5.590065	10	10	10	10
211	5.859901	5.683853	5.380955	10	10	10
210	10	10	5.370221	10	10	10
209	5.467574	5.291465	5.297412	5.882406	10	5.182883
208	5.268338	5.783298	5.295432	10	5.425597	5.978724
207	5.752798	5.497459	10	6.053363	5.336651	6.274644
206	10	5.401189	10	5.087515	5.876538	10
205	10	10	10	10	10	10
204	4.977557	5.005319	10	4.951512	5.258813	4.941694
203	10	10	4.8651	10	10	10
202	4.365524	4.518612	10	4.583596	5.369644	4.66358
201	3.860486	4.006576	3.867528	4.106519	4.548669	4.313734
200	3.299556	3.216929	3.45045	3.23949	3.507171	3.284147

EXCLI Journal 2020;19:48-70 – ISSN 1611-2156
Received: October 03, 2019, accepted: December 19, 2019, published: January 03, 2020

Table 4: Absorption intensity in the wavelength range of 200-800 nm for absorption spectra of different concentration of CT-DNA

Wavelength (nm)	***Absorption intensity***					
	**concentration of CT-DNA (M)**					
	A	*B*	*C*	*D*	*E*	*F*
	0.00	$2.66x10^{-5}$	$6.21x10^{-5}$	$7.98x10^{-5}$	$1.33x10^{-4}$	$1.73x10^{-4}$
800	0.000433	0.00116	0.001862	0.001084	0.002374	0.003259
799	0.000161	0.00145	0.001633	0.001111	0.002493	0.003001
798	0.000262	0.001365	0.001588	0.001152	0.002541	0.003188
797	0.000725	0.00151	0.001922	0.001487	0.0027	0.003623
796	0.000725	0.001563	0.001984	0.001283	0.002613	0.003412
795	0.000443	0.001449	0.001716	0.000846	0.002624	0.003155
794	0.000651	0.001263	0.001689	0.001269	0.002442	0.002983
793	0.000595	0.001521	0.001888	0.001303	0.002677	0.003295
792	0.00108	0.001953	0.002202	0.001772	0.003003	0.00353
791	0.000526	0.001557	0.001602	0.00127	0.002252	0.003161
790	0.000707	0.001734	0.001827	0.001461	0.002733	0.003671
789	0.000606	0.001532	0.002094	0.00099	0.002831	0.003045
788	0.0004	0.001517	0.001677	0.001303	0.002721	0.003319
787	0.00027	0.001443	0.001152	0.000961	0.002264	0.002947
786	0.00054	0.001465	0.001776	0.001276	0.002656	0.003241
785	0.000295	0.001363	0.00164	0.001208	0.002553	0.003087
784	0.000462	0.001507	0.001886	0.001269	0.002715	0.003045
783	0.000599	0.001597	0.001778	0.001368	0.002788	0.003337
782	0.000269	0.001282	0.001561	0.001119	0.002784	0.003344
781	0.000602	0.001641	0.001919	0.001362	0.002723	0.003085
780	0.000297	0.001744	0.001729	0.000825	0.002437	0.003004
779	0.000335	0.001227	0.001641	0.000939	0.002296	0.003057
778	0.000251	0.00132	0.001469	0.001198	0.002657	0.002924
777	0.000239	0.001413	0.001659	0.001034	0.002404	0.003158
776	0.000405	0.001369	0.001568	0.001193	0.002638	0.002903
775	0.000506	0.001594	0.001645	0.001094	0.002532	0.003375
774	0.000565	0.001419	0.001608	0.001251	0.002596	0.003369
773	0.000593	0.001504	0.001646	0.001208	0.002541	0.003272
772	0.000475	0.001542	0.00175	0.001128	0.00268	0.00315
771	0.00037	0.001262	0.001435	0.001063	0.002404	0.002798
770	0.000261	0.001198	0.00126	0.001153	0.002357	0.002743
769	0.000474	0.001602	0.001781	0.001375	0.002761	0.003287
768	0.000457	0.001428	0.001761	0.001275	0.002735	0.003015
767	3.31E-05	0.001499	0.001455	0.000992	0.00251	0.002964
766	0.000454	0.001429	0.001724	0.00112	0.002608	0.003257
765	0.000623	0.001414	0.001773	0.001258	0.002457	0.002995
764	0.000452	0.001518	0.001794	0.001398	0.002589	0.003126
763	0.000222	0.00119	0.001746	0.001212	0.002606	0.00328
762	0.000104	0.00138	0.001705	0.001174	0.002451	0.003194
761	0.000634	0.001544	0.001832	0.001399	0.002804	0.002979
760	0.00042	0.001455	0.001713	0.001114	0.002409	0.003104
759	0.000512	0.001373	0.001715	0.001198	0.002677	0.003198
758	0.000383	0.001397	0.001446	0.000972	0.002457	0.003155
757	0.000481	0.001532	0.001704	0.001205	0.002689	0.003198
756	0.00044	0.001517	0.001767	0.001206	0.002677	0.003105

EXCLI Journal 2020;19:48-70 – ISSN 1611-2156
Received: October 03, 2019, accepted: December 19, 2019, published: January 03, 2020

Wavelength (nm)	***Absorption intensity***					
	**concentration of CT-DNA (M)**					
	A	*B*	*C*	*D*	*E*	*F*
	0.00	$2.66x10^{-5}$	$6.21x10^{-5}$	$7.98x10^{-5}$	$1.33x10^{-4}$	$1.73x10^{-4}$
755	0.000594	0.001734	0.001936	0.001199	0.002918	0.003351
754	0.000463	0.001592	0.001723	0.001356	0.002589	0.003411
753	0.000545	0.001309	0.001733	0.000969	0.002495	0.003161
752	0.000303	0.001498	0.001816	0.001217	0.002688	0.003125
751	0.000494	0.001361	0.001679	0.001228	0.002719	0.003163
750	0.000183	0.001068	0.001588	0.001059	0.002441	0.003107
749	0.000382	0.001427	0.001892	0.001039	0.002559	0.003255
748	0.000464	0.001238	0.001747	0.001311	0.002641	0.003348
747	0.000281	0.001407	0.001586	0.001169	0.002574	0.003158
746	0.000209	0.001256	0.001566	0.001045	0.002371	0.003087
745	0.000239	0.001327	0.001545	0.001073	0.002569	0.003127
744	0.00022	0.001205	0.001474	0.001101	0.0024	0.003163
743	0.000219	0.001395	0.001575	0.001238	0.002624	0.003072
742	0.000327	0.001316	0.001605	0.001022	0.002505	0.003118
741	0.000113	0.001213	0.001452	0.001024	0.002587	0.002779
740	-2.53E-05	0.001281	0.001505	0.000855	0.002591	0.003108
739	0.000261	0.001441	0.001644	0.001157	0.002717	0.002977
738	0.000286	0.001333	0.001386	0.00114	0.002651	0.003126
737	0.000208	0.001328	0.001472	0.001101	0.002653	0.003127
736	0.000284	0.001442	0.001485	0.001124	0.002447	0.003263
735	0.000408	0.001456	0.001635	0.001219	0.002815	0.003162
734	0.000293	0.001391	0.001443	0.001095	0.002671	0.003302
733	0.000403	0.001429	0.001615	0.001193	0.002704	0.00329
732	0.000472	0.001492	0.001593	0.001187	0.002836	0.003415
731	0.000531	0.001514	0.00169	0.001215	0.002662	0.003328
730	0.00039	0.001263	0.001538	0.00112	0.002585	0.002972
729	0.000493	0.001598	0.001835	0.00123	0.002825	0.003365
728	0.000383	0.001457	0.00183	0.001221	0.00283	0.003384
727	0.000326	0.001387	0.001678	0.00123	0.002699	0.003153
726	0.00032	0.001529	0.001802	0.001183	0.002836	0.003318
725	0.000472	0.001677	0.001748	0.001352	0.00289	0.003325
724	0.000288	0.001693	0.001909	0.001254	0.002926	0.003232
723	0.000384	0.001497	0.001807	0.001385	0.002952	0.003424
722	0.000373	0.00165	0.001912	0.001388	0.003052	0.003324
721	0.00046	0.001713	0.001756	0.001349	0.002958	0.003256
720	0.000158	0.001602	0.001676	0.001262	0.002761	0.003308
719	0.000421	0.001617	0.001766	0.001275	0.002928	0.003391
718	0.000331	0.001528	0.001787	0.001219	0.003143	0.003401
717	0.000501	0.001577	0.001787	0.00122	0.003092	0.003435
716	0.000326	0.00149	0.001722	0.001296	0.002926	0.003397
715	0.000591	0.001692	0.002003	0.001638	0.003291	0.00348
714	0.000317	0.001651	0.001856	0.001379	0.002896	0.003513
713	0.000539	0.001749	0.00168	0.00119	0.003201	0.003442
712	0.000436	0.00153	0.001889	0.001341	0.003238	0.003477
711	0.000608	0.001651	0.002024	0.001498	0.00335	0.003372
710	0.000627	0.001728	0.002153	0.001333	0.003351	0.003711
709	0.000429	0.001564	0.001718	0.00114	0.00307	0.003468
708	0.000501	0.001572	0.001856	0.001314	0.003218	0.003482

EXCLI Journal 2020;19:48-70 – ISSN 1611-2156
Received: October 03, 2019, accepted: December 19, 2019, published: January 03, 2020

Wavelength (nm)	***Absorption intensity***					
	**concentration of CT-DNA (M)**					
	A	*B*	*C*	*D*	*E*	*F*
	0.00	$2.66x10^{-5}$	$6.21x10^{-5}$	$7.98x10^{-5}$	$1.33x10^{-4}$	$1.73x10^{-4}$
707	0.000681	0.001854	0.002111	0.00151	0.003518	0.003599
706	0.000521	0.001768	0.001931	0.001591	0.003531	0.003648
705	0.000548	0.001794	0.002129	0.001419	0.003303	0.003463
704	0.000498	0.00169	0.001946	0.0013	0.003296	0.003556
703	0.000533	0.001903	0.002119	0.001453	0.003546	0.003617
702	0.000506	0.001764	0.0021	0.001388	0.003416	0.00363
701	0.000486	0.001626	0.002047	0.001317	0.003446	0.00369
700	0.000438	0.001838	0.002083	0.001517	0.003297	0.003517
699	0.000507	0.001746	0.002011	0.001578	0.003534	0.003543
698	0.00053	0.001956	0.002168	0.001471	0.00349	0.003713
697	0.000467	0.001659	0.00189	0.001396	0.003314	0.003379
696	0.000422	0.001874	0.001964	0.001429	0.003497	0.00363
695	0.000442	0.00184	0.001845	0.001329	0.003465	0.003513
694	0.000639	0.001877	0.00209	0.001601	0.003673	0.003741
693	0.000656	0.001798	0.002095	0.001579	0.00359	0.003619
692	0.000552	0.001736	0.002079	0.001425	0.003474	0.003476
691	0.000614	0.0018	0.002123	0.001564	0.003565	0.00378
690	0.000453	0.001658	0.001934	0.001563	0.003661	0.003746
689	0.000604	0.001762	0.002044	0.001411	0.003545	0.003702
688	0.000616	0.001827	0.002223	0.001362	0.003656	0.003775
687	0.000645	0.001903	0.002071	0.001444	0.003498	0.003634
686	0.000531	0.001854	0.002018	0.001347	0.003677	0.003838
685	0.000523	0.001837	0.002061	0.001389	0.003721	0.003703
684	0.000472	0.001948	0.002047	0.001549	0.003614	0.003658
683	0.000668	0.001983	0.002268	0.001618	0.003776	0.003708
682	0.000682	0.002043	0.002264	0.001617	0.00364	0.003815
681	0.000684	0.001916	0.002078	0.001564	0.003656	0.003789
680	0.000369	0.001867	0.002074	0.001513	0.003617	0.003682
679	0.000716	0.002058	0.002167	0.0016	0.003712	0.003807
678	0.000509	0.00189	0.002234	0.001315	0.003665	0.00375
677	0.000612	0.001987	0.00218	0.001582	0.003892	0.003833
676	0.000527	0.001851	0.0021	0.00147	0.003688	0.003771
675	0.000435	0.001847	0.002183	0.001416	0.003631	0.003792
674	0.000751	0.001941	0.00225	0.00164	0.003936	0.00383
673	0.000747	0.00196	0.002145	0.00161	0.003728	0.003848
672	0.000542	0.001773	0.00209	0.001438	0.00368	0.003719
671	0.000514	0.002134	0.002077	0.001597	0.003892	0.003821
670	0.000686	0.001965	0.002148	0.00155	0.003901	0.004024
669	0.000686	0.00208	0.002341	0.001654	0.003759	0.004038
668	0.000579	0.001872	0.00217	0.001567	0.003812	0.003884
667	0.000597	0.002004	0.002269	0.001529	0.003836	0.003828
666	0.000681	0.002049	0.002092	0.00155	0.003817	0.003855
665	0.000619	0.001996	0.002239	0.001576	0.003875	0.003905
664	0.00059	0.002001	0.002317	0.00159	0.003865	0.003822
663	0.000675	0.002003	0.00232	0.001546	0.003853	0.004004
662	0.000758	0.002183	0.002384	0.001791	0.003961	0.004039
661	0.000573	0.002069	0.002211	0.001536	0.003783	0.003934
660	0.000584	0.002027	0.002314	0.00147	0.003784	0.003941

EXCLI Journal 2020;19:48-70 – ISSN 1611-2156
Received: October 03, 2019, accepted: December 19, 2019, published: January 03, 2020

Wavelength (nm)	***Absorption intensity***					
	**concentration of CT-DNA (M)**					
	A	*B*	*C*	*D*	*E*	*F*
	0.00	$2.66x10^{-5}$	$6.21x10^{-5}$	$7.98x10^{-5}$	$1.33x10^{-4}$	$1.73x10^{-4}$
659	0.000468	0.00206	0.002364	0.001689	0.0038	0.003883
658	0.000504	0.002025	0.002156	0.001626	0.003718	0.003879
657	0.00065	0.002084	0.002281	0.001641	0.003998	0.004025
656	0.000547	0.002001	0.00229	0.001633	0.003792	0.00399
655	0.000562	0.002011	0.002245	0.001641	0.003951	0.003867
654	0.000678	0.002042	0.002372	0.001576	0.003932	0.003982
653	0.000693	0.002161	0.002363	0.00179	0.00403	0.003877
652	0.00075	0.002192	0.002305	0.001681	0.004025	0.004117
651	0.000725	0.002175	0.00238	0.00166	0.003905	0.003924
650	0.000711	0.00213	0.00231	0.00174	0.003941	0.004165
649	0.000665	0.001961	0.002092	0.001676	0.00384	0.003991
648	0.000675	0.00218	0.002405	0.001611	0.003969	0.004189
647	0.000578	0.002024	0.002156	0.001522	0.003955	0.004135
646	0.000682	0.002068	0.002299	0.001668	0.003867	0.00403
645	0.00061	0.002072	0.00234	0.001666	0.003868	0.004008
644	0.000563	0.001938	0.002341	0.001661	0.004041	0.004111
643	0.000712	0.002205	0.002515	0.001838	0.003995	0.00412
642	0.000583	0.002104	0.002263	0.001549	0.003827	0.004013
641	0.000681	0.0021	0.002388	0.001668	0.003889	0.003983
640	0.000583	0.002134	0.002308	0.001647	0.003856	0.004035
639	0.000583	0.002036	0.00224	0.00173	0.003902	0.00402
638	0.000418	0.002095	0.002208	0.001651	0.003959	0.004
637	0.000433	0.002052	0.002241	0.001592	0.003955	0.003882
636	0.000572	0.002143	0.002365	0.001713	0.003909	0.003966
635	0.000581	0.002217	0.002178	0.001725	0.003894	0.004074
634	0.000392	0.002003	0.002133	0.00164	0.003872	0.003892
633	0.000493	0.001908	0.002239	0.00162	0.003812	0.003994
632	0.00058	0.002179	0.002388	0.00182	0.003859	0.004174
631	0.000581	0.002107	0.002324	0.001693	0.003877	0.004059
630	0.000716	0.00217	0.002286	0.001644	0.003985	0.004136
629	0.0006	0.002165	0.002274	0.001663	0.003858	0.004082
628	0.0005	0.002086	0.002367	0.001605	0.003807	0.004094
627	0.000688	0.002188	0.002469	0.001778	0.003926	0.004142
626	0.000689	0.002128	0.002497	0.001773	0.00394	0.004281
625	0.000477	0.00204	0.002243	0.001638	0.003741	0.00412
624	0.000719	0.002314	0.002525	0.001849	0.004052	0.004393
623	0.000647	0.002151	0.002392	0.001828	0.003947	0.004216
622	0.000661	0.002301	0.002495	0.001715	0.003868	0.004261
621	0.000534	0.00212	0.002367	0.001831	0.003857	0.004123
620	0.000516	0.002312	0.002419	0.001753	0.003885	0.004214
619	0.000451	0.002234	0.00244	0.001741	0.003935	0.004122
618	0.000456	0.002205	0.00228	0.001715	0.003702	0.004004
617	0.000467	0.002297	0.002319	0.001766	0.003796	0.004284
616	0.000321	0.002299	0.002448	0.001758	0.003808	0.004171
615	0.000509	0.002176	0.002376	0.001745	0.003817	0.004101
614	0.000533	0.002204	0.002438	0.001865	0.003783	0.004262
613	0.000526	0.002111	0.002298	0.001769	0.003747	0.004171
612	0.000449	0.002114	0.002383	0.001719	0.003794	0.004133

Wavelength (nm)	***Absorption intensity***					
	**concentration of CT-DNA (M)**					
	A	*B*	*C*	*D*	*E*	*F*
	0.00	$2.66x10^{-5}$	$6.21x10^{-5}$	$7.98x10^{-5}$	$1.33x10^{-4}$	$1.73x10^{-4}$
611	0.000589	0.002293	0.002412	0.001771	0.003916	0.004319
610	0.000533	0.002194	0.002442	0.001845	0.00389	0.004282
609	0.000614	0.002141	0.002453	0.001843	0.003729	0.004198
608	0.000491	0.002152	0.002372	0.001725	0.003787	0.004149
607	0.000578	0.002102	0.002379	0.00173	0.003803	0.004352
606	0.00055	0.002226	0.002414	0.001726	0.003776	0.004305
605	0.000614	0.002374	0.00265	0.00189	0.003906	0.004479
604	0.000546	0.002305	0.002551	0.00172	0.003811	0.00437
603	0.000514	0.002272	0.002567	0.001824	0.00378	0.004403
602	0.000441	0.00235	0.002531	0.001776	0.003699	0.004284
601	0.000506	0.002419	0.002694	0.001944	0.00382	0.00428
600	0.000436	0.002326	0.002541	0.001784	0.00368	0.004319
599	0.000511	0.002474	0.002697	0.002036	0.003868	0.004429
598	0.000393	0.002326	0.002506	0.001869	0.003619	0.004337
597	0.00054	0.002428	0.002651	0.001961	0.003707	0.004307
596	0.000388	0.002428	0.002662	0.001827	0.003743	0.004234
595	0.000382	0.002413	0.002517	0.001947	0.003721	0.004304
594	0.000386	0.002361	0.002583	0.001908	0.003748	0.004337
593	0.00057	0.002435	0.002838	0.002084	0.003929	0.004417
592	0.000543	0.002437	0.002674	0.002017	0.003781	0.00435
591	0.000394	0.002381	0.002465	0.001853	0.003728	0.004221
590	0.000552	0.002358	0.00267	0.001868	0.003946	0.004393
589	0.000667	0.002514	0.002754	0.001957	0.00385	0.004459
588	0.00064	0.002395	0.002581	0.002053	0.003903	0.004514
587	0.000424	0.002149	0.002496	0.00182	0.003732	0.004402
586	0.000502	0.00231	0.002481	0.002016	0.003669	0.0043
585	0.000572	0.002275	0.002645	0.001966	0.003718	0.0044
584	0.000571	0.002397	0.002795	0.001874	0.003834	0.004539
583	0.000495	0.002603	0.002751	0.001997	0.003933	0.004564
582	0.000416	0.002621	0.002836	0.002103	0.003917	0.0046
581	0.000574	0.002491	0.002807	0.001999	0.00389	0.004583
580	0.00045	0.002503	0.002823	0.002027	0.00391	0.004468
579	0.000387	0.002495	0.002629	0.002003	0.003716	0.004379
578	0.000402	0.002499	0.002714	0.002115	0.003869	0.004438
577	0.000485	0.0026	0.002793	0.00203	0.003935	0.004475
576	0.000443	0.002474	0.002795	0.002036	0.003902	0.004503
575	0.000423	0.002512	0.002824	0.002014	0.003856	0.004508
574	0.000611	0.002738	0.002889	0.002179	0.003986	0.004666
573	0.000498	0.002506	0.002681	0.002073	0.003772	0.004606
572	0.000594	0.002543	0.002766	0.002166	0.003898	0.004513
571	0.000584	0.002633	0.002892	0.002196	0.004064	0.004599
570	0.000433	0.002597	0.002821	0.002158	0.004058	0.004476
569	0.0006	0.002605	0.002771	0.002153	0.004117	0.004619
568	0.000357	0.002603	0.002712	0.002096	0.003979	0.004538
567	0.000508	0.002764	0.002869	0.002205	0.004024	0.004577
566	0.000617	0.002699	0.002825	0.002191	0.004014	0.004652
565	0.000632	0.002654	0.002847	0.002323	0.004089	0.004707
564	0.000658	0.002678	0.002924	0.002148	0.00404	0.00474

Wavelength (nm)	***Absorption intensity***					
	**concentration of CT-DNA (M)**					
	A	*B*	*C*	*D*	*E*	*F*
	0.00	2.66x10^{-5}	6.21x10^{-5}	7.98x10^{-5}	1.33x10^{-4}	1.73x10^{-4}
563	0.00055	0.002775	0.002885	0.002242	0.004163	0.004732
562	0.000542	0.00263	0.002903	0.002322	0.004142	0.004684
561	0.000599	0.002733	0.003013	0.002387	0.003987	0.004694
560	0.000634	0.002804	0.003048	0.002344	0.004083	0.004813
559	0.000457	0.002618	0.003016	0.002165	0.003911	0.004737
558	0.000637	0.002768	0.003129	0.002394	0.004106	0.004942
557	0.000679	0.002607	0.002953	0.002341	0.003968	0.004638
556	0.000689	0.002824	0.003216	0.00239	0.004157	0.004788
555	0.000462	0.002746	0.002905	0.002229	0.003992	0.004742
554	0.000378	0.002583	0.002975	0.00219	0.003979	0.004663
553	0.000373	0.00252	0.002993	0.002277	0.003936	0.0046
552	0.000482	0.002736	0.00306	0.002381	0.004047	0.004733
551	0.000316	0.002682	0.003078	0.002342	0.004063	0.004673
550	0.000693	0.002837	0.003172	0.002482	0.004285	0.004803
549	0.000648	0.002831	0.003061	0.002425	0.004033	0.00484
548	0.000596	0.002702	0.003093	0.002345	0.004065	0.004717
547	0.000625	0.002665	0.002998	0.002312	0.004104	0.004886
546	0.000522	0.002811	0.002989	0.002345	0.00413	0.004725
545	0.000517	0.002901	0.003237	0.002439	0.004191	0.004852
544	0.000627	0.002862	0.003231	0.002553	0.004309	0.004994
543	0.000657	0.002885	0.003234	0.002509	0.00427	0.004959
542	0.000515	0.002817	0.003191	0.002469	0.004134	0.004837
541	0.000589	0.002708	0.003166	0.002387	0.00414	0.004934
540	0.000664	0.002914	0.003406	0.002516	0.004246	0.005203
539	0.000697	0.002864	0.003277	0.002545	0.004279	0.005025
538	0.000602	0.002905	0.003214	0.002457	0.004156	0.004918
537	0.00056	0.002946	0.003367	0.002472	0.004222	0.004942
536	0.000543	0.002943	0.003209	0.002546	0.00416	0.004967
535	0.000621	0.00293	0.003284	0.002481	0.004315	0.004872
534	0.000666	0.002975	0.003391	0.002585	0.00433	0.005062
533	0.000616	0.00289	0.003273	0.002603	0.004145	0.004977
532	0.000558	0.002967	0.003221	0.002539	0.004282	0.004978
531	0.000561	0.003089	0.003415	0.002746	0.004244	0.00505
530	0.000654	0.003005	0.00328	0.00258	0.004257	0.004997
529	0.000569	0.003008	0.003231	0.002624	0.004259	0.005166
528	0.000708	0.00305	0.00342	0.0027	0.004375	0.005164
527	0.000632	0.003047	0.003301	0.002548	0.004264	0.005113
526	0.000636	0.002972	0.003421	0.002689	0.004274	0.005048
525	0.000816	0.003218	0.003495	0.002867	0.004485	0.005339
524	0.000713	0.003082	0.003315	0.002625	0.004239	0.00539
523	0.000795	0.003012	0.003465	0.002713	0.004537	0.00535
522	0.000708	0.003012	0.003411	0.002652	0.00437	0.005227
521	0.000789	0.003084	0.003591	0.002702	0.004443	0.005404
520	0.000555	0.003004	0.003458	0.002678	0.004317	0.005383
519	0.000665	0.003114	0.003464	0.002785	0.004417	0.005403
518	0.000923	0.003264	0.003646	0.002893	0.004577	0.005452
517	0.000664	0.003108	0.003602	0.002696	0.004529	0.005439
516	0.000569	0.002951	0.003468	0.002655	0.004252	0.005208

EXCLI Journal 2020;19:48-70 – ISSN 1611-2156
Received: October 03, 2019, accepted: December 19, 2019, published: January 03, 2020

**Wavelength (nm)**	***Absorption intensity***					
	**concentration of CT-DNA (M)**					
	A	*B*	*C*	*D*	*E*	*F*
	0.00	$2.66x10^{-5}$	$6.21x10^{-5}$	$7.98x10^{-5}$	$1.33x10^{-4}$	$1.73x10^{-4}$
515	0.00083	0.003186	0.003725	0.002877	0.004484	0.005366
514	0.000636	0.003215	0.003416	0.002764	0.0044	0.005286
513	0.00078	0.003189	0.003663	0.003018	0.004601	0.005421
512	0.000641	0.003122	0.003628	0.0029	0.0045	0.005232
511	0.000675	0.003194	0.003631	0.003052	0.26234	0.005168
510	0.000718	0.00301	0.003439	0.002787	0.004305	0.005103
509	0.0008	0.003046	0.003435	0.002731	0.004345	0.005246
508	0.0007	0.00288	0.003331	0.002738	0.004293	0.005187
507	0.000537	0.002862	0.003426	0.00271	0.004288	0.005131
506	0.000538	0.002835	0.00339	0.00261	0.004283	0.005138
505	0.000394	0.002806	0.003237	0.002612	0.004192	0.005078
504	0.000395	0.002857	0.003329	0.002735	0.004202	0.005314
503	0.00061	0.002835	0.003286	0.00261	0.004343	0.005259
502	0.00073	0.003055	0.003455	0.0028	0.004398	0.005437
501	0.000859	0.002937	0.003499	0.002743	0.004451	0.005354
500	0.000969	0.003195	0.003695	0.003002	0.004533	0.005659
499	0.000894	0.003287	0.003776	0.003104	0.004458	0.005438
498	0.00082	0.003428	0.003956	0.003124	0.004718	0.005533
497	0.000777	0.003305	0.003953	0.003154	0.004803	0.005788
496	0.000716	0.003303	0.003929	0.003163	0.004746	0.005707
495	0.000522	0.003168	0.00368	0.002999	0.004575	0.005531
494	0.000599	0.003144	0.003724	0.003075	0.004704	0.005787
493	0.000656	0.003149	0.003667	0.003041	0.004642	0.00557
492	0.000691	0.00327	0.003854	0.003202	0.004775	0.005728
491	0.000697	0.003183	0.003872	0.003184	0.00463	0.005637
490	0.000558	0.003322	0.003821	0.003157	0.004742	0.005733
489	0.000834	0.00333	0.003924	0.003312	0.004761	0.005826
488	0.000616	0.003359	0.003853	0.003234	0.004782	0.005744
487	0.00088	0.003272	0.00384	0.003225	0.004832	0.005847
486	0.00078	0.003217	0.003734	0.003189	0.004807	0.005758
485	0.000746	0.003456	0.003827	0.003228	0.004933	0.005851
484	0.000867	0.003364	0.003895	0.00327	0.004977	0.006043
483	0.001012	0.003387	0.003964	0.003464	0.005193	0.006018
482	0.000897	0.003348	0.00398	0.003265	0.004988	0.005952
481	0.000837	0.003432	0.003969	0.00335	0.005078	0.006031
480	0.000799	0.00344	0.004023	0.003282	0.005141	0.006149
479	0.000808	0.003423	0.004025	0.003331	0.005116	0.006078
478	0.000865	0.003541	0.004107	0.003477	0.00519	0.006163
477	0.000858	0.003516	0.003961	0.003414	0.005114	0.00611
476	0.000785	0.003598	0.004093	0.003582	0.005224	0.006273
475	0.000934	0.003481	0.00418	0.003521	0.005224	0.006222
474	0.000779	0.003414	0.004042	0.003484	0.005027	0.005956
473	0.000832	0.003562	0.004294	0.003585	0.005271	0.0062
472	0.000685	0.003527	0.004061	0.003482	0.005177	0.006245
471	0.000846	0.003573	0.004165	0.003667	0.005234	0.006146
470	0.000877	0.003621	0.004119	0.003664	0.005284	0.263364
469	0.000623	0.003192	0.003767	0.003154	0.004949	0.005772
468	0.000743	0.003441	0.004026	0.00357	0.005193	0.006174

Wavelength (nm)	***Absorption intensity***					
	**concentration of CT-DNA (M)**					
	A	*B*	*C*	*D*	*E*	*F*
	0.00	$2.66x10^{-5}$	$6.21x10^{-5}$	$7.98x10^{-5}$	$1.33x10^{-4}$	$1.73x10^{-4}$
467	0.001089	0.003606	0.004221	0.003726	0.005415	0.006379
466	0.000905	0.003543	0.004187	0.003664	0.005324	0.006313
465	0.000972	0.00357	0.004246	0.003758	0.005399	0.006336
464	0.001063	0.003533	0.004042	0.003667	0.005341	0.006291
463	0.000889	0.00376	0.004197	0.003773	0.005391	0.006376
462	0.000935	0.003615	0.004249	0.003719	0.005493	0.006513
461	0.001002	0.00367	0.004174	0.003798	0.005558	0.006464
460	0.000897	0.003501	0.004243	0.00377	0.005364	0.006593
459	0.001071	0.00376	0.004393	0.003892	0.005491	0.006675
458	0.00095	0.00362	0.004247	0.003754	0.005545	0.006551
457	0.000898	0.003714	0.004275	0.003764	0.005419	0.006601
456	0.000944	0.003758	0.004492	0.004098	0.005623	0.006743
455	0.000953	0.003757	0.00434	0.003845	0.005601	0.006549
454	0.000936	0.003885	0.004349	0.003899	0.005694	0.006723
453	0.00093	0.003829	0.004471	0.004078	0.005605	0.006729
452	0.000812	0.003883	0.004356	0.004034	0.005659	0.006767
451	0.00077	0.003791	0.004241	0.003992	0.005555	0.006508
450	0.000928	0.003931	0.00446	0.004172	0.005607	0.006783
449	0.000811	0.003747	0.004344	0.003978	0.005653	0.006583
448	0.00102	0.003925	0.004579	0.00412	0.005974	0.006872
447	0.001028	0.00379	0.004423	0.004206	0.0057	0.006799
446	0.001062	0.004014	0.004573	0.004055	0.005847	0.006928
445	0.001006	0.003838	0.004493	0.004284	0.005987	0.006998
444	0.001183	0.003957	0.004524	0.004381	0.00599	0.006957
443	0.001112	0.003897	0.004389	0.004285	0.005907	0.006935
442	0.001093	0.00386	0.004529	0.004156	0.005951	0.007149
441	0.001198	0.00392	0.004622	0.004246	0.006029	0.007128
440	0.0011	0.004031	0.004507	0.004346	0.005897	0.007291
439	0.000999	0.003851	0.004523	0.004114	0.005909	0.007037
438	0.000975	0.003867	0.004501	0.004199	0.00596	0.007175
437	0.001128	0.004088	0.004807	0.004517	0.006134	0.007299
436	0.001051	0.003946	0.004725	0.00435	0.006102	0.007315
435	0.001021	0.004109	0.00474	0.004503	0.006088	0.007297
434	0.000988	0.003949	0.004685	0.004545	0.005875	0.007252
433	0.000953	0.004105	0.00472	0.004492	0.006052	0.007343
432	0.000919	0.004003	0.004754	0.004552	0.0061	0.007376
431	0.001049	0.004169	0.004812	0.004638	0.006174	0.007494
430	0.001045	0.004009	0.00463	0.004544	0.005993	0.007343
429	0.001033	0.003987	0.004661	0.004467	0.006041	0.007484
428	0.001016	0.004035	0.004734	0.004614	0.006268	0.007454
427	0.00109	0.004012	0.004783	0.004615	0.00623	0.00734
426	0.001088	0.004246	0.00477	0.004824	0.006604	0.007536
425	0.00142	0.004134	0.004654	0.004755	0.006303	0.007547
424	0.001133	0.004028	0.004794	0.004754	0.00628	0.007499
423	0.001201	0.004247	0.004983	0.004845	0.006523	0.007584
422	0.00126	0.004124	0.004877	0.00479	0.006361	0.007656
421	0.001447	0.00421	0.005071	0.005156	0.00661	0.007853
420	0.001386	0.004236	0.004949	0.004825	0.006592	0.007919

Wavelength (nm)	***Absorption intensity***					
	**concentration of CT-DNA (M)**					
	A	*B*	*C*	*D*	*E*	*F*
	0.00	$2.66x10^{-5}$	$6.21x10^{-5}$	$7.98x10^{-5}$	$1.33x10^{-4}$	$1.73x10^{-4}$
419	0.001571	0.004412	0.005171	0.005171	0.006711	0.007953
418	0.001507	0.00428	0.005037	0.005141	0.006662	0.007887
417	0.001214	0.004265	0.005029	0.005047	0.006623	0.007856
416	0.001294	0.004483	0.005272	0.005272	0.006862	0.008061
415	0.001409	0.004468	0.005317	0.005273	0.006855	0.008096
414	0.001265	0.00444	0.005131	0.005113	0.00684	0.008089
413	0.001311	0.004499	0.005133	0.005392	0.00687	0.008191
412	0.001163	0.004365	0.005268	0.00555	0.00684	0.008169
411	0.001124	0.00436	0.004984	0.005234	0.006842	0.008107
410	0.001264	0.004512	0.005186	0.00526	0.006884	0.008146
409	0.001317	0.004566	0.005216	0.00536	0.006747	0.008309
408	0.001275	0.004494	0.005121	0.005446	0.006871	0.008477
407	0.001028	0.004452	0.00489	0.005346	0.006711	0.007926
406	0.001242	0.004324	0.004999	0.005381	0.006908	0.00822
405	0.001521	0.004565	0.005246	0.005835	0.007038	0.008453
404	0.001668	0.004726	0.005498	0.00583	0.007049	0.008594
403	0.001562	0.004553	0.005277	0.005717	0.007067	0.008596
402	0.001441	0.004442	0.0054	0.005782	0.007325	0.008562
401	0.001386	0.004647	0.005396	0.005777	0.007245	0.008715
400	0.001461	0.004475	0.005242	0.005766	0.007212	0.008361
399	0.001551	0.004714	0.005368	0.005697	0.007236	0.008466
398	0.001376	0.004801	0.0055	0.005915	0.007484	0.008611
397	0.001353	0.004582	0.005395	0.005809	0.007205	0.008484
396	0.001472	0.005073	0.005669	0.006286	0.007588	0.00888
395	0.001506	0.004897	0.005621	0.006189	0.007466	0.008732
394	0.001568	0.004899	0.005735	0.006307	0.007614	0.008878
393	0.001611	0.005092	0.005752	0.006447	0.007846	0.009006
392	0.001413	0.004884	0.005765	0.006457	0.007736	0.009109
391	0.00154	0.005436	0.006069	0.006681	0.007888	0.009293
390	0.000974	0.004219	0.005031	0.005898	0.007116	0.008317
389	0.001555	0.004809	0.005913	0.00656	0.007748	0.009176
388	0.00159	0.00508	0.005681	0.006385	0.007566	0.009189
387	0.001469	0.0051	0.005715	0.006532	0.007619	0.00921
386	0.001264	0.004523	0.005708	0.006544	0.007579	0.009068
385	0.001612	0.005042	0.005787	0.006887	0.007761	0.009217
384	0.001827	0.004958	0.005829	0.006519	0.007749	0.009066
383	0.001882	0.004902	0.005871	0.006863	0.007934	0.009365
382	0.00188	0.005099	0.005788	0.006923	0.007916	0.009392
381	0.002032	0.005229	0.006019	0.006985	0.008321	0.009468
380	0.001633	0.005251	0.006164	0.006912	0.008391	0.00962
379	0.001931	0.005102	0.006153	0.006752	0.008073	0.009613
378	0.002051	0.005279	0.006448	0.007456	0.008251	0.00962
377	0.001598	0.005207	0.006139	0.007014	0.00801	0.009603
376	0.001578	0.005595	0.006309	0.007259	0.008286	0.010008
375	0.001863	0.004686	0.006253	0.007424	0.008247	0.009628
374	0.001682	0.005113	0.005998	0.00723	0.008032	0.009545
373	0.001971	0.005706	0.006225	0.007976	0.008328	0.010029
372	0.001984	0.005672	0.006572	0.007654	0.008873	0.010304

EXCLI Journal 2020;19:48-70 – ISSN 1611-2156
Received: October 03, 2019, accepted: December 19, 2019, published: January 03, 2020

Absorption intensity						
**Wavelength (nm)**	**concentration of CT-DNA (M)**					
	A	*B*	*C*	*D*	*E*	*F*
	0.00	$2.66x10^{-5}$	$6.21x10^{-5}$	$7.98x10^{-5}$	$1.33x10^{-4}$	$1.73x10^{-4}$
371	0.001768	0.00499	0.00611	0.007082	0.008411	0.0096
370	0.001696	0.005318	0.006306	0.007614	0.008246	0.01007
369	0.001267	0.00538	0.006253	0.007245	0.008406	0.009593
368	0.001997	0.005741	0.006502	0.007941	0.009066	0.010585
367	0.001957	0.005615	0.005871	0.007938	0.0081	0.009857
366	0.001852	0.005476	0.006505	0.007918	0.008967	0.010629
365	0.002088	0.005631	0.006396	0.007922	0.009119	0.010414
364	0.002112	0.00546	0.006533	0.007638	0.008868	0.010226
363	0.001887	0.005126	0.006014	0.008088	0.008833	0.010516
362	0.002784	0.006713	0.007183	0.008637	0.009404	0.011282
361	0.000608	0.004045	0.004741	0.00643	0.00724	0.00897
360	0.002325	0.005769	0.006696	0.008431	0.009067	0.01035
359	0.002053	0.004991	0.00628	0.008884	0.009526	0.011159
358	0.002317	0.005899	0.006898	0.008079	0.009056	0.011301
357	0.002357	0.005641	0.007403	0.007889	0.008773	0.010829
356	0.002146	0.005669	0.006587	0.008437	0.008764	0.011338
355	0.003133	0.005206	0.007928	0.008701	0.009924	0.011373
354	0.002199	0.005316	0.007296	0.009151	0.009236	0.010705
353	0.002818	0.005758	0.007777	0.00904	0.009672	0.012393
352	0.0023	0.005694	0.006975	0.009121	0.009132	0.011357
351	0.00235	0.005056	0.007591	0.010071	0.008947	0.011713
350	0.000828	0.00565	0.006821	0.009152	0.009922	0.011574
349	0.002493	0.007479	0.009029	0.009657	0.009033	0.012318
348	0.001548	0.005552	0.006397	0.0092	0.00991	0.011264
347	0.001871	0.005519	0.006916	0.009545	0.010212	0.011452
346	0.001657	0.005489	0.006896	0.009513	0.010544	0.011403
345	0.002123	0.007011	0.007635	0.010134	0.010993	0.011969
344	0.002588	0.006349	0.007234	0.00976	0.010666	0.012175
343	0.00252	0.006271	0.007565	0.010879	0.010951	0.011923
342	0.002163	0.005945	0.006795	0.009513	0.010418	0.011688
341	0.002565	0.005927	0.007366	0.010053	0.010781	0.012482
340	0.002483	0.006572	0.007227	0.010215	0.010911	0.012283
339	0.001852	0.006207	0.007029	0.009894	0.010305	0.011698
338	0.002307	0.006419	0.007187	0.009948	0.010971	0.011855
337	0.002422	0.006302	0.007207	0.009978	0.010888	0.012319
336	0.002456	0.006293	0.007537	0.010281	0.011249	0.013108
335	0.002352	0.006137	0.007727	0.010038	0.01066	0.012965
334	0.002159	0.006435	0.007941	0.010645	0.011646	0.012807
333	0.002109	0.006367	0.007572	0.01072	0.011156	0.012668
332	0.002894	0.006856	0.008066	0.011055	0.011438	0.013401
331	0.002405	0.00642	0.008342	0.011029	0.01154	0.013159
330	0.002975	0.006608	0.008557	0.011116	0.011972	0.0135
329	0.002362	0.007107	0.008266	0.01108	0.011892	0.01355
328	0.002227	0.006427	0.008076	0.010676	0.011232	0.012982
327	0.003275	0.007595	0.009138	0.011396	0.012614	0.014073
326	0.002515	0.006786	0.008105	0.011207	0.011679	0.013339
325	0.002861	0.007391	0.008549	0.011699	0.012484	0.014121
324	0.002933	0.007277	0.008432	0.011828	0.012891	0.014195

EXCLI Journal 2020;19:48-70 – ISSN 1611-2156
Received: October 03, 2019, accepted: December 19, 2019, published: January 03, 2020

Wavelength (nm)	***Absorption intensity***					
	**concentration of CT-DNA (M)**					
	A	*B*	*C*	*D*	*E*	*F*
	0.00	2.66×10^{-5}	6.21×10^{-5}	7.98×10^{-5}	1.33×10^{-4}	1.73×10^{-4}
323	0.00295	0.007194	0.008991	0.011609	0.012512	0.0143
322	0.002772	0.007588	0.00918	0.012341	0.012304	0.014762
321	0.003261	0.007974	0.009267	0.012755	0.012922	0.015262
320	0.003195	0.007742	0.00975	0.012554	0.013671	0.015072
319	0.003199	0.007903	0.009875	0.01283	0.013036	0.01544
318	0.003511	0.008227	0.009797	0.012824	0.013266	0.015055
317	0.003056	0.007754	0.00972	0.012929	0.013524	0.01546
316	0.003309	0.008085	0.010088	0.013315	0.014065	0.015587
315	0.003728	0.008245	0.010201	0.013584	0.014233	0.015943
314	0.003485	0.007718	0.009954	0.013185	0.014374	0.016063
313	0.003302	0.007647	0.010426	0.013523	0.014192	0.016318
312	0.003534	0.008773	0.010668	0.014247	0.014832	0.017259
311	0.002871	0.007914	0.010252	0.014195	0.014778	0.017289
310	0.003059	0.008536	0.010972	0.014418	0.01506	0.017659
309	0.003462	0.008925	0.011587	0.01545	0.016658	0.019097
308	0.003533	0.008964	0.012649	0.016005	0.017526	0.020496
307	0.003548	0.009228	0.013054	0.01656	0.018843	0.021354
306	0.004255	0.009985	0.014678	0.018272	0.020289	0.023187
305	0.004409	0.010394	0.015805	0.019723	0.022869	0.025739
304	0.003653	0.010592	0.016547	0.020929	0.024739	0.028165
303	0.00419	0.011031	0.018675	0.022804	0.02814	0.031789
302	0.004064	0.012005	0.021109	0.025758	0.032121	0.03653
301	0.003973	0.012688	0.023559	0.028817	0.037349	0.041713
300	0.003807	0.013696	0.027155	0.032796	0.043436	0.048455
299	0.003269	0.014517	0.031163	0.037971	0.051563	0.057495
298	0.004125	0.016507	0.036534	0.044802	0.061544	0.068382
297	0.00388	0.018424	0.042956	0.052364	0.073556	0.081856
296	0.004575	0.020524	0.050615	0.061387	0.08797	0.097284
295	0.004315	0.022981	0.059748	0.072204	0.104928	0.116396
294	0.004268	0.026097	0.06967	0.085136	0.124538	0.137779
293	0.004504	0.029105	0.081345	0.099572	0.147101	0.162661
292	0.004876	0.032938	0.093528	0.115366	0.170818	0.188722
291	0.004591	0.036749	0.10667	0.131614	0.197066	0.217545
290	0.004715	0.040612	0.120883	0.14943	0.22489	0.247745
289	0.004876	0.045265	0.13603	0.168699	0.253732	0.279539
288	0.004885	0.049735	0.151669	0.187499	0.283574	0.312777
287	0.005168	0.054272	0.167513	0.208141	0.315509	0.347396
286	0.005135	0.058817	0.184391	0.229168	0.348036	0.383405
285	0.004876	0.063931	0.201799	0.25071	0.381525	0.420053
284	0.005161	0.06892	0.219452	0.272874	0.416012	0.457582
283	0.004834	0.073456	0.236061	0.294848	0.450161	0.494693
282	0.00562	0.078609	0.253534	0.316546	0.482892	0.531191
281	0.005628	0.083057	0.269492	0.336323	0.514887	0.566491
280	0.005475	0.087896	0.284762	0.355869	0.544944	0.599484
279	0.005433	0.091547	0.299731	0.374975	0.574203	0.631966
278	0.005831	0.096116	0.314737	0.39424	0.603656	0.664635
277	0.005457	0.09993	0.329644	0.413341	0.633162	0.69746
276	0.005404	0.104261	0.344764	0.432009	0.663503	0.729242

Wavelength (nm)	***Absorption intensity***					
	**concentration of CT-DNA (M)**					
	A	*B*	*C*	*D*	*E*	*F*
	0.00	$2.66x10^{-5}$	$6.21x10^{-5}$	$7.98x10^{-5}$	$1.33x10^{-4}$	$1.73x10^{-4}$
275	0.005475	0.108393	0.360322	0.451682	0.693752	0.763265
274	0.00573	0.112726	0.375755	0.471421	0.72358	0.795941
273	0.005781	0.117467	0.390801	0.490827	0.753802	0.829668
272	0.006042	0.121632	0.405812	0.509817	0.78325	0.861494
271	0.006294	0.125673	0.419857	0.528131	0.811637	0.89222
270	0.006284	0.13004	0.433389	0.544913	0.837835	0.921967
269	0.006008	0.133198	0.4461	0.560917	0.862528	0.948547
268	0.006073	0.136465	0.457729	0.575629	0.885938	0.97449
267	0.006507	0.140274	0.469418	0.591261	0.909343	0.999843
266	0.006538	0.142905	0.480675	0.605394	0.930786	1.024141
265	0.006847	0.146099	0.491477	0.618596	0.95203	1.04735
264	0.006669	0.149224	0.501382	0.631912	0.972464	1.070006
263	0.006712	0.151732	0.510779	0.644397	0.990794	1.089974
262	0.006948	0.154298	0.51955	0.655005	1.007643	1.108263
261	0.006836	0.155919	0.52604	0.663862	1.021359	1.123392
260	0.007234	0.157594	0.531148	0.669906	1.030994	1.134203
259	0.007144	0.15862	0.53446	0.674433	1.037575	1.141296
258	0.007415	0.158944	0.535303	0.676054	1.040038	1.1443
257	0.007415	0.158678	0.535179	0.675482	1.039185	1.142744
256	0.007613	0.158053	0.532833	0.672503	1.034613	1.138164
255	0.00759	0.157292	0.529369	0.667889	1.027609	1.130394
254	0.007417	0.155885	0.523453	0.661395	1.017142	1.119004
253	0.007789	0.154215	0.51662	0.65276	1.003229	1.102787
252	0.007785	0.151859	0.507394	0.6415	0.986273	1.084838
251	0.007772	0.148809	0.496742	0.627965	0.964521	1.061188
250	0.008282	0.145644	0.484242	0.612425	0.940143	1.034602
249	0.007923	0.141534	0.469773	0.594044	0.911571	1.002975
248	0.008087	0.137703	0.453729	0.574681	0.881045	0.969948
247	0.008681	0.133702	0.437814	0.553535	0.848814	0.934525
246	0.008573	0.128748	0.420751	0.532311	0.815728	0.898355
245	0.00873	0.124208	0.403139	0.510265	0.781242	0.8603
244	0.008929	0.119762	0.386194	0.488921	0.7477	0.823504
243	0.008874	0.114846	0.368313	0.466707	0.713254	0.785188
242	0.009167	0.110485	0.351906	0.445648	0.68013	0.749051
241	0.009509	0.106244	0.33504	0.424231	0.64755	0.71379
240	0.009824	0.102339	0.31976	0.404923	0.616957	0.679866
239	0.01007	0.098215	0.304785	0.385862	0.587773	0.647736
238	0.010103	0.094624	0.291071	0.368813	0.561014	0.618369
237	0.010471	0.091785	0.279121	0.353537	0.537175	0.592368
236	0.010308	0.089193	0.268051	0.339744	0.516056	0.569429
235	0.011145	0.087294	0.25963	0.328362	0.4983	0.550238
234	0.01127	0.085369	0.252009	0.319224	0.483796	0.534383
233	0.011719	0.084581	0.246643	0.311965	0.472975	0.522478
232	0.01201	0.084205	0.243045	0.307375	0.465907	0.515013
231	0.011958	0.084007	0.241127	0.304902	0.461849	0.510408
230	0.012564	0.084669	0.241138	0.304806	0.461761	0.510341
229	0.012984	0.085953	0.243146	0.307002	0.465385	0.514296
228	0.013409	0.087416	0.246884	0.311382	0.471978	0.521817

EXCLI Journal 2020;19:48-70 – ISSN 1611-2156
Received: October 03, 2019, accepted: December 19, 2019, published: January 03, 2020

Absorption intensity						
**Wavelength (nm)**	**concentration of CT-DNA (M)**					
	A	*B*	*C*	*D*	*E*	*F*
	0.00	$2.66x10^{-5}$	$6.21x10^{-5}$	$7.98x10^{-5}$	$1.33x10^{-4}$	$1.73x10^{-4}$
227	0.013871	0.090001	0.252476	0.318586	0.482751	0.533944
226	0.01411	0.092373	0.260011	0.327843	0.496905	0.5498
225	0.015009	0.096166	0.269477	0.339973	0.515223	0.570165
224	0.01552	0.100084	0.281321	0.354419	0.538203	0.594914
223	0.016464	0.10544	0.296143	0.373234	0.566667	0.626536
222	0.017138	0.11099	0.312984	0.394192	0.599111	0.662522
221	0.018641	0.117881	0.332603	0.41864	0.637248	0.703882
220	0.019499	0.125092	0.353628	0.44519	0.678065	0.749122
219	0.020836	0.132972	0.376052	0.473575	0.721841	0.797394
218	0.022452	0.141159	0.399647	0.503161	0.767326	0.847509
217	0.02374	0.150128	0.423473	0.533554	0.814647	0.899742
216	0.024763	0.158406	0.447918	0.565073	0.862581	0.953234
215	0.026606	0.168267	0.473452	0.597591	0.913061	1.008077
214	0.028789	0.17769	0.499034	0.630227	0.96271	1.06353
213	0.030115	0.186884	0.524942	0.662142	1.013197	1.119202
212	0.03112	0.195106	0.549304	0.693716	1.062714	1.17242
211	0.033019	0.204902	0.574154	0.72555	1.111966	1.226947
210	0.0338	0.213822	0.600273	0.757948	1.162096	1.282866
209	0.035361	0.220102	0.621419	0.784428	1.205058	1.330964
208	0.032199	0.225009	0.642937	0.812646	1.250442	1.378313
207	0.032512	0.232985	0.665327	0.841675	1.293932	1.430244
206	0.029962	0.237386	0.685542	0.86656	1.337085	1.477988
205	0.028872	0.245367	0.708774	0.895598	1.382373	1.529211
204	0.017256	0.238179	0.713852	0.905046	1.408221	1.560536
203	0.016433	0.25452	0.741869	0.942795	1.462184	1.614185
202	0.008699	0.226735	0.725778	0.929266	1.463107	1.619191
201	0	0.243106	0.745941	0.955655	1.500836	1.666241
200	0	0.22834	0.726151	0.938649	1.48396	1.639244
